# Supplementary figures and images for: A computational model for the evaluation of complement system regulation under homeostasis, disease, and drug intervention
Source: PLoS One. 2018 Jun 6;13(6):e0198644. doi: 10.1371/journal.pone.0198644 (PMC5991421; doi:10.1371/journal.pone.0198644)

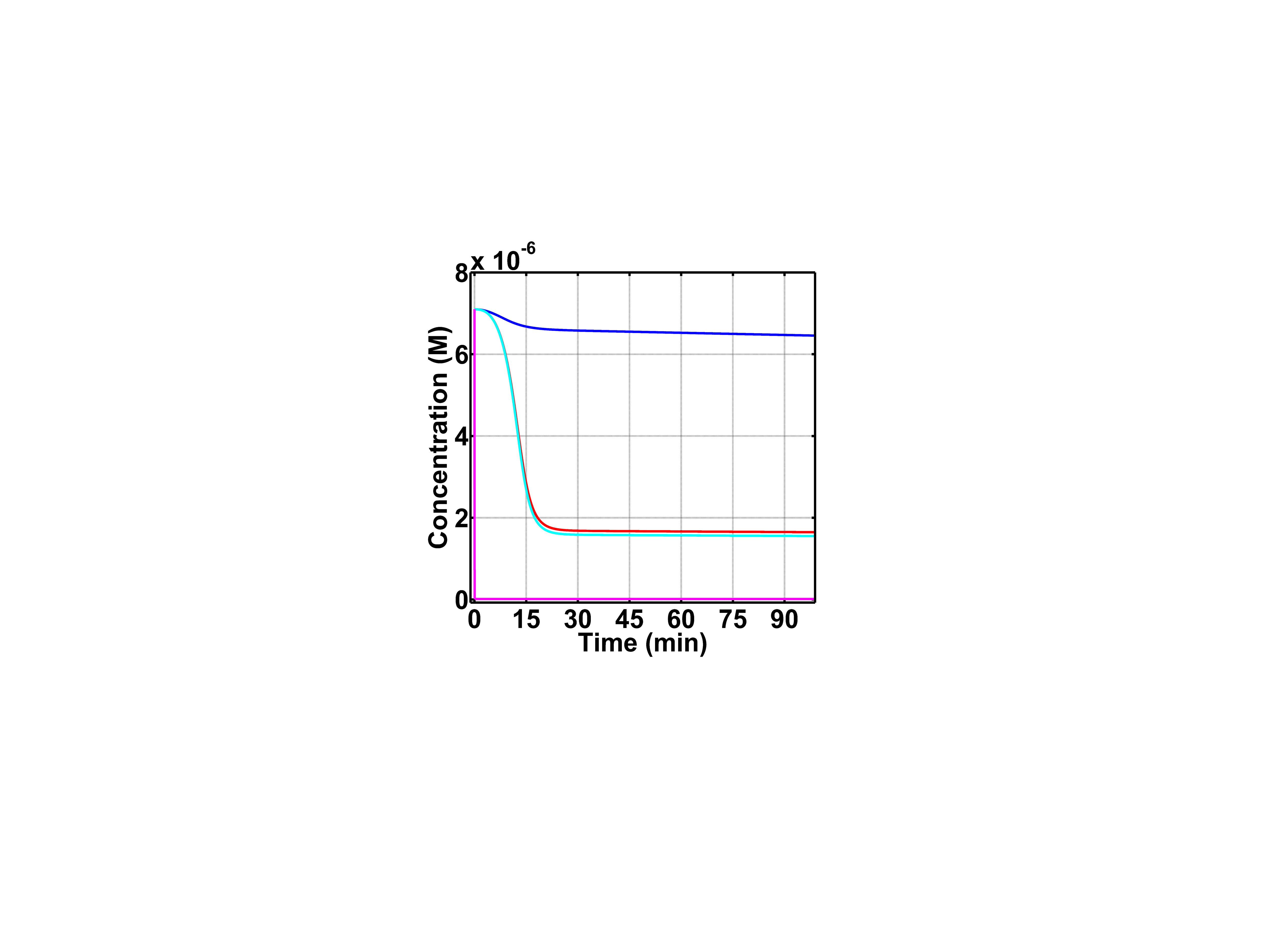

Supplement: S7 Fig — Compstatin and eculizumab are at concentrations 20-fold higher than the concentrations of their respective targets, C3 and C5. C3 is significantly consumed in FH disorder state and in FH disorder state with eculizumab treatment. Treatment with compstatin and dual treatment with compstatin and eculizumab consume C3 to form the compstatin:C3 complex. (TIF) [file pone.0198644.s007.tif]

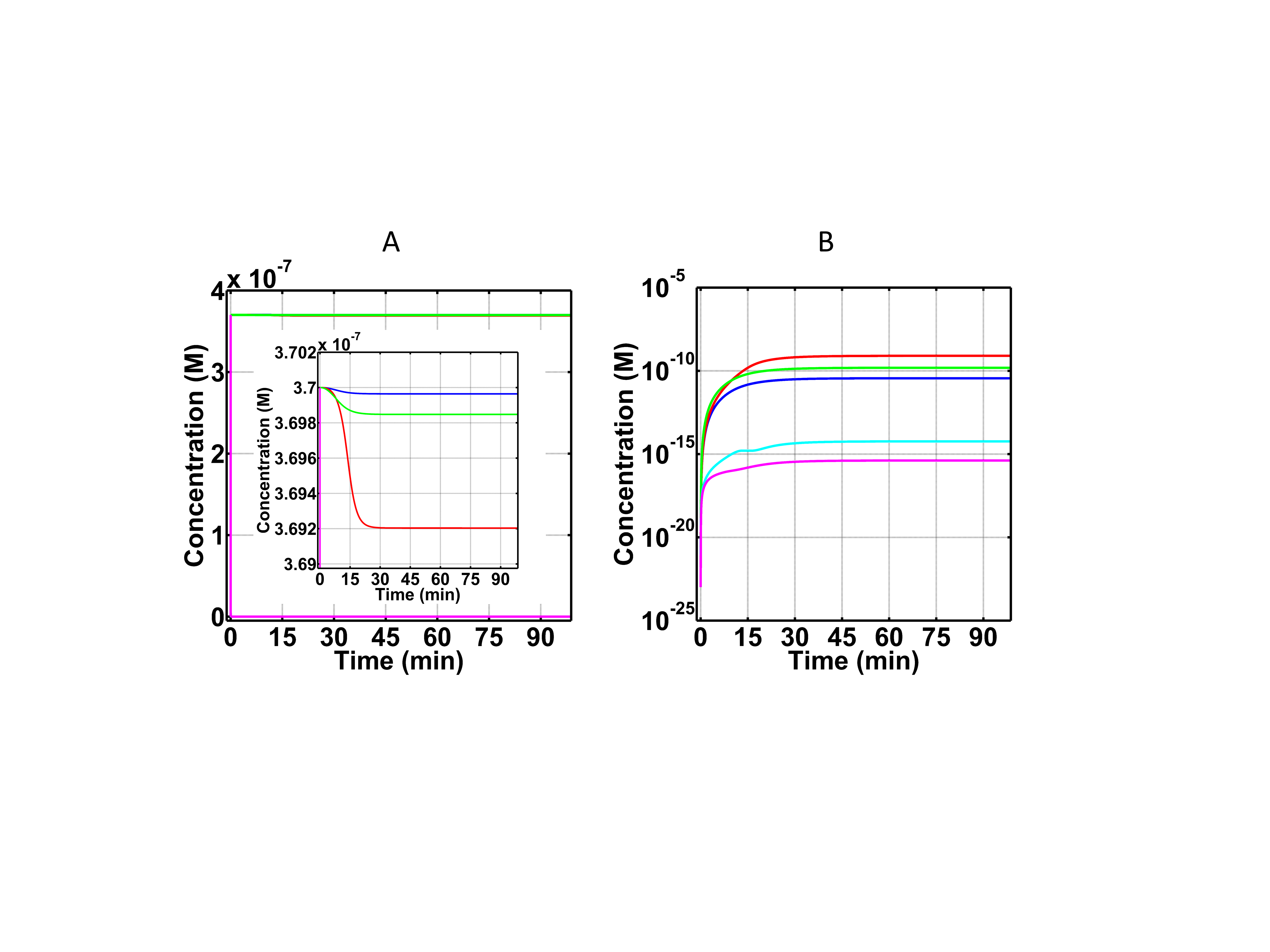

Supplement: S8 Fig — Compstatin and eculizumab are at concentrations 20-fold higher than the concentrations of their respective targets, C3 and C5. (A) Consumption of C5. The first three states (i–iii) consume small amounts of C5, as shown in the inset for the zoom-in time profiles. Treatment with eculizumab and dual treatment with compstatin and eculizumab generate similar (to each other) concentration-time profiles by removing C5 to form the eculizumab:C5 complex. (B) Production of C5a-desArg. The FH disorder state generates the highest concentration level of C5a-desArg, followed by the FH disorder state with compstatin treatment. Treatment with eculizumab over-restores the level of C5a-desArg, whereas dual treatment with compstatin and eculizumab produces the lowest level of C5a-desArg. (TIF) [file pone.0198644.s008.tif]

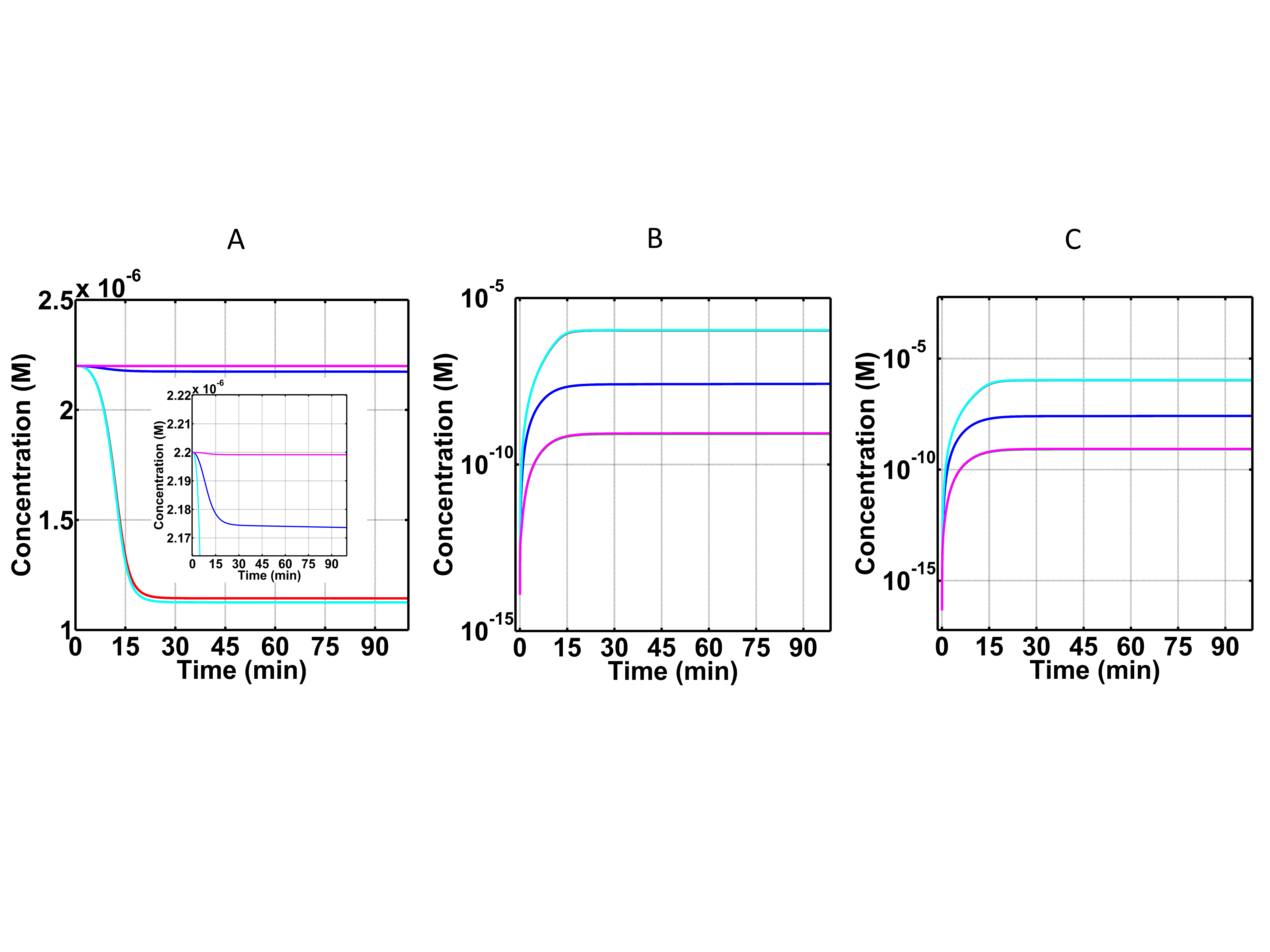

Supplement: S9 Fig — Compstatin and eculizumab are at concentrations 20-fold higher than the concentrations of their respective targets, C3 and C5. (A) Significant amount of FB is consumed in FH disorder state and FH disorder state with eculizumab treatment. Treatment with compstatin and dual treatment with compstatin and eculizumab over-restores the consumption of FB as shown in the inset. (B and C) Cleavage fragments Ba (Panel B) and Bb (Panel C) have the highest concentration in FH disorder state and FH disorder state with eculizumab treatment. The concentration-time profiles of Ba and Bb in FH disorder state are below those generated with eculizumab treatment. Compstatin and dual treatment with compstatin and eculizumab over-regulate the production of Ba and Bb (overlapping concentration-time profiles). (TIF) [file pone.0198644.s009.tif]

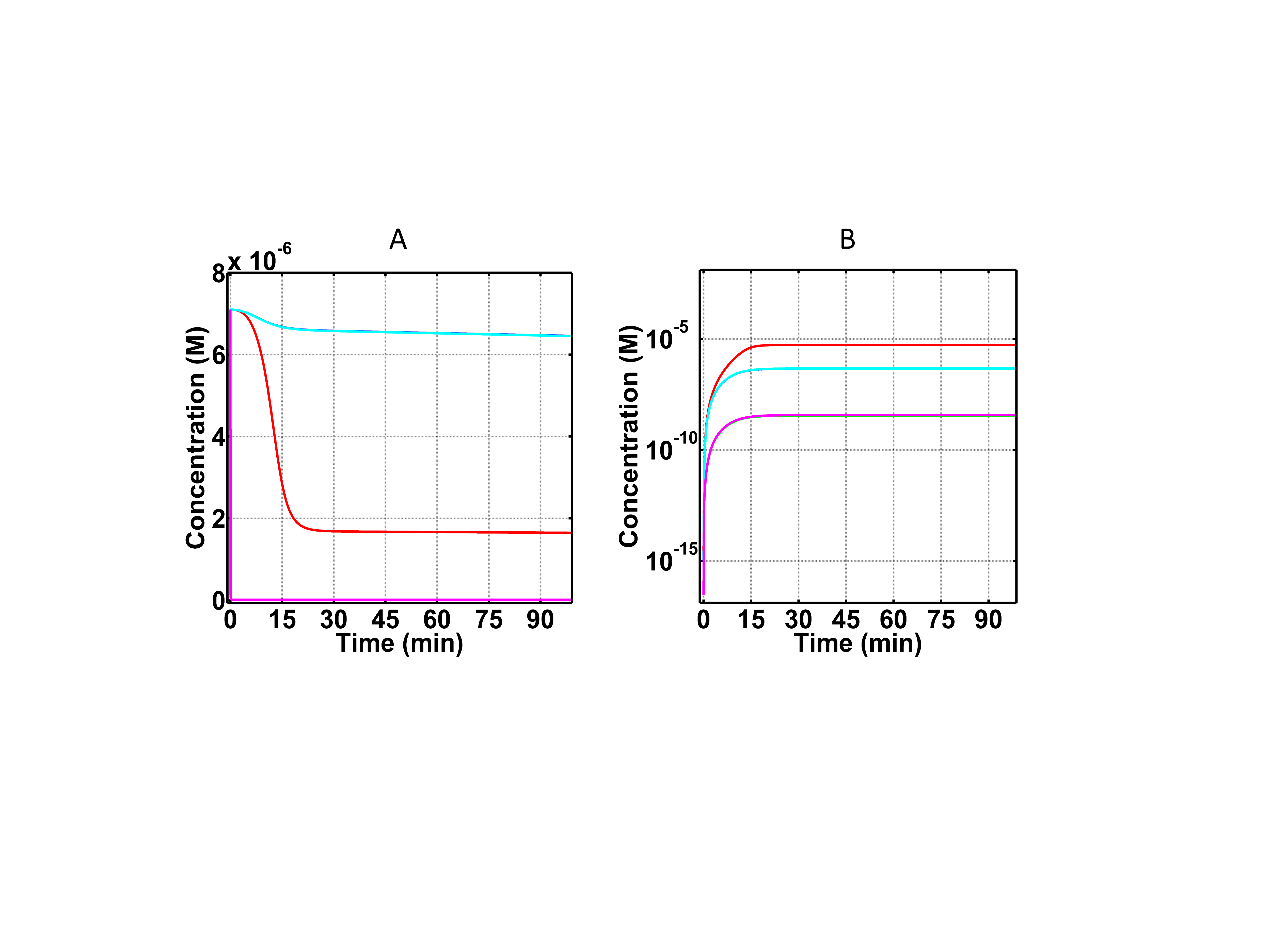

Supplement: S10 Fig — This figure is similar to S7 (C3) and main text Fig 9 (C3a-desArg), but shows the effects of inhibitors on the normal state instead of the FH disorder state. (TIF) [file pone.0198644.s010.tif]

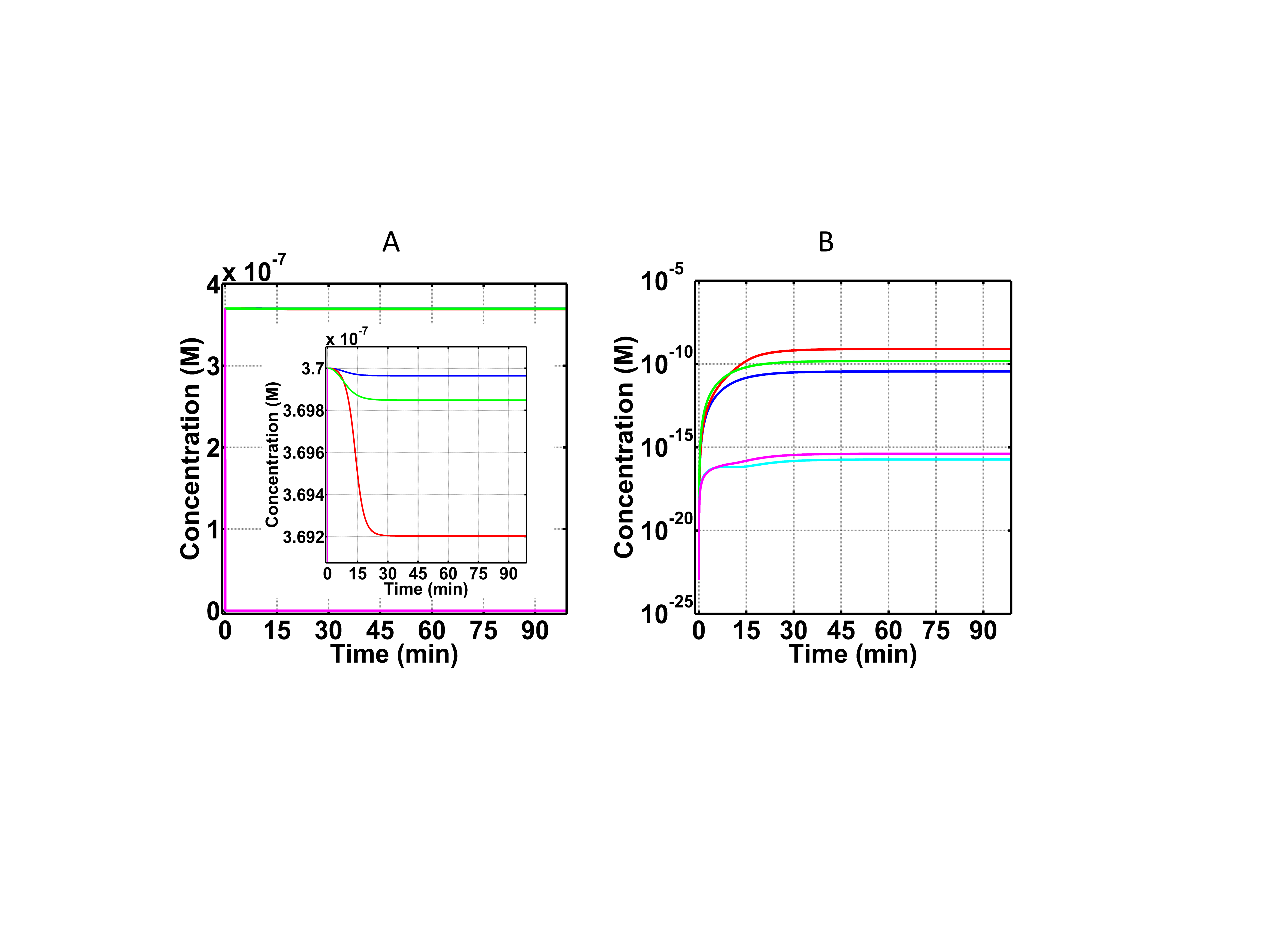

Supplement: S11 Fig — This figure is similar to S8, but shows the effects of inhibitors on the normal state instead of the FH disorder state. (TIF) [file pone.0198644.s011.tif]

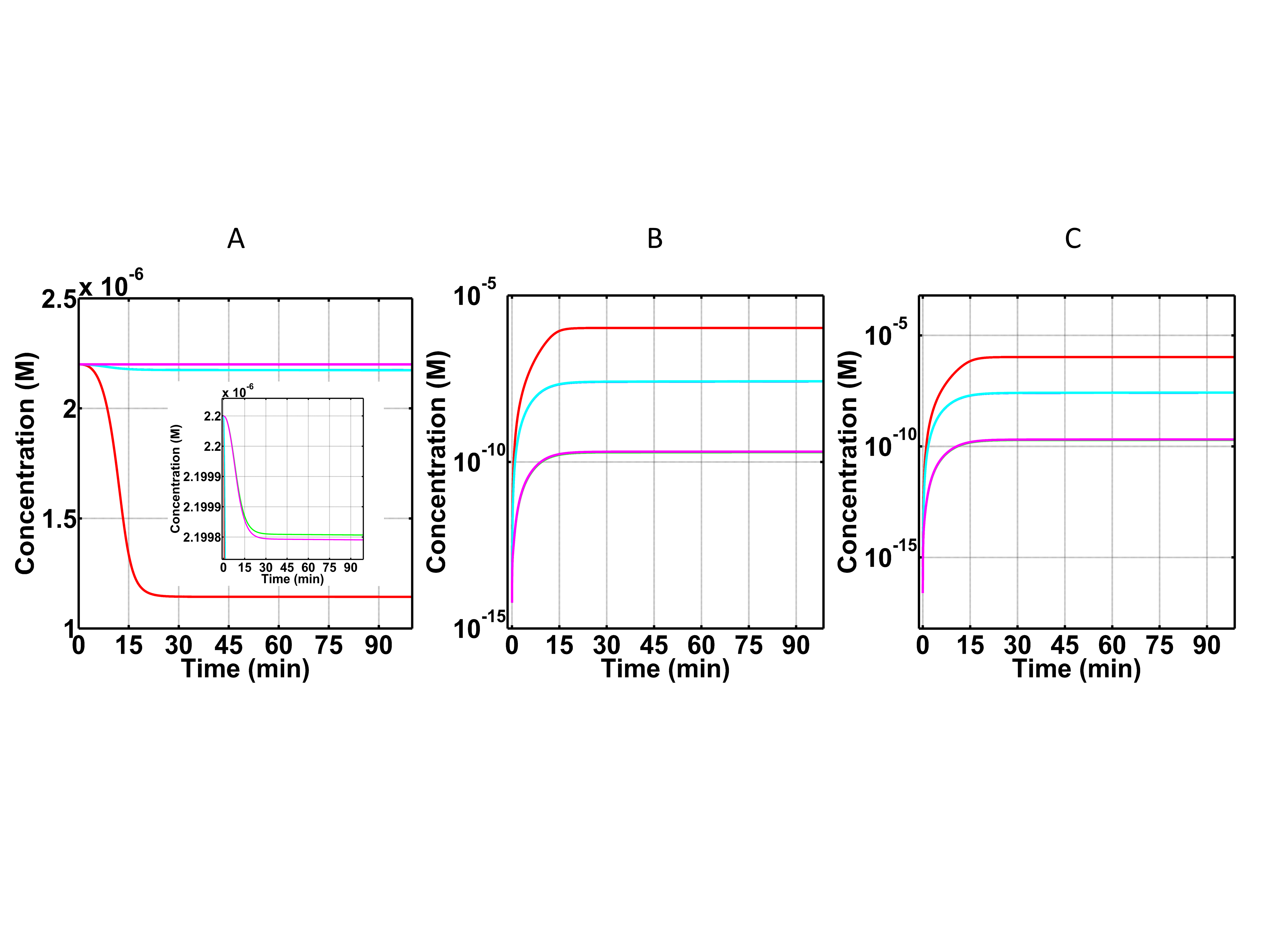

Supplement: S12 Fig — This figure is similar to S9, but shows the effects of inhibitors on the normal state instead of the FH disorder state. (TIF) [file pone.0198644.s012.tif]

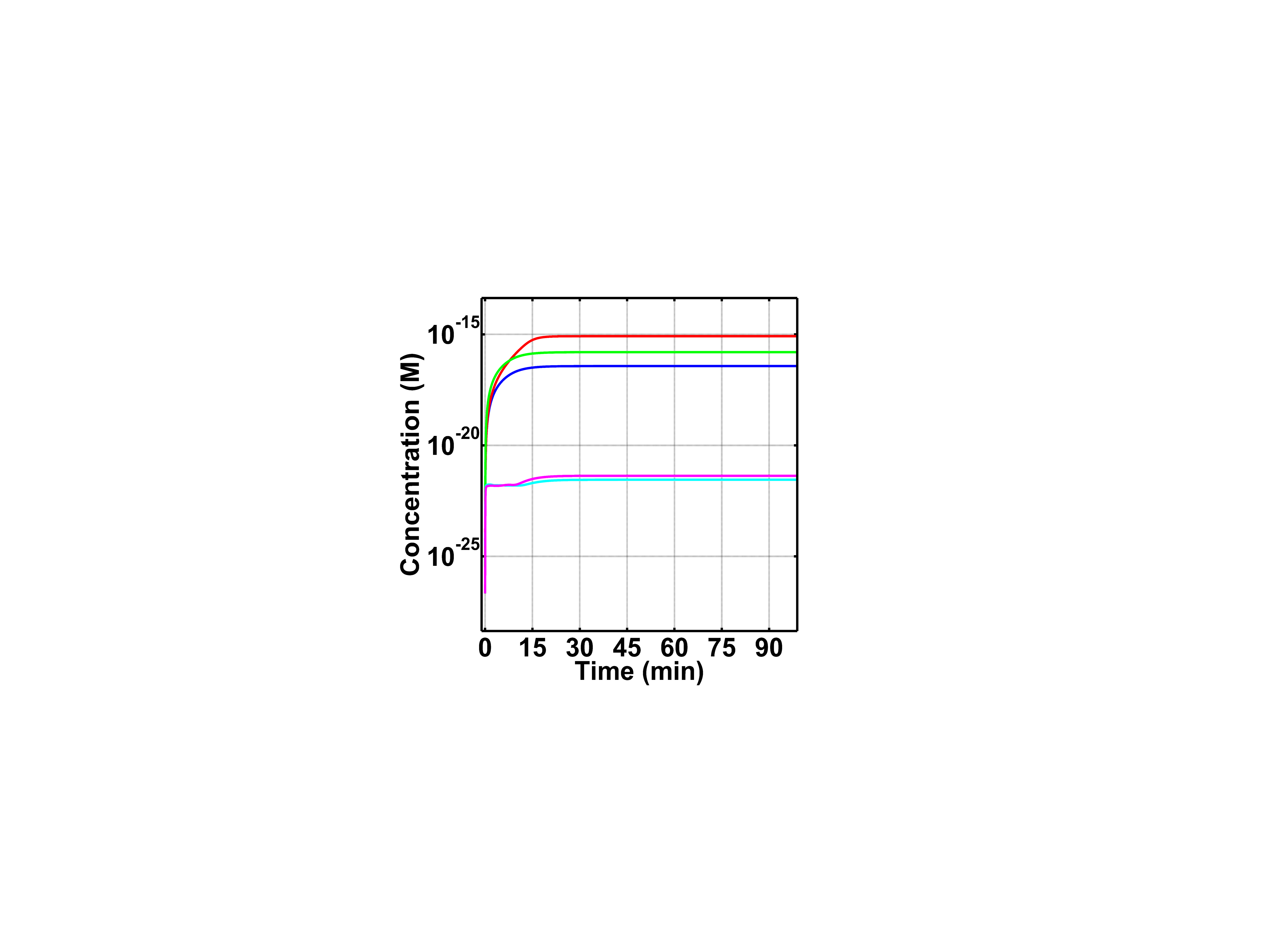

Supplement: S13 Fig — This figure is similar to main text Fig 9B, but shows the effects of inhibitors on the normal state instead of the FH disorder state. (TIF) [file pone.0198644.s013.tif]
