# Supplementary material for: A computational model for the evaluation of complement system regulation under homeostasis, disease, and drug intervention
Source: PLoS One. 2018 Jun 6;13(6):e0198644. doi: 10.1371/journal.pone.0198644 (PMC5991421; doi:10.1371/journal.pone.0198644)
Supplement: S1 Table — (A) Complement protein molecular masses and concentrations. (B) Host cell concentration. (C) Disease state FH concentration. (D) Inhibitor concentrations, chosen to be 20-fold higher than the concentration of the respective target proteins. (E) Inhibitor concentrations, Inhibitor concentrations, chosen to be at one-to-one ratio with the concentration of the respective target proteins. (F) Inhibitor concentrations, chosen to be 5-fold lower than the concentration of the respective target proteins. (PDF) [file pone.0198644.s014.pdf]

S1 Table

A. Complement protein molecular masses and concentrations.

Complement protein	Molecular mass (kDa)	Concentration (µM)	Source
C3	183	7.1	[1]
C4	205	3.0	[2]
C5	191	0.37	[3]
C1	632	0.136	[2]
C1q	459	0.034	[2]
$(C1rC1s)_2$	173	0.064	[2]
C2	102	0.20	[2]
C4BP	560	1.32	[2]
C1-INH	110	1.8	[2]
C6	120	0.53	[3]
C7	110	0.51	[3]
C8	151	0.36	[3]
C9	71	0.83	[3]
FB	93	2.2	[1]
FD	24	0.083	[1]
FI	88	0.40	[1]
Properdin	53	0.47	[1]
FH	155	3.2	[1]
FHL-1	43	0.87	[1,4]
Carboxypeptidase N	280	0.12	[5]
CR1	190	0.024	[1,6]
DAF	70	0.027	[1,6]
Vitronectin	83	6.1	[3]
Clusterin	80	0.88	[7]
IgG	150	81.0	[8]
CD59	18	0.21	[9]

B. Host cell concentration.

Host cell	Concentration (µM)	Source
Red blood cell	12.0	[10]

C. Disease state FH concentration.

Complement protein	Molecular mass (kDa)	Concentration (µM)
Impaired FH	155	0.32

D. Inhibitor concentrations, chosen to be 20-fold higher than the concentration of the respective target proteins.

**Inhibitor**	**Target protein**	**Concentration (M)**
Compstatin	C3	1.4×10^{-4}
Eculizumab	C5	7.4×10^{-6}

E. Inhibitor concentrations, chosen to be at one-to-one ratio with the concentration of the respective target proteins.

**Inhibitor**	**Target protein**	**Concentration (M)**
Compstatin	C3	7.1×10^{-6}
Eculizumab	C5	3.7×10^{-7}

F. Inhibitor concentrations, chosen to be 5-fold lower than the concentration of the respective target proteins.

**Inhibitor**	**Target protein**	**Concentration (M)**
Compstatin	C3	1.4×10^{-6}
Eculizumab	C5	7.4×10^{-8}

References

1. Zipfel PF. Complement: Alternative Pathway. eLS. John Wiley & Sons, Ltd; 2001. Available: http://onlinelibrary.wiley.com/doi/10.1038/npg.els.0000509/abstract

2. Arlaud GJ, Colomb MG. Complement: Classical Pathway. eLS. John Wiley & Sons, Ltd; 2001. doi:10.1038/npg.els.0000510

3. Muller-Eberhard HJ. The Membrane Attack Complex of Complement. Annu Rev Immunol. 1986;4: 503–528. doi:10.1146/annurev.iy.04.040186.002443

4. Schwaeble W, Zwirner J, Schulz TF, Linke RP, Dierich MP, Weiss EH. Human complement factor H: expression of an additional truncated gene product of 43 kDa in human liver. Eur J Immunol. 1987;17: 1485–1489. doi:10.1002/eji.1830171015

5. Matthews KW, Mueller-Ortiz SL, Wetsel RA. Carboxypeptidase N: a pleiotropic regulator of inflammation. Mol Immunol. 2004;40: 785–793. doi:10.1016/j.molimm.2003.10.002

6. Seya T. Human Regulator of Complement Activation (RCA) Gene Family Proteins and Their Relationship to Microbial Infection. Microbiol Immunol. 1995;39: 295–305. doi:10.1111/j.1348-0421.1995.tb02205.x

7. Murphy BF, Kirszbaum L, Walker ID, d'Apice AJ. SP-40,40, a newly identified normal human serum protein found in the SC5b-9 complex of complement and in the immune deposits in glomerulonephritis. J Clin Invest. 1988;81: 1858–1864.

8. Gonzalez-Quintela A, Alende R, Gude F, Campos J, Rey J, Meijide LM, et al. Serum levels of immunoglobulins (IgG, IgA, IgM) in a general adult population and their relationship with alcohol consumption, smoking and common metabolic abnormalities. Clin Exp Immunol. 2008;151: 42–50. doi:10.1111/j.1365-2249.2007.03545.x

9. Meri S, Morgan BP, Davies A, Daniels RH, Olavesen MG, Waldmann H, et al. Human protectin (CD59), an 18,000-20,000 MW complement lysis restricting factor, inhibits C5b-8 catalysed insertion of C9 into lipid bilayers. Immunology. 1990;71: 1–9.

10. Zewde N, Jr RDG, Dorado A, Morikis D. Quantitative Modeling of the Alternative Pathway of the Complement System. PLOS ONE. 2016;11: e0152337. doi:10.1371/journal.pone.0152337
