# Supplementary material for: A computational model for the evaluation of complement system regulation under homeostasis, disease, and drug intervention
Source: PLoS One. 2018 Jun 6;13(6):e0198644. doi: 10.1371/journal.pone.0198644 (PMC5991421; doi:10.1371/journal.pone.0198644)
Supplement: S2 Table — (PDF) [file pone.0198644.s015.pdf]

S2 Table. Kinetic rate constants.

Biochemical reaction	Rate constant	Value	Source
Hydrolysis of $C3(H_2O)$	$k^{+}_{\mathrm{C3(H_2O)}}$	4.5×10^{-6} s^{-1}	[1]
Association of Factor B to $C3(H_2O)$	$k^{+}_{\mathrm{C3(H_2O)B}}$	1.1×10^{4} $M^{-1}s^{-1}$	[2]
Dissociation of complex $C3(H_2O)B$	$k^{-}_{\mathrm{C3(H_2O)B}}$	1.4×10^{-3} s^{-1}	[2]
Association of Factor H to $C3(H_2O)$	$k^{+}_{\mathrm{C3(H_2O)H}}$	1.1×10^{6} $M^{-1}s^{-1}$	Assumption based on the interaction of functionally homologous protein C3b and FH
Dissociation of complex $C3(H_2O)H$	$k^{-}_{\mathrm{C3(H_2O)H}}$	5.9×10^{-2} s^{-1}	Assumption based on the interaction of functionally homologous protein C3b and FH
Association of Factor H Like to $C3(H_2O)$	$k^{+}_{\mathrm{C3(H_2O)HL}}$	1.1×10^{6} $M^{-1}s^{-1}$	Assumption based on the interaction of functionally homologous protein C3b and FH
Dissociation of complex $C3(H_2O)HL$	$k^{-}_{\mathrm{C3(H_2O)HL}}$	5.9×10^{-2} s^{-1}	Assumption based on the interaction of functionally homologous protein C3b and FH
Dissociation of complex $C3(H_2O)Bb$	$k^{-}_{\mathrm{C3(H_2O)Bb}}$	9.0×10^{-3} s^{-1}	[3]
Association of Factor B to C3b	k^{+}_{C3bB}	21.3×10^{4} $M^{-1}s^{-1}$	[4]
Dissociation of complex C3bB	k^{-}_{C3bB}	15.5×10^{-2} s^{-1}	[4]
Dissociation of complex C3bBb	k^{-}_{C3bBb}	7.7×10^{-3} s^{-1}	[3]
Dissociation of complex C3bBbP	k^{-}_{C3bBbP}	7.7×10^{-4} s^{-1}	[5]
Dissociation of complex C4bC2a	k^{-}_{C4bC2a}	4.0×10^{-3} s^{-1}	[6]

Association of properdin to C3b	k^{+}_{C3bP}	1.5×10^{5} $M^{-1}s^{-1}$	[7]
Dissociation of complex C3bP	k^{-}_{C3bP}	15.3×10^{-5} s^{-1}	[7]
Attachment of nfC3b to host cell	k^{+}_{hC3b}	4.2×10^{8} $M^{-1}s^{-1}$	[8]
Association of nfC3b to water	k^{+}_{fC3b}	4.2×10^{8} $M^{-1}s^{-1}$	[8]
Association of nfC3b to C3b	k^{+}_{C3bC3b}	4.2×10^{8} $M^{-1}s^{-1}$	[8] Calculated based on rates of diffusion in blood
Association of nfC3b to IgG	k^{+}_{IgGC3b}	4.2×10^{8} $M^{-1}s^{-1}$	[8] Calculated based on rates of diffusion in blood
Association of nfC3b to C4b	k^{+}_{C3bC4b}	4.2×10^{8} $M^{-1}s^{-1}$	[8] Calculated based on rates of diffusion in blood
Attachment of nfC4b to host cell	k^{+}_{hC3b}	4.2×10^{8} $M^{-1}s^{-1}$	[8] Calculated based on rates of diffusion in blood
Association of nfC4b to water	k^{+}_{fC4b}	4.2×10^{8} $M^{-1}s^{-1}$	[8] Calculated based on rates of diffusion in blood
Association of nfC4b to C4b	k^{+}_{C4bC4b}	4.2×10^{8} $M^{-1}s^{-1}$	[8] Calculated based on rates of diffusion in blood
Association of Factor H to C3b	k^{+}_{C3bH}	1.1×10^{6} $M^{-1}s^{-1}$	[9]
Dissociation of complex C3bH	k^{-}_{C3bH}	5.9×10^{-2} s^{-1}	[9]
Association of Factor H Like to C3b	k^{+}_{C3bHL}	1.1×10^{6} $M^{-1}s^{-1}$	[9]
Dissociation of complex C3bHL	k^{-}_{C3bHL}	5.9×10^{-2} s^{-1}	[9]
Association of CR1 to C3b	k^{+}_{C3bCR1}	4.4×10^{6} $M^{-1}s^{-1}$	[9]
Dissociation of complex	k^{-}_{C3bCR1}	5.7×10^{-2} s^{-1}	[9]

C3bCR1			
Association of DAF to C3 convertase on host cell	$k^{+}_{\mathrm{C3bBbDAF}}$	1.4×10^{3} $M^{-1}s^{-1}$	[10]
Dissociation of complex C3bBbDAF	$k^{-}_{\mathrm{C3bBbDAF}}$	1.2×10^{-3} s^{-1}	[10]
Decay of C3 convertase by inhibitor DAF on host cell	$k^{-}_{\mathrm{C3bBbDAF_{decay}}}$	1.0×10^{-2} s^{-1}	Assumption based on the decay rate of functionally homologous protein FH on C3bBb
Decay of C3 convertase by inhibitor CR1 on host cell	$k^{-}_{\mathrm{C3bBbCR1_{decay}}}$	1.0×10^{-2} s^{-1}	Assumption based on the decay rate of functionally homologous protein FH on C3bBb
Decay of C3 convertase by inhibitor CR1 on host cell	$k^{-}_{\mathrm{C4bC2aCR1_{decay}}}$	1.0×10^{-2} s^{-1}	Assumption based on the decay rate of functionally homologous protein FH on C3bBb
Decay of C3 convertase by inhibitor C4BP on host cell	$k^{-}_{\mathrm{C4bC2aC4BP_{decay}}}$	1.0×10^{-2} s^{-1}	Assumption based on the decay rate of functionally homologous protein FH on C3bBb
Decay of C3 convertase by inhibitor Factor H on host cell	$k^{-}_{\mathrm{C3bBbH_{decay}}}$	1.0×10^{-2} s^{-1}	[11]
Decay of C3 convertase by inhibitor Factor H-Like on host cell	$k^{-}_{\mathrm{C3bBbHL_{decay}}}$	1.0×10^{-2} s^{-1}	[11]
Decay of C3 convertase by inhibitor Factor H on host cell	$k^{-}_{\mathrm{C3(H_2O)BbH_{decay}}}$	1.0×10^{-2} s^{-1}	Assumption based on the decay rate of functionally homologous protein FH on C3bBb
Decay of C3 convertase by inhibitor Factor H Like on host cell	$k^{-}_{\mathrm{C3(H_2O)BbHL_{decay}}}$	1.0×10^{-2} s^{-1}	Assumption based on the decay rate of functionally homologous protein FH on C3bBb
Association of CR1 to iC3b	k^{+}_{iC3bCR1}	2.0×10^{3} $M^{-1}s^{-1}$	[8]
Dissociation of complex iC3bCR1	k^{-}_{iC3bCR1}	1.0×10^{-2} s^{-1}	[8]
Association of CR1 to	$k^{+}_{\mathrm{C3bC3bCR1}}$	9.8×10^{4} $M^{-1}s^{-1}$	[12]

C3bC3b			
Dissociation of complex C3bC3bCR1	$k^{-}_{\mathrm{C3bC3bCR1}}$	2.1×10^{-3} s^{-1}	[12]
Association of CR1 to C3biC3b	$k^{+}_{\mathrm{C3biC3bCR1}}$	9.8×10^{4} M^{-1}s^{-1}	[12]
Dissociation of complex C3biC3bCR1	$k^{-}_{\mathrm{C3biC3bCR1}}$	2.1×10^{-3} s^{-1}	[12]
Association of CR1 to iC3biC3b	$k^{+}_{\mathrm{iC3biC3bCR1}}$	9.8×10^{4} M^{-1}s^{-1}	Assumption based on the interaction of dimerized C3b and CR1
Dissociation of complex iC3biC3bCR1	$k^{-}_{\mathrm{iC3biC3bCR1}}$	2.1×10^{-3} s^{-1}	Assumption based on the interaction of dimerized C3b and CR1
Association of CR1 to iC3bC3dg	$k^{+}_{\mathrm{iC3bC3dgCR1}}$	9.8×10^{4} M^{-1}s^{-1}	Assumption based on the interaction of dimerized C3b and CR1
Dissociation of complex iC3bC3dgCR1	$k^{-}_{\mathrm{iC3bC3dgCR1}}$	2.1×10^{-3} s^{-1}	Assumption based on the interaction of dimerized C3b and CR1
Association of CR1 to C3bC4b	$k^{+}_{\mathrm{C3bC4bCR1}}$	9.8×10^{4} M^{-1}s^{-1}	[12]
Dissociation of complex C3bC4bCR1	$k^{-}_{\mathrm{C3bC4bCR1}}$	2.1×10^{-3} s^{-1}	[12]
Association of CR1 to C3bC4d	$k^{+}_{\mathrm{C3bC4dCR1}}$	9.8×10^{4} M^{-1}s^{-1}	[12]
Dissociation of complex C3bC4dCR1	$k^{-}_{\mathrm{C3bC4dCR1}}$	2.1×10^{-3} s^{-1}	[12]
Association of CR1 to iC3bC4b	$k^{+}_{\mathrm{iC3bC4bCR1}}$	9.8×10^{4} M^{-1}s^{-1}	Assumption based on the interaction of dimerized C3b and CR1
Dissociation of complex iC3bC4bCR1	$k^{-}_{\mathrm{iC3bC4bCR1}}$	2.1×10^{-3} s^{-1}	Assumption based on the interaction of dimerized C3b and CR1
Association of CR1 to iC3bC4d	$k^{+}_{\mathrm{iC3bC4dCR1}}$	9.8×10^{4} M^{-1}s^{-1}	Assumption based on the interaction dimerized C3b and CR1
Dissociation of complex	$k^{-}_{\mathrm{iC3bC4dCR1}}$	2.1×10^{-3} s^{-1}	Assumption based on the

iC3bC4dCR1			interaction of dimerized C3b and CR1
Association of CR1 to C3dgC4b	$k^{+}_{\mathrm{C3dgC4bCR1}}$	9.8×10^{4} $M^{-1}s^{-1}$	Assumption based on the interaction of dimerized C3b and CR1
Dissociation of complex C3dgC4bCR1	$k^{-}_{\mathrm{C3dgC4bCR1}}$	2.1×10^{-3} s^{-1}	Assumption based on the interaction of dimerized C3b and CR1
Association of CR1 to C4b	k^{+}_{C4bCR1}	3.8×10^{6} $M^{-1}s^{-1}$	[9]
Dissociation of complex C4bCR1	k^{-}_{C4bCR1}	4.2×10^{-2} s^{-1}	[9]
Association of C4BP to C4b	k^{+}_{C4bC4BP}	2.0×10^{5} $M^{-1}s^{-1}$	[13–15]
Dissociation of complex C4bC4BP	k^{-}_{C4bC4BP}	1.6×10^{-2} s^{-1}	[13–15]
Association of C2 to C4b	k^{+}_{C4bC2}	1.6×10^{6} $M^{-1}s^{-1}$	[2]
Dissociation of complex C4bC2	k^{-}_{C4bC2}	4.2×10^{-3} s^{-1}	[2]
Association of CR1 to C4bC4b	$k^{+}_{\mathrm{C4bC4bCR1}}$	9.8×10^{4} $M^{-1}s^{-1}$	Assumption based on the interaction of structurally and functionally homologous protein C3b and CR1
Dissociation of complex C4bC4bCR1	$k^{-}_{\mathrm{C4bC4bCR1}}$	2.1×10^{-3} s^{-1}	Assumption based on the interaction of structurally and functionally homologous protein C3b and CR1
Association of CR1 to C4bC4d	$k^{+}_{\mathrm{C4bC4dCR1}}$	9.8×10^{4} $M^{-1}s^{-1}$	Assumption based on the interaction of structurally and functionally homologous protein C3b and CR1
Dissociation of complex C4bC4dCR1	$k^{-}_{\mathrm{C4bC4dCR1}}$	2.1×10^{-3} s^{-1}	Assumption based on the interaction of structurally and functionally homologous protein C3b and CR1
Association of C1q to $(C1rC1s)_2$	k^{+}_{C1}	0.8×10^{6} $M^{-1}s^{-1}$	[16]
Dissociation of complex C1	k^{-}_{C1}	1.2×10^{-3} s^{-1}	[16]

Activation of C1	$k^{+}_{\text{activation}}$	2.8×10^{-3} s^{-1}	[17]
Association of C1-INH to C1*	$k^{+}_{\text{C1*C1-INH}}$	4.3×10^{5} $M^{-1}s^{-1}$	[18]
Dissociation of complex C3bC3bBb	k^{-}_{C3bC3bBb}	5.7×10^{-3} s^{-1}	[19]
Dissociation of complex C3bC3bBbP	$k^{-}_{\text{C3bC3bBbP}}$	5.7×10^{-4} s^{-1}	[5]
Dissociation of complex C3bC4bBb	k^{-}_{C3bC4bBb}	5.7×10^{-3} s^{-1}	Assumption based on the interaction of structurally and functionally homologous protein C3bC3bBb
Dissociation of complex C3bC4bBbP	$k^{-}_{\text{C3bC4bBbP}}$	5.7×10^{-4} s^{-1}	[5]
Dissociation of complex C3bC4bC2a	$k^{-}_{\text{C3bC4bC2a}}$	5.0×10^{-3} s^{-1}	[6]
Dissociation of complex C4bC4bC2a	$k^{-}_{\text{C4bC4bC2a}}$	6.0×10^{-3} s^{-1}	[6]
Dissociation of complex C5b	$k^{-}_{\text{C5b*}}$	5.0×10^{-3} s^{-1}	[19]
Association of C6 to C3bC3BbbC5b	k^{+}_{C5bC6}	6.0×10^{4} $M^{-1}s^{-1}$	[13,20]
Dissociation of complex C3bC3bBbC5bC6	k^{-}_{C5bC6}	9.0×10^{-8} s^{-1}	[13,20]
Association of C7 to C3bC3bBbC5bC6	k^{+}_{C5b7}	2.7×10^{6} $M^{-1}s^{-1}$	[21]
Dissociation of complex C5bC6C7	k^{-}_{C5b7}	5.5×10^{-7} s^{-1}	[21]
Attachment of C5b7 to host cell	$k^{+}_{\text{C5b7}_{\text{surface}}}$	4.2×10^{8} $M^{-1}s^{-1}$	[8]
Formation of C5b7 micelle in fluid	k^{+}_{micelle}	69.3 s^{-1}	[19]

Association of C8 to C5b7	k^{+}_{C5b8}	2.7×10^{6} $M^{-1}s^{-1}$	[21]
Dissociation of complex C5b8	k^{-}_{C5b8}	2.5×10^{-6} s^{-1}	[21]
Association of C9 to C5b8	k^{+}_{C5b9}	2.7×10^{6} $M^{-1}s^{-1}$	[21]
Dissociation of complex C5b9	k^{-}_{C5b9}	3.1×10^{-7} s^{-1}	[21]
Association of Cn to C5b7	k^{+}_{CnC5b7}	4.1×10^{5} $M^{-1}s^{-1}$	[13]
Dissociation of complex CnC5b7	k^{-}_{CnC5b7}	4.0×10^{-3} s^{-1}	[13]
Association of Cn to C5b8	k^{+}_{CnC5b8}	4.1×10^{5} $M^{-1}s^{-1}$	[13]
Dissociation of complex CnC5b8	k^{-}_{CnC5b8}	4.0×10^{-3} s^{-1}	[13]
Association of Cn to C5b9	k^{+}_{CnC5b9}	4.1×10^{5} $M^{-1}s^{-1}$	[13]
Dissociation of complex CnC5b9	k^{-}_{CnC5b9}	4.0×10^{-3} s^{-1}	[13]
Association of Vn to C5b7	k^{+}_{VnC5b7}	2.4×10^{5} $M^{-1}s^{-1}$	[13,22]
Dissociation of complex VnC5b7	k^{-}_{VnC5b7}	4.0×10^{-3} s^{-1}	[8]
Association of Vn to C5b8	k^{+}_{VnC5b8}	2.4×10^{5} $M^{-1}s^{-1}$	[13,22]
Dissociation of complex VnC5b8	k^{-}_{VnC5b8}	4.0×10^{-3} s^{-1}	[8]
Association of Vn to C5b9	k^{+}_{VnC5b9}	2.4×10^{5} $M^{-1}s^{-1}$	[13,22]

Dissociation of complex VnC5b9	k^{-}_{VnC5b9}	$4.0\times10^{-3}\ s^{-1}$	[8]
Association of CD59 to C5b8	$k^{+}_{\mathrm{C5b8CD59}}$	$1.0\times10^{6}\ M^{-1}s^{-1}$	Assumption based on the interaction of functionally homologous protein C5b9 and CD59
Dissociation of complex C5b8CD59	$k^{-}_{\mathrm{C5b8CD59}}$	$2.0\times10^{-4}\ s^{-1}$	Assumption based on the interaction of functionally homologous protein C5b9 and CD59
Association of CD59 to C5b9	$k^{+}_{\mathrm{C5b9CD59}}$	$1.0\times10^{6}\ M^{-1}s^{-1}$	[8]
Dissociation of complex C5b9CD59	$k^{-}_{\mathrm{C5b9CD59}}$	$2.0\times10^{-4}\ s^{-1}$	[8]
Association of Compstatin to C3	k^{+}_{CompC3}	$1.3\times10^{4}\ M^{-1}s^{-1}$	[23]
Dissociation of complex CompC3	k^{-}_{CompC3}	$2.9\times10^{-3}\ s^{-1}$	[23]
Association of Eculizumab to C5	k^{+}_{EcuC5}	$1.3\times10^{6}\ M^{-1}s^{-1}$	[24]
Dissociation of complex EcuC5	k^{-}_{EcuC5}	$2.3\times10^{-5}\ s^{-1}$	[24]
Cleavage of C3 by C3 convertase, $C3(H_2O)Bb$	k_{cat} $C3(H_2O)Bb$<br><br>K_m $C3(H_2O)Bb$	$1.8\ s^{-1}$<br><br>10.6×10^{-6} M	[3]
Cleavage of C3 by C3 convertase, C3bBb	k_{cat} C3bBb<br><br>K_m C3bBb	$1.8\ s^{-1}$<br><br>5.9×10^{-6} M	[3]
Cleavage of C5 by C3 convertase, C3bBb	k_{cat} C3bBb<br><br>K_m C3bBb	$1.1\times10^{-2}\ s^{-1}$<br><br>24.0×10^{-6} M	[25]
Cleavage of C3 by C3 convertase, C4bC2a	k_{cat} C4bC2a<br><br>K_m C4bC2a	$3.2\ s^{-1}$<br><br>1.8×10^{-6} M	[13,26]
Cleavage of C5 by the	k_{cat} C4bC2a	$2.2\times10^{-2}\ s^{-1}$	[6]

C3 convertase, C4bC2a	K_m C4bC2a	8.9×10^{-6} M	
Cleavage of C4 by activated C1, C1*	k_{cat} C1*<br>K_m C1*	$5.4\ s^{-1}$<br>6100×10^{-9} M	[27]
Cleavage of C2 by activated C1, C1*	k_{cat} C1*<br>K_m C1*	$5.1\ s^{-1}$<br>6.1×10^{-6} M	[27]
Activation of complex C3bB by enzyme Factor D	k_{cat} C3bB<br>K_m C3bB	$5.0\ s^{-1}$<br>2.5×10^{-6} M	Assumption based on the interaction of functionally homologous protein C3(H_2O) and FB
Activation of complex C3(H_2O)B by enzyme Factor D	k_{cat} C3(H_2O)B<br>K_m C3(H_2O)B	$5.0\ s^{-1}$<br>2.5×10^{-6} M	[28]
Cleavage of C3b by inhibitor Factor I	k_{cat} C3bH<br>K_m C3bH	$1.3\ s^{-1}$<br>2.5×10^{-7} M	[29]
Cleavage of C5 by the C5 convertase, C3bC3bBb	k_{cat} C3bC3bBb<br>K_m C3bC3bBb	$3.0\times10^{-3}\ s^{-1}$<br>1.7×10^{-6} M	[30]
Cleavage of C5 by the C5 convertase, C3bC4bBb	k_{cat} C3bC4bBb<br>K_m C3bC4bBb	$3.0\times10^{-3}\ s^{-1}$<br>1.7×10^{-6} M	Assumption based on the interaction of structurally and functionally homologous protein C3bC3bBb
Cleavage of C5 by the C5 convertase, C3bC4bC2a	k_{cat} C3bC4bC2a<br>K_m C3bC4bC2a	$1.8\times10^{-2}\ s^{-1}$<br>5.1×10^{-9} M	[6]
Cleavage of C5 by the C5 convertase, C4bC4bC2a	k_{cat} C4bC4bC2a<br>K_m C4bC4bC2a	$3.0\times10^{-2}\ s^{-1}$<br>5.6×10^{-6} M	[6]
Cleavage of C3a by Carboxypeptidase N, CPN	k_{cat} CPN<br>K_m CPN	$57.9\ s^{-1}$<br>77.1×10^{-6} M	[31]
Cleavage of C5a by Carboxypeptidase N, CPN	k_{cat} CPN<br>K_m CPN	$9.3\ s^{-1}$<br>602.2×10^{-6} M	[31]

Association of Impaired Factor H to $C3(H_2O)$ and C3b species	$k^{+}_{C3(H_2O)H}$<br>k^{+}_{C3bH}	1.1×10^{5} $M^{-1}s^{-1}$	Implemented for alternative pathway dysregulation

References

1. Pangburn MK, Schreiber RD, Müller-Eberhard HJ. Formation of the initial C3 convertase of the alternative complement pathway. Acquisition of C3b-like activities by spontaneous hydrolysis of the putative thioester in native C3. J Exp Med. 1981;154: 856–867.

2. Laich A, Sim RB. Complement C4bC2 complex formation: an investigation by surface plasmon resonance. Biochim Biophys Acta. 2001;1544: 96–112.

3. Pangburn MK, Müller-Eberhard HJ. The C3 convertase of the alternative pathway of human complement. Enzymic properties of the bimolecular proteinase. Biochem J. 1986;235: 723–730.

4. Chen H, Ricklin D, Hammel M, Garcia BL, McWhorter WJ, Sfyroera G, et al. Allosteric inhibition of complement function by a staphylococcal immune evasion protein. Proc Natl Acad Sci. 2010;107: 17621–17626. doi:10.1073/pnas.1003750107

5. Hourcade DE. The Role of Properdin in the Assembly of the Alternative Pathway C3 Convertases of Complement. J Biol Chem. 2006;281: 2128–2132. doi:10.1074/jbc.M508928200

6. Rawal N, Pangburn MK. Formation of High Affinity C5 Convertase of the Classical Pathway of Complement. J Biol Chem. 2003;278: 38476–38483. doi:10.1074/jbc.M307017200

7. Pedersen DV, Roumenina L, Jensen RK, Gadeberg TA, Marinozzi C, Picard C, et al. Functional and structural insight into properdin control of complement alternative pathway amplification. EMBO J. 2017;36: 1084–1099. doi:10.15252/embj.201696173

8. Zewde N, Jr RDG, Dorado A, Morikis D. Quantitative Modeling of the Alternative Pathway of the Complement System. PLOS ONE. 2016;11: e0152337. doi:10.1371/journal.pone.0152337

9. Bernet J, Mullick J, Panse Y, Parab PB, Sahu A. Kinetic Analysis of the Interactions between Vaccinia Virus Complement Control Protein and Human Complement Proteins C3b and C4b. J Virol. 2004;78: 9446–9457. doi:10.1128/JVI.78.17.9446-9457.2004

10. Harris CL, Abbott RJM, Smith RA, Morgan BP, Lea SM. Molecular Dissection of Interactions between Components of the Alternative Pathway of Complement and Decay Accelerating Factor (CD55). J Biol Chem. 2005;280: 2569–2578. doi:10.1074/jbc.M410179200

11. Wu J, Wu Y-Q, Ricklin D, Janssen BJC, Lambris JD, Gros P. Structure of C3b-factor H and implications for host protection by complement regulators. Nat Immunol. 2009;10: 728–733. doi:10.1038/ni.1755

12. Klickstein LB, Barbashov SF, Liu T, Jack RM, Nicholson-Weller A. Complement receptor type 1 (CR1, CD35) is a receptor for C1q. Immunity. 1997;7: 345–355.

13. Korotaevskiy AA, Hanin LG, Khanin MA. Non-linear dynamics of the complement system activation. Math Biosci. 2009;222: 127–143. doi:10.1016/j.mbs.2009.10.003

14. Fujita T, Tamura N. Interaction of C4-binding protein with cell-bound C4b. A quantitative analysis of binding and the role of C4-binding protein in proteolysis of cell-bound C4b. J Exp Med. 1983;157: 1239–1251.

15. Ziccardi RJ, Dahlback B, Müller-Eberhard HJ. Characterization of the interaction of human C4b-binding protein with physiological ligands. J Biol Chem. 1984;259: 13674–13679.

16. Bally I, Rossi V, Lunardi T, Thielens NM, Gaboriaud C, Arlaud GJ. Identification of the c1q-binding sites of human c1r and c1s a refined three-dimensional model of the c1 complex of complement. J Biol Chem. 2009;284: 19340–19348. doi:10.1074/jbc.M109.004473

17. Ziccardi RJ. Spontaneous activation of the first component of human complement (C1) by an intramolecular autocatalytic mechanism. J Immunol Baltim Md 1950. 1982;128: 2500–2504.

18. Kerr FK, Thomas AR, Wijeyewickrema LC, Whisstock JC, Boyd SE, Kaiserman D, et al. Elucidation of the substrate specificity of the MASP-2 protease of the lectin complement pathway and identification of the enzyme as a major physiological target of the serpin, C1-inhibitor. Mol Immunol. 2008;45: 670–677. doi:10.1016/j.molimm.2007.07.008

19. Muller-Eberhard HJ. The Membrane Attack Complex of Complement. Annu Rev Immunol. 1986;4: 503–528. doi:10.1146/annurev.iy.04.040186.002443

20. Li CKN, Levine RP. Rate process in the final stage of complement hemolysis. Immunochemistry. 1977;14: 421–428. doi:10.1016/0019-2791(77)90167-7

21. Podack ER, Biesecker G, Kolb WP, Müller-Eberhard HJ. The C5b-6 complex: reaction with C7, C8, C9. J Immunol Baltim Md 1950. 1978;121: 484–490.

22. McDonald JF, Nelsestuen GL. Potent inhibition of terminal complement assembly by clusterin: characterization of its impact on C9 polymerization. Biochemistry (Mosc). 1997;36: 7464–7473. doi:10.1021/bi962895r

23. López de Victoria A, Gorham RD, Bellows ML, Ling J, Lo DD, Floudas CA, et al. A New Generation of Potent Complement Inhibitors of the Compstatin Family. Chem Biol Drug Des. 2011;77: 431–440. doi:10.1111/j.1747-0285.2011.01111.x

24. Schatz-Jakobsen JA, Zhang Y, Johnson K, Neill A, Sheridan D, Andersen GR. Structural Basis for Eculizumab-Mediated Inhibition of the Complement Terminal Pathway. J Immunol. 2016;197: 337–344. doi:10.4049/jimmunol.1600280

25. Rawal N, Pangburn MK. C5 convertase of the alternative pathway of complement. Kinetic analysis of the free and surface-bound forms of the enzyme. J Biol Chem. 1998;273: 16828–16835.

26. Cooper NR. Enzymatic activity of the second component of complement. Biochemistry (Mosc). 1975;14: 4245–4251.

27. Rossi V, Teillet F, Thielens NM, Bally I, Arlaud GJ. Functional Characterization of Complement Proteases C1s/Mannan-binding Lectin-associated Serine Protease-2 (MASP-2) Chimeras Reveals the Higher C4 Recognition Efficacy of the MASP-2 Complement Control Protein Modules. J Biol Chem. 2005;280: 41811–41818. doi:10.1074/jbc.M503813200

28. Taylor FR, Bixler SA, Budman JI, Wen D, Karpusas M, Ryan ST, et al. Induced Fit Activation Mechanism of the Exceptionally Specific Serine Protease, Complement Factor D†. Biochemistry (Mosc). 1999;38: 2849–2859. doi:10.1021/bi982140f

29. Pangburn MK, Mueller-Eberhard HJ. Kinetic and thermodynamic analysis of the control of C3b by the complement regulatory proteins factors H and I. Biochemistry (Mosc). 1983;22: 178–185. doi:10.1021/bi00270a026

30. Rawal N, Pangburn M. Formation of high-affinity C5 convertases of the alternative pathway of complement. J Immunol Baltim Md 1950. 2001;166: 2635–2642.

31. Du X-Y, Zabel BA, Myles T, Allen SJ, Handel TM, Lee PP, et al. Regulation of chemerin bioactivity by plasma carboxypeptidase N, carboxypeptidase B (activated thrombin-activable fibrinolysis inhibitor), and platelets. J Biol Chem. 2009;284: 751–758. doi:10.1074/jbc.M805000200
