# Supplementary material for: A computational model for the evaluation of complement system regulation under homeostasis, disease, and drug intervention
Source: PLoS One. 2018 Jun 6;13(6):e0198644. doi: 10.1371/journal.pone.0198644 (PMC5991421; doi:10.1371/journal.pone.0198644)
Supplement: S3 Table — (PDF) [file pone.0198644.s016.pdf]

S3 Table. Range of kinetic rate constants implemented in sensitivity analysis.

Biochemical reaction	Rate constant	Value	Range of variation
Hydrolysis of $C3(H_2O)$	$k^{+}_{C3(H_2O)}$	4.5×10^{-6} s^{-1}	$4.5\times10^{-7} - 4.5\times10^{-5}$ s^{-1}
Association of Factor B to $C3(H_2O)$	$k^{+}_{C3(H_2O)B}$	1.1×10^{4} $M^{-1}s^{-1}$	$1.1\times10^{3} - 1.1\times10^{5}$ $M^{-1}s^{-1}$
Dissociation of complex $C3(H_2O)B$	$k^{-}_{C3(H_2O)B}$	1.4×10^{-3} s^{-1}	$1.4\times10^{-4} - 1.4\times10^{-2}$ s^{-1}
Association of Factor H to $C3(H_2O)$	$k^{+}_{C3(H_2O)H}$	1.1×10^{6} $M^{-1}s^{-1}$	$1.1\times10^{5} - 1.1\times10^{7}$ $M^{-1}s^{-1}$
Dissociation of complex $C3(H_2O)H$	$k^{-}_{C3(H_2O)H}$	5.9×10^{-2} s^{-1}	$5.9\times10^{-3} - 5.9\times10^{-1}$ s^{-1}
Association of Factor H Like to $C3(H_2O)$	$k^{+}_{C3(H_2O)HL}$	1.1×10^{6} $M^{-1}s^{-1}$	$1.1\times10^{5} - 1.1\times10^{7}$ $M^{-1}s^{-1}$
Dissociation of complex $C3(H_2O)HL$	$k^{-}_{C3(H_2O)HL}$	5.9×10^{-2} s^{-1}	$5.9\times10^{-3} - 5.9\times10^{-1}$ s^{-1}
Dissociation of complex $C3(H_2O)Bb$	$k^{-}_{C3(H_2O)Bb}$	9.0×10^{-3} s^{-1}	$9.0\times10^{-4} - 9.0\times10^{-2}$ s^{-1}
Association of Factor B to C3b	k^{+}_{C3bB}	21.3×10^{4} $M^{-1}s^{-1}$	$21.3\times10^{3} - 21.3\times10^{5}$ $M^{-1}s^{-1}$
Dissociation of complex C3bB	k^{-}_{C3bB}	15.5×10^{-2} s^{-1}	$15.5\times10^{-3} - 15.5\times10^{-1}$ s^{-1}
Dissociation of complex C3bBb	k^{-}_{C3bBb}	7.7×10^{-3} s^{-1}	$7.7\times10^{-4} - 7.7\times10^{-2}$ s^{-1}
Dissociation of complex C3bBbP	k^{-}_{C3bBbP}	7.7×10^{-4} s^{-1}	$7.7\times10^{-5} - 7.7\times10^{-3}$ s^{-1}
Dissociation of complex C4bC2a	k^{-}_{C4bC2a}	4.0×10^{-3} s^{-1}	$4.0\times10^{-4} - 4.0\times10^{-2}$ s^{-1}
Association of properdin to C3b	k^{+}_{C3bP}	1.5×10^{5} $M^{-1}s^{-1}$	$1.5\times10^{4} - 1.5\times10^{6}$ $M^{-1}s^{-1}$

Dissociation of complex C3bP	k^{-}_{C3bP}	15.3×10^{-5} s^{-1}	15.3×10^{-6} – 15.3×10^{-4} s^{-1}
Attachment of nfC3b to host cell	k^{+}_{hC3b}	4.2×10^{8} M^{-1}s^{-1}	4.2×10^{7} – 4.2×10^{9} M^{-1}s^{-1}
Association of nfC3b to water	k^{+}_{fC3b}	4.2×10^{8} M^{-1}s^{-1}	4.2×10^{7} – 4.2×10^{9} M^{-1}s^{-1}
Association of nfC3b to C3b	k^{+}_{C3bC3b}	4.2×10^{8} M^{-1}s^{-1}	4.2×10^{7} – 4.2×10^{9} M^{-1}s^{-1}
Association of nfC3b to IgG	k^{+}_{IgGC3b}	4.2×10^{8} M^{-1}s^{-1}	4.2×10^{7} – 4.2×10^{9} M^{-1}s^{-1}
Association of nfC3b to C4b	k^{+}_{C3bC4b}	4.2×10^{8} M^{-1}s^{-1}	4.2×10^{7} – 4.2×10^{9} M^{-1}s^{-1}
Attachment of nfC4b to host cell	k^{+}_{hC3b}	4.2×10^{8} M^{-1}s^{-1}	4.2×10^{7} – 4.2×10^{9} M^{-1}s^{-1}
Association of nfC4b to water	k^{+}_{fC4b}	4.2×10^{8} M^{-1}s^{-1}	4.2×10^{7} – 4.2×10^{9} M^{-1}s^{-1}
Association of nfC4b to C4b	k^{+}_{C4bC4b}	4.2×10^{8} M^{-1}s^{-1}	4.2×10^{7} – 4.2×10^{9} M^{-1}s^{-1}
Association of Factor H to C3b	k^{+}_{C3bH}	1.1×10^{6} M^{-1}s^{-1}	1.1×10^{5} – 1.1×10^{7} M^{-1}s^{-1}
Dissociation of complex C3bH	k^{-}_{C3bH}	5.9×10^{-2} s^{-1}	5.9×10^{-3} – 5.9×10^{-1} s^{-1}
Association of Factor H Like to C3b	k^{+}_{C3bHL}	1.1×10^{6} M^{-1}s^{-1}	1.1×10^{5} – 1.1×10^{7} M^{-1}s^{-1}
Dissociation of complex C3bHL	k^{-}_{C3bHL}	5.9×10^{-2} s^{-1}	5.9×10^{-3} – 5.9×10^{-1} s^{-1}
Association of CR1 to C3b	k^{+}_{C3bCR1}	4.4×10^{6} M^{-1}s^{-1}	4.4×10^{5} – 4.4×10^{7} M^{-1}s^{-1}
Dissociation of complex C3bCR1	k^{-}_{C3bCR1}	5.7×10^{-2} s^{-1}	5.7×10^{-3} – 5.7×10^{-1} s^{-1}

Association of DAF to C3 convertase on host cell	$k^{+}_{\mathrm{C3bBbDAF}}$	1.4×10^{3} $M^{-1}s^{-1}$	$1.4\times10^{2} - 1.4\times10^{4}$ $M^{-1}s^{-1}$
Dissociation of complex C3bBbDAF	$k^{-}_{\mathrm{C3bBbDAF}}$	1.2×10^{-3} s^{-1}	$1.2\times10^{-4} - 1.2\times10^{-2}$ s^{-1}
Decay of C3 convertase by inhibitor DAF on host cell	$k^{-}_{\mathrm{C3bBbDAF}_{\mathrm{decay}}}$	1.0×10^{-2} s^{-1}	$1.0\times10^{-3} - 1.0\times10^{-1}$ s^{-1}
Decay of C3 convertase by inhibitor CR1 on host cell	$k^{-}_{\mathrm{C3bBbCR1}_{\mathrm{decay}}}$	1.0×10^{-2} s^{-1}	$1.0\times10^{-3} - 1.0\times10^{-1}$ s^{-1}
Decay of C3 convertase by inhibitor CR1 on host cell	$k^{-}_{\mathrm{C4bC2aCR1}_{\mathrm{decay}}}$	1.0×10^{-2} s^{-1}	$1.0\times10^{-3} - 1.0\times10^{-1}$ s^{-1}
Decay of C3 convertase by inhibitor C4BP on host cell	$k^{-}_{\mathrm{C4bC2aC4BP}_{\mathrm{decay}}}$	1.0×10^{-2} s^{-1}	$1.0\times10^{-3} - 1.0\times10^{-1}$ s^{-1}
Decay of C3 convertase by inhibitor Factor H on host cell	$k^{-}_{\mathrm{C3bBbH}_{\mathrm{decay}}}$	1.0×10^{-2} s^{-1}	$1.0\times10^{-3} - 1.0\times10^{-1}$ s^{-1}
Decay of C3 convertase by inhibitor Factor H-Like on host cell	$k^{-}_{\mathrm{C3bBbHL}_{\mathrm{decay}}}$	1.0×10^{-2} s^{-1}	$1.0\times10^{-3} - 1.0\times10^{-1}$ s^{-1}
Decay of C3 convertase by inhibitor Factor H on host cell	$k^{-}_{\mathrm{C3(H_2O)BbH}_{\mathrm{decay}}}$	1.0×10^{-2} s^{-1}	$1.0\times10^{-3} - 1.0\times10^{-1}$ s^{-1}
Decay of C3 convertase by inhibitor Factor H Like on host cell	$k^{-}_{\mathrm{C3(H_2O)BbHL}_{\mathrm{decay}}}$	1.0×10^{-2} s^{-1}	$1.0\times10^{-3} - 1.0\times10^{-1}$ s^{-1}
Association of CR1 to iC3b	k^{+}_{iC3bCR1}	4.4×10^{6} $M^{-1}s^{-1}$	$4.4\times10^{5} - 4.4\times10^{7}$ $M^{-1}s^{-1}$
Dissociation of complex iC3bCR1	k^{-}_{iC3bCR1}	5.7×10^{-2} s^{-1}	$5.7\times10^{-3} - 5.7\times10^{-1}$ s^{-1}
Association of CR1 to C3bC3b	$k^{+}_{\mathrm{C3bC3bCR1}}$	9.8×10^{4} $M^{-1}s^{-1}$	$9.8\times10^{3} - 9.8\times10^{5}$ $M^{-1}s^{-1}$
Dissociation of complex C3bC3bCR1	$k^{-}_{\mathrm{C3bC3bCR1}}$	2.1×10^{-3} s^{-1}	$2.1\times10^{-4} - 2.1\times10^{-2}$ s^{-1}

Association of CR1 to C3biC3b	$k^{+}_{\text{C3biC3bCR1}}$	9.8×10^{4} $M^{-1}s^{-1}$	$9.8\times10^{3} - 9.8\times10^{5}$ $M^{-1}s^{-1}$
Dissociation of complex C3biC3bCR1	$k^{-}_{\text{C3biC3bCR1}}$	2.1×10^{-3} s^{-1}	$2.1\times10^{-4} - 2.1\times10^{-2}$ s^{-1}
Association of CR1 to iC3biC3b	$k^{+}_{\text{iC3biC3bCR1}}$	9.8×10^{4} $M^{-1}s^{-1}$	$9.8\times10^{3} - 9.8\times10^{5}$ $M^{-1}s^{-1}$
Dissociation of complex iC3biC3bCR1	$k^{-}_{\text{iC3biC3bCR1}}$	2.1×10^{-3} s^{-1}	$2.1\times10^{-4} - 2.1\times10^{-2}$ s^{-1}
Association of CR1 to iC3bC3dg	$k^{+}_{\text{iC3bC3dgCR1}}$	9.8×10^{4} $M^{-1}s^{-1}$	$9.8\times10^{3} - 9.8\times10^{5}$ $M^{-1}s^{-1}$
Dissociation of complex iC3bC3dgCR1	$k^{-}_{\text{iC3bC3dgCR1}}$	2.1×10^{-3} s^{-1}	$2.1\times10^{-4} - 2.1\times10^{-2}$ s^{-1}
Association of CR1 to C3bC4b	$k^{+}_{\text{C3bC4bCR1}}$	9.8×10^{4} $M^{-1}s^{-1}$	$9.8\times10^{3} - 9.8\times10^{5}$ $M^{-1}s^{-1}$
Dissociation of complex C3bC4bCR1	$k^{-}_{\text{C3bC4bCR1}}$	2.1×10^{-3} s^{-1}	$2.1\times10^{-4} - 2.1\times10^{-2}$ s^{-1}
Association of CR1 to C3bC4d	$k^{+}_{\text{C3bC4dCR1}}$	9.8×10^{4} $M^{-1}s^{-1}$	$9.8\times10^{3} - 9.8\times10^{5}$ $M^{-1}s^{-1}$
Dissociation of complex C3bC4dCR1	$k^{-}_{\text{C3bC4dCR1}}$	2.1×10^{-3} s^{-1}	$2.1\times10^{-4} - 2.1\times10^{-2}$ s^{-1}
Association of CR1 to iC3bC4b	$k^{+}_{\text{iC3bC4bCR1}}$	9.8×10^{4} $M^{-1}s^{-1}$	$9.8\times10^{3} - 9.8\times10^{5}$ $M^{-1}s^{-1}$
Dissociation of complex iC3bC4bCR1	$k^{-}_{\text{iC3bC4bCR1}}$	2.1×10^{-3} s^{-1}	$2.1\times10^{-4} - 2.1\times10^{-2}$ s^{-1}
Association of CR1 to iC3bC4d	$k^{+}_{\text{iC3bC4dCR1}}$	9.8×10^{4} $M^{-1}s^{-1}$	$9.8\times10^{3} - 9.8\times10^{5}$ $M^{-1}s^{-1}$
Dissociation of complex iC3bC4dCR1	$k^{-}_{\text{iC3bC4dCR1}}$	2.1×10^{-3} s^{-1}	$2.1\times10^{-4} - 2.1\times10^{-2}$ s^{-1}

Association of CR1 to C3dgC4b	$k^{+}_{\mathrm{C3dgC4bCR1}}$	9.8×10^{4} $M^{-1}s^{-1}$	$9.8\times10^{3} - 9.8\times10^{5}$ $M^{-1}s^{-1}$
Dissociation of complex C3dgC4bCR1	$k^{-}_{\mathrm{C3dgC4bCR1}}$	2.1×10^{-3} s^{-1}	$2.1\times10^{-4} - 2.1\times10^{-2}$ s^{-1}
Association of CR1 to C4b	k^{+}_{C4bCR1}	3.8×10^{6} $M^{-1}s^{-1}$	$3.8\times10^{5} - 3.8\times10^{7}$ $M^{-1}s^{-1}$
Dissociation of complex C4bCR1	k^{-}_{C4bCR1}	4.2×10^{-2} s^{-1}	$4.2\times10^{-3} - 4.2\times10^{-1}$ s^{-1}
Association of C4BP to C4b	k^{+}_{C4bC4BP}	2.0×10^{5} $M^{-1}s^{-1}$	$2.0\times10^{4} - 2.0\times10^{6}$ $M^{-1}s^{-1}$
Dissociation of complex C4bC4BP	k^{-}_{C4bC4BP}	1.6×10^{-2} s^{-1}	$1.6\times10^{-3} - 1.6\times10^{-1}$ s^{-1}
Association of C2 to C4b	k^{+}_{C4bC2}	1.6×10^{6} $M^{-1}s^{-1}$	$1.6\times10^{5} - 1.6\times10^{7}$ $M^{-1}s^{-1}$
Dissociation of complex C4bC2	k^{-}_{C4bC2}	4.2×10^{-3} s^{-1}	$4.2\times10^{-4} - 4.2\times10^{-2}$ s^{-1}
Association of CR1 to C4bC4b	$k^{+}_{\mathrm{C4bC4bCR1}}$	3.8×10^{6} $M^{-1}s^{-1}$	$3.8\times10^{5} - 3.8\times10^{7}$ $M^{-1}s^{-1}$
Dissociation of complex C4bC4bCR1	$k^{-}_{\mathrm{C4bC4bCR1}}$	4.2×10^{-2} s^{-1}	$4.2\times10^{-3} - 4.2\times10^{-1}$ s^{-1}
Association of CR1 to C4bC4d	$k^{+}_{\mathrm{C4bC4dCR1}}$	3.8×10^{6} $M^{-1}s^{-1}$	$3.8\times10^{5} - 3.8\times10^{7}$ $M^{-1}s^{-1}$
Dissociation of complex C4bC4dCR1	$k^{-}_{\mathrm{C4bC4dCR1}}$	4.2×10^{-2} s^{-1}	$4.2\times10^{-3} - 4.2\times10^{-1}$ s^{-1}
Association of C1q to $(C1rC1s)_2$	k^{+}_{C1}	0.8×10^{6} $M^{-1}s^{-1}$	$0.8\times10^{5} - 0.8\times10^{7}$ $M^{-1}s^{-1}$
Dissociation of complex C1	k^{-}_{C1}	1.2×10^{-3} s^{-1}	$1.2\times10^{-4} - 1.2\times10^{-2}$ s^{-1}
Activation of C1	$k^{+}_{\mathrm{activation}}$	2.8×10^{-3} s^{-1}	$2.8\times10^{-4} - 2.8\times10^{-2}$ s^{-1}
Association of C1-INH to C1*	$k^{+}_{\mathrm{C1*C1-INH}}$	4.3×10^{5} $M^{-1}s^{-1}$	$4.3\times10^{4} - 4.3\times10^{6}$ $M^{-1}s^{-1}$

Dissociation of complex C3bC3bBb	$k^{-}_{\mathrm{C3bC3bBb}}$	5.7×10^{-3} s^{-1}	$5.7\times10^{-4} - 5.7\times10^{-2}$ s^{-1}
Dissociation of complex C3bC3bBbP	$k^{-}_{\mathrm{C3bC3bBbP}}$	5.7×10^{-4} s^{-1}	$5.7\times10^{-5} - 5.7\times10^{-3}$ s^{-1}
Dissociation of complex C3bC4bBb	$k^{-}_{\mathrm{C3bC4bBb}}$	5.7×10^{-3} s^{-1}	$5.7\times10^{-4} - 5.7\times10^{-2}$ s^{-1}
Dissociation of complex C3bC4bBbP	$k^{-}_{\mathrm{C3bC4bBbP}}$	5.7×10^{-4} s^{-1}	$5.7\times10^{-5} - 5.7\times10^{-3}$ s^{-1}
Dissociation of complex C3bC4bC2a	$k^{-}_{\mathrm{C3bC4bC2a}}$	5.0×10^{-3} s^{-1}	$5.7\times10^{-4} - 5.7\times10^{-2}$ s^{-1}
Dissociation of complex C4bC4bC2a	$k^{-}_{\mathrm{C4bC4bC2a}}$	6.0×10^{-3} s^{-1}	$6.0\times10^{-4} - 6.0\times10^{-2}$ s^{-1}
Dissociation of complex C5b	$k^{-}_{\mathrm{C5b*}}$	5.0×10^{-3} s^{-1}	$5.0\times10^{-4} - 5.0\times10^{-2}$ s^{-1}
Association of C6 to C3bC3BbbC5b	k^{+}_{C5bC6}	6.0×10^{4} M^{-1}s^{-1}	$6.0\times10^{3} - 6.0\times10^{5}$ M^{-1}s^{-1}
Dissociation of complex C3bC3bBbC5bC6	k^{-}_{C5bC6}	9.0×10^{-8} s^{-1}	$9.0\times10^{-9} - 9.0\times10^{-7}$ s^{-1}
Association of C7 to C3bC3bBbC5bC6	k^{+}_{C5b7}	2.7×10^{6} M^{-1}s^{-1}	$2.7\times10^{5} - 2.7\times10^{7}$ M^{-1}s^{-1}
Dissociation of complex C5bC6C7	k^{-}_{C5b7}	5.5×10^{-7} s^{-1}	$5.5\times10^{-8} - 5.5\times10^{-6}$ s^{-1}
Attachment of C5b7 to host cell	$k^{+}_{\mathrm{C5b7_{surface}}}$	4.2×10^{8} M^{-1}s^{-1}	$4.2\times10^{7} - 4.2\times10^{9}$ M^{-1}s^{-1}
Formation of C5b7 micelle in fluid	k^{+}_{micelle}	69.3 s^{-1}	6.93 – 693 s^{-1}
Association of C8 to C5b7	k^{+}_{C5b8}	2.7×10^{6} M^{-1}s^{-1}	$2.7\times10^{5} - 2.7\times10^{7}$ M^{-1}s^{-1}
Dissociation of complex C5b8	k^{-}_{C5b8}	2.5×10^{-6} s^{-1}	$2.5\times10^{-7} - 2.5\times10^{-5}$ M^{-1}s^{-1}

Association of C9 to C5b8	k^{+}_{C5b9}	2.7×10^{6} M^{-1}s^{-1}	2.7×10^{5} – 2.7×10^{7} M^{-1}s^{-1}
Dissociation of complex C5b9	k^{-}_{C5b9}	3.1×10^{-7} s^{-1}	3.1×10^{-8} – 3.1×10^{-6} M^{-1}s^{-1}
Association of Cn to C5b7	k^{+}_{CnC5b7}	4.1×10^{5} M^{-1}s^{-1}	4.1×10^{4} – 4.1×10^{6} M^{-1}s^{-1}
Dissociation of complex CnC5b7	k^{-}_{CnC5b7}	4.0×10^{-3} s^{-1}	4.0×10^{-4} – 4.0×10^{-2} s^{-1}
Association of Cn to C5b8	k^{+}_{CnC5b8}	4.1×10^{5} M^{-1}s^{-1}	4.1×10^{4} – 4.1×10^{6} M^{-1}s^{-1}
Dissociation of complex CnC5b8	k^{-}_{CnC5b8}	4.0×10^{-3} s^{-1}	4.0×10^{-4} – 4.0×10^{-2} s^{-1}
Association of Cn to C5b9	k^{+}_{CnC5b9}	4.1×10^{5} M^{-1}s^{-1}	4.1×10^{4} – 4.1×10^{6} M^{-1}s^{-1}
Dissociation of complex CnC5b9	k^{-}_{CnC5b9}	4.0×10^{-3} s^{-1}	4.0×10^{-4} – 4.0×10^{-2} s^{-1}
Association of Vn to C5b7	k^{+}_{VnC5b7}	2.4×10^{5} M^{-1}s^{-1}	2.4×10^{4} – 2.4×10^{6} M^{-1}s^{-1}
Dissociation of complex VnC5b7	k^{-}_{VnC5b7}	4.0×10^{-3} s^{-1}	4.0×10^{-4} – 4.0×10^{-2} s^{-1}
Association of Vn to C5b8	k^{+}_{VnC5b8}	2.4×10^{5} M^{-1}s^{-1}	2.4×10^{4} – 2.4×10^{6} M^{-1}s^{-1}
Dissociation of complex VnC5b8	k^{-}_{VnC5b8}	4.0×10^{-3} s^{-1}	4.0×10^{-4} – 4.0×10^{-2} s^{-1}
Association of Vn to C5b9	k^{+}_{VnC5b9}	2.4×10^{5} M^{-1}s^{-1}	2.4×10^{4} – 2.4×10^{6} M^{-1}s^{-1}
Dissociation of complex VnC5b9	k^{-}_{VnC5b9}	4.0×10^{-3} s^{-1}	4.0×10^{-4} – 4.0×10^{-2} s^{-1}
Association of CD59 to C5b8	$k^{+}_{\mathrm{C5b8CD59}}$	1.0×10^{6} M^{-1}s^{-1}	1.0×10^{5} – 1.0×10^{7} M^{-1}s^{-1}
Dissociation of complex C5b8CD59	$k^{-}_{\mathrm{C5b8CD59}}$	2.0×10^{-4} s^{-1}	2.0×10^{-5} – 2.0×10^{-3} s^{-1}

Association of CD59 to C5b9	$k^{+}_{\mathrm{C5b9CD59}}$	1.0×10^{6} $M^{-1}s^{-1}$	$1.0\times10^{5} - 1.0\times10^{7}$ $M^{-1}s^{-1}$
Dissociation of complex C5b9CD59	$k^{-}_{\mathrm{C5b9CD59}}$	2.0×10^{-4} s^{-1}	$2.0\times10^{-5} - 2.0\times10^{-3}$ s^{-1}
Cleavage of C3 by C3 convertase, $C3(H_2O)Bb$	k_{cat} $C3(H_2O)Bb$<br>K_m $C3(H_2O)Bb$	1.8 s^{-1}<br>10.6×10^{-6} M	$0.18 - 18$ s^{-1}<br>$10.6\times10^{-7} - 10.6\times10^{-5}$ M
Cleavage of C3 by C3 convertase, C3bBb	k_{cat} C3bBb<br>K_m C3bBb	1.8 s^{-1}<br>5.9×10^{-6} M	$0.18 - 18$ s^{-1}<br>$5.9\times10^{-7} - 5.9\times10^{-5}$ M
Cleavage of C5 by C3 convertase, C3bBb	k_{cat} C3bBb<br>K_m C3bBb	1.1×10^{-2} s^{-1}<br>24.0×10^{-6} M	$1.1\times10^{-3} - 1.1\times10^{-1}$ s^{-1}<br>$24.0\times10^{-7} - 24.0\times10^{-5}$ M
Cleavage of C3 by C3 convertase, C4bC2a	k_{cat} C4bC2a<br>K_m C4bC2a	3.2 s^{-1}<br>1.8×10^{-6} M	$0.32 - 32$ s^{-1}<br>$1.8\times10^{-7} - 1.8\times10^{-5}$ M
Cleavage of C5 by the C3 convertase, C4bC2a	k_{cat} C4bC2a<br>K_m C4bC2a	2.2×10^{-2} s^{-1}<br>8.9×10^{-6} M	$2.2\times10^{-3} - 2.2\times10^{-1}$ s^{-1}<br>$8.9\times10^{-7} - 8.9\times10^{-5}$ M
Cleavage of C4 by activated C1, C1*	k_{cat} C1*<br>K_m C1*	5.4 s^{-1}<br>6100×10^{-9} M	$0.54 - 54$ s^{-1}<br>$6100\times10^{-10} - 6100\times10^{-8}$ M
Cleavage of C2 by activated C1, C1*	k_{cat} C1*<br>K_m C1*	5.1 s^{-1}<br>6.1×10^{-6} M	$0.51 - 51$ s^{-1}<br>$6.1\times10^{-7} - 6.1\times10^{-5}$ M
Activation of complex C3bB by enzyme Factor D	k_{cat} C3bB<br>K_m C3bB	5.0 s^{-1}<br>2.5×10^{-6} M	$0.50 - 50$ s^{-1}<br>$2.5\times10^{-7} - 2.5\times10^{-5}$ M
Activation of complex $C3(H_2O)B$ by enzyme Factor D	k_{cat} $C3(H_2O)B$<br>K_m $C3(H_2O)B$	5.0 s^{-1}<br>2.5×10^{-6} M	$0.50 - 50$ s^{-1}<br>$2.5\times10^{-7} - 2.5\times10^{-5}$ M
Cleavage of C3b by inhibitor Factor I	k_{cat} C3bH	1.3 s^{-1}	$0.13 - 13$ s^{-1}

	K_m C3bH	2.5×10^{-7} M	$2.5\times10^{-8} - 2.5\times10^{-6}$ M
Cleavage of C5 by the C5 convertase, C3bC3bBb	k_{cat} C3bC3bBb<br><br>K_m C3bC3bBb	3.0×10^{-3} s^{-1}<br><br>1.7×10^{-6} M	$3.0\times10^{-4} - 3.0\times10^{-2}$ s^{-1}<br><br>$1.7\times10^{-7} - 1.7\times10^{-5}$ M
Cleavage of C5 by the C5 convertase, C3bC4bBb	k_{cat} C3bC3bBb<br><br>K_m C3bC3bBb	3.0×10^{-3} s^{-1}<br><br>1.7×10^{-6} M	$3.0\times10^{-4} - 3.0\times10^{-2}$ s^{-1}<br><br>$1.7\times10^{-7} - 1.7\times10^{-5}$ M
Cleavage of C5 by the C5 convertase, C3bC4bC2a	k_{cat} C3bC4bC2a<br><br>K_m C3bC4bC2a	1.8×10^{-2} s^{-1}<br><br>5.1×10^{-9} M	$1.8\times10^{-3} - 1.8\times10^{-1}$ s^{-1}<br><br>$5.1\times10^{-10} - 5.1\times10^{-8}$ M
Cleavage of C5 by the C5 convertase, C4bC4bC2a	k_{cat} C4bC4bC2a<br><br>K_m C4bC4bC2a	3.0×10^{-2} s^{-1}<br><br>5.6×10^{-6} M	$3.0\times10^{-3} - 3.0\times10^{-1}$ s^{-1}<br><br>$5.6\times10^{-7} - 5.6\times10^{-5}$ M
Cleavage of C3a by Carboxypeptidase N, CPN	k_{cat} CPN<br><br>K_m CPN	57.9 s^{-1}<br><br>77.1×10^{-6} M	$5.79 - 579$ s^{-1}<br><br>$77.1\times10^{-7} - 77.1\times10^{-5}$ M
Cleavage of C5a by Carboxypeptidase N, CPN	k_{cat} CPN<br><br>K_m CPN	9.3 s^{-1}<br><br>602.2×10^{-6} M	$0.93 - 93$ s^{-1}<br><br>$602.2\times10^{-7} - 602.2\times10^{-5}$ M
