# Supplementary material for: A computational model for the evaluation of complement system regulation under homeostasis, disease, and drug intervention
Source: PLoS One. 2018 Jun 6;13(6):e0198644. doi: 10.1371/journal.pone.0198644 (PMC5991421; doi:10.1371/journal.pone.0198644)
Supplement: S1 Equations — System of 290 ordinary differential equations. (PDF) [file pone.0198644.s017.pdf]

S1 Equations. Mathematical model of complement system. System of 290 ordinary differential equations.

(i) Initiation (fluid phase)

S1

$$\frac{d[\mathrm{C3(H_2O)}]}{dt} = k^{+}_{\mathrm{C3(H_2O)}}[\mathrm{C3}] - k^{+}_{\mathrm{C3(H_2O)B}}[\mathrm{C3(H_2O)}][\mathrm{B}] + k^{-}_{\mathrm{C3(H_2O)B}}[\mathrm{C3(H_2O)B}] + k^{-}_{\mathrm{C3(H_2O)Bb}}[\mathrm{C3(H_2O)Bb}]$$
$$- k^{+}_{\mathrm{C3bH}}[\mathrm{C3(H_2O)}][\mathrm{H}] + k^{-}_{\mathrm{C3bH}}[\mathrm{C3(H_2O)H}] + k^{-}_{\mathrm{C3bBbH_{decay}}}[\mathrm{C3(H_2O)BbH}] - k^{+}_{\mathrm{C3bHL}}[\mathrm{C3(H_2O)}][\mathrm{HL}] + k^{-}_{\mathrm{C3bHL}}[\mathrm{C3(H_2O)HL}]$$
$$+ k^{-}_{\mathrm{C3bBbHL_{decay}}}[\mathrm{C3(H_2O)BbHL}]$$

S2

$$\frac{d[\mathrm{C3(H_2O)B}]}{dt} = k^{+}_{\mathrm{C3(H_2O)B}}[\mathrm{C3(H_2O)}][\mathrm{B}] - k^{-}_{\mathrm{C3(H_2O)B}}[\mathrm{C3(H_2O)B}]$$
$$-\left(\frac{k^{\mathrm{D}}_{\mathrm{catC3(H_2O)B}}[\mathrm{D}][\mathrm{C3(H_2O)B}]}{K^{\mathrm{D}}_{\mathrm{mC3(H_2O)B}} + [\mathrm{C3(H_2O)B}] + [\mathrm{fC3bB}] + [\mathrm{fC3bC3bB}] + [\mathrm{fC3bC3bBP}] + [\mathrm{IgGC3bC3bB}] + [\mathrm{IgGC3bC3bBP}] + [\mathrm{fC3bC4bB}] + [\mathrm{fC3bC4bBP}]} \cdots \frac{1}{+ [\mathrm{hC3bB}] + [\mathrm{hC3bC3bB}] + [\mathrm{hC3bC3bBP}] + [\mathrm{hC3bC4bB}] + [\mathrm{hC3bC4bBP}]}\right)$$

S3

$$\frac{d[\mathrm{C3(H_2O)Bb}]}{dt} =$$
$$\left(\frac{k^{\mathrm{D}}_{\mathrm{catC3(H_2O)B}}[\mathrm{D}][\mathrm{C3(H_2O)B}]}{K^{\mathrm{D}}_{\mathrm{mC3(H_2O)B}} + [\mathrm{C3(H_2O)B}] + [\mathrm{fC3bB}] + [\mathrm{fC3bC3bB}] + [\mathrm{fC3bC3bBP}] + [\mathrm{IgGC3bC3bB}] + [\mathrm{IgGC3bC3bBP}] + [\mathrm{fC3bC4bB}] + [\mathrm{fC3bC4bBP}]} \cdots \frac{1}{+ [\mathrm{hC3bB}] + [\mathrm{hC3bC3bB}] + [\mathrm{hC3bC3bBP}] + [\mathrm{hC3bC4bB}] + [\mathrm{hC3bC4bBP}]}\right)$$
$$- k^{-}_{\mathrm{C3(H_2O)Bb}}[\mathrm{C3(H_2O)Bb}] - k^{+}_{\mathrm{C3bH}}[\mathrm{C3(H_2O)Bb}][\mathrm{H}] + k^{-}_{\mathrm{C3bH}}[\mathrm{C3(H_2O)BbH}] - k^{+}_{\mathrm{C3bHL}}[\mathrm{C3(H_2O)Bb}][\mathrm{HL}] + k^{-}_{\mathrm{C3bHL}}[\mathrm{C3(H_2O)BbHL}]$$

S4

$$\frac{d[\mathrm{fC3b}]}{dt} = k^{+}_{\mathrm{fC3b}}[\mathrm{nfC3b}][\mathrm{H_2O}] - k^{+}_{\mathrm{C3bB}}[\mathrm{fC3b}][\mathrm{B}] + k^{-}_{\mathrm{C3bB}}[\mathrm{fC3bB}] + k^{-}_{\mathrm{C3bBb}}[\mathrm{fC3bBb}] - k^{+}_{\mathrm{C3bC3b}}[\mathrm{nfC3b}][\mathrm{fC3b}] - k^{+}_{\mathrm{C3bC4b}}[\mathrm{nfC4b}][\mathrm{fC3b}]$$
$$- k^{+}_{\mathrm{C3bH}}[\mathrm{fC3b}][\mathrm{H}] + k^{-}_{\mathrm{C3bH}}[\mathrm{fC3bH}] + k^{-}_{\mathrm{C3bBbH_{decay}}}[\mathrm{fC3bBbH}] - k^{+}_{\mathrm{C3bHL}}[\mathrm{fC3b}][\mathrm{HL}] + k^{-}_{\mathrm{C3bHL}}[\mathrm{fC3bHL}] + k^{-}_{\mathrm{C3bBbHL_{decay}}}[\mathrm{fC3bBbHL}]$$
$$- k^{+}_{\mathrm{C3bCR1}}[\mathrm{fC3b}][\mathrm{CR1}] + k^{-}_{\mathrm{C3bCR1}}[\mathrm{fC3bCR1}] + k^{-}_{\mathrm{C3bBbCR1_{decay}}}[\mathrm{fC3bBbCR1}]$$

S5

$$\frac{d[\mathrm{fC3bB}]}{dt} = k^{+}_{\mathrm{C3bB}}[\mathrm{fC3b}][\mathrm{B}] - k^{-}_{\mathrm{C3bB}}[\mathrm{fC3bB}]$$
$$-\left(\frac{k^{\mathrm{D}}_{\mathrm{catC3(H_2O)B}}[\mathrm{D}][\mathrm{fC3bB}]}{K^{\mathrm{D}}_{\mathrm{mC3(H_2O)B}} + [\mathrm{C3(H_2O)B}] + [\mathrm{fC3bB}] + [\mathrm{fC3bC3bB}] + [\mathrm{fC3bC3bBP}] + [\mathrm{IgGC3bC3bB}] + [\mathrm{IgGC3bC3bBP}] + [\mathrm{fC3bC4bB}] + [\mathrm{fC3bC4bBP}]} \cdots \frac{1}{+ [\mathrm{hC3bB}] + [\mathrm{hC3bC3bB}] + [\mathrm{hC3bC3bBP}] + [\mathrm{hC3bC4bB}] + [\mathrm{hC3bC4bBP}]}\right)$$

S6

$$\frac{d[\mathrm{fC3bBb}]}{dt} =$$

$$\left(\frac{k^{\mathrm{D}}_{\mathrm{catC3(H_2O)B}}[\mathrm{D}][\mathrm{fC3bB}]}{K^{\mathrm{D}}_{\mathrm{mC3(H_2O)B}} + [\mathrm{C3(H_2O)B}] + [\mathrm{fC3bB}] + [\mathrm{fC3bC3bB}] + [\mathrm{fC3bC3bBP}] + [\mathrm{IgGC3bC3bB}] + [\mathrm{IgGC3bC3bBP}] + [\mathrm{fC3bC4bB}] + [\mathrm{fC3bC4bBP}]} \cdots \right.$$

$$\left. \frac{1}{+ [\mathrm{hC3bB}] + [\mathrm{hC3bC3bB}] + [\mathrm{hC3bC3bBP}] + [\mathrm{hC3bC4bB}] + [\mathrm{hC3bC4bBP}]} \right)$$

$$- k^{-}_{\mathrm{C3bBb}}[\mathrm{fC3bBb}] - k^{+}_{\mathrm{C3bC3b}}[\mathrm{fC3bBb}][\mathrm{nfC3b}] - k^{+}_{\mathrm{C3bC4b}}[\mathrm{fC3bBb}][\mathrm{nfC4b}] - k^{+}_{\mathrm{C3bH}}[\mathrm{fC3bBb}][\mathrm{H}] + k^{-}_{\mathrm{C3bH}}[\mathrm{fC3bBbH}]$$

$$- k^{+}_{\mathrm{C3bHL}}[\mathrm{fC3bBb}][\mathrm{HL}] + k^{-}_{\mathrm{C3bHL}}[\mathrm{fC3bBbHL}] - k^{+}_{\mathrm{C3bCR1}}[\mathrm{fC3bBb}][\mathrm{CR1}] + k^{-}_{\mathrm{C3bCR1}}[\mathrm{fC3bBbCR1}]$$

S7

$$\frac{d[\mathrm{nfC3b}]}{dt} = \frac{k^{\mathrm{C3}}_{\mathrm{catC3(H_2O)Bb}}[\mathrm{C3}][\mathrm{C3(H_2O)Bb}]}{K^{\mathrm{C3}}_{\mathrm{mC3(H_2O)Bb}} + [\mathrm{C3}]} + \frac{k^{\mathrm{C3}}_{\mathrm{catC3bBb}}[\mathrm{C3}][\mathrm{fC3bBb}]}{K^{\mathrm{C3}}_{\mathrm{mC3bBb}}\left(1 + \frac{[\mathrm{C3}]}{K^{\mathrm{C3}}_{\mathrm{mC3bBb}}} + \frac{[\mathrm{C5}]}{K^{\mathrm{C5}}_{\mathrm{mC3bBb}}}\right)} + \frac{k^{\mathrm{C3}}_{\mathrm{catC3bBb}}[\mathrm{C3}][\mathrm{hC3bBb}]}{K^{\mathrm{C3}}_{\mathrm{mC3bBb}}\left(1 + \frac{[\mathrm{C3}]}{K^{\mathrm{C3}}_{\mathrm{mC3bBb}}} + \frac{[\mathrm{C5}]}{K^{\mathrm{C5}}_{\mathrm{mC3bBb}}}\right)}$$

$$+ \frac{k^{\mathrm{C3}}_{\mathrm{catC3bBb}}[\mathrm{C3}][\mathrm{hC3bBbP}]}{K^{\mathrm{C3}}_{\mathrm{mC3bBb}}\left(1 + \frac{[\mathrm{C3}]}{K^{\mathrm{C3}}_{\mathrm{mC3bBb}}} + \frac{[\mathrm{C5}]}{K^{\mathrm{C5}}_{\mathrm{mC3bBb}}}\right)} + \frac{k^{\mathrm{C3}}_{\mathrm{catC3bBb}}[\mathrm{C3}][\mathrm{IgGC3bC3bBb}]}{K^{\mathrm{C3}}_{\mathrm{mC3bBb}} + [\mathrm{C3}]} + \frac{k^{\mathrm{C3}}_{\mathrm{catC3bBb}}[\mathrm{C3}][\mathrm{IgGC3bC3bBbP}]}{K^{\mathrm{C3}}_{\mathrm{mC3bBb}} + [\mathrm{C3}]}$$

$$+ \frac{k^{\mathrm{C3}}_{\mathrm{catC3bBb}}[\mathrm{C3}][\mathrm{fC4bC2a}]}{K^{\mathrm{C3}}_{\mathrm{mC3bBb}}\left(1 + \frac{[\mathrm{C3}]}{K^{\mathrm{C3}}_{\mathrm{mC4bC2a}}} + \frac{[\mathrm{C5}]}{K^{\mathrm{C5}}_{\mathrm{mC4bC2a}}}\right)} + \frac{k^{\mathrm{C3}}_{\mathrm{catC3bBb}}[\mathrm{C3}][\mathrm{fC3bC3bBb}]}{K^{\mathrm{C3}}_{\mathrm{mC3bBb}}\left(1 + \frac{[\mathrm{C3}]}{K^{\mathrm{C3}}_{\mathrm{mC3bBb}}} + \frac{[\mathrm{C5}]}{K^{\mathrm{C5}}_{\mathrm{mC3bC3bBb}}}\right)}$$

$$+ \frac{k^{\mathrm{C3}}_{\mathrm{catC3bBb}}[\mathrm{C3}][\mathrm{fC3bC3bBbP}]}{K^{\mathrm{C3}}_{\mathrm{mC3bBb}}\left(1 + \frac{[\mathrm{C3}]}{K^{\mathrm{C3}}_{\mathrm{mC3bBb}}} + \frac{[\mathrm{C5}]}{K^{\mathrm{C5}}_{\mathrm{mC3bC3bBb}}}\right)} + \frac{k^{\mathrm{C3}}_{\mathrm{catC3bBb}}[\mathrm{C3}][\mathrm{fC3bC4bBb}]}{K^{\mathrm{C3}}_{\mathrm{mC3bBb}}\left(1 + \frac{[\mathrm{C3}]}{K^{\mathrm{C3}}_{\mathrm{mC3bBb}}} + \frac{[\mathrm{C5}]}{K^{\mathrm{C5}}_{\mathrm{mC3bC4bBb}}}\right)}$$

$$+ \frac{k^{\mathrm{C3}}_{\mathrm{catC3bBb}}[\mathrm{C3}][\mathrm{fC3bC4bBbP}]}{K^{\mathrm{C3}}_{\mathrm{mC3bBb}}\left(1 + \frac{[\mathrm{C3}]}{K^{\mathrm{C3}}_{\mathrm{mC3bBb}}} + \frac{[\mathrm{C5}]}{K^{\mathrm{C5}}_{\mathrm{mC3bC4bBb}}}\right)} + \frac{k^{\mathrm{C3}}_{\mathrm{catC4bC2a}}[\mathrm{C3}][\mathrm{fC3bC4bC2a}]}{K^{\mathrm{C3}}_{\mathrm{mC4bC2a}}\left(1 + \frac{[\mathrm{C3}]}{K^{\mathrm{C3}}_{\mathrm{mC4bC2a}}} + \frac{[\mathrm{C5}]}{K^{\mathrm{C5}}_{\mathrm{mC3bC4bC2a}}}\right)}$$

$$+ \frac{k^{\mathrm{C3}}_{\mathrm{catC4bC2a}}[\mathrm{C3}][\mathrm{fC4bC4bC2a}]}{K^{\mathrm{C3}}_{\mathrm{mC4bC2a}}\left(1 + \frac{[\mathrm{C3}]}{K^{\mathrm{C3}}_{\mathrm{mC4bC2a}}} + \frac{[\mathrm{C5}]}{K^{\mathrm{C5}}_{\mathrm{mC4bC4bC2a}}}\right)} + \frac{k^{\mathrm{C3}}_{\mathrm{catC3bBb}}[\mathrm{C3}][\mathrm{hC3bC3bBb}]}{K^{\mathrm{C3}}_{\mathrm{mC3bBb}}\left(1 + \frac{[\mathrm{C3}]}{K^{\mathrm{C3}}_{\mathrm{mC3bBb}}} + \frac{[\mathrm{C5}]}{K^{\mathrm{C5}}_{\mathrm{mC3bC3bBb}}}\right)}$$

$$+ \frac{k^{\mathrm{C3}}_{\mathrm{catC3bBb}}[\mathrm{C3}][\mathrm{hC3bC3bBbP}]}{K^{\mathrm{C3}}_{\mathrm{mC3bBb}}\left(1 + \frac{[\mathrm{C3}]}{K^{\mathrm{C3}}_{\mathrm{mC3bBb}}} + \frac{[\mathrm{C5}]}{K^{\mathrm{C5}}_{\mathrm{mC3bC3bBb}}}\right)} + \frac{k^{\mathrm{C3}}_{\mathrm{catC3bBb}}[\mathrm{C3}][\mathrm{hC3bC4bBb}]}{K^{\mathrm{C3}}_{\mathrm{mC3bBb}}\left(1 + \frac{[\mathrm{C3}]}{K^{\mathrm{C3}}_{\mathrm{mC3bBb}}} + \frac{[\mathrm{C5}]}{K^{\mathrm{C5}}_{\mathrm{mC3bC4bBb}}}\right)}$$

$$+ \frac{k^{\mathrm{C3}}_{\mathrm{catC3bBb}}[\mathrm{C3}][\mathrm{hC3bC4bBbP}]}{K^{\mathrm{C3}}_{\mathrm{mC3bBb}}\left(1 + \frac{[\mathrm{C3}]}{K^{\mathrm{C3}}_{\mathrm{mC3bBb}}} + \frac{[\mathrm{C5}]}{K^{\mathrm{C5}}_{\mathrm{mC3bC4bBb}}}\right)} - k^{+}_{\mathrm{IgGC3b}}[\mathrm{IgG}][\mathrm{nfC3b}] - k^{+}_{\mathrm{C3bC3b}}[\mathrm{nfC3b}][\mathrm{nfC3b}] - k^{+}_{\mathrm{C3bC4b}}[\mathrm{nfC3b}][\mathrm{nfC4b}]$$

$$- k^{+}_{\mathrm{fC3b}}[\mathrm{nfC3b}][\mathrm{H_2O}] - k^{+}_{\mathrm{C3bC3b}}[\mathrm{IgGnfC3b}][\mathrm{nfC3b}] - k^{+}_{\mathrm{C3bC3b}}[\mathrm{IgGfC3b}][\mathrm{nfC3b}] - k^{+}_{\mathrm{C3bC3b}}[\mathrm{fC3bBb}][\mathrm{nfC3b}]$$

$$- k^{+}_{\mathrm{C3bC4b}}[\mathrm{nfC3b}][\mathrm{fC4b}] - k^{+}_{\mathrm{C3bC3b}}[\mathrm{nfC3b}][\mathrm{fC3b}] - k^{+}_{\mathrm{C3bC4b}}[\mathrm{fC4bC2a}][\mathrm{nfC3b}] - k^{+}_{\mathrm{C3bC4b}}[\mathrm{hC4b}][\mathrm{nfC3b}]$$

$$- k^{+}_{\mathrm{hC3b}}[\mathrm{Surface_{host}}][\mathrm{nfC3b}] - k^{+}_{\mathrm{C3bC3b}}[\mathrm{hC3b}][\mathrm{nfC3b}] - k^{+}_{\mathrm{C3bC3b}}[\mathrm{hC3bBb}][\mathrm{nfC3b}] - k^{+}_{\mathrm{C3bC3b}}[\mathrm{hC3bBbP}][\mathrm{nfC3b}]$$

S8

$$\frac{d[\mathrm{H_2O}]}{dt} = -k^{+}_{\mathrm{fC3b}}[\mathrm{nfC3b}][\mathrm{H_2O}] - k^{+}_{\mathrm{fC3b}}[\mathrm{npC3b}][\mathrm{H_2O}] - k^{+}_{\mathrm{fC3b}}[\mathrm{nhC3b}][\mathrm{H_2O}]$$

S9

$$\frac{d[\mathrm{nfC3bC3b}]}{dt} = k^{+}_{\mathrm{C3bC3b}}[\mathrm{nfC3b}][\mathrm{nfC3b}] - k^{+}_{\mathrm{fC3b}}[\mathrm{nfC3bnC3b}][\mathrm{H_2O}]$$

S10

$$\frac{d[\mathrm{fC3bC3b}]}{dt} = k^{+}_{\mathrm{C3bC3b}}[\mathrm{nfC3b}][\mathrm{fC3b}] + k^{+}_{\mathrm{fC3b}}[\mathrm{nfC3bnC3b}][\mathrm{H_2O}] - k^{+}_{\mathrm{C3bB}}[\mathrm{fC3bC3b}][\mathrm{B}] + k^{-}_{\mathrm{C3bB}}[\mathrm{fC3bC3bB}] - k^{+}_{\mathrm{C3bP}}[\mathrm{fC3bC3b}][\mathrm{P}]$$

$$+ k^{-}_{\mathrm{C3bP}}[\mathrm{fC3bC3bP}] + k^{-}_{\mathrm{C3bC3bBb}}[\mathrm{fC3bC3bBb}] + k^{-}_{\mathrm{C3bC3bBbP}}[\mathrm{fC3bC3bBbP}] - k^{+}_{\mathrm{C3bH}}[\mathrm{fC3bC3b}][\mathrm{H}] + k^{-}_{\mathrm{C3bH}}[\mathrm{fC3bC3bH}]$$

$$+ k^{-}_{\mathrm{C3bBbH}_{\mathrm{decay}}}[\mathrm{fC3bC3bBbH}] - k^{+}_{\mathrm{C3bHL}}[\mathrm{fC3bC3b}][\mathrm{HL}] + k^{-}_{\mathrm{C3bHL}}[\mathrm{fC3bC3bHL}] + k^{-}_{\mathrm{C3bBbHL}_{\mathrm{decay}}}[\mathrm{fC3bC3bBbHL}]$$

$$- k^{+}_{\mathrm{C3bC3bCR1}}[\mathrm{fC3bC3b}][\mathrm{CR1}] + k^{-}_{\mathrm{C3bC3bCR1}}[\mathrm{fC3bC3bCR1}] + k^{-}_{\mathrm{C3bBbCR1}_{\mathrm{decay}}}[\mathrm{fC3bC3bBbCR1}]$$

S11

$$\frac{d[\mathrm{fC3bC3bB}]}{dt} = k^{+}_{\mathrm{C3bB}}[\mathrm{fC3bC3b}][\mathrm{B}] - k^{-}_{\mathrm{C3bB}}[\mathrm{fC3bC3bB}]$$

$$-\left(\frac{k^{\mathrm{D}}_{\mathrm{catC3(H_2O)B}}[\mathrm{D}][\mathrm{fC3bC3bB}]}{K^{\mathrm{D}}_{\mathrm{mC3(H_2O)B}} + [\mathrm{C3(H_2O)B}] + [\mathrm{fC3bB}] + [\mathrm{fC3bC3bB}] + [\mathrm{fC3bC3bBP}] + [\mathrm{IgGC3bC3bB}] + [\mathrm{IgGC3bC3bBP}] + [\mathrm{fC3bC4bB}] + [\mathrm{fC3bC4bBP}]} \cdots \frac{1}{+ [\mathrm{hC3bB}] + [\mathrm{hC3bC3bB}] + [\mathrm{hC3bC3bBP}] + [\mathrm{hC3bC4bB}] + [\mathrm{hC3bC4bBP}]}\right)$$

S12

$$\frac{d[\mathrm{fC3bC3bBb}]}{dt} =$$

$$\left(\frac{k^{\mathrm{D}}_{\mathrm{catC3(H_2O)B}}[\mathrm{D}][\mathrm{fC3bC3bB}]}{K^{\mathrm{D}}_{\mathrm{mC3(H_2O)B}} + [\mathrm{C3(H_2O)B}] + [\mathrm{fC3bB}] + [\mathrm{fC3bC3bB}] + [\mathrm{fC3bC3bBP}] + [\mathrm{IgGC3bC3bB}] + [\mathrm{IgGC3bC3bBP}] + [\mathrm{fC3bC4bB}] + [\mathrm{fC3bC4bBP}]} \cdots \frac{1}{+ [\mathrm{hC3bB}] + [\mathrm{hC3bC3bB}] + [\mathrm{hC3bC3bBP}] + [\mathrm{hC3bC4bB}] + [\mathrm{hC3bC4bBP}]}\right)$$

$$+ k^{+}_{\mathrm{C3bC3b}}[\mathrm{fC3bBb}][\mathrm{nfC3b}] - k^{-}_{\mathrm{C3bC3bBb}}[\mathrm{fC3bC3bBb}] - k^{+}_{\mathrm{C3bP}}[\mathrm{fC3bC3bBb}][\mathrm{P}] + k^{-}_{\mathrm{C3bP}}[\mathrm{fC3bC3bBbP}]$$

$$+ k^{+}_{\mathrm{C5b7}}[\mathrm{fC3bC3bBbC5b6}][\mathrm{C7}] + k^{-}_{\mathrm{C5b*}}[\mathrm{fC3bC3bBbC5b}] - k^{+}_{\mathrm{C3bH}}[\mathrm{fC3bC3bBb}][\mathrm{H}] + k^{-}_{\mathrm{C3bH}}[\mathrm{fC3bC3bBbH}]$$

$$- k^{+}_{\mathrm{C3bHL}}[\mathrm{fC3bC3bBb}][\mathrm{HL}] + k^{-}_{\mathrm{C3bHL}}[\mathrm{fC3bC3bBbHL}] - k^{+}_{\mathrm{C3bC3bCR1}}[\mathrm{fC3bC3bBb}][\mathrm{CR1}] + k^{-}_{\mathrm{C3bC3bCR1}}[\mathrm{fC3bC3bBbCR1}]$$

S13

$$\frac{d[\mathrm{fC3bC3bP}]}{dt} = k^{+}_{\mathrm{C3bP}}[\mathrm{fC3bC3b}][\mathrm{P}] - k^{-}_{\mathrm{C3bP}}[\mathrm{fC3bC3bP}] - k^{+}_{\mathrm{C3bB}}[\mathrm{fC3bC3bP}][\mathrm{B}] + k^{-}_{\mathrm{C3bB}}[\mathrm{fC3bC3bPB}]$$

S14

$$\frac{d[\mathrm{fC3bC3bBP}]}{dt} = k^{+}_{\mathrm{C3bB}}[\mathrm{fC3bC3bP}][\mathrm{B}] - k^{-}_{\mathrm{C3bB}}[\mathrm{fC3bC3bBP}]$$

$$- \left(\frac{k^{\mathrm{D}}_{\mathrm{catC3(H_2O)B}}[\mathrm{D}][\mathrm{fC3bC3bBP}]}{K^{\mathrm{D}}_{\mathrm{mC3(H_2O)B}} + [\mathrm{C3(H_2O)B}] + [\mathrm{fC3bB}] + [\mathrm{fC3bC3bB}] + [\mathrm{fC3bC3bBP}] + [\mathrm{IgGC3bC3bB}] + [\mathrm{IgGC3bC3bBP}] + [\mathrm{fC3bC4bB}] + [\mathrm{fC3bC4bBP}]} \cdots \\ \frac{1}{+ [\mathrm{hC3bB}] + [\mathrm{hC3bC3bB}] + [\mathrm{hC3bC3bBP}] + [\mathrm{hC3bC4bB}] + [\mathrm{hC3bC4bBP}]} \right)$$

S15

$$\frac{d[\mathrm{fC3bC3bBbP}]}{dt} =$$

$$\left(\frac{k^{\mathrm{D}}_{\mathrm{catC3(H_2O)B}}[\mathrm{D}][\mathrm{fC3bC3bBP}]}{K^{\mathrm{D}}_{\mathrm{mC3(H_2O)B}} + [\mathrm{C3(H_2O)B}] + [\mathrm{fC3bB}] + [\mathrm{fC3bC3bB}] + [\mathrm{fC3bC3bBP}] + [\mathrm{IgGC3bC3bB}] + [\mathrm{IgGC3bC3bBP}] + [\mathrm{fC3bC4bB}] + [\mathrm{fC3bC4bBP}]} \cdots \\ \frac{1}{+ [\mathrm{hC3bB}] + [\mathrm{hC3bC3bB}] + [\mathrm{hC3bC3bBP}] + [\mathrm{hC3bC4bB}] + [\mathrm{hC3bC4bBP}]} \right)$$

$$+ k^{+}_{\mathrm{C3bP}}[\mathrm{fC3bC3bBb}][\mathrm{P}] - k^{-}_{\mathrm{C3bP}}[\mathrm{fC3bC3bBbP}] - k^{-}_{\mathrm{C3bC3bBbP}}[\mathrm{fC3bC3bBbP}] + k^{+}_{\mathrm{C5b7}}[\mathrm{fC3bC3bBbPC5b6}][\mathrm{C7}]$$

$$+ k^{-}_{\mathrm{C5b*}}[\mathrm{fC3bC3bBbPC5b}]$$

S16

$$\frac{d[\mathrm{IgGnfC3b}]}{dt} = k^{+}_{\mathrm{IgGC3b}}[\mathrm{IgG}][\mathrm{nfC3b}] - k^{+}_{\mathrm{C3bC3b}}[\mathrm{IgGnfC3b}][\mathrm{nfC3b}] - k^{+}_{\mathrm{fC3b}}[\mathrm{IgGnfC3b}][\mathrm{H_2O}]$$

S17

$$\frac{d[\mathrm{IgGfC3b}]}{dt} = k^{+}_{\mathrm{fC3b}}[\mathrm{IgGnfC3b}][\mathrm{H_2O}] - k^{+}_{\mathrm{C3bC3b}}[\mathrm{IgGfC3b}][\mathrm{nfC3b}]$$

S18

$$\frac{d[\mathrm{IgGnfC3bC3b}]}{dt} = k^{+}_{\mathrm{C3bC3b}}[\mathrm{IgGnfC3b}][\mathrm{nfC3b}] - k^{+}_{\mathrm{fC3b}}[\mathrm{IgGnfC3bC3b}][\mathrm{H_2O}]$$

S19

$$\frac{d[\mathrm{IgGC3bC3b}]}{dt} = k^{+}_{\mathrm{fC3b}}[\mathrm{IgGnfC3bC3b}][\mathrm{H_2O}] + k^{+}_{\mathrm{C3bC3b}}[\mathrm{IgGfC3b}][\mathrm{nfC3b}] - k^{+}_{\mathrm{C3bB}}[\mathrm{IgGC3bC3b}][\mathrm{B}] + k^{-}_{\mathrm{C3bB}}[\mathrm{IgGC3bC3bB}] - k^{+}_{\mathrm{C3bP}}[\mathrm{IgGC3bC3b}][\mathrm{P}]$$

$$+ k^{-}_{\mathrm{C3bP}}[\mathrm{IgGC3bC3bP}] + k^{-}_{\mathrm{C3bC3bBb}}[\mathrm{IgGC3bC3bBb}] + k^{-}_{\mathrm{C3bC3bBbP}}[\mathrm{IgGC3bC3bBbP}] - k^{+}_{\mathrm{C3bH}}[\mathrm{IgGC3bC3b}][\mathrm{H}] + k^{-}_{\mathrm{C3bH}}[\mathrm{IgGC3bC3bH}]$$

$$+ k^{-}_{\mathrm{C3bBbH_{decay}}}[\mathrm{IgGC3bC3bBbH}] - k^{+}_{\mathrm{C3bHL}}[\mathrm{IgGC3bC3b}][\mathrm{HL}] + k^{-}_{\mathrm{C3bHL}}[\mathrm{IgGC3bC3bHL}] + k^{-}_{\mathrm{C3bBbHL_{decay}}}[\mathrm{IgGC3bC3bBbHL}]$$

$$- k^{+}_{\mathrm{C3bC3bCR1}}[\mathrm{IgGC3bC3b}][\mathrm{CR1}] + k^{-}_{\mathrm{C3bC3bCR1}}[\mathrm{IgGC3bC3bCR1}] + k^{-}_{\mathrm{C3bBbCR1_{decay}}}[\mathrm{IgGC3bC3bBbCR1}]$$

S20

$$\frac{d[\text{IgGC3bC3bB}]}{dt} = k^{+}_{\text{C3bB}}[\text{IgGC3bC3b}][\text{B}] - k^{-}_{\text{C3bB}}[\text{IgGC3bC3bB}]$$

$$-\left(\frac{k^{\text{D}}_{\text{catC3(H}_2\text{O)B}}[\text{D}][\text{IgGC3bC3bB}]}{K^{\text{D}}_{\text{mC3(H}_2\text{O)B}} + [\text{C3(H}_2\text{O)B}] + [\text{fC3bB}] + [\text{fC3bC3bB}] + [\text{fC3bC3bBP}] + [\text{IgGC3bC3bB}] + [\text{IgGC3bC3bBP}] + [\text{fC3bC4bB}] + [\text{fC3bC4bBP}]} \cdots \frac{1}{+[\text{hC3bB}] + [\text{hC3bC3bB}] + [\text{hC3bC3bBP}] + [\text{hC3bC4bB}] + [\text{hC3bC4bBP}]}\right)$$

S21

$$\frac{d[\text{IgGC3bC3bBb}]}{dt} =$$

$$\left(\frac{k^{\text{D}}_{\text{catC3(H}_2\text{O)B}}[\text{D}][\text{IgGC3bC3bB}]}{K^{\text{D}}_{\text{mC3(H}_2\text{O)B}} + [\text{C3(H}_2\text{O)B}] + [\text{fC3bB}] + [\text{fC3bC3bB}] + [\text{fC3bC3bBP}] + [\text{IgGC3bC3bB}] + [\text{IgGC3bC3bBP}] + [\text{fC3bC4bB}] + [\text{fC3bC4bBP}]} \cdots \frac{1}{+[\text{hC3bB}] + [\text{hC3bC3bB}] + [\text{hC3bC3bBP}] + [\text{hC3bC4bB}] + [\text{hC3bC4bBP}]}\right)$$

$$- k^{-}_{\text{C3bC3bBb}}[\text{IgGC3bC3bBb}] - k^{+}_{\text{C3bP}}[\text{IgGC3bC3bBb}][\text{P}] + k^{-}_{\text{C3bP}}[\text{IgGC3bC3bBbP}]$$

$$- k^{+}_{\text{C3bH}}[\text{IgGC3bC3bBb}][\text{H}] + k^{-}_{\text{C3bH}}[\text{IgGC3bC3bBbH}] - k^{+}_{\text{C3bHL}}[\text{IgGC3bC3bBb}][\text{HL}] + k^{-}_{\text{C3bHL}}[\text{IgGC3bC3bBbHL}]$$

$$- k^{+}_{\text{C3bC3bCR1}}[\text{IgGC3bC3bBb}][\text{CR1}] + k^{-}_{\text{C3bC3bCR1}}[\text{IgGC3bC3bBbCR1}]$$

S22

$$\frac{d[\text{IgGC3bC3bP}]}{dt} = k^{+}_{\text{C3bP}}[\text{IgGC3bC3b}][\text{P}] - k^{-}_{\text{C3bP}}[\text{IgGC3bC3bP}] - k^{+}_{\text{C3bB}}[\text{IgGC3bC3bP}][\text{B}] + k^{-}_{\text{C3bB}}[\text{IgGC3bC3bPB}]$$

S23

$$\frac{d[\text{IgGC3bC3bBP}]}{dt} = k^{+}_{\text{C3bB}}[\text{IgGC3bC3bP}][\text{B}] - k^{-}_{\text{C3bB}}[\text{IgGC3bC3bBP}]$$

$$-\left(\frac{k^{\text{D}}_{\text{catC3(H}_2\text{O)B}}[\text{D}][\text{IgGC3bC3bBP}]}{K^{\text{D}}_{\text{mC3(H}_2\text{O)B}} + [\text{C3(H}_2\text{O)B}] + [\text{fC3bB}] + [\text{fC3bC3bB}] + [\text{fC3bC3bBP}] + [\text{IgGC3bC3bB}] + [\text{IgGC3bC3bBP}] + [\text{fC3bC4bB}] + [\text{fC3bC4bBP}]} \cdots \frac{1}{+[\text{hC3bB}] + [\text{hC3bC3bB}] + [\text{hC3bC3bBP}] + [\text{hC3bC4bB}] + [\text{hC3bC4bBP}]}\right)$$

S24

$$\frac{d[\text{IgGC3bC3bBbP}]}{dt} =$$

$$\left(\frac{k^{\text{D}}_{\text{catC3(H}_2\text{O)B}}[\text{D}][\text{IgGC3bC3bBP}]}{K^{\text{D}}_{\text{mC3(H}_2\text{O)B}} + [\text{C3(H}_2\text{O)B}] + [\text{fC3bB}] + [\text{fC3bC3bB}] + [\text{fC3bC3bBP}] + [\text{IgGC3bC3bB}] + [\text{IgGC3bC3bBP}] + [\text{fC3bC4bB}] + [\text{fC3bC4bBP}]} \cdots \frac{1}{+[\text{hC3bB}] + [\text{hC3bC3bB}] + [\text{hC3bC3bBP}] + [\text{hC3bC4bB}] + [\text{hC3bC4bBP}]}\right)$$

$$+ k^{+}_{\text{C3bP}}[\text{IgGC3bC3bBb}][\text{P}] - k^{-}_{\text{C3bP}}[\text{IgGC3bC3bBbP}] - k^{-}_{\text{C3bC3bBbP}}[\text{IgGC3bC3bBbP}]$$

S25

$$\frac{d[\text{nfC3bC4b}]}{dt} = k^{+}_{\text{C3bC4b}}[\text{nfC3b}][\text{nfC4b}] - k^{+}_{\text{fC3bC4b}}[\text{nfC3bnC4b}][\text{H}_2\text{O}]$$

S26

$$\frac{d[\text{fC3bC4b}]}{dt} = k^{+}_{\text{fC3bC4b}}[\text{nfC3bnC4b}][\text{H}_2\text{O}] + k^{+}_{\text{C3bC4b}}[\text{nfC4b}][\text{fC3b}] + k^{+}_{\text{C3bC4b}}[\text{nfC3b}][\text{fC4b}] - k^{+}_{\text{C3bB}}[\text{fC3bC4b}][\text{B}] + k^{-}_{\text{C3bB}}[\text{fC3bC4bB}]$$
$$- k^{+}_{\text{C3bP}}[\text{fC3bC4b}][\text{P}] + k^{-}_{\text{C3bP}}[\text{fC3bC4bP}] + k^{-}_{\text{C3bC4bBb}}[\text{fC3bC4bBb}] + k^{-}_{\text{C3bC4bBbP}}[\text{fC3bC4bBbP}] - k^{+}_{\text{C4bC2}}[\text{fC3bC4b}][\text{C2}]$$
$$+ k^{-}_{\text{C4bC2}}[\text{fC3bC4bC2}] + k^{-}_{\text{C3bC4bC2a}}[\text{fC3bC4bC2a}] - k^{+}_{\text{C3bH}}[\text{fC3bC4b}][\text{H}] + k^{-}_{\text{C3bH}}[\text{fC3bC4bH}] + k^{-}_{\text{C3bBbH}_{\text{decay}}}[\text{fC3bC4bBbH}]$$
$$- k^{+}_{\text{C3bHL}}[\text{fC3bC4b}][\text{HL}] + k^{-}_{\text{C3bHL}}[\text{fC3bC4bHL}] + k^{-}_{\text{C3bBbHL}_{\text{decay}}}[\text{fC3bC4bBbHL}] - k^{+}_{\text{C3bC4bCR1}}[\text{fC3bC4b}][\text{CR1}] + k^{-}_{\text{C3bC4bCR1}}[\text{fC3bC4bCR1}]$$
$$+ k^{-}_{\text{C3bBbCR1}_{\text{decay}}}[\text{fC3bC4bBbCR1}] + k^{-}_{\text{C3bBbCR1}_{\text{decay}}}[\text{fC3bC4bC2aCR1}] - k^{+}_{\text{C4bC4BP}}[\text{fC3bC4b}][\text{C4BP}] + k^{-}_{\text{C4bC4BP}}[\text{fC3bC4bC4BP}]$$
$$+ k^{-}_{\text{C4bC2aC4BP}_{\text{decay}}}[\text{fC3bC4bC2aC4BP}]$$

S27

$$\frac{d[\text{fC3bC4bB}]}{dt} = k^{+}_{\text{C3bB}}[\text{fC3bC4b}][\text{B}] - k^{-}_{\text{C3bB}}[\text{fC3bC4bB}]$$
$$-\left(\frac{k^{\text{D}}_{\text{catC3(H}_2\text{O)B}}[\text{D}][\text{fC3bC4bB}]}{K^{\text{D}}_{\text{mC3(H}_2\text{O)B}} + [\text{C3(H}_2\text{O)B}] + [\text{fC3bB}] + [\text{fC3bC3bB}] + [\text{fC3bC3bBP}] + [\text{IgGC3bC3bB}] + [\text{IgGC3bC3bBP}] + [\text{fC3bC4bB}] + [\text{fC3bC4bBP}]} \cdots \frac{1}{+[\text{hC3bB}] + [\text{hC3bC3bB}] + [\text{hC3bC3bBP}] + [\text{hC3bC4bB}] + [\text{hC3bC4bBP}]}\right)$$

S28

$$\frac{d[\text{fC3bC4bBb}]}{dt} =$$
$$\left(\frac{k^{\text{D}}_{\text{catC3(H}_2\text{O)B}}[\text{D}][\text{fC3bC4bB}]}{K^{\text{D}}_{\text{mC3(H}_2\text{O)B}} + [\text{C3(H}_2\text{O)B}] + [\text{fC3bB}] + [\text{fC3bC3bB}] + [\text{fC3bC3bBP}] + [\text{IgGC3bC3bB}] + [\text{IgGC3bC3bBP}] + [\text{fC3bC4bB}] + [\text{fC3bC4bBP}]} \cdots \frac{1}{+[\text{hC3bB}] + [\text{hC3bC3bB}] + [\text{hC3bC3bBP}] + [\text{hC3bC4bB}] + [\text{hC3bC4bBP}]}\right)$$
$$- k^{+}_{\text{C3bP}}[\text{fC3bC4bBb}][\text{P}] + k^{-}_{\text{C3bP}}[\text{fC3bC4bBbP}] + k^{+}_{\text{C3bC4b}}[\text{fC3bBb}][\text{nfC4b}] - k^{-}_{\text{C3bC4bBb}}[\text{fC3bC4bBb}] + k^{+}_{\text{C5b7}}[\text{fC3bC4bBbC5b6}][\text{C7}]$$
$$+ k^{-}_{\text{C5b*}}[\text{fC3bC4bBbC5b}] - k^{+}_{\text{C3bH}}[\text{fC3bC4bBb}][\text{H}] + k^{-}_{\text{C3bH}}[\text{fC3bC4bBbH}] - k^{+}_{\text{C3bHL}}[\text{fC3bC4bBb}][\text{HL}] + k^{-}_{\text{C3bHL}}[\text{fC3bC4bBbHL}]$$
$$- k^{+}_{\text{C3bC4bCR1}}[\text{fC3bC4bBb}][\text{CR1}] + k^{-}_{\text{C3bC4bCR1}}[\text{fC3bC4bBbCR1}]$$

S29

$$\frac{d[\text{fC3bC4bP}]}{dt} = k^{+}_{\text{C3bP}}[\text{fC3bC4b}][\text{P}] - k^{-}_{\text{C3bP}}[\text{fC3bC4bP}] - k^{+}_{\text{C3bB}}[\text{fC3bC4bP}][\text{B}] + k^{-}_{\text{C3bB}}[\text{fC3bC4bPB}]$$

S30

$$\frac{d[\mathrm{fC3bC4bBP}]}{dt} = k^{+}_{\mathrm{C3bB}}[\mathrm{fC3bC4bP}][\mathrm{B}] - k^{-}_{\mathrm{C3bB}}[\mathrm{fC3bC4bBP}]$$
$$- \left(\frac{k^{\mathrm{D}}_{\mathrm{catC3(H_2O)B}}[\mathrm{D}][\mathrm{fC3bC4bBP}]}{K^{\mathrm{D}}_{\mathrm{mC3(H_2O)B}} + [\mathrm{C3(H_2O)B}] + [\mathrm{fC3bB}] + [\mathrm{fC3bC3bB}] + [\mathrm{fC3bC3bBP}] + [\mathrm{IgGC3bC3bB}] + [\mathrm{IgGC3bC3bBP}] + [\mathrm{fC3bC4bB}] + [\mathrm{fC3bC4bBP}]} \cdots \frac{1}{+ [\mathrm{hC3bB}] + [\mathrm{hC3bC3bB}] + [\mathrm{hC3bC3bBP}] + [\mathrm{hC3bC4bB}] + [\mathrm{hC3bC4bBP}]} \right)$$

S31

$$\frac{d[\mathrm{fC3bC4bBbP}]}{dt} =$$
$$\left(\frac{k^{\mathrm{D}}_{\mathrm{catC3(H_2O)B}}[\mathrm{D}][\mathrm{fC3bC4bBP}]}{K^{\mathrm{D}}_{\mathrm{mC3(H_2O)B}} + [\mathrm{C3(H_2O)B}] + [\mathrm{fC3bB}] + [\mathrm{fC3bC3bB}] + [\mathrm{fC3bC3bBP}] + [\mathrm{IgGC3bC3bB}] + [\mathrm{IgGC3bC3bBP}] + [\mathrm{fC3bC4bB}] + [\mathrm{fC3bC4bBP}]} \cdots \frac{1}{+ [\mathrm{hC3bB}] + [\mathrm{hC3bC3bB}] + [\mathrm{hC3bC3bBP}] + [\mathrm{hC3bC4bB}] + [\mathrm{hC3bC4bBP}]} \right)$$
$$+ k^{+}_{\mathrm{C3bP}}[\mathrm{fC3bC4bBb}][\mathrm{P}] - k^{-}_{\mathrm{C3bP}}[\mathrm{fC3bC4bBbP}] - k^{-}_{\mathrm{C3bC4bBbP}}[\mathrm{fC3bC4bBbP}] + k^{+}_{\mathrm{C5b7}}[\mathrm{fC3bC4bBbPC5b6}][\mathrm{C7}]$$
$$+ k^{-}_{\mathrm{C5b*}}[\mathrm{fC3bC4bBbPC5b}]$$

S32

$$\frac{d[\mathrm{fC3bC4bC2}]}{dt} = k^{+}_{\mathrm{C4bC2}}[\mathrm{fC3bC4b}][\mathrm{C2}] - k^{-}_{\mathrm{C4bC2}}[\mathrm{fC3bC4bC2}] - \frac{k^{\mathrm{C2}}_{\mathrm{catC1*}}[\mathrm{fC3bC4bC2}][\mathrm{C1*}]}{K^{\mathrm{C2}}_{\mathrm{mC1*}}\left(1 + \frac{[\mathrm{fC4bC2}]}{K^{\mathrm{C2}}_{\mathrm{mC1*}}} + \frac{[\mathrm{fC4bC4bC2}]}{K^{\mathrm{C2}}_{\mathrm{mC1*}}} + \frac{[\mathrm{fC3bC4bC2}]}{K^{\mathrm{C2}}_{\mathrm{mC1*}}} + \frac{[\mathrm{C4}]}{K^{\mathrm{C4}}_{\mathrm{mC1*}}}\right)}$$

S33

$$\frac{d[\mathrm{fC3bC4bC2a}]}{dt} = \frac{k^{\mathrm{C2}}_{\mathrm{catC1*}}[\mathrm{fC3bC4bC2}][\mathrm{C1*}]}{K^{\mathrm{C2}}_{\mathrm{mC1*}}\left(1 + \frac{[\mathrm{fC4bC2}]}{K^{\mathrm{C2}}_{\mathrm{mC1*}}} + \frac{[\mathrm{fC4bC4bC2}]}{K^{\mathrm{C2}}_{\mathrm{mC1*}}} + \frac{[\mathrm{fC3bC4bC2}]}{K^{\mathrm{C2}}_{\mathrm{mC1*}}} + \frac{[\mathrm{C4}]}{K^{\mathrm{C4}}_{\mathrm{mC1*}}}\right)} + k^{+}_{\mathrm{C3bC4b}}[\mathrm{fC4bC2a}][\mathrm{nfC3b}]$$
$$- k^{-}_{\mathrm{C3bC4bC2a}}[\mathrm{fC3bC4bC2a}] + k^{-}_{\mathrm{C5b*}}[\mathrm{fC3bC4bC2aC5b}] + k^{+}_{\mathrm{C5b7}}[\mathrm{fC3bC4bC2aC5b6}][\mathrm{C7}] - k^{+}_{\mathrm{C3bC4bCR1}}[\mathrm{fC3bC4bC2a}][\mathrm{CR1}]$$
$$+ k^{-}_{\mathrm{C3bC4bCR1}}[\mathrm{fC3bC4bC2aCR1}] - k^{+}_{\mathrm{C4bC4BP}}[\mathrm{fC3bC4bC2a}][\mathrm{C4BP}] + k^{-}_{\mathrm{C4bC4BP}}[\mathrm{fC3bC4bC2aC4BP}]$$

S34

$$\frac{d[\mathrm{nfC4b}]}{dt} = \frac{k^{\mathrm{C4}}_{\mathrm{catC1*}}[\mathrm{C4}][\mathrm{C1*}]}{K^{\mathrm{C4}}_{\mathrm{mC1*}}\left(1 + \frac{[\mathrm{fC4bC2}]}{K^{\mathrm{C2}}_{\mathrm{mC1*}}} + \frac{[\mathrm{fC4bC4bC2}]}{K^{\mathrm{C2}}_{\mathrm{mC1*}}} + \frac{[\mathrm{fC3bC4bC2}]}{K^{\mathrm{C2}}_{\mathrm{mC1*}}} + \frac{[\mathrm{C4}]}{K^{\mathrm{C4}}_{\mathrm{mC1*}}}\right)} - k^{+}_{\mathrm{fC4b}}[\mathrm{nfC4b}][\mathrm{H_2O}] - k^{+}_{\mathrm{C4bC4b}}[\mathrm{nfC4b}][\mathrm{nfC4b}]$$
$$- k^{+}_{\mathrm{C4bC4b}}[\mathrm{nfC4b}][\mathrm{fC4b}] - k^{+}_{\mathrm{C3bC4b}}[\mathrm{nfC4b}][\mathrm{nfC3b}] - k^{+}_{\mathrm{C3bC4b}}[\mathrm{nfC4b}][\mathrm{fC3b}] - k^{+}_{\mathrm{C3bC4b}}[\mathrm{fC3bBb}][\mathrm{nfC4b}] - k^{+}_{\mathrm{C4bC4b}}[\mathrm{fC4bC2a}][\mathrm{nfC4b}]$$
$$- k^{+}_{\mathrm{C4bC4BP}}[\mathrm{nfC4b}][\mathrm{C4BP}] - k^{+}_{\mathrm{hC4b}}[\mathrm{Surface_{host}}][\mathrm{nfC4b}] - k^{+}_{\mathrm{C4bC4b}}[\mathrm{hC4b}][\mathrm{nfC4b}] - k^{+}_{\mathrm{C3bC4b}}[\mathrm{hC3bBb}][\mathrm{nfC4b}]$$
$$- k^{+}_{\mathrm{C3bC4b}}[\mathrm{hC3bBbP}][\mathrm{nfC4b}] - k^{+}_{\mathrm{C3bC4b}}[\mathrm{hC3b}][\mathrm{nfC4b}]$$

S35

$$\frac{d[\text{fC4b}]}{dt}=k^{+}_{\text{fC4b}}[\text{nfC4b}][\text{H}_2\text{O}]-k^{+}_{\text{C4bC2}}[\text{fC4b}][\text{C2}]+k^{-}_{\text{C4bC2}}[\text{fC4bC2}]+k^{-}_{\text{C4bC2a}}[\text{fC4bC2a}]-k^{+}_{\text{C4bC4b}}[\text{nfC4b}][\text{fC4b}]-k^{+}_{\text{C3bC4b}}[\text{nfC3b}][\text{fC4b}]$$
$$-k^{+}_{\text{C4bC4BP}}[\text{fC4b}][\text{C4BP}]+k^{-}_{\text{C4bC4BP}}[\text{fC4bC4BP}]+k^{-}_{\text{C4bC2aC4BP}_{\text{decay}}}[\text{fC4bC2aC4BP}]-k^{+}_{\text{C4bCR1}}[\text{fC4b}][\text{CR1}]+k^{-}_{\text{C4bCR1}}[\text{fC4bCR1}]$$
$$+k^{-}_{\text{C4bC2aCR1}_{\text{decay}}}[\text{fC4bC2aCR1}]$$

S36

$$\frac{d[\text{fC4bC2}]}{dt}=k^{+}_{\text{C4bC2}}[\text{fC4b}][\text{C2}]-k^{-}_{\text{C4bC2}}[\text{fC4bC2}]-\frac{k^{\text{C2}}_{\text{catC1*}}[\text{fC4bC2}][\text{C1*}]}{K^{\text{C2}}_{\text{mC1*}}\left(1+\frac{[\text{fC4bC2}]}{K^{\text{C2}}_{\text{mC1*}}}+\frac{[\text{fC4bC4bC2}]}{K^{\text{C2}}_{\text{mC1*}}}+\frac{[\text{fC3bC4bC2}]}{K^{\text{C2}}_{\text{mC1*}}}+\frac{[\text{C4}]}{K^{\text{C4}}_{\text{mC1*}}}\right)}$$

S37

$$\frac{d[\text{fC4bC2a}]}{dt}=\frac{k^{\text{C2}}_{\text{catC1*}}[\text{fC4bC2}][\text{C1*}]}{K^{\text{C2}}_{\text{mC1*}}\left(1+\frac{[\text{fC4bC2}]}{K^{\text{C2}}_{\text{mC1*}}}+\frac{[\text{fC4bC4bC2}]}{K^{\text{C2}}_{\text{mC1*}}}+\frac{[\text{fC3bC4bC2}]}{K^{\text{C2}}_{\text{mC1*}}}+\frac{[\text{C4}]}{K^{\text{C4}}_{\text{mC1*}}}\right)}-k^{-}_{\text{C4bC2a}}[\text{fC4bC2a}]-k^{+}_{\text{C4bC4b}}[\text{fC4bC2a}][\text{nfC4b}]$$
$$-k^{+}_{\text{C3bC4b}}[\text{fC4bC2a}][\text{nfC3b}]-k^{+}_{\text{C4bCR1}}[\text{fC4bC2a}][\text{CR1}]+k^{-}_{\text{C4bCR1}}[\text{fC4bC2aCR1}]-k^{+}_{\text{C4bC4BP}}[\text{fC4bC2a}][\text{C4BP}]$$
$$+k^{-}_{\text{C4bC4BP}}[\text{fC4bC2aC4BP}]$$

S38

$$\frac{d[\text{nfC4bC4b}]}{dt}=k^{+}_{\text{C4bC4b}}[\text{nfC4bC4b}][\text{nfC4b}]-k^{+}_{\text{fC4bC4b}}[\text{nfC4bC4b}][\text{H}_2\text{O}]$$

S39

$$\frac{d[\text{fC4bC4b}]}{dt}=k^{+}_{\text{fC4b}}[\text{nfC4bC4b}][\text{H}_2\text{O}]+k^{+}_{\text{C4bC4b}}[\text{nfC4b}][\text{fC4b}]-k^{+}_{\text{C4bC2}}[\text{fC4bC4b}][\text{C2}]+k^{-}_{\text{C4bC2}}[\text{fC4bC4bC2}]+k^{-}_{\text{C4bC4bC2a}}[\text{fC4bC4bC2a}]$$
$$-k^{+}_{\text{C4bC4BP}}[\text{fC4bC4b}][\text{C4BP}]+k^{-}_{\text{C4bC4BP}}[\text{fC4bC4bC4BP}]+k^{-}_{\text{C4bC2aC4BP}_{\text{decay}}}[\text{fC4bC4bC2aC4BP}]-k^{+}_{\text{C4bC4bCR1}}[\text{fC4bC4b}][\text{CR1}]$$
$$+k^{-}_{\text{C4bC4bCR1}}[\text{fC4bC4bCR1}]+k^{-}_{\text{C4bC2aCR1}_{\text{decay}}}[\text{fC4bC4bC2aCR1}]$$

S40

$$\frac{d[\text{fC4bC4bC2}]}{dt}=k^{+}_{\text{C4bC2}}[\text{fC4bC4b}][\text{C2}]-k^{-}_{\text{C4bC2}}[\text{fC4bC4bC2}]-\frac{k^{\text{C2}}_{\text{catC1*}}[\text{fC4bC4bC2}][\text{C1*}]}{K^{\text{C2}}_{\text{mC1*}}\left(1+\frac{[\text{fC4bC2}]}{K^{\text{C2}}_{\text{mC1*}}}+\frac{[\text{fC4bC4bC2}]}{K^{\text{C2}}_{\text{mC1*}}}+\frac{[\text{fC3bC4bC2}]}{K^{\text{C2}}_{\text{mC1*}}}+\frac{[\text{C4}]}{K^{\text{C4}}_{\text{mC1*}}}\right)}$$

S41

$$\frac{d[\text{fC4bC4bC2a}]}{dt}=\frac{k^{\text{C2}}_{\text{catC1*}}[\text{fC4bC4bC2}][\text{C1*}]}{K^{\text{C2}}_{\text{mC1*}}\left(1+\frac{[\text{fC4bC2}]}{K^{\text{C2}}_{\text{mC1*}}}+\frac{[\text{fC4bC4bC2}]}{K^{\text{C2}}_{\text{mC1*}}}+\frac{[\text{fC3bC4bC2}]}{K^{\text{C2}}_{\text{mC1*}}}+\frac{[\text{C4}]}{K^{\text{C4}}_{\text{mC1*}}}\right)}+k^{+}_{\text{C4bC4b}}[\text{fC4bC2a}][\text{nfC4b}]$$
$$-k^{-}_{\text{C4bC4bC2a}}[\text{fC4bC4bC2a}]+k^{-}_{\text{C5b*}}[\text{fC4bC4bC2aC5b}]+k^{+}_{\text{C5b7}}[\text{fC4bC4bC2aC5b6}][\text{C7}]-k^{+}_{\text{C4bC4bCR1}}[\text{fC4bC4bC2a}][\text{CR1}]$$
$$+k^{-}_{\text{C4bC4bCR1}}[\text{fC4bC4bC2aCR1}]-k^{+}_{\text{C4bC4BP}}[\text{fC4bC4bC2a}][\text{C4BP}]+k^{-}_{\text{C4bC4BP}}[\text{fC4bC4bC2aC4BP}]$$

S42

$$\frac{d[\mathrm{fC5b}]}{dt}=\frac{k_{\mathrm{catC3bBb}}^{\mathrm{C5}}[\mathrm{C5}][\mathrm{fC3bBb}]}{K_{\mathrm{mC3bBb}}^{\mathrm{C5}}\left(1+\frac{[\mathrm{C3}]}{K_{\mathrm{mC3bBb}}^{\mathrm{C3}}}+\frac{[\mathrm{C5}]}{K_{\mathrm{mC3bBb}}^{\mathrm{C5}}}\right)}+\frac{k_{\mathrm{catC4bC2a}}^{\mathrm{C5}}[\mathrm{C5}][\mathrm{fC4bC2a}]}{K_{\mathrm{mC4bC2a}}^{\mathrm{C5}}\left(1+\frac{[\mathrm{C3}]}{K_{\mathrm{mC4bC2a}}^{\mathrm{C3}}}+\frac{[\mathrm{C5}]}{K_{\mathrm{mC4bC2a}}^{\mathrm{C5}}}\right)}$$

$$+\frac{k_{\mathrm{catC3bBb}}^{\mathrm{C5}}[\mathrm{C5}][\mathrm{hC3bBb}]}{K_{\mathrm{mC3bBb}}^{\mathrm{C5}}\left(1+\frac{[\mathrm{C3}]}{K_{\mathrm{mC3bBb}}^{\mathrm{C3}}}+\frac{[\mathrm{C5}]}{K_{\mathrm{mC3bBb}}^{\mathrm{C5}}}\right)}+\frac{k_{\mathrm{catC3bBb}}^{\mathrm{C5}}[\mathrm{C5}][\mathrm{hC3bBbP}]}{K_{\mathrm{mC3bBb}}^{\mathrm{C5}}\left(1+\frac{[\mathrm{C3}]}{K_{\mathrm{mC3bBb}}^{\mathrm{C3}}}+\frac{[\mathrm{C5}]}{K_{\mathrm{mC3bBb}}^{\mathrm{C5}}}\right)}-k_{\mathrm{C5b*}}^{-}[\mathrm{fC5b}]-k_{\mathrm{C5b6}}^{+}[\mathrm{fC5b}][\mathrm{C6}]+k_{\mathrm{C5b6}}^{-}[\mathrm{fC5b6}]$$

S43

$$\frac{d[\mathrm{fC5b6}]}{dt}=k_{\mathrm{C5b6}}^{+}[\mathrm{fC5b}][\mathrm{C6}]-k_{\mathrm{C5b6}}^{-}[\mathrm{fC5b6}]-k_{\mathrm{C5b7}}^{+}[\mathrm{fC5b6}][\mathrm{C7}]+k_{\mathrm{C5b7}}^{-}[\mathrm{fC5b7}]$$

S44

$$\frac{d[\mathrm{fC3bC3bBbC5b}]}{dt}=\frac{k_{\mathrm{catC3bC3bBb}}^{\mathrm{C5}}[\mathrm{C5}][\mathrm{fC3bC3bBb}]}{K_{\mathrm{mC3bC3bBb}}^{\mathrm{C5}}\left(1+\frac{[\mathrm{C3}]}{K_{\mathrm{mC3bBb}}^{\mathrm{C3}}}+\frac{[\mathrm{C5}]}{K_{\mathrm{mC3bC3bBb}}^{\mathrm{C5}}}\right)}-k_{\mathrm{C5b*}}^{-}[\mathrm{fC3bC3bBbC5b}]-k_{\mathrm{C5b6}}^{+}[\mathrm{fC3bC3bBbC5b}][\mathrm{C6}]$$

$$+k_{\mathrm{C5b6}}^{-}[\mathrm{fC3bC3bBbC5b6}]$$

S45

$$\frac{d[\mathrm{fC3bC3bBbC5b6}]}{dt}=k_{\mathrm{C5b6}}^{+}[\mathrm{fC3bC3bBbC5b}][\mathrm{C6}]-k_{\mathrm{C5b6}}^{-}[\mathrm{fC3bC3bBbC5b6}]-k_{\mathrm{C5b7}}^{+}[\mathrm{fC3bC3bBbC5b6}][\mathrm{C7}]$$

S46

$$\frac{d[\mathrm{fC3bC3bBbPC5b}]}{dt}=\frac{k_{\mathrm{catC3bC3bBb}}^{\mathrm{C5}}[\mathrm{C5}][\mathrm{fC3bC3bBbP}]}{K_{\mathrm{mC3bC3bBb}}^{\mathrm{C5}}\left(1+\frac{[\mathrm{C3}]}{K_{\mathrm{mC3bBb}}^{\mathrm{C3}}}+\frac{[\mathrm{C5}]}{K_{\mathrm{mC3bC3bBb}}^{\mathrm{C5}}}\right)}-k_{\mathrm{C5b*}}^{-}[\mathrm{fC3bC3bBbPC5b}]-k_{\mathrm{C5b6}}^{+}[\mathrm{fC3bC3bBbPC5b}][\mathrm{C6}]$$

$$+k_{\mathrm{C5b6}}^{-}[\mathrm{fC3bC3bBbPC5b6}]$$

S47

$$\frac{d[\mathrm{fC3bC3bBbPC5b6}]}{dt}=k_{\mathrm{C5b6}}^{+}[\mathrm{fC3bC3bBbPC5b}][\mathrm{C6}]-k_{\mathrm{C5b6}}^{-}[\mathrm{fC3bC3bBbPC5b6}]-k_{\mathrm{C5b7}}^{+}[\mathrm{fC3bC3bBbPC5b6}][\mathrm{C7}]$$

S48

$$\frac{d[\mathrm{fC3bC4bBbC5b}]}{dt}=\frac{k_{\mathrm{catC3bC4bBb}}^{\mathrm{C5}}[\mathrm{C5}][\mathrm{fC3bC4bBb}]}{K_{\mathrm{mC3bC4bBb}}^{\mathrm{C5}}\left(1+\frac{[\mathrm{C3}]}{K_{\mathrm{mC3bBb}}^{\mathrm{C3}}}+\frac{[\mathrm{C5}]}{K_{\mathrm{mC3bC4bBb}}^{\mathrm{C5}}}\right)}-k_{\mathrm{C5b*}}^{-}[\mathrm{fC3bC4bBbC5b}]-k_{\mathrm{C5b6}}^{+}[\mathrm{fC3bC4bBbC5b}][\mathrm{C6}]$$

$$+k_{\mathrm{C5b6}}^{-}[\mathrm{fC3bC4bBbC5b6}]$$

S49

$$\frac{d[\text{fC3bC4bBbC5b6}]}{dt} = k^{+}_{\text{C5b6}}[\text{fC3bC4bBbC5b}][\text{C6}] - k^{-}_{\text{C5b6}}[\text{fC3bC4bBbC5b6}] - k^{+}_{\text{C5b7}}[\text{fC3bC4bBbC5b6}][\text{C7}]$$

S50

$$\frac{d[\text{fC3bC4bBbPC5b}]}{dt} = \frac{k^{\text{C5}}_{\text{catC3bC4bBb}}[\text{C5}][\text{fC3bC4bBbP}]}{K^{\text{C5}}_{\text{mC3bC4bBb}}\left(1+\frac{[\text{C3}]}{K^{\text{C3}}_{\text{mC3bBb}}}+\frac{[\text{C5}]}{K^{\text{C5}}_{\text{mC3bC4bBb}}}\right)} - k^{-}_{\text{C5b*}}[\text{fC3bC4bBbC5bP}] - k^{+}_{\text{C5b6}}[\text{fC3bC4bBbC5bP}][\text{C6}]$$
$$+ k^{-}_{\text{C5b6}}[\text{fC3bC4bBbC5b6P}]$$

S51

$$\frac{d[\text{fC3bC4bBbPC5b6}]}{dt} = k^{+}_{\text{C5b6}}[\text{fC3bC4bBbPC5b}][\text{C6}] - k^{-}_{\text{C5b6}}[\text{fC3bC4bBbPC5b6}] - k^{+}_{\text{C5b7}}[\text{fC3bC4bBbPC5b6}][\text{C7}]$$

S52

$$\frac{d[\text{fC3bC4bC2aC5b}]}{dt} = \frac{k^{\text{C5}}_{\text{catC3bC4bC2a}}[\text{C5}][\text{fC3bC4bC2a}]}{K^{\text{C5}}_{\text{mC3bC4bC2a}}\left(1+\frac{[\text{C3}]}{K^{\text{C3}}_{\text{mC3bBb}}}+\frac{[\text{C5}]}{K^{\text{C5}}_{\text{mC3bC4bC2a}}}\right)} - k^{-}_{\text{C5b*}}[\text{fC3bC4bC2aC5b}] - k^{+}_{\text{C5b6}}[\text{fC3bC4bC2aC5b}][\text{C6}]$$
$$+ k^{-}_{\text{C5b6}}[\text{fC3bC4bC2aC5b6}]$$

S53

$$\frac{d[\text{fC3bC4bC2aC5b6}]}{dt} = k^{+}_{\text{C5b6}}[\text{fC3bC4bC2aC5b}][\text{C6}] - k^{-}_{\text{C5b6}}[\text{fC3bC4bC2aC5b6}] - k^{+}_{\text{C5b7}}[\text{fC3bC4bC2aC5b6}][\text{C7}]$$

S54

$$\frac{d[\text{fC4bC4bC2aC5b}]}{dt} = \frac{k^{\text{C5}}_{\text{catC4bC4bC2a}}[\text{C5}][\text{fC4bC4bC2a}]}{K^{\text{C5}}_{\text{mC4bC4bC2a}}\left(1+\frac{[\text{C3}]}{K^{\text{C3}}_{\text{mC3bBb}}}+\frac{[\text{C5}]}{K^{\text{C5}}_{\text{mC4bC4bC2a}}}\right)} - k^{-}_{\text{C5b*}}[\text{fC4bC4bC2aC5b}] - k^{+}_{\text{C5b6}}[\text{fC4bC4bC2aC5b}][\text{C6}]$$
$$+ k^{-}_{\text{C5b6}}[\text{fC4bC4bC2aC5b6}]$$

S55

$$\frac{d[\text{fC4bC4bC2aC5b6}]}{dt} = k^{+}_{\text{C5b6}}[\text{fC4bC4bC2aC5b}][\text{C6}] - k^{-}_{\text{C5b6}}[\text{fC4bC4bC2aC5b6}] - k^{+}_{\text{C5b7}}[\text{fC4bC4bC2aC5b6}][\text{C7}]$$

(ii) Amplification (host cell)

S56

$$\frac{d[\text{Surface}_{\text{host}}]}{dt} = -k^{+}_{\text{hC3b}}[\text{Surface}_{\text{host}}][\text{nfC3b}] - k^{+}_{\text{hC4b}}[\text{Surface}_{\text{host}}][\text{nfC4b}] - k^{+}_{\text{hC5b7}}[\text{Surface}_{\text{host}}][\text{fC5b7}]$$

S57

$$\frac{d[\text{hC3b}]}{dt} = k^{+}_{\text{hC3b}}[\text{Surface}_{\text{host}}][\text{nfC3b}] - k^{+}_{\text{C3bC3b}}[\text{hC3b}][\text{nfC3b}] - k^{+}_{\text{C3bC4b}}[\text{hC3b}][\text{nfC4b}] - k^{+}_{\text{C3bB}}[\text{hC3b}][\text{B}] + k^{-}_{\text{C3bB}}[\text{hC3bB}] + k^{-}_{\text{C3bBb}}[\text{hC3bBb}]$$
$$+ k^{-}_{\text{C3bBbP}}[\text{hC3bBbP}] - k^{+}_{\text{C3bH}}[\text{hC3b}][\text{H}] + k^{-}_{\text{C3bH}}[\text{hC3bH}] + k^{-}_{\text{C3bBbH}_{\text{decay}}}[\text{hC3bBbH}] - k^{+}_{\text{C3bHL}}[\text{hC3b}][\text{HL}] + k^{-}_{\text{C3bHL}}[\text{hC3bHL}]$$
$$+ k^{-}_{\text{C3bBbHL}_{\text{decay}}}[\text{hC3bBbHL}] - k^{+}_{\text{C3bCR1}}[\text{hC3b}][\text{CR1}] + k^{-}_{\text{C3bCR1}}[\text{hC3bCR1}] + k^{-}_{\text{C3bBbCR1}_{\text{decay}}}[\text{hC3bBbCR1}] + k^{-}_{\text{C3bBbDAF}_{\text{decay}}}[\text{hC3bBbDAF}]$$

S58

$$\frac{d[\text{hC3bB}]}{dt} = k^{+}_{\text{C3bB}}[\text{hC3b}][\text{B}] - k^{-}_{\text{C3bB}}[\text{hC3bB}]$$
$$-\left(\frac{k^{\text{D}}_{\text{catC3(H}_2\text{O)B}}[\text{D}][\text{hC3bB}]}{K^{\text{D}}_{\text{mC3(H}_2\text{O)B}} + [\text{C3(H}_2\text{O)B}] + [\text{fC3bB}] + [\text{fC3bC3bB}] + [\text{fC3bC3bBP}] + [\text{IgGC3bC3bB}] + [\text{IgGC3bC3bBP}] + [\text{fC3bC4bB}] + [\text{fC3bC4bBP}]} \cdots \frac{1}{+ [\text{hC3bB}] + [\text{hC3bC3bB}] + [\text{hC3bC3bBP}] + [\text{hC3bC4bB}] + [\text{hC3bC4bBP}]}\right)$$

S59

$$\frac{d[\text{hC3bBb}]}{dt} =$$
$$\left(\frac{k^{\text{D}}_{\text{catC3(H}_2\text{O)B}}[\text{D}][\text{hC3bB}]}{K^{\text{D}}_{\text{mC3(H}_2\text{O)B}} + [\text{C3(H}_2\text{O)B}] + [\text{fC3bB}] + [\text{fC3bC3bB}] + [\text{fC3bC3bBP}] + [\text{IgGC3bC3bB}] + [\text{IgGC3bC3bBP}] + [\text{fC3bC4bB}] + [\text{fC3bC4bBP}]} \cdots \frac{1}{+ [\text{hC3bB}] + [\text{hC3bC3bB}] + [\text{hC3bC3bBP}] + [\text{hC3bC4bB}] + [\text{hC3bC4bBP}]}\right)$$
$$- k^{-}_{\text{C3bBb}}[\text{hC3bBb}] - k^{+}_{\text{C3bC3b}}[\text{hC3bBb}][\text{nfC3b}] - k^{+}_{\text{C3bP}}[\text{hC3bBb}][\text{P}] + k^{-}_{\text{C3bP}}[\text{hC3bBbP}] - k^{+}_{\text{C3bC4b}}[\text{hC3bBb}][\text{nfC4b}]$$
$$- k^{+}_{\text{C3bH}}[\text{hC3bBb}][\text{H}] + k^{-}_{\text{C3bH}}[\text{hC3bBbH}] - k^{+}_{\text{C3bHL}}[\text{hC3bBb}][\text{HL}] + k^{-}_{\text{C3bHL}}[\text{hC3bBbHL}] - k^{+}_{\text{C3bCR1}}[\text{hC3bBb}][\text{CR1}]$$
$$+ k^{-}_{\text{C3bCR1}}[\text{hC3bBbCR1}] - k^{+}_{\text{C3bBbDAF}}[\text{hC3bBb}][\text{DAF}] + k^{-}_{\text{C3bBbDAF}}[\text{hC3bBbDAF}]$$

S60

$$\frac{d[\text{hC3bBbP}]}{dt} = k^{+}_{\text{C3bP}}[\text{hC3bBb}][\text{P}] - k^{-}_{\text{C3bP}}[\text{hC3bBbP}] - k^{-}_{\text{C3bBbP}}[\text{hC3bBbP}] - k^{+}_{\text{C3bC3b}}[\text{hC3bBbP}][\text{nfC3b}]$$

S61

$$\frac{d[\text{hC3bC3b}]}{dt} = k^{+}_{\text{C3bC3b}}[\text{hC3b}][\text{nfC3b}] - k^{+}_{\text{C3bB}}[\text{hC3bC3b}][\text{B}] + k^{-}_{\text{C3bB}}[\text{hC3bC3bB}] - k^{+}_{\text{C3bP}}[\text{hC3bC3b}][\text{P}]$$
$$+ k^{-}_{\text{C3bP}}[\text{hC3bC3bP}] + k^{-}_{\text{C3bC3bBb}}[\text{hC3bC3bBb}] + k^{-}_{\text{C3bC3bBbP}}[\text{hC3bC3bBbP}] - k^{+}_{\text{C3bH}}[\text{hC3bC3b}][\text{H}] + k^{-}_{\text{C3bH}}[\text{hC3bC3bH}]$$
$$+ k^{-}_{\text{C3bBbH}_{\text{decay}}}[\text{hC3bC3bBbH}] - k^{+}_{\text{C3bHL}}[\text{hC3bC3b}][\text{HL}] + k^{-}_{\text{C3bHL}}[\text{hC3bC3bHL}] + k^{-}_{\text{C3bBbHL}_{\text{decay}}}[\text{hC3bC3bBbHL}]$$
$$- k^{+}_{\text{C3bC3bCR1}}[\text{hC3bC3b}][\text{CR1}] + k^{-}_{\text{C3bC3bCR1}}[\text{hC3bC3bCR1}] + k^{-}_{\text{C3bBbCR1}_{\text{decay}}}[\text{hC3bC3bBbCR1}] + k^{-}_{\text{C3bBbDAF}_{\text{decay}}}[\text{hC3bC3bBbDAF}]$$

S62

$$\frac{d[\mathrm{hC3bC3bB}]}{dt}=k^{+}_{\mathrm{C3bB}}[\mathrm{hC3bC3b}][\mathrm{B}]-k^{-}_{\mathrm{C3bB}}[\mathrm{hC3bC3bB}]$$

$$-\left(\frac{k^{\mathrm{D}}_{\mathrm{catC3(H_2O)B}}[\mathrm{D}][\mathrm{hC3bC3bB}]}{K^{\mathrm{D}}_{\mathrm{mC3(H_2O)B}}+[\mathrm{C3(H_2O)B}]+[\mathrm{fC3bB}]+[\mathrm{fC3bC3bB}]+[\mathrm{fC3bC3bBP}]+[\mathrm{IgGC3bC3bB}]+[\mathrm{IgGC3bC3bBP}]+[\mathrm{fC3bC4bB}]+[\mathrm{fC3bC4bBP}]}\cdots\right.$$
$$\left.\frac{1}{+[\mathrm{hC3bB}]+[\mathrm{hC3bC3bB}]+[\mathrm{hC3bC3bBP}]+[\mathrm{hC3bC4bB}]+[\mathrm{hC3bC4bBP}]}\right)$$

S63

$$\frac{d[\mathrm{hC3bC3bBb}]}{dt}=$$

$$\left(\frac{k^{\mathrm{D}}_{\mathrm{catC3(H_2O)B}}[\mathrm{D}][\mathrm{hC3bC3bB}]}{K^{\mathrm{D}}_{\mathrm{mC3(H_2O)B}}+[\mathrm{C3(H_2O)B}]+[\mathrm{fC3bB}]+[\mathrm{fC3bC3bB}]+[\mathrm{fC3bC3bBP}]+[\mathrm{IgGC3bC3bB}]+[\mathrm{IgGC3bC3bBP}]+[\mathrm{fC3bC4bB}]+[\mathrm{fC3bC4bBP}]}\cdots\right.$$
$$\left.\frac{1}{+[\mathrm{hC3bB}]+[\mathrm{hC3bC3bB}]+[\mathrm{hC3bC3bBP}]+[\mathrm{hC3bC4bB}]+[\mathrm{hC3bC4bBP}]}\right)$$

$$+k^{+}_{\mathrm{C3bC3b}}[\mathrm{hC3bBb}][\mathrm{nfC3b}]-k^{-}_{\mathrm{C3bC3bBb}}[\mathrm{hC3bC3bBb}]-k^{+}_{\mathrm{C3bP}}[\mathrm{hC3bC3bBb}][\mathrm{P}]+k^{-}_{\mathrm{C3bP}}[\mathrm{hC3bC3bBbP}]$$

$$+k^{+}_{\mathrm{C5b7}}[\mathrm{hC3bC3bBbC5b6}][\mathrm{C7}]+k^{-}_{\mathrm{C5b*}}[\mathrm{hC3bC3bBbC5b}]-k^{+}_{\mathrm{C3bH}}[\mathrm{hC3bC3bBb}][\mathrm{H}]+k^{-}_{\mathrm{C3bH}}[\mathrm{hC3bC3bBbH}]$$

$$-k^{+}_{\mathrm{C3bHL}}[\mathrm{hC3bC3bBb}][\mathrm{HL}]+k^{-}_{\mathrm{C3bHL}}[\mathrm{hC3bC3bBbHL}]-k^{+}_{\mathrm{C3bC3bCR1}}[\mathrm{hC3bC3bBb}][\mathrm{CR1}]+k^{-}_{\mathrm{C3bC3bCR1}}[\mathrm{hC3bC3bBbCR1}]$$

$$-k^{+}_{\mathrm{C3bBbDAF}}[\mathrm{hC3bC3bBb}][\mathrm{DAF}]+k^{-}_{\mathrm{C3bBbDAF}}[\mathrm{hC3bC3bBbDAF}]$$

S64

$$\frac{d[\mathrm{hC3bC3bP}]}{dt}=k^{+}_{\mathrm{C3bP}}[\mathrm{hC3bC3b}][\mathrm{P}]-k^{-}_{\mathrm{C3bP}}[\mathrm{hC3bC3bP}]-k^{+}_{\mathrm{C3bB}}[\mathrm{hC3bC3bP}][\mathrm{B}]+k^{-}_{\mathrm{C3bB}}[\mathrm{hC3bC3bPB}]$$

S65

$$\frac{d[\mathrm{hC3bC3bBP}]}{dt}=k^{+}_{\mathrm{C3bB}}[\mathrm{hC3bC3bP}][\mathrm{B}]-k^{-}_{\mathrm{C3bB}}[\mathrm{hC3bC3bBP}]$$

$$-\left(\frac{k^{\mathrm{D}}_{\mathrm{catC3(H_2O)B}}[\mathrm{D}][\mathrm{hC3bC3bBP}]}{K^{\mathrm{D}}_{\mathrm{mC3(H_2O)B}}+[\mathrm{C3(H_2O)B}]+[\mathrm{fC3bB}]+[\mathrm{fC3bC3bB}]+[\mathrm{fC3bC3bBP}]+[\mathrm{IgGC3bC3bB}]+[\mathrm{IgGC3bC3bBP}]+[\mathrm{fC3bC4bB}]+[\mathrm{fC3bC4bBP}]}\cdots\right.$$
$$\left.\frac{1}{+[\mathrm{hC3bB}]+[\mathrm{hC3bC3bB}]+[\mathrm{hC3bC3bBP}]+[\mathrm{hC3bC4bB}]+[\mathrm{hC3bC4bBP}]}\right)$$

S66

$$\frac{d[\text{hC3bC3bBbP}]}{dt}=$$

$$\left(\frac{k^{D}_{\text{catC3(H}_2\text{O)B}}[\text{D}][\text{hC3bC3bBP}]}{K^{D}_{\text{mC3(H}_2\text{O)B}}+[\text{C3(H}_2\text{O)B}]+[\text{fC3bB}]+[\text{fC3bC3bB}]+[\text{fC3bC3bBP}]+[\text{IgGC3bC3bB}]+[\text{IgGC3bC3bBP}]+[\text{fC3bC4bB}]+[\text{fC3bC4bBP}]}\cdots \right.$$
$$\left.\frac{1}{+[\text{hC3bB}]+[\text{hC3bC3bB}]+[\text{hC3bC3bBP}]+[\text{hC3bC4bB}]+[\text{hC3bC4bBP}]}\right)$$

$$+k^{+}_{\text{C3bC3b}}[\text{hC3bBbP}][\text{nfC3b}]+k^{+}_{\text{C3bP}}[\text{hC3bC3bBb}][\text{P}]-k^{-}_{\text{C3bP}}[\text{hC3bC3bBbP}]-k^{-}_{\text{C3bC3bBbP}}[\text{hC3bC3bBbP}]+k^{+}_{\text{C5b7}}[\text{hC3bC3bBbPC5b6}][\text{C7}]$$
$$+k^{-}_{\text{C5b*}}[\text{hC3bC3bBbPC5b}]$$

S67

$$\frac{d[\text{hC4b}]}{dt}=k^{+}_{\text{hC4b}}[\text{Surface}_{\text{host}}][\text{nfC4b}]-k^{+}_{\text{C4bC2}}[\text{hC4b}][\text{C2}]+k^{-}_{\text{C4bC2}}[\text{hC4bC2}]-k^{+}_{\text{C4bC4b}}[\text{hC4b}][\text{nfC4b}]-k^{+}_{\text{C3bC4b}}[\text{hC4b}][\text{nfC3b}]$$
$$-k^{+}_{\text{C4bC4BP}}[\text{hC4b}][\text{C4BP}]+k^{-}_{\text{C4bC4BP}}[\text{hC4bC4BP}]+k^{+}_{\text{C4bCR1}}[\text{hC4b}][\text{CR1}]+k^{-}_{\text{C4bCR1}}[\text{hC4bCR1}]$$

S68

$$\frac{d[\text{hC4bC2}]}{dt}=k^{+}_{\text{C4bC2}}[\text{hC4b}][\text{C2}]-k^{-}_{\text{C4bC2}}[\text{hC4bC2}]$$

S69

$$\frac{d[\text{hC4bC4b}]}{dt}=k^{+}_{\text{C4bC4b}}[\text{hC4b}][\text{nfC4b}]-k^{+}_{\text{C4bC2}}[\text{hC4bC4b}][\text{C2}]+k^{-}_{\text{C4bC2}}[\text{hC4bC4bC2}]-k^{+}_{\text{C4bC4BP}}[\text{hC4bC4b}][\text{C4BP}]$$
$$+k^{-}_{\text{C4bC4BP}}[\text{hC4bC4bC4BP}]-k^{+}_{\text{C4bC4bCR1}}[\text{hC4bC4b}][\text{CR1}]+k^{-}_{\text{C4bC4bCR1}}[\text{hC4bC4bCR1}]$$

S70

$$\frac{d[\text{hC4bC4bC2}]}{dt}=k^{+}_{\text{C4bC2}}[\text{hC4bC4b}][\text{C2}]-k^{-}_{\text{C4bC2}}[\text{hC4bC4bC2}]$$

S71

$$\frac{d[\text{hC3bC4b}]}{dt}=k^{+}_{\text{C3bC4b}}[\text{hC4b}][\text{nfC3b}]+k^{+}_{\text{C3bC4b}}[\text{hC3b}][\text{nfC4b}]-k^{+}_{\text{C4bC2}}[\text{hC3bC4b}][\text{C2}]+k^{-}_{\text{C4bC2}}[\text{hC3bC4bC2}]-k^{+}_{\text{C3bB}}[\text{hC3bC4b}][\text{B}]$$
$$+k^{-}_{\text{C3bB}}[\text{hC3bC4bB}]-k^{+}_{\text{C3bP}}[\text{hC3bC4b}][\text{P}]+k^{-}_{\text{C3bP}}[\text{hC3bC4bP}]+k^{-}_{\text{C3bC4bBb}}[\text{hC3bC4bBb}]+k^{-}_{\text{C3bC4bBbP}}[\text{hC3bC4bBbP}]$$
$$-k^{+}_{\text{C3bH}}[\text{hC3bC4b}][\text{H}]+k^{-}_{\text{C3bH}}[\text{hC3bC4bH}]+k^{-}_{\text{C3bBbH}_{\text{decay}}}[\text{hC3bC4bBbH}]-k^{+}_{\text{C3bHL}}[\text{hC3bC4b}][\text{HL}]+k^{-}_{\text{C3bHL}}[\text{hC3bC4bHL}]$$
$$+k^{-}_{\text{C3bBbHL}_{\text{decay}}}[\text{hC3bC4bBbHL}]-k^{+}_{\text{C3bC4bCR1}}[\text{hC3bC4b}][\text{CR1}]+k^{-}_{\text{C3bC4bCR1}}[\text{hC3bC4bCR1}]+k^{-}_{\text{C3bBbCR1}_{\text{decay}}}[\text{hC3bC4bBbCR1}]$$
$$-k^{+}_{\text{C4bC4BP}}[\text{hC3bC4b}][\text{C4BP}]+k^{-}_{\text{C4bC4BP}}[\text{hC3bC4bC4BP}]+k^{-}_{\text{C3bBbDAF}_{\text{decay}}}[\text{hC3bC4bBbDAF}]$$

S72

$$\frac{d[\text{hC3bC4bC2}]}{dt}=k^{+}_{\text{C4bC2}}[\text{hC3bC4b}][\text{C2}]-k^{-}_{\text{C4bC2}}[\text{hC3bC4bC2}]$$

S73

$$\frac{d[\mathrm{hC3bC4bB}]}{dt} = k^{+}_{\mathrm{C3bB}}[\mathrm{hC3bC4b}][\mathrm{B}] - k^{-}_{\mathrm{C3bB}}[\mathrm{hC3bC4bB}]$$

$$-\left(\frac{k^{\mathrm{D}}_{\mathrm{catC3(H_2O)B}}[\mathrm{D}][\mathrm{hC3bC4bB}]}{K^{\mathrm{D}}_{\mathrm{mC3(H_2O)B}} + [\mathrm{C3(H_2O)B}] + [\mathrm{fC3bB}] + [\mathrm{fC3bC3bB}] + [\mathrm{fC3bC3bBP}] + [\mathrm{IgGC3bC3bB}] + [\mathrm{IgGC3bC3bBP}] + [\mathrm{fC3bC4bB}] + [\mathrm{fC3bC4bBP}]} \cdots \frac{1}{+[\mathrm{hC3bB}] + [\mathrm{hC3bC3bB}] + [\mathrm{hC3bC3bBP}] + [\mathrm{hC3bC4bB}] + [\mathrm{hC3bC4bBP}]}\right)$$

S74

$$\frac{d[\mathrm{hC3bC4bBb}]}{dt} =$$

$$\left(\frac{k^{\mathrm{D}}_{\mathrm{catC3(H_2O)B}}[\mathrm{D}][\mathrm{hC3bC4bB}]}{K^{\mathrm{D}}_{\mathrm{mC3(H_2O)B}} + [\mathrm{C3(H_2O)B}] + [\mathrm{fC3bB}] + [\mathrm{fC3bC3bB}] + [\mathrm{fC3bC3bBP}] + [\mathrm{IgGC3bC3bB}] + [\mathrm{IgGC3bC3bBP}] + [\mathrm{fC3bC4bB}] + [\mathrm{fC3bC4bBP}]} \cdots \frac{1}{+[\mathrm{hC3bB}] + [\mathrm{hC3bC3bB}] + [\mathrm{hC3bC3bBP}] + [\mathrm{hC3bC4bB}] + [\mathrm{hC3bC4bBP}]}\right)$$

$$- k^{+}_{\mathrm{C3bP}}[\mathrm{hC3bC4bBb}][\mathrm{P}] + k^{-}_{\mathrm{C3bP}}[\mathrm{hC3bC4bBbP}] + k^{+}_{\mathrm{C3bC4b}}[\mathrm{hC3bBb}][\mathrm{nfC4b}] - k^{-}_{\mathrm{C3bC4bBb}}[\mathrm{hC3bC4bBb}] + k^{+}_{\mathrm{C5b7}}[\mathrm{hC3bC4bBbC5b6}][\mathrm{C7}]$$

$$+ k^{-}_{\mathrm{C5b*}}[\mathrm{hC3bC4bBbC5b}] - k^{+}_{\mathrm{C3bH}}[\mathrm{hC3bC4bBb}][\mathrm{H}] + k^{-}_{\mathrm{C3bH}}[\mathrm{hC3bC4bBbH}] - k^{+}_{\mathrm{C3bHL}}[\mathrm{hC3bC4bBb}][\mathrm{HL}] + k^{-}_{\mathrm{C3bHL}}[\mathrm{hC3bC4bBbHL}]$$

$$- k^{+}_{\mathrm{C3bC4bCR1}}[\mathrm{hC3bC4bBb}][\mathrm{CR1}] + k^{-}_{\mathrm{C3bC4bCR1}}[\mathrm{hC3bC4bBbCR1}] - k^{+}_{\mathrm{C3bBbDAF}}[\mathrm{hC3bC4bBb}][\mathrm{DAF}] + k^{-}_{\mathrm{C3bBbDAF}}[\mathrm{hC3bC4bBbDAF}]$$

S75

$$\frac{d[\mathrm{hC3bC4bP}]}{dt} = k^{+}_{\mathrm{C3bP}}[\mathrm{hC3bC4b}][\mathrm{P}] - k^{-}_{\mathrm{C3bP}}[\mathrm{hC3bC4bP}] - k^{+}_{\mathrm{C3bB}}[\mathrm{hC3bC4bP}][\mathrm{B}] + k^{-}_{\mathrm{C3bB}}[\mathrm{hC3bC4bPB}]$$

S76

$$\frac{d[\mathrm{hC3bC4bBP}]}{dt} = k^{+}_{\mathrm{C3bB}}[\mathrm{hC3bC4bP}][\mathrm{B}] - k^{-}_{\mathrm{C3bB}}[\mathrm{hC3bC4bBP}]$$

$$-\left(\frac{k^{\mathrm{D}}_{\mathrm{catC3(H_2O)B}}[\mathrm{D}][\mathrm{hC3bC4bBP}]}{K^{\mathrm{D}}_{\mathrm{mC3(H_2O)B}} + [\mathrm{C3(H_2O)B}] + [\mathrm{fC3bB}] + [\mathrm{fC3bC3bB}] + [\mathrm{fC3bC3bBP}] + [\mathrm{IgGC3bC3bB}] + [\mathrm{IgGC3bC3bBP}] + [\mathrm{fC3bC4bB}] + [\mathrm{fC3bC4bBP}]} \cdots \frac{1}{+[\mathrm{hC3bB}] + [\mathrm{hC3bC3bB}] + [\mathrm{hC3bC3bBP}] + [\mathrm{hC3bC4bB}] + [\mathrm{hC3bC4bBP}]}\right)$$

S77

$$\frac{d[\mathrm{hC3bC4bBbP}]}{dt} =$$

$$\left(\frac{k^{\mathrm{D}}_{\mathrm{catC3(H_2O)B}}[\mathrm{D}][\mathrm{hC3bC4bBP}]}{K^{\mathrm{D}}_{\mathrm{mC3(H_2O)B}} + [\mathrm{C3(H_2O)B}] + [\mathrm{fC3bB}] + [\mathrm{fC3bC3bB}] + [\mathrm{fC3bC3bBP}] + [\mathrm{IgGC3bC3bB}] + [\mathrm{IgGC3bC3bBP}] + [\mathrm{fC3bC4bB}] + [\mathrm{fC3bC4bBP}]} \cdots \frac{1}{+[\mathrm{hC3bB}] + [\mathrm{hC3bC3bB}] + [\mathrm{hC3bC3bBP}] + [\mathrm{hC3bC4bB}] + [\mathrm{hC3bC4bBP}]}\right)$$

$$+ k^{+}_{\mathrm{C3bP}}[\mathrm{hC3bC4bBb}][\mathrm{P}] - k^{-}_{\mathrm{C3bP}}[\mathrm{hC3bC4bBbP}] - k^{-}_{\mathrm{C3bC4bBbP}}[\mathrm{hC3bC4bBbP}] + k^{+}_{\mathrm{C5b7}}[\mathrm{hC3bC4bBbPC5b6}][\mathrm{C7}]$$

$$+ k^{-}_{\mathrm{C5b*}}[\mathrm{fC3bC4bBbPC5b}]$$

(iii) Termination (host cell and fluid state)

S78

$$\frac{d[\text{hC3bC4bBbC5b}]}{dt} = \frac{k_{\text{catC3bC4bBb}}^{\text{C5}}[\text{C5}][\text{hC3bC4bBb}]}{K_{\text{mC3bC4bBb}}^{\text{C5}}\left(1+\frac{[\text{C3}]}{K_{\text{mC3bBb}}^{\text{C3}}}+\frac{[\text{C5}]}{K_{\text{mC3bC4bBb}}^{\text{C5}}}\right)} - k_{\text{C5b*}}^{-}[\text{hC3bC4bBbC5b}] - k_{\text{C5b6}}^{+}[\text{hC3bC4bBbC5b}][\text{C6}] + k_{\text{C5b6}}^{-}[\text{hC3bC4bBbC5b6}]$$

S79

$$\frac{d[\text{hC3bC4bBbC5b6}]}{dt} = k_{\text{C5b6}}^{+}[\text{hC3bC4bBbC5b}][\text{C6}] - k_{\text{C5b6}}^{-}[\text{hC3bC4bBbC5b6}] - k_{\text{C5b7}}^{+}[\text{hC3bC4bBbC5b6}][\text{C7}]$$

S80

$$\frac{d[\text{hC3bC4bBbPC5b}]}{dt} = \frac{k_{\text{catC3bC4bBb}}^{\text{C5}}[\text{C5}][\text{hC3bC4bBbP}]}{K_{\text{mC3bC4bBb}}^{\text{C5}}\left(1+\frac{[\text{C3}]}{K_{\text{mC3bBb}}^{\text{C3}}}+\frac{[\text{C5}]}{K_{\text{mC3bC4bBb}}^{\text{C5}}}\right)} - k_{\text{C5b*}}^{-}[\text{hC3bC4bBbC5bP}] - k_{\text{C5b6}}^{+}[\text{hC3bC4bBbC5bP}][\text{C6}] + k_{\text{C5b6}}^{-}[\text{hC3bC4bBbC5b6P}]$$

S81

$$\frac{d[\text{hC3bC4bBbPC5b6}]}{dt} = k_{\text{C5b6}}^{+}[\text{hC3bC4bBbPC5b}][\text{C6}] - k_{\text{C5b6}}^{-}[\text{hC3bC4bBbPC5b6}] - k_{\text{C5b7}}^{+}[\text{hC3bC4bBbPC5b6}][\text{C7}]$$

S82

$$\frac{d[\text{hC3bC3bBbC5b}]}{dt} = \frac{k_{\text{catC3bC3bBb}}^{\text{C5}}[\text{C5}][\text{hC3bC3bBb}]}{K_{\text{mC3bC3bBb}}^{\text{C5}}\left(1+\frac{[\text{C3}]}{K_{\text{mC3bBb}}^{\text{C3}}}+\frac{[\text{C5}]}{K_{\text{mC3bC3bBb}}^{\text{C5}}}\right)} - k_{\text{C5b*}}^{-}[\text{hC3bC3bBbC5b}] - k_{\text{C5b6}}^{+}[\text{hC3bC3bBbC5b}][\text{C6}] + k_{\text{C5b6}}^{-}[\text{hC3bC3bBbC5b6}]$$

S83

$$\frac{d[\text{hC3bC3bBbC5b6}]}{dt} = k_{\text{C5b6}}^{+}[\text{hC3bC3bBbC5b}][\text{C6}] - k_{\text{C5b6}}^{-}[\text{hC3bC3bBbC5b6}] - k_{\text{C5b7}}^{+}[\text{hC3bC3bBbC5b6}][\text{C7}]$$

S84

$$\frac{d[\text{hC3bC3bBbPC5b}]}{dt} = \frac{k_{\text{catC3bC3bBb}}^{\text{C5}}[\text{C5}][\text{hC3bC3bBbP}]}{K_{\text{mC3bC3bBb}}^{\text{C5}}\left(1+\frac{[\text{C3}]}{K_{\text{mC3bBb}}^{\text{C3}}}+\frac{[\text{C5}]}{K_{\text{mC3bC3bBb}}^{\text{C5}}}\right)} - k_{\text{C5b*}}^{-}[\text{hC3bC3bBbPC5b}] - k_{\text{C5b6}}^{+}[\text{hC3bC3bBbPC5b}][\text{C6}] + k_{\text{C5b6}}^{-}[\text{hC3bC3bBbPC5b6}]$$

S85

$$\frac{d[\text{hC3bC3bBbPC5b6}]}{dt}=k^{+}_{\text{C5b6}}[\text{hC3bC3bBbPC5b}][\text{C6}]-k^{-}_{\text{C5b6}}[\text{hC3bC3bBbPC5b6}]-k^{+}_{\text{C5b7}}[\text{hC3bC3bBbPC5b6}][\text{C7}]$$

S86

$$\frac{d[\text{fC5b7}]}{dt}=k^{+}_{\text{C5b7}}[\text{fC5b6}][\text{C7}]-k^{-}_{\text{C5b7}}[\text{fC5b7}]+k^{+}_{\text{C5b7}}[\text{fC3bC3bBbC5b6}][\text{C7}]+k^{+}_{\text{C5b7}}[\text{fC3bC3bBbPC5b6}][\text{C7}]$$
$$+k^{+}_{\text{C5b7}}[\text{fC3bC4bBbC5b6}][\text{C7}]+k^{+}_{\text{C5b7}}[\text{fC3bC4bBbPC5b6}][\text{C7}]+k^{+}_{\text{C5b7}}[\text{fC3bC4bC2aC5b6}][\text{C7}]$$
$$+k^{+}_{\text{C5b7}}[\text{fC4bC4bC2aC5b6}][\text{C7}]-k^{+}_{\text{C5b8}}[\text{fC5b7}][\text{C8}]+k^{-}_{\text{C5b8}}[\text{fC5b8}]-k^{+}_{\text{C5b7}_{\text{micelle}}}[\text{fC5b7}]-k^{+}_{\text{VnC5b7}}[\text{fC5b7}][\text{Vn}]$$
$$+k^{-}_{\text{VnC5b7}}[\text{VnC5b7}]-k^{+}_{\text{CnC5b7}}[\text{fC5b7}][\text{Cn}]+k^{-}_{\text{CnC5b7}}[\text{CnC5b7}]-k^{+}_{\text{hC5b7}}[\text{Surface}_{\text{host}}][\text{fC5b7}]+k^{+}_{\text{C5b7}}[\text{hC3bC4bBbC5b6}][\text{C7}]$$
$$+k^{+}_{\text{C5b7}}[\text{hC3bC4bBbPC5b6}][\text{C7}]+k^{+}_{\text{C5b7}}[\text{hC3bC3bBbC5b6}][\text{C7}]+k^{+}_{\text{C5b7}}[\text{hC3bC3bBbPC5b6}][\text{C7}]$$

S87

$$\frac{d[\text{fC5b*}_{\text{inactive}}]}{dt}=k^{-}_{\text{C5b*}}[\text{fC5b*}]+k^{-}_{\text{C5b*}}[\text{fC3bC3bBbC5b}]+k^{-}_{\text{C5b*}}[\text{fC3bC3bBbPC5b}]+k^{-}_{\text{C5b*}}[\text{fC3bC4bBbC5b}]+k^{-}_{\text{C5b*}}[\text{fC3bC4bBbPC5b}]$$
$$+k^{-}_{\text{C5b*}}[\text{fC4bC4bC2aC5b}]+k^{-}_{\text{C5b*}}[\text{fC3bC4bC2aC5b}]+k^{-}_{\text{C5b*}}[\text{hC3bC3bBbC5b}]+k^{-}_{\text{C5b*}}[\text{hC3bC3bBbPC5b}]+k^{-}_{\text{C5b*}}[\text{hC3bC4bBbC5b}]$$
$$+k^{-}_{\text{C5b*}}[\text{hC3bC4bBbPC5b}]$$

S88

$$\frac{d[\text{fC5b7}_{\text{micelle}}]}{dt}=k^{+}_{\text{C5b7}_{\text{micelle}}}[\text{fC5b7}]$$

S89

$$\frac{d[\text{fC5b8}]}{dt}=k^{+}_{\text{C5b8}}[\text{fC5b7}][\text{C8}]-k^{-}_{\text{C5b8}}[\text{fC5b8}]-k^{+}_{\text{C5b9}}[\text{fC5b8}][\text{C9}_1]+k^{-}_{\text{C5b9}}[\text{fC5b9}_1]-k^{+}_{\text{VnC5b8}}[\text{fC5b8}][\text{Vn}]+k^{-}_{\text{VnC5b8}}[\text{VnC5b8}]$$
$$-k^{+}_{\text{CnC5b8}}[\text{fC5b8}][\text{Cn}]+k^{-}_{\text{CnC5b8}}[\text{CnC5b8}]$$

S90

$$\frac{d[\text{fC5b9}_1]}{dt}=k^{+}_{\text{C5b9}}[\text{fC5b8}][\text{C9}_1]-k^{-}_{\text{C5b9}}[\text{fC5b9}_1]-k^{+}_{\text{VnC5b9}}[\text{fC5b9}][\text{Vn}]+k^{-}_{\text{VnC5b9}}[\text{VnC5b9}]-k^{+}_{\text{CnC5b9}}[\text{fC5b9}][\text{Cn}]$$
$$+k^{-}_{\text{CnC5b9}}[\text{CnC5b9}]$$

S91

$$\frac{d[\text{hC5b7}]}{dt}=k^{+}_{\text{hC5b7}}[\text{fC5b7}][\text{Host Surface}]-k^{+}_{\text{C5b8}}[\text{hC5b7}][\text{C8}]$$

S92

$$\frac{d[\text{hC5b8}]}{dt}=k^{+}_{\text{C5b8}}[\text{hC5b7}][\text{C8}]-k^{+}_{\text{C5b9}}[\text{hC5b8}][\text{C9}_1]-k^{+}_{\text{C5b8CD59}}[\text{hC5b8}][\text{CD59}]+k^{-}_{\text{C5b8CD59}}[\text{hC5b8CD59}]$$

S93

$$\frac{d[\text{hC5b9}_1]}{dt} = k^+_{\text{C5b9}}[\text{hC5b8}][\text{C9}_1] - k^+_{\text{C5b9}}[\text{hC5b9}_1][\text{C9}_2] - k^+_{\text{C5b9}_1\text{CD59}}[\text{hC5b9}_1][\text{CD59}] + k^-_{\text{C5b9}_1\text{CD59}}[\text{hC5b9}_1\text{CD59}]$$

S94

$$\frac{d[\text{hC5b9}_2]}{dt} = k^+_{\text{C5b9}}[\text{hC5b9}_1][\text{C9}_2] - k^+_{\text{C5b9}}[\text{hC5b9}_2][\text{C9}_3]$$

S95

$$\frac{d[\text{hC5b9}_3]}{dt} = k^+_{\text{C5b9}}[\text{hC5b9}_2][\text{C9}_3] - k^+_{\text{C5b9}}[\text{hC5b9}_3][\text{C9}_4]$$

S96

$$\frac{d[\text{hC5b9}_4]}{dt} = k^+_{\text{C5b9}}[\text{hC5b9}_3][\text{C9}_4] - k^+_{\text{C5b9}}[\text{hC5b9}_4][\text{C9}_5]$$

S97

$$\frac{d[\text{hC5b9}_5]}{dt} = k^+_{\text{C5b9}}[\text{hC5b9}_4][\text{C9}_5] - k^+_{\text{C5b9}}[\text{hC5b9}_5][\text{C9}_6]$$

S98

$$\frac{d[\text{hC5b9}_6]}{dt} = k^+_{\text{C5b9}}[\text{hC5b9}_5][\text{C9}_6] - k^+_{\text{C5b9}}[\text{hC5b9}_6][\text{C9}_7]$$

S99

$$\frac{d[\text{hC5b9}_7]}{dt} = k^+_{\text{C5b9}}[\text{hC5b9}_6][\text{C9}_7] - k^+_{\text{C5b9}}[\text{hC5b9}_7][\text{C9}_8]$$

S100

$$\frac{d[\text{hC5b9}_8]}{dt} = k^+_{\text{C5b9}}[\text{hC5b9}_7][\text{C9}_8] - k^+_{\text{C5b9}}[\text{hC5b9}_8][\text{C9}_9]$$

S101

$$\frac{d[\text{hC5b9}_9]}{dt} = k^+_{\text{C5b9}}[\text{hC5b9}_8][\text{C9}_9] - k^+_{\text{C5b9}}[\text{hC5b9}_9][\text{C9}_{10}]$$

S102

$$\frac{d[\text{hC5b9}_{10}]}{dt} = k^+_{\text{C5b9}}[\text{hC5b9}_9][\text{C9}_{10}] - k^+_{\text{C5b9}}[\text{hC5b9}_{10}][\text{C9}_{11}]$$

S103

$$\frac{d[\text{hC5b9}_{11}]}{dt} = k^+_{\text{C5b9}}[\text{hC5b9}_{10}][\text{C9}_{11}] - k^+_{\text{C5b9}}[\text{hC5b9}_{11}][\text{C9}_{12}]$$

S104

$$\frac{d[\mathrm{hC5b9}_{12}]}{dt} = k^{+}_{\mathrm{C5b9}}[\mathrm{hC5b9}_{11}][\mathrm{C9}_{12}] - k^{+}_{\mathrm{C5b9}}[\mathrm{hC5b9}_{12}][\mathrm{C9}_{13}]$$

S105

$$\frac{d[\mathrm{hC5b9}_{13}]}{dt} = k^{+}_{\mathrm{C5b9}}[\mathrm{hC5b9}_{12}][\mathrm{C9}_{13}] - k^{+}_{\mathrm{C5b9}}[\mathrm{hC5b9}_{13}][\mathrm{C9}_{14}]$$

S106

$$\frac{d[\mathrm{hC5b9}_{14}]}{dt} = k^{+}_{\mathrm{C5b9}}[\mathrm{hC5b9}_{13}][\mathrm{C9}_{14}] - k^{+}_{\mathrm{C5b9}}[\mathrm{hC5b9}_{14}][\mathrm{C9}_{15}]$$

S107

$$\frac{d[\mathrm{hC5b9}_{15}]}{dt} = k^{+}_{\mathrm{C5b9}}[\mathrm{hC5b9}_{14}][\mathrm{C9}_{15}] - k^{+}_{\mathrm{C5b9}}[\mathrm{hC5b9}_{15}][\mathrm{C9}_{16}]$$

S108

$$\frac{d[\mathrm{hC5b9}_{16}]}{dt} = k^{+}_{\mathrm{C5b9}}[\mathrm{hC5b9}_{15}][\mathrm{C9}_{16}] - k^{+}_{\mathrm{C5b9}}[\mathrm{hC5b9}_{16}][\mathrm{C9}_{17}]$$

S109

$$\frac{d[\mathrm{hC5b9}_{17}]}{dt} = k^{+}_{\mathrm{C5b9}}[\mathrm{hC5b9}_{16}][\mathrm{C9}_{17}] - k^{+}_{\mathrm{C5b9}}[\mathrm{hC5b9}_{17}][\mathrm{C9}_{18}]$$

S110

$$\frac{d[\mathrm{hC5b9}_{18}]}{dt} = k^{+}_{\mathrm{C5b9}}[\mathrm{hC5b9}_{17}][\mathrm{C9}_{18}]$$

(iv) Regulation (host cell and fluid state)

S111

$$\frac{d[\mathrm{C3(H_2O)H}]}{dt} = k^{+}_{\mathrm{C3bH}}[\mathrm{C3(H_2O)}][\mathrm{H}] - k^{-}_{\mathrm{C3bH}}[\mathrm{C3(H_2O)H}]$$

$$- \left(\frac{k^{\mathrm{FI}}_{\mathrm{catC3bH}}[\mathrm{I}][\mathrm{C3(H_2O)H}]}{K^{\mathrm{FI}}_{\mathrm{mC3bH}} + [\mathrm{C3(H_2O)H}] + [\mathrm{C3(H_2O)HL}] + [\mathrm{fC3bH}] + [\mathrm{fC3bHL}] + [\mathrm{fC3bCR1}] + [\mathrm{fiC3bCR1}] + [\mathrm{IgGC3bC3bH}] + [\mathrm{IgGC3biC3bH}] + [\mathrm{IgGC3bC3bHL}]} \cdots \right.$$
$$\frac{1}{+ [\mathrm{IgGC3biC3bHL}] + [\mathrm{IgGC3bC3bCR1}] + [\mathrm{IgGC3biC3bCR1}] + [\mathrm{IgGiC3biC3bCR1}] + [\mathrm{IgGiC3bC3dgCR1}] + [\mathrm{fC3bC4bH}] + [\mathrm{fC3bC4bHL}]} \cdots$$
$$\frac{1}{+ [\mathrm{fC3bC4bCR1}] + [\mathrm{fC3bC4bC4BP}] + [\mathrm{fC3bC4dH}] + [\mathrm{fC3bC4dHL}] + [\mathrm{fC3bC4dCR1}] + [\mathrm{fiC3bC4dCR1}] + [\mathrm{fiC3bC4bCR1}] + [\mathrm{fiC3bC4bC4BP}]} \cdots$$
$$\frac{1}{+ [\mathrm{fC3dgC4bCR1}] + [\mathrm{fC3dgC4bC4BP}] + [\mathrm{fC3bC3bH}] + [\mathrm{fC3bC3bHL}] + [\mathrm{fC3bC3bCR1}] + [\mathrm{fC3biC3bH}] + [\mathrm{fC3biC3bHL}] + [\mathrm{fC3biC3bCR1}]} \cdots$$
$$\frac{1}{+ [\mathrm{fiC3biC3bCR1}] + [\mathrm{fiC3bC3dgCR1}] + [\mathrm{hC3bH}] + [\mathrm{hC3bHL}] + [\mathrm{hC3bCR1}] + [\mathrm{hiC3bCR1}] + [\mathrm{hC3bC4bH}] + [\mathrm{hC3bC4bHL}] + [\mathrm{hC3bC4bCR1}]} \cdots$$
$$\frac{1}{+ [\mathrm{hC3bC4bC4BP}] + [\mathrm{hC3bC4dH}] + [\mathrm{hC3bC4dHL}] + [\mathrm{hC3bC4dCR1}] + [\mathrm{hiC3bC4dCR1}] + [\mathrm{hiC3bC4bCR1}] + [\mathrm{hiC3bC4bC4BP}] + [\mathrm{hC3dgC4bCR1}]} \cdots$$
$$\frac{1}{+ [\mathrm{hC3dgC4bC4BP}] + [\mathrm{hC3bC3bH}] + [\mathrm{hC3bC3bHL}] + [\mathrm{hC3bC3bCR1}] + [\mathrm{hC3biC3bH}] + [\mathrm{hC3biC3bHL}] + [\mathrm{hC3biC3bCR1}] + [\mathrm{hiC3biC3bCR1}]} \cdots$$
$$\frac{1}{+ [\mathrm{hiC3bC3dgCR1}] + [\mathrm{fC4bCR1}] + [\mathrm{nfC4bC4BP}] + [\mathrm{fC4bC4BP}] + [\mathrm{fC4bC4bCR1}] + [\mathrm{fC4bC4bC4BP}] + [\mathrm{fC4bC4dCR1}] + [\mathrm{fC4bC4dC4BP}] + [\mathrm{hC4bCR1}]} \cdots$$
$$\frac{1}{+ [\mathrm{hC4bC4BP}] + [\mathrm{hC4bC4bCR1}] + [\mathrm{hC4bC4bC4BP}] + [\mathrm{hC4bC4dCR1}] + [\mathrm{hC4bC4dC4BP}]} \cdots$$

S112

$$\frac{d[\mathrm{C3(H_2O)HL}]}{dt} = k^{+}_{\mathrm{C3bHL}}[\mathrm{C3(H_2O)}][\mathrm{HL}] - k^{-}_{\mathrm{C3bHL}}[\mathrm{C3(H_2O)HL}]$$

$$- \left(\frac{k^{\mathrm{FI}}_{\mathrm{catC3bH}}[\mathrm{I}][\mathrm{C3(H_2O)HL}]}{K^{\mathrm{FI}}_{\mathrm{mC3bH}} + [\mathrm{C3(H_2O)H}] + [\mathrm{C3(H_2O)HL}] + [\mathrm{fC3bH}] + [\mathrm{fC3bHL}] + [\mathrm{fC3bCR1}] + [\mathrm{fiC3bCR1}] + [\mathrm{IgGC3bC3bH}] + [\mathrm{IgGC3biC3bH}] + [\mathrm{IgGC3bC3bHL}]} \cdots \right.$$
$$\frac{1}{+ [\mathrm{IgGC3biC3bHL}] + [\mathrm{IgGC3bC3bCR1}] + [\mathrm{IgGC3biC3bCR1}] + [\mathrm{IgGiC3biC3bCR1}] + [\mathrm{IgGiC3bC3dgCR1}] + [\mathrm{fC3bC4bH}] + [\mathrm{fC3bC4bHL}]} \cdots$$
$$\frac{1}{+ [\mathrm{fC3bC4bCR1}] + [\mathrm{fC3bC4bC4BP}] + [\mathrm{fC3bC4dH}] + [\mathrm{fC3bC4dHL}] + [\mathrm{fC3bC4dCR1}] + [\mathrm{fiC3bC4dCR1}] + [\mathrm{fiC3bC4bCR1}] + [\mathrm{fiC3bC4bC4BP}]} \cdots$$
$$\frac{1}{+ [\mathrm{fC3dgC4bCR1}] + [\mathrm{fC3dgC4bC4BP}] + [\mathrm{fC3bC3bH}] + [\mathrm{fC3bC3bHL}] + [\mathrm{fC3bC3bCR1}] + [\mathrm{fC3biC3bH}] + [\mathrm{fC3biC3bHL}] + [\mathrm{fC3biC3bCR1}]} \cdots$$
$$\frac{1}{+ [\mathrm{fiC3biC3bCR1}] + [\mathrm{fiC3bC3dgCR1}] + [\mathrm{hC3bH}] + [\mathrm{hC3bHL}] + [\mathrm{hC3bCR1}] + [\mathrm{hiC3bCR1}] + [\mathrm{hC3bC4bH}] + [\mathrm{hC3bC4bHL}] + [\mathrm{hC3bC4bCR1}]} \cdots$$
$$\frac{1}{+ [\mathrm{hC3bC4bC4BP}] + [\mathrm{hC3bC4dH}] + [\mathrm{hC3bC4dHL}] + [\mathrm{hC3bC4dCR1}] + [\mathrm{hiC3bC4dCR1}] + [\mathrm{hiC3bC4bCR1}] + [\mathrm{hiC3bC4bC4BP}] + [\mathrm{hC3dgC4bCR1}]} \cdots$$
$$\frac{1}{+ [\mathrm{hC3dgC4bC4BP}] + [\mathrm{hC3bC3bH}] + [\mathrm{hC3bC3bHL}] + [\mathrm{hC3bC3bCR1}] + [\mathrm{hC3biC3bH}] + [\mathrm{hC3biC3bHL}] + [\mathrm{hC3biC3bCR1}] + [\mathrm{hiC3biC3bCR1}]} \cdots$$
$$\frac{1}{+ [\mathrm{hiC3bC3dgCR1}] + [\mathrm{fC4bCR1}] + [\mathrm{nfC4bC4BP}] + [\mathrm{fC4bC4BP}] + [\mathrm{fC4bC4bCR1}] + [\mathrm{fC4bC4bC4BP}] + [\mathrm{fC4bC4dCR1}] + [\mathrm{fC4bC4dC4BP}] + [\mathrm{hC4bCR1}]} \cdots$$
$$\frac{1}{+ [\mathrm{hC4bC4BP}] + [\mathrm{hC4bC4bCR1}] + [\mathrm{hC4bC4bC4BP}] + [\mathrm{hC4bC4dCR1}] + [\mathrm{hC4bC4dC4BP}]} \cdots$$

S113

$$\frac{d[\mathrm{C3(H_2O)BbH}]}{dt} = k^{+}_{\mathrm{C3bH}}[\mathrm{C3(H_2O)Bb}][\mathrm{H}] - k^{-}_{\mathrm{C3bH}}[\mathrm{C3(H_2O)BbH}] - k^{-}_{\mathrm{C3bBbH}_{\mathrm{decay}}}[\mathrm{C3(H_2O)BbH}]$$

S114

$$\frac{d[\mathrm{C3(H_2O)BbHL}]}{dt} = k^{+}_{\mathrm{C3bHL}}[\mathrm{C3(H_2O)Bb}][\mathrm{HL}] - k^{-}_{\mathrm{C3bHL}}[\mathrm{C3(H_2O)BbHL}] - k^{-}_{\mathrm{C3bBbHL}_{\mathrm{decay}}}[\mathrm{C3(H_2O)BbHL}]$$

S115

$$\frac{d[\mathrm{iC3(H_2O)}]}{dt} =$$

$$\left(\frac{k^{\mathrm{FI}}_{\mathrm{catC3bH}}[\mathrm{I}][\mathrm{C3(H_2O)H}]}{K^{\mathrm{FI}}_{\mathrm{mC3bH}} + [\mathrm{C3(H_2O)H}] + [\mathrm{C3(H_2O)HL}] + [\mathrm{fC3bH}] + [\mathrm{fC3bHL}] + [\mathrm{fC3bCR1}] + [\mathrm{fiC3bCR1}] + [\mathrm{IgGC3bC3bH}] + [\mathrm{IgGC3biC3bH}] + [\mathrm{IgGC3bC3bHL}]} \cdots \right.$$
$$\frac{1}{+[\mathrm{IgGC3biC3bHL}] + [\mathrm{IgGC3bC3bCR1}] + [\mathrm{IgGC3biC3bCR1}] + [\mathrm{IgGiC3biC3bCR1}] + [\mathrm{IgGiC3bC3dgCR1}] + [\mathrm{fC3bC4bH}] + [\mathrm{fC3bC4bHL}]} \cdots$$
$$\frac{1}{+[\mathrm{fC3bC4bCR1}] + [\mathrm{fC3bC4bC4BP}] + [\mathrm{fC3bC4dH}] + [\mathrm{fC3bC4dHL}] + [\mathrm{fC3bC4dCR1}] + [\mathrm{fiC3bC4dCR1}] + [\mathrm{fiC3bC4bCR1}] + [\mathrm{fiC3bC4bC4BP}]} \cdots$$
$$\frac{1}{+[\mathrm{fC3dgC4bCR1}] + [\mathrm{fC3dgC4bC4BP}] + [\mathrm{fC3bC3bH}] + [\mathrm{fC3bC3bHL}] + [\mathrm{fC3bC3bCR1}] + [\mathrm{fC3biC3bH}] + [\mathrm{fC3biC3bHL}] + [\mathrm{fC3biC3bCR1}]} \cdots$$
$$\frac{1}{+[\mathrm{fiC3biC3bCR1}] + [\mathrm{fiC3bC3dgCR1}] + [\mathrm{hC3bH}] + [\mathrm{hC3bHL}] + [\mathrm{hC3bCR1}] + [\mathrm{hiC3bCR1}] + [\mathrm{hC3bC4bH}] + [\mathrm{hC3bC4bHL}] + [\mathrm{hC3bC4bCR1}]} \cdots$$
$$\frac{1}{+[\mathrm{hC3bC4bC4BP}] + [\mathrm{hC3bC4dH}] + [\mathrm{hC3bC4dHL}] + [\mathrm{hC3bC4dCR1}] + [\mathrm{hiC3bC4dCR1}] + [\mathrm{hiC3bC4bCR1}] + [\mathrm{hiC3bC4bC4BP}] + [\mathrm{hC3dgC4bCR1}]} \cdots$$
$$\frac{1}{+[\mathrm{hC3dgC4bC4BP}] + [\mathrm{hC3bC3bH}] + [\mathrm{hC3bC3bHL}] + [\mathrm{hC3bC3bCR1}] + [\mathrm{hC3biC3bH}] + [\mathrm{hC3biC3bHL}] + [\mathrm{hC3biC3bCR1}] + [\mathrm{hiC3biC3bCR1}]} \cdots$$
$$\frac{1}{+[\mathrm{hiC3bC3dgCR1}] + [\mathrm{fC4bCR1}] + [\mathrm{nfC4bC4BP}] + [\mathrm{fC4bC4BP}] + [\mathrm{fC4bC4bCR1}] + [\mathrm{fC4bC4bC4BP}] + [\mathrm{fC4bC4dCR1}] + [\mathrm{fC4bC4dC4BP}] + [\mathrm{hC4bCR1}]} \cdots$$
$$\left. \frac{1}{+[\mathrm{hC4bC4BP}] + [\mathrm{hC4bC4bCR1}] + [\mathrm{hC4bC4bC4BP}] + [\mathrm{hC4bC4dCR1}] + [\mathrm{hC4bC4dC4BP}]} \cdots \right)$$

$$+ \left(\frac{k^{\mathrm{FI}}_{\mathrm{catC3bH}}[\mathrm{I}][\mathrm{C3(H_2O)HL}]}{K^{\mathrm{FI}}_{\mathrm{mC3bH}} + [\mathrm{C3(H_2O)H}] + [\mathrm{C3(H_2O)HL}] + [\mathrm{fC3bH}] + [\mathrm{fC3bHL}] + [\mathrm{fC3bCR1}] + [\mathrm{fiC3bCR1}] + [\mathrm{IgGC3bC3bH}] + [\mathrm{IgGC3biC3bH}] + [\mathrm{IgGC3bC3bHL}]} \cdots \right.$$
$$\frac{1}{+[\mathrm{IgGC3biC3bHL}] + [\mathrm{IgGC3bC3bCR1}] + [\mathrm{IgGC3biC3bCR1}] + [\mathrm{IgGiC3biC3bCR1}] + [\mathrm{IgGiC3bC3dgCR1}] + [\mathrm{fC3bC4bH}] + [\mathrm{fC3bC4bHL}]} \cdots$$
$$\frac{1}{+[\mathrm{fC3bC4bCR1}] + [\mathrm{fC3bC4bC4BP}] + [\mathrm{fC3bC4dH}] + [\mathrm{fC3bC4dHL}] + [\mathrm{fC3bC4dCR1}] + [\mathrm{fiC3bC4dCR1}] + [\mathrm{fiC3bC4bCR1}] + [\mathrm{fiC3bC4bC4BP}]} \cdots$$
$$\frac{1}{+[\mathrm{fC3dgC4bCR1}] + [\mathrm{fC3dgC4bC4BP}] + [\mathrm{fC3bC3bH}] + [\mathrm{fC3bC3bHL}] + [\mathrm{fC3bC3bCR1}] + [\mathrm{fC3biC3bH}] + [\mathrm{fC3biC3bHL}] + [\mathrm{fC3biC3bCR1}]} \cdots$$
$$\frac{1}{+[\mathrm{fiC3biC3bCR1}] + [\mathrm{fiC3bC3dgCR1}] + [\mathrm{hC3bH}] + [\mathrm{hC3bHL}] + [\mathrm{hC3bCR1}] + [\mathrm{hiC3bCR1}] + [\mathrm{hC3bC4bH}] + [\mathrm{hC3bC4bHL}] + [\mathrm{hC3bC4bCR1}]} \cdots$$
$$\frac{1}{+[\mathrm{hC3bC4bC4BP}] + [\mathrm{hC3bC4dH}] + [\mathrm{hC3bC4dHL}] + [\mathrm{hC3bC4dCR1}] + [\mathrm{hiC3bC4dCR1}] + [\mathrm{hiC3bC4bCR1}] + [\mathrm{hiC3bC4bC4BP}] + [\mathrm{hC3dgC4bCR1}]} \cdots$$
$$\frac{1}{+[\mathrm{hC3dgC4bC4BP}] + [\mathrm{hC3bC3bH}] + [\mathrm{hC3bC3bHL}] + [\mathrm{hC3bC3bCR1}] + [\mathrm{hC3biC3bH}] + [\mathrm{hC3biC3bHL}] + [\mathrm{hC3biC3bCR1}] + [\mathrm{hiC3biC3bCR1}]} \cdots$$
$$\frac{1}{+[\mathrm{hiC3bC3dgCR1}] + [\mathrm{fC4bCR1}] + [\mathrm{nfC4bC4BP}] + [\mathrm{fC4bC4BP}] + [\mathrm{fC4bC4bCR1}] + [\mathrm{fC4bC4bC4BP}] + [\mathrm{fC4bC4dCR1}] + [\mathrm{fC4bC4dC4BP}] + [\mathrm{hC4bCR1}]} \cdots$$
$$\left. \frac{1}{+[\mathrm{hC4bC4BP}] + [\mathrm{hC4bC4bCR1}] + [\mathrm{hC4bC4bC4BP}] + [\mathrm{hC4bC4dCR1}] + [\mathrm{hC4bC4dC4BP}]} \cdots \right)$$

S116

$$\frac{d[\mathrm{fC3bH}]}{dt} = k^{+}_{\mathrm{C3bH}}[\mathrm{fC3b}][\mathrm{H}] - k^{-}_{\mathrm{C3bH}}[\mathrm{fC3bH}]$$

$$-\left(\begin{array}{l} \dfrac{k^{\mathrm{FI}}_{\mathrm{catC3bH}}[\mathrm{I}][\mathrm{fC3bH}]}{K^{\mathrm{FI}}_{\mathrm{mC3bH}} + [\mathrm{C3(H_2O)H}] + [\mathrm{C3(H_2O)HL}] + [\mathrm{fC3bH}] + [\mathrm{fC3bHL}] + [\mathrm{fC3bCR1}] + [\mathrm{fiC3bCR1}] + [\mathrm{IgGC3bC3bH}] + [\mathrm{IgGC3biC3bH}] + [\mathrm{IgGC3bC3bHL}]} \cdots \\ \dfrac{1}{+ [\mathrm{IgGC3biC3bHL}] + [\mathrm{IgGC3bC3bCR1}] + [\mathrm{IgGC3biC3bCR1}] + [\mathrm{IgGiC3biC3bCR1}] + [\mathrm{IgGiC3bC3dgCR1}] + [\mathrm{fC3bC4bH}] + [\mathrm{fC3bC4bHL}]} \cdots \\ \dfrac{1}{+ [\mathrm{fC3bC4bCR1}] + [\mathrm{fC3bC4bC4BP}] + [\mathrm{fC3bC4dH}] + [\mathrm{fC3bC4dHL}] + [\mathrm{fC3bC4dCR1}] + [\mathrm{fiC3bC4dCR1}] + [\mathrm{fiC3bC4bCR1}] + [\mathrm{fiC3bC4bC4BP}]} \cdots \\ \dfrac{1}{+ [\mathrm{fC3dgC4bCR1}] + [\mathrm{fC3dgC4bC4BP}] + [\mathrm{fC3bC3bH}] + [\mathrm{fC3bC3bHL}] + [\mathrm{fC3bC3bCR1}] + [\mathrm{fC3biC3bH}] + [\mathrm{fC3biC3bHL}] + [\mathrm{fC3biC3bCR1}]} \cdots \\ \dfrac{1}{+ [\mathrm{fiC3biC3bCR1}] + [\mathrm{fiC3bC3dgCR1}] + [\mathrm{hC3bH}] + [\mathrm{hC3bHL}] + [\mathrm{hC3bCR1}] + [\mathrm{hiC3bCR1}] + [\mathrm{hC3bC4bH}] + [\mathrm{hC3bC4bHL}] + [\mathrm{hC3bC4bCR1}]} \cdots \\ \dfrac{1}{+ [\mathrm{hC3bC4bC4BP}] + [\mathrm{hC3bC4dH}] + [\mathrm{hC3bC4dHL}] + [\mathrm{hC3bC4dCR1}] + [\mathrm{hiC3bC4dCR1}] + [\mathrm{hiC3bC4bCR1}] + [\mathrm{hiC3bC4bC4BP}] + [\mathrm{hC3dgC4bCR1}]} \cdots \\ \dfrac{1}{+ [\mathrm{hC3dgC4bC4BP}] + [\mathrm{hC3bC3bH}] + [\mathrm{hC3bC3bHL}] + [\mathrm{hC3bC3bCR1}] + [\mathrm{hC3biC3bH}] + [\mathrm{hC3biC3bHL}] + [\mathrm{hC3biC3bCR1}] + [\mathrm{hiC3biC3bCR1}]} \cdots \\ \dfrac{1}{+ [\mathrm{hiC3bC3dgCR1}] + [\mathrm{fC4bCR1}] + [\mathrm{nfC4bC4BP}] + [\mathrm{fC4bC4BP}] + [\mathrm{fC4bC4bCR1}] + [\mathrm{fC4bC4bC4BP}] + [\mathrm{fC4bC4dCR1}] + [\mathrm{fC4bC4dC4BP}] + [\mathrm{hC4bCR1}]} \cdots \\ \dfrac{1}{+ [\mathrm{hC4bC4BP}] + [\mathrm{hC4bC4bCR1}] + [\mathrm{hC4bC4bC4BP}] + [\mathrm{hC4bC4dCR1}] + [\mathrm{hC4bC4dC4BP}]} \cdots \end{array} \right)$$

S117

$$\frac{d[\mathrm{fC3bBbH}]}{dt} = k^{+}_{\mathrm{C3bH}}[\mathrm{fC3bBb}][\mathrm{H}] - k^{-}_{\mathrm{C3bH}}[\mathrm{fC3bBbH}] - k^{-}_{\mathrm{C3bBbH}_{\mathrm{decay}}}[\mathrm{fC3bBbH}]$$

S118

$$\frac{d[\mathrm{fC3bHL}]}{dt} = k^{+}_{\mathrm{C3bHL}}[\mathrm{fC3b}][\mathrm{HL}] - k^{-}_{\mathrm{C3bHL}}[\mathrm{fC3bHL}]$$

$$-\left(\begin{array}{l} \dfrac{k^{\mathrm{FI}}_{\mathrm{catC3bH}}[\mathrm{I}][\mathrm{fC3bHL}]}{K^{\mathrm{FI}}_{\mathrm{mC3bH}} + [\mathrm{C3(H_2O)H}] + [\mathrm{C3(H_2O)HL}] + [\mathrm{fC3bH}] + [\mathrm{fC3bHL}] + [\mathrm{fC3bCR1}] + [\mathrm{fiC3bCR1}] + [\mathrm{IgGC3bC3bH}] + [\mathrm{IgGC3biC3bH}] + [\mathrm{IgGC3bC3bHL}]} \cdots \\ \dfrac{1}{+ [\mathrm{IgGC3biC3bHL}] + [\mathrm{IgGC3bC3bCR1}] + [\mathrm{IgGC3biC3bCR1}] + [\mathrm{IgGiC3biC3bCR1}] + [\mathrm{IgGiC3bC3dgCR1}] + [\mathrm{fC3bC4bH}] + [\mathrm{fC3bC4bHL}]} \cdots \\ \dfrac{1}{+ [\mathrm{fC3bC4bCR1}] + [\mathrm{fC3bC4bC4BP}] + [\mathrm{fC3bC4dH}] + [\mathrm{fC3bC4dHL}] + [\mathrm{fC3bC4dCR1}] + [\mathrm{fiC3bC4dCR1}] + [\mathrm{fiC3bC4bCR1}] + [\mathrm{fiC3bC4bC4BP}]} \cdots \\ \dfrac{1}{+ [\mathrm{fC3dgC4bCR1}] + [\mathrm{fC3dgC4bC4BP}] + [\mathrm{fC3bC3bH}] + [\mathrm{fC3bC3bHL}] + [\mathrm{fC3bC3bCR1}] + [\mathrm{fC3biC3bH}] + [\mathrm{fC3biC3bHL}] + [\mathrm{fC3biC3bCR1}]} \cdots \\ \dfrac{1}{+ [\mathrm{fiC3biC3bCR1}] + [\mathrm{fiC3bC3dgCR1}] + [\mathrm{hC3bH}] + [\mathrm{hC3bHL}] + [\mathrm{hC3bCR1}] + [\mathrm{hiC3bCR1}] + [\mathrm{hC3bC4bH}] + [\mathrm{hC3bC4bHL}] + [\mathrm{hC3bC4bCR1}]} \cdots \\ \dfrac{1}{+ [\mathrm{hC3bC4bC4BP}] + [\mathrm{hC3bC4dH}] + [\mathrm{hC3bC4dHL}] + [\mathrm{hC3bC4dCR1}] + [\mathrm{hiC3bC4dCR1}] + [\mathrm{hiC3bC4bCR1}] + [\mathrm{hiC3bC4bC4BP}] + [\mathrm{hC3dgC4bCR1}]} \cdots \\ \dfrac{1}{+ [\mathrm{hC3dgC4bC4BP}] + [\mathrm{hC3bC3bH}] + [\mathrm{hC3bC3bHL}] + [\mathrm{hC3bC3bCR1}] + [\mathrm{hC3biC3bH}] + [\mathrm{hC3biC3bHL}] + [\mathrm{hC3biC3bCR1}] + [\mathrm{hiC3biC3bCR1}]} \cdots \\ \dfrac{1}{+ [\mathrm{hiC3bC3dgCR1}] + [\mathrm{fC4bCR1}] + [\mathrm{nfC4bC4BP}] + [\mathrm{fC4bC4BP}] + [\mathrm{fC4bC4bCR1}] + [\mathrm{fC4bC4bC4BP}] + [\mathrm{fC4bC4dCR1}] + [\mathrm{fC4bC4dC4BP}] + [\mathrm{hC4bCR1}]} \cdots \\ \dfrac{1}{+ [\mathrm{hC4bC4BP}] + [\mathrm{hC4bC4bCR1}] + [\mathrm{hC4bC4bC4BP}] + [\mathrm{hC4bC4dCR1}] + [\mathrm{hC4bC4dC4BP}]} \cdots \end{array} \right)$$

S119

$$\frac{d[\text{fC3bBbHL}]}{dt} = k^{+}_{\text{C3bHL}}[\text{fC3bBb}][\text{HL}] - k^{-}_{\text{C3bHL}}[\text{fC3bBbHL}] - k^{-}_{\text{C3bBbHL}_{\text{decay}}}[\text{fC3bBbHL}]$$

S120

$$\frac{d[\text{fC3bCR1}]}{dt} = k^{+}_{\text{C3bCR1}}[\text{fC3b}][\text{CR1}] - k^{-}_{\text{C3bCR1}}[\text{fC3bCR1}]$$

$$- \left(\begin{array}{l} \dfrac{k^{\text{FI}}_{\text{catC3bH}}[\text{I}][\text{fC3bCR1}]}{K^{\text{FI}}_{\text{mC3bH}} + [\text{C3(H}_2\text{O)H}] + [\text{C3(H}_2\text{O)HL}] + [\text{fC3bH}] + [\text{fC3bHL}] + [\text{fC3bCR1}] + [\text{fiC3bCR1}] + [\text{IgGC3bC3bH}] + [\text{IgGC3biC3bH}] + [\text{IgGC3bC3bHL}]} \cdots \\ \dfrac{1}{+[\text{IgGC3biC3bHL}] + [\text{IgGC3bC3bCR1}] + [\text{IgGC3biC3bCR1}] + [\text{IgGiC3biC3bCR1}] + [\text{IgGiC3bC3dgCR1}] + [\text{fC3bC4bH}] + [\text{fC3bC4bHL}]} \cdots \\ \dfrac{1}{+[\text{fC3bC4bCR1}] + [\text{fC3bC4bC4BP}] + [\text{fC3bC4dH}] + [\text{fC3bC4dHL}] + [\text{fC3bC4dCR1}] + [\text{fiC3bC4dCR1}] + [\text{fiC3bC4bCR1}] + [\text{fiC3bC4bC4BP}]} \cdots \\ \dfrac{1}{+[\text{fC3dgC4bCR1}] + [\text{fC3dgC4bC4BP}] + [\text{fC3bC3bH}] + [\text{fC3bC3bHL}] + [\text{fC3bC3bCR1}] + [\text{fC3biC3bH}] + [\text{fC3biC3bHL}] + [\text{fC3biC3bCR1}]} \cdots \\ \dfrac{1}{+[\text{fiC3biC3bCR1}] + [\text{fiC3bC3dgCR1}] + [\text{hC3bH}] + [\text{hC3bHL}] + [\text{hC3bCR1}] + [\text{hiC3bCR1}] + [\text{hC3bC4bH}] + [\text{hC3bC4bHL}] + [\text{hC3bC4bCR1}]} \cdots \\ \dfrac{1}{+[\text{hC3bC4bC4BP}] + [\text{hC3bC4dH}] + [\text{hC3bC4dHL}] + [\text{hC3bC4dCR1}] + [\text{hiC3bC4dCR1}] + [\text{hiC3bC4bCR1}] + [\text{hiC3bC4bC4BP}] + [\text{hC3dgC4bCR1}]} \cdots \\ \dfrac{1}{+[\text{hC3dgC4bC4BP}] + [\text{hC3bC3bH}] + [\text{hC3bC3bHL}] + [\text{hC3bC3bCR1}] + [\text{hC3biC3bH}] + [\text{hC3biC3bHL}] + [\text{hC3biC3bCR1}] + [\text{hiC3biC3bCR1}]} \cdots \\ \dfrac{1}{+[\text{hiC3bC3dgCR1}] + [\text{fC4bCR1}] + [\text{nfC4bC4BP}] + [\text{fC4bC4BP}] + [\text{fC4bC4bCR1}] + [\text{fC4bC4bC4BP}] + [\text{fC4bC4dCR1}] + [\text{fC4bC4dC4BP}] + [\text{hC4bCR1}]} \cdots \\ \dfrac{1}{+[\text{hC4bC4BP}] + [\text{hC4bC4bCR1}] + [\text{hC4bC4bC4BP}] + [\text{hC4bC4dCR1}] + [\text{hC4bC4dC4BP}]} \cdots \end{array} \right)$$

S121

$$\frac{d[\text{fC3bBbCR1}]}{dt} = k^{+}_{\text{C3bCR1}}[\text{fC3bBb}][\text{CR1}] - k^{-}_{\text{C3bCR1}}[\text{fC3bBbCR1}] - k^{-}_{\text{C3bBbCR1}_{\text{decay}}}[\text{fC3bBbCR1}]$$

S122

$$
\frac{d[\text{fiC3b}]}{dt} =
$$

$$
\left(
\begin{array}{l}
\frac{k_{\text{catC3bH}}^{\text{FI}}[\text{I}][\text{fC3bH}]}{K_{\text{mC3bH}}^{\text{FI}}+[\text{C3(H}_2\text{O)H}]+[\text{C3(H}_2\text{O)HL}]+[\text{fC3bH}]+[\text{fC3bHL}]+[\text{fC3bCR1}]+[\text{fiC3bCR1}]+[\text{IgGC3bC3bH}]+[\text{IgGC3biC3bH}]+[\text{IgGC3bC3bHL}]}\cdots \\
\frac{1}{+[\text{IgGC3biC3bHL}]+[\text{IgGC3bC3bCR1}]+[\text{IgGC3biC3bCR1}]+[\text{IgGiC3biC3bCR1}]+[\text{IgGiC3bC3dgCR1}]+[\text{fC3bC4bH}]+[\text{fC3bC4bHL}]}\cdots \\
\frac{1}{+[\text{fC3bC4bCR1}]+[\text{fC3bC4bC4BP}]+[\text{fC3bC4dH}]+[\text{fC3bC4dHL}]+[\text{fC3bC4dCR1}]+[\text{fiC3bC4dCR1}]+[\text{fiC3bC4bCR1}]+[\text{fiC3bC4bC4BP}]}\cdots \\
\frac{1}{+[\text{fC3dgC4bCR1}]+[\text{fC3dgC4bC4BP}]+[\text{fC3bC3bH}]+[\text{fC3bC3bHL}]+[\text{fC3bC3bCR1}]+[\text{fC3biC3bH}]+[\text{fC3biC3bHL}]+[\text{fC3biC3bCR1}]}\cdots \\
\frac{1}{+[\text{fiC3biC3bCR1}]+[\text{fiC3bC3dgCR1}]+[\text{hC3bH}]+[\text{hC3bHL}]+[\text{hC3bCR1}]+[\text{hiC3bCR1}]+[\text{hC3bC4bH}]+[\text{hC3bC4bHL}]+[\text{hC3bC4bCR1}]}\cdots \\
\frac{1}{+[\text{hC3bC4bC4BP}]+[\text{hC3bC4dH}]+[\text{hC3bC4dHL}]+[\text{hC3bC4dCR1}]+[\text{hiC3bC4dCR1}]+[\text{hiC3bC4bCR1}]+[\text{hiC3bC4bC4BP}]+[\text{hC3dgC4bCR1}]}\cdots \\
\frac{1}{+[\text{hC3dgC4bC4BP}]+[\text{hC3bC3bH}]+[\text{hC3bC3bHL}]+[\text{hC3bC3bCR1}]+[\text{hC3biC3bH}]+[\text{hC3biC3bHL}]+[\text{hC3biC3bCR1}]+[\text{hiC3biC3bCR1}]}\cdots \\
\frac{1}{+[\text{hiC3bC3dgCR1}]+[\text{fC4bCR1}]+[\text{nfC4bC4BP}]+[\text{fC4bC4BP}]+[\text{fC4bC4bCR1}]+[\text{fC4bC4bC4BP}]+[\text{fC4bC4dCR1}]+[\text{fC4bC4dC4BP}]+[\text{hC4bCR1}]}\cdots \\
\frac{1}{+[\text{hC4bC4BP}]+[\text{hC4bC4bCR1}]+[\text{hC4bC4bC4BP}]+[\text{hC4bC4dCR1}]+[\text{hC4bC4dC4BP}]}\cdots
\end{array}
\right)
$$

$$
+\left(
\begin{array}{l}
\frac{k_{\text{catC3bH}}^{\text{FI}}[\text{I}][\text{fC3bHL}]}{K_{\text{mC3bH}}^{\text{FI}}+[\text{C3(H}_2\text{O)H}]+[\text{C3(H}_2\text{O)HL}]+[\text{fC3bH}]+[\text{fC3bHL}]+[\text{fC3bCR1}]+[\text{fiC3bCR1}]+[\text{IgGC3bC3bH}]+[\text{IgGC3biC3bH}]+[\text{IgGC3bC3bHL}]}\cdots \\
\frac{1}{+[\text{IgGC3biC3bHL}]+[\text{IgGC3bC3bCR1}]+[\text{IgGC3biC3bCR1}]+[\text{IgGiC3biC3bCR1}]+[\text{IgGiC3bC3dgCR1}]+[\text{fC3bC4bH}]+[\text{fC3bC4bHL}]}\cdots \\
\frac{1}{+[\text{fC3bC4bCR1}]+[\text{fC3bC4bC4BP}]+[\text{fC3bC4dH}]+[\text{fC3bC4dHL}]+[\text{fC3bC4dCR1}]+[\text{fiC3bC4dCR1}]+[\text{fiC3bC4bCR1}]+[\text{fiC3bC4bC4BP}]}\cdots \\
\frac{1}{+[\text{fC3dgC4bCR1}]+[\text{fC3dgC4bC4BP}]+[\text{fC3bC3bH}]+[\text{fC3bC3bHL}]+[\text{fC3bC3bCR1}]+[\text{fC3biC3bH}]+[\text{fC3biC3bHL}]+[\text{fC3biC3bCR1}]}\cdots \\
\frac{1}{+[\text{fiC3biC3bCR1}]+[\text{fiC3bC3dgCR1}]+[\text{hC3bH}]+[\text{hC3bHL}]+[\text{hC3bCR1}]+[\text{hiC3bCR1}]+[\text{hC3bC4bH}]+[\text{hC3bC4bHL}]+[\text{hC3bC4bCR1}]}\cdots \\
\frac{1}{+[\text{hC3bC4bC4BP}]+[\text{hC3bC4dH}]+[\text{hC3bC4dHL}]+[\text{hC3bC4dCR1}]+[\text{hiC3bC4dCR1}]+[\text{hiC3bC4bCR1}]+[\text{hiC3bC4bC4BP}]+[\text{hC3dgC4bCR1}]}\cdots \\
\frac{1}{+[\text{hC3dgC4bC4BP}]+[\text{hC3bC3bH}]+[\text{hC3bC3bHL}]+[\text{hC3bC3bCR1}]+[\text{hC3biC3bH}]+[\text{hC3biC3bHL}]+[\text{hC3biC3bCR1}]+[\text{hiC3biC3bCR1}]}\cdots \\
\frac{1}{+[\text{hiC3bC3dgCR1}]+[\text{fC4bCR1}]+[\text{nfC4bC4BP}]+[\text{fC4bC4BP}]+[\text{fC4bC4bCR1}]+[\text{fC4bC4bC4BP}]+[\text{fC4bC4dCR1}]+[\text{fC4bC4dC4BP}]+[\text{hC4bCR1}]}\cdots \\
\frac{1}{+[\text{hC4bC4BP}]+[\text{hC4bC4bCR1}]+[\text{hC4bC4bC4BP}]+[\text{hC4bC4dCR1}]+[\text{hC4bC4dC4BP}]}\cdots
\end{array}
\right)
$$

$$
+\left(\begin{array}{l}
\frac{k^{FI}_{catC3bH}[I][fC3bCR1]}{K^{FI}_{mC3bH}+[C3(H_2O)H]+[C3(H_2O)HL]+[fC3bH]+[fC3bHL]+[fC3bCR1]+[fiC3bCR1]+[IgGC3bC3bH]+[IgGC3biC3bH]+[IgGC3bC3bHL]}\cdots \\
\frac{1}{+[IgGC3biC3bHL]+[IgGC3bC3bCR1]+[IgGC3biC3bCR1]+[IgGiC3biC3bCR1]+[IgGiC3bC3dgCR1]+[fC3bC4bH]+[fC3bC4bHL]}\cdots \\
\frac{1}{+[fC3bC4bCR1]+[fC3bC4bC4BP]+[fC3bC4dH]+[fC3bC4dHL]+[fC3bC4dCR1]+[fiC3bC4dCR1]+[fiC3bC4bCR1]+[fiC3bC4bC4BP]}\cdots \\
\frac{1}{+[fC3dgC4bCR1]+[fC3dgC4bC4BP]+[fC3bC3bH]+[fC3bC3bHL]+[fC3bC3bCR1]+[fC3biC3bH]+[fC3biC3bHL]+[fC3biC3bCR1]}\cdots \\
\frac{1}{+[fiC3biC3bCR1]+[fiC3bC3dgCR1]+[hC3bH]+[hC3bHL]+[hC3bCR1]+[hiC3bCR1]+[hC3bC4bH]+[hC3bC4bHL]+[hC3bC4bCR1]}\cdots \\
\frac{1}{+[hC3bC4bC4BP]+[hC3bC4dH]+[hC3bC4dHL]+[hC3bC4dCR1]+[hiC3bC4dCR1]+[hiC3bC4bCR1]+[hiC3bC4bC4BP]+[hC3dgC4bCR1]}\cdots \\
\frac{1}{+[hC3dgC4bC4BP]+[hC3bC3bH]+[hC3bC3bHL]+[hC3bC3bCR1]+[hC3biC3bH]+[hC3biC3bHL]+[hC3biC3bCR1]+[hiC3biC3bCR1]}\cdots \\
\frac{1}{+[hiC3bC3dgCR1]+[fC4bCR1]+[nfC4bC4BP]+[fC4bC4BP]+[fC4bC4bCR1]+[fC4bC4bC4BP]+[fC4bC4dCR1]+[fC4bC4dC4BP]+[hC4bCR1]}\cdots \\
\frac{1}{+[hC4bC4BP]+[hC4bC4bCR1]+[hC4bC4bC4BP]+[hC4bC4dCR1]+[hC4bC4dC4BP]}\cdots
\end{array}\right)
$$

$$
-k^{+}_{iC3bCR1}[fiC3b][CR1]+k^{-}_{iC3bCR1}[fiC3bCR1]
$$

S123

$$
\frac{d[fiC3bCR1]}{dt}=k^{+}_{iC3bCR1}[fiC3b][CR1]-k^{-}_{iC3bCR1}[fiC3bCR1]
$$

$$
-\left(\begin{array}{l}
\frac{k^{FI}_{catC3bH}[I][fiC3bCR1]}{K^{FI}_{mC3bH}+[C3(H_2O)H]+[C3(H_2O)HL]+[fC3bH]+[fC3bHL]+[fC3bCR1]+[fiC3bCR1]+[IgGC3bC3bH]+[IgGC3biC3bH]+[IgGC3bC3bHL]}\cdots \\
\frac{1}{+[IgGC3biC3bHL]+[IgGC3bC3bCR1]+[IgGC3biC3bCR1]+[IgGiC3biC3bCR1]+[IgGiC3bC3dgCR1]+[fC3bC4bH]+[fC3bC4bHL]}\cdots \\
\frac{1}{+[fC3bC4bCR1]+[fC3bC4bC4BP]+[fC3bC4dH]+[fC3bC4dHL]+[fC3bC4dCR1]+[fiC3bC4dCR1]+[fiC3bC4bCR1]+[fiC3bC4bC4BP]}\cdots \\
\frac{1}{+[fC3dgC4bCR1]+[fC3dgC4bC4BP]+[fC3bC3bH]+[fC3bC3bHL]+[fC3bC3bCR1]+[fC3biC3bH]+[fC3biC3bHL]+[fC3biC3bCR1]}\cdots \\
\frac{1}{+[fiC3biC3bCR1]+[fiC3bC3dgCR1]+[hC3bH]+[hC3bHL]+[hC3bCR1]+[hiC3bCR1]+[hC3bC4bH]+[hC3bC4bHL]+[hC3bC4bCR1]}\cdots \\
\frac{1}{+[hC3bC4bC4BP]+[hC3bC4dH]+[hC3bC4dHL]+[hC3bC4dCR1]+[hiC3bC4dCR1]+[hiC3bC4bCR1]+[hiC3bC4bC4BP]+[hC3dgC4bCR1]}\cdots \\
\frac{1}{+[hC3dgC4bC4BP]+[hC3bC3bH]+[hC3bC3bHL]+[hC3bC3bCR1]+[hC3biC3bH]+[hC3biC3bHL]+[hC3biC3bCR1]+[hiC3biC3bCR1]}\cdots \\
\frac{1}{+[hiC3bC3dgCR1]+[fC4bCR1]+[nfC4bC4BP]+[fC4bC4BP]+[fC4bC4bCR1]+[fC4bC4bC4BP]+[fC4bC4dCR1]+[fC4bC4dC4BP]+[hC4bCR1]}\cdots \\
\frac{1}{+[hC4bC4BP]+[hC4bC4bCR1]+[hC4bC4bC4BP]+[hC4bC4dCR1]+[hC4bC4dC4BP]}\cdots
\end{array}\right)
$$

S124

$$\frac{d[\text{fC3dg}]}{dt}=$$

$$\left(\begin{array}{l}
\dfrac{k_{\text{catC3bH}}^{\text{FI}}[\text{I}][\text{fiC3bCR1}]}{K_{\text{mC3bH}}^{\text{FI}}+[\text{C3(H}_2\text{O)H}]+[\text{C3(H}_2\text{O)HL}]+[\text{fC3bH}]+[\text{fC3bHL}]+[\text{fC3bCR1}]+[\text{fiC3bCR1}]+[\text{IgGC3bC3bH}]+[\text{IgGC3biC3bH}]+[\text{IgGC3bC3bHL}]}\cdots \\
\dfrac{1}{+[\text{IgGC3biC3bHL}]+[\text{IgGC3bC3bCR1}]+[\text{IgGC3biC3bCR1}]+[\text{IgGiC3biC3bCR1}]+[\text{IgGiC3bC3dgCR1}]+[\text{fC3bC4bH}]+[\text{fC3bC4bHL}]}\cdots \\
\dfrac{1}{+[\text{fC3bC4bCR1}]+[\text{fC3bC4bC4BP}]+[\text{fC3bC4dH}]+[\text{fC3bC4dHL}]+[\text{fC3bC4dCR1}]+[\text{fiC3bC4dCR1}]+[\text{fiC3bC4bCR1}]+[\text{fiC3bC4bC4BP}]}\cdots \\
\dfrac{1}{+[\text{fC3dgC4bCR1}]+[\text{fC3dgC4bC4BP}]+[\text{fC3bC3bH}]+[\text{fC3bC3bHL}]+[\text{fC3bC3bCR1}]+[\text{fC3biC3bH}]+[\text{fC3biC3bHL}]+[\text{fC3biC3bCR1}]}\cdots \\
\dfrac{1}{+[\text{fiC3biC3bCR1}]+[\text{fiC3bC3dgCR1}]+[\text{hC3bH}]+[\text{hC3bHL}]+[\text{hC3bCR1}]+[\text{hiC3bCR1}]+[\text{hC3bC4bH}]+[\text{hC3bC4bHL}]+[\text{hC3bC4bCR1}]}\cdots \\
\dfrac{1}{+[\text{hC3bC4bC4BP}]+[\text{hC3bC4dH}]+[\text{hC3bC4dHL}]+[\text{hC3bC4dCR1}]+[\text{hiC3bC4dCR1}]+[\text{hiC3bC4bCR1}]+[\text{hiC3bC4bC4BP}]+[\text{hC3dgC4bCR1}]}\cdots \\
\dfrac{1}{+[\text{hC3dgC4bC4BP}]+[\text{hC3bC3bH}]+[\text{hC3bC3bHL}]+[\text{hC3bC3bCR1}]+[\text{hC3biC3bH}]+[\text{hC3biC3bHL}]+[\text{hC3biC3bCR1}]+[\text{hiC3biC3bCR1}]}\cdots \\
\dfrac{1}{+[\text{hiC3bC3dgCR1}]+[\text{fC4bCR1}]+[\text{nfC4bC4BP}]+[\text{fC4bC4BP}]+[\text{fC4bC4bCR1}]+[\text{fC4bC4bC4BP}]+[\text{fC4bC4dCR1}]+[\text{fC4bC4dC4BP}]+[\text{hC4bCR1}]}\cdots \\
\dfrac{1}{+[\text{hC4bC4BP}]+[\text{hC4bC4bCR1}]+[\text{hC4bC4bC4BP}]+[\text{hC4bC4dCR1}]+[\text{hC4bC4dC4BP}]}\cdots
\end{array}\right)$$

S125

$$\frac{d[\text{IgGC3bC3bH}]}{dt}=k_{\text{C3bH}}^{+}[\text{IgGC3bC3b}][\text{H}]-k_{\text{C3bH}}^{-}[\text{IgGC3bC3bH}]$$

$$-\left(\begin{array}{l}
\dfrac{k_{\text{catC3bH}}^{\text{FI}}[\text{I}][\text{IgGC3bC3bH}]}{K_{\text{mC3bH}}^{\text{FI}}+[\text{C3(H}_2\text{O)H}]+[\text{C3(H}_2\text{O)HL}]+[\text{fC3bH}]+[\text{fC3bHL}]+[\text{fC3bCR1}]+[\text{fiC3bCR1}]+[\text{IgGC3bC3bH}]+[\text{IgGC3biC3bH}]+[\text{IgGC3bC3bHL}]}\cdots \\
\dfrac{1}{+[\text{IgGC3biC3bHL}]+[\text{IgGC3bC3bCR1}]+[\text{IgGC3biC3bCR1}]+[\text{IgGiC3biC3bCR1}]+[\text{IgGiC3bC3dgCR1}]+[\text{fC3bC4bH}]+[\text{fC3bC4bHL}]}\cdots \\
\dfrac{1}{+[\text{fC3bC4bCR1}]+[\text{fC3bC4bC4BP}]+[\text{fC3bC4dH}]+[\text{fC3bC4dHL}]+[\text{fC3bC4dCR1}]+[\text{fiC3bC4dCR1}]+[\text{fiC3bC4bCR1}]+[\text{fiC3bC4bC4BP}]}\cdots \\
\dfrac{1}{+[\text{fC3dgC4bCR1}]+[\text{fC3dgC4bC4BP}]+[\text{fC3bC3bH}]+[\text{fC3bC3bHL}]+[\text{fC3bC3bCR1}]+[\text{fC3biC3bH}]+[\text{fC3biC3bHL}]+[\text{fC3biC3bCR1}]}\cdots \\
\dfrac{1}{+[\text{fiC3biC3bCR1}]+[\text{fiC3bC3dgCR1}]+[\text{hC3bH}]+[\text{hC3bHL}]+[\text{hC3bCR1}]+[\text{hiC3bCR1}]+[\text{hC3bC4bH}]+[\text{hC3bC4bHL}]+[\text{hC3bC4bCR1}]}\cdots \\
\dfrac{1}{+[\text{hC3bC4bC4BP}]+[\text{hC3bC4dH}]+[\text{hC3bC4dHL}]+[\text{hC3bC4dCR1}]+[\text{hiC3bC4dCR1}]+[\text{hiC3bC4bCR1}]+[\text{hiC3bC4bC4BP}]+[\text{hC3dgC4bCR1}]}\cdots \\
\dfrac{1}{+[\text{hC3dgC4bC4BP}]+[\text{hC3bC3bH}]+[\text{hC3bC3bHL}]+[\text{hC3bC3bCR1}]+[\text{hC3biC3bH}]+[\text{hC3biC3bHL}]+[\text{hC3biC3bCR1}]+[\text{hiC3biC3bCR1}]}\cdots \\
\dfrac{1}{+[\text{hiC3bC3dgCR1}]+[\text{fC4bCR1}]+[\text{nfC4bC4BP}]+[\text{fC4bC4BP}]+[\text{fC4bC4bCR1}]+[\text{fC4bC4bC4BP}]+[\text{fC4bC4dCR1}]+[\text{fC4bC4dC4BP}]+[\text{hC4bCR1}]}\cdots \\
\dfrac{1}{+[\text{hC4bC4BP}]+[\text{hC4bC4bCR1}]+[\text{hC4bC4bC4BP}]+[\text{hC4bC4dCR1}]+[\text{hC4bC4dC4BP}]}\cdots
\end{array}\right)$$

S126

$$\frac{d[\text{IgGC3bC3bHL}]}{dt} = k^{+}_{\text{C3bH}}[\text{IgGC3bC3b}][\text{HL}] - k^{-}_{\text{C3bH}}[\text{IgGC3bC3bHL}]$$

$$-\left(\begin{array}{l}
\dfrac{k^{\text{FI}}_{\text{catC3bH}}[\text{I}][\text{IgGC3bC3bHL}]}{K^{\text{FI}}_{\text{mC3bH}} + [\text{C3(H}_2\text{O)H}] + [\text{C3(H}_2\text{O)HL}] + [\text{fC3bH}] + [\text{fC3bHL}] + [\text{fC3bCR1}] + [\text{fiC3bCR1}] + [\text{IgGC3bC3bH}] + [\text{IgGC3biC3bH}] + [\text{IgGC3bC3bHL}]} \cdots \\
\dfrac{1}{+[\text{IgGC3biC3bHL}] + [\text{IgGC3bC3bCR1}] + [\text{IgGC3biC3bCR1}] + [\text{IgGiC3biC3bCR1}] + [\text{IgGiC3bC3dgCR1}] + [\text{fC3bC4bH}] + [\text{fC3bC4bHL}]} \cdots \\
\dfrac{1}{+[\text{fC3bC4bCR1}] + [\text{fC3bC4bC4BP}] + [\text{fC3bC4dH}] + [\text{fC3bC4dHL}] + [\text{fC3bC4dCR1}] + [\text{fiC3bC4dCR1}] + [\text{fiC3bC4bCR1}] + [\text{fiC3bC4bC4BP}]} \cdots \\
\dfrac{1}{+[\text{fC3dgC4bCR1}] + [\text{fC3dgC4bC4BP}] + [\text{fC3bC3bH}] + [\text{fC3bC3bHL}] + [\text{fC3bC3bCR1}] + [\text{fC3biC3bH}] + [\text{fC3biC3bHL}] + [\text{fC3biC3bCR1}]} \cdots \\
\dfrac{1}{+[\text{fiC3biC3bCR1}] + [\text{fiC3bC3dgCR1}] + [\text{hC3bH}] + [\text{hC3bHL}] + [\text{hC3bCR1}] + [\text{hiC3bCR1}] + [\text{hC3bC4bH}] + [\text{hC3bC4bHL}] + [\text{hC3bC4bCR1}]} \cdots \\
\dfrac{1}{+[\text{hC3bC4bC4BP}] + [\text{hC3bC4dH}] + [\text{hC3bC4dHL}] + [\text{hC3bC4dCR1}] + [\text{hiC3bC4dCR1}] + [\text{hiC3bC4bCR1}] + [\text{hiC3bC4bC4BP}] + [\text{hC3dgC4bCR1}]} \cdots \\
\dfrac{1}{+[\text{hC3dgC4bC4BP}] + [\text{hC3bC3bH}] + [\text{hC3bC3bHL}] + [\text{hC3bC3bCR1}] + [\text{hC3biC3bH}] + [\text{hC3biC3bHL}] + [\text{hC3biC3bCR1}] + [\text{hiC3biC3bCR1}]} \cdots \\
\dfrac{1}{+[\text{hiC3bC3dgCR1}] + [\text{fC4bCR1}] + [\text{nfC4bC4BP}] + [\text{fC4bC4BP}] + [\text{fC4bC4bCR1}] + [\text{fC4bC4bC4BP}] + [\text{fC4bC4dCR1}] + [\text{fC4bC4dC4BP}] + [\text{hC4bCR1}]} \cdots \\
\dfrac{1}{+[\text{hC4bC4BP}] + [\text{hC4bC4bCR1}] + [\text{hC4bC4bC4BP}] + [\text{hC4bC4dCR1}] + [\text{hC4bC4dC4BP}]} \cdots
\end{array} \right)$$

S127

$$\frac{d[\text{IgGC3bC3bCR1}]}{dt} = k^{+}_{\text{C3bC3bCR1}}[\text{IgGC3bC3b}][\text{CR1}] - k^{-}_{\text{C3bC3bCR1}}[\text{IgGC3bC3bCR1}]$$

$$-\left(\begin{array}{l}
\dfrac{k^{\text{FI}}_{\text{catC3bH}}[\text{I}][\text{IgGC3bC3bCR1}]}{K^{\text{FI}}_{\text{mC3bH}} + [\text{C3(H}_2\text{O)H}] + [\text{C3(H}_2\text{O)HL}] + [\text{fC3bH}] + [\text{fC3bHL}] + [\text{fC3bCR1}] + [\text{fiC3bCR1}] + [\text{IgGC3bC3bH}] + [\text{IgGC3biC3bH}] + [\text{IgGC3bC3bHL}]} \cdots \\
\dfrac{1}{+[\text{IgGC3biC3bHL}] + [\text{IgGC3bC3bCR1}] + [\text{IgGC3biC3bCR1}] + [\text{IgGiC3biC3bCR1}] + [\text{IgGiC3bC3dgCR1}] + [\text{fC3bC4bH}] + [\text{fC3bC4bHL}]} \cdots \\
\dfrac{1}{+[\text{fC3bC4bCR1}] + [\text{fC3bC4bC4BP}] + [\text{fC3bC4dH}] + [\text{fC3bC4dHL}] + [\text{fC3bC4dCR1}] + [\text{fiC3bC4dCR1}] + [\text{fiC3bC4bCR1}] + [\text{fiC3bC4bC4BP}]} \cdots \\
\dfrac{1}{+[\text{fC3dgC4bCR1}] + [\text{fC3dgC4bC4BP}] + [\text{fC3bC3bH}] + [\text{fC3bC3bHL}] + [\text{fC3bC3bCR1}] + [\text{fC3biC3bH}] + [\text{fC3biC3bHL}] + [\text{fC3biC3bCR1}]} \cdots \\
\dfrac{1}{+[\text{fiC3biC3bCR1}] + [\text{fiC3bC3dgCR1}] + [\text{hC3bH}] + [\text{hC3bHL}] + [\text{hC3bCR1}] + [\text{hiC3bCR1}] + [\text{hC3bC4bH}] + [\text{hC3bC4bHL}] + [\text{hC3bC4bCR1}]} \cdots \\
\dfrac{1}{+[\text{hC3bC4bC4BP}] + [\text{hC3bC4dH}] + [\text{hC3bC4dHL}] + [\text{hC3bC4dCR1}] + [\text{hiC3bC4dCR1}] + [\text{hiC3bC4bCR1}] + [\text{hiC3bC4bC4BP}] + [\text{hC3dgC4bCR1}]} \cdots \\
\dfrac{1}{+[\text{hC3dgC4bC4BP}] + [\text{hC3bC3bH}] + [\text{hC3bC3bHL}] + [\text{hC3bC3bCR1}] + [\text{hC3biC3bH}] + [\text{hC3biC3bHL}] + [\text{hC3biC3bCR1}] + [\text{hiC3biC3bCR1}]} \cdots \\
\dfrac{1}{+[\text{hiC3bC3dgCR1}] + [\text{fC4bCR1}] + [\text{nfC4bC4BP}] + [\text{fC4bC4BP}] + [\text{fC4bC4bCR1}] + [\text{fC4bC4bC4BP}] + [\text{fC4bC4dCR1}] + [\text{fC4bC4dC4BP}] + [\text{hC4bCR1}]} \cdots \\
\dfrac{1}{+[\text{hC4bC4BP}] + [\text{hC4bC4bCR1}] + [\text{hC4bC4bC4BP}] + [\text{hC4bC4dCR1}] + [\text{hC4bC4dC4BP}]} \cdots
\end{array} \right)$$

S128

$$\frac{d[\mathrm{IgGC3biC3b}]}{dt} =$$

$$\left(\begin{array}{l}
\frac{k^{\mathrm{FI}}_{\mathrm{catC3bH}}[\mathrm{I}][\mathrm{IgGC3bC3bH}]}{K^{\mathrm{FI}}_{\mathrm{mC3bH}} + [\mathrm{C3(H_2O)H}] + [\mathrm{C3(H_2O)HL}] + [\mathrm{fC3bH}] + [\mathrm{fC3bHL}] + [\mathrm{fC3bCR1}] + [\mathrm{fiC3bCR1}] + [\mathrm{IgGC3bC3bH}] + [\mathrm{IgGC3biC3bH}] + [\mathrm{IgGC3bC3bHL}]} \cdots \\
\frac{1}{+ [\mathrm{IgGC3biC3bHL}] + [\mathrm{IgGC3bC3bCR1}] + [\mathrm{IgGC3biC3bCR1}] + [\mathrm{IgGiC3biC3bCR1}] + [\mathrm{IgGiC3bC3dgCR1}] + [\mathrm{fC3bC4bH}] + [\mathrm{fC3bC4bHL}]} \cdots \\
\frac{1}{+ [\mathrm{fC3bC4bCR1}] + [\mathrm{fC3bC4bC4BP}] + [\mathrm{fC3bC4dH}] + [\mathrm{fC3bC4dHL}] + [\mathrm{fC3bC4dCR1}] + [\mathrm{fiC3bC4dCR1}] + [\mathrm{fiC3bC4bCR1}] + [\mathrm{fiC3bC4bC4BP}]} \cdots \\
\frac{1}{+ [\mathrm{fC3dgC4bCR1}] + [\mathrm{fC3dgC4bC4BP}] + [\mathrm{fC3bC3bH}] + [\mathrm{fC3bC3bHL}] + [\mathrm{fC3bC3bCR1}] + [\mathrm{fC3biC3bH}] + [\mathrm{fC3biC3bHL}] + [\mathrm{fC3biC3bCR1}]} \cdots \\
\frac{1}{+ [\mathrm{fiC3biC3bCR1}] + [\mathrm{fiC3bC3dgCR1}] + [\mathrm{hC3bH}] + [\mathrm{hC3bHL}] + [\mathrm{hC3bCR1}] + [\mathrm{hiC3bCR1}] + [\mathrm{hC3bC4bH}] + [\mathrm{hC3bC4bHL}] + [\mathrm{hC3bC4bCR1}]} \cdots \\
\frac{1}{+ [\mathrm{hC3bC4bC4BP}] + [\mathrm{hC3bC4dH}] + [\mathrm{hC3bC4dHL}] + [\mathrm{hC3bC4dCR1}] + [\mathrm{hiC3bC4dCR1}] + [\mathrm{hiC3bC4bCR1}] + [\mathrm{hiC3bC4bC4BP}] + [\mathrm{hC3dgC4bCR1}]} \cdots \\
\frac{1}{+ [\mathrm{hC3dgC4bC4BP}] + [\mathrm{hC3bC3bH}] + [\mathrm{hC3bC3bHL}] + [\mathrm{hC3bC3bCR1}] + [\mathrm{hC3biC3bH}] + [\mathrm{hC3biC3bHL}] + [\mathrm{hC3biC3bCR1}] + [\mathrm{hiC3biC3bCR1}]} \cdots \\
\frac{1}{+ [\mathrm{hiC3bC3dgCR1}] + [\mathrm{fC4bCR1}] + [\mathrm{nfC4bC4BP}] + [\mathrm{fC4bC4BP}] + [\mathrm{fC4bC4bCR1}] + [\mathrm{fC4bC4bC4BP}] + [\mathrm{fC4bC4dCR1}] + [\mathrm{fC4bC4dC4BP}] + [\mathrm{hC4bCR1}]} \cdots \\
\frac{1}{+ [\mathrm{hC4bC4BP}] + [\mathrm{hC4bC4bCR1}] + [\mathrm{hC4bC4bC4BP}] + [\mathrm{hC4bC4dCR1}] + [\mathrm{hC4bC4dC4BP}]} \cdots
\end{array} \right)$$

$$+ \left(\begin{array}{l}
\frac{k^{\mathrm{FI}}_{\mathrm{catC3bH}}[\mathrm{I}][\mathrm{IgGC3bC3bHL}]}{K^{\mathrm{FI}}_{\mathrm{mC3bH}} + [\mathrm{C3(H_2O)H}] + [\mathrm{C3(H_2O)HL}] + [\mathrm{fC3bH}] + [\mathrm{fC3bHL}] + [\mathrm{fC3bCR1}] + [\mathrm{fiC3bCR1}] + [\mathrm{IgGC3bC3bH}] + [\mathrm{IgGC3biC3bH}] + [\mathrm{IgGC3bC3bHL}]} \cdots \\
\frac{1}{+ [\mathrm{IgGC3biC3bHL}] + [\mathrm{IgGC3bC3bCR1}] + [\mathrm{IgGC3biC3bCR1}] + [\mathrm{IgGiC3biC3bCR1}] + [\mathrm{IgGiC3bC3dgCR1}] + [\mathrm{fC3bC4bH}] + [\mathrm{fC3bC4bHL}]} \cdots \\
\frac{1}{+ [\mathrm{fC3bC4bCR1}] + [\mathrm{fC3bC4bC4BP}] + [\mathrm{fC3bC4dH}] + [\mathrm{fC3bC4dHL}] + [\mathrm{fC3bC4dCR1}] + [\mathrm{fiC3bC4dCR1}] + [\mathrm{fiC3bC4bCR1}] + [\mathrm{fiC3bC4bC4BP}]} \cdots \\
\frac{1}{+ [\mathrm{fC3dgC4bCR1}] + [\mathrm{fC3dgC4bC4BP}] + [\mathrm{fC3bC3bH}] + [\mathrm{fC3bC3bHL}] + [\mathrm{fC3bC3bCR1}] + [\mathrm{fC3biC3bH}] + [\mathrm{fC3biC3bHL}] + [\mathrm{fC3biC3bCR1}]} \cdots \\
\frac{1}{+ [\mathrm{fiC3biC3bCR1}] + [\mathrm{fiC3bC3dgCR1}] + [\mathrm{hC3bH}] + [\mathrm{hC3bHL}] + [\mathrm{hC3bCR1}] + [\mathrm{hiC3bCR1}] + [\mathrm{hC3bC4bH}] + [\mathrm{hC3bC4bHL}] + [\mathrm{hC3bC4bCR1}]} \cdots \\
\frac{1}{+ [\mathrm{hC3bC4bC4BP}] + [\mathrm{hC3bC4dH}] + [\mathrm{hC3bC4dHL}] + [\mathrm{hC3bC4dCR1}] + [\mathrm{hiC3bC4dCR1}] + [\mathrm{hiC3bC4bCR1}] + [\mathrm{hiC3bC4bC4BP}] + [\mathrm{hC3dgC4bCR1}]} \cdots \\
\frac{1}{+ [\mathrm{hC3dgC4bC4BP}] + [\mathrm{hC3bC3bH}] + [\mathrm{hC3bC3bHL}] + [\mathrm{hC3bC3bCR1}] + [\mathrm{hC3biC3bH}] + [\mathrm{hC3biC3bHL}] + [\mathrm{hC3biC3bCR1}] + [\mathrm{hiC3biC3bCR1}]} \cdots \\
\frac{1}{+ [\mathrm{hiC3bC3dgCR1}] + [\mathrm{fC4bCR1}] + [\mathrm{nfC4bC4BP}] + [\mathrm{fC4bC4BP}] + [\mathrm{fC4bC4bCR1}] + [\mathrm{fC4bC4bC4BP}] + [\mathrm{fC4bC4dCR1}] + [\mathrm{fC4bC4dC4BP}] + [\mathrm{hC4bCR1}]} \cdots \\
\frac{1}{+ [\mathrm{hC4bC4BP}] + [\mathrm{hC4bC4bCR1}] + [\mathrm{hC4bC4bC4BP}] + [\mathrm{hC4bC4dCR1}] + [\mathrm{hC4bC4dC4BP}]} \cdots
\end{array} \right)$$

$$
+\left(\begin{array}{l}
\dfrac{k^{\mathrm{FI}}_{\mathrm{catC3bH}}[\mathrm{I}][\mathrm{IgGC3bC3bCR1}]}{K^{\mathrm{FI}}_{\mathrm{mC3bH}}+[\mathrm{C3(H_2O)H}]+[\mathrm{C3(H_2O)HL}]+[\mathrm{fC3bH}]+[\mathrm{fC3bHL}]+[\mathrm{fC3bCR1}]+[\mathrm{fiC3bCR1}]+[\mathrm{IgGC3bC3bH}]+[\mathrm{IgGC3biC3bH}]+[\mathrm{IgGC3bC3bHL}]}\cdots \\
\dfrac{1}{+[\mathrm{IgGC3biC3bHL}]+[\mathrm{IgGC3bC3bCR1}]+[\mathrm{IgGC3biC3bCR1}]+[\mathrm{IgGiC3biC3bCR1}]+[\mathrm{IgGiC3bC3dgCR1}]+[\mathrm{fC3bC4bH}]+[\mathrm{fC3bC4bHL}]}\cdots \\
\dfrac{1}{+[\mathrm{fC3bC4bCR1}]+[\mathrm{fC3bC4bC4BP}]+[\mathrm{fC3bC4dH}]+[\mathrm{fC3bC4dHL}]+[\mathrm{fC3bC4dCR1}]+[\mathrm{fiC3bC4dCR1}]+[\mathrm{fiC3bC4bCR1}]+[\mathrm{fiC3bC4bC4BP}]}\cdots \\
\dfrac{1}{+[\mathrm{fC3dgC4bCR1}]+[\mathrm{fC3dgC4bC4BP}]+[\mathrm{fC3bC3bH}]+[\mathrm{fC3bC3bHL}]+[\mathrm{fC3bC3bCR1}]+[\mathrm{fC3biC3bH}]+[\mathrm{fC3biC3bHL}]+[\mathrm{fC3biC3bCR1}]}\cdots \\
\dfrac{1}{+[\mathrm{fiC3biC3bCR1}]+[\mathrm{fiC3bC3dgCR1}]+[\mathrm{hC3bH}]+[\mathrm{hC3bHL}]+[\mathrm{hC3bCR1}]+[\mathrm{hiC3bCR1}]+[\mathrm{hC3bC4bH}]+[\mathrm{hC3bC4bHL}]+[\mathrm{hC3bC4bCR1}]}\cdots \\
\dfrac{1}{+[\mathrm{hC3bC4bC4BP}]+[\mathrm{hC3bC4dH}]+[\mathrm{hC3bC4dHL}]+[\mathrm{hC3bC4dCR1}]+[\mathrm{hiC3bC4dCR1}]+[\mathrm{hiC3bC4bCR1}]+[\mathrm{hiC3bC4bC4BP}]+[\mathrm{hC3dgC4bCR1}]}\cdots \\
\dfrac{1}{+[\mathrm{hC3dgC4bC4BP}]+[\mathrm{hC3bC3bH}]+[\mathrm{hC3bC3bHL}]+[\mathrm{hC3bC3bCR1}]+[\mathrm{hC3biC3bH}]+[\mathrm{hC3biC3bHL}]+[\mathrm{hC3biC3bCR1}]+[\mathrm{hiC3biC3bCR1}]}\cdots \\
\dfrac{1}{+[\mathrm{hiC3bC3dgCR1}]+[\mathrm{fC4bCR1}]+[\mathrm{nfC4bC4BP}]+[\mathrm{fC4bC4BP}]+[\mathrm{fC4bC4bCR1}]+[\mathrm{fC4bC4bC4BP}]+[\mathrm{fC4bC4dCR1}]+[\mathrm{fC4bC4dC4BP}]+[\mathrm{hC4bCR1}]}\cdots \\
\dfrac{1}{+[\mathrm{hC4bC4BP}]+[\mathrm{hC4bC4bCR1}]+[\mathrm{hC4bC4bC4BP}]+[\mathrm{hC4bC4dCR1}]+[\mathrm{hC4bC4dC4BP}]}\cdots
\end{array}\right)
$$

$$
-k^{+}_{\mathrm{C3bH}}[\mathrm{IgGC3biC3b}][\mathrm{H}]+k^{-}_{\mathrm{C3bH}}[\mathrm{IgGC3biC3bH}]-k^{+}_{\mathrm{C3bHL}}[\mathrm{IgGC3biC3b}][\mathrm{HL}]+k^{-}_{\mathrm{C3bHL}}[\mathrm{IgGC3biC3bHL}]-k^{+}_{\mathrm{C3biC3bCR1}}[\mathrm{IgGC3biC3b}][\mathrm{CR1}]
$$

$$
+k^{-}_{\mathrm{C3biC3bCR1}}[\mathrm{IgGC3biC3bCR1}]
$$

S129

$$
\frac{d[\mathrm{IgGC3biC3bH}]}{dt}=k^{+}_{\mathrm{C3bH}}[\mathrm{IgGC3biC3b}][\mathrm{H}]-k^{-}_{\mathrm{C3bH}}[\mathrm{IgGC3biC3bH}]
$$

$$
-\left(\begin{array}{l}
\dfrac{k^{\mathrm{FI}}_{\mathrm{catC3bH}}[\mathrm{I}][\mathrm{IgGC3biC3bH}]}{K^{\mathrm{FI}}_{\mathrm{mC3bH}}+[\mathrm{C3(H_2O)H}]+[\mathrm{C3(H_2O)HL}]+[\mathrm{fC3bH}]+[\mathrm{fC3bHL}]+[\mathrm{fC3bCR1}]+[\mathrm{fiC3bCR1}]+[\mathrm{IgGC3bC3bH}]+[\mathrm{IgGC3biC3bH}]+[\mathrm{IgGC3bC3bHL}]}\cdots \\
\dfrac{1}{+[\mathrm{IgGC3biC3bHL}]+[\mathrm{IgGC3bC3bCR1}]+[\mathrm{IgGC3biC3bCR1}]+[\mathrm{IgGiC3biC3bCR1}]+[\mathrm{IgGiC3bC3dgCR1}]+[\mathrm{fC3bC4bH}]+[\mathrm{fC3bC4bHL}]}\cdots \\
\dfrac{1}{+[\mathrm{fC3bC4bCR1}]+[\mathrm{fC3bC4bC4BP}]+[\mathrm{fC3bC4dH}]+[\mathrm{fC3bC4dHL}]+[\mathrm{fC3bC4dCR1}]+[\mathrm{fiC3bC4dCR1}]+[\mathrm{fiC3bC4bCR1}]+[\mathrm{fiC3bC4bC4BP}]}\cdots \\
\dfrac{1}{+[\mathrm{fC3dgC4bCR1}]+[\mathrm{fC3dgC4bC4BP}]+[\mathrm{fC3bC3bH}]+[\mathrm{fC3bC3bHL}]+[\mathrm{fC3bC3bCR1}]+[\mathrm{fC3biC3bH}]+[\mathrm{fC3biC3bHL}]+[\mathrm{fC3biC3bCR1}]}\cdots \\
\dfrac{1}{+[\mathrm{fiC3biC3bCR1}]+[\mathrm{fiC3bC3dgCR1}]+[\mathrm{hC3bH}]+[\mathrm{hC3bHL}]+[\mathrm{hC3bCR1}]+[\mathrm{hiC3bCR1}]+[\mathrm{hC3bC4bH}]+[\mathrm{hC3bC4bHL}]+[\mathrm{hC3bC4bCR1}]}\cdots \\
\dfrac{1}{+[\mathrm{hC3bC4bC4BP}]+[\mathrm{hC3bC4dH}]+[\mathrm{hC3bC4dHL}]+[\mathrm{hC3bC4dCR1}]+[\mathrm{hiC3bC4dCR1}]+[\mathrm{hiC3bC4bCR1}]+[\mathrm{hiC3bC4bC4BP}]+[\mathrm{hC3dgC4bCR1}]}\cdots \\
\dfrac{1}{+[\mathrm{hC3dgC4bC4BP}]+[\mathrm{hC3bC3bH}]+[\mathrm{hC3bC3bHL}]+[\mathrm{hC3bC3bCR1}]+[\mathrm{hC3biC3bH}]+[\mathrm{hC3biC3bHL}]+[\mathrm{hC3biC3bCR1}]+[\mathrm{hiC3biC3bCR1}]}\cdots \\
\dfrac{1}{+[\mathrm{hiC3bC3dgCR1}]+[\mathrm{fC4bCR1}]+[\mathrm{nfC4bC4BP}]+[\mathrm{fC4bC4BP}]+[\mathrm{fC4bC4bCR1}]+[\mathrm{fC4bC4bC4BP}]+[\mathrm{fC4bC4dCR1}]+[\mathrm{fC4bC4dC4BP}]+[\mathrm{hC4bCR1}]}\cdots \\
\dfrac{1}{+[\mathrm{hC4bC4BP}]+[\mathrm{hC4bC4bCR1}]+[\mathrm{hC4bC4bC4BP}]+[\mathrm{hC4bC4dCR1}]+[\mathrm{hC4bC4dC4BP}]}\cdots
\end{array}\right)
$$

S130

$$\frac{d[\text{IgGC3biC3bHL}]}{dt} = k^{+}_{\text{C3bHL}}[\text{IgGC3biC3b}][\text{HL}] - k^{-}_{\text{C3bHL}}[\text{IgGC3biC3bHL}]$$

$$-\left(\frac{k^{\text{FI}}_{\text{catC3bH}}[\text{I}][\text{IgGC3biC3bHL}]}{K^{\text{FI}}_{\text{mC3bH}} + [\text{C3(H}_2\text{O)H}] + [\text{C3(H}_2\text{O)HL}] + [\text{fC3bH}] + [\text{fC3bHL}] + [\text{fC3bCR1}] + [\text{fiC3bCR1}] + [\text{IgGC3bC3bH}] + [\text{IgGC3biC3bH}] + [\text{IgGC3bC3bHL}]}\cdots\right.$$

$$\frac{1}{+[\text{IgGC3biC3bHL}] + [\text{IgGC3bC3bCR1}] + [\text{IgGC3biC3bCR1}] + [\text{IgGiC3biC3bCR1}] + [\text{IgGiC3bC3dgCR1}] + [\text{fC3bC4bH}] + [\text{fC3bC4bHL}]}\cdots$$

$$\frac{1}{+[\text{fC3bC4bCR1}] + [\text{fC3bC4bC4BP}] + [\text{fC3bC4dH}] + [\text{fC3bC4dHL}] + [\text{fC3bC4dCR1}] + [\text{fiC3bC4dCR1}] + [\text{fiC3bC4bCR1}] + [\text{fiC3bC4bC4BP}]}\cdots$$

$$\frac{1}{+[\text{fC3dgC4bCR1}] + [\text{fC3dgC4bC4BP}] + [\text{fC3bC3bH}] + [\text{fC3bC3bHL}] + [\text{fC3bC3bCR1}] + [\text{fC3biC3bH}] + [\text{fC3biC3bHL}] + [\text{fC3biC3bCR1}]}\cdots$$

$$\frac{1}{+[\text{fiC3biC3bCR1}] + [\text{fiC3bC3dgCR1}] + [\text{hC3bH}] + [\text{hC3bHL}] + [\text{hC3bCR1}] + [\text{hiC3bCR1}] + [\text{hC3bC4bH}] + [\text{hC3bC4bHL}] + [\text{hC3bC4bCR1}]}\cdots$$

$$\frac{1}{+[\text{hC3bC4bC4BP}] + [\text{hC3bC4dH}] + [\text{hC3bC4dHL}] + [\text{hC3bC4dCR1}] + [\text{hiC3bC4dCR1}] + [\text{hiC3bC4bCR1}] + [\text{hiC3bC4bC4BP}] + [\text{hC3dgC4bCR1}]}\cdots$$

$$\frac{1}{+[\text{hC3dgC4bC4BP}] + [\text{hC3bC3bH}] + [\text{hC3bC3bHL}] + [\text{hC3bC3bCR1}] + [\text{hC3biC3bH}] + [\text{hC3biC3bHL}] + [\text{hC3biC3bCR1}] + [\text{hiC3biC3bCR1}]}\cdots$$

$$\frac{1}{+[\text{hiC3bC3dgCR1}] + [\text{fC4bCR1}] + [\text{nfC4bC4BP}] + [\text{fC4bC4BP}] + [\text{fC4bC4bCR1}] + [\text{fC4bC4bC4BP}] + [\text{fC4bC4dCR1}] + [\text{fC4bC4dC4BP}] + [\text{hC4bCR1}]}\cdots$$

$$\left.\frac{1}{+[\text{hC4bC4BP}] + [\text{hC4bC4bCR1}] + [\text{hC4bC4bC4BP}] + [\text{hC4bC4dCR1}] + [\text{hC4bC4dC4BP}]}\cdots\right)$$

S131

$$\frac{d[\text{IgGC3biC3bCR1}]}{dt} = k^{+}_{\text{C3biC3bCR1}}[\text{IgGC3biC3b}][\text{CR1}] - k^{-}_{\text{C3biC3bCR1}}[\text{IgGC3biC3bCR1}]$$

$$-\left(\frac{k^{\text{FI}}_{\text{catC3bH}}[\text{I}][\text{IgGC3biC3bCR1}]}{K^{\text{FI}}_{\text{mC3bH}} + [\text{C3(H}_2\text{O)H}] + [\text{C3(H}_2\text{O)HL}] + [\text{fC3bH}] + [\text{fC3bHL}] + [\text{fC3bCR1}] + [\text{fiC3bCR1}] + [\text{IgGC3bC3bH}] + [\text{IgGC3biC3bH}] + [\text{IgGC3bC3bHL}]}\cdots\right.$$

$$\frac{1}{+[\text{IgGC3biC3bHL}] + [\text{IgGC3bC3bCR1}] + [\text{IgGC3biC3bCR1}] + [\text{IgGiC3biC3bCR1}] + [\text{IgGiC3bC3dgCR1}] + [\text{fC3bC4bH}] + [\text{fC3bC4bHL}]}\cdots$$

$$\frac{1}{+[\text{fC3bC4bCR1}] + [\text{fC3bC4bC4BP}] + [\text{fC3bC4dH}] + [\text{fC3bC4dHL}] + [\text{fC3bC4dCR1}] + [\text{fiC3bC4dCR1}] + [\text{fiC3bC4bCR1}] + [\text{fiC3bC4bC4BP}]}\cdots$$

$$\frac{1}{+[\text{fC3dgC4bCR1}] + [\text{fC3dgC4bC4BP}] + [\text{fC3bC3bH}] + [\text{fC3bC3bHL}] + [\text{fC3bC3bCR1}] + [\text{fC3biC3bH}] + [\text{fC3biC3bHL}] + [\text{fC3biC3bCR1}]}\cdots$$

$$\frac{1}{+[\text{fiC3biC3bCR1}] + [\text{fiC3bC3dgCR1}] + [\text{hC3bH}] + [\text{hC3bHL}] + [\text{hC3bCR1}] + [\text{hiC3bCR1}] + [\text{hC3bC4bH}] + [\text{hC3bC4bHL}] + [\text{hC3bC4bCR1}]}\cdots$$

$$\frac{1}{+[\text{hC3bC4bC4BP}] + [\text{hC3bC4dH}] + [\text{hC3bC4dHL}] + [\text{hC3bC4dCR1}] + [\text{hiC3bC4dCR1}] + [\text{hiC3bC4bCR1}] + [\text{hiC3bC4bC4BP}] + [\text{hC3dgC4bCR1}]}\cdots$$

$$\frac{1}{+[\text{hC3dgC4bC4BP}] + [\text{hC3bC3bH}] + [\text{hC3bC3bHL}] + [\text{hC3bC3bCR1}] + [\text{hC3biC3bH}] + [\text{hC3biC3bHL}] + [\text{hC3biC3bCR1}] + [\text{hiC3biC3bCR1}]}\cdots$$

$$\frac{1}{+[\text{hiC3bC3dgCR1}] + [\text{fC4bCR1}] + [\text{nfC4bC4BP}] + [\text{fC4bC4BP}] + [\text{fC4bC4bCR1}] + [\text{fC4bC4bC4BP}] + [\text{fC4bC4dCR1}] + [\text{fC4bC4dC4BP}] + [\text{hC4bCR1}]}\cdots$$

$$\left.\frac{1}{+[\text{hC4bC4BP}] + [\text{hC4bC4bCR1}] + [\text{hC4bC4bC4BP}] + [\text{hC4bC4dCR1}] + [\text{hC4bC4dC4BP}]}\cdots\right)$$

S132

$$\frac{d[\mathrm{IgGiC3biC3b}]}{dt} =$$

$$\left(\frac{k_{\mathrm{catC3bH}}^{\mathrm{FI}}[\mathrm{I}][\mathrm{IgGC3biC3bH}]}{K_{\mathrm{mC3bH}}^{\mathrm{FI}} + [\mathrm{C3(H_2O)H}] + [\mathrm{C3(H_2O)HL}] + [\mathrm{fC3bH}] + [\mathrm{fC3bHL}] + [\mathrm{fC3bCR1}] + [\mathrm{fiC3bCR1}] + [\mathrm{IgGC3bC3bH}] + [\mathrm{IgGC3biC3bH}] + [\mathrm{IgGC3bC3bHL}]} \cdots \right.$$

$$\frac{1}{+ [\mathrm{IgGC3biC3bHL}] + [\mathrm{IgGC3bC3bCR1}] + [\mathrm{IgGC3biC3bCR1}] + [\mathrm{IgGiC3biC3bCR1}] + [\mathrm{IgGiC3bC3dgCR1}] + [\mathrm{fC3bC4bH}] + [\mathrm{fC3bC4bHL}]} \cdots$$

$$\frac{1}{+ [\mathrm{fC3bC4bCR1}] + [\mathrm{fC3bC4bC4BP}] + [\mathrm{fC3bC4dH}] + [\mathrm{fC3bC4dHL}] + [\mathrm{fC3bC4dCR1}] + [\mathrm{fiC3bC4dCR1}] + [\mathrm{fiC3bC4bCR1}] + [\mathrm{fiC3bC4bC4BP}]} \cdots$$

$$\frac{1}{+ [\mathrm{fC3dgC4bCR1}] + [\mathrm{fC3dgC4bC4BP}] + [\mathrm{fC3bC3bH}] + [\mathrm{fC3bC3bHL}] + [\mathrm{fC3bC3bCR1}] + [\mathrm{fC3biC3bH}] + [\mathrm{fC3biC3bHL}] + [\mathrm{fC3biC3bCR1}]} \cdots$$

$$\frac{1}{+ [\mathrm{fiC3biC3bCR1}] + [\mathrm{fiC3bC3dgCR1}] + [\mathrm{hC3bH}] + [\mathrm{hC3bHL}] + [\mathrm{hC3bCR1}] + [\mathrm{hiC3bCR1}] + [\mathrm{hC3bC4bH}] + [\mathrm{hC3bC4bHL}] + [\mathrm{hC3bC4bCR1}]} \cdots$$

$$\frac{1}{+ [\mathrm{hC3bC4bC4BP}] + [\mathrm{hC3bC4dH}] + [\mathrm{hC3bC4dHL}] + [\mathrm{hC3bC4dCR1}] + [\mathrm{hiC3bC4dCR1}] + [\mathrm{hiC3bC4bCR1}] + [\mathrm{hiC3bC4bC4BP}] + [\mathrm{hC3dgC4bCR1}]} \cdots$$

$$\frac{1}{+ [\mathrm{hC3dgC4bC4BP}] + [\mathrm{hC3bC3bH}] + [\mathrm{hC3bC3bHL}] + [\mathrm{hC3bC3bCR1}] + [\mathrm{hC3biC3bH}] + [\mathrm{hC3biC3bHL}] + [\mathrm{hC3biC3bCR1}] + [\mathrm{hiC3biC3bCR1}]} \cdots$$

$$\frac{1}{+ [\mathrm{hiC3bC3dgCR1}] + [\mathrm{fC4bCR1}] + [\mathrm{nfC4bC4BP}] + [\mathrm{fC4bC4BP}] + [\mathrm{fC4bC4bCR1}] + [\mathrm{fC4bC4bC4BP}] + [\mathrm{fC4bC4dCR1}] + [\mathrm{fC4bC4dC4BP}] + [\mathrm{hC4bCR1}]} \cdots$$

$$\left. \frac{1}{+ [\mathrm{hC4bC4BP}] + [\mathrm{hC4bC4bCR1}] + [\mathrm{hC4bC4bC4BP}] + [\mathrm{hC4bC4dCR1}] + [\mathrm{hC4bC4dC4BP}]} \cdots \right)$$

$$+ \left(\frac{k_{\mathrm{catC3bH}}^{\mathrm{FI}}[\mathrm{I}][\mathrm{IgGC3biC3bHL}]}{K_{\mathrm{mC3bH}}^{\mathrm{FI}} + [\mathrm{C3(H_2O)H}] + [\mathrm{C3(H_2O)HL}] + [\mathrm{fC3bH}] + [\mathrm{fC3bHL}] + [\mathrm{fC3bCR1}] + [\mathrm{fiC3bCR1}] + [\mathrm{IgGC3bC3bH}] + [\mathrm{IgGC3biC3bH}] + [\mathrm{IgGC3bC3bHL}]} \cdots \right.$$

$$\frac{1}{+ [\mathrm{IgGC3biC3bHL}] + [\mathrm{IgGC3bC3bCR1}] + [\mathrm{IgGC3biC3bCR1}] + [\mathrm{IgGiC3biC3bCR1}] + [\mathrm{IgGiC3bC3dgCR1}] + [\mathrm{fC3bC4bH}] + [\mathrm{fC3bC4bHL}]} \cdots$$

$$\frac{1}{+ [\mathrm{fC3bC4bCR1}] + [\mathrm{fC3bC4bC4BP}] + [\mathrm{fC3bC4dH}] + [\mathrm{fC3bC4dHL}] + [\mathrm{fC3bC4dCR1}] + [\mathrm{fiC3bC4dCR1}] + [\mathrm{fiC3bC4bCR1}] + [\mathrm{fiC3bC4bC4BP}]} \cdots$$

$$\frac{1}{+ [\mathrm{fC3dgC4bCR1}] + [\mathrm{fC3dgC4bC4BP}] + [\mathrm{fC3bC3bH}] + [\mathrm{fC3bC3bHL}] + [\mathrm{fC3bC3bCR1}] + [\mathrm{fC3biC3bH}] + [\mathrm{fC3biC3bHL}] + [\mathrm{fC3biC3bCR1}]} \cdots$$

$$\frac{1}{+ [\mathrm{fiC3biC3bCR1}] + [\mathrm{fiC3bC3dgCR1}] + [\mathrm{hC3bH}] + [\mathrm{hC3bHL}] + [\mathrm{hC3bCR1}] + [\mathrm{hiC3bCR1}] + [\mathrm{hC3bC4bH}] + [\mathrm{hC3bC4bHL}] + [\mathrm{hC3bC4bCR1}]} \cdots$$

$$\frac{1}{+ [\mathrm{hC3bC4bC4BP}] + [\mathrm{hC3bC4dH}] + [\mathrm{hC3bC4dHL}] + [\mathrm{hC3bC4dCR1}] + [\mathrm{hiC3bC4dCR1}] + [\mathrm{hiC3bC4bCR1}] + [\mathrm{hiC3bC4bC4BP}] + [\mathrm{hC3dgC4bCR1}]} \cdots$$

$$\frac{1}{+ [\mathrm{hC3dgC4bC4BP}] + [\mathrm{hC3bC3bH}] + [\mathrm{hC3bC3bHL}] + [\mathrm{hC3bC3bCR1}] + [\mathrm{hC3biC3bH}] + [\mathrm{hC3biC3bHL}] + [\mathrm{hC3biC3bCR1}] + [\mathrm{hiC3biC3bCR1}]} \cdots$$

$$\frac{1}{+ [\mathrm{hiC3bC3dgCR1}] + [\mathrm{fC4bCR1}] + [\mathrm{nfC4bC4BP}] + [\mathrm{fC4bC4BP}] + [\mathrm{fC4bC4bCR1}] + [\mathrm{fC4bC4bC4BP}] + [\mathrm{fC4bC4dCR1}] + [\mathrm{fC4bC4dC4BP}] + [\mathrm{hC4bCR1}]} \cdots$$

$$\left. \frac{1}{+ [\mathrm{hC4bC4BP}] + [\mathrm{hC4bC4bCR1}] + [\mathrm{hC4bC4bC4BP}] + [\mathrm{hC4bC4dCR1}] + [\mathrm{hC4bC4dC4BP}]} \cdots \right)$$

$$+\left(\begin{array}{l}\frac{k_{\mathrm{catC3bH}}^{\mathrm{FI}}[\mathrm{I}][\mathrm{IgGC3biC3bCR1}]}{K_{\mathrm{mC3bH}}^{\mathrm{FI}}+[\mathrm{C3(H_2O)H}]+[\mathrm{C3(H_2O)HL}]+[\mathrm{fC3bH}]+[\mathrm{fC3bHL}]+[\mathrm{fC3bCR1}]+[\mathrm{fiC3bCR1}]+[\mathrm{IgGC3bC3bH}]+[\mathrm{IgGC3biC3bH}]+[\mathrm{IgGC3bC3bHL}]}\cdots \\ \frac{1}{+[\mathrm{IgGC3biC3bHL}]+[\mathrm{IgGC3bC3bCR1}]+[\mathrm{IgGC3biC3bCR1}]+[\mathrm{IgGiC3biC3bCR1}]+[\mathrm{IgGiC3bC3dgCR1}]+[\mathrm{fC3bC4bH}]+[\mathrm{fC3bC4bHL}]}\cdots \\ \frac{1}{+[\mathrm{fC3bC4bCR1}]+[\mathrm{fC3bC4bC4BP}]+[\mathrm{fC3bC4dH}]+[\mathrm{fC3bC4dHL}]+[\mathrm{fC3bC4dCR1}]+[\mathrm{fiC3bC4dCR1}]+[\mathrm{fiC3bC4bCR1}]+[\mathrm{fiC3bC4bC4BP}]}\cdots \\ \frac{1}{+[\mathrm{fC3dgC4bCR1}]+[\mathrm{fC3dgC4bC4BP}]+[\mathrm{fC3bC3bH}]+[\mathrm{fC3bC3bHL}]+[\mathrm{fC3bC3bCR1}]+[\mathrm{fC3biC3bH}]+[\mathrm{fC3biC3bHL}]+[\mathrm{fC3biC3bCR1}]}\cdots \\ \frac{1}{+[\mathrm{fiC3biC3bCR1}]+[\mathrm{fiC3bC3dgCR1}]+[\mathrm{hC3bH}]+[\mathrm{hC3bHL}]+[\mathrm{hC3bCR1}]+[\mathrm{hiC3bCR1}]+[\mathrm{hC3bC4bH}]+[\mathrm{hC3bC4bHL}]+[\mathrm{hC3bC4bCR1}]}\cdots \\ \frac{1}{+[\mathrm{hC3bC4bC4BP}]+[\mathrm{hC3bC4dH}]+[\mathrm{hC3bC4dHL}]+[\mathrm{hC3bC4dCR1}]+[\mathrm{hiC3bC4dCR1}]+[\mathrm{hiC3bC4bCR1}]+[\mathrm{hiC3bC4bC4BP}]+[\mathrm{hC3dgC4bCR1}]}\cdots \\ \frac{1}{+[\mathrm{hC3dgC4bC4BP}]+[\mathrm{hC3bC3bH}]+[\mathrm{hC3bC3bHL}]+[\mathrm{hC3bC3bCR1}]+[\mathrm{hC3biC3bH}]+[\mathrm{hC3biC3bHL}]+[\mathrm{hC3biC3bCR1}]+[\mathrm{hiC3biC3bCR1}]}\cdots \\ \frac{1}{+[\mathrm{hiC3bC3dgCR1}]+[\mathrm{fC4bCR1}]+[\mathrm{nfC4bC4BP}]+[\mathrm{fC4bC4BP}]+[\mathrm{fC4bC4bCR1}]+[\mathrm{fC4bC4bC4BP}]+[\mathrm{fC4bC4dCR1}]+[\mathrm{fC4bC4dC4BP}]+[\mathrm{hC4bCR1}]}\cdots \\ \frac{1}{+[\mathrm{hC4bC4BP}]+[\mathrm{hC4bC4bCR1}]+[\mathrm{hC4bC4bC4BP}]+[\mathrm{hC4bC4dCR1}]+[\mathrm{hC4bC4dC4BP}]}\cdots\end{array}\right)$$

$$-k_{\mathrm{iC3biC3bCR1}}^{+}[\mathrm{IgGiC3biC3b}][\mathrm{CR1}]+k_{\mathrm{iC3biC3bCR1}}^{-}[\mathrm{IgGiC3biC3bCR1}]$$

S133

$$\frac{d[\mathrm{IgGiC3biC3bCR1}]}{dt}=k_{\mathrm{iC3biC3bCR1}}^{+}[\mathrm{IgGiC3biC3b}][\mathrm{CR1}]-k_{\mathrm{iC3biC3bCR1}}^{-}[\mathrm{IgGiC3biC3bCR1}]$$

$$-\left(\begin{array}{l}\frac{k_{\mathrm{catC3bH}}^{\mathrm{FI}}[\mathrm{I}][\mathrm{IgGiC3biC3bCR1}]}{K_{\mathrm{mC3bH}}^{\mathrm{FI}}+[\mathrm{C3(H_2O)H}]+[\mathrm{C3(H_2O)HL}]+[\mathrm{fC3bH}]+[\mathrm{fC3bHL}]+[\mathrm{fC3bCR1}]+[\mathrm{fiC3bCR1}]+[\mathrm{IgGC3bC3bH}]+[\mathrm{IgGC3biC3bH}]+[\mathrm{IgGC3bC3bHL}]}\cdots \\ \frac{1}{+[\mathrm{IgGC3biC3bHL}]+[\mathrm{IgGC3bC3bCR1}]+[\mathrm{IgGC3biC3bCR1}]+[\mathrm{IgGiC3biC3bCR1}]+[\mathrm{IgGiC3bC3dgCR1}]+[\mathrm{fC3bC4bH}]+[\mathrm{fC3bC4bHL}]}\cdots \\ \frac{1}{+[\mathrm{fC3bC4bCR1}]+[\mathrm{fC3bC4bC4BP}]+[\mathrm{fC3bC4dH}]+[\mathrm{fC3bC4dHL}]+[\mathrm{fC3bC4dCR1}]+[\mathrm{fiC3bC4dCR1}]+[\mathrm{fiC3bC4bCR1}]+[\mathrm{fiC3bC4bC4BP}]}\cdots \\ \frac{1}{+[\mathrm{fC3dgC4bCR1}]+[\mathrm{fC3dgC4bC4BP}]+[\mathrm{fC3bC3bH}]+[\mathrm{fC3bC3bHL}]+[\mathrm{fC3bC3bCR1}]+[\mathrm{fC3biC3bH}]+[\mathrm{fC3biC3bHL}]+[\mathrm{fC3biC3bCR1}]}\cdots \\ \frac{1}{+[\mathrm{fiC3biC3bCR1}]+[\mathrm{fiC3bC3dgCR1}]+[\mathrm{hC3bH}]+[\mathrm{hC3bHL}]+[\mathrm{hC3bCR1}]+[\mathrm{hiC3bCR1}]+[\mathrm{hC3bC4bH}]+[\mathrm{hC3bC4bHL}]+[\mathrm{hC3bC4bCR1}]}\cdots \\ \frac{1}{+[\mathrm{hC3bC4bC4BP}]+[\mathrm{hC3bC4dH}]+[\mathrm{hC3bC4dHL}]+[\mathrm{hC3bC4dCR1}]+[\mathrm{hiC3bC4dCR1}]+[\mathrm{hiC3bC4bCR1}]+[\mathrm{hiC3bC4bC4BP}]+[\mathrm{hC3dgC4bCR1}]}\cdots \\ \frac{1}{+[\mathrm{hC3dgC4bC4BP}]+[\mathrm{hC3bC3bH}]+[\mathrm{hC3bC3bHL}]+[\mathrm{hC3bC3bCR1}]+[\mathrm{hC3biC3bH}]+[\mathrm{hC3biC3bHL}]+[\mathrm{hC3biC3bCR1}]+[\mathrm{hiC3biC3bCR1}]}\cdots \\ \frac{1}{+[\mathrm{hiC3bC3dgCR1}]+[\mathrm{fC4bCR1}]+[\mathrm{nfC4bC4BP}]+[\mathrm{fC4bC4BP}]+[\mathrm{fC4bC4bCR1}]+[\mathrm{fC4bC4bC4BP}]+[\mathrm{fC4bC4dCR1}]+[\mathrm{fC4bC4dC4BP}]+[\mathrm{hC4bCR1}]}\cdots \\ \frac{1}{+[\mathrm{hC4bC4BP}]+[\mathrm{hC4bC4bCR1}]+[\mathrm{hC4bC4bC4BP}]+[\mathrm{hC4bC4dCR1}]+[\mathrm{hC4bC4dC4BP}]}\cdots\end{array}\right)$$

S134

$$\frac{d[\mathrm{IgGiC3bC3dg}]}{dt} =$$

$$\left(\frac{k^{\mathrm{FI}}_{\mathrm{catC3bH}}[\mathrm{I}][\mathrm{IgGiC3biC3bCR1}]}{K^{\mathrm{FI}}_{\mathrm{mC3bH}} + [\mathrm{C3(H_2O)H}] + [\mathrm{C3(H_2O)HL}] + [\mathrm{fC3bH}] + [\mathrm{fC3bHL}] + [\mathrm{fC3bCR1}] + [\mathrm{fiC3bCR1}] + [\mathrm{IgGC3bC3bH}] + [\mathrm{IgGC3biC3bH}] + [\mathrm{IgGC3bC3bHL}] \ldots} \right.$$

$$\frac{1}{+[\mathrm{IgGC3biC3bHL}] + [\mathrm{IgGC3bC3bCR1}] + [\mathrm{IgGC3biC3bCR1}] + [\mathrm{IgGiC3biC3bCR1}] + [\mathrm{IgGiC3bC3dgCR1}] + [\mathrm{fC3bC4bH}] + [\mathrm{fC3bC4bHL}] \ldots}$$

$$\frac{1}{+[\mathrm{fC3bC4bCR1}] + [\mathrm{fC3bC4bC4BP}] + [\mathrm{fC3bC4dH}] + [\mathrm{fC3bC4dHL}] + [\mathrm{fC3bC4dCR1}] + [\mathrm{fiC3bC4dCR1}] + [\mathrm{fiC3bC4bCR1}] + [\mathrm{fiC3bC4bC4BP}] \ldots}$$

$$\frac{1}{+[\mathrm{fC3dgC4bCR1}] + [\mathrm{fC3dgC4bC4BP}] + [\mathrm{fC3bC3bH}] + [\mathrm{fC3bC3bHL}] + [\mathrm{fC3bC3bCR1}] + [\mathrm{fC3biC3bH}] + [\mathrm{fC3biC3bHL}] + [\mathrm{fC3biC3bCR1}] \ldots}$$

$$\frac{1}{+[\mathrm{fiC3biC3bCR1}] + [\mathrm{fiC3bC3dgCR1}] + [\mathrm{hC3bH}] + [\mathrm{hC3bHL}] + [\mathrm{hC3bCR1}] + [\mathrm{hiC3bCR1}] + [\mathrm{hC3bC4bH}] + [\mathrm{hC3bC4bHL}] + [\mathrm{hC3bC4bCR1}] \ldots}$$

$$\frac{1}{+[\mathrm{hC3bC4bC4BP}] + [\mathrm{hC3bC4dH}] + [\mathrm{hC3bC4dHL}] + [\mathrm{hC3bC4dCR1}] + [\mathrm{hiC3bC4dCR1}] + [\mathrm{hiC3bC4bCR1}] + [\mathrm{hiC3bC4bC4BP}] + [\mathrm{hC3dgC4bCR1}] \ldots}$$

$$\frac{1}{+[\mathrm{hC3dgC4bC4BP}] + [\mathrm{hC3bC3bH}] + [\mathrm{hC3bC3bHL}] + [\mathrm{hC3bC3bCR1}] + [\mathrm{hC3biC3bH}] + [\mathrm{hC3biC3bHL}] + [\mathrm{hC3biC3bCR1}] + [\mathrm{hiC3biC3bCR1}] \ldots}$$

$$\frac{1}{+[\mathrm{hiC3bC3dgCR1}] + [\mathrm{fC4bCR1}] + [\mathrm{nfC4bC4BP}] + [\mathrm{fC4bC4BP}] + [\mathrm{fC4bC4bCR1}] + [\mathrm{fC4bC4bC4BP}] + [\mathrm{fC4bC4dCR1}] + [\mathrm{fC4bC4dC4BP}] + [\mathrm{hC4bCR1}] \ldots}$$

$$\left. \frac{1}{+[\mathrm{hC4bC4BP}] + [\mathrm{hC4bC4bCR1}] + [\mathrm{hC4bC4bC4BP}] + [\mathrm{hC4bC4dCR1}] + [\mathrm{hC4bC4dC4BP}] \ldots} \right)$$

$$- k^{+}_{\mathrm{iC3bC3dgCR1}}[\mathrm{IgGiC3bC3dg}][\mathrm{CR1}] + k^{-}_{\mathrm{iC3bC3dgCR1}}[\mathrm{IgGiC3bC3dgCR1}]$$

S135

$$\frac{d[\mathrm{IgGiC3bC3dgCR1}]}{dt} = k^{+}_{\mathrm{iC3bC3dgCR1}}[\mathrm{IgGiC3bC3dg}][\mathrm{CR1}] - k^{-}_{\mathrm{iC3bC3dgCR1}}[\mathrm{IgGiC3bC3dgCR1}]$$

$$- \left(\frac{k^{\mathrm{FI}}_{\mathrm{catC3bH}}[\mathrm{I}][\mathrm{IgGiC3bC3dgCR1}]}{K^{\mathrm{FI}}_{\mathrm{mC3bH}} + [\mathrm{C3(H_2O)H}] + [\mathrm{C3(H_2O)HL}] + [\mathrm{fC3bH}] + [\mathrm{fC3bHL}] + [\mathrm{fC3bCR1}] + [\mathrm{fiC3bCR1}] + [\mathrm{IgGC3bC3bH}] + [\mathrm{IgGC3biC3bH}] + [\mathrm{IgGC3bC3bHL}] \ldots} \right.$$

$$\frac{1}{+[\mathrm{IgGC3biC3bHL}] + [\mathrm{IgGC3bC3bCR1}] + [\mathrm{IgGC3biC3bCR1}] + [\mathrm{IgGiC3biC3bCR1}] + [\mathrm{IgGiC3bC3dgCR1}] + [\mathrm{fC3bC4bH}] + [\mathrm{fC3bC4bHL}] \ldots}$$

$$\frac{1}{+[\mathrm{fC3bC4bCR1}] + [\mathrm{fC3bC4bC4BP}] + [\mathrm{fC3bC4dH}] + [\mathrm{fC3bC4dHL}] + [\mathrm{fC3bC4dCR1}] + [\mathrm{fiC3bC4dCR1}] + [\mathrm{fiC3bC4bCR1}] + [\mathrm{fiC3bC4bC4BP}] \ldots}$$

$$\frac{1}{+[\mathrm{fC3dgC4bCR1}] + [\mathrm{fC3dgC4bC4BP}] + [\mathrm{fC3bC3bH}] + [\mathrm{fC3bC3bHL}] + [\mathrm{fC3bC3bCR1}] + [\mathrm{fC3biC3bH}] + [\mathrm{fC3biC3bHL}] + [\mathrm{fC3biC3bCR1}] \ldots}$$

$$\frac{1}{+[\mathrm{fiC3biC3bCR1}] + [\mathrm{fiC3bC3dgCR1}] + [\mathrm{hC3bH}] + [\mathrm{hC3bHL}] + [\mathrm{hC3bCR1}] + [\mathrm{hiC3bCR1}] + [\mathrm{hC3bC4bH}] + [\mathrm{hC3bC4bHL}] + [\mathrm{hC3bC4bCR1}] \ldots}$$

$$\frac{1}{+[\mathrm{hC3bC4bC4BP}] + [\mathrm{hC3bC4dH}] + [\mathrm{hC3bC4dHL}] + [\mathrm{hC3bC4dCR1}] + [\mathrm{hiC3bC4dCR1}] + [\mathrm{hiC3bC4bCR1}] + [\mathrm{hiC3bC4bC4BP}] + [\mathrm{hC3dgC4bCR1}] \ldots}$$

$$\frac{1}{+[\mathrm{hC3dgC4bC4BP}] + [\mathrm{hC3bC3bH}] + [\mathrm{hC3bC3bHL}] + [\mathrm{hC3bC3bCR1}] + [\mathrm{hC3biC3bH}] + [\mathrm{hC3biC3bHL}] + [\mathrm{hC3biC3bCR1}] + [\mathrm{hiC3biC3bCR1}] \ldots}$$

$$\frac{1}{+[\mathrm{hiC3bC3dgCR1}] + [\mathrm{fC4bCR1}] + [\mathrm{nfC4bC4BP}] + [\mathrm{fC4bC4BP}] + [\mathrm{fC4bC4bCR1}] + [\mathrm{fC4bC4bC4BP}] + [\mathrm{fC4bC4dCR1}] + [\mathrm{fC4bC4dC4BP}] + [\mathrm{hC4bCR1}] \ldots}$$

$$\left. \frac{1}{+[\mathrm{hC4bC4BP}] + [\mathrm{hC4bC4bCR1}] + [\mathrm{hC4bC4bC4BP}] + [\mathrm{hC4bC4dCR1}] + [\mathrm{hC4bC4dC4BP}] \ldots} \right)$$

S136

$$\frac{d[\text{IgGC3dgC3dg}]}{dt} =$$

$$\left(\frac{k_{\text{catC3bH}}^{\text{FI}}[\text{I}][\text{IgGiC3bC3dgCR1}]}{K_{\text{mC3bH}}^{\text{FI}} + [\text{C3(H}_2\text{O)H}] + [\text{C3(H}_2\text{O)HL}] + [\text{fC3bH}] + [\text{fC3bHL}] + [\text{fC3bCR1}] + [\text{fiC3bCR1}] + [\text{IgGC3bC3bH}] + [\text{IgGC3biC3bH}] + [\text{IgGC3bC3bHL}]} \cdots \right.$$

$$\frac{1}{+[\text{IgGC3biC3bHL}] + [\text{IgGC3bC3bCR1}] + [\text{IgGC3biC3bCR1}] + [\text{IgGiC3biC3bCR1}] + [\text{IgGiC3bC3dgCR1}] + [\text{fC3bC4bH}] + [\text{fC3bC4bHL}]} \cdots$$

$$\frac{1}{+[\text{fC3bC4bCR1}] + [\text{fC3bC4bC4BP}] + [\text{fC3bC4dH}] + [\text{fC3bC4dHL}] + [\text{fC3bC4dCR1}] + [\text{fiC3bC4dCR1}] + [\text{fiC3bC4bCR1}] + [\text{fiC3bC4bC4BP}]} \cdots$$

$$\frac{1}{+[\text{fC3dgC4bCR1}] + [\text{fC3dgC4bC4BP}] + [\text{fC3bC3bH}] + [\text{fC3bC3bHL}] + [\text{fC3bC3bCR1}] + [\text{fC3biC3bH}] + [\text{fC3biC3bHL}] + [\text{fC3biC3bCR1}]} \cdots$$

$$\frac{1}{+[\text{fiC3biC3bCR1}] + [\text{fiC3bC3dgCR1}] + [\text{hC3bH}] + [\text{hC3bHL}] + [\text{hC3bCR1}] + [\text{hiC3bCR1}] + [\text{hC3bC4bH}] + [\text{hC3bC4bHL}] + [\text{hC3bC4bCR1}]} \cdots$$

$$\frac{1}{+[\text{hC3bC4bC4BP}] + [\text{hC3bC4dH}] + [\text{hC3bC4dHL}] + [\text{hC3bC4dCR1}] + [\text{hiC3bC4dCR1}] + [\text{hiC3bC4bCR1}] + [\text{hiC3bC4bC4BP}] + [\text{hC3dgC4bCR1}]} \cdots$$

$$\frac{1}{+[\text{hC3dgC4bC4BP}] + [\text{hC3bC3bH}] + [\text{hC3bC3bHL}] + [\text{hC3bC3bCR1}] + [\text{hC3biC3bH}] + [\text{hC3biC3bHL}] + [\text{hC3biC3bCR1}] + [\text{hiC3biC3bCR1}]} \cdots$$

$$\frac{1}{+[\text{hiC3bC3dgCR1}] + [\text{fC4bCR1}] + [\text{nfC4bC4BP}] + [\text{fC4bC4BP}] + [\text{fC4bC4bCR1}] + [\text{fC4bC4bC4BP}] + [\text{fC4bC4dCR1}] + [\text{fC4bC4dC4BP}] + [\text{hC4bCR1}]} \cdots$$

$$\left. \frac{1}{+[\text{hC4bC4BP}] + [\text{hC4bC4bCR1}] + [\text{hC4bC4bC4BP}] + [\text{hC4bC4dCR1}] + [\text{hC4bC4dC4BP}]} \cdots \right)$$

S137

$$\frac{d[\text{fC3bC4bH}]}{dt} = k_{\text{C3bH}}^{+}[\text{fC3bC4b}][\text{H}] - k_{\text{C3bH}}^{-}[\text{fC3bC4bH}]$$

$$-\left(\frac{k_{\text{catC3bH}}^{\text{FI}}[\text{I}][\text{fC3bC4bH}]}{K_{\text{mC3bH}}^{\text{FI}} + [\text{C3(H}_2\text{O)H}] + [\text{C3(H}_2\text{O)HL}] + [\text{fC3bH}] + [\text{fC3bHL}] + [\text{fC3bCR1}] + [\text{fiC3bCR1}] + [\text{IgGC3bC3bH}] + [\text{IgGC3biC3bH}] + [\text{IgGC3bC3bHL}]} \cdots \right.$$

$$\frac{1}{+[\text{IgGC3biC3bHL}] + [\text{IgGC3bC3bCR1}] + [\text{IgGC3biC3bCR1}] + [\text{IgGiC3biC3bCR1}] + [\text{IgGiC3bC3dgCR1}] + [\text{fC3bC4bH}] + [\text{fC3bC4bHL}]} \cdots$$

$$\frac{1}{+[\text{fC3bC4bCR1}] + [\text{fC3bC4bC4BP}] + [\text{fC3bC4dH}] + [\text{fC3bC4dHL}] + [\text{fC3bC4dCR1}] + [\text{fiC3bC4dCR1}] + [\text{fiC3bC4bCR1}] + [\text{fiC3bC4bC4BP}]} \cdots$$

$$\frac{1}{+[\text{fC3dgC4bCR1}] + [\text{fC3dgC4bC4BP}] + [\text{fC3bC3bH}] + [\text{fC3bC3bHL}] + [\text{fC3bC3bCR1}] + [\text{fC3biC3bH}] + [\text{fC3biC3bHL}] + [\text{fC3biC3bCR1}]} \cdots$$

$$\frac{1}{+[\text{fiC3biC3bCR1}] + [\text{fiC3bC3dgCR1}] + [\text{hC3bH}] + [\text{hC3bHL}] + [\text{hC3bCR1}] + [\text{hiC3bCR1}] + [\text{hC3bC4bH}] + [\text{hC3bC4bHL}] + [\text{hC3bC4bCR1}]} \cdots$$

$$\frac{1}{+[\text{hC3bC4bC4BP}] + [\text{hC3bC4dH}] + [\text{hC3bC4dHL}] + [\text{hC3bC4dCR1}] + [\text{hiC3bC4dCR1}] + [\text{hiC3bC4bCR1}] + [\text{hiC3bC4bC4BP}] + [\text{hC3dgC4bCR1}]} \cdots$$

$$\frac{1}{+[\text{hC3dgC4bC4BP}] + [\text{hC3bC3bH}] + [\text{hC3bC3bHL}] + [\text{hC3bC3bCR1}] + [\text{hC3biC3bH}] + [\text{hC3biC3bHL}] + [\text{hC3biC3bCR1}] + [\text{hiC3biC3bCR1}]} \cdots$$

$$\frac{1}{+[\text{hiC3bC3dgCR1}] + [\text{fC4bCR1}] + [\text{nfC4bC4BP}] + [\text{fC4bC4BP}] + [\text{fC4bC4bCR1}] + [\text{fC4bC4bC4BP}] + [\text{fC4bC4dCR1}] + [\text{fC4bC4dC4BP}] + [\text{hC4bCR1}]} \cdots$$

$$\left. \frac{1}{+[\text{hC4bC4BP}] + [\text{hC4bC4bCR1}] + [\text{hC4bC4bC4BP}] + [\text{hC4bC4dCR1}] + [\text{hC4bC4dC4BP}]} \cdots \right)$$

S138

$$\frac{d[\mathrm{fC3bC4bHL}]}{dt} = k^{+}_{\mathrm{C3bHL}}[\mathrm{fC3bC4b}][\mathrm{HL}] - k^{-}_{\mathrm{C3bHL}}[\mathrm{fC3bC4bHL}]$$

$$-\left(\frac{k^{\mathrm{FI}}_{\mathrm{catC3bH}}[\mathrm{I}][\mathrm{fC3bC4bHL}]}{K^{\mathrm{FI}}_{\mathrm{mC3bH}} + [\mathrm{C3(H_2O)H}] + [\mathrm{C3(H_2O)HL}] + [\mathrm{fC3bH}] + [\mathrm{fC3bHL}] + [\mathrm{fC3bCR1}] + [\mathrm{fiC3bCR1}] + [\mathrm{IgGC3bC3bH}] + [\mathrm{IgGC3biC3bH}] + [\mathrm{IgGC3bC3bHL}]} \cdots \right.$$

$$\frac{1}{+ [\mathrm{IgGC3biC3bHL}] + [\mathrm{IgGC3bC3bCR1}] + [\mathrm{IgGC3biC3bCR1}] + [\mathrm{IgGiC3biC3bCR1}] + [\mathrm{IgGiC3bC3dgCR1}] + [\mathrm{fC3bC4bH}] + [\mathrm{fC3bC4bHL}]} \cdots$$

$$\frac{1}{+ [\mathrm{fC3bC4bCR1}] + [\mathrm{fC3bC4bC4BP}] + [\mathrm{fC3bC4dH}] + [\mathrm{fC3bC4dHL}] + [\mathrm{fC3bC4dCR1}] + [\mathrm{fiC3bC4dCR1}] + [\mathrm{fiC3bC4bCR1}] + [\mathrm{fiC3bC4bC4BP}]} \cdots$$

$$\frac{1}{+ [\mathrm{fC3dgC4bCR1}] + [\mathrm{fC3dgC4bC4BP}] + [\mathrm{fC3bC3bH}] + [\mathrm{fC3bC3bHL}] + [\mathrm{fC3bC3bCR1}] + [\mathrm{fC3biC3bH}] + [\mathrm{fC3biC3bHL}] + [\mathrm{fC3biC3bCR1}]} \cdots$$

$$\frac{1}{+ [\mathrm{fiC3biC3bCR1}] + [\mathrm{fiC3bC3dgCR1}] + [\mathrm{hC3bH}] + [\mathrm{hC3bHL}] + [\mathrm{hC3bCR1}] + [\mathrm{hiC3bCR1}] + [\mathrm{hC3bC4bH}] + [\mathrm{hC3bC4bHL}] + [\mathrm{hC3bC4bCR1}]} \cdots$$

$$\frac{1}{+ [\mathrm{hC3bC4bC4BP}] + [\mathrm{hC3bC4dH}] + [\mathrm{hC3bC4dHL}] + [\mathrm{hC3bC4dCR1}] + [\mathrm{hiC3bC4dCR1}] + [\mathrm{hiC3bC4bCR1}] + [\mathrm{hiC3bC4bC4BP}] + [\mathrm{hC3dgC4bCR1}]} \cdots$$

$$\frac{1}{+ [\mathrm{hC3dgC4bC4BP}] + [\mathrm{hC3bC3bH}] + [\mathrm{hC3bC3bHL}] + [\mathrm{hC3bC3bCR1}] + [\mathrm{hC3biC3bH}] + [\mathrm{hC3biC3bHL}] + [\mathrm{hC3biC3bCR1}] + [\mathrm{hiC3biC3bCR1}]} \cdots$$

$$\frac{1}{+ [\mathrm{hiC3bC3dgCR1}] + [\mathrm{fC4bCR1}] + [\mathrm{nfC4bC4BP}] + [\mathrm{fC4bC4BP}] + [\mathrm{fC4bC4bCR1}] + [\mathrm{fC4bC4bC4BP}] + [\mathrm{fC4bC4dCR1}] + [\mathrm{fC4bC4dC4BP}] + [\mathrm{hC4bCR1}]} \cdots$$

$$\left. \frac{1}{+ [\mathrm{hC4bC4BP}] + [\mathrm{hC4bC4bCR1}] + [\mathrm{hC4bC4bC4BP}] + [\mathrm{hC4bC4dCR1}] + [\mathrm{hC4bC4dC4BP}]} \cdots \right)$$

S139

$$\frac{d[\mathrm{fC3bC4bCR1}]}{dt} = k^{+}_{\mathrm{C3bC4bCR1}}[\mathrm{fC3bC4b}][\mathrm{CR1}] - k^{-}_{\mathrm{C3bC4bCR1}}[\mathrm{fC3bC4bCR1}]$$

$$-\left(\frac{k^{\mathrm{FI}}_{\mathrm{catC3bH}}[\mathrm{I}][\mathrm{fC3bC4bCR1}]}{K^{\mathrm{FI}}_{\mathrm{mC3bH}} + [\mathrm{C3(H_2O)H}] + [\mathrm{C3(H_2O)HL}] + [\mathrm{fC3bH}] + [\mathrm{fC3bHL}] + [\mathrm{fC3bCR1}] + [\mathrm{fiC3bCR1}] + [\mathrm{IgGC3bC3bH}] + [\mathrm{IgGC3biC3bH}] + [\mathrm{IgGC3bC3bHL}]} \cdots \right.$$

$$\frac{1}{+ [\mathrm{IgGC3biC3bHL}] + [\mathrm{IgGC3bC3bCR1}] + [\mathrm{IgGC3biC3bCR1}] + [\mathrm{IgGiC3biC3bCR1}] + [\mathrm{IgGiC3bC3dgCR1}] + [\mathrm{fC3bC4bH}] + [\mathrm{fC3bC4bHL}]} \cdots$$

$$\frac{1}{+ [\mathrm{fC3bC4bCR1}] + [\mathrm{fC3bC4bC4BP}] + [\mathrm{fC3bC4dH}] + [\mathrm{fC3bC4dHL}] + [\mathrm{fC3bC4dCR1}] + [\mathrm{fiC3bC4dCR1}] + [\mathrm{fiC3bC4bCR1}] + [\mathrm{fiC3bC4bC4BP}]} \cdots$$

$$\frac{1}{+ [\mathrm{fC3dgC4bCR1}] + [\mathrm{fC3dgC4bC4BP}] + [\mathrm{fC3bC3bH}] + [\mathrm{fC3bC3bHL}] + [\mathrm{fC3bC3bCR1}] + [\mathrm{fC3biC3bH}] + [\mathrm{fC3biC3bHL}] + [\mathrm{fC3biC3bCR1}]} \cdots$$

$$\frac{1}{+ [\mathrm{fiC3biC3bCR1}] + [\mathrm{fiC3bC3dgCR1}] + [\mathrm{hC3bH}] + [\mathrm{hC3bHL}] + [\mathrm{hC3bCR1}] + [\mathrm{hiC3bCR1}] + [\mathrm{hC3bC4bH}] + [\mathrm{hC3bC4bHL}] + [\mathrm{hC3bC4bCR1}]} \cdots$$

$$\frac{1}{+ [\mathrm{hC3bC4bC4BP}] + [\mathrm{hC3bC4dH}] + [\mathrm{hC3bC4dHL}] + [\mathrm{hC3bC4dCR1}] + [\mathrm{hiC3bC4dCR1}] + [\mathrm{hiC3bC4bCR1}] + [\mathrm{hiC3bC4bC4BP}] + [\mathrm{hC3dgC4bCR1}]} \cdots$$

$$\frac{1}{+ [\mathrm{hC3dgC4bC4BP}] + [\mathrm{hC3bC3bH}] + [\mathrm{hC3bC3bHL}] + [\mathrm{hC3bC3bCR1}] + [\mathrm{hC3biC3bH}] + [\mathrm{hC3biC3bHL}] + [\mathrm{hC3biC3bCR1}] + [\mathrm{hiC3biC3bCR1}]} \cdots$$

$$\frac{1}{+ [\mathrm{hiC3bC3dgCR1}] + [\mathrm{fC4bCR1}] + [\mathrm{nfC4bC4BP}] + [\mathrm{fC4bC4BP}] + [\mathrm{fC4bC4bCR1}] + [\mathrm{fC4bC4bC4BP}] + [\mathrm{fC4bC4dCR1}] + [\mathrm{fC4bC4dC4BP}] + [\mathrm{hC4bCR1}]} \cdots$$

$$\left. \frac{1}{+ [\mathrm{hC4bC4BP}] + [\mathrm{hC4bC4bCR1}] + [\mathrm{hC4bC4bC4BP}] + [\mathrm{hC4bC4dCR1}] + [\mathrm{hC4bC4dC4BP}]} \cdots \right)$$

S140

$$\frac{d[\mathrm{fC3bC4bC4BP}]}{dt} = k^{+}_{\mathrm{C4bC4BP}}[\mathrm{fC3bC4b}][\mathrm{C4BP}] - k^{-}_{\mathrm{C4bC4BP}}[\mathrm{fC3bC4bC4BP}]$$

$$-\left(\frac{k^{\mathrm{FI}}_{\mathrm{catC3bH}}[\mathrm{I}][\mathrm{fC3bC4bC4BP}]}{K^{\mathrm{FI}}_{\mathrm{mC3bH}} + [\mathrm{C3(H_2O)H}] + [\mathrm{C3(H_2O)HL}] + [\mathrm{fC3bH}] + [\mathrm{fC3bHL}] + [\mathrm{fC3bCR1}] + [\mathrm{fiC3bCR1}] + [\mathrm{IgGC3bC3bH}] + [\mathrm{IgGC3biC3bH}] + [\mathrm{IgGC3bC3bHL}]} \cdots \right.$$

$$\frac{1}{+ [\mathrm{IgGC3biC3bHL}] + [\mathrm{IgGC3bC3bCR1}] + [\mathrm{IgGC3biC3bCR1}] + [\mathrm{IgGiC3biC3bCR1}] + [\mathrm{IgGiC3bC3dgCR1}] + [\mathrm{fC3bC4bH}] + [\mathrm{fC3bC4bHL}]} \cdots$$

$$\frac{1}{+ [\mathrm{fC3bC4bCR1}] + [\mathrm{fC3bC4bC4BP}] + [\mathrm{fC3bC4dH}] + [\mathrm{fC3bC4dHL}] + [\mathrm{fC3bC4dCR1}] + [\mathrm{fiC3bC4dCR1}] + [\mathrm{fiC3bC4bCR1}] + [\mathrm{fiC3bC4bC4BP}]} \cdots$$

$$\frac{1}{+ [\mathrm{fC3dgC4bCR1}] + [\mathrm{fC3dgC4bC4BP}] + [\mathrm{fC3bC3bH}] + [\mathrm{fC3bC3bHL}] + [\mathrm{fC3bC3bCR1}] + [\mathrm{fC3biC3bH}] + [\mathrm{fC3biC3bHL}] + [\mathrm{fC3biC3bCR1}]} \cdots$$

$$\frac{1}{+ [\mathrm{fiC3biC3bCR1}] + [\mathrm{fiC3bC3dgCR1}] + [\mathrm{hC3bH}] + [\mathrm{hC3bHL}] + [\mathrm{hC3bCR1}] + [\mathrm{hiC3bCR1}] + [\mathrm{hC3bC4bH}] + [\mathrm{hC3bC4bHL}] + [\mathrm{hC3bC4bCR1}]} \cdots$$

$$\frac{1}{+ [\mathrm{hC3bC4bC4BP}] + [\mathrm{hC3bC4dH}] + [\mathrm{hC3bC4dHL}] + [\mathrm{hC3bC4dCR1}] + [\mathrm{hiC3bC4dCR1}] + [\mathrm{hiC3bC4bCR1}] + [\mathrm{hiC3bC4bC4BP}] + [\mathrm{hC3dgC4bCR1}]} \cdots$$

$$\frac{1}{+ [\mathrm{hC3dgC4bC4BP}] + [\mathrm{hC3bC3bH}] + [\mathrm{hC3bC3bHL}] + [\mathrm{hC3bC3bCR1}] + [\mathrm{hC3biC3bH}] + [\mathrm{hC3biC3bHL}] + [\mathrm{hC3biC3bCR1}] + [\mathrm{hiC3biC3bCR1}]} \cdots$$

$$\frac{1}{+ [\mathrm{hiC3bC3dgCR1}] + [\mathrm{fC4bCR1}] + [\mathrm{nfC4bC4BP}] + [\mathrm{fC4bC4BP}] + [\mathrm{fC4bC4bCR1}] + [\mathrm{fC4bC4bC4BP}] + [\mathrm{fC4bC4dCR1}] + [\mathrm{fC4bC4dC4BP}] + [\mathrm{hC4bCR1}]} \cdots$$

$$\left. \frac{1}{+ [\mathrm{hC4bC4BP}] + [\mathrm{hC4bC4bCR1}] + [\mathrm{hC4bC4bC4BP}] + [\mathrm{hC4bC4dCR1}] + [\mathrm{hC4bC4dC4BP}]} \cdots \right)$$

S141

$$\frac{d[\mathrm{fiC3bC4b}]}{dt} =$$

$$\left(\frac{k^{\mathrm{FI}}_{\mathrm{catC3bH}}[\mathrm{I}][\mathrm{fC3bC4bH}]}{K^{\mathrm{FI}}_{\mathrm{mC3bH}} + [\mathrm{C3(H_2O)H}] + [\mathrm{C3(H_2O)HL}] + [\mathrm{fC3bH}] + [\mathrm{fC3bHL}] + [\mathrm{fC3bCR1}] + [\mathrm{fiC3bCR1}] + [\mathrm{IgGC3bC3bH}] + [\mathrm{IgGC3biC3bH}] + [\mathrm{IgGC3bC3bHL}]} \cdots \right.$$

$$\frac{1}{+ [\mathrm{IgGC3biC3bHL}] + [\mathrm{IgGC3bC3bCR1}] + [\mathrm{IgGC3biC3bCR1}] + [\mathrm{IgGiC3biC3bCR1}] + [\mathrm{IgGiC3bC3dgCR1}] + [\mathrm{fC3bC4bH}] + [\mathrm{fC3bC4bHL}]} \cdots$$

$$\frac{1}{+ [\mathrm{fC3bC4bCR1}] + [\mathrm{fC3bC4bC4BP}] + [\mathrm{fC3bC4dH}] + [\mathrm{fC3bC4dHL}] + [\mathrm{fC3bC4dCR1}] + [\mathrm{fiC3bC4dCR1}] + [\mathrm{fiC3bC4bCR1}] + [\mathrm{fiC3bC4bC4BP}]} \cdots$$

$$\frac{1}{+ [\mathrm{fC3dgC4bCR1}] + [\mathrm{fC3dgC4bC4BP}] + [\mathrm{fC3bC3bH}] + [\mathrm{fC3bC3bHL}] + [\mathrm{fC3bC3bCR1}] + [\mathrm{fC3biC3bH}] + [\mathrm{fC3biC3bHL}] + [\mathrm{fC3biC3bCR1}]} \cdots$$

$$\frac{1}{+ [\mathrm{fiC3biC3bCR1}] + [\mathrm{fiC3bC3dgCR1}] + [\mathrm{hC3bH}] + [\mathrm{hC3bHL}] + [\mathrm{hC3bCR1}] + [\mathrm{hiC3bCR1}] + [\mathrm{hC3bC4bH}] + [\mathrm{hC3bC4bHL}] + [\mathrm{hC3bC4bCR1}]} \cdots$$

$$\frac{1}{+ [\mathrm{hC3bC4bC4BP}] + [\mathrm{hC3bC4dH}] + [\mathrm{hC3bC4dHL}] + [\mathrm{hC3bC4dCR1}] + [\mathrm{hiC3bC4dCR1}] + [\mathrm{hiC3bC4bCR1}] + [\mathrm{hiC3bC4bC4BP}] + [\mathrm{hC3dgC4bCR1}]} \cdots$$

$$\frac{1}{+ [\mathrm{hC3dgC4bC4BP}] + [\mathrm{hC3bC3bH}] + [\mathrm{hC3bC3bHL}] + [\mathrm{hC3bC3bCR1}] + [\mathrm{hC3biC3bH}] + [\mathrm{hC3biC3bHL}] + [\mathrm{hC3biC3bCR1}] + [\mathrm{hiC3biC3bCR1}]} \cdots$$

$$\frac{1}{+ [\mathrm{hiC3bC3dgCR1}] + [\mathrm{fC4bCR1}] + [\mathrm{nfC4bC4BP}] + [\mathrm{fC4bC4BP}] + [\mathrm{fC4bC4bCR1}] + [\mathrm{fC4bC4bC4BP}] + [\mathrm{fC4bC4dCR1}] + [\mathrm{fC4bC4dC4BP}] + [\mathrm{hC4bCR1}]} \cdots$$

$$\left. \frac{1}{+ [\mathrm{hC4bC4BP}] + [\mathrm{hC4bC4bCR1}] + [\mathrm{hC4bC4bC4BP}] + [\mathrm{hC4bC4dCR1}] + [\mathrm{hC4bC4dC4BP}]} \cdots \right)$$

$$
+\left(\begin{array}{l}
\dfrac{k^{\mathrm{FI}}_{\mathrm{catC3bH}}[\mathrm{I}][\mathrm{fC3bC4bHL}]}{K^{\mathrm{FI}}_{\mathrm{mC3bH}}+[\mathrm{C3(H_2O)H}]+[\mathrm{C3(H_2O)HL}]+[\mathrm{fC3bH}]+[\mathrm{fC3bHL}]+[\mathrm{fC3bCR1}]+[\mathrm{fiC3bCR1}]+[\mathrm{IgGC3bC3bH}]+[\mathrm{IgGC3biC3bH}]+[\mathrm{IgGC3bC3bHL}]}\cdots \\
\dfrac{1}{+[\mathrm{IgGC3biC3bHL}]+[\mathrm{IgGC3bC3bCR1}]+[\mathrm{IgGC3biC3bCR1}]+[\mathrm{IgGiC3biC3bCR1}]+[\mathrm{IgGiC3bC3dgCR1}]+[\mathrm{fC3bC4bH}]+[\mathrm{fC3bC4bHL}]}\cdots \\
\dfrac{1}{+[\mathrm{fC3bC4bCR1}]+[\mathrm{fC3bC4bC4BP}]+[\mathrm{fC3bC4dH}]+[\mathrm{fC3bC4dHL}]+[\mathrm{fC3bC4dCR1}]+[\mathrm{fiC3bC4dCR1}]+[\mathrm{fiC3bC4bCR1}]+[\mathrm{fiC3bC4bC4BP}]}\cdots \\
\dfrac{1}{+[\mathrm{fC3dgC4bCR1}]+[\mathrm{fC3dgC4bC4BP}]+[\mathrm{fC3bC3bH}]+[\mathrm{fC3bC3bHL}]+[\mathrm{fC3bC3bCR1}]+[\mathrm{fC3biC3bH}]+[\mathrm{fC3biC3bHL}]+[\mathrm{fC3biC3bCR1}]}\cdots \\
\dfrac{1}{+[\mathrm{fiC3biC3bCR1}]+[\mathrm{fiC3bC3dgCR1}]+[\mathrm{hC3bH}]+[\mathrm{hC3bHL}]+[\mathrm{hC3bCR1}]+[\mathrm{hiC3bCR1}]+[\mathrm{hC3bC4bH}]+[\mathrm{hC3bC4bHL}]+[\mathrm{hC3bC4bCR1}]}\cdots \\
\dfrac{1}{+[\mathrm{hC3bC4bC4BP}]+[\mathrm{hC3bC4dH}]+[\mathrm{hC3bC4dHL}]+[\mathrm{hC3bC4dCR1}]+[\mathrm{hiC3bC4dCR1}]+[\mathrm{hiC3bC4bCR1}]+[\mathrm{hiC3bC4bC4BP}]+[\mathrm{hC3dgC4bCR1}]}\cdots \\
\dfrac{1}{+[\mathrm{hC3dgC4bC4BP}]+[\mathrm{hC3bC3bH}]+[\mathrm{hC3bC3bHL}]+[\mathrm{hC3bC3bCR1}]+[\mathrm{hC3biC3bH}]+[\mathrm{hC3biC3bHL}]+[\mathrm{hC3biC3bCR1}]+[\mathrm{hiC3biC3bCR1}]}\cdots \\
\dfrac{1}{+[\mathrm{hiC3bC3dgCR1}]+[\mathrm{fC4bCR1}]+[\mathrm{nfC4bC4BP}]+[\mathrm{fC4bC4BP}]+[\mathrm{fC4bC4bCR1}]+[\mathrm{fC4bC4bC4BP}]+[\mathrm{fC4bC4dCR1}]+[\mathrm{fC4bC4dC4BP}]+[\mathrm{hC4bCR1}]}\cdots \\
\dfrac{1}{+[\mathrm{hC4bC4BP}]+[\mathrm{hC4bC4bCR1}]+[\mathrm{hC4bC4bC4BP}]+[\mathrm{hC4bC4dCR1}]+[\mathrm{hC4bC4dC4BP}]}\cdots
\end{array}\right)
$$

$$
+\left(\begin{array}{l}
\dfrac{k^{\mathrm{FI}}_{\mathrm{catC3bH}}[\mathrm{I}][\mathrm{fC3bC4bCR1}]}{K^{\mathrm{FI}}_{\mathrm{mC3bH}}+[\mathrm{C3(H_2O)H}]+[\mathrm{C3(H_2O)HL}]+[\mathrm{fC3bH}]+[\mathrm{fC3bHL}]+[\mathrm{fC3bCR1}]+[\mathrm{fiC3bCR1}]+[\mathrm{IgGC3bC3bH}]+[\mathrm{IgGC3biC3bH}]+[\mathrm{IgGC3bC3bHL}]}\cdots \\
\dfrac{1}{+[\mathrm{IgGC3biC3bHL}]+[\mathrm{IgGC3bC3bCR1}]+[\mathrm{IgGC3biC3bCR1}]+[\mathrm{IgGiC3biC3bCR1}]+[\mathrm{IgGiC3bC3dgCR1}]+[\mathrm{fC3bC4bH}]+[\mathrm{fC3bC4bHL}]}\cdots \\
\dfrac{1}{+[\mathrm{fC3bC4bCR1}]+[\mathrm{fC3bC4bC4BP}]+[\mathrm{fC3bC4dH}]+[\mathrm{fC3bC4dHL}]+[\mathrm{fC3bC4dCR1}]+[\mathrm{fiC3bC4dCR1}]+[\mathrm{fiC3bC4bCR1}]+[\mathrm{fiC3bC4bC4BP}]}\cdots \\
\dfrac{1}{+[\mathrm{fC3dgC4bCR1}]+[\mathrm{fC3dgC4bC4BP}]+[\mathrm{fC3bC3bH}]+[\mathrm{fC3bC3bHL}]+[\mathrm{fC3bC3bCR1}]+[\mathrm{fC3biC3bH}]+[\mathrm{fC3biC3bHL}]+[\mathrm{fC3biC3bCR1}]}\cdots \\
\dfrac{1}{+[\mathrm{fiC3biC3bCR1}]+[\mathrm{fiC3bC3dgCR1}]+[\mathrm{hC3bH}]+[\mathrm{hC3bHL}]+[\mathrm{hC3bCR1}]+[\mathrm{hiC3bCR1}]+[\mathrm{hC3bC4bH}]+[\mathrm{hC3bC4bHL}]+[\mathrm{hC3bC4bCR1}]}\cdots \\
\dfrac{1}{+[\mathrm{hC3bC4bC4BP}]+[\mathrm{hC3bC4dH}]+[\mathrm{hC3bC4dHL}]+[\mathrm{hC3bC4dCR1}]+[\mathrm{hiC3bC4dCR1}]+[\mathrm{hiC3bC4bCR1}]+[\mathrm{hiC3bC4bC4BP}]+[\mathrm{hC3dgC4bCR1}]}\cdots \\
\dfrac{1}{+[\mathrm{hC3dgC4bC4BP}]+[\mathrm{hC3bC3bH}]+[\mathrm{hC3bC3bHL}]+[\mathrm{hC3bC3bCR1}]+[\mathrm{hC3biC3bH}]+[\mathrm{hC3biC3bHL}]+[\mathrm{hC3biC3bCR1}]+[\mathrm{hiC3biC3bCR1}]}\cdots \\
\dfrac{1}{+[\mathrm{hiC3bC3dgCR1}]+[\mathrm{fC4bCR1}]+[\mathrm{nfC4bC4BP}]+[\mathrm{fC4bC4BP}]+[\mathrm{fC4bC4bCR1}]+[\mathrm{fC4bC4bC4BP}]+[\mathrm{fC4bC4dCR1}]+[\mathrm{fC4bC4dC4BP}]+[\mathrm{hC4bCR1}]}\cdots \\
\dfrac{1}{+[\mathrm{hC4bC4BP}]+[\mathrm{hC4bC4bCR1}]+[\mathrm{hC4bC4bC4BP}]+[\mathrm{hC4bC4dCR1}]+[\mathrm{hC4bC4dC4BP}]}\cdots
\end{array}\right)
$$

$$
-k^{+}_{\mathrm{iC3bC4bCR1}}[\mathrm{fiC3bC4b}][\mathrm{CR1}]+k^{-}_{\mathrm{iC3bC4bCR1}}[\mathrm{fiC3bC4bCR1}]-k^{+}_{\mathrm{C4bC4BP}}[\mathrm{fiC3bC4b}][\mathrm{C4BP}]+k^{-}_{\mathrm{C4bC4BP}}[\mathrm{fiC3bC4bC4BP}]
$$

S142

$$\frac{d[\text{fiC3bC4bCR1}]}{dt} = k^{+}_{\text{iC3bC4bCR1}}[\text{fiC3bC4b}][\text{CR1}] - k^{-}_{\text{iC3bC4bCR1}}[\text{fiC3bC4bCR1}]$$

$$- \left(\frac{k^{\text{FI}}_{\text{catC3bH}}[\text{I}][\text{fiC3bC4bCR1}]}{K^{\text{FI}}_{\text{mC3bH}} + [\text{C3(H}_2\text{O)H}] + [\text{C3(H}_2\text{O)HL}] + [\text{fC3bH}] + [\text{fC3bHL}] + [\text{fC3bCR1}] + [\text{fiC3bCR1}] + [\text{IgGC3bC3bH}] + [\text{IgGC3biC3bH}] + [\text{IgGC3bC3bHL}]} \cdots \right.$$

$$\frac{1}{+ [\text{IgGC3biC3bHL}] + [\text{IgGC3bC3bCR1}] + [\text{IgGC3biC3bCR1}] + [\text{IgGiC3biC3bCR1}] + [\text{IgGiC3bC3dgCR1}] + [\text{fC3bC4bH}] + [\text{fC3bC4bHL}]} \cdots$$

$$\frac{1}{+ [\text{fC3bC4bCR1}] + [\text{fC3bC4bC4BP}] + [\text{fC3bC4dH}] + [\text{fC3bC4dHL}] + [\text{fC3bC4dCR1}] + [\text{fiC3bC4dCR1}] + [\text{fiC3bC4bCR1}] + [\text{fiC3bC4bC4BP}]} \cdots$$

$$\frac{1}{+ [\text{fC3dgC4bCR1}] + [\text{fC3dgC4bC4BP}] + [\text{fC3bC3bH}] + [\text{fC3bC3bHL}] + [\text{fC3bC3bCR1}] + [\text{fC3biC3bH}] + [\text{fC3biC3bHL}] + [\text{fC3biC3bCR1}]} \cdots$$

$$\frac{1}{+ [\text{fiC3biC3bCR1}] + [\text{fiC3bC3dgCR1}] + [\text{hC3bH}] + [\text{hC3bHL}] + [\text{hC3bCR1}] + [\text{hiC3bCR1}] + [\text{hC3bC4bH}] + [\text{hC3bC4bHL}] + [\text{hC3bC4bCR1}]} \cdots$$

$$\frac{1}{+ [\text{hC3bC4bC4BP}] + [\text{hC3bC4dH}] + [\text{hC3bC4dHL}] + [\text{hC3bC4dCR1}] + [\text{hiC3bC4dCR1}] + [\text{hiC3bC4bCR1}] + [\text{hiC3bC4bC4BP}] + [\text{hC3dgC4bCR1}]} \cdots$$

$$\frac{1}{+ [\text{hC3dgC4bC4BP}] + [\text{hC3bC3bH}] + [\text{hC3bC3bHL}] + [\text{hC3bC3bCR1}] + [\text{hC3biC3bH}] + [\text{hC3biC3bHL}] + [\text{hC3biC3bCR1}] + [\text{hiC3biC3bCR1}]} \cdots$$

$$\frac{1}{+ [\text{hiC3bC3dgCR1}] + [\text{fC4bCR1}] + [\text{nfC4bC4BP}] + [\text{fC4bC4BP}] + [\text{fC4bC4bCR1}] + [\text{fC4bC4bC4BP}] + [\text{fC4bC4dCR1}] + [\text{fC4bC4dC4BP}] + [\text{hC4bCR1}]} \cdots$$

$$\left. \frac{1}{+ [\text{hC4bC4BP}] + [\text{hC4bC4bCR1}] + [\text{hC4bC4bC4BP}] + [\text{hC4bC4dCR1}] + [\text{hC4bC4dC4BP}]} \cdots \right)$$

S143

$$\frac{d[\text{fiC3bC4bC4BP}]}{dt} = k^{+}_{\text{C4bC4BP}}[\text{fiC3bC4b}][\text{C4BP}] - k^{-}_{\text{C4bC4BP}}[\text{fiC3bC4bBP}]$$

$$- \left(\frac{k^{\text{FI}}_{\text{catC3bH}}[\text{I}][\text{fiC3bC4bC4BP}]}{K^{\text{FI}}_{\text{mC3bH}} + [\text{C3(H}_2\text{O)H}] + [\text{C3(H}_2\text{O)HL}] + [\text{fC3bH}] + [\text{fC3bHL}] + [\text{fC3bCR1}] + [\text{fiC3bCR1}] + [\text{IgGC3bC3bH}] + [\text{IgGC3biC3bH}] + [\text{IgGC3bC3bHL}]} \cdots \right.$$

$$\frac{1}{+ [\text{IgGC3biC3bHL}] + [\text{IgGC3bC3bCR1}] + [\text{IgGC3biC3bCR1}] + [\text{IgGiC3biC3bCR1}] + [\text{IgGiC3bC3dgCR1}] + [\text{fC3bC4bH}] + [\text{fC3bC4bHL}]} \cdots$$

$$\frac{1}{+ [\text{fC3bC4bCR1}] + [\text{fC3bC4bC4BP}] + [\text{fC3bC4dH}] + [\text{fC3bC4dHL}] + [\text{fC3bC4dCR1}] + [\text{fiC3bC4dCR1}] + [\text{fiC3bC4bCR1}] + [\text{fiC3bC4bC4BP}]} \cdots$$

$$\frac{1}{+ [\text{fC3dgC4bCR1}] + [\text{fC3dgC4bC4BP}] + [\text{fC3bC3bH}] + [\text{fC3bC3bHL}] + [\text{fC3bC3bCR1}] + [\text{fC3biC3bH}] + [\text{fC3biC3bHL}] + [\text{fC3biC3bCR1}]} \cdots$$

$$\frac{1}{+ [\text{fiC3biC3bCR1}] + [\text{fiC3bC3dgCR1}] + [\text{hC3bH}] + [\text{hC3bHL}] + [\text{hC3bCR1}] + [\text{hiC3bCR1}] + [\text{hC3bC4bH}] + [\text{hC3bC4bHL}] + [\text{hC3bC4bCR1}]} \cdots$$

$$\frac{1}{+ [\text{hC3bC4bC4BP}] + [\text{hC3bC4dH}] + [\text{hC3bC4dHL}] + [\text{hC3bC4dCR1}] + [\text{hiC3bC4dCR1}] + [\text{hiC3bC4bCR1}] + [\text{hiC3bC4bC4BP}] + [\text{hC3dgC4bCR1}]} \cdots$$

$$\frac{1}{+ [\text{hC3dgC4bC4BP}] + [\text{hC3bC3bH}] + [\text{hC3bC3bHL}] + [\text{hC3bC3bCR1}] + [\text{hC3biC3bH}] + [\text{hC3biC3bHL}] + [\text{hC3biC3bCR1}] + [\text{hiC3biC3bCR1}]} \cdots$$

$$\frac{1}{+ [\text{hiC3bC3dgCR1}] + [\text{fC4bCR1}] + [\text{nfC4bC4BP}] + [\text{fC4bC4BP}] + [\text{fC4bC4bCR1}] + [\text{fC4bC4bC4BP}] + [\text{fC4bC4dCR1}] + [\text{fC4bC4dC4BP}] + [\text{hC4bCR1}]} \cdots$$

$$\left. \frac{1}{+ [\text{hC4bC4BP}] + [\text{hC4bC4bCR1}] + [\text{hC4bC4bC4BP}] + [\text{hC4bC4dCR1}] + [\text{hC4bC4dC4BP}]} \cdots \right)$$

S144

$$\frac{d[\text{fC3bC4d}]}{dt} =$$

$$\left(\frac{k_{\text{catC3bH}}^{\text{FI}}[\text{I}][\text{fC3bC4bC4BP}]}{K_{\text{mC3bH}}^{\text{FI}} + [\text{C3(H}_2\text{O)H}] + [\text{C3(H}_2\text{O)HL}] + [\text{fC3bH}] + [\text{fC3bHL}] + [\text{fC3bCR1}] + [\text{fiC3bCR1}] + [\text{IgGC3bC3bH}] + [\text{IgGC3biC3bH}] + [\text{IgGC3bC3bHL}]} \cdots \right.$$

$$\frac{1}{+[\text{IgGC3biC3bHL}] + [\text{IgGC3bC3bCR1}] + [\text{IgGC3biC3bCR1}] + [\text{IgGiC3biC3bCR1}] + [\text{IgGiC3bC3dgCR1}] + [\text{fC3bC4bH}] + [\text{fC3bC4bHL}]} \cdots$$

$$\frac{1}{+[\text{fC3bC4bCR1}] + [\text{fC3bC4bC4BP}] + [\text{fC3bC4dH}] + [\text{fC3bC4dHL}] + [\text{fC3bC4dCR1}] + [\text{fiC3bC4dCR1}] + [\text{fiC3bC4bCR1}] + [\text{fiC3bC4bC4BP}]} \cdots$$

$$\frac{1}{+[\text{fC3dgC4bCR1}] + [\text{fC3dgC4bC4BP}] + [\text{fC3bC3bH}] + [\text{fC3bC3bHL}] + [\text{fC3bC3bCR1}] + [\text{fC3biC3bH}] + [\text{fC3biC3bHL}] + [\text{fC3biC3bCR1}]} \cdots$$

$$\frac{1}{+[\text{fiC3biC3bCR1}] + [\text{fiC3bC3dgCR1}] + [\text{hC3bH}] + [\text{hC3bHL}] + [\text{hC3bCR1}] + [\text{hiC3bCR1}] + [\text{hC3bC4bH}] + [\text{hC3bC4bHL}] + [\text{hC3bC4bCR1}]} \cdots$$

$$\frac{1}{+[\text{hC3bC4bC4BP}] + [\text{hC3bC4dH}] + [\text{hC3bC4dHL}] + [\text{hC3bC4dCR1}] + [\text{hiC3bC4dCR1}] + [\text{hiC3bC4bCR1}] + [\text{hiC3bC4bC4BP}] + [\text{hC3dgC4bCR1}]} \cdots$$

$$\frac{1}{+[\text{hC3dgC4bC4BP}] + [\text{hC3bC3bH}] + [\text{hC3bC3bHL}] + [\text{hC3bC3bCR1}] + [\text{hC3biC3bH}] + [\text{hC3biC3bHL}] + [\text{hC3biC3bCR1}] + [\text{hiC3biC3bCR1}]} \cdots$$

$$\frac{1}{+[\text{hiC3bC3dgCR1}] + [\text{fC4bCR1}] + [\text{nfC4bC4BP}] + [\text{fC4bC4BP}] + [\text{fC4bC4bCR1}] + [\text{fC4bC4bC4BP}] + [\text{fC4bC4dCR1}] + [\text{fC4bC4dC4BP}] + [\text{hC4bCR1}]} \cdots$$

$$\left. \frac{1}{+[\text{hC4bC4BP}] + [\text{hC4bC4bCR1}] + [\text{hC4bC4bC4BP}] + [\text{hC4bC4dCR1}] + [\text{hC4bC4dC4BP}]} \cdots \right)$$

$$- k_{\text{C3bH}}^{+}[\text{fC3bC4d}][\text{H}] + k_{\text{C3bH}}^{-}[\text{fC3bC4dH}] - k_{\text{C3bHL}}^{+}[\text{fC3bC4d}][\text{HL}] + k_{\text{C3bHL}}^{-}[\text{fC3bC4dHL}] - k_{\text{C3bC4dCR1}}^{+}[\text{fC3bC4d}][\text{CR1}]$$

$$+ k_{\text{C3bC4dCR1}}^{-}[\text{fC3bC4dCR1}]$$

S145

$$\frac{d[\text{fC3bC4dH}]}{dt} = k_{\text{C3bH}}^{+}[\text{fC3bC4d}][\text{H}] - k_{\text{C3bH}}^{-}[\text{fC3bC4dH}]$$

$$- \left(\frac{k_{\text{catC3bH}}^{\text{FI}}[\text{I}][\text{fC3bC4dH}]}{K_{\text{mC3bH}}^{\text{FI}} + [\text{C3(H}_2\text{O)H}] + [\text{C3(H}_2\text{O)HL}] + [\text{fC3bH}] + [\text{fC3bHL}] + [\text{fC3bCR1}] + [\text{fiC3bCR1}] + [\text{IgGC3bC3bH}] + [\text{IgGC3biC3bH}] + [\text{IgGC3bC3bHL}]} \cdots \right.$$

$$\frac{1}{+[\text{IgGC3biC3bHL}] + [\text{IgGC3bC3bCR1}] + [\text{IgGC3biC3bCR1}] + [\text{IgGiC3biC3bCR1}] + [\text{IgGiC3bC3dgCR1}] + [\text{fC3bC4bH}] + [\text{fC3bC4bHL}]} \cdots$$

$$\frac{1}{+[\text{fC3bC4bCR1}] + [\text{fC3bC4bC4BP}] + [\text{fC3bC4dH}] + [\text{fC3bC4dHL}] + [\text{fC3bC4dCR1}] + [\text{fiC3bC4dCR1}] + [\text{fiC3bC4bCR1}] + [\text{fiC3bC4bC4BP}]} \cdots$$

$$\frac{1}{+[\text{fC3dgC4bCR1}] + [\text{fC3dgC4bC4BP}] + [\text{fC3bC3bH}] + [\text{fC3bC3bHL}] + [\text{fC3bC3bCR1}] + [\text{fC3biC3bH}] + [\text{fC3biC3bHL}] + [\text{fC3biC3bCR1}]} \cdots$$

$$\frac{1}{+[\text{fiC3biC3bCR1}] + [\text{fiC3bC3dgCR1}] + [\text{hC3bH}] + [\text{hC3bHL}] + [\text{hC3bCR1}] + [\text{hiC3bCR1}] + [\text{hC3bC4bH}] + [\text{hC3bC4bHL}] + [\text{hC3bC4bCR1}]} \cdots$$

$$\frac{1}{+[\text{hC3bC4bC4BP}] + [\text{hC3bC4dH}] + [\text{hC3bC4dHL}] + [\text{hC3bC4dCR1}] + [\text{hiC3bC4dCR1}] + [\text{hiC3bC4bCR1}] + [\text{hiC3bC4bC4BP}] + [\text{hC3dgC4bCR1}]} \cdots$$

$$\frac{1}{+[\text{hC3dgC4bC4BP}] + [\text{hC3bC3bH}] + [\text{hC3bC3bHL}] + [\text{hC3bC3bCR1}] + [\text{hC3biC3bH}] + [\text{hC3biC3bHL}] + [\text{hC3biC3bCR1}] + [\text{hiC3biC3bCR1}]} \cdots$$

$$\frac{1}{+[\text{hiC3bC3dgCR1}] + [\text{fC4bCR1}] + [\text{nfC4bC4BP}] + [\text{fC4bC4BP}] + [\text{fC4bC4bCR1}] + [\text{fC4bC4bC4BP}] + [\text{fC4bC4dCR1}] + [\text{fC4bC4dC4BP}] + [\text{hC4bCR1}]} \cdots$$

$$\left. \frac{1}{+[\text{hC4bC4BP}] + [\text{hC4bC4bCR1}] + [\text{hC4bC4bC4BP}] + [\text{hC4bC4dCR1}] + [\text{hC4bC4dC4BP}]} \cdots \right)$$

S146

$$\frac{d[\text{fC3bC4dHL}]}{dt} = k^{+}_{\text{C3bHL}}[\text{fC3bC4d}][\text{HL}] - k^{-}_{\text{C3bHL}}[\text{fC3bC4dHL}]$$

$$-\left(\frac{k^{\text{FI}}_{\text{catC3bH}}[\text{I}][\text{fC3bC4dHL}]}{K^{\text{FI}}_{\text{mC3bH}} + [\text{C3(H}_2\text{O)H}] + [\text{C3(H}_2\text{O)HL}] + [\text{fC3bH}] + [\text{fC3bHL}] + [\text{fC3bCR1}] + [\text{fiC3bCR1}] + [\text{IgGC3bC3bH}] + [\text{IgGC3biC3bH}] + [\text{IgGC3bC3bHL}]} \cdots \right.$$

$$\frac{1}{+[\text{IgGC3biC3bHL}] + [\text{IgGC3bC3bCR1}] + [\text{IgGC3biC3bCR1}] + [\text{IgGiC3biC3bCR1}] + [\text{IgGiC3bC3dgCR1}] + [\text{fC3bC4bH}] + [\text{fC3bC4bHL}]} \cdots$$

$$\frac{1}{+[\text{fC3bC4bCR1}] + [\text{fC3bC4bC4BP}] + [\text{fC3bC4dH}] + [\text{fC3bC4dHL}] + [\text{fC3bC4dCR1}] + [\text{fiC3bC4dCR1}] + [\text{fiC3bC4bCR1}] + [\text{fiC3bC4bC4BP}]} \cdots$$

$$\frac{1}{+[\text{fC3dgC4bCR1}] + [\text{fC3dgC4bC4BP}] + [\text{fC3bC3bH}] + [\text{fC3bC3bHL}] + [\text{fC3bC3bCR1}] + [\text{fC3biC3bH}] + [\text{fC3biC3bHL}] + [\text{fC3biC3bCR1}]} \cdots$$

$$\frac{1}{+[\text{fiC3biC3bCR1}] + [\text{fiC3bC3dgCR1}] + [\text{hC3bH}] + [\text{hC3bHL}] + [\text{hC3bCR1}] + [\text{hiC3bCR1}] + [\text{hC3bC4bH}] + [\text{hC3bC4bHL}] + [\text{hC3bC4bCR1}]} \cdots$$

$$\frac{1}{+[\text{hC3bC4bC4BP}] + [\text{hC3bC4dH}] + [\text{hC3bC4dHL}] + [\text{hC3bC4dCR1}] + [\text{hiC3bC4dCR1}] + [\text{hiC3bC4bCR1}] + [\text{hiC3bC4bC4BP}] + [\text{hC3dgC4bCR1}]} \cdots$$

$$\frac{1}{+[\text{hC3dgC4bC4BP}] + [\text{hC3bC3bH}] + [\text{hC3bC3bHL}] + [\text{hC3bC3bCR1}] + [\text{hC3biC3bH}] + [\text{hC3biC3bHL}] + [\text{hC3biC3bCR1}] + [\text{hiC3biC3bCR1}]} \cdots$$

$$\frac{1}{+[\text{hiC3bC3dgCR1}] + [\text{fC4bCR1}] + [\text{nfC4bC4BP}] + [\text{fC4bC4BP}] + [\text{fC4bC4bCR1}] + [\text{fC4bC4bC4BP}] + [\text{fC4bC4dCR1}] + [\text{fC4bC4dC4BP}] + [\text{hC4bCR1}]} \cdots$$

$$\left. \frac{1}{+[\text{hC4bC4BP}] + [\text{hC4bC4bCR1}] + [\text{hC4bC4bC4BP}] + [\text{hC4bC4dCR1}] + [\text{hC4bC4dC4BP}]} \cdots \right)$$

S147

$$\frac{d[\text{fC3bC4dCR1}]}{dt} = k^{+}_{\text{C3bC4dCR1}}[\text{fC3bC4d}][\text{CR1}] - k^{-}_{\text{C3bC4dCR1}}[\text{fC3bC4dCR1}]$$

$$-\left(\frac{k^{\text{FI}}_{\text{catC3bH}}[\text{I}][\text{fC3bC4dCR1}]}{K^{\text{FI}}_{\text{mC3bH}} + [\text{C3(H}_2\text{O)H}] + [\text{C3(H}_2\text{O)HL}] + [\text{fC3bH}] + [\text{fC3bHL}] + [\text{fC3bCR1}] + [\text{fiC3bCR1}] + [\text{IgGC3bC3bH}] + [\text{IgGC3biC3bH}] + [\text{IgGC3bC3bHL}]} \cdots \right.$$

$$\frac{1}{+[\text{IgGC3biC3bHL}] + [\text{IgGC3bC3bCR1}] + [\text{IgGC3biC3bCR1}] + [\text{IgGiC3biC3bCR1}] + [\text{IgGiC3bC3dgCR1}] + [\text{fC3bC4bH}] + [\text{fC3bC4bHL}]} \cdots$$

$$\frac{1}{+[\text{fC3bC4bCR1}] + [\text{fC3bC4bC4BP}] + [\text{fC3bC4dH}] + [\text{fC3bC4dHL}] + [\text{fC3bC4dCR1}] + [\text{fiC3bC4dCR1}] + [\text{fiC3bC4bCR1}] + [\text{fiC3bC4bC4BP}]} \cdots$$

$$\frac{1}{+[\text{fC3dgC4bCR1}] + [\text{fC3dgC4bC4BP}] + [\text{fC3bC3bH}] + [\text{fC3bC3bHL}] + [\text{fC3bC3bCR1}] + [\text{fC3biC3bH}] + [\text{fC3biC3bHL}] + [\text{fC3biC3bCR1}]} \cdots$$

$$\frac{1}{+[\text{fiC3biC3bCR1}] + [\text{fiC3bC3dgCR1}] + [\text{hC3bH}] + [\text{hC3bHL}] + [\text{hC3bCR1}] + [\text{hiC3bCR1}] + [\text{hC3bC4bH}] + [\text{hC3bC4bHL}] + [\text{hC3bC4bCR1}]} \cdots$$

$$\frac{1}{+[\text{hC3bC4bC4BP}] + [\text{hC3bC4dH}] + [\text{hC3bC4dHL}] + [\text{hC3bC4dCR1}] + [\text{hiC3bC4dCR1}] + [\text{hiC3bC4bCR1}] + [\text{hiC3bC4bC4BP}] + [\text{hC3dgC4bCR1}]} \cdots$$

$$\frac{1}{+[\text{hC3dgC4bC4BP}] + [\text{hC3bC3bH}] + [\text{hC3bC3bHL}] + [\text{hC3bC3bCR1}] + [\text{hC3biC3bH}] + [\text{hC3biC3bHL}] + [\text{hC3biC3bCR1}] + [\text{hiC3biC3bCR1}]} \cdots$$

$$\frac{1}{+[\text{hiC3bC3dgCR1}] + [\text{fC4bCR1}] + [\text{nfC4bC4BP}] + [\text{fC4bC4BP}] + [\text{fC4bC4bCR1}] + [\text{fC4bC4bC4BP}] + [\text{fC4bC4dCR1}] + [\text{fC4bC4dC4BP}] + [\text{hC4bCR1}]} \cdots$$

$$\left. \frac{1}{+[\text{hC4bC4BP}] + [\text{hC4bC4bCR1}] + [\text{hC4bC4bC4BP}] + [\text{hC4bC4dCR1}] + [\text{hC4bC4dC4BP}]} \cdots \right)$$

S148

$$\frac{d[\text{fiC3bC4d}]}{dt} =$$

$$\left(\frac{k^{\text{FI}}_{\text{catC3bH}}[\text{I}][\text{fC3bC4dH}]}{K^{\text{FI}}_{\text{mC3bH}} + [\text{C3(H}_2\text{O)H}] + [\text{C3(H}_2\text{O)HL}] + [\text{fC3bH}] + [\text{fC3bHL}] + [\text{fC3bCR1}] + [\text{fiC3bCR1}] + [\text{IgGC3bC3bH}] + [\text{IgGC3biC3bH}] + [\text{IgGC3bC3bHL}]} \cdots \right.$$
$$\frac{1}{+ [\text{IgGC3biC3bHL}] + [\text{IgGC3bC3bCR1}] + [\text{IgGC3biC3bCR1}] + [\text{IgGiC3biC3bCR1}] + [\text{IgGiC3bC3dgCR1}] + [\text{fC3bC4bH}] + [\text{fC3bC4bHL}]} \cdots$$
$$\frac{1}{+ [\text{fC3bC4bCR1}] + [\text{fC3bC4bC4BP}] + [\text{fC3bC4dH}] + [\text{fC3bC4dHL}] + [\text{fC3bC4dCR1}] + [\text{fiC3bC4dCR1}] + [\text{fiC3bC4bCR1}] + [\text{fiC3bC4bC4BP}]} \cdots$$
$$\frac{1}{+ [\text{fC3dgC4bCR1}] + [\text{fC3dgC4bC4BP}] + [\text{fC3bC3bH}] + [\text{fC3bC3bHL}] + [\text{fC3bC3bCR1}] + [\text{fC3biC3bH}] + [\text{fC3biC3bHL}] + [\text{fC3biC3bCR1}]} \cdots$$
$$\frac{1}{+ [\text{fiC3biC3bCR1}] + [\text{fiC3bC3dgCR1}] + [\text{hC3bH}] + [\text{hC3bHL}] + [\text{hC3bCR1}] + [\text{hiC3bCR1}] + [\text{hC3bC4bH}] + [\text{hC3bC4bHL}] + [\text{hC3bC4bCR1}]} \cdots$$
$$\frac{1}{+ [\text{hC3bC4bC4BP}] + [\text{hC3bC4dH}] + [\text{hC3bC4dHL}] + [\text{hC3bC4dCR1}] + [\text{hiC3bC4dCR1}] + [\text{hiC3bC4bCR1}] + [\text{hiC3bC4bC4BP}] + [\text{hC3dgC4bCR1}]} \cdots$$
$$\frac{1}{+ [\text{hC3dgC4bC4BP}] + [\text{hC3bC3bH}] + [\text{hC3bC3bHL}] + [\text{hC3bC3bCR1}] + [\text{hC3biC3bH}] + [\text{hC3biC3bHL}] + [\text{hC3biC3bCR1}] + [\text{hiC3biC3bCR1}]} \cdots$$
$$\frac{1}{+ [\text{hiC3bC3dgCR1}] + [\text{fC4bCR1}] + [\text{nfC4bC4BP}] + [\text{fC4bC4BP}] + [\text{fC4bC4bCR1}] + [\text{fC4bC4bC4BP}] + [\text{fC4bC4dCR1}] + [\text{fC4bC4dC4BP}] + [\text{hC4bCR1}]} \cdots$$
$$\left. \frac{1}{+ [\text{hC4bC4BP}] + [\text{hC4bC4bCR1}] + [\text{hC4bC4bC4BP}] + [\text{hC4bC4dCR1}] + [\text{hC4bC4dC4BP}]} \cdots \right)$$

$$+ \left(\frac{k^{\text{FI}}_{\text{catC3bH}}[\text{I}][\text{fC3bC4dHL}]}{K^{\text{FI}}_{\text{mC3bH}} + [\text{C3(H}_2\text{O)H}] + [\text{C3(H}_2\text{O)HL}] + [\text{fC3bH}] + [\text{fC3bHL}] + [\text{fC3bCR1}] + [\text{fiC3bCR1}] + [\text{IgGC3bC3bH}] + [\text{IgGC3biC3bH}] + [\text{IgGC3bC3bHL}]} \cdots \right.$$
$$\frac{1}{+ [\text{IgGC3biC3bHL}] + [\text{IgGC3bC3bCR1}] + [\text{IgGC3biC3bCR1}] + [\text{IgGiC3biC3bCR1}] + [\text{IgGiC3bC3dgCR1}] + [\text{fC3bC4bH}] + [\text{fC3bC4bHL}]} \cdots$$
$$\frac{1}{+ [\text{fC3bC4bCR1}] + [\text{fC3bC4bC4BP}] + [\text{fC3bC4dH}] + [\text{fC3bC4dHL}] + [\text{fC3bC4dCR1}] + [\text{fiC3bC4dCR1}] + [\text{fiC3bC4bCR1}] + [\text{fiC3bC4bC4BP}]} \cdots$$
$$\frac{1}{+ [\text{fC3dgC4bCR1}] + [\text{fC3dgC4bC4BP}] + [\text{fC3bC3bH}] + [\text{fC3bC3bHL}] + [\text{fC3bC3bCR1}] + [\text{fC3biC3bH}] + [\text{fC3biC3bHL}] + [\text{fC3biC3bCR1}]} \cdots$$
$$\frac{1}{+ [\text{fiC3biC3bCR1}] + [\text{fiC3bC3dgCR1}] + [\text{hC3bH}] + [\text{hC3bHL}] + [\text{hC3bCR1}] + [\text{hiC3bCR1}] + [\text{hC3bC4bH}] + [\text{hC3bC4bHL}] + [\text{hC3bC4bCR1}]} \cdots$$
$$\frac{1}{+ [\text{hC3bC4bC4BP}] + [\text{hC3bC4dH}] + [\text{hC3bC4dHL}] + [\text{hC3bC4dCR1}] + [\text{hiC3bC4dCR1}] + [\text{hiC3bC4bCR1}] + [\text{hiC3bC4bC4BP}] + [\text{hC3dgC4bCR1}]} \cdots$$
$$\frac{1}{+ [\text{hC3dgC4bC4BP}] + [\text{hC3bC3bH}] + [\text{hC3bC3bHL}] + [\text{hC3bC3bCR1}] + [\text{hC3biC3bH}] + [\text{hC3biC3bHL}] + [\text{hC3biC3bCR1}] + [\text{hiC3biC3bCR1}]} \cdots$$
$$\frac{1}{+ [\text{hiC3bC3dgCR1}] + [\text{fC4bCR1}] + [\text{nfC4bC4BP}] + [\text{fC4bC4BP}] + [\text{fC4bC4bCR1}] + [\text{fC4bC4bC4BP}] + [\text{fC4bC4dCR1}] + [\text{fC4bC4dC4BP}] + [\text{hC4bCR1}]} \cdots$$
$$\left. \frac{1}{+ [\text{hC4bC4BP}] + [\text{hC4bC4bCR1}] + [\text{hC4bC4bC4BP}] + [\text{hC4bC4dCR1}] + [\text{hC4bC4dC4BP}]} \cdots \right)$$

$$
+\left(\begin{array}{l}
\dfrac{k_{\text{catC3bH}}^{\text{FI}}[\text{I}][\text{fC3bC4dCR1}]}{K_{\text{mC3bH}}^{\text{FI}}+[\text{C3(H}_2\text{O)H}]+[\text{C3(H}_2\text{O)HL}]+[\text{fC3bH}]+[\text{fC3bHL}]+[\text{fC3bCR1}]+[\text{fiC3bCR1}]+[\text{IgGC3bC3bH}]+[\text{IgGC3biC3bH}]+[\text{IgGC3bC3bHL}]}\cdots \\
\dfrac{1}{+[\text{IgGC3biC3bHL}]+[\text{IgGC3bC3bCR1}]+[\text{IgGC3biC3bCR1}]+[\text{IgGiC3biC3bCR1}]+[\text{IgGiC3bC3dgCR1}]+[\text{fC3bC4bH}]+[\text{fC3bC4bHL}]}\cdots \\
\dfrac{1}{+[\text{fC3bC4bCR1}]+[\text{fC3bC4bC4BP}]+[\text{fC3bC4dH}]+[\text{fC3bC4dHL}]+[\text{fC3bC4dCR1}]+[\text{fiC3bC4dCR1}]+[\text{fiC3bC4bCR1}]+[\text{fiC3bC4bC4BP}]}\cdots \\
\dfrac{1}{+[\text{fC3dgC4bCR1}]+[\text{fC3dgC4bC4BP}]+[\text{fC3bC3bH}]+[\text{fC3bC3bHL}]+[\text{fC3bC3bCR1}]+[\text{fC3biC3bH}]+[\text{fC3biC3bHL}]+[\text{fC3biC3bCR1}]}\cdots \\
\dfrac{1}{+[\text{fiC3biC3bCR1}]+[\text{fiC3bC3dgCR1}]+[\text{hC3bH}]+[\text{hC3bHL}]+[\text{hC3bCR1}]+[\text{hiC3bCR1}]+[\text{hC3bC4bH}]+[\text{hC3bC4bHL}]+[\text{hC3bC4bCR1}]}\cdots \\
\dfrac{1}{+[\text{hC3bC4bC4BP}]+[\text{hC3bC4dH}]+[\text{hC3bC4dHL}]+[\text{hC3bC4dCR1}]+[\text{hiC3bC4dCR1}]+[\text{hiC3bC4bCR1}]+[\text{hiC3bC4bC4BP}]+[\text{hC3dgC4bCR1}]}\cdots \\
\dfrac{1}{+[\text{hC3dgC4bC4BP}]+[\text{hC3bC3bH}]+[\text{hC3bC3bHL}]+[\text{hC3bC3bCR1}]+[\text{hC3biC3bH}]+[\text{hC3biC3bHL}]+[\text{hC3biC3bCR1}]+[\text{hiC3biC3bCR1}]}\cdots \\
\dfrac{1}{+[\text{hiC3bC3dgCR1}]+[\text{fC4bCR1}]+[\text{nfC4bC4BP}]+[\text{fC4bC4BP}]+[\text{fC4bC4bCR1}]+[\text{fC4bC4bC4BP}]+[\text{fC4bC4dCR1}]+[\text{fC4bC4dC4BP}]+[\text{hC4bCR1}]}\cdots \\
\dfrac{1}{+[\text{hC4bC4BP}]+[\text{hC4bC4bCR1}]+[\text{hC4bC4bC4BP}]+[\text{hC4bC4dCR1}]+[\text{hC4bC4dC4BP}]}\cdots
\end{array}\right)
$$

$$
+\left(\begin{array}{l}
\dfrac{k_{\text{catC3bH}}^{\text{FI}}[\text{I}][\text{fiC3bC4bC4BP}]}{K_{\text{mC3bH}}^{\text{FI}}+[\text{C3(H}_2\text{O)H}]+[\text{C3(H}_2\text{O)HL}]+[\text{fC3bH}]+[\text{fC3bHL}]+[\text{fC3bCR1}]+[\text{fiC3bCR1}]+[\text{IgGC3bC3bH}]+[\text{IgGC3biC3bH}]+[\text{IgGC3bC3bHL}]}\cdots \\
\dfrac{1}{+[\text{IgGC3biC3bHL}]+[\text{IgGC3bC3bCR1}]+[\text{IgGC3biC3bCR1}]+[\text{IgGiC3biC3bCR1}]+[\text{IgGiC3bC3dgCR1}]+[\text{fC3bC4bH}]+[\text{fC3bC4bHL}]}\cdots \\
\dfrac{1}{+[\text{fC3bC4bCR1}]+[\text{fC3bC4bC4BP}]+[\text{fC3bC4dH}]+[\text{fC3bC4dHL}]+[\text{fC3bC4dCR1}]+[\text{fiC3bC4dCR1}]+[\text{fiC3bC4bCR1}]+[\text{fiC3bC4bC4BP}]}\cdots \\
\dfrac{1}{+[\text{fC3dgC4bCR1}]+[\text{fC3dgC4bC4BP}]+[\text{fC3bC3bH}]+[\text{fC3bC3bHL}]+[\text{fC3bC3bCR1}]+[\text{fC3biC3bH}]+[\text{fC3biC3bHL}]+[\text{fC3biC3bCR1}]}\cdots \\
\dfrac{1}{+[\text{fiC3biC3bCR1}]+[\text{fiC3bC3dgCR1}]+[\text{hC3bH}]+[\text{hC3bHL}]+[\text{hC3bCR1}]+[\text{hiC3bCR1}]+[\text{hC3bC4bH}]+[\text{hC3bC4bHL}]+[\text{hC3bC4bCR1}]}\cdots \\
\dfrac{1}{+[\text{hC3bC4bC4BP}]+[\text{hC3bC4dH}]+[\text{hC3bC4dHL}]+[\text{hC3bC4dCR1}]+[\text{hiC3bC4dCR1}]+[\text{hiC3bC4bCR1}]+[\text{hiC3bC4bC4BP}]+[\text{hC3dgC4bCR1}]}\cdots \\
\dfrac{1}{+[\text{hC3dgC4bC4BP}]+[\text{hC3bC3bH}]+[\text{hC3bC3bHL}]+[\text{hC3bC3bCR1}]+[\text{hC3biC3bH}]+[\text{hC3biC3bHL}]+[\text{hC3biC3bCR1}]+[\text{hiC3biC3bCR1}]}\cdots \\
\dfrac{1}{+[\text{hiC3bC3dgCR1}]+[\text{fC4bCR1}]+[\text{nfC4bC4BP}]+[\text{fC4bC4BP}]+[\text{fC4bC4bCR1}]+[\text{fC4bC4bC4BP}]+[\text{fC4bC4dCR1}]+[\text{fC4bC4dC4BP}]+[\text{hC4bCR1}]}\cdots \\
\dfrac{1}{+[\text{hC4bC4BP}]+[\text{hC4bC4bCR1}]+[\text{hC4bC4bC4BP}]+[\text{hC4bC4dCR1}]+[\text{hC4bC4dC4BP}]}\cdots
\end{array}\right)
$$

$$
-k_{\text{iC3bC4dCR1}}^{+}[\text{fiC3bC4d}][\text{CR1}]+k_{\text{iC3bC4dCR1}}^{-}[\text{fiC3bC4dCR1}]
$$

S149

$$\frac{d[\mathrm{fiC3bC4dCR1}]}{dt} = k^{+}_{\mathrm{iC3bC4dCR1}}[\mathrm{fiC3bC4d}][\mathrm{CR1}] - k^{-}_{\mathrm{iC3bC4dCR1}}[\mathrm{fiC3bC4dCR1}]$$

$$-\left(\begin{array}{l} \dfrac{k^{\mathrm{FI}}_{\mathrm{catC3bH}}[\mathrm{I}][\mathrm{fiC3bC4dCR1}]}{K^{\mathrm{FI}}_{\mathrm{mC3bH}} + [\mathrm{C3(H_2O)H}] + [\mathrm{C3(H_2O)HL}] + [\mathrm{fC3bH}] + [\mathrm{fC3bHL}] + [\mathrm{fC3bCR1}] + [\mathrm{fiC3bCR1}] + [\mathrm{IgGC3bC3bH}] + [\mathrm{IgGC3biC3bH}] + [\mathrm{IgGC3bC3bHL}]} \cdots \\ \dfrac{1}{+[\mathrm{IgGC3biC3bHL}] + [\mathrm{IgGC3bC3bCR1}] + [\mathrm{IgGC3biC3bCR1}] + [\mathrm{IgGiC3biC3bCR1}] + [\mathrm{IgGiC3bC3dgCR1}] + [\mathrm{fC3bC4bH}] + [\mathrm{fC3bC4bHL}]} \cdots \\ \dfrac{1}{+[\mathrm{fC3bC4bCR1}] + [\mathrm{fC3bC4bC4BP}] + [\mathrm{fC3bC4dH}] + [\mathrm{fC3bC4dHL}] + [\mathrm{fC3bC4dCR1}] + [\mathrm{fiC3bC4dCR1}] + [\mathrm{fiC3bC4bCR1}] + [\mathrm{fiC3bC4bC4BP}]} \cdots \\ \dfrac{1}{+[\mathrm{fC3dgC4bCR1}] + [\mathrm{fC3dgC4bC4BP}] + [\mathrm{fC3bC3bH}] + [\mathrm{fC3bC3bHL}] + [\mathrm{fC3bC3bCR1}] + [\mathrm{fC3biC3bH}] + [\mathrm{fC3biC3bHL}] + [\mathrm{fC3biC3bCR1}]} \cdots \\ \dfrac{1}{+[\mathrm{fiC3biC3bCR1}] + [\mathrm{fiC3bC3dgCR1}] + [\mathrm{hC3bH}] + [\mathrm{hC3bHL}] + [\mathrm{hC3bCR1}] + [\mathrm{hiC3bCR1}] + [\mathrm{hC3bC4bH}] + [\mathrm{hC3bC4bHL}] + [\mathrm{hC3bC4bCR1}]} \cdots \\ \dfrac{1}{+[\mathrm{hC3bC4bC4BP}] + [\mathrm{hC3bC4dH}] + [\mathrm{hC3bC4dHL}] + [\mathrm{hC3bC4dCR1}] + [\mathrm{hiC3bC4dCR1}] + [\mathrm{hiC3bC4bCR1}] + [\mathrm{hiC3bC4bC4BP}] + [\mathrm{hC3dgC4bCR1}]} \cdots \\ \dfrac{1}{+[\mathrm{hC3dgC4bC4BP}] + [\mathrm{hC3bC3bH}] + [\mathrm{hC3bC3bHL}] + [\mathrm{hC3bC3bCR1}] + [\mathrm{hC3biC3bH}] + [\mathrm{hC3biC3bHL}] + [\mathrm{hC3biC3bCR1}] + [\mathrm{hiC3biC3bCR1}]} \cdots \\ \dfrac{1}{+[\mathrm{hiC3bC3dgCR1}] + [\mathrm{fC4bCR1}] + [\mathrm{nfC4bC4BP}] + [\mathrm{fC4bC4BP}] + [\mathrm{fC4bC4bCR1}] + [\mathrm{fC4bC4bC4BP}] + [\mathrm{fC4bC4dCR1}] + [\mathrm{fC4bC4dC4BP}] + [\mathrm{hC4bCR1}]} \cdots \\ \dfrac{1}{+[\mathrm{hC4bC4BP}] + [\mathrm{hC4bC4bCR1}] + [\mathrm{hC4bC4bC4BP}] + [\mathrm{hC4bC4dCR1}] + [\mathrm{hC4bC4dC4BP}]} \cdots \end{array} \right)$$

S150

$$\frac{d[\mathrm{fC3dgC4b}]}{dt} =$$

$$\left(\begin{array}{l} \dfrac{k^{\mathrm{FI}}_{\mathrm{catC3bH}}[\mathrm{I}][\mathrm{fiC3bC4bCR1}]}{K^{\mathrm{FI}}_{\mathrm{mC3bH}} + [\mathrm{C3(H_2O)H}] + [\mathrm{C3(H_2O)HL}] + [\mathrm{fC3bH}] + [\mathrm{fC3bHL}] + [\mathrm{fC3bCR1}] + [\mathrm{fiC3bCR1}] + [\mathrm{IgGC3bC3bH}] + [\mathrm{IgGC3biC3bH}] + [\mathrm{IgGC3bC3bHL}]} \cdots \\ \dfrac{1}{+[\mathrm{IgGC3biC3bHL}] + [\mathrm{IgGC3bC3bCR1}] + [\mathrm{IgGC3biC3bCR1}] + [\mathrm{IgGiC3biC3bCR1}] + [\mathrm{IgGiC3bC3dgCR1}] + [\mathrm{fC3bC4bH}] + [\mathrm{fC3bC4bHL}]} \cdots \\ \dfrac{1}{+[\mathrm{fC3bC4bCR1}] + [\mathrm{fC3bC4bC4BP}] + [\mathrm{fC3bC4dH}] + [\mathrm{fC3bC4dHL}] + [\mathrm{fC3bC4dCR1}] + [\mathrm{fiC3bC4dCR1}] + [\mathrm{fiC3bC4bCR1}] + [\mathrm{fiC3bC4bC4BP}]} \cdots \\ \dfrac{1}{+[\mathrm{fC3dgC4bCR1}] + [\mathrm{fC3dgC4bC4BP}] + [\mathrm{fC3bC3bH}] + [\mathrm{fC3bC3bHL}] + [\mathrm{fC3bC3bCR1}] + [\mathrm{fC3biC3bH}] + [\mathrm{fC3biC3bHL}] + [\mathrm{fC3biC3bCR1}]} \cdots \\ \dfrac{1}{+[\mathrm{fiC3biC3bCR1}] + [\mathrm{fiC3bC3dgCR1}] + [\mathrm{hC3bH}] + [\mathrm{hC3bHL}] + [\mathrm{hC3bCR1}] + [\mathrm{hiC3bCR1}] + [\mathrm{hC3bC4bH}] + [\mathrm{hC3bC4bHL}] + [\mathrm{hC3bC4bCR1}]} \cdots \\ \dfrac{1}{+[\mathrm{hC3bC4bC4BP}] + [\mathrm{hC3bC4dH}] + [\mathrm{hC3bC4dHL}] + [\mathrm{hC3bC4dCR1}] + [\mathrm{hiC3bC4dCR1}] + [\mathrm{hiC3bC4bCR1}] + [\mathrm{hiC3bC4bC4BP}] + [\mathrm{hC3dgC4bCR1}]} \cdots \\ \dfrac{1}{+[\mathrm{hC3dgC4bC4BP}] + [\mathrm{hC3bC3bH}] + [\mathrm{hC3bC3bHL}] + [\mathrm{hC3bC3bCR1}] + [\mathrm{hC3biC3bH}] + [\mathrm{hC3biC3bHL}] + [\mathrm{hC3biC3bCR1}] + [\mathrm{hiC3biC3bCR1}]} \cdots \\ \dfrac{1}{+[\mathrm{hiC3bC3dgCR1}] + [\mathrm{fC4bCR1}] + [\mathrm{nfC4bC4BP}] + [\mathrm{fC4bC4BP}] + [\mathrm{fC4bC4bCR1}] + [\mathrm{fC4bC4bC4BP}] + [\mathrm{fC4bC4dCR1}] + [\mathrm{fC4bC4dC4BP}] + [\mathrm{hC4bCR1}]} \cdots \\ \dfrac{1}{+[\mathrm{hC4bC4BP}] + [\mathrm{hC4bC4bCR1}] + [\mathrm{hC4bC4bC4BP}] + [\mathrm{hC4bC4dCR1}] + [\mathrm{hC4bC4dC4BP}]} \cdots \end{array} \right)$$

$$- k^{+}_{\mathrm{C3dgC4bCR1}}[\mathrm{fC3dgC4b}][\mathrm{CR1}] + k^{-}_{\mathrm{C3dgC4bCR1}}[\mathrm{fC3dgC4bCR1}] - k^{+}_{\mathrm{C4bC4BP}}[\mathrm{fC3dgC4b}][\mathrm{C4BP}] + k^{-}_{\mathrm{C4bC4BP}}[\mathrm{fC3dgC4bC4BP}]$$

S151

$$\frac{d[\mathrm{fC3dgC4bCR1}]}{dt} = k^{+}_{\mathrm{C3dgC4bCR1}}[\mathrm{fC3dgC4b}][\mathrm{CR1}] - k^{-}_{\mathrm{C3dgC4bCR1}}[\mathrm{fC3dgC4bCR1}]$$

$$-\left(\begin{array}{l}
\dfrac{k^{\mathrm{FI}}_{\mathrm{catC3bH}}[\mathrm{I}][\mathrm{fC3dgC4bCR1}]}{K^{\mathrm{FI}}_{\mathrm{mC3bH}}+[\mathrm{C3(H_2O)H}]+[\mathrm{C3(H_2O)HL}]+[\mathrm{fC3bH}]+[\mathrm{fC3bHL}]+[\mathrm{fC3bCR1}]+[\mathrm{fiC3bCR1}]+[\mathrm{IgGC3bC3bH}]+[\mathrm{IgGC3biC3bH}]+[\mathrm{IgGC3bC3bHL}]}\cdots \\
\dfrac{1}{+[\mathrm{IgGC3biC3bHL}]+[\mathrm{IgGC3bC3bCR1}]+[\mathrm{IgGC3biC3bCR1}]+[\mathrm{IgGiC3biC3bCR1}]+[\mathrm{IgGiC3bC3dgCR1}]+[\mathrm{fC3bC4bH}]+[\mathrm{fC3bC4bHL}]}\cdots \\
\dfrac{1}{+[\mathrm{fC3bC4bCR1}]+[\mathrm{fC3bC4bC4BP}]+[\mathrm{fC3bC4dH}]+[\mathrm{fC3bC4dHL}]+[\mathrm{fC3bC4dCR1}]+[\mathrm{fiC3bC4dCR1}]+[\mathrm{fiC3bC4bCR1}]+[\mathrm{fiC3bC4bC4BP}]}\cdots \\
\dfrac{1}{+[\mathrm{fC3dgC4bCR1}]+[\mathrm{fC3dgC4bC4BP}]+[\mathrm{fC3bC3bH}]+[\mathrm{fC3bC3bHL}]+[\mathrm{fC3bC3bCR1}]+[\mathrm{fC3biC3bH}]+[\mathrm{fC3biC3bHL}]+[\mathrm{fC3biC3bCR1}]}\cdots \\
\dfrac{1}{+[\mathrm{fiC3biC3bCR1}]+[\mathrm{fiC3bC3dgCR1}]+[\mathrm{hC3bH}]+[\mathrm{hC3bHL}]+[\mathrm{hC3bCR1}]+[\mathrm{hiC3bCR1}]+[\mathrm{hC3bC4bH}]+[\mathrm{hC3bC4bHL}]+[\mathrm{hC3bC4bCR1}]}\cdots \\
\dfrac{1}{+[\mathrm{hC3bC4bC4BP}]+[\mathrm{hC3bC4dH}]+[\mathrm{hC3bC4dHL}]+[\mathrm{hC3bC4dCR1}]+[\mathrm{hiC3bC4dCR1}]+[\mathrm{hiC3bC4bCR1}]+[\mathrm{hiC3bC4bC4BP}]+[\mathrm{hC3dgC4bCR1}]}\cdots \\
\dfrac{1}{+[\mathrm{hC3dgC4bC4BP}]+[\mathrm{hC3bC3bH}]+[\mathrm{hC3bC3bHL}]+[\mathrm{hC3bC3bCR1}]+[\mathrm{hC3biC3bH}]+[\mathrm{hC3biC3bHL}]+[\mathrm{hC3biC3bCR1}]+[\mathrm{hiC3biC3bCR1}]}\cdots \\
\dfrac{1}{+[\mathrm{hiC3bC3dgCR1}]+[\mathrm{fC4bCR1}]+[\mathrm{nfC4bC4BP}]+[\mathrm{fC4bC4BP}]+[\mathrm{fC4bC4bCR1}]+[\mathrm{fC4bC4bC4BP}]+[\mathrm{fC4bC4dCR1}]+[\mathrm{fC4bC4dC4BP}]+[\mathrm{hC4bCR1}]}\cdots \\
\dfrac{1}{+[\mathrm{hC4bC4BP}]+[\mathrm{hC4bC4bCR1}]+[\mathrm{hC4bC4bC4BP}]+[\mathrm{hC4bC4dCR1}]+[\mathrm{hC4bC4dC4BP}]}\cdots
\end{array}\right)$$

S152

$$\frac{d[\mathrm{fC3dgC4bC4BP}]}{dt} = k^{+}_{\mathrm{C4bC4BP}}[\mathrm{fC3dgC4b}][\mathrm{C4BP}] - k^{-}_{\mathrm{C4bC4BP}}[\mathrm{fC3dgC4bC4BP}]$$

$$-\left(\begin{array}{l}
\dfrac{k^{\mathrm{FI}}_{\mathrm{catC3bH}}[\mathrm{I}][\mathrm{fC3dgC4bC4BP}]}{K^{\mathrm{FI}}_{\mathrm{mC3bH}}+[\mathrm{C3(H_2O)H}]+[\mathrm{C3(H_2O)HL}]+[\mathrm{fC3bH}]+[\mathrm{fC3bHL}]+[\mathrm{fC3bCR1}]+[\mathrm{fiC3bCR1}]+[\mathrm{IgGC3bC3bH}]+[\mathrm{IgGC3biC3bH}]+[\mathrm{IgGC3bC3bHL}]}\cdots \\
\dfrac{1}{+[\mathrm{IgGC3biC3bHL}]+[\mathrm{IgGC3bC3bCR1}]+[\mathrm{IgGC3biC3bCR1}]+[\mathrm{IgGiC3biC3bCR1}]+[\mathrm{IgGiC3bC3dgCR1}]+[\mathrm{fC3bC4bH}]+[\mathrm{fC3bC4bHL}]}\cdots \\
\dfrac{1}{+[\mathrm{fC3bC4bCR1}]+[\mathrm{fC3bC4bC4BP}]+[\mathrm{fC3bC4dH}]+[\mathrm{fC3bC4dHL}]+[\mathrm{fC3bC4dCR1}]+[\mathrm{fiC3bC4dCR1}]+[\mathrm{fiC3bC4bCR1}]+[\mathrm{fiC3bC4bC4BP}]}\cdots \\
\dfrac{1}{+[\mathrm{fC3dgC4bCR1}]+[\mathrm{fC3dgC4bC4BP}]+[\mathrm{fC3bC3bH}]+[\mathrm{fC3bC3bHL}]+[\mathrm{fC3bC3bCR1}]+[\mathrm{fC3biC3bH}]+[\mathrm{fC3biC3bHL}]+[\mathrm{fC3biC3bCR1}]}\cdots \\
\dfrac{1}{+[\mathrm{fiC3biC3bCR1}]+[\mathrm{fiC3bC3dgCR1}]+[\mathrm{hC3bH}]+[\mathrm{hC3bHL}]+[\mathrm{hC3bCR1}]+[\mathrm{hiC3bCR1}]+[\mathrm{hC3bC4bH}]+[\mathrm{hC3bC4bHL}]+[\mathrm{hC3bC4bCR1}]}\cdots \\
\dfrac{1}{+[\mathrm{hC3bC4bC4BP}]+[\mathrm{hC3bC4dH}]+[\mathrm{hC3bC4dHL}]+[\mathrm{hC3bC4dCR1}]+[\mathrm{hiC3bC4dCR1}]+[\mathrm{hiC3bC4bCR1}]+[\mathrm{hiC3bC4bC4BP}]+[\mathrm{hC3dgC4bCR1}]}\cdots \\
\dfrac{1}{+[\mathrm{hC3dgC4bC4BP}]+[\mathrm{hC3bC3bH}]+[\mathrm{hC3bC3bHL}]+[\mathrm{hC3bC3bCR1}]+[\mathrm{hC3biC3bH}]+[\mathrm{hC3biC3bHL}]+[\mathrm{hC3biC3bCR1}]+[\mathrm{hiC3biC3bCR1}]}\cdots \\
\dfrac{1}{+[\mathrm{hiC3bC3dgCR1}]+[\mathrm{fC4bCR1}]+[\mathrm{nfC4bC4BP}]+[\mathrm{fC4bC4BP}]+[\mathrm{fC4bC4bCR1}]+[\mathrm{fC4bC4bC4BP}]+[\mathrm{fC4bC4dCR1}]+[\mathrm{fC4bC4dC4BP}]+[\mathrm{hC4bCR1}]}\cdots \\
\dfrac{1}{+[\mathrm{hC4bC4BP}]+[\mathrm{hC4bC4bCR1}]+[\mathrm{hC4bC4bC4BP}]+[\mathrm{hC4bC4dCR1}]+[\mathrm{hC4bC4dC4BP}]}\cdots
\end{array}\right)$$

S153

$$\frac{d[\mathrm{fC3dgC4d}]}{dt}=$$

$$\left(\frac{k_{\mathrm{catC3bH}}^{\mathrm{FI}}[\mathrm{I}][\mathrm{fiC3bC4dCR1}]}{K_{\mathrm{mC3bH}}^{\mathrm{FI}}+[\mathrm{C3(H_2O)H}]+[\mathrm{C3(H_2O)HL}]+[\mathrm{fC3bH}]+[\mathrm{fC3bHL}]+[\mathrm{fC3bCR1}]+[\mathrm{fiC3bCR1}]+[\mathrm{IgGC3bC3bH}]+[\mathrm{IgGC3biC3bH}]+[\mathrm{IgGC3bC3bHL}]}\cdots\right.$$

$$\frac{1}{+[\mathrm{IgGC3biC3bHL}]+[\mathrm{IgGC3bC3bCR1}]+[\mathrm{IgGC3biC3bCR1}]+[\mathrm{IgGiC3biC3bCR1}]+[\mathrm{IgGiC3bC3dgCR1}]+[\mathrm{fC3bC4bH}]+[\mathrm{fC3bC4bHL}]}\cdots$$

$$\frac{1}{+[\mathrm{fC3bC4bCR1}]+[\mathrm{fC3bC4bC4BP}]+[\mathrm{fC3bC4dH}]+[\mathrm{fC3bC4dHL}]+[\mathrm{fC3bC4dCR1}]+[\mathrm{fiC3bC4dCR1}]+[\mathrm{fiC3bC4bCR1}]+[\mathrm{fiC3bC4bC4BP}]}\cdots$$

$$\frac{1}{+[\mathrm{fC3dgC4bCR1}]+[\mathrm{fC3dgC4bC4BP}]+[\mathrm{fC3bC3bH}]+[\mathrm{fC3bC3bHL}]+[\mathrm{fC3bC3bCR1}]+[\mathrm{fC3biC3bH}]+[\mathrm{fC3biC3bHL}]+[\mathrm{fC3biC3bCR1}]}\cdots$$

$$\frac{1}{+[\mathrm{fiC3biC3bCR1}]+[\mathrm{fiC3bC3dgCR1}]+[\mathrm{hC3bH}]+[\mathrm{hC3bHL}]+[\mathrm{hC3bCR1}]+[\mathrm{hiC3bCR1}]+[\mathrm{hC3bC4bH}]+[\mathrm{hC3bC4bHL}]+[\mathrm{hC3bC4bCR1}]}\cdots$$

$$\frac{1}{+[\mathrm{hC3bC4bC4BP}]+[\mathrm{hC3bC4dH}]+[\mathrm{hC3bC4dHL}]+[\mathrm{hC3bC4dCR1}]+[\mathrm{hiC3bC4dCR1}]+[\mathrm{hiC3bC4bCR1}]+[\mathrm{hiC3bC4bC4BP}]+[\mathrm{hC3dgC4bCR1}]}\cdots$$

$$\frac{1}{+[\mathrm{hC3dgC4bC4BP}]+[\mathrm{hC3bC3bH}]+[\mathrm{hC3bC3bHL}]+[\mathrm{hC3bC3bCR1}]+[\mathrm{hC3biC3bH}]+[\mathrm{hC3biC3bHL}]+[\mathrm{hC3biC3bCR1}]+[\mathrm{hiC3biC3bCR1}]}\cdots$$

$$\frac{1}{+[\mathrm{hiC3bC3dgCR1}]+[\mathrm{fC4bCR1}]+[\mathrm{nfC4bC4BP}]+[\mathrm{fC4bC4BP}]+[\mathrm{fC4bC4bCR1}]+[\mathrm{fC4bC4bC4BP}]+[\mathrm{fC4bC4dCR1}]+[\mathrm{fC4bC4dC4BP}]+[\mathrm{hC4bCR1}]}\cdots$$

$$\left.\frac{1}{+[\mathrm{hC4bC4BP}]+[\mathrm{hC4bC4bCR1}]+[\mathrm{hC4bC4bC4BP}]+[\mathrm{hC4bC4dCR1}]+[\mathrm{hC4bC4dC4BP}]}\cdots\right)$$

$$+\left(\frac{k_{\mathrm{catC3bH}}^{\mathrm{FI}}[\mathrm{I}][\mathrm{fC3dgC4bCR1}]}{K_{\mathrm{mC3bH}}^{\mathrm{FI}}+[\mathrm{C3(H_2O)H}]+[\mathrm{C3(H_2O)HL}]+[\mathrm{fC3bH}]+[\mathrm{fC3bHL}]+[\mathrm{fC3bCR1}]+[\mathrm{fiC3bCR1}]+[\mathrm{IgGC3bC3bH}]+[\mathrm{IgGC3biC3bH}]+[\mathrm{IgGC3bC3bHL}]}\cdots\right.$$

$$\frac{1}{+[\mathrm{IgGC3biC3bHL}]+[\mathrm{IgGC3bC3bCR1}]+[\mathrm{IgGC3biC3bCR1}]+[\mathrm{IgGiC3biC3bCR1}]+[\mathrm{IgGiC3bC3dgCR1}]+[\mathrm{fC3bC4bH}]+[\mathrm{fC3bC4bHL}]}\cdots$$

$$\frac{1}{+[\mathrm{fC3bC4bCR1}]+[\mathrm{fC3bC4bC4BP}]+[\mathrm{fC3bC4dH}]+[\mathrm{fC3bC4dHL}]+[\mathrm{fC3bC4dCR1}]+[\mathrm{fiC3bC4dCR1}]+[\mathrm{fiC3bC4bCR1}]+[\mathrm{fiC3bC4bC4BP}]}\cdots$$

$$\frac{1}{+[\mathrm{fC3dgC4bCR1}]+[\mathrm{fC3dgC4bC4BP}]+[\mathrm{fC3bC3bH}]+[\mathrm{fC3bC3bHL}]+[\mathrm{fC3bC3bCR1}]+[\mathrm{fC3biC3bH}]+[\mathrm{fC3biC3bHL}]+[\mathrm{fC3biC3bCR1}]}\cdots$$

$$\frac{1}{+[\mathrm{fiC3biC3bCR1}]+[\mathrm{fiC3bC3dgCR1}]+[\mathrm{hC3bH}]+[\mathrm{hC3bHL}]+[\mathrm{hC3bCR1}]+[\mathrm{hiC3bCR1}]+[\mathrm{hC3bC4bH}]+[\mathrm{hC3bC4bHL}]+[\mathrm{hC3bC4bCR1}]}\cdots$$

$$\frac{1}{+[\mathrm{hC3bC4bC4BP}]+[\mathrm{hC3bC4dH}]+[\mathrm{hC3bC4dHL}]+[\mathrm{hC3bC4dCR1}]+[\mathrm{hiC3bC4dCR1}]+[\mathrm{hiC3bC4bCR1}]+[\mathrm{hiC3bC4bC4BP}]+[\mathrm{hC3dgC4bCR1}]}\cdots$$

$$\frac{1}{+[\mathrm{hC3dgC4bC4BP}]+[\mathrm{hC3bC3bH}]+[\mathrm{hC3bC3bHL}]+[\mathrm{hC3bC3bCR1}]+[\mathrm{hC3biC3bH}]+[\mathrm{hC3biC3bHL}]+[\mathrm{hC3biC3bCR1}]+[\mathrm{hiC3biC3bCR1}]}\cdots$$

$$\frac{1}{+[\mathrm{hiC3bC3dgCR1}]+[\mathrm{fC4bCR1}]+[\mathrm{nfC4bC4BP}]+[\mathrm{fC4bC4BP}]+[\mathrm{fC4bC4bCR1}]+[\mathrm{fC4bC4bC4BP}]+[\mathrm{fC4bC4dCR1}]+[\mathrm{fC4bC4dC4BP}]+[\mathrm{hC4bCR1}]}\cdots$$

$$\left.\frac{1}{+[\mathrm{hC4bC4BP}]+[\mathrm{hC4bC4bCR1}]+[\mathrm{hC4bC4bC4BP}]+[\mathrm{hC4bC4dCR1}]+[\mathrm{hC4bC4dC4BP}]}\cdots\right)$$

$$+\left(\frac{k^{FI}_{catC3bH}[I][fC3dgC4bC4BP]}{K^{FI}_{mC3bH}+[C3(H_2O)H]+[C3(H_2O)HL]+[fC3bH]+[fC3bHL]+[fC3bCR1]+[fiC3bCR1]+[IgGC3bC3bH]+[IgGC3biC3bH]+[IgGC3bC3bHL]} \cdots \right.$$

$$\frac{1}{+[IgGC3biC3bHL]+[IgGC3bC3bCR1]+[IgGC3biC3bCR1]+[IgGiC3biC3bCR1]+[IgGiC3bC3dgCR1]+[fC3bC4bH]+[fC3bC4bHL]} \cdots$$

$$\frac{1}{+[fC3bC4bCR1]+[fC3bC4bC4BP]+[fC3bC4dH]+[fC3bC4dHL]+[fC3bC4dCR1]+[fiC3bC4dCR1]+[fiC3bC4bCR1]+[fiC3bC4bC4BP]} \cdots$$

$$\frac{1}{+[fC3dgC4bCR1]+[fC3dgC4bC4BP]+[fC3bC3bH]+[fC3bC3bHL]+[fC3bC3bCR1]+[fC3biC3bH]+[fC3biC3bHL]+[fC3biC3bCR1]} \cdots$$

$$\frac{1}{+[fiC3biC3bCR1]+[fiC3bC3dgCR1]+[hC3bH]+[hC3bHL]+[hC3bCR1]+[hiC3bCR1]+[hC3bC4bH]+[hC3bC4bHL]+[hC3bC4bCR1]} \cdots$$

$$\frac{1}{+[hC3bC4bC4BP]+[hC3bC4dH]+[hC3bC4dHL]+[hC3bC4dCR1]+[hiC3bC4dCR1]+[hiC3bC4bCR1]+[hiC3bC4bC4BP]+[hC3dgC4bCR1]} \cdots$$

$$\frac{1}{+[hC3dgC4bC4BP]+[hC3bC3bH]+[hC3bC3bHL]+[hC3bC3bCR1]+[hC3biC3bH]+[hC3biC3bHL]+[hC3biC3bCR1]+[hiC3biC3bCR1]} \cdots$$

$$\frac{1}{+[hiC3bC3dgCR1]+[fC4bCR1]+[nfC4bC4BP]+[fC4bC4BP]+[fC4bC4bCR1]+[fC4bC4bC4BP]+[fC4bC4dCR1]+[fC4bC4dC4BP]+[hC4bCR1]} \cdots$$

$$\left.\frac{1}{+[hC4bC4BP]+[hC4bC4bCR1]+[hC4bC4bC4BP]+[hC4bC4dCR1]+[hC4bC4dC4BP]} \cdots \right)$$

S154

$$\frac{d[fC3bC4bBbH]}{dt}=k^{+}_{C3bH}[fC3bC4bBb][H]-k^{-}_{C3bH}[fC3bC4bBbH]-k^{-}_{C3bBbH_{decay}}[fC3bC4bBbH]$$

S155

$$\frac{d[fC3bC4bBbHL]}{dt}=k^{+}_{C3bHL}[fC3bC4bBb][HL]-k^{-}_{C3bHL}[fC3bC4bBbHL]-k^{-}_{C3bBbHL_{decay}}[fC3bC4bBbHL]$$

S156

$$\frac{d[fC3bC4bBbCR1]}{dt}=k^{+}_{C3bC4bCR1}[fC3bC4bBb][CR1]-k^{-}_{C3bC4bCR1}[fC3bC4bBbCR1]-k^{-}_{C3bBbCR1_{decay}}[fC3bC4bBbCR1]$$

S157

$$\frac{d[\text{fC3bC3bH}]}{dt} = k^{+}_{\text{C3bH}}[\text{fC3bC3b}][\text{H}] - k^{-}_{\text{C3bH}}[\text{fC3bC3bH}]$$

$$-\left(\begin{aligned}
&\frac{k^{\text{FI}}_{\text{catC3bH}}[\text{I}][\text{fC3bC3bH}]}{K^{\text{FI}}_{\text{mC3bH}} + [\text{C3(H}_2\text{O)H}] + [\text{C3(H}_2\text{O)HL}] + [\text{fC3bH}] + [\text{fC3bHL}] + [\text{fC3bCR1}] + [\text{fiC3bCR1}] + [\text{IgGC3bC3bH}] + [\text{IgGC3biC3bH}] + [\text{IgGC3bC3bHL}]}\cdots\\
&\frac{1}{+[\text{IgGC3biC3bHL}] + [\text{IgGC3bC3bCR1}] + [\text{IgGC3biC3bCR1}] + [\text{IgGiC3biC 3bCR1}] + [\text{IgGiC3bC3dgCR1}] + [\text{fC3bC4bH}] + [\text{fC3bC4bHL}]}\cdots\\
&\frac{1}{+[\text{fC3bC4bCR1}] + [\text{fC3bC4bC4BP}] + [\text{fC3bC4dH}] + [\text{fC3bC4dHL}] + [\text{fC3bC4dCR1}] + [\text{fiC3bC4dCR1}] + [\text{fiC3bC4bCR1}] + [\text{fiC3bC4bC4BP}]}\cdots\\
&\frac{1}{+[\text{fC3dgC4bCR1}] + [\text{fC3dgC4bC4BP}] + [\text{fC3bC3bH}] + [\text{fC3bC3bHL}] + [\text{fC3bC3bCR1}] + [\text{fC3biC3bH}] + [\text{fC3biC3bHL}] + [\text{fC3biC3bCR1}]}\cdots\\
&\frac{1}{+[\text{fiC3biC3bCR1}] + [\text{fiC3bC3dgCR1}] + [\text{hC3bH}] + [\text{hC3bHL}] + [\text{hC3bCR1}] + [\text{hiC3bCR1}] + [\text{hC3bC4bH}] + [\text{hC3bC4bHL}] + [\text{hC3bC4bCR1}]}\cdots\\
&\frac{1}{+[\text{hC3bC4bC4BP}] + [\text{hC3bC4dH}] + [\text{hC3bC4dHL}] + [\text{hC3bC4dCR1}] + [\text{hiC3bC4dCR1}] + [\text{hiC3bC4bCR1}] + [\text{hiC3bC4bC4BP}] + [\text{hC3dgC4bCR1}]}\cdots\\
&\frac{1}{+[\text{hC3dgC4bC4BP}] + [\text{hC3bC3bH}] + [\text{hC3bC3bHL}] + [\text{hC3bC3bCR1}] + [\text{hC3biC3bH}] + [\text{hC3biC3bHL}] + [\text{hC3biC3bCR1}] + [\text{hiC3biC3bCR1}]}\cdots\\
&\frac{1}{+[\text{hiC3bC3dgCR1}] + [\text{fC4bCR1}] + [\text{nfC4bC4BP}] + [\text{fC4bC4BP}] + [\text{fC4bC4bCR1}] + [\text{fC4bC4bC4BP}] + [\text{fC4bC4dCR1}] + [\text{fC4bC4dC4BP}] + [\text{hC4bCR1}]}\cdots\\
&\frac{1}{+[\text{hC4bC4BP}] + [\text{hC4bC4bCR1}] + [\text{hC4bC4bC4BP}] + [\text{hC4bC4dCR1}] + [\text{hC4bC4dC4BP}]}\cdots
\end{aligned}\right)$$

S158

$$\frac{d[\text{fC3bC3bHL}]}{dt} = k^{+}_{\text{C3bHL}}[\text{fC3bC3b}][\text{HL}] - k^{-}_{\text{C3bHL}}[\text{fC3bC3bHL}]$$

$$-\left(\begin{aligned}
&\frac{k^{\text{FI}}_{\text{catC3bH}}[\text{I}][\text{fC3bC3bHl}]}{K^{\text{FI}}_{\text{mC3bH}} + [\text{C3(H}_2\text{O)H}] + [\text{C3(H}_2\text{O)HL}] + [\text{fC3bH}] + [\text{fC3bHL}] + [\text{fC3bCR1}] + [\text{fiC3bCR1}] + [\text{IgGC3bC3bH}] + [\text{IgGC3biC3bH}] + [\text{IgGC3bC3bHL}]}\cdots\\
&\frac{1}{+[\text{IgGC3biC3bHL}] + [\text{IgGC3bC3bCR1}] + [\text{IgGC3biC3bCR1}] + [\text{IgGiC3biC 3bCR1}] + [\text{IgGiC3bC3dgCR1}] + [\text{fC3bC4bH}] + [\text{fC3bC4bHL}]}\cdots\\
&\frac{1}{+[\text{fC3bC4bCR1}] + [\text{fC3bC4bC4BP}] + [\text{fC3bC4dH}] + [\text{fC3bC4dHL}] + [\text{fC3bC4dCR1}] + [\text{fiC3bC4dCR1}] + [\text{fiC3bC4bCR1}] + [\text{fiC3bC4bC4BP}]}\cdots\\
&\frac{1}{+[\text{fC3dgC4bCR1}] + [\text{fC3dgC4bC4BP}] + [\text{fC3bC3bH}] + [\text{fC3bC3bHL}] + [\text{fC3bC3bCR1}] + [\text{fC3biC3bH}] + [\text{fC3biC3bHL}] + [\text{fC3biC3bCR1}]}\cdots\\
&\frac{1}{+[\text{fiC3biC3bCR1}] + [\text{fiC3bC3dgCR1}] + [\text{hC3bH}] + [\text{hC3bHL}] + [\text{hC3bCR1}] + [\text{hiC3bCR1}] + [\text{hC3bC4bH}] + [\text{hC3bC4bHL}] + [\text{hC3bC4bCR1}]}\cdots\\
&\frac{1}{+[\text{hC3bC4bC4BP}] + [\text{hC3bC4dH}] + [\text{hC3bC4dHL}] + [\text{hC3bC4dCR1}] + [\text{hiC3bC4dCR1}] + [\text{hiC3bC4bCR1}] + [\text{hiC3bC4bC4BP}] + [\text{hC3dgC4bCR1}]}\cdots\\
&\frac{1}{+[\text{hC3dgC4bC4BP}] + [\text{hC3bC3bH}] + [\text{hC3bC3bHL}] + [\text{hC3bC3bCR1}] + [\text{hC3biC3bH}] + [\text{hC3biC3bHL}] + [\text{hC3biC3bCR1}] + [\text{hiC3biC3bCR1}]}\cdots\\
&\frac{1}{+[\text{hiC3bC3dgCR1}] + [\text{fC4bCR1}] + [\text{nfC4bC4BP}] + [\text{fC4bC4BP}] + [\text{fC4bC4bCR1}] + [\text{fC4bC4bC4BP}] + [\text{fC4bC4dCR1}] + [\text{fC4bC4dC4BP}] + [\text{hC4bCR1}]}\cdots\\
&\frac{1}{+[\text{hC4bC4BP}] + [\text{hC4bC4bCR1}] + [\text{hC4bC4bC4BP}] + [\text{hC4bC4dCR1}] + [\text{hC4bC4dC4BP}]}\cdots
\end{aligned}\right)$$

S159

$$\frac{d[\text{fC3bC3bCR1}]}{dt} = k^{+}_{\text{C3bC3bCR1}}[\text{fC3bC3b}][\text{CR1}] - k^{-}_{\text{C3bC3bCR1}}[\text{fC3bC3bCR1}]$$

$$-\left(\begin{array}{l}
\dfrac{k^{\text{FI}}_{\text{catC3bH}}[\text{I}][\text{fC3bC3bCR1}]}{K^{\text{FI}}_{\text{mC3bH}} + [\text{C3(H}_2\text{O)H}] + [\text{C3(H}_2\text{O)HL}] + [\text{fC3bH}] + [\text{fC3bHL}] + [\text{fC3bCR1}] + [\text{fiC3bCR1}] + [\text{IgGC3bC3bH}] + [\text{IgGC3biC3bH}] + [\text{IgGC3bC3bHL}]} \cdots \\
\dfrac{1}{+[\text{IgGC3biC3bHL}] + [\text{IgGC3bC3bCR1}] + [\text{IgGC3biC3bCR1}] + [\text{IgGiC3biC3bCR1}] + [\text{IgGiC3bC3dgCR1}] + [\text{fC3bC4bH}] + [\text{fC3bC4bHL}]} \cdots \\
\dfrac{1}{+[\text{fC3bC4bCR1}] + [\text{fC3bC4bC4BP}] + [\text{fC3bC4dH}] + [\text{fC3bC4dHL}] + [\text{fC3bC4dCR1}] + [\text{fiC3bC4dCR1}] + [\text{fiC3bC4bCR1}] + [\text{fiC3bC4bC4BP}]} \cdots \\
\dfrac{1}{+[\text{fC3dgC4bCR1}] + [\text{fC3dgC4bC4BP}] + [\text{fC3bC3bH}] + [\text{fC3bC3bHL}] + [\text{fC3bC3bCR1}] + [\text{fC3biC3bH}] + [\text{fC3biC3bHL}] + [\text{fC3biC3bCR1}]} \cdots \\
\dfrac{1}{+[\text{fiC3biC3bCR1}] + [\text{fiC3bC3dgCR1}] + [\text{hC3bH}] + [\text{hC3bHL}] + [\text{hC3bCR1}] + [\text{hiC3bCR1}] + [\text{hC3bC4bH}] + [\text{hC3bC4bHL}] + [\text{hC3bC4bCR1}]} \cdots \\
\dfrac{1}{+[\text{hC3bC4bC4BP}] + [\text{hC3bC4dH}] + [\text{hC3bC4dHL}] + [\text{hC3bC4dCR1}] + [\text{hiC3bC4dCR1}] + [\text{hiC3bC4bCR1}] + [\text{hiC3bC4bC4BP}] + [\text{hC3dgC4bCR1}]} \cdots \\
\dfrac{1}{+[\text{hC3dgC4bC4BP}] + [\text{hC3bC3bH}] + [\text{hC3bC3bHL}] + [\text{hC3bC3bCR1}] + [\text{hC3biC3bH}] + [\text{hC3biC3bHL}] + [\text{hC3biC3bCR1}] + [\text{hiC3biC3bCR1}]} \cdots \\
\dfrac{1}{+[\text{hiC3bC3dgCR1}] + [\text{fC4bCR1}] + [\text{nfC4bC4BP}] + [\text{fC4bC4BP}] + [\text{fC4bC4bCR1}] + [\text{fC4bC4bC4BP}] + [\text{fC4bC4dCR1}] + [\text{fC4bC4dC4BP}] + [\text{hC4bCR1}]} \cdots \\
\dfrac{1}{+[\text{hC4bC4BP}] + [\text{hC4bC4bCR1}] + [\text{hC4bC4bC4BP}] + [\text{hC4bC4dCR1}] + [\text{hC4bC4dC4BP}]} \cdots
\end{array} \right)$$

S160

$$\frac{d[\text{fC3biC3b}]}{dt} =$$

$$\left(\begin{array}{l}
\dfrac{k^{\text{FI}}_{\text{catC3bH}}[\text{I}][\text{fC3bC3bH}]}{K^{\text{FI}}_{\text{mC3bH}} + [\text{C3(H}_2\text{O)H}] + [\text{C3(H}_2\text{O)HL}] + [\text{fC3bH}] + [\text{fC3bHL}] + [\text{fC3bCR1}] + [\text{fiC3bCR1}] + [\text{IgGC3bC3bH}] + [\text{IgGC3biC3bH}] + [\text{IgGC3bC3bHL}]} \cdots \\
\dfrac{1}{+[\text{IgGC3biC3bHL}] + [\text{IgGC3bC3bCR1}] + [\text{IgGC3biC3bCR1}] + [\text{IgGiC3biC3bCR1}] + [\text{IgGiC3bC3dgCR1}] + [\text{fC3bC4bH}] + [\text{fC3bC4bHL}]} \cdots \\
\dfrac{1}{+[\text{fC3bC4bCR1}] + [\text{fC3bC4bC4BP}] + [\text{fC3bC4dH}] + [\text{fC3bC4dHL}] + [\text{fC3bC4dCR1}] + [\text{fiC3bC4dCR1}] + [\text{fiC3bC4bCR1}] + [\text{fiC3bC4bC4BP}]} \cdots \\
\dfrac{1}{+[\text{fC3dgC4bCR1}] + [\text{fC3dgC4bC4BP}] + [\text{fC3bC3bH}] + [\text{fC3bC3bHL}] + [\text{fC3bC3bCR1}] + [\text{fC3biC3bH}] + [\text{fC3biC3bHL}] + [\text{fC3biC3bCR1}]} \cdots \\
\dfrac{1}{+[\text{fiC3biC3bCR1}] + [\text{fiC3bC3dgCR1}] + [\text{hC3bH}] + [\text{hC3bHL}] + [\text{hC3bCR1}] + [\text{hiC3bCR1}] + [\text{hC3bC4bH}] + [\text{hC3bC4bHL}] + [\text{hC3bC4bCR1}]} \cdots \\
\dfrac{1}{+[\text{hC3bC4bC4BP}] + [\text{hC3bC4dH}] + [\text{hC3bC4dHL}] + [\text{hC3bC4dCR1}] + [\text{hiC3bC4dCR1}] + [\text{hiC3bC4bCR1}] + [\text{hiC3bC4bC4BP}] + [\text{hC3dgC4bCR1}]} \cdots \\
\dfrac{1}{+[\text{hC3dgC4bC4BP}] + [\text{hC3bC3bH}] + [\text{hC3bC3bHL}] + [\text{hC3bC3bCR1}] + [\text{hC3biC3bH}] + [\text{hC3biC3bHL}] + [\text{hC3biC3bCR1}] + [\text{hiC3biC3bCR1}]} \cdots \\
\dfrac{1}{+[\text{hiC3bC3dgCR1}] + [\text{fC4bCR1}] + [\text{nfC4bC4BP}] + [\text{fC4bC4BP}] + [\text{fC4bC4bCR1}] + [\text{fC4bC4bC4BP}] + [\text{fC4bC4dCR1}] + [\text{fC4bC4dC4BP}] + [\text{hC4bCR1}]} \cdots \\
\dfrac{1}{+[\text{hC4bC4BP}] + [\text{hC4bC4bCR1}] + [\text{hC4bC4bC4BP}] + [\text{hC4bC4dCR1}] + [\text{hC4bC4dC4BP}]} \cdots
\end{array} \right)$$

$$+\left(\frac{k^{\mathrm{FI}}_{\mathrm{catC3bH}}[\mathrm{I}][\mathrm{fC3bC3bHL}]}{K^{\mathrm{FI}}_{\mathrm{mC3bH}}+[\mathrm{C3(H_2O)H}]+[\mathrm{C3(H_2O)HL}]+[\mathrm{fC3bH}]+[\mathrm{fC3bHL}]+[\mathrm{fC3bCR1}]+[\mathrm{fiC3bCR1}]+[\mathrm{IgGC3bC3bH}]+[\mathrm{IgGC3biC3bH}]+[\mathrm{IgGC3bC3bHL}]}\cdots\right.$$

$$\frac{1}{+[\mathrm{IgGC3biC3bHL}]+[\mathrm{IgGC3bC3bCR1}]+[\mathrm{IgGC3biC3bCR1}]+[\mathrm{IgGiC3biC3bCR1}]+[\mathrm{IgGiC3bC3dgCR1}]+[\mathrm{fC3bC4bH}]+[\mathrm{fC3bC4bHL}]}\cdots$$

$$\frac{1}{+[\mathrm{fC3bC4bCR1}]+[\mathrm{fC3bC4bC4BP}]+[\mathrm{fC3bC4dH}]+[\mathrm{fC3bC4dHL}]+[\mathrm{fC3bC4dCR1}]+[\mathrm{fiC3bC4dCR1}]+[\mathrm{fiC3bC4bCR1}]+[\mathrm{fiC3bC4bC4BP}]}\cdots$$

$$\frac{1}{+[\mathrm{fC3dgC4bCR1}]+[\mathrm{fC3dgC4bC4BP}]+[\mathrm{fC3bC3bH}]+[\mathrm{fC3bC3bHL}]+[\mathrm{fC3bC3bCR1}]+[\mathrm{fC3biC3bH}]+[\mathrm{fC3biC3bHL}]+[\mathrm{fC3biC3bCR1}]}\cdots$$

$$\frac{1}{+[\mathrm{fiC3biC3bCR1}]+[\mathrm{fiC3bC3dgCR1}]+[\mathrm{hC3bH}]+[\mathrm{hC3bHL}]+[\mathrm{hC3bCR1}]+[\mathrm{hiC3bCR1}]+[\mathrm{hC3bC4bH}]+[\mathrm{hC3bC4bHL}]+[\mathrm{hC3bC4bCR1}]}\cdots$$

$$\frac{1}{+[\mathrm{hC3bC4bC4BP}]+[\mathrm{hC3bC4dH}]+[\mathrm{hC3bC4dHL}]+[\mathrm{hC3bC4dCR1}]+[\mathrm{hiC3bC4dCR1}]+[\mathrm{hiC3bC4bCR1}]+[\mathrm{hiC3bC4bC4BP}]+[\mathrm{hC3dgC4bCR1}]}\cdots$$

$$\frac{1}{+[\mathrm{hC3dgC4bC4BP}]+[\mathrm{hC3bC3bH}]+[\mathrm{hC3bC3bHL}]+[\mathrm{hC3bC3bCR1}]+[\mathrm{hC3biC3bH}]+[\mathrm{hC3biC3bHL}]+[\mathrm{hC3biC3bCR1}]+[\mathrm{hiC3biC3bCR1}]}\cdots$$

$$\frac{1}{+[\mathrm{hiC3bC3dgCR1}]+[\mathrm{fC4bCR1}]+[\mathrm{nfC4bC4BP}]+[\mathrm{fC4bC4BP}]+[\mathrm{fC4bC4bCR1}]+[\mathrm{fC4bC4bC4BP}]+[\mathrm{fC4bC4dCR1}]+[\mathrm{fC4bC4dC4BP}]+[\mathrm{hC4bCR1}]}\cdots$$

$$\left.\frac{1}{+[\mathrm{hC4bC4BP}]+[\mathrm{hC4bC4bCR1}]+[\mathrm{hC4bC4bC4BP}]+[\mathrm{hC4bC4dCR1}]+[\mathrm{hC4bC4dC4BP}]}\cdots\right)$$

$$+\left(\frac{k^{\mathrm{FI}}_{\mathrm{catC3bH}}[\mathrm{I}][\mathrm{fC3bC3bCR1}]}{K^{\mathrm{FI}}_{\mathrm{mC3bH}}+[\mathrm{C3(H_2O)H}]+[\mathrm{C3(H_2O)HL}]+[\mathrm{fC3bH}]+[\mathrm{fC3bHL}]+[\mathrm{fC3bCR1}]+[\mathrm{fiC3bCR1}]+[\mathrm{IgGC3bC3bH}]+[\mathrm{IgGC3biC3bH}]+[\mathrm{IgGC3bC3bHL}]}\cdots\right.$$

$$\frac{1}{+[\mathrm{IgGC3biC3bHL}]+[\mathrm{IgGC3bC3bCR1}]+[\mathrm{IgGC3biC3bCR1}]+[\mathrm{IgGiC3biC3bCR1}]+[\mathrm{IgGiC3bC3dgCR1}]+[\mathrm{fC3bC4bH}]+[\mathrm{fC3bC4bHL}]}\cdots$$

$$\frac{1}{+[\mathrm{fC3bC4bCR1}]+[\mathrm{fC3bC4bC4BP}]+[\mathrm{fC3bC4dH}]+[\mathrm{fC3bC4dHL}]+[\mathrm{fC3bC4dCR1}]+[\mathrm{fiC3bC4dCR1}]+[\mathrm{fiC3bC4bCR1}]+[\mathrm{fiC3bC4bC4BP}]}\cdots$$

$$\frac{1}{+[\mathrm{fC3dgC4bCR1}]+[\mathrm{fC3dgC4bC4BP}]+[\mathrm{fC3bC3bH}]+[\mathrm{fC3bC3bHL}]+[\mathrm{fC3bC3bCR1}]+[\mathrm{fC3biC3bH}]+[\mathrm{fC3biC3bHL}]+[\mathrm{fC3biC3bCR1}]}\cdots$$

$$\frac{1}{+[\mathrm{fiC3biC3bCR1}]+[\mathrm{fiC3bC3dgCR1}]+[\mathrm{hC3bH}]+[\mathrm{hC3bHL}]+[\mathrm{hC3bCR1}]+[\mathrm{hiC3bCR1}]+[\mathrm{hC3bC4bH}]+[\mathrm{hC3bC4bHL}]+[\mathrm{hC3bC4bCR1}]}\cdots$$

$$\frac{1}{+[\mathrm{hC3bC4bC4BP}]+[\mathrm{hC3bC4dH}]+[\mathrm{hC3bC4dHL}]+[\mathrm{hC3bC4dCR1}]+[\mathrm{hiC3bC4dCR1}]+[\mathrm{hiC3bC4bCR1}]+[\mathrm{hiC3bC4bC4BP}]+[\mathrm{hC3dgC4bCR1}]}\cdots$$

$$\frac{1}{+[\mathrm{hC3dgC4bC4BP}]+[\mathrm{hC3bC3bH}]+[\mathrm{hC3bC3bHL}]+[\mathrm{hC3bC3bCR1}]+[\mathrm{hC3biC3bH}]+[\mathrm{hC3biC3bHL}]+[\mathrm{hC3biC3bCR1}]+[\mathrm{hiC3biC3bCR1}]}\cdots$$

$$\frac{1}{+[\mathrm{hiC3bC3dgCR1}]+[\mathrm{fC4bCR1}]+[\mathrm{nfC4bC4BP}]+[\mathrm{fC4bC4BP}]+[\mathrm{fC4bC4bCR1}]+[\mathrm{fC4bC4bC4BP}]+[\mathrm{fC4bC4dCR1}]+[\mathrm{fC4bC4dC4BP}]+[\mathrm{hC4bCR1}]}\cdots$$

$$\left.\frac{1}{+[\mathrm{hC4bC4BP}]+[\mathrm{hC4bC4bCR1}]+[\mathrm{hC4bC4bC4BP}]+[\mathrm{hC4bC4dCR1}]+[\mathrm{hC4bC4dC4BP}]}\cdots\right)$$

$$-k^{+}_{\mathrm{C3bH}}[\mathrm{fC3biC3b}][\mathrm{H}]+k^{-}_{\mathrm{C3bH}}[\mathrm{fC3biC3bH}]-k^{+}_{\mathrm{C3bHL}}[\mathrm{fC3biC3b}][\mathrm{HL}]+k^{-}_{\mathrm{C3bHL}}[\mathrm{fC3biC3bHL}]-k^{+}_{\mathrm{C3biC3bCR1}}[\mathrm{fC3biC3b}][\mathrm{CR1}]$$

$$+k^{-}_{\mathrm{C3biC3bCR1}}[\mathrm{fC3biC3bCR1}]$$

S161

$$\frac{d[\text{fC3biC3bH}]}{dt} = k^{+}_{\text{C3bH}}[\text{fC3biC3b}][\text{H}] - k^{-}_{\text{C3bH}}[\text{fC3biC3bH}]$$

$$-\left(\begin{array}{l}\dfrac{k^{\text{FI}}_{\text{catC3bH}}[\text{I}][\text{fC3biC3bH}]}{K^{\text{FI}}_{\text{mC3bH}} + [\text{C3(H}_2\text{O)H}] + [\text{C3(H}_2\text{O)HL}] + [\text{fC3bH}] + [\text{fC3bHL}] + [\text{fC3bCR1}] + [\text{fiC3bCR1}] + [\text{IgGC3bC3bH}] + [\text{IgGC3biC3bH}] + [\text{IgGC3bC3bHL}]}\cdots \\ \dfrac{1}{+[\text{IgGC3biC3bHL}] + [\text{IgGC3bC3bCR1}] + [\text{IgGC3biC3bCR1}] + [\text{IgGiC3biC3bCR1}] + [\text{IgGiC3bC3dgCR1}] + [\text{fC3bC4bH}] + [\text{fC3bC4bHL}]}\cdots \\ \dfrac{1}{+[\text{fC3bC4bCR1}] + [\text{fC3bC4bC4BP}] + [\text{fC3bC4dH}] + [\text{fC3bC4dHL}] + [\text{fC3bC4dCR1}] + [\text{fiC3bC4dCR1}] + [\text{fiC3bC4bCR1}] + [\text{fiC3bC4bC4BP}]}\cdots \\ \dfrac{1}{+[\text{fC3dgC4bCR1}] + [\text{fC3dgC4bC4BP}] + [\text{fC3bC3bH}] + [\text{fC3bC3bHL}] + [\text{fC3bC3bCR1}] + [\text{fC3biC3bH}] + [\text{fC3biC3bHL}] + [\text{fC3biC3bCR1}]}\cdots \\ \dfrac{1}{+[\text{fiC3biC3bCR1}] + [\text{fiC3bC3dgCR1}] + [\text{hC3bH}] + [\text{hC3bHL}] + [\text{hC3bCR1}] + [\text{hiC3bCR1}] + [\text{hC3bC4bH}] + [\text{hC3bC4bHL}] + [\text{hC3bC4bCR1}]}\cdots \\ \dfrac{1}{+[\text{hC3bC4bC4BP}] + [\text{hC3bC4dH}] + [\text{hC3bC4dHL}] + [\text{hC3bC4dCR1}] + [\text{hiC3bC4dCR1}] + [\text{hiC3bC4bCR1}] + [\text{hiC3bC4bC4BP}] + [\text{hC3dgC4bCR1}]}\cdots \\ \dfrac{1}{+[\text{hC3dgC4bC4BP}] + [\text{hC3bC3bH}] + [\text{hC3bC3bHL}] + [\text{hC3bC3bCR1}] + [\text{hC3biC3bH}] + [\text{hC3biC3bHL}] + [\text{hC3biC3bCR1}] + [\text{hiC3biC3bCR1}]}\cdots \\ \dfrac{1}{+[\text{hiC3bC3dgCR1}] + [\text{fC4bCR1}] + [\text{nfC4bC4BP}] + [\text{fC4bC4BP}] + [\text{fC4bC4bCR1}] + [\text{fC4bC4bC4BP}] + [\text{fC4bC4dCR1}] + [\text{fC4bC4dC4BP}] + [\text{hC4bCR1}]}\cdots \\ \dfrac{1}{+[\text{hC4bC4BP}] + [\text{hC4bC4bCR1}] + [\text{hC4bC4bC4BP}] + [\text{hC4bC4dCR1}] + [\text{hC4bC4dC4BP}]}\cdots\end{array}\right)$$

S162

$$\frac{d[\text{fC3biC3bHL}]}{dt} = k^{+}_{\text{C3bHL}}[\text{fC3biC3b}][\text{HL}] - k^{-}_{\text{C3bHL}}[\text{fC3biC3bHL}]$$

$$-\left(\begin{array}{l}\dfrac{k^{\text{FI}}_{\text{catC3bH}}[\text{I}][\text{fC3biC3bHL}]}{K^{\text{FI}}_{\text{mC3bH}} + [\text{C3(H}_2\text{O)H}] + [\text{C3(H}_2\text{O)HL}] + [\text{fC3bH}] + [\text{fC3bHL}] + [\text{fC3bCR1}] + [\text{fiC3bCR1}] + [\text{IgGC3bC3bH}] + [\text{IgGC3biC3bH}] + [\text{IgGC3bC3bHL}]}\cdots \\ \dfrac{1}{+[\text{IgGC3biC3bHL}] + [\text{IgGC3bC3bCR1}] + [\text{IgGC3biC3bCR1}] + [\text{IgGiC3biC3bCR1}] + [\text{IgGiC3bC3dgCR1}] + [\text{fC3bC4bH}] + [\text{fC3bC4bHL}]}\cdots \\ \dfrac{1}{+[\text{fC3bC4bCR1}] + [\text{fC3bC4bC4BP}] + [\text{fC3bC4dH}] + [\text{fC3bC4dHL}] + [\text{fC3bC4dCR1}] + [\text{fiC3bC4dCR1}] + [\text{fiC3bC4bCR1}] + [\text{fiC3bC4bC4BP}]}\cdots \\ \dfrac{1}{+[\text{fC3dgC4bCR1}] + [\text{fC3dgC4bC4BP}] + [\text{fC3bC3bH}] + [\text{fC3bC3bHL}] + [\text{fC3bC3bCR1}] + [\text{fC3biC3bH}] + [\text{fC3biC3bHL}] + [\text{fC3biC3bCR1}]}\cdots \\ \dfrac{1}{+[\text{fiC3biC3bCR1}] + [\text{fiC3bC3dgCR1}] + [\text{hC3bH}] + [\text{hC3bHL}] + [\text{hC3bCR1}] + [\text{hiC3bCR1}] + [\text{hC3bC4bH}] + [\text{hC3bC4bHL}] + [\text{hC3bC4bCR1}]}\cdots \\ \dfrac{1}{+[\text{hC3bC4bC4BP}] + [\text{hC3bC4dH}] + [\text{hC3bC4dHL}] + [\text{hC3bC4dCR1}] + [\text{hiC3bC4dCR1}] + [\text{hiC3bC4bCR1}] + [\text{hiC3bC4bC4BP}] + [\text{hC3dgC4bCR1}]}\cdots \\ \dfrac{1}{+[\text{hC3dgC4bC4BP}] + [\text{hC3bC3bH}] + [\text{hC3bC3bHL}] + [\text{hC3bC3bCR1}] + [\text{hC3biC3bH}] + [\text{hC3biC3bHL}] + [\text{hC3biC3bCR1}] + [\text{hiC3biC3bCR1}]}\cdots \\ \dfrac{1}{+[\text{hiC3bC3dgCR1}] + [\text{fC4bCR1}] + [\text{nfC4bC4BP}] + [\text{fC4bC4BP}] + [\text{fC4bC4bCR1}] + [\text{fC4bC4bC4BP}] + [\text{fC4bC4dCR1}] + [\text{fC4bC4dC4BP}] + [\text{hC4bCR1}]}\cdots \\ \dfrac{1}{+[\text{hC4bC4BP}] + [\text{hC4bC4bCR1}] + [\text{hC4bC4bC4BP}] + [\text{hC4bC4dCR1}] + [\text{hC4bC4dC4BP}]}\cdots\end{array}\right)$$

S163

$$\frac{d[\mathrm{fC3biC3bCR1}]}{dt} = k^{+}_{\mathrm{C3biC3bCR1}}[\mathrm{fC3biC3b}][\mathrm{CR1}] - k^{-}_{\mathrm{C3biC3bCR1}}[\mathrm{fC3biC3bCR1}]$$

$$-\left(\frac{k^{\mathrm{FI}}_{\mathrm{catC3bH}}[\mathrm{I}][\mathrm{fC3biC3bCR1}]}{K^{\mathrm{FI}}_{\mathrm{mC3bH}} + [\mathrm{C3(H_2O)H}] + [\mathrm{C3(H_2O)HL}] + [\mathrm{fC3bH}] + [\mathrm{fC3bHL}] + [\mathrm{fC3bCR1}] + [\mathrm{fiC3bCR1}] + [\mathrm{IgGC3bC3bH}] + [\mathrm{IgGC3biC3bH}] + [\mathrm{IgGC3bC3bHL}]} \cdots \right.$$

$$\frac{1}{+ [\mathrm{IgGC3biC3bHL}] + [\mathrm{IgGC3bC3bCR1}] + [\mathrm{IgGC3biC3bCR1}] + [\mathrm{IgGiC3biC3bCR1}] + [\mathrm{IgGiC3bC3dgCR1}] + [\mathrm{fC3bC4bH}] + [\mathrm{fC3bC4bHL}]} \cdots$$

$$\frac{1}{+ [\mathrm{fC3bC4bCR1}] + [\mathrm{fC3bC4bC4BP}] + [\mathrm{fC3bC4dH}] + [\mathrm{fC3bC4dHL}] + [\mathrm{fC3bC4dCR1}] + [\mathrm{fiC3bC4dCR1}] + [\mathrm{fiC3bC4bCR1}] + [\mathrm{fiC3bC4bC4BP}]} \cdots$$

$$\frac{1}{+ [\mathrm{fC3dgC4bCR1}] + [\mathrm{fC3dgC4bC4BP}] + [\mathrm{fC3bC3bH}] + [\mathrm{fC3bC3bHL}] + [\mathrm{fC3bC3bCR1}] + [\mathrm{fC3biC3bH}] + [\mathrm{fC3biC3bHL}] + [\mathrm{fC3biC3bCR1}]} \cdots$$

$$\frac{1}{+ [\mathrm{fiC3biC3bCR1}] + [\mathrm{fiC3bC3dgCR1}] + [\mathrm{hC3bH}] + [\mathrm{hC3bHL}] + [\mathrm{hC3bCR1}] + [\mathrm{hiC3bCR1}] + [\mathrm{hC3bC4bH}] + [\mathrm{hC3bC4bHL}] + [\mathrm{hC3bC4bCR1}]} \cdots$$

$$\frac{1}{+ [\mathrm{hC3bC4bC4BP}] + [\mathrm{hC3bC4dH}] + [\mathrm{hC3bC4dHL}] + [\mathrm{hC3bC4dCR1}] + [\mathrm{hiC3bC4dCR1}] + [\mathrm{hiC3bC4bCR1}] + [\mathrm{hiC3bC4bC4BP}] + [\mathrm{hC3dgC4bCR1}]} \cdots$$

$$\frac{1}{+ [\mathrm{hC3dgC4bC4BP}] + [\mathrm{hC3bC3bH}] + [\mathrm{hC3bC3bHL}] + [\mathrm{hC3bC3bCR1}] + [\mathrm{hC3biC3bH}] + [\mathrm{hC3biC3bHL}] + [\mathrm{hC3biC3bCR1}] + [\mathrm{hiC3biC3bCR1}]} \cdots$$

$$\frac{1}{+ [\mathrm{hiC3bC3dgCR1}] + [\mathrm{fC4bCR1}] + [\mathrm{nfC4bC4BP}] + [\mathrm{fC4bC4BP}] + [\mathrm{fC4bC4bCR1}] + [\mathrm{fC4bC4bC4BP}] + [\mathrm{fC4bC4dCR1}] + [\mathrm{fC4bC4dC4BP}] + [\mathrm{hC4bCR1}]} \cdots$$

$$\left. \frac{1}{+ [\mathrm{hC4bC4BP}] + [\mathrm{hC4bC4bCR1}] + [\mathrm{hC4bC4bC4BP}] + [\mathrm{hC4bC4dCR1}] + [\mathrm{hC4bC4dC4BP}]} \cdots \right)$$

S164

$$\frac{d[\mathrm{fiC3biC3b}]}{dt} =$$

$$\left(\frac{k^{\mathrm{FI}}_{\mathrm{catC3bH}}[\mathrm{I}][\mathrm{fC3biC3bH}]}{K^{\mathrm{FI}}_{\mathrm{mC3bH}} + [\mathrm{C3(H_2O)H}] + [\mathrm{C3(H_2O)HL}] + [\mathrm{fC3bH}] + [\mathrm{fC3bHL}] + [\mathrm{fC3bCR1}] + [\mathrm{fiC3bCR1}] + [\mathrm{IgGC3bC3bH}] + [\mathrm{IgGC3biC3bH}] + [\mathrm{IgGC3bC3bHL}]} \cdots \right.$$

$$\frac{1}{+ [\mathrm{IgGC3biC3bHL}] + [\mathrm{IgGC3bC3bCR1}] + [\mathrm{IgGC3biC3bCR1}] + [\mathrm{IgGiC3biC3bCR1}] + [\mathrm{IgGiC3bC3dgCR1}] + [\mathrm{fC3bC4bH}] + [\mathrm{fC3bC4bHL}]} \cdots$$

$$\frac{1}{+ [\mathrm{fC3bC4bCR1}] + [\mathrm{fC3bC4bC4BP}] + [\mathrm{fC3bC4dH}] + [\mathrm{fC3bC4dHL}] + [\mathrm{fC3bC4dCR1}] + [\mathrm{fiC3bC4dCR1}] + [\mathrm{fiC3bC4bCR1}] + [\mathrm{fiC3bC4bC4BP}]} \cdots$$

$$\frac{1}{+ [\mathrm{fC3dgC4bCR1}] + [\mathrm{fC3dgC4bC4BP}] + [\mathrm{fC3bC3bH}] + [\mathrm{fC3bC3bHL}] + [\mathrm{fC3bC3bCR1}] + [\mathrm{fC3biC3bH}] + [\mathrm{fC3biC3bHL}] + [\mathrm{fC3biC3bCR1}]} \cdots$$

$$\frac{1}{+ [\mathrm{fiC3biC3bCR1}] + [\mathrm{fiC3bC3dgCR1}] + [\mathrm{hC3bH}] + [\mathrm{hC3bHL}] + [\mathrm{hC3bCR1}] + [\mathrm{hiC3bCR1}] + [\mathrm{hC3bC4bH}] + [\mathrm{hC3bC4bHL}] + [\mathrm{hC3bC4bCR1}]} \cdots$$

$$\frac{1}{+ [\mathrm{hC3bC4bC4BP}] + [\mathrm{hC3bC4dH}] + [\mathrm{hC3bC4dHL}] + [\mathrm{hC3bC4dCR1}] + [\mathrm{hiC3bC4dCR1}] + [\mathrm{hiC3bC4bCR1}] + [\mathrm{hiC3bC4bC4BP}] + [\mathrm{hC3dgC4bCR1}]} \cdots$$

$$\frac{1}{+ [\mathrm{hC3dgC4bC4BP}] + [\mathrm{hC3bC3bH}] + [\mathrm{hC3bC3bHL}] + [\mathrm{hC3bC3bCR1}] + [\mathrm{hC3biC3bH}] + [\mathrm{hC3biC3bHL}] + [\mathrm{hC3biC3bCR1}] + [\mathrm{hiC3biC3bCR1}]} \cdots$$

$$\frac{1}{+ [\mathrm{hiC3bC3dgCR1}] + [\mathrm{fC4bCR1}] + [\mathrm{nfC4bC4BP}] + [\mathrm{fC4bC4BP}] + [\mathrm{fC4bC4bCR1}] + [\mathrm{fC4bC4bC4BP}] + [\mathrm{fC4bC4dCR1}] + [\mathrm{fC4bC4dC4BP}] + [\mathrm{hC4bCR1}]} \cdots$$

$$\left. \frac{1}{+ [\mathrm{hC4bC4BP}] + [\mathrm{hC4bC4bCR1}] + [\mathrm{hC4bC4bC4BP}] + [\mathrm{hC4bC4dCR1}] + [\mathrm{hC4bC4dC4BP}]} \cdots \right)$$

$$
+\left(\begin{array}{l}
\dfrac{k_{\mathrm{catC3bH}}^{\mathrm{FI}}[\mathrm{I}][\mathrm{fC3biC3bHL}]}{K_{\mathrm{mC3bH}}^{\mathrm{FI}}+[\mathrm{C3(H_2O)H}]+[\mathrm{C3(H_2O)HL}]+[\mathrm{fC3bH}]+[\mathrm{fC3bHL}]+[\mathrm{fC3bCR1}]+[\mathrm{fiC3bCR1}]+[\mathrm{IgGC3bC3bH}]+[\mathrm{IgGC3biC3bH}]+[\mathrm{IgGC3bC3bHL}]}\cdots \\
\dfrac{1}{+[\mathrm{IgGC3biC3bHL}]+[\mathrm{IgGC3bC3bCR1}]+[\mathrm{IgGC3biC3bCR1}]+[\mathrm{IgGiC3biC3bCR1}]+[\mathrm{IgGiC3bC3dgCR1}]+[\mathrm{fC3bC4bH}]+[\mathrm{fC3bC4bHL}]}\cdots \\
\dfrac{1}{+[\mathrm{fC3bC4bCR1}]+[\mathrm{fC3bC4bC4BP}]+[\mathrm{fC3bC4dH}]+[\mathrm{fC3bC4dHL}]+[\mathrm{fC3bC4dCR1}]+[\mathrm{fiC3bC4dCR1}]+[\mathrm{fiC3bC4bCR1}]+[\mathrm{fiC3bC4bC4BP}]}\cdots \\
\dfrac{1}{+[\mathrm{fC3dgC4bCR1}]+[\mathrm{fC3dgC4bC4BP}]+[\mathrm{fC3bC3bH}]+[\mathrm{fC3bC3bHL}]+[\mathrm{fC3bC3bCR1}]+[\mathrm{fC3biC3bH}]+[\mathrm{fC3biC3bHL}]+[\mathrm{fC3biC3bCR1}]}\cdots \\
\dfrac{1}{+[\mathrm{fiC3biC3bCR1}]+[\mathrm{fiC3bC3dgCR1}]+[\mathrm{hC3bH}]+[\mathrm{hC3bHL}]+[\mathrm{hC3bCR1}]+[\mathrm{hiC3bCR1}]+[\mathrm{hC3bC4bH}]+[\mathrm{hC3bC4bHL}]+[\mathrm{hC3bC4bCR1}]}\cdots \\
\dfrac{1}{+[\mathrm{hC3bC4bC4BP}]+[\mathrm{hC3bC4dH}]+[\mathrm{hC3bC4dHL}]+[\mathrm{hC3bC4dCR1}]+[\mathrm{hiC3bC4dCR1}]+[\mathrm{hiC3bC4bCR1}]+[\mathrm{hiC3bC4bC4BP}]+[\mathrm{hC3dgC4bCR1}]}\cdots \\
\dfrac{1}{+[\mathrm{hC3dgC4bC4BP}]+[\mathrm{hC3bC3bH}]+[\mathrm{hC3bC3bHL}]+[\mathrm{hC3bC3bCR1}]+[\mathrm{hC3biC3bH}]+[\mathrm{hC3biC3bHL}]+[\mathrm{hC3biC3bCR1}]+[\mathrm{hiC3biC3bCR1}]}\cdots \\
\dfrac{1}{+[\mathrm{hiC3bC3dgCR1}]+[\mathrm{fC4bCR1}]+[\mathrm{nfC4bC4BP}]+[\mathrm{fC4bC4BP}]+[\mathrm{fC4bC4bCR1}]+[\mathrm{fC4bC4bC4BP}]+[\mathrm{fC4bC4dCR1}]+[\mathrm{fC4bC4dC4BP}]+[\mathrm{hC4bCR1}]}\cdots \\
\dfrac{1}{+[\mathrm{hC4bC4BP}]+[\mathrm{hC4bC4bCR1}]+[\mathrm{hC4bC4bC4BP}]+[\mathrm{hC4bC4dCR1}]+[\mathrm{hC4bC4dC4BP}]}\cdots
\end{array}\right)
$$

$$
+\left(\begin{array}{l}
\dfrac{k_{\mathrm{catC3bH}}^{\mathrm{FI}}[\mathrm{I}][\mathrm{fC3biC3bCR1}]}{K_{\mathrm{mC3bH}}^{\mathrm{FI}}+[\mathrm{C3(H_2O)H}]+[\mathrm{C3(H_2O)HL}]+[\mathrm{fC3bH}]+[\mathrm{fC3bHL}]+[\mathrm{fC3bCR1}]+[\mathrm{fiC3bCR1}]+[\mathrm{IgGC3bC3bH}]+[\mathrm{IgGC3biC3bH}]+[\mathrm{IgGC3bC3bHL}]}\cdots \\
\dfrac{1}{+[\mathrm{IgGC3biC3bHL}]+[\mathrm{IgGC3bC3bCR1}]+[\mathrm{IgGC3biC3bCR1}]+[\mathrm{IgGiC3biC3bCR1}]+[\mathrm{IgGiC3bC3dgCR1}]+[\mathrm{fC3bC4bH}]+[\mathrm{fC3bC4bHL}]}\cdots \\
\dfrac{1}{+[\mathrm{fC3bC4bCR1}]+[\mathrm{fC3bC4bC4BP}]+[\mathrm{fC3bC4dH}]+[\mathrm{fC3bC4dHL}]+[\mathrm{fC3bC4dCR1}]+[\mathrm{fiC3bC4dCR1}]+[\mathrm{fiC3bC4bCR1}]+[\mathrm{fiC3bC4bC4BP}]}\cdots \\
\dfrac{1}{+[\mathrm{fC3dgC4bCR1}]+[\mathrm{fC3dgC4bC4BP}]+[\mathrm{fC3bC3bH}]+[\mathrm{fC3bC3bHL}]+[\mathrm{fC3bC3bCR1}]+[\mathrm{fC3biC3bH}]+[\mathrm{fC3biC3bHL}]+[\mathrm{fC3biC3bCR1}]}\cdots \\
\dfrac{1}{+[\mathrm{fiC3biC3bCR1}]+[\mathrm{fiC3bC3dgCR1}]+[\mathrm{hC3bH}]+[\mathrm{hC3bHL}]+[\mathrm{hC3bCR1}]+[\mathrm{hiC3bCR1}]+[\mathrm{hC3bC4bH}]+[\mathrm{hC3bC4bHL}]+[\mathrm{hC3bC4bCR1}]}\cdots \\
\dfrac{1}{+[\mathrm{hC3bC4bC4BP}]+[\mathrm{hC3bC4dH}]+[\mathrm{hC3bC4dHL}]+[\mathrm{hC3bC4dCR1}]+[\mathrm{hiC3bC4dCR1}]+[\mathrm{hiC3bC4bCR1}]+[\mathrm{hiC3bC4bC4BP}]+[\mathrm{hC3dgC4bCR1}]}\cdots \\
\dfrac{1}{+[\mathrm{hC3dgC4bC4BP}]+[\mathrm{hC3bC3bH}]+[\mathrm{hC3bC3bHL}]+[\mathrm{hC3bC3bCR1}]+[\mathrm{hC3biC3bH}]+[\mathrm{hC3biC3bHL}]+[\mathrm{hC3biC3bCR1}]+[\mathrm{hiC3biC3bCR1}]}\cdots \\
\dfrac{1}{+[\mathrm{hiC3bC3dgCR1}]+[\mathrm{fC4bCR1}]+[\mathrm{nfC4bC4BP}]+[\mathrm{fC4bC4BP}]+[\mathrm{fC4bC4bCR1}]+[\mathrm{fC4bC4bC4BP}]+[\mathrm{fC4bC4dCR1}]+[\mathrm{fC4bC4dC4BP}]+[\mathrm{hC4bCR1}]}\cdots \\
\dfrac{1}{+[\mathrm{hC4bC4BP}]+[\mathrm{hC4bC4bCR1}]+[\mathrm{hC4bC4bC4BP}]+[\mathrm{hC4bC4dCR1}]+[\mathrm{hC4bC4dC4BP}]}\cdots
\end{array}\right)
$$

$$
-k_{\mathrm{iC3biC3bCR1}}^{+}[\mathrm{fiC3biC3b}][\mathrm{CR1}]+k_{\mathrm{iC3biC3bCR1}}^{-}[\mathrm{fiC3biC3bCR1}]
$$

S165

$$\frac{d[\mathrm{fiC3biC3bCR1}]}{dt} = k^{+}_{\mathrm{iC3biC3bCR1}}[\mathrm{fiC3biC3b}][\mathrm{CR1}] - k^{-}_{\mathrm{iC3biC3bCR1}}[\mathrm{fiC3biC3bCR1}]$$

$$- \left(\begin{array}{l}
\dfrac{k^{\mathrm{FI}}_{\mathrm{catC3bH}}[\mathrm{I}][\mathrm{fiC3biC3bCR1}]}{K^{\mathrm{FI}}_{\mathrm{mC3bH}} + [\mathrm{C3(H_2O)H}] + [\mathrm{C3(H_2O)HL}] + [\mathrm{fC3bH}] + [\mathrm{fC3bHL}] + [\mathrm{fC3bCR1}] + [\mathrm{fiC3bCR1}] + [\mathrm{IgGC3bC3bH}] + [\mathrm{IgGC3biC3bH}] + [\mathrm{IgGC3bC3bHL}]} \cdots \\
\dfrac{1}{+ [\mathrm{IgGC3biC3bHL}] + [\mathrm{IgGC3bC3bCR1}] + [\mathrm{IgGC3biC3bCR1}] + [\mathrm{IgGiC3biC3bCR1}] + [\mathrm{IgGiC3bC3dgCR1}] + [\mathrm{fC3bC4bH}] + [\mathrm{fC3bC4bHL}]} \cdots \\
\dfrac{1}{+ [\mathrm{fC3bC4bCR1}] + [\mathrm{fC3bC4bC4BP}] + [\mathrm{fC3bC4dH}] + [\mathrm{fC3bC4dHL}] + [\mathrm{fC3bC4dCR1}] + [\mathrm{fiC3bC4dCR1}] + [\mathrm{fiC3bC4bCR1}] + [\mathrm{fiC3bC4bC4BP}]} \cdots \\
\dfrac{1}{+ [\mathrm{fC3dgC4bCR1}] + [\mathrm{fC3dgC4bC4BP}] + [\mathrm{fC3bC3bH}] + [\mathrm{fC3bC3bHL}] + [\mathrm{fC3bC3bCR1}] + [\mathrm{fC3biC3bH}] + [\mathrm{fC3biC3bHL}] + [\mathrm{fC3biC3bCR1}]} \cdots \\
\dfrac{1}{+ [\mathrm{fiC3biC3bCR1}] + [\mathrm{fiC3bC3dgCR1}] + [\mathrm{hC3bH}] + [\mathrm{hC3bHL}] + [\mathrm{hC3bCR1}] + [\mathrm{hiC3bCR1}] + [\mathrm{hC3bC4bH}] + [\mathrm{hC3bC4bHL}] + [\mathrm{hC3bC4bCR1}]} \cdots \\
\dfrac{1}{+ [\mathrm{hC3bC4bC4BP}] + [\mathrm{hC3bC4dH}] + [\mathrm{hC3bC4dHL}] + [\mathrm{hC3bC4dCR1}] + [\mathrm{hiC3bC4dCR1}] + [\mathrm{hiC3bC4bCR1}] + [\mathrm{hiC3bC4bC4BP}] + [\mathrm{hC3dgC4bCR1}]} \cdots \\
\dfrac{1}{+ [\mathrm{hC3dgC4bC4BP}] + [\mathrm{hC3bC3bH}] + [\mathrm{hC3bC3bHL}] + [\mathrm{hC3bC3bCR1}] + [\mathrm{hC3biC3bH}] + [\mathrm{hC3biC3bHL}] + [\mathrm{hC3biC3bCR1}] + [\mathrm{hiC3biC3bCR1}]} \cdots \\
\dfrac{1}{+ [\mathrm{hiC3bC3dgCR1}] + [\mathrm{fC4bCR1}] + [\mathrm{nfC4bC4BP}] + [\mathrm{fC4bC4BP}] + [\mathrm{fC4bC4bCR1}] + [\mathrm{fC4bC4bC4BP}] + [\mathrm{fC4bC4dCR1}] + [\mathrm{fC4bC4dC4BP}] + [\mathrm{hC4bCR1}]} \cdots \\
\dfrac{1}{+ [\mathrm{hC4bC4BP}] + [\mathrm{hC4bC4bCR1}] + [\mathrm{hC4bC4bC4BP}] + [\mathrm{hC4bC4dCR1}] + [\mathrm{hC4bC4dC4BP}]} \cdots
\end{array} \right)$$

S166

$$\frac{d[\mathrm{fiC3bC3dg}]}{dt} =$$

$$\left(\begin{array}{l}
\dfrac{k^{\mathrm{FI}}_{\mathrm{catC3bH}}[\mathrm{I}][\mathrm{fiC3biC3bCR1}]}{K^{\mathrm{FI}}_{\mathrm{mC3bH}} + [\mathrm{C3(H_2O)H}] + [\mathrm{C3(H_2O)HL}] + [\mathrm{fC3bH}] + [\mathrm{fC3bHL}] + [\mathrm{fC3bCR1}] + [\mathrm{fiC3bCR1}] + [\mathrm{IgGC3bC3bH}] + [\mathrm{IgGC3biC3bH}] + [\mathrm{IgGC3bC3bHL}]} \cdots \\
\dfrac{1}{+ [\mathrm{IgGC3biC3bHL}] + [\mathrm{IgGC3bC3bCR1}] + [\mathrm{IgGC3biC3bCR1}] + [\mathrm{IgGiC3biC3bCR1}] + [\mathrm{IgGiC3bC3dgCR1}] + [\mathrm{fC3bC4bH}] + [\mathrm{fC3bC4bHL}]} \cdots \\
\dfrac{1}{+ [\mathrm{fC3bC4bCR1}] + [\mathrm{fC3bC4bC4BP}] + [\mathrm{fC3bC4dH}] + [\mathrm{fC3bC4dHL}] + [\mathrm{fC3bC4dCR1}] + [\mathrm{fiC3bC4dCR1}] + [\mathrm{fiC3bC4bCR1}] + [\mathrm{fiC3bC4bC4BP}]} \cdots \\
\dfrac{1}{+ [\mathrm{fC3dgC4bCR1}] + [\mathrm{fC3dgC4bC4BP}] + [\mathrm{fC3bC3bH}] + [\mathrm{fC3bC3bHL}] + [\mathrm{fC3bC3bCR1}] + [\mathrm{fC3biC3bH}] + [\mathrm{fC3biC3bHL}] + [\mathrm{fC3biC3bCR1}]} \cdots \\
\dfrac{1}{+ [\mathrm{fiC3biC3bCR1}] + [\mathrm{fiC3bC3dgCR1}] + [\mathrm{hC3bH}] + [\mathrm{hC3bHL}] + [\mathrm{hC3bCR1}] + [\mathrm{hiC3bCR1}] + [\mathrm{hC3bC4bH}] + [\mathrm{hC3bC4bHL}] + [\mathrm{hC3bC4bCR1}]} \cdots \\
\dfrac{1}{+ [\mathrm{hC3bC4bC4BP}] + [\mathrm{hC3bC4dH}] + [\mathrm{hC3bC4dHL}] + [\mathrm{hC3bC4dCR1}] + [\mathrm{hiC3bC4dCR1}] + [\mathrm{hiC3bC4bCR1}] + [\mathrm{hiC3bC4bC4BP}] + [\mathrm{hC3dgC4bCR1}]} \cdots \\
\dfrac{1}{+ [\mathrm{hC3dgC4bC4BP}] + [\mathrm{hC3bC3bH}] + [\mathrm{hC3bC3bHL}] + [\mathrm{hC3bC3bCR1}] + [\mathrm{hC3biC3bH}] + [\mathrm{hC3biC3bHL}] + [\mathrm{hC3biC3bCR1}] + [\mathrm{hiC3biC3bCR1}]} \cdots \\
\dfrac{1}{+ [\mathrm{hiC3bC3dgCR1}] + [\mathrm{fC4bCR1}] + [\mathrm{nfC4bC4BP}] + [\mathrm{fC4bC4BP}] + [\mathrm{fC4bC4bCR1}] + [\mathrm{fC4bC4bC4BP}] + [\mathrm{fC4bC4dCR1}] + [\mathrm{fC4bC4dC4BP}] + [\mathrm{hC4bCR1}]} \cdots \\
\dfrac{1}{+ [\mathrm{hC4bC4BP}] + [\mathrm{hC4bC4bCR1}] + [\mathrm{hC4bC4bC4BP}] + [\mathrm{hC4bC4dCR1}] + [\mathrm{hC4bC4dC4BP}]} \cdots
\end{array} \right)$$

$$- k^{+}_{\mathrm{iC3bC3dgCR1}}[\mathrm{fiC3bC3dg}][\mathrm{CR1}] + k^{-}_{\mathrm{iC3bC3dgCR1}}[\mathrm{fiC3bC3dgCR1}]$$

S167

$$\frac{d[\mathrm{fiC3bC3dgCR1}]}{dt} = k^{+}_{\mathrm{iC3bC3dgCR1}}[\mathrm{fiC3bC3dg}][\mathrm{CR1}] - k^{-}_{\mathrm{iC3bC3dgCR1}}[\mathrm{fiC3bC3dgCR1}]$$

$$-\left(\begin{array}{l}
\dfrac{k^{\mathrm{FI}}_{\mathrm{catC3bH}}[\mathrm{I}][\mathrm{fiC3bC3dgCR1}]}{K^{\mathrm{FI}}_{\mathrm{mC3bH}} + [\mathrm{C3(H_2O)H}] + [\mathrm{C3(H_2O)HL}] + [\mathrm{fC3bH}] + [\mathrm{fC3bHL}] + [\mathrm{fC3bCR1}] + [\mathrm{fiC3bCR1}] + [\mathrm{IgGC3bC3bH}] + [\mathrm{IgGC3biC3bH}] + [\mathrm{IgGC3bC3bHL}]}\cdots \\
\dfrac{1}{+[\mathrm{IgGC3biC3bHL}] + [\mathrm{IgGC3bC3bCR1}] + [\mathrm{IgGC3biC3bCR1}] + [\mathrm{IgGiC3biC3bCR1}] + [\mathrm{IgGiC3bC3dgCR1}] + [\mathrm{fC3bC4bH}] + [\mathrm{fC3bC4bHL}]}\cdots \\
\dfrac{1}{+[\mathrm{fC3bC4bCR1}] + [\mathrm{fC3bC4bC4BP}] + [\mathrm{fC3bC4dH}] + [\mathrm{fC3bC4dHL}] + [\mathrm{fC3bC4dCR1}] + [\mathrm{fiC3bC4dCR1}] + [\mathrm{fiC3bC4bCR1}] + [\mathrm{fiC3bC4bC4BP}]}\cdots \\
\dfrac{1}{+[\mathrm{fC3dgC4bCR1}] + [\mathrm{fC3dgC4bC4BP}] + [\mathrm{fC3bC3bH}] + [\mathrm{fC3bC3bHL}] + [\mathrm{fC3bC3bCR1}] + [\mathrm{fC3biC3bH}] + [\mathrm{fC3biC3bHL}] + [\mathrm{fC3biC3bCR1}]}\cdots \\
\dfrac{1}{+[\mathrm{fiC3biC3bCR1}] + [\mathrm{fiC3bC3dgCR1}] + [\mathrm{hC3bH}] + [\mathrm{hC3bHL}] + [\mathrm{hC3bCR1}] + [\mathrm{hiC3bCR1}] + [\mathrm{hC3bC4bH}] + [\mathrm{hC3bC4bHL}] + [\mathrm{hC3bC4bCR1}]}\cdots \\
\dfrac{1}{+[\mathrm{hC3bC4bC4BP}] + [\mathrm{hC3bC4dH}] + [\mathrm{hC3bC4dHL}] + [\mathrm{hC3bC4dCR1}] + [\mathrm{hiC3bC4dCR1}] + [\mathrm{hiC3bC4bCR1}] + [\mathrm{hiC3bC4bC4BP}] + [\mathrm{hC3dgC4bCR1}]}\cdots \\
\dfrac{1}{+[\mathrm{hC3dgC4bC4BP}] + [\mathrm{hC3bC3bH}] + [\mathrm{hC3bC3bHL}] + [\mathrm{hC3bC3bCR1}] + [\mathrm{hC3biC3bH}] + [\mathrm{hC3biC3bHL}] + [\mathrm{hC3biC3bCR1}] + [\mathrm{hiC3biC3bCR1}]}\cdots \\
\dfrac{1}{+[\mathrm{hiC3bC3dgCR1}] + [\mathrm{fC4bCR1}] + [\mathrm{nfC4bC4BP}] + [\mathrm{fC4bC4BP}] + [\mathrm{fC4bC4bCR1}] + [\mathrm{fC4bC4bC4BP}] + [\mathrm{fC4bC4dCR1}] + [\mathrm{fC4bC4dC4BP}] + [\mathrm{hC4bCR1}]}\cdots \\
\dfrac{1}{+[\mathrm{hC4bC4BP}] + [\mathrm{hC4bC4bCR1}] + [\mathrm{hC4bC4bC4BP}] + [\mathrm{hC4bC4dCR1}] + [\mathrm{hC4bC4dC4BP}]}\cdots
\end{array}\right)$$

S168

$$\frac{d[\mathrm{fC3dgC3dg}]}{dt} =$$

$$\left(\begin{array}{l}
\dfrac{k^{\mathrm{FI}}_{\mathrm{catC3bH}}[\mathrm{I}][\mathrm{fiC3bC3dgCR1}]}{K^{\mathrm{FI}}_{\mathrm{mC3bH}} + [\mathrm{C3(H_2O)H}] + [\mathrm{C3(H_2O)HL}] + [\mathrm{fC3bH}] + [\mathrm{fC3bHL}] + [\mathrm{fC3bCR1}] + [\mathrm{fiC3bCR1}] + [\mathrm{IgGC3bC3bH}] + [\mathrm{IgGC3biC3bH}] + [\mathrm{IgGC3bC3bHL}]}\cdots \\
\dfrac{1}{+[\mathrm{IgGC3biC3bHL}] + [\mathrm{IgGC3bC3bCR1}] + [\mathrm{IgGC3biC3bCR1}] + [\mathrm{IgGiC3biC3bCR1}] + [\mathrm{IgGiC3bC3dgCR1}] + [\mathrm{fC3bC4bH}] + [\mathrm{fC3bC4bHL}]}\cdots \\
\dfrac{1}{+[\mathrm{fC3bC4bCR1}] + [\mathrm{fC3bC4bC4BP}] + [\mathrm{fC3bC4dH}] + [\mathrm{fC3bC4dHL}] + [\mathrm{fC3bC4dCR1}] + [\mathrm{fiC3bC4dCR1}] + [\mathrm{fiC3bC4bCR1}] + [\mathrm{fiC3bC4bC4BP}]}\cdots \\
\dfrac{1}{+[\mathrm{fC3dgC4bCR1}] + [\mathrm{fC3dgC4bC4BP}] + [\mathrm{fC3bC3bH}] + [\mathrm{fC3bC3bHL}] + [\mathrm{fC3bC3bCR1}] + [\mathrm{fC3biC3bH}] + [\mathrm{fC3biC3bHL}] + [\mathrm{fC3biC3bCR1}]}\cdots \\
\dfrac{1}{+[\mathrm{fiC3biC3bCR1}] + [\mathrm{fiC3bC3dgCR1}] + [\mathrm{hC3bH}] + [\mathrm{hC3bHL}] + [\mathrm{hC3bCR1}] + [\mathrm{hiC3bCR1}] + [\mathrm{hC3bC4bH}] + [\mathrm{hC3bC4bHL}] + [\mathrm{hC3bC4bCR1}]}\cdots \\
\dfrac{1}{+[\mathrm{hC3bC4bC4BP}] + [\mathrm{hC3bC4dH}] + [\mathrm{hC3bC4dHL}] + [\mathrm{hC3bC4dCR1}] + [\mathrm{hiC3bC4dCR1}] + [\mathrm{hiC3bC4bCR1}] + [\mathrm{hiC3bC4bC4BP}] + [\mathrm{hC3dgC4bCR1}]}\cdots \\
\dfrac{1}{+[\mathrm{hC3dgC4bC4BP}] + [\mathrm{hC3bC3bH}] + [\mathrm{hC3bC3bHL}] + [\mathrm{hC3bC3bCR1}] + [\mathrm{hC3biC3bH}] + [\mathrm{hC3biC3bHL}] + [\mathrm{hC3biC3bCR1}] + [\mathrm{hiC3biC3bCR1}]}\cdots \\
\dfrac{1}{+[\mathrm{hiC3bC3dgCR1}] + [\mathrm{fC4bCR1}] + [\mathrm{nfC4bC4BP}] + [\mathrm{fC4bC4BP}] + [\mathrm{fC4bC4bCR1}] + [\mathrm{fC4bC4bC4BP}] + [\mathrm{fC4bC4dCR1}] + [\mathrm{fC4bC4dC4BP}] + [\mathrm{hC4bCR1}]}\cdots \\
\dfrac{1}{+[\mathrm{hC4bC4BP}] + [\mathrm{hC4bC4bCR1}] + [\mathrm{hC4bC4bC4BP}] + [\mathrm{hC4bC4dCR1}] + [\mathrm{hC4bC4dC4BP}]}\cdots
\end{array}\right)$$

S169

$$\frac{d[\text{fC3bC3bBbH}]}{dt} = k^{+}_{\text{C3bH}}[\text{fC3bC3bBb}][\text{H}] - k^{-}_{\text{C3bH}}[\text{fC3bC3bBbH}] - k^{-}_{\text{C3bBbH}_{\text{decay}}}[\text{fC3bC3bBbH}]$$

S170

$$\frac{d[\text{fC3bC3bBbHL}]}{dt} = k^{+}_{\text{C3bHL}}[\text{fC3bC3bBb}][\text{HL}] - k^{-}_{\text{C3bHL}}[\text{fC3bC3bBbHL}] - k^{-}_{\text{C3bBbHL}_{\text{decay}}}[\text{fC3bC3bBbHL}]$$

S171

$$\frac{d[\text{fC3bC3bBbCR1}]}{dt} = k^{+}_{\text{C3bC3bCR1}}[\text{fC3bC3bBb}][\text{CR1}] - k^{-}_{\text{C3bC3bCR1}}[\text{fC3bC3bBbCR1}] - k^{-}_{\text{C3bBbCR1}_{\text{decay}}}[\text{fC3bC3bBbCR1}]$$

S172

$$\frac{d[\text{IgGC3bC3bBbH}]}{dt} = k^{+}_{\text{C3bH}}[\text{IgGC3bC3bBb}][\text{H}] - k^{-}_{\text{C3bH}}[\text{IgGC3bC3bBbH}] - k^{-}_{\text{C3bBbH}_{\text{decay}}}[\text{IgGC3bC3bBbH}]$$

S173

$$\frac{d[\text{IgGC3bC3bBbHL}]}{dt} = k^{+}_{\text{C3bHL}}[\text{IgGC3bC3bBb}][\text{HL}] - k^{-}_{\text{C3bHL}}[\text{IgGC3bC3bBbHL}] - k^{-}_{\text{C3bBbHL}_{\text{decay}}}[\text{IgGC3bC3bBbHL}]$$

S174

$$\frac{d[\text{IgGC3bC3bBbCR1}]}{dt} = k^{+}_{\text{C3bC3bCR1}}[\text{IgGC3bC3bBb}][\text{CR1}] - k^{-}_{\text{C3bC3bCR1}}[\text{IgGC3bC3bBbCR1}] - k^{-}_{\text{C3bBbCR1}_{\text{decay}}}[\text{IgGC3bC3bBbCR1}]$$

S175

$$\frac{d[\text{C1* C1 - INH}]}{dt} = k^{+}_{\text{C1*C1-INH}}[\text{C1*}][\text{C1 - INH}]$$

S176

$$\frac{d[\text{nfC4bC4BP}]}{dt} = k^{+}_{\text{C4bC4BP}}[\text{nfC4b}][\text{C4BP}]$$

$$- \left(\frac{k^{\text{FI}}_{\text{catC3bH}}[\text{I}][\text{nfC4bC4BP}]}{K^{\text{FI}}_{\text{mC3bH}} + [\text{C3(H}_2\text{O)H}] + [\text{C3(H}_2\text{O)HL}] + [\text{fC3bH}] + [\text{fC3bHL}] + [\text{fC3bCR1}] + [\text{fiC3bCR1}] + [\text{IgGC3bC3bH}] + [\text{IgGC3biC3bH}] + [\text{IgGC3bC3bHL}]} \cdots \right.$$
$$\frac{1}{+ [\text{IgGC3biC3bHL}] + [\text{IgGC3bC3bCR1}] + [\text{IgGC3biC3bCR1}] + [\text{IgGiC3biC3bCR1}] + [\text{IgGiC3bC3dgCR1}] + [\text{fC3bC4bH}] + [\text{fC3bC4bHL}]} \cdots$$
$$\frac{1}{+ [\text{fC3bC4bCR1}] + [\text{fC3bC4bC4BP}] + [\text{fC3bC4dH}] + [\text{fC3bC4dHL}] + [\text{fC3bC4dCR1}] + [\text{fiC3bC4dCR1}] + [\text{fiC3bC4bCR1}] + [\text{fiC3bC4bC4BP}]} \cdots$$
$$\frac{1}{+ [\text{fC3dgC4bCR1}] + [\text{fC3dgC4bC4BP}] + [\text{fC3bC3bH}] + [\text{fC3bC3bHL}] + [\text{fC3bC3bCR1}] + [\text{fC3biC3bH}] + [\text{fC3biC3bHL}] + [\text{fC3biC3bCR1}]} \cdots$$
$$\frac{1}{+ [\text{fiC3biC3bCR1}] + [\text{fiC3bC3dgCR1}] + [\text{hC3bH}] + [\text{hC3bHL}] + [\text{hC3bCR1}] + [\text{hiC3bCR1}] + [\text{hC3bC4bH}] + [\text{hC3bC4bHL}] + [\text{hC3bC4bCR1}]} \cdots$$
$$\frac{1}{+ [\text{hC3bC4bC4BP}] + [\text{hC3bC4dH}] + [\text{hC3bC4dHL}] + [\text{hC3bC4dCR1}] + [\text{hiC3bC4dCR1}] + [\text{hiC3bC4bCR1}] + [\text{hiC3bC4bC4BP}] + [\text{hC3dgC4bCR1}]} \cdots$$
$$\frac{1}{+ [\text{hC3dgC4bC4BP}] + [\text{hC3bC3bH}] + [\text{hC3bC3bHL}] + [\text{hC3bC3bCR1}] + [\text{hC3biC3bH}] + [\text{hC3biC3bHL}] + [\text{hC3biC3bCR1}] + [\text{hiC3biC3bCR1}]} \cdots$$
$$\frac{1}{+ [\text{hiC3bC3dgCR1}] + [\text{fC4bCR1}] + [\text{nfC4bC4BP}] + [\text{fC4bC4BP}] + [\text{fC4bC4bCR1}] + [\text{fC4bC4bC4BP}] + [\text{fC4bC4dCR1}] + [\text{fC4bC4dC4BP}] + [\text{hC4bCR1}]} \cdots$$
$$\left. \frac{1}{+ [\text{hC4bC4BP}] + [\text{hC4bC4bCR1}] + [\text{hC4bC4bC4BP}] + [\text{hC4bC4dCR1}] + [\text{hC4bC4dC4BP}]} \cdots \right)$$

S177

$$\frac{d[\mathrm{fC4dC4BP}]}{dt} =$$

$$\left(\frac{k^{\mathrm{FI}}_{\mathrm{catC3bH}}[\mathrm{I}][\mathrm{nfC4bC4BP}]}{K^{\mathrm{FI}}_{\mathrm{mC3bH}} + [\mathrm{C3(H_2O)H}] + [\mathrm{C3(H_2O)HL}] + [\mathrm{fC3bH}] + [\mathrm{fC3bHL}] + [\mathrm{fC3bCR1}] + [\mathrm{fiC3bCR1}] + [\mathrm{IgGC3bC3bH}] + [\mathrm{IgGC3biC3bH}] + [\mathrm{IgGC3bC3bHL}]} \cdots \right.$$

$$\frac{1}{+ [\mathrm{IgGC3biC3bHL}] + [\mathrm{IgGC3bC3bCR1}] + [\mathrm{IgGC3biC3bCR1}] + [\mathrm{IgGiC3biC3bCR1}] + [\mathrm{IgGiC3bC3dgCR1}] + [\mathrm{fC3bC4bH}] + [\mathrm{fC3bC4bHL}]} \cdots$$

$$\frac{1}{+ [\mathrm{fC3bC4bCR1}] + [\mathrm{fC3bC4bC4BP}] + [\mathrm{fC3bC4dH}] + [\mathrm{fC3bC4dHL}] + [\mathrm{fC3bC4dCR1}] + [\mathrm{fiC3bC4dCR1}] + [\mathrm{fiC3bC4bCR1}] + [\mathrm{fiC3bC4bC4BP}]} \cdots$$

$$\frac{1}{+ [\mathrm{fC3dgC4bCR1}] + [\mathrm{fC3dgC4bC4BP}] + [\mathrm{fC3bC3bH}] + [\mathrm{fC3bC3bHL}] + [\mathrm{fC3bC3bCR1}] + [\mathrm{fC3biC3bH}] + [\mathrm{fC3biC3bHL}] + [\mathrm{fC3biC3bCR1}]} \cdots$$

$$\frac{1}{+ [\mathrm{fiC3biC3bCR1}] + [\mathrm{fiC3bC3dgCR1}] + [\mathrm{hC3bH}] + [\mathrm{hC3bHL}] + [\mathrm{hC3bCR1}] + [\mathrm{hiC3bCR1}] + [\mathrm{hC3bC4bH}] + [\mathrm{hC3bC4bHL}] + [\mathrm{hC3bC4bCR1}]} \cdots$$

$$\frac{1}{+ [\mathrm{hC3bC4bC4BP}] + [\mathrm{hC3bC4dH}] + [\mathrm{hC3bC4dHL}] + [\mathrm{hC3bC4dCR1}] + [\mathrm{hiC3bC4dCR1}] + [\mathrm{hiC3bC4bCR1}] + [\mathrm{hiC3bC4bC4BP}] + [\mathrm{hC3dgC4bCR1}]} \cdots$$

$$\frac{1}{+ [\mathrm{hC3dgC4bC4BP}] + [\mathrm{hC3bC3bH}] + [\mathrm{hC3bC3bHL}] + [\mathrm{hC3bC3bCR1}] + [\mathrm{hC3biC3bH}] + [\mathrm{hC3biC3bHL}] + [\mathrm{hC3biC3bCR1}] + [\mathrm{hiC3biC3bCR1}]} \cdots$$

$$\frac{1}{+ [\mathrm{hiC3bC3dgCR1}] + [\mathrm{fC4bCR1}] + [\mathrm{nfC4bC4BP}] + [\mathrm{fC4bC4BP}] + [\mathrm{fC4bC4bCR1}] + [\mathrm{fC4bC4bC4BP}] + [\mathrm{fC4bC4dCR1}] + [\mathrm{fC4bC4dC4BP}] + [\mathrm{hC4bCR1}]} \cdots$$

$$\left. \frac{1}{+ [\mathrm{hC4bC4BP}] + [\mathrm{hC4bC4bCR1}] + [\mathrm{hC4bC4bC4BP}] + [\mathrm{hC4bC4dCR1}] + [\mathrm{hC4bC4dC4BP}]} \cdots \right)$$

S178

$$\frac{d[\mathrm{fC4bC4BP}]}{dt} = k^{+}_{\mathrm{C4bC4BP}}[\mathrm{fC4b}][\mathrm{C4BP}] - k^{-}_{\mathrm{C4bC4BP}}[\mathrm{fC4bC4BP}]$$

$$- \left(\frac{k^{\mathrm{FI}}_{\mathrm{catC3bH}}[\mathrm{I}][\mathrm{fC4bC4BP}]}{K^{\mathrm{FI}}_{\mathrm{mC3bH}} + [\mathrm{C3(H_2O)H}] + [\mathrm{C3(H_2O)HL}] + [\mathrm{fC3bH}] + [\mathrm{fC3bHL}] + [\mathrm{fC3bCR1}] + [\mathrm{fiC3bCR1}] + [\mathrm{IgGC3bC3bH}] + [\mathrm{IgGC3biC3bH}] + [\mathrm{IgGC3bC3bHL}]} \cdots \right.$$

$$\frac{1}{+ [\mathrm{IgGC3biC3bHL}] + [\mathrm{IgGC3bC3bCR1}] + [\mathrm{IgGC3biC3bCR1}] + [\mathrm{IgGiC3biC3bCR1}] + [\mathrm{IgGiC3bC3dgCR1}] + [\mathrm{fC3bC4bH}] + [\mathrm{fC3bC4bHL}]} \cdots$$

$$\frac{1}{+ [\mathrm{fC3bC4bCR1}] + [\mathrm{fC3bC4bC4BP}] + [\mathrm{fC3bC4dH}] + [\mathrm{fC3bC4dHL}] + [\mathrm{fC3bC4dCR1}] + [\mathrm{fiC3bC4dCR1}] + [\mathrm{fiC3bC4bCR1}] + [\mathrm{fiC3bC4bC4BP}]} \cdots$$

$$\frac{1}{+ [\mathrm{fC3dgC4bCR1}] + [\mathrm{fC3dgC4bC4BP}] + [\mathrm{fC3bC3bH}] + [\mathrm{fC3bC3bHL}] + [\mathrm{fC3bC3bCR1}] + [\mathrm{fC3biC3bH}] + [\mathrm{fC3biC3bHL}] + [\mathrm{fC3biC3bCR1}]} \cdots$$

$$\frac{1}{+ [\mathrm{fiC3biC3bCR1}] + [\mathrm{fiC3bC3dgCR1}] + [\mathrm{hC3bH}] + [\mathrm{hC3bHL}] + [\mathrm{hC3bCR1}] + [\mathrm{hiC3bCR1}] + [\mathrm{hC3bC4bH}] + [\mathrm{hC3bC4bHL}] + [\mathrm{hC3bC4bCR1}]} \cdots$$

$$\frac{1}{+ [\mathrm{hC3bC4bC4BP}] + [\mathrm{hC3bC4dH}] + [\mathrm{hC3bC4dHL}] + [\mathrm{hC3bC4dCR1}] + [\mathrm{hiC3bC4dCR1}] + [\mathrm{hiC3bC4bCR1}] + [\mathrm{hiC3bC4bC4BP}] + [\mathrm{hC3dgC4bCR1}]} \cdots$$

$$\frac{1}{+ [\mathrm{hC3dgC4bC4BP}] + [\mathrm{hC3bC3bH}] + [\mathrm{hC3bC3bHL}] + [\mathrm{hC3bC3bCR1}] + [\mathrm{hC3biC3bH}] + [\mathrm{hC3biC3bHL}] + [\mathrm{hC3biC3bCR1}] + [\mathrm{hiC3biC3bCR1}]} \cdots$$

$$\frac{1}{+ [\mathrm{hiC3bC3dgCR1}] + [\mathrm{fC4bCR1}] + [\mathrm{nfC4bC4BP}] + [\mathrm{fC4bC4BP}] + [\mathrm{fC4bC4bCR1}] + [\mathrm{fC4bC4bC4BP}] + [\mathrm{fC4bC4dCR1}] + [\mathrm{fC4bC4dC4BP}] + [\mathrm{hC4bCR1}]} \cdots$$

$$\left. \frac{1}{+ [\mathrm{hC4bC4BP}] + [\mathrm{hC4bC4bCR1}] + [\mathrm{hC4bC4bC4BP}] + [\mathrm{hC4bC4dCR1}] + [\mathrm{hC4bC4dC4BP}]} \cdots \right)$$

S179

$$\frac{d[\mathrm{fC4bCR1}]}{dt} = k^{+}_{\mathrm{C4bCR1}}[\mathrm{fC4b}][\mathrm{CR1}] - k^{-}_{\mathrm{C4bCR1}}[\mathrm{fC4bCR1}]$$

$$-\left(\begin{array}{l}
\frac{k^{\mathrm{FI}}_{\mathrm{catC3bH}}[\mathrm{I}][\mathrm{fC4bCR1}]}{K^{\mathrm{FI}}_{\mathrm{mC3bH}} + [\mathrm{C3(H_2O)H}] + [\mathrm{C3(H_2O)HL}] + [\mathrm{fC3bH}] + [\mathrm{fC3bHL}] + [\mathrm{fC3bCR1}] + [\mathrm{fiC3bCR1}] + [\mathrm{IgGC3bC3bH}] + [\mathrm{IgGC3biC3bH}] + [\mathrm{IgGC3bC3bHL}]}\cdots \\
\frac{1}{+[\mathrm{IgGC3biC3bHL}] + [\mathrm{IgGC3bC3bCR1}] + [\mathrm{IgGC3biC3bCR1}] + [\mathrm{IgGiC3biC3bCR1}] + [\mathrm{IgGiC3bC3dgCR1}] + [\mathrm{fC3bC4bH}] + [\mathrm{fC3bC4bHL}]}\cdots \\
\frac{1}{+[\mathrm{fC3bC4bCR1}] + [\mathrm{fC3bC4bC4BP}] + [\mathrm{fC3bC4dH}] + [\mathrm{fC3bC4dHL}] + [\mathrm{fC3bC4dCR1}] + [\mathrm{fiC3bC4dCR1}] + [\mathrm{fiC3bC4bCR1}] + [\mathrm{fiC3bC4bC4BP}]}\cdots \\
\frac{1}{+[\mathrm{fC3dgC4bCR1}] + [\mathrm{fC3dgC4bC4BP}] + [\mathrm{fC3bC3bH}] + [\mathrm{fC3bC3bHL}] + [\mathrm{fC3bC3bCR1}] + [\mathrm{fC3biC3bH}] + [\mathrm{fC3biC3bHL}] + [\mathrm{fC3biC3bCR1}]}\cdots \\
\frac{1}{+[\mathrm{fiC3biC3bCR1}] + [\mathrm{fiC3bC3dgCR1}] + [\mathrm{hC3bH}] + [\mathrm{hC3bHL}] + [\mathrm{hC3bCR1}] + [\mathrm{hiC3bCR1}] + [\mathrm{hC3bC4bH}] + [\mathrm{hC3bC4bHL}] + [\mathrm{hC3bC4bCR1}]}\cdots \\
\frac{1}{+[\mathrm{hC3bC4bC4BP}] + [\mathrm{hC3bC4dH}] + [\mathrm{hC3bC4dHL}] + [\mathrm{hC3bC4dCR1}] + [\mathrm{hiC3bC4dCR1}] + [\mathrm{hiC3bC4bCR1}] + [\mathrm{hiC3bC4bC4BP}] + [\mathrm{hC3dgC4bCR1}]}\cdots \\
\frac{1}{+[\mathrm{hC3dgC4bC4BP}] + [\mathrm{hC3bC3bH}] + [\mathrm{hC3bC3bHL}] + [\mathrm{hC3bC3bCR1}] + [\mathrm{hC3biC3bH}] + [\mathrm{hC3biC3bHL}] + [\mathrm{hC3biC3bCR1}] + [\mathrm{hiC3biC3bCR1}]}\cdots \\
\frac{1}{+[\mathrm{hiC3bC3dgCR1}] + [\mathrm{fC4bCR1}] + [\mathrm{nfC4bC4BP}] + [\mathrm{fC4bC4BP}] + [\mathrm{fC4bC4bCR1}] + [\mathrm{fC4bC4bC4BP}] + [\mathrm{fC4bC4dCR1}] + [\mathrm{fC4bC4dC4BP}] + [\mathrm{hC4bCR1}]}\cdots \\
\frac{1}{+[\mathrm{hC4bC4BP}] + [\mathrm{hC4bC4bCR1}] + [\mathrm{hC4bC4bC4BP}] + [\mathrm{hC4bC4dCR1}] + [\mathrm{hC4bC4dC4BP}]}\cdots
\end{array}\right)$$

S180

$$\frac{d[\mathrm{fC4d}]}{dt} =$$

$$+\left(\begin{array}{l}
\frac{k^{\mathrm{FI}}_{\mathrm{catC3bH}}[\mathrm{I}][\mathrm{fC4bC4BP}]}{K^{\mathrm{FI}}_{\mathrm{mC3bH}} + [\mathrm{C3(H_2O)H}] + [\mathrm{C3(H_2O)HL}] + [\mathrm{fC3bH}] + [\mathrm{fC3bHL}] + [\mathrm{fC3bCR1}] + [\mathrm{fiC3bCR1}] + [\mathrm{IgGC3bC3bH}] + [\mathrm{IgGC3biC3bH}] + [\mathrm{IgGC3bC3bHL}]}\cdots \\
\frac{1}{+[\mathrm{IgGC3biC3bHL}] + [\mathrm{IgGC3bC3bCR1}] + [\mathrm{IgGC3biC3bCR1}] + [\mathrm{IgGiC3biC3bCR1}] + [\mathrm{IgGiC3bC3dgCR1}] + [\mathrm{fC3bC4bH}] + [\mathrm{fC3bC4bHL}]}\cdots \\
\frac{1}{+[\mathrm{fC3bC4bCR1}] + [\mathrm{fC3bC4bC4BP}] + [\mathrm{fC3bC4dH}] + [\mathrm{fC3bC4dHL}] + [\mathrm{fC3bC4dCR1}] + [\mathrm{fiC3bC4dCR1}] + [\mathrm{fiC3bC4bCR1}] + [\mathrm{fiC3bC4bC4BP}]}\cdots \\
\frac{1}{+[\mathrm{fC3dgC4bCR1}] + [\mathrm{fC3dgC4bC4BP}] + [\mathrm{fC3bC3bH}] + [\mathrm{fC3bC3bHL}] + [\mathrm{fC3bC3bCR1}] + [\mathrm{fC3biC3bH}] + [\mathrm{fC3biC3bHL}] + [\mathrm{fC3biC3bCR1}]}\cdots \\
\frac{1}{+[\mathrm{fiC3biC3bCR1}] + [\mathrm{fiC3bC3dgCR1}] + [\mathrm{hC3bH}] + [\mathrm{hC3bHL}] + [\mathrm{hC3bCR1}] + [\mathrm{hiC3bCR1}] + [\mathrm{hC3bC4bH}] + [\mathrm{hC3bC4bHL}] + [\mathrm{hC3bC4bCR1}]}\cdots \\
\frac{1}{+[\mathrm{hC3bC4bC4BP}] + [\mathrm{hC3bC4dH}] + [\mathrm{hC3bC4dHL}] + [\mathrm{hC3bC4dCR1}] + [\mathrm{hiC3bC4dCR1}] + [\mathrm{hiC3bC4bCR1}] + [\mathrm{hiC3bC4bC4BP}] + [\mathrm{hC3dgC4bCR1}]}\cdots \\
\frac{1}{+[\mathrm{hC3dgC4bC4BP}] + [\mathrm{hC3bC3bH}] + [\mathrm{hC3bC3bHL}] + [\mathrm{hC3bC3bCR1}] + [\mathrm{hC3biC3bH}] + [\mathrm{hC3biC3bHL}] + [\mathrm{hC3biC3bCR1}] + [\mathrm{hiC3biC3bCR1}]}\cdots \\
\frac{1}{+[\mathrm{hiC3bC3dgCR1}] + [\mathrm{fC4bCR1}] + [\mathrm{nfC4bC4BP}] + [\mathrm{fC4bC4BP}] + [\mathrm{fC4bC4bCR1}] + [\mathrm{fC4bC4bC4BP}] + [\mathrm{fC4bC4dCR1}] + [\mathrm{fC4bC4dC4BP}] + [\mathrm{hC4bCR1}]}\cdots \\
\frac{1}{+[\mathrm{hC4bC4BP}] + [\mathrm{hC4bC4bCR1}] + [\mathrm{hC4bC4bC4BP}] + [\mathrm{hC4bC4dCR1}] + [\mathrm{hC4bC4dC4BP}]}\cdots
\end{array}\right)$$

$$
+\left(\frac{k_{\text{catC3bH}}^{\text{FI}}[\text{I}][\text{fC4bCR1}]}{K_{\text{mC3bH}}^{\text{FI}}+[\text{C3(H}_2\text{O)H}]+[\text{C3(H}_2\text{O)HL}]+[\text{fC3bH}]+[\text{fC3bHL}]+[\text{fC3bCR1}]+[\text{fiC3bCR1}]+[\text{IgGC3bC3bH}]+[\text{IgGC3biC3bH}]+[\text{IgGC3bC3bHL}]}\cdots
\frac{1}{+[\text{IgGC3biC3bHL}]+[\text{IgGC3bC3bCR1}]+[\text{IgGC3biC3bCR1}]+[\text{IgGiC3biC3bCR1}]+[\text{IgGiC3bC3dgCR1}]+[\text{fC3bC4bH}]+[\text{fC3bC4bHL}]}\cdots
\frac{1}{+[\text{fC3bC4bCR1}]+[\text{fC3bC4bC4BP}]+[\text{fC3bC4dH}]+[\text{fC3bC4dHL}]+[\text{fC3bC4dCR1}]+[\text{fiC3bC4dCR1}]+[\text{fiC3bC4bCR1}]+[\text{fiC3bC4bC4BP}]}\cdots
\frac{1}{+[\text{fC3dgC4bCR1}]+[\text{fC3dgC4bC4BP}]+[\text{fC3bC3bH}]+[\text{fC3bC3bHL}]+[\text{fC3bC3bCR1}]+[\text{fC3biC3bH}]+[\text{fC3biC3bHL}]+[\text{fC3biC3bCR1}]}\cdots
\frac{1}{+[\text{fiC3biC3bCR1}]+[\text{fiC3bC3dgCR1}]+[\text{hC3bH}]+[\text{hC3bHL}]+[\text{hC3bCR1}]+[\text{hiC3bCR1}]+[\text{hC3bC4bH}]+[\text{hC3bC4bHL}]+[\text{hC3bC4bCR1}]}\cdots
\frac{1}{+[\text{hC3bC4bC4BP}]+[\text{hC3bC4dH}]+[\text{hC3bC4dHL}]+[\text{hC3bC4dCR1}]+[\text{hiC3bC4dCR1}]+[\text{hiC3bC4bCR1}]+[\text{hiC3bC4bC4BP}]+[\text{hC3dgC4bCR1}]}\cdots
\frac{1}{+[\text{hC3dgC4bC4BP}]+[\text{hC3bC3bH}]+[\text{hC3bC3bHL}]+[\text{hC3bC3bCR1}]+[\text{hC3biC3bH}]+[\text{hC3biC3bHL}]+[\text{hC3biC3bCR1}]+[\text{hiC3biC3bCR1}]}\cdots
\frac{1}{+[\text{hiC3bC3dgCR1}]+[\text{fC4bCR1}]+[\text{nfC4bC4BP}]+[\text{fC4bC4BP}]+[\text{fC4bC4bCR1}]+[\text{fC4bC4bC4BP}]+[\text{fC4bC4dCR1}]+[\text{fC4bC4dC4BP}]+[\text{hC4bCR1}]}\cdots
\frac{1}{+[\text{hC4bC4BP}]+[\text{hC4bC4bCR1}]+[\text{hC4bC4bC4BP}]+[\text{hC4bC4dCR1}]+[\text{hC4bC4dC4BP}]}\cdots\right)
$$

S181

$$
\frac{d[\text{fC4bC2aC4BP}]}{dt}=k_{\text{C4bC4BP}}^{+}[\text{fC4bC2a}][\text{C4BP}]-k_{\text{C4bC4BP}}^{-}[\text{fC4bC2aC4BP}]-k_{\text{C4bC2aC4BP}_{\text{decay}}}^{-}[\text{fC4bC2aC4BP}]
$$

S182

$$
\frac{d[\text{fC4bC2aCR1}]}{dt}=k_{\text{C4bCR1}}^{+}[\text{fC4bC2a}][\text{CR1}]-k_{\text{C4bCR1}}^{-}[\text{fC4bC2aCR1}]-k_{\text{C4bC2aCR1}_{\text{decay}}}^{-}[\text{fC4bC2aCR1}]
$$

S183

$$
\frac{d[\text{fC4bC4bC2aC4BP}]}{dt}=k_{\text{C4bC4BP}}^{+}[\text{fC4bC4bC2a}][\text{C4BP}]-k_{\text{C4bC4BP}}^{-}[\text{fC4bC4bC2aC4BP}]-k_{\text{C4bC2aC4BP}_{\text{decay}}}^{-}[\text{fC4bC4bC2aC4BP}]
$$

S184

$$
\frac{d[\text{fC4bC4bC2aCR1}]}{dt}=k_{\text{C4bC4bCR1}}^{+}[\text{fC4bC4bC2a}][\text{CR1}]-k_{\text{C4bC4bCR1}}^{-}[\text{fC4bC4bC2aCR1}]-k_{\text{C4bC2aCR1}_{\text{decay}}}^{-}[\text{fC4bC4bC2aCR1}]
$$

S185

$$\frac{d[\mathrm{fC4bC4bC4BP}]}{dt} = k^{+}_{\mathrm{C4bC4BP}}[\mathrm{fC4bC4b}][\mathrm{C4BP}] - k^{-}_{\mathrm{C4bC4BP}}[\mathrm{fC4bC4bC4BP}]$$

$$- \left(\frac{k^{\mathrm{FI}}_{\mathrm{catC3bH}}[\mathrm{I}][\mathrm{fC4bC4bC4BP}]}{K^{\mathrm{FI}}_{\mathrm{mC3bH}} + [\mathrm{C3(H_2O)H}] + [\mathrm{C3(H_2O)HL}] + [\mathrm{fC3bH}] + [\mathrm{fC3bHL}] + [\mathrm{fC3bCR1}] + [\mathrm{fiC3bCR1}] + [\mathrm{IgGC3bC3bH}] + [\mathrm{IgGC3biC3bH}] + [\mathrm{IgGC3bC3bHL}]} \cdots \right.$$

$$\frac{1}{+[\mathrm{IgGC3biC3bHL}] + [\mathrm{IgGC3bC3bCR1}] + [\mathrm{IgGC3biC3bCR1}] + [\mathrm{IgGiC3biC3bCR1}] + [\mathrm{IgGiC3bC3dgCR1}] + [\mathrm{fC3bC4bH}] + [\mathrm{fC3bC4bHL}]} \cdots$$

$$\frac{1}{+[\mathrm{fC3bC4bCR1}] + [\mathrm{fC3bC4bC4BP}] + [\mathrm{fC3bC4dH}] + [\mathrm{fC3bC4dHL}] + [\mathrm{fC3bC4dCR1}] + [\mathrm{fiC3bC4dCR1}] + [\mathrm{fiC3bC4bCR1}] + [\mathrm{fiC3bC4bC4BP}]} \cdots$$

$$\frac{1}{+[\mathrm{fC3dgC4bCR1}] + [\mathrm{fC3dgC4bC4BP}] + [\mathrm{fC3bC3bH}] + [\mathrm{fC3bC3bHL}] + [\mathrm{fC3bC3bCR1}] + [\mathrm{fC3biC3bH}] + [\mathrm{fC3biC3bHL}] + [\mathrm{fC3biC3bCR1}]} \cdots$$

$$\frac{1}{+[\mathrm{fiC3biC3bCR1}] + [\mathrm{fiC3bC3dgCR1}] + [\mathrm{hC3bH}] + [\mathrm{hC3bHL}] + [\mathrm{hC3bCR1}] + [\mathrm{hiC3bCR1}] + [\mathrm{hC3bC4bH}] + [\mathrm{hC3bC4bHL}] + [\mathrm{hC3bC4bCR1}]} \cdots$$

$$\frac{1}{+[\mathrm{hC3bC4bC4BP}] + [\mathrm{hC3bC4dH}] + [\mathrm{hC3bC4dHL}] + [\mathrm{hC3bC4dCR1}] + [\mathrm{hiC3bC4dCR1}] + [\mathrm{hiC3bC4bCR1}] + [\mathrm{hiC3bC4bC4BP}] + [\mathrm{hC3dgC4bCR1}]} \cdots$$

$$\frac{1}{+[\mathrm{hC3dgC4bC4BP}] + [\mathrm{hC3bC3bH}] + [\mathrm{hC3bC3bHL}] + [\mathrm{hC3bC3bCR1}] + [\mathrm{hC3biC3bH}] + [\mathrm{hC3biC3bHL}] + [\mathrm{hC3biC3bCR1}] + [\mathrm{hiC3biC3bCR1}]} \cdots$$

$$\frac{1}{+[\mathrm{hiC3bC3dgCR1}] + [\mathrm{fC4bCR1}] + [\mathrm{nfC4bC4BP}] + [\mathrm{fC4bC4BP}] + [\mathrm{fC4bC4bCR1}] + [\mathrm{fC4bC4bC4BP}] + [\mathrm{fC4bC4dCR1}] + [\mathrm{fC4bC4dC4BP}] + [\mathrm{hC4bCR1}]} \cdots$$

$$\left. \frac{1}{+[\mathrm{hC4bC4BP}] + [\mathrm{hC4bC4bCR1}] + [\mathrm{hC4bC4bC4BP}] + [\mathrm{hC4bC4dCR1}] + [\mathrm{hC4bC4dC4BP}]} \cdots \right)$$

S186

$$\frac{d[\mathrm{fC4bC4bCR1}]}{dt} = k^{+}_{\mathrm{C4bC4bCR1}}[\mathrm{fC4bC4b}][\mathrm{CR1}] - k^{-}_{\mathrm{C4bC4bCR1}}[\mathrm{fC4bC4bCR1}]$$

$$- \left(\frac{k^{\mathrm{FI}}_{\mathrm{catC3bH}}[\mathrm{I}][\mathrm{fC4bC4bCR1}]}{K^{\mathrm{FI}}_{\mathrm{mC3bH}} + [\mathrm{C3(H_2O)H}] + [\mathrm{C3(H_2O)HL}] + [\mathrm{fC3bH}] + [\mathrm{fC3bHL}] + [\mathrm{fC3bCR1}] + [\mathrm{fiC3bCR1}] + [\mathrm{IgGC3bC3bH}] + [\mathrm{IgGC3biC3bH}] + [\mathrm{IgGC3bC3bHL}]} \cdots \right.$$

$$\frac{1}{+[\mathrm{IgGC3biC3bHL}] + [\mathrm{IgGC3bC3bCR1}] + [\mathrm{IgGC3biC3bCR1}] + [\mathrm{IgGiC3biC3bCR1}] + [\mathrm{IgGiC3bC3dgCR1}] + [\mathrm{fC3bC4bH}] + [\mathrm{fC3bC4bHL}]} \cdots$$

$$\frac{1}{+[\mathrm{fC3bC4bCR1}] + [\mathrm{fC3bC4bC4BP}] + [\mathrm{fC3bC4dH}] + [\mathrm{fC3bC4dHL}] + [\mathrm{fC3bC4dCR1}] + [\mathrm{fiC3bC4dCR1}] + [\mathrm{fiC3bC4bCR1}] + [\mathrm{fiC3bC4bC4BP}]} \cdots$$

$$\frac{1}{+[\mathrm{fC3dgC4bCR1}] + [\mathrm{fC3dgC4bC4BP}] + [\mathrm{fC3bC3bH}] + [\mathrm{fC3bC3bHL}] + [\mathrm{fC3bC3bCR1}] + [\mathrm{fC3biC3bH}] + [\mathrm{fC3biC3bHL}] + [\mathrm{fC3biC3bCR1}]} \cdots$$

$$\frac{1}{+[\mathrm{fiC3biC3bCR1}] + [\mathrm{fiC3bC3dgCR1}] + [\mathrm{hC3bH}] + [\mathrm{hC3bHL}] + [\mathrm{hC3bCR1}] + [\mathrm{hiC3bCR1}] + [\mathrm{hC3bC4bH}] + [\mathrm{hC3bC4bHL}] + [\mathrm{hC3bC4bCR1}]} \cdots$$

$$\frac{1}{+[\mathrm{hC3bC4bC4BP}] + [\mathrm{hC3bC4dH}] + [\mathrm{hC3bC4dHL}] + [\mathrm{hC3bC4dCR1}] + [\mathrm{hiC3bC4dCR1}] + [\mathrm{hiC3bC4bCR1}] + [\mathrm{hiC3bC4bC4BP}] + [\mathrm{hC3dgC4bCR1}]} \cdots$$

$$\frac{1}{+[\mathrm{hC3dgC4bC4BP}] + [\mathrm{hC3bC3bH}] + [\mathrm{hC3bC3bHL}] + [\mathrm{hC3bC3bCR1}] + [\mathrm{hC3biC3bH}] + [\mathrm{hC3biC3bHL}] + [\mathrm{hC3biC3bCR1}] + [\mathrm{hiC3biC3bCR1}]} \cdots$$

$$\frac{1}{+[\mathrm{hiC3bC3dgCR1}] + [\mathrm{fC4bCR1}] + [\mathrm{nfC4bC4BP}] + [\mathrm{fC4bC4BP}] + [\mathrm{fC4bC4bCR1}] + [\mathrm{fC4bC4bC4BP}] + [\mathrm{fC4bC4dCR1}] + [\mathrm{fC4bC4dC4BP}] + [\mathrm{hC4bCR1}]} \cdots$$

$$\left. \frac{1}{+[\mathrm{hC4bC4BP}] + [\mathrm{hC4bC4bCR1}] + [\mathrm{hC4bC4bC4BP}] + [\mathrm{hC4bC4dCR1}] + [\mathrm{hC4bC4dC4BP}]} \cdots \right)$$

S187

$$\frac{d[\mathrm{fC4bC4d}]}{dt} =$$

$$\left(\begin{array}{l}
\frac{k_{\mathrm{catC3bH}}^{\mathrm{FI}}[\mathrm{I}][\mathrm{fC4bC4bC4BP}]}{K_{\mathrm{mC3bH}}^{\mathrm{FI}} + [\mathrm{C3(H_2O)H}] + [\mathrm{C3(H_2O)HL}] + [\mathrm{fC3bH}] + [\mathrm{fC3bHL}] + [\mathrm{fC3bCR1}] + [\mathrm{fiC3bCR1}] + [\mathrm{IgGC3bC3bH}] + [\mathrm{IgGC3biC3bH}] + [\mathrm{IgGC3bC3bHL}]} \cdots \\
\frac{1}{+ [\mathrm{IgGC3biC3bHL}] + [\mathrm{IgGC3bC3bCR1}] + [\mathrm{IgGC3biC3bCR1}] + [\mathrm{IgGiC3biC3bCR1}] + [\mathrm{IgGiC3bC3dgCR1}] + [\mathrm{fC3bC4bH}] + [\mathrm{fC3bC4bHL}]} \cdots \\
\frac{1}{+ [\mathrm{fC3bC4bCR1}] + [\mathrm{fC3bC4bC4BP}] + [\mathrm{fC3bC4dH}] + [\mathrm{fC3bC4dHL}] + [\mathrm{fC3bC4dCR1}] + [\mathrm{fiC3bC4dCR1}] + [\mathrm{fiC3bC4bCR1}] + [\mathrm{fiC3bC4bC4BP}]} \cdots \\
\frac{1}{+ [\mathrm{fC3dgC4bCR1}] + [\mathrm{fC3dgC4bC4BP}] + [\mathrm{fC3bC3bH}] + [\mathrm{fC3bC3bHL}] + [\mathrm{fC3bC3bCR1}] + [\mathrm{fC3biC3bH}] + [\mathrm{fC3biC3bHL}] + [\mathrm{fC3biC3bCR1}]} \cdots \\
\frac{1}{+ [\mathrm{fiC3biC3bCR1}] + [\mathrm{fiC3bC3dgCR1}] + [\mathrm{hC3bH}] + [\mathrm{hC3bHL}] + [\mathrm{hC3bCR1}] + [\mathrm{hiC3bCR1}] + [\mathrm{hC3bC4bH}] + [\mathrm{hC3bC4bHL}] + [\mathrm{hC3bC4bCR1}]} \cdots \\
\frac{1}{+ [\mathrm{hC3bC4bC4BP}] + [\mathrm{hC3bC4dH}] + [\mathrm{hC3bC4dHL}] + [\mathrm{hC3bC4dCR1}] + [\mathrm{hiC3bC4dCR1}] + [\mathrm{hiC3bC4bCR1}] + [\mathrm{hiC3bC4bC4BP}] + [\mathrm{hC3dgC4bCR1}]} \cdots \\
\frac{1}{+ [\mathrm{hC3dgC4bC4BP}] + [\mathrm{hC3bC3bH}] + [\mathrm{hC3bC3bHL}] + [\mathrm{hC3bC3bCR1}] + [\mathrm{hC3biC3bH}] + [\mathrm{hC3biC3bHL}] + [\mathrm{hC3biC3bCR1}] + [\mathrm{hiC3biC3bCR1}]} \cdots \\
\frac{1}{+ [\mathrm{hiC3bC3dgCR1}] + [\mathrm{fC4bCR1}] + [\mathrm{nfC4bC4BP}] + [\mathrm{fC4bC4BP}] + [\mathrm{fC4bC4bCR1}] + [\mathrm{fC4bC4bC4BP}] + [\mathrm{fC4bC4dCR1}] + [\mathrm{fC4bC4dC4BP}] + [\mathrm{hC4bCR1}]} \cdots \\
\frac{1}{+ [\mathrm{hC4bC4BP}] + [\mathrm{hC4bC4bCR1}] + [\mathrm{hC4bC4bC4BP}] + [\mathrm{hC4bC4dCR1}] + [\mathrm{hC4bC4dC4BP}]} \cdots
\end{array}\right)$$

$$+\left(\begin{array}{l}
\frac{k_{\mathrm{catC3bH}}^{\mathrm{FI}}[\mathrm{I}][\mathrm{fC4bC4bCR1}]}{K_{\mathrm{mC3bH}}^{\mathrm{FI}} + [\mathrm{C3(H_2O)H}] + [\mathrm{C3(H_2O)HL}] + [\mathrm{fC3bH}] + [\mathrm{fC3bHL}] + [\mathrm{fC3bCR1}] + [\mathrm{fiC3bCR1}] + [\mathrm{IgGC3bC3bH}] + [\mathrm{IgGC3biC3bH}] + [\mathrm{IgGC3bC3bHL}]} \cdots \\
\frac{1}{+ [\mathrm{IgGC3biC3bHL}] + [\mathrm{IgGC3bC3bCR1}] + [\mathrm{IgGC3biC3bCR1}] + [\mathrm{IgGiC3biC3bCR1}] + [\mathrm{IgGiC3bC3dgCR1}] + [\mathrm{fC3bC4bH}] + [\mathrm{fC3bC4bHL}]} \cdots \\
\frac{1}{+ [\mathrm{fC3bC4bCR1}] + [\mathrm{fC3bC4bC4BP}] + [\mathrm{fC3bC4dH}] + [\mathrm{fC3bC4dHL}] + [\mathrm{fC3bC4dCR1}] + [\mathrm{fiC3bC4dCR1}] + [\mathrm{fiC3bC4bCR1}] + [\mathrm{fiC3bC4bC4BP}]} \cdots \\
\frac{1}{+ [\mathrm{fC3dgC4bCR1}] + [\mathrm{fC3dgC4bC4BP}] + [\mathrm{fC3bC3bH}] + [\mathrm{fC3bC3bHL}] + [\mathrm{fC3bC3bCR1}] + [\mathrm{fC3biC3bH}] + [\mathrm{fC3biC3bHL}] + [\mathrm{fC3biC3bCR1}]} \cdots \\
\frac{1}{+ [\mathrm{fiC3biC3bCR1}] + [\mathrm{fiC3bC3dgCR1}] + [\mathrm{hC3bH}] + [\mathrm{hC3bHL}] + [\mathrm{hC3bCR1}] + [\mathrm{hiC3bCR1}] + [\mathrm{hC3bC4bH}] + [\mathrm{hC3bC4bHL}] + [\mathrm{hC3bC4bCR1}]} \cdots \\
\frac{1}{+ [\mathrm{hC3bC4bC4BP}] + [\mathrm{hC3bC4dH}] + [\mathrm{hC3bC4dHL}] + [\mathrm{hC3bC4dCR1}] + [\mathrm{hiC3bC4dCR1}] + [\mathrm{hiC3bC4bCR1}] + [\mathrm{hiC3bC4bC4BP}] + [\mathrm{hC3dgC4bCR1}]} \cdots \\
\frac{1}{+ [\mathrm{hC3dgC4bC4BP}] + [\mathrm{hC3bC3bH}] + [\mathrm{hC3bC3bHL}] + [\mathrm{hC3bC3bCR1}] + [\mathrm{hC3biC3bH}] + [\mathrm{hC3biC3bHL}] + [\mathrm{hC3biC3bCR1}] + [\mathrm{hiC3biC3bCR1}]} \cdots \\
\frac{1}{+ [\mathrm{hiC3bC3dgCR1}] + [\mathrm{fC4bCR1}] + [\mathrm{nfC4bC4BP}] + [\mathrm{fC4bC4BP}] + [\mathrm{fC4bC4bCR1}] + [\mathrm{fC4bC4bC4BP}] + [\mathrm{fC4bC4dCR1}] + [\mathrm{fC4bC4dC4BP}] + [\mathrm{hC4bCR1}]} \cdots \\
\frac{1}{+ [\mathrm{hC4bC4BP}] + [\mathrm{hC4bC4bCR1}] + [\mathrm{hC4bC4bC4BP}] + [\mathrm{hC4bC4dCR1}] + [\mathrm{hC4bC4dC4BP}]} \cdots
\end{array}\right)$$

$$- k_{\mathrm{C4bC4BP}}^{+}[\mathrm{fC4bC4d}][\mathrm{C4BP}] + k_{\mathrm{C4bC4BP}}^{-}[\mathrm{fC4bC4dC4BP}] - k_{\mathrm{C4bC4dCR1}}^{+}[\mathrm{fC4bC4d}][\mathrm{CR1}] + k_{\mathrm{C4bC4dCR1}}^{-}[\mathrm{fC4bC4dCR1}]$$

S188

$$\frac{d[\text{fC4bC4dC4BP}]}{dt} = k^{+}_{\text{C4bC4BP}}[\text{fC4bC4d}][\text{C4BP}] - k^{-}_{\text{C4bC4BP}}[\text{fC4bC4dC4BP}]$$

$$- \left(\begin{array}{l}
\frac{k^{\text{FI}}_{\text{catC3bH}}[\text{I}][\text{fC4bC4dC4BP}]}{K^{\text{FI}}_{\text{mC3bH}} + [\text{C3(H}_2\text{O)H}] + [\text{C3(H}_2\text{O)HL}] + [\text{fC3bH}] + [\text{fC3bHL}] + [\text{fC3bCR1}] + [\text{fiC3bCR1}] + [\text{IgGC3bC3bH}] + [\text{IgGC3biC3bH}] + [\text{IgGC3bC3bHL}]} \cdots \\
\frac{1}{+[\text{IgGC3biC3bHL}] + [\text{IgGC3bC3bCR1}] + [\text{IgGC3biC3bCR1}] + [\text{IgGiC3biC3bCR1}] + [\text{IgGiC3bC3dgCR1}] + [\text{fC3bC4bH}] + [\text{fC3bC4bHL}]} \cdots \\
\frac{1}{+[\text{fC3bC4bCR1}] + [\text{fC3bC4bC4BP}] + [\text{fC3bC4dH}] + [\text{fC3bC4dHL}] + [\text{fC3bC4dCR1}] + [\text{fiC3bC4dCR1}] + [\text{fiC3bC4bCR1}] + [\text{fiC3bC4bC4BP}]} \cdots \\
\frac{1}{+[\text{fC3dgC4bCR1}] + [\text{fC3dgC4bC4BP}] + [\text{fC3bC3bH}] + [\text{fC3bC3bHL}] + [\text{fC3bC3bCR1}] + [\text{fC3biC3bH}] + [\text{fC3biC3bHL}] + [\text{fC3biC3bCR1}]} \cdots \\
\frac{1}{+[\text{fiC3biC3bCR1}] + [\text{fiC3bC3dgCR1}] + [\text{hC3bH}] + [\text{hC3bHL}] + [\text{hC3bCR1}] + [\text{hiC3bCR1}] + [\text{hC3bC4bH}] + [\text{hC3bC4bHL}] + [\text{hC3bC4bCR1}]} \cdots \\
\frac{1}{+[\text{hC3bC4bC4BP}] + [\text{hC3bC4dH}] + [\text{hC3bC4dHL}] + [\text{hC3bC4dCR1}] + [\text{hiC3bC4dCR1}] + [\text{hiC3bC4bCR1}] + [\text{hiC3bC4bC4BP}] + [\text{hC3dgC4bCR1}]} \cdots \\
\frac{1}{+[\text{hC3dgC4bC4BP}] + [\text{hC3bC3bH}] + [\text{hC3bC3bHL}] + [\text{hC3bC3bCR1}] + [\text{hC3biC3bH}] + [\text{hC3biC3bHL}] + [\text{hC3biC3bCR1}] + [\text{hiC3biC3bCR1}]} \cdots \\
\frac{1}{+[\text{hiC3bC3dgCR1}] + [\text{fC4bCR1}] + [\text{nfC4bC4BP}] + [\text{fC4bC4BP}] + [\text{fC4bC4bCR1}] + [\text{fC4bC4bC4BP}] + [\text{fC4bC4dCR1}] + [\text{fC4bC4dC4BP}] + [\text{hC4bCR1}]} \cdots \\
\frac{1}{+[\text{hC4bC4BP}] + [\text{hC4bC4bCR1}] + [\text{hC4bC4bC4BP}] + [\text{hC4bC4dCR1}] + [\text{hC4bC4dC4BP}]} \cdots
\end{array} \right)$$

S189

$$\frac{d[\text{fC4bC4dCR1}]}{dt} = k^{+}_{\text{C4bC4dCR1}}[\text{fC4bC4d}][\text{CR1}] - k^{-}_{\text{C4bC4dCR1}}[\text{fC4bC4dCR1}]$$

$$- \left(\begin{array}{l}
\frac{k^{\text{FI}}_{\text{catC3bH}}[\text{I}][\text{fC4bC4dCR1}]}{K^{\text{FI}}_{\text{mC3bH}} + [\text{C3(H}_2\text{O)H}] + [\text{C3(H}_2\text{O)HL}] + [\text{fC3bH}] + [\text{fC3bHL}] + [\text{fC3bCR1}] + [\text{fiC3bCR1}] + [\text{IgGC3bC3bH}] + [\text{IgGC3biC3bH}] + [\text{IgGC3bC3bHL}]} \cdots \\
\frac{1}{+[\text{IgGC3biC3bHL}] + [\text{IgGC3bC3bCR1}] + [\text{IgGC3biC3bCR1}] + [\text{IgGiC3biC3bCR1}] + [\text{IgGiC3bC3dgCR1}] + [\text{fC3bC4bH}] + [\text{fC3bC4bHL}]} \cdots \\
\frac{1}{+[\text{fC3bC4bCR1}] + [\text{fC3bC4bC4BP}] + [\text{fC3bC4dH}] + [\text{fC3bC4dHL}] + [\text{fC3bC4dCR1}] + [\text{fiC3bC4dCR1}] + [\text{fiC3bC4bCR1}] + [\text{fiC3bC4bC4BP}]} \cdots \\
\frac{1}{+[\text{fC3dgC4bCR1}] + [\text{fC3dgC4bC4BP}] + [\text{fC3bC3bH}] + [\text{fC3bC3bHL}] + [\text{fC3bC3bCR1}] + [\text{fC3biC3bH}] + [\text{fC3biC3bHL}] + [\text{fC3biC3bCR1}]} \cdots \\
\frac{1}{+[\text{fiC3biC3bCR1}] + [\text{fiC3bC3dgCR1}] + [\text{hC3bH}] + [\text{hC3bHL}] + [\text{hC3bCR1}] + [\text{hiC3bCR1}] + [\text{hC3bC4bH}] + [\text{hC3bC4bHL}] + [\text{hC3bC4bCR1}]} \cdots \\
\frac{1}{+[\text{hC3bC4bC4BP}] + [\text{hC3bC4dH}] + [\text{hC3bC4dHL}] + [\text{hC3bC4dCR1}] + [\text{hiC3bC4dCR1}] + [\text{hiC3bC4bCR1}] + [\text{hiC3bC4bC4BP}] + [\text{hC3dgC4bCR1}]} \cdots \\
\frac{1}{+[\text{hC3dgC4bC4BP}] + [\text{hC3bC3bH}] + [\text{hC3bC3bHL}] + [\text{hC3bC3bCR1}] + [\text{hC3biC3bH}] + [\text{hC3biC3bHL}] + [\text{hC3biC3bCR1}] + [\text{hiC3biC3bCR1}]} \cdots \\
\frac{1}{+[\text{hiC3bC3dgCR1}] + [\text{fC4bCR1}] + [\text{nfC4bC4BP}] + [\text{fC4bC4BP}] + [\text{fC4bC4bCR1}] + [\text{fC4bC4bC4BP}] + [\text{fC4bC4dCR1}] + [\text{fC4bC4dC4BP}] + [\text{hC4bCR1}]} \cdots \\
\frac{1}{+[\text{hC4bC4BP}] + [\text{hC4bC4bCR1}] + [\text{hC4bC4bC4BP}] + [\text{hC4bC4dCR1}] + [\text{hC4bC4dC4BP}]} \cdots
\end{array} \right)$$

S190

$$\frac{d[\mathrm{fC4dC4d}]}{dt}=$$

$$\left(\begin{array}{l}\frac{k_{\mathrm{catC3bH}}^{\mathrm{FI}}[\mathrm{I}][\mathrm{fC4bC4dC4BP}]}{K_{\mathrm{mC3bH}}^{\mathrm{FI}}+[\mathrm{C3(H_2O)H}]+[\mathrm{C3(H_2O)HL}]+[\mathrm{fC3bH}]+[\mathrm{fC3bHL}]+[\mathrm{fC3bCR1}]+[\mathrm{fiC3bCR1}]+[\mathrm{IgGC3bC3bH}]+[\mathrm{IgGC3biC3bH}]+[\mathrm{IgGC3bC3bHL}]}\cdots\\ \frac{1}{+[\mathrm{IgGC3biC3bHL}]+[\mathrm{IgGC3bC3bCR1}]+[\mathrm{IgGC3biC3bCR1}]+[\mathrm{IgGiC3biC3bCR1}]+[\mathrm{IgGiC3bC3dgCR1}]+[\mathrm{fC3bC4bH}]+[\mathrm{fC3bC4bHL}]}\cdots\\ \frac{1}{+[\mathrm{fC3bC4bCR1}]+[\mathrm{fC3bC4bC4BP}]+[\mathrm{fC3bC4dH}]+[\mathrm{fC3bC4dHL}]+[\mathrm{fC3bC4dCR1}]+[\mathrm{fiC3bC4dCR1}]+[\mathrm{fiC3bC4bCR1}]+[\mathrm{fiC3bC4bC4BP}]}\cdots\\ \frac{1}{+[\mathrm{fC3dgC4bCR1}]+[\mathrm{fC3dgC4bC4BP}]+[\mathrm{fC3bC3bH}]+[\mathrm{fC3bC3bHL}]+[\mathrm{fC3bC3bCR1}]+[\mathrm{fC3biC3bH}]+[\mathrm{fC3biC3bHL}]+[\mathrm{fC3biC3bCR1}]}\cdots\\ \frac{1}{+[\mathrm{fiC3biC3bCR1}]+[\mathrm{fiC3bC3dgCR1}]+[\mathrm{hC3bH}]+[\mathrm{hC3bHL}]+[\mathrm{hC3bCR1}]+[\mathrm{hiC3bCR1}]+[\mathrm{hC3bC4bH}]+[\mathrm{hC3bC4bHL}]+[\mathrm{hC3bC4bCR1}]}\cdots\\ \frac{1}{+[\mathrm{hC3bC4bC4BP}]+[\mathrm{hC3bC4dH}]+[\mathrm{hC3bC4dHL}]+[\mathrm{hC3bC4dCR1}]+[\mathrm{hiC3bC4dCR1}]+[\mathrm{hiC3bC4bCR1}]+[\mathrm{hiC3bC4bC4BP}]+[\mathrm{hC3dgC4bCR1}]}\cdots\\ \frac{1}{+[\mathrm{hC3dgC4bC4BP}]+[\mathrm{hC3bC3bH}]+[\mathrm{hC3bC3bHL}]+[\mathrm{hC3bC3bCR1}]+[\mathrm{hC3biC3bH}]+[\mathrm{hC3biC3bHL}]+[\mathrm{hC3biC3bCR1}]+[\mathrm{hiC3biC3bCR1}]}\cdots\\ \frac{1}{+[\mathrm{hiC3bC3dgCR1}]+[\mathrm{fC4bCR1}]+[\mathrm{nfC4bC4BP}]+[\mathrm{fC4bC4BP}]+[\mathrm{fC4bC4bCR1}]+[\mathrm{fC4bC4bC4BP}]+[\mathrm{fC4bC4dCR1}]+[\mathrm{fC4bC4dC4BP}]+[\mathrm{hC4bCR1}]}\cdots\\ \frac{1}{+[\mathrm{hC4bC4BP}]+[\mathrm{hC4bC4bCR1}]+[\mathrm{hC4bC4bC4BP}]+[\mathrm{hC4bC4dCR1}]+[\mathrm{hC4bC4dC4BP}]}\cdots\end{array}\right)$$

$$+\left(\begin{array}{l}\frac{k_{\mathrm{catC3bH}}^{\mathrm{FI}}[\mathrm{I}][\mathrm{fC4bC4dCR1}]}{K_{\mathrm{mC3bH}}^{\mathrm{FI}}+[\mathrm{C3(H_2O)H}]+[\mathrm{C3(H_2O)HL}]+[\mathrm{fC3bH}]+[\mathrm{fC3bHL}]+[\mathrm{fC3bCR1}]+[\mathrm{fiC3bCR1}]+[\mathrm{IgGC3bC3bH}]+[\mathrm{IgGC3biC3bH}]+[\mathrm{IgGC3bC3bHL}]}\cdots\\ \frac{1}{+[\mathrm{IgGC3biC3bHL}]+[\mathrm{IgGC3bC3bCR1}]+[\mathrm{IgGC3biC3bCR1}]+[\mathrm{IgGiC3biC3bCR1}]+[\mathrm{IgGiC3bC3dgCR1}]+[\mathrm{fC3bC4bH}]+[\mathrm{fC3bC4bHL}]}\cdots\\ \frac{1}{+[\mathrm{fC3bC4bCR1}]+[\mathrm{fC3bC4bC4BP}]+[\mathrm{fC3bC4dH}]+[\mathrm{fC3bC4dHL}]+[\mathrm{fC3bC4dCR1}]+[\mathrm{fiC3bC4dCR1}]+[\mathrm{fiC3bC4bCR1}]+[\mathrm{fiC3bC4bC4BP}]}\cdots\\ \frac{1}{+[\mathrm{fC3dgC4bCR1}]+[\mathrm{fC3dgC4bC4BP}]+[\mathrm{fC3bC3bH}]+[\mathrm{fC3bC3bHL}]+[\mathrm{fC3bC3bCR1}]+[\mathrm{fC3biC3bH}]+[\mathrm{fC3biC3bHL}]+[\mathrm{fC3biC3bCR1}]}\cdots\\ \frac{1}{+[\mathrm{fiC3biC3bCR1}]+[\mathrm{fiC3bC3dgCR1}]+[\mathrm{hC3bH}]+[\mathrm{hC3bHL}]+[\mathrm{hC3bCR1}]+[\mathrm{hiC3bCR1}]+[\mathrm{hC3bC4bH}]+[\mathrm{hC3bC4bHL}]+[\mathrm{hC3bC4bCR1}]}\cdots\\ \frac{1}{+[\mathrm{hC3bC4bC4BP}]+[\mathrm{hC3bC4dH}]+[\mathrm{hC3bC4dHL}]+[\mathrm{hC3bC4dCR1}]+[\mathrm{hiC3bC4dCR1}]+[\mathrm{hiC3bC4bCR1}]+[\mathrm{hiC3bC4bC4BP}]+[\mathrm{hC3dgC4bCR1}]}\cdots\\ \frac{1}{+[\mathrm{hC3dgC4bC4BP}]+[\mathrm{hC3bC3bH}]+[\mathrm{hC3bC3bHL}]+[\mathrm{hC3bC3bCR1}]+[\mathrm{hC3biC3bH}]+[\mathrm{hC3biC3bHL}]+[\mathrm{hC3biC3bCR1}]+[\mathrm{hiC3biC3bCR1}]}\cdots\\ \frac{1}{+[\mathrm{hiC3bC3dgCR1}]+[\mathrm{fC4bCR1}]+[\mathrm{nfC4bC4BP}]+[\mathrm{fC4bC4BP}]+[\mathrm{fC4bC4bCR1}]+[\mathrm{fC4bC4bC4BP}]+[\mathrm{fC4bC4dCR1}]+[\mathrm{fC4bC4dC4BP}]+[\mathrm{hC4bCR1}]}\cdots\\ \frac{1}{+[\mathrm{hC4bC4BP}]+[\mathrm{hC4bC4bCR1}]+[\mathrm{hC4bC4bC4BP}]+[\mathrm{hC4bC4dCR1}]+[\mathrm{hC4bC4dC4BP}]}\cdots\end{array}\right)$$

S191

$$\frac{d[\mathrm{fC3bC4bC2aC4BP}]}{dt}=k_{\mathrm{C4bC4BP}}^{+}[\mathrm{fC3bC4bC2a}][\mathrm{C4BP}]-k_{\mathrm{C4bC4BP}}^{-}[\mathrm{fC3bC4bC2aC4BP}]-k_{\mathrm{C4bC2aC4BP}_{\mathrm{decay}}}^{-}[\mathrm{fC3bC4bC2aC4BP}]$$

S192

$$\frac{d[\text{fC3bC4bC2aCR1}]}{dt} = k^{+}_{\text{C3bC4bCR1}}[\text{fC3bC4bC2a}][\text{CR1}] - k^{-}_{\text{C3bC4bCR1}}[\text{fC3bC4bC2aCR1}] - k^{-}_{\text{C4bC2aCR1}_{\text{decay}}}[\text{fC3bC4bC2aCR1}]$$

S193

$$\frac{d[\text{hC3bH}]}{dt} = k^{+}_{\text{C3bH}}[\text{hC3b}][\text{H}] - k^{-}_{\text{C3bH}}[\text{hC3bH}]$$

$$\begin{aligned} - \Bigg(& \frac{k^{\text{FI}}_{\text{catC3bH}}[\text{I}][\text{hC3bH}]}{K^{\text{FI}}_{\text{mC3bH}} + [\text{C3(H}_2\text{O)H}] + [\text{C3(H}_2\text{O)HL}] + [\text{fC3bH}] + [\text{fC3bHL}] + [\text{fC3bCR1}] + [\text{fiC3bCR1}] + [\text{IgGC3bC3bH}] + [\text{IgGC3biC3bH}] + [\text{IgGC3bC3bHL}]} \cdots \\ & \frac{1}{+[\text{IgGC3biC3bHL}] + [\text{IgGC3bC3bCR1}] + [\text{IgGC3biC3bCR1}] + [\text{IgGiC3biC3bCR1}] + [\text{IgGiC3bC3dgCR1}] + [\text{fC3bC4bH}] + [\text{fC3bC4bHL}]} \cdots \\ & \frac{1}{+[\text{fC3bC4bCR1}] + [\text{fC3bC4bC4BP}] + [\text{fC3bC4dH}] + [\text{fC3bC4dHL}] + [\text{fC3bC4dCR1}] + [\text{fiC3bC4dCR1}] + [\text{fiC3bC4bCR1}] + [\text{fiC3bC4bC4BP}]} \cdots \\ & \frac{1}{+[\text{fC3dgC4bCR1}] + [\text{fC3dgC4bC4BP}] + [\text{fC3bC3bH}] + [\text{fC3bC3bHL}] + [\text{fC3bC3bCR1}] + [\text{fC3biC3bH}] + [\text{fC3biC3bHL}] + [\text{fC3biC3bCR1}]} \cdots \\ & \frac{1}{+[\text{fiC3biC3bCR1}] + [\text{fiC3bC3dgCR1}] + [\text{hC3bH}] + [\text{hC3bHL}] + [\text{hC3bCR1}] + [\text{hiC3bCR1}] + [\text{hC3bC4bH}] + [\text{hC3bC4bHL}] + [\text{hC3bC4bCR1}]} \cdots \\ & \frac{1}{+[\text{hC3bC4bC4BP}] + [\text{hC3bC4dH}] + [\text{hC3bC4dHL}] + [\text{hC3bC4dCR1}] + [\text{hiC3bC4dCR1}] + [\text{hiC3bC4bCR1}] + [\text{hiC3bC4bC4BP}] + [\text{hC3dgC4bCR1}]} \cdots \\ & \frac{1}{+[\text{hC3dgC4bC4BP}] + [\text{hC3bC3bH}] + [\text{hC3bC3bHL}] + [\text{hC3bC3bCR1}] + [\text{hC3biC3bH}] + [\text{hC3biC3bHL}] + [\text{hC3biC3bCR1}] + [\text{hiC3biC3bCR1}]} \cdots \\ & \frac{1}{+[\text{hiC3bC3dgCR1}] + [\text{fC4bCR1}] + [\text{nfC4bC4BP}] + [\text{fC4bC4BP}] + [\text{fC4bC4bCR1}] + [\text{fC4bC4bC4BP}] + [\text{fC4bC4dCR1}] + [\text{fC4bC4dC4BP}] + [\text{hC4bCR1}]} \cdots \\ & \frac{1}{+[\text{hC4bC4BP}] + [\text{hC4bC4bCR1}] + [\text{hC4bC4bC4BP}] + [\text{hC4bC4dCR1}] + [\text{hC4bC4dC4BP}]} \cdots \Bigg) \end{aligned}$$

S194

$$\frac{d[\text{hC3bBbH}]}{dt} = k^{+}_{\text{C3bH}}[\text{hC3bBb}][\text{H}] - k^{-}_{\text{C3bH}}[\text{hC3bBbH}] - k^{-}_{\text{C3bBbH}_{\text{decay}}}[\text{hC3bBbH}]$$

S195

$$\frac{d[\text{hC3bBbDAF}]}{dt} = k^{+}_{\text{C3bBbDAF}}[\text{hC3bBb}][\text{DAF}] - k^{-}_{\text{C3bBbDAF}}[\text{hC3bBbDAF}] - k^{-}_{\text{C3bBbDAF}_{\text{decay}}}[\text{hC3bBbDAF}]$$

S196

$$\frac{d[\mathrm{hC3bHL}]}{dt} = k^{+}_{\mathrm{C3bHL}}[\mathrm{hC3b}][\mathrm{HL}] - k^{-}_{\mathrm{C3bHL}}[\mathrm{hC3bHL}]$$

$$-\left(\begin{array}{l}
\dfrac{k^{\mathrm{FI}}_{\mathrm{catC3bH}}[\mathrm{I}][\mathrm{hC3bHL}]}{K^{\mathrm{FI}}_{\mathrm{mC3bH}} + [\mathrm{C3(H_2O)H}] + [\mathrm{C3(H_2O)HL}] + [\mathrm{fC3bH}] + [\mathrm{fC3bHL}] + [\mathrm{fC3bCR1}] + [\mathrm{fiC3bCR1}] + [\mathrm{IgGC3bC3bH}] + [\mathrm{IgGC3biC3bH}] + [\mathrm{IgGC3bC3bHL}]}\ldots \\
\dfrac{1}{+[\mathrm{IgGC3biC3bHL}] + [\mathrm{IgGC3bC3bCR1}] + [\mathrm{IgGC3biC3bCR1}] + [\mathrm{IgGiC3biC3bCR1}] + [\mathrm{IgGiC3bC3dgCR1}] + [\mathrm{fC3bC4bH}] + [\mathrm{fC3bC4bHL}]}\ldots \\
\dfrac{1}{+[\mathrm{fC3bC4bCR1}] + [\mathrm{fC3bC4bC4BP}] + [\mathrm{fC3bC4dH}] + [\mathrm{fC3bC4dHL}] + [\mathrm{fC3bC4dCR1}] + [\mathrm{fiC3bC4dCR1}] + [\mathrm{fiC3bC4bCR1}] + [\mathrm{fiC3bC4bC4BP}]}\ldots \\
\dfrac{1}{+[\mathrm{fC3dgC4bCR1}] + [\mathrm{fC3dgC4bC4BP}] + [\mathrm{fC3bC3bH}] + [\mathrm{fC3bC3bHL}] + [\mathrm{fC3bC3bCR1}] + [\mathrm{fC3biC3bH}] + [\mathrm{fC3biC3bHL}] + [\mathrm{fC3biC3bCR1}]}\ldots \\
\dfrac{1}{+[\mathrm{fiC3biC3bCR1}] + [\mathrm{fiC3bC3dgCR1}] + [\mathrm{hC3bH}] + [\mathrm{hC3bHL}] + [\mathrm{hC3bCR1}] + [\mathrm{hiC3bCR1}] + [\mathrm{hC3bC4bH}] + [\mathrm{hC3bC4bHL}] + [\mathrm{hC3bC4bCR1}]}\ldots \\
\dfrac{1}{+[\mathrm{hC3bC4bC4BP}] + [\mathrm{hC3bC4dH}] + [\mathrm{hC3bC4dHL}] + [\mathrm{hC3bC4dCR1}] + [\mathrm{hiC3bC4dCR1}] + [\mathrm{hiC3bC4bCR1}] + [\mathrm{hiC3bC4bC4BP}] + [\mathrm{hC3dgC4bCR1}]}\ldots \\
\dfrac{1}{+[\mathrm{hC3dgC4bC4BP}] + [\mathrm{hC3bC3bH}] + [\mathrm{hC3bC3bHL}] + [\mathrm{hC3bC3bCR1}] + [\mathrm{hC3biC3bH}] + [\mathrm{hC3biC3bHL}] + [\mathrm{hC3biC3bCR1}] + [\mathrm{hiC3biC3bCR1}]}\ldots \\
\dfrac{1}{+[\mathrm{hiC3bC3dgCR1}] + [\mathrm{fC4bCR1}] + [\mathrm{nfC4bC4BP}] + [\mathrm{fC4bC4BP}] + [\mathrm{fC4bC4bCR1}] + [\mathrm{fC4bC4bC4BP}] + [\mathrm{fC4bC4dCR1}] + [\mathrm{fC4bC4dC4BP}] + [\mathrm{hC4bCR1}]}\ldots \\
\dfrac{1}{+[\mathrm{hC4bC4BP}] + [\mathrm{hC4bC4bCR1}] + [\mathrm{hC4bC4bC4BP}] + [\mathrm{hC4bC4dCR1}] + [\mathrm{hC4bC4dC4BP}]}\ldots
\end{array}\right)$$

S197

$$\frac{d[\mathrm{hC3bBbHL}]}{dt} = k^{+}_{\mathrm{C3bHL}}[\mathrm{hC3bBb}][\mathrm{HL}] - k^{-}_{\mathrm{C3bHL}}[\mathrm{hC3bBbHL}] - k^{-}_{\mathrm{C3bBbHL}_{\mathrm{decay}}}[\mathrm{hC3bBbHL}]$$

S198

$$\frac{d[\mathrm{hC3bCR1}]}{dt} = k^{+}_{\mathrm{C3bCR1}}[\mathrm{hC3b}][\mathrm{CR1}] - k^{-}_{\mathrm{C3bCR1}}[\mathrm{hC3bCR1}]$$

$$-\left(\begin{array}{l}
\dfrac{k^{\mathrm{FI}}_{\mathrm{catC3bH}}[\mathrm{I}][\mathrm{hC3bCR1}]}{K^{\mathrm{FI}}_{\mathrm{mC3bH}} + [\mathrm{C3(H_2O)H}] + [\mathrm{C3(H_2O)HL}] + [\mathrm{fC3bH}] + [\mathrm{fC3bHL}] + [\mathrm{fC3bCR1}] + [\mathrm{fiC3bCR1}] + [\mathrm{IgGC3bC3bH}] + [\mathrm{IgGC3biC3bH}] + [\mathrm{IgGC3bC3bHL}]}\ldots \\
\dfrac{1}{+[\mathrm{IgGC3biC3bHL}] + [\mathrm{IgGC3bC3bCR1}] + [\mathrm{IgGC3biC3bCR1}] + [\mathrm{IgGiC3biC3bCR1}] + [\mathrm{IgGiC3bC3dgCR1}] + [\mathrm{fC3bC4bH}] + [\mathrm{fC3bC4bHL}]}\ldots \\
\dfrac{1}{+[\mathrm{fC3bC4bCR1}] + [\mathrm{fC3bC4bC4BP}] + [\mathrm{fC3bC4dH}] + [\mathrm{fC3bC4dHL}] + [\mathrm{fC3bC4dCR1}] + [\mathrm{fiC3bC4dCR1}] + [\mathrm{fiC3bC4bCR1}] + [\mathrm{fiC3bC4bC4BP}]}\ldots \\
\dfrac{1}{+[\mathrm{fC3dgC4bCR1}] + [\mathrm{fC3dgC4bC4BP}] + [\mathrm{fC3bC3bH}] + [\mathrm{fC3bC3bHL}] + [\mathrm{fC3bC3bCR1}] + [\mathrm{fC3biC3bH}] + [\mathrm{fC3biC3bHL}] + [\mathrm{fC3biC3bCR1}]}\ldots \\
\dfrac{1}{+[\mathrm{fiC3biC3bCR1}] + [\mathrm{fiC3bC3dgCR1}] + [\mathrm{hC3bH}] + [\mathrm{hC3bHL}] + [\mathrm{hC3bCR1}] + [\mathrm{hiC3bCR1}] + [\mathrm{hC3bC4bH}] + [\mathrm{hC3bC4bHL}] + [\mathrm{hC3bC4bCR1}]}\ldots \\
\dfrac{1}{+[\mathrm{hC3bC4bC4BP}] + [\mathrm{hC3bC4dH}] + [\mathrm{hC3bC4dHL}] + [\mathrm{hC3bC4dCR1}] + [\mathrm{hiC3bC4dCR1}] + [\mathrm{hiC3bC4bCR1}] + [\mathrm{hiC3bC4bC4BP}] + [\mathrm{hC3dgC4bCR1}]}\ldots \\
\dfrac{1}{+[\mathrm{hC3dgC4bC4BP}] + [\mathrm{hC3bC3bH}] + [\mathrm{hC3bC3bHL}] + [\mathrm{hC3bC3bCR1}] + [\mathrm{hC3biC3bH}] + [\mathrm{hC3biC3bHL}] + [\mathrm{hC3biC3bCR1}] + [\mathrm{hiC3biC3bCR1}]}\ldots \\
\dfrac{1}{+[\mathrm{hiC3bC3dgCR1}] + [\mathrm{fC4bCR1}] + [\mathrm{nfC4bC4BP}] + [\mathrm{fC4bC4BP}] + [\mathrm{fC4bC4bCR1}] + [\mathrm{fC4bC4bC4BP}] + [\mathrm{fC4bC4dCR1}] + [\mathrm{fC4bC4dC4BP}] + [\mathrm{hC4bCR1}]}\ldots \\
\dfrac{1}{+[\mathrm{hC4bC4BP}] + [\mathrm{hC4bC4bCR1}] + [\mathrm{hC4bC4bC4BP}] + [\mathrm{hC4bC4dCR1}] + [\mathrm{hC4bC4dC4BP}]}\ldots
\end{array}\right)$$

S199

$$\frac{d[\mathrm{hC3bBbCR1}]}{dt} = k^{+}_{\mathrm{C3bCR1}}[\mathrm{hC3bBb}][\mathrm{CR1}] - k^{-}_{\mathrm{C3bCR1}}[\mathrm{hC3bBbCR1}] - k^{-}_{\mathrm{C3bBbCR1}_{\mathrm{decay}}}[\mathrm{hC3bBbCR1}]$$

S200

$$\frac{d[\mathrm{hiC3b}]}{dt} =$$

$$\left(\begin{array}{l}
\frac{k^{\mathrm{FI}}_{\mathrm{catC3bH}}[\mathrm{I}][\mathrm{hC3bH}]}{K^{\mathrm{FI}}_{\mathrm{mC3bH}} + [\mathrm{C3(H_2O)H}] + [\mathrm{C3(H_2O)HL}] + [\mathrm{fC3bH}] + [\mathrm{fC3bHL}] + [\mathrm{fC3bCR1}] + [\mathrm{fiC3bCR1}] + [\mathrm{IgGC3bC3bH}] + [\mathrm{IgGC3biC3bH}] + [\mathrm{IgGC3bC3bHL}]}\cdots \\
\frac{1}{+ [\mathrm{IgGC3biC3bHL}] + [\mathrm{IgGC3bC3bCR1}] + [\mathrm{IgGC3biC3bCR1}] + [\mathrm{IgGiC3biC3bCR1}] + [\mathrm{IgGiC3bC3dgCR1}] + [\mathrm{fC3bC4bH}] + [\mathrm{fC3bC4bHL}]}\cdots \\
\frac{1}{+ [\mathrm{fC3bC4bCR1}] + [\mathrm{fC3bC4bC4BP}] + [\mathrm{fC3bC4dH}] + [\mathrm{fC3bC4dHL}] + [\mathrm{fC3bC4dCR1}] + [\mathrm{fiC3bC4dCR1}] + [\mathrm{fiC3bC4bCR1}] + [\mathrm{fiC3bC4bC4BP}]}\cdots \\
\frac{1}{+ [\mathrm{fC3dgC4bCR1}] + [\mathrm{fC3dgC4bC4BP}] + [\mathrm{fC3bC3bH}] + [\mathrm{fC3bC3bHL}] + [\mathrm{fC3bC3bCR1}] + [\mathrm{fC3biC3bH}] + [\mathrm{fC3biC3bHL}] + [\mathrm{fC3biC3bCR1}]}\cdots \\
\frac{1}{+ [\mathrm{fiC3biC3bCR1}] + [\mathrm{fiC3bC3dgCR1}] + [\mathrm{hC3bH}] + [\mathrm{hC3bHL}] + [\mathrm{hC3bCR1}] + [\mathrm{hiC3bCR1}] + [\mathrm{hC3bC4bH}] + [\mathrm{hC3bC4bHL}] + [\mathrm{hC3bC4bCR1}]}\cdots \\
\frac{1}{+ [\mathrm{hC3bC4bC4BP}] + [\mathrm{hC3bC4dH}] + [\mathrm{hC3bC4dHL}] + [\mathrm{hC3bC4dCR1}] + [\mathrm{hiC3bC4dCR1}] + [\mathrm{hiC3bC4bCR1}] + [\mathrm{hiC3bC4bC4BP}] + [\mathrm{hC3dgC4bCR1}]}\cdots \\
\frac{1}{+ [\mathrm{hC3dgC4bC4BP}] + [\mathrm{hC3bC3bH}] + [\mathrm{hC3bC3bHL}] + [\mathrm{hC3bC3bCR1}] + [\mathrm{hC3biC3bH}] + [\mathrm{hC3biC3bHL}] + [\mathrm{hC3biC3bCR1}] + [\mathrm{hiC3biC3bCR1}]}\cdots \\
\frac{1}{+ [\mathrm{hiC3bC3dgCR1}] + [\mathrm{fC4bCR1}] + [\mathrm{nfC4bC4BP}] + [\mathrm{fC4bC4BP}] + [\mathrm{fC4bC4bCR1}] + [\mathrm{fC4bC4bC4BP}] + [\mathrm{fC4bC4dCR1}] + [\mathrm{fC4bC4dC4BP}] + [\mathrm{hC4bCR1}]}\cdots \\
\frac{1}{+ [\mathrm{hC4bC4BP}] + [\mathrm{hC4bC4bCR1}] + [\mathrm{hC4bC4bC4BP}] + [\mathrm{hC4bC4dCR1}] + [\mathrm{hC4bC4dC4BP}]}\cdots
\end{array} \right)$$

$$+ \left(\begin{array}{l}
\frac{k^{\mathrm{FI}}_{\mathrm{catC3bH}}[\mathrm{I}][\mathrm{hC3bHL}]}{K^{\mathrm{FI}}_{\mathrm{mC3bH}} + [\mathrm{C3(H_2O)H}] + [\mathrm{C3(H_2O)HL}] + [\mathrm{fC3bH}] + [\mathrm{fC3bHL}] + [\mathrm{fC3bCR1}] + [\mathrm{fiC3bCR1}] + [\mathrm{IgGC3bC3bH}] + [\mathrm{IgGC3biC3bH}] + [\mathrm{IgGC3bC3bHL}]}\cdots \\
\frac{1}{+ [\mathrm{IgGC3biC3bHL}] + [\mathrm{IgGC3bC3bCR1}] + [\mathrm{IgGC3biC3bCR1}] + [\mathrm{IgGiC3biC3bCR1}] + [\mathrm{IgGiC3bC3dgCR1}] + [\mathrm{fC3bC4bH}] + [\mathrm{fC3bC4bHL}]}\cdots \\
\frac{1}{+ [\mathrm{fC3bC4bCR1}] + [\mathrm{fC3bC4bC4BP}] + [\mathrm{fC3bC4dH}] + [\mathrm{fC3bC4dHL}] + [\mathrm{fC3bC4dCR1}] + [\mathrm{fiC3bC4dCR1}] + [\mathrm{fiC3bC4bCR1}] + [\mathrm{fiC3bC4bC4BP}]}\cdots \\
\frac{1}{+ [\mathrm{fC3dgC4bCR1}] + [\mathrm{fC3dgC4bC4BP}] + [\mathrm{fC3bC3bH}] + [\mathrm{fC3bC3bHL}] + [\mathrm{fC3bC3bCR1}] + [\mathrm{fC3biC3bH}] + [\mathrm{fC3biC3bHL}] + [\mathrm{fC3biC3bCR1}]}\cdots \\
\frac{1}{+ [\mathrm{fiC3biC3bCR1}] + [\mathrm{fiC3bC3dgCR1}] + [\mathrm{hC3bH}] + [\mathrm{hC3bHL}] + [\mathrm{hC3bCR1}] + [\mathrm{hiC3bCR1}] + [\mathrm{hC3bC4bH}] + [\mathrm{hC3bC4bHL}] + [\mathrm{hC3bC4bCR1}]}\cdots \\
\frac{1}{+ [\mathrm{hC3bC4bC4BP}] + [\mathrm{hC3bC4dH}] + [\mathrm{hC3bC4dHL}] + [\mathrm{hC3bC4dCR1}] + [\mathrm{hiC3bC4dCR1}] + [\mathrm{hiC3bC4bCR1}] + [\mathrm{hiC3bC4bC4BP}] + [\mathrm{hC3dgC4bCR1}]}\cdots \\
\frac{1}{+ [\mathrm{hC3dgC4bC4BP}] + [\mathrm{hC3bC3bH}] + [\mathrm{hC3bC3bHL}] + [\mathrm{hC3bC3bCR1}] + [\mathrm{hC3biC3bH}] + [\mathrm{hC3biC3bHL}] + [\mathrm{hC3biC3bCR1}] + [\mathrm{hiC3biC3bCR1}]}\cdots \\
\frac{1}{+ [\mathrm{hiC3bC3dgCR1}] + [\mathrm{fC4bCR1}] + [\mathrm{nfC4bC4BP}] + [\mathrm{fC4bC4BP}] + [\mathrm{fC4bC4bCR1}] + [\mathrm{fC4bC4bC4BP}] + [\mathrm{fC4bC4dCR1}] + [\mathrm{fC4bC4dC4BP}] + [\mathrm{hC4bCR1}]}\cdots \\
\frac{1}{+ [\mathrm{hC4bC4BP}] + [\mathrm{hC4bC4bCR1}] + [\mathrm{hC4bC4bC4BP}] + [\mathrm{hC4bC4dCR1}] + [\mathrm{hC4bC4dC4BP}]}\cdots
\end{array} \right)$$

$$
+\left(\begin{array}{l}
\dfrac{k^{\mathrm{FI}}_{\mathrm{catC3bH}}[\mathrm{I}][\mathrm{hC3bCR1}]}{K^{\mathrm{FI}}_{\mathrm{mC3bH}}+[\mathrm{C3(H_2O)H}]+[\mathrm{C3(H_2O)HL}]+[\mathrm{fC3bH}]+[\mathrm{fC3bHL}]+[\mathrm{fC3bCR1}]+[\mathrm{fiC3bCR1}]+[\mathrm{IgGC3bC3bH}]+[\mathrm{IgGC3biC3bH}]+[\mathrm{IgGC3bC3bHL}]}\cdots \\
\dfrac{1}{+[\mathrm{IgGC3biC3bHL}]+[\mathrm{IgGC3bC3bCR1}]+[\mathrm{IgGC3biC3bCR1}]+[\mathrm{IgGiC3biC3bCR1}]+[\mathrm{IgGiC3bC3dgCR1}]+[\mathrm{fC3bC4bH}]+[\mathrm{fC3bC4bHL}]}\cdots \\
\dfrac{1}{+[\mathrm{fC3bC4bCR1}]+[\mathrm{fC3bC4bC4BP}]+[\mathrm{fC3bC4dH}]+[\mathrm{fC3bC4dHL}]+[\mathrm{fC3bC4dCR1}]+[\mathrm{fiC3bC4dCR1}]+[\mathrm{fiC3bC4bCR1}]+[\mathrm{fiC3bC4bC4BP}]}\cdots \\
\dfrac{1}{+[\mathrm{fC3dgC4bCR1}]+[\mathrm{fC3dgC4bC4BP}]+[\mathrm{fC3bC3bH}]+[\mathrm{fC3bC3bHL}]+[\mathrm{fC3bC3bCR1}]+[\mathrm{fC3biC3bH}]+[\mathrm{fC3biC3bHL}]+[\mathrm{fC3biC3bCR1}]}\cdots \\
\dfrac{1}{+[\mathrm{fiC3biC3bCR1}]+[\mathrm{fiC3bC3dgCR1}]+[\mathrm{hC3bH}]+[\mathrm{hC3bHL}]+[\mathrm{hC3bCR1}]+[\mathrm{hiC3bCR1}]+[\mathrm{hC3bC4bH}]+[\mathrm{hC3bC4bHL}]+[\mathrm{hC3bC4bCR1}]}\cdots \\
\dfrac{1}{+[\mathrm{hC3bC4bC4BP}]+[\mathrm{hC3bC4dH}]+[\mathrm{hC3bC4dHL}]+[\mathrm{hC3bC4dCR1}]+[\mathrm{hiC3bC4dCR1}]+[\mathrm{hiC3bC4bCR1}]+[\mathrm{hiC3bC4bC4BP}]+[\mathrm{hC3dgC4bCR1}]}\cdots \\
\dfrac{1}{+[\mathrm{hC3dgC4bC4BP}]+[\mathrm{hC3bC3bH}]+[\mathrm{hC3bC3bHL}]+[\mathrm{hC3bC3bCR1}]+[\mathrm{hC3biC3bH}]+[\mathrm{hC3biC3bHL}]+[\mathrm{hC3biC3bCR1}]+[\mathrm{hiC3biC3bCR1}]}\cdots \\
\dfrac{1}{+[\mathrm{hiC3bC3dgCR1}]+[\mathrm{fC4bCR1}]+[\mathrm{nfC4bC4BP}]+[\mathrm{fC4bC4BP}]+[\mathrm{fC4bC4bCR1}]+[\mathrm{fC4bC4bC4BP}]+[\mathrm{fC4bC4dCR1}]+[\mathrm{fC4bC4dC4BP}]+[\mathrm{hC4bCR1}]}\cdots \\
\dfrac{1}{+[\mathrm{hC4bC4BP}]+[\mathrm{hC4bC4bCR1}]+[\mathrm{hC4bC4bC4BP}]+[\mathrm{hC4bC4dCR1}]+[\mathrm{hC4bC4dC4BP}]}\cdots
\end{array}\right)
$$

$$
-k^{+}_{\mathrm{iC3bCR1}}[\mathrm{hiC3b}][\mathrm{CR1}]+k^{-}_{\mathrm{iC3bCR1}}[\mathrm{hiC3bCR1}]
$$

S201

$$
\frac{d[\mathrm{hiC3bCR1}]}{dt}=k^{+}_{\mathrm{iC3bCR1}}[\mathrm{hiC3b}][\mathrm{CR1}]-k^{-}_{\mathrm{iC3bCR1}}[\mathrm{hiC3bCR1}]
$$

$$
-\left(\begin{array}{l}
\dfrac{k^{\mathrm{FI}}_{\mathrm{catC3bH}}[\mathrm{I}][\mathrm{hiC3bCR1}]}{K^{\mathrm{FI}}_{\mathrm{mC3bH}}+[\mathrm{C3(H_2O)H}]+[\mathrm{C3(H_2O)HL}]+[\mathrm{fC3bH}]+[\mathrm{fC3bHL}]+[\mathrm{fC3bCR1}]+[\mathrm{fiC3bCR1}]+[\mathrm{IgGC3bC3bH}]+[\mathrm{IgGC3biC3bH}]+[\mathrm{IgGC3bC3bHL}]}\cdots \\
\dfrac{1}{+[\mathrm{IgGC3biC3bHL}]+[\mathrm{IgGC3bC3bCR1}]+[\mathrm{IgGC3biC3bCR1}]+[\mathrm{IgGiC3biC3bCR1}]+[\mathrm{IgGiC3bC3dgCR1}]+[\mathrm{fC3bC4bH}]+[\mathrm{fC3bC4bHL}]}\cdots \\
\dfrac{1}{+[\mathrm{fC3bC4bCR1}]+[\mathrm{fC3bC4bC4BP}]+[\mathrm{fC3bC4dH}]+[\mathrm{fC3bC4dHL}]+[\mathrm{fC3bC4dCR1}]+[\mathrm{fiC3bC4dCR1}]+[\mathrm{fiC3bC4bCR1}]+[\mathrm{fiC3bC4bC4BP}]}\cdots \\
\dfrac{1}{+[\mathrm{fC3dgC4bCR1}]+[\mathrm{fC3dgC4bC4BP}]+[\mathrm{fC3bC3bH}]+[\mathrm{fC3bC3bHL}]+[\mathrm{fC3bC3bCR1}]+[\mathrm{fC3biC3bH}]+[\mathrm{fC3biC3bHL}]+[\mathrm{fC3biC3bCR1}]}\cdots \\
\dfrac{1}{+[\mathrm{fiC3biC3bCR1}]+[\mathrm{fiC3bC3dgCR1}]+[\mathrm{hC3bH}]+[\mathrm{hC3bHL}]+[\mathrm{hC3bCR1}]+[\mathrm{hiC3bCR1}]+[\mathrm{hC3bC4bH}]+[\mathrm{hC3bC4bHL}]+[\mathrm{hC3bC4bCR1}]}\cdots \\
\dfrac{1}{+[\mathrm{hC3bC4bC4BP}]+[\mathrm{hC3bC4dH}]+[\mathrm{hC3bC4dHL}]+[\mathrm{hC3bC4dCR1}]+[\mathrm{hiC3bC4dCR1}]+[\mathrm{hiC3bC4bCR1}]+[\mathrm{hiC3bC4bC4BP}]+[\mathrm{hC3dgC4bCR1}]}\cdots \\
\dfrac{1}{+[\mathrm{hC3dgC4bC4BP}]+[\mathrm{hC3bC3bH}]+[\mathrm{hC3bC3bHL}]+[\mathrm{hC3bC3bCR1}]+[\mathrm{hC3biC3bH}]+[\mathrm{hC3biC3bHL}]+[\mathrm{hC3biC3bCR1}]+[\mathrm{hiC3biC3bCR1}]}\cdots \\
\dfrac{1}{+[\mathrm{hiC3bC3dgCR1}]+[\mathrm{fC4bCR1}]+[\mathrm{nfC4bC4BP}]+[\mathrm{fC4bC4BP}]+[\mathrm{fC4bC4bCR1}]+[\mathrm{fC4bC4bC4BP}]+[\mathrm{fC4bC4dCR1}]+[\mathrm{fC4bC4dC4BP}]+[\mathrm{hC4bCR1}]}\cdots \\
\dfrac{1}{+[\mathrm{hC4bC4BP}]+[\mathrm{hC4bC4bCR1}]+[\mathrm{hC4bC4bC4BP}]+[\mathrm{hC4bC4dCR1}]+[\mathrm{hC4bC4dC4BP}]}\cdots
\end{array}\right)
$$

S202

$$\frac{d[\mathrm{hC3dg}]}{dt} =$$

$$\left(\begin{array}{l}
\frac{k_{\mathrm{catC3bH}}^{\mathrm{FI}}[\mathrm{I}][\mathrm{hiC3bCR1}]}{K_{\mathrm{mC3bH}}^{\mathrm{FI}} + [\mathrm{C3(H_2O)H}] + [\mathrm{C3(H_2O)HL}] + [\mathrm{fC3bH}] + [\mathrm{fC3bHL}] + [\mathrm{fC3bCR1}] + [\mathrm{fiC3bCR1}] + [\mathrm{IgGC3bC3bH}] + [\mathrm{IgGC3biC3bH}] + [\mathrm{IgGC3bC3bHL}]} \cdots \\
\frac{1}{+ [\mathrm{IgGC3biC3bHL}] + [\mathrm{IgGC3bC3bCR1}] + [\mathrm{IgGC3biC3bCR1}] + [\mathrm{IgGiC3biC3bCR1}] + [\mathrm{IgGiC3bC3dgCR1}] + [\mathrm{fC3bC4bH}] + [\mathrm{fC3bC4bHL}]} \cdots \\
\frac{1}{+ [\mathrm{fC3bC4bCR1}] + [\mathrm{fC3bC4bC4BP}] + [\mathrm{fC3bC4dH}] + [\mathrm{fC3bC4dHL}] + [\mathrm{fC3bC4dCR1}] + [\mathrm{fiC3bC4dCR1}] + [\mathrm{fiC3bC4bCR1}] + [\mathrm{fiC3bC4bC4BP}]} \cdots \\
\frac{1}{+ [\mathrm{fC3dgC4bCR1}] + [\mathrm{fC3dgC4bC4BP}] + [\mathrm{fC3bC3bH}] + [\mathrm{fC3bC3bHL}] + [\mathrm{fC3bC3bCR1}] + [\mathrm{fC3biC3bH}] + [\mathrm{fC3biC3bHL}] + [\mathrm{fC3biC3bCR1}]} \cdots \\
\frac{1}{+ [\mathrm{fiC3biC3bCR1}] + [\mathrm{fiC3bC3dgCR1}] + [\mathrm{hC3bH}] + [\mathrm{hC3bHL}] + [\mathrm{hC3bCR1}] + [\mathrm{hiC3bCR1}] + [\mathrm{hC3bC4bH}] + [\mathrm{hC3bC4bHL}] + [\mathrm{hC3bC4bCR1}]} \cdots \\
\frac{1}{+ [\mathrm{hC3bC4bC4BP}] + [\mathrm{hC3bC4dH}] + [\mathrm{hC3bC4dHL}] + [\mathrm{hC3bC4dCR1}] + [\mathrm{hiC3bC4dCR1}] + [\mathrm{hiC3bC4bCR1}] + [\mathrm{hiC3bC4bC4BP}] + [\mathrm{hC3dgC4bCR1}]} \cdots \\
\frac{1}{+ [\mathrm{hC3dgC4bC4BP}] + [\mathrm{hC3bC3bH}] + [\mathrm{hC3bC3bHL}] + [\mathrm{hC3bC3bCR1}] + [\mathrm{hC3biC3bH}] + [\mathrm{hC3biC3bHL}] + [\mathrm{hC3biC3bCR1}] + [\mathrm{hiC3biC3bCR1}]} \cdots \\
\frac{1}{+ [\mathrm{hiC3bC3dgCR1}] + [\mathrm{fC4bCR1}] + [\mathrm{nfC4bC4BP}] + [\mathrm{fC4bC4BP}] + [\mathrm{fC4bC4bCR1}] + [\mathrm{fC4bC4bC4BP}] + [\mathrm{fC4bC4dCR1}] + [\mathrm{fC4bC4dC4BP}] + [\mathrm{hC4bCR1}]} \cdots \\
\frac{1}{+ [\mathrm{hC4bC4BP}] + [\mathrm{hC4bC4bCR1}] + [\mathrm{hC4bC4bC4BP}] + [\mathrm{hC4bC4dCR1}] + [\mathrm{hC4bC4dC4BP}]} \cdots
\end{array} \right)$$

S203

$$\frac{d[\mathrm{hC3bC4bH}]}{dt} = k_{\mathrm{C3bH}}^{+}[\mathrm{hC3bC4b}][\mathrm{H}] - k_{\mathrm{C3bH}}^{-}[\mathrm{hC3bC4bH}]$$

$$- \left(\begin{array}{l}
\frac{k_{\mathrm{catC3bH}}^{\mathrm{FI}}[\mathrm{I}][\mathrm{hC3bC4bH}]}{K_{\mathrm{mC3bH}}^{\mathrm{FI}} + [\mathrm{C3(H_2O)H}] + [\mathrm{C3(H_2O)HL}] + [\mathrm{fC3bH}] + [\mathrm{fC3bHL}] + [\mathrm{fC3bCR1}] + [\mathrm{fiC3bCR1}] + [\mathrm{IgGC3bC3bH}] + [\mathrm{IgGC3biC3bH}] + [\mathrm{IgGC3bC3bHL}]} \cdots \\
\frac{1}{+ [\mathrm{IgGC3biC3bHL}] + [\mathrm{IgGC3bC3bCR1}] + [\mathrm{IgGC3biC3bCR1}] + [\mathrm{IgGiC3biC3bCR1}] + [\mathrm{IgGiC3bC3dgCR1}] + [\mathrm{fC3bC4bH}] + [\mathrm{fC3bC4bHL}]} \cdots \\
\frac{1}{+ [\mathrm{fC3bC4bCR1}] + [\mathrm{fC3bC4bC4BP}] + [\mathrm{fC3bC4dH}] + [\mathrm{fC3bC4dHL}] + [\mathrm{fC3bC4dCR1}] + [\mathrm{fiC3bC4dCR1}] + [\mathrm{fiC3bC4bCR1}] + [\mathrm{fiC3bC4bC4BP}]} \cdots \\
\frac{1}{+ [\mathrm{fC3dgC4bCR1}] + [\mathrm{fC3dgC4bC4BP}] + [\mathrm{fC3bC3bH}] + [\mathrm{fC3bC3bHL}] + [\mathrm{fC3bC3bCR1}] + [\mathrm{fC3biC3bH}] + [\mathrm{fC3biC3bHL}] + [\mathrm{fC3biC3bCR1}]} \cdots \\
\frac{1}{+ [\mathrm{fiC3biC3bCR1}] + [\mathrm{fiC3bC3dgCR1}] + [\mathrm{hC3bH}] + [\mathrm{hC3bHL}] + [\mathrm{hC3bCR1}] + [\mathrm{hiC3bCR1}] + [\mathrm{hC3bC4bH}] + [\mathrm{hC3bC4bHL}] + [\mathrm{hC3bC4bCR1}]} \cdots \\
\frac{1}{+ [\mathrm{hC3bC4bC4BP}] + [\mathrm{hC3bC4dH}] + [\mathrm{hC3bC4dHL}] + [\mathrm{hC3bC4dCR1}] + [\mathrm{hiC3bC4dCR1}] + [\mathrm{hiC3bC4bCR1}] + [\mathrm{hiC3bC4bC4BP}] + [\mathrm{hC3dgC4bCR1}]} \cdots \\
\frac{1}{+ [\mathrm{hC3dgC4bC4BP}] + [\mathrm{hC3bC3bH}] + [\mathrm{hC3bC3bHL}] + [\mathrm{hC3bC3bCR1}] + [\mathrm{hC3biC3bH}] + [\mathrm{hC3biC3bHL}] + [\mathrm{hC3biC3bCR1}] + [\mathrm{hiC3biC3bCR1}]} \cdots \\
\frac{1}{+ [\mathrm{hiC3bC3dgCR1}] + [\mathrm{fC4bCR1}] + [\mathrm{nfC4bC4BP}] + [\mathrm{fC4bC4BP}] + [\mathrm{fC4bC4bCR1}] + [\mathrm{fC4bC4bC4BP}] + [\mathrm{fC4bC4dCR1}] + [\mathrm{fC4bC4dC4BP}] + [\mathrm{hC4bCR1}]} \cdots \\
\frac{1}{+ [\mathrm{hC4bC4BP}] + [\mathrm{hC4bC4bCR1}] + [\mathrm{hC4bC4bC4BP}] + [\mathrm{hC4bC4dCR1}] + [\mathrm{hC4bC4dC4BP}]} \cdots
\end{array} \right)$$

S204

$$\frac{d[\mathrm{hC3bC4bHL}]}{dt} = k^{+}_{\mathrm{C3bHL}}[\mathrm{hC3bC4b}][\mathrm{HL}] - k^{-}_{\mathrm{C3bHL}}[\mathrm{hC3bC4bHL}]$$

$$- \left(\frac{k^{\mathrm{FI}}_{\mathrm{catC3bH}}[\mathrm{I}][\mathrm{hC3bC4bHL}]}{K^{\mathrm{FI}}_{\mathrm{mC3bH}} + [\mathrm{C3(H_2O)H}] + [\mathrm{C3(H_2O)HL}] + [\mathrm{fC3bH}] + [\mathrm{fC3bHL}] + [\mathrm{fC3bCR1}] + [\mathrm{fiC3bCR1}] + [\mathrm{IgGC3bC3bH}] + [\mathrm{IgGC3biC3bH}] + [\mathrm{IgGC3bC3bHL}]} \cdots \right.$$
$$\frac{1}{+ [\mathrm{IgGC3biC3bHL}] + [\mathrm{IgGC3bC3bCR1}] + [\mathrm{IgGC3biC3bCR1}] + [\mathrm{IgGiC3biC3bCR1}] + [\mathrm{IgGiC3bC3dgCR1}] + [\mathrm{fC3bC4bH}] + [\mathrm{fC3bC4bHL}]} \cdots$$
$$\frac{1}{+ [\mathrm{fC3bC4bCR1}] + [\mathrm{fC3bC4bC4BP}] + [\mathrm{fC3bC4dH}] + [\mathrm{fC3bC4dHL}] + [\mathrm{fC3bC4dCR1}] + [\mathrm{fiC3bC4dCR1}] + [\mathrm{fiC3bC4bCR1}] + [\mathrm{fiC3bC4bC4BP}]} \cdots$$
$$\frac{1}{+ [\mathrm{fC3dgC4bCR1}] + [\mathrm{fC3dgC4bC4BP}] + [\mathrm{fC3bC3bH}] + [\mathrm{fC3bC3bHL}] + [\mathrm{fC3bC3bCR1}] + [\mathrm{fC3biC3bH}] + [\mathrm{fC3biC3bHL}] + [\mathrm{fC3biC3bCR1}]} \cdots$$
$$\frac{1}{+ [\mathrm{fiC3biC3bCR1}] + [\mathrm{fiC3bC3dgCR1}] + [\mathrm{hC3bH}] + [\mathrm{hC3bHL}] + [\mathrm{hC3bCR1}] + [\mathrm{hiC3bCR1}] + [\mathrm{hC3bC4bH}] + [\mathrm{hC3bC4bHL}] + [\mathrm{hC3bC4bCR1}]} \cdots$$
$$\frac{1}{+ [\mathrm{hC3bC4bC4BP}] + [\mathrm{hC3bC4dH}] + [\mathrm{hC3bC4dHL}] + [\mathrm{hC3bC4dCR1}] + [\mathrm{hiC3bC4dCR1}] + [\mathrm{hiC3bC4bCR1}] + [\mathrm{hiC3bC4bC4BP}] + [\mathrm{hC3dgC4bCR1}]} \cdots$$
$$\frac{1}{+ [\mathrm{hC3dgC4bC4BP}] + [\mathrm{hC3bC3bH}] + [\mathrm{hC3bC3bHL}] + [\mathrm{hC3bC3bCR1}] + [\mathrm{hC3biC3bH}] + [\mathrm{hC3biC3bHL}] + [\mathrm{hC3biC3bCR1}] + [\mathrm{hiC3biC3bCR1}]} \cdots$$
$$\frac{1}{+ [\mathrm{hiC3bC3dgCR1}] + [\mathrm{fC4bCR1}] + [\mathrm{nfC4bC4BP}] + [\mathrm{fC4bC4BP}] + [\mathrm{fC4bC4bCR1}] + [\mathrm{fC4bC4bC4BP}] + [\mathrm{fC4bC4dCR1}] + [\mathrm{fC4bC4dC4BP}] + [\mathrm{hC4bCR1}]} \cdots$$
$$\left. \frac{1}{+ [\mathrm{hC4bC4BP}] + [\mathrm{hC4bC4bCR1}] + [\mathrm{hC4bC4bC4BP}] + [\mathrm{hC4bC4dCR1}] + [\mathrm{hC4bC4dC4BP}]} \cdots \right)$$

S205

$$\frac{d[\mathrm{hC3bC4bCR1}]}{dt} = k^{+}_{\mathrm{C3bC4bCR1}}[\mathrm{hC3bC4b}][\mathrm{CR1}] - k^{-}_{\mathrm{C3bC4bCR1}}[\mathrm{hC3bC4bCR1}]$$

$$- \left(\frac{k^{\mathrm{FI}}_{\mathrm{catC3bH}}[\mathrm{I}][\mathrm{hC3bC4bCR1}]}{K^{\mathrm{FI}}_{\mathrm{mC3bH}} + [\mathrm{C3(H_2O)H}] + [\mathrm{C3(H_2O)HL}] + [\mathrm{fC3bH}] + [\mathrm{fC3bHL}] + [\mathrm{fC3bCR1}] + [\mathrm{fiC3bCR1}] + [\mathrm{IgGC3bC3bH}] + [\mathrm{IgGC3biC3bH}] + [\mathrm{IgGC3bC3bHL}]} \cdots \right.$$
$$\frac{1}{+ [\mathrm{IgGC3biC3bHL}] + [\mathrm{IgGC3bC3bCR1}] + [\mathrm{IgGC3biC3bCR1}] + [\mathrm{IgGiC3biC3bCR1}] + [\mathrm{IgGiC3bC3dgCR1}] + [\mathrm{fC3bC4bH}] + [\mathrm{fC3bC4bHL}]} \cdots$$
$$\frac{1}{+ [\mathrm{fC3bC4bCR1}] + [\mathrm{fC3bC4bC4BP}] + [\mathrm{fC3bC4dH}] + [\mathrm{fC3bC4dHL}] + [\mathrm{fC3bC4dCR1}] + [\mathrm{fiC3bC4dCR1}] + [\mathrm{fiC3bC4bCR1}] + [\mathrm{fiC3bC4bC4BP}]} \cdots$$
$$\frac{1}{+ [\mathrm{fC3dgC4bCR1}] + [\mathrm{fC3dgC4bC4BP}] + [\mathrm{fC3bC3bH}] + [\mathrm{fC3bC3bHL}] + [\mathrm{fC3bC3bCR1}] + [\mathrm{fC3biC3bH}] + [\mathrm{fC3biC3bHL}] + [\mathrm{fC3biC3bCR1}]} \cdots$$
$$\frac{1}{+ [\mathrm{fiC3biC3bCR1}] + [\mathrm{fiC3bC3dgCR1}] + [\mathrm{hC3bH}] + [\mathrm{hC3bHL}] + [\mathrm{hC3bCR1}] + [\mathrm{hiC3bCR1}] + [\mathrm{hC3bC4bH}] + [\mathrm{hC3bC4bHL}] + [\mathrm{hC3bC4bCR1}]} \cdots$$
$$\frac{1}{+ [\mathrm{hC3bC4bC4BP}] + [\mathrm{hC3bC4dH}] + [\mathrm{hC3bC4dHL}] + [\mathrm{hC3bC4dCR1}] + [\mathrm{hiC3bC4dCR1}] + [\mathrm{hiC3bC4bCR1}] + [\mathrm{hiC3bC4bC4BP}] + [\mathrm{hC3dgC4bCR1}]} \cdots$$
$$\frac{1}{+ [\mathrm{hC3dgC4bC4BP}] + [\mathrm{hC3bC3bH}] + [\mathrm{hC3bC3bHL}] + [\mathrm{hC3bC3bCR1}] + [\mathrm{hC3biC3bH}] + [\mathrm{hC3biC3bHL}] + [\mathrm{hC3biC3bCR1}] + [\mathrm{hiC3biC3bCR1}]} \cdots$$
$$\frac{1}{+ [\mathrm{hiC3bC3dgCR1}] + [\mathrm{fC4bCR1}] + [\mathrm{nfC4bC4BP}] + [\mathrm{fC4bC4BP}] + [\mathrm{fC4bC4bCR1}] + [\mathrm{fC4bC4bC4BP}] + [\mathrm{fC4bC4dCR1}] + [\mathrm{fC4bC4dC4BP}] + [\mathrm{hC4bCR1}]} \cdots$$
$$\left. \frac{1}{+ [\mathrm{hC4bC4BP}] + [\mathrm{hC4bC4bCR1}] + [\mathrm{hC4bC4bC4BP}] + [\mathrm{hC4bC4dCR1}] + [\mathrm{hC4bC4dC4BP}]} \cdots \right)$$

S206

$$\frac{d[\mathrm{hC3bC4bC4BP}]}{dt} = k^{+}_{\mathrm{C4bC4BP}}[\mathrm{hC3bC4b}][\mathrm{C4BP}] - k^{-}_{\mathrm{C4bC4BP}}[\mathrm{hC3bC4bC4BP}]$$

$$-\left(\frac{k^{\mathrm{FI}}_{\mathrm{catC3bH}}[\mathrm{I}][\mathrm{hC3bC4bC4BP}]}{K^{\mathrm{FI}}_{\mathrm{mC3bH}} + [\mathrm{C3(H_2O)H}] + [\mathrm{C3(H_2O)HL}] + [\mathrm{fC3bH}] + [\mathrm{fC3bHL}] + [\mathrm{fC3bCR1}] + [\mathrm{fiC3bCR1}] + [\mathrm{IgGC3bC3bH}] + [\mathrm{IgGC3biC3bH}] + [\mathrm{IgGC3bC3bHL}]}\cdots\right.$$

$$\frac{1}{+[\mathrm{IgGC3biC3bHL}] + [\mathrm{IgGC3bC3bCR1}] + [\mathrm{IgGC3biC3bCR1}] + [\mathrm{IgGiC3biC3bCR1}] + [\mathrm{IgGiC3bC3dgCR1}] + [\mathrm{fC3bC4bH}] + [\mathrm{fC3bC4bHL}]}\cdots$$

$$\frac{1}{+[\mathrm{fC3bC4bCR1}] + [\mathrm{fC3bC4bC4BP}] + [\mathrm{fC3bC4dH}] + [\mathrm{fC3bC4dHL}] + [\mathrm{fC3bC4dCR1}] + [\mathrm{fiC3bC4dCR1}] + [\mathrm{fiC3bC4bCR1}] + [\mathrm{fiC3bC4bC4BP}]}\cdots$$

$$\frac{1}{+[\mathrm{fC3dgC4bCR1}] + [\mathrm{fC3dgC4bC4BP}] + [\mathrm{fC3bC3bH}] + [\mathrm{fC3bC3bHL}] + [\mathrm{fC3bC3bCR1}] + [\mathrm{fC3biC3bH}] + [\mathrm{fC3biC3bHL}] + [\mathrm{fC3biC3bCR1}]}\cdots$$

$$\frac{1}{+[\mathrm{fiC3biC3bCR1}] + [\mathrm{fiC3bC3dgCR1}] + [\mathrm{hC3bH}] + [\mathrm{hC3bHL}] + [\mathrm{hC3bCR1}] + [\mathrm{hiC3bCR1}] + [\mathrm{hC3bC4bH}] + [\mathrm{hC3bC4bHL}] + [\mathrm{hC3bC4bCR1}]}\cdots$$

$$\frac{1}{+[\mathrm{hC3bC4bC4BP}] + [\mathrm{hC3bC4dH}] + [\mathrm{hC3bC4dHL}] + [\mathrm{hC3bC4dCR1}] + [\mathrm{hiC3bC4dCR1}] + [\mathrm{hiC3bC4bCR1}] + [\mathrm{hiC3bC4bC4BP}] + [\mathrm{hC3dgC4bCR1}]}\cdots$$

$$\frac{1}{+[\mathrm{hC3dgC4bC4BP}] + [\mathrm{hC3bC3bH}] + [\mathrm{hC3bC3bHL}] + [\mathrm{hC3bC3bCR1}] + [\mathrm{hC3biC3bH}] + [\mathrm{hC3biC3bHL}] + [\mathrm{hC3biC3bCR1}] + [\mathrm{hiC3biC3bCR1}]}\cdots$$

$$\frac{1}{+[\mathrm{hiC3bC3dgCR1}] + [\mathrm{fC4bCR1}] + [\mathrm{nfC4bC4BP}] + [\mathrm{fC4bC4BP}] + [\mathrm{fC4bC4bCR1}] + [\mathrm{fC4bC4bC4BP}] + [\mathrm{fC4bC4dCR1}] + [\mathrm{fC4bC4dC4BP}] + [\mathrm{hC4bCR1}]}\cdots$$

$$\left.\frac{1}{+[\mathrm{hC4bC4BP}] + [\mathrm{hC4bC4bCR1}] + [\mathrm{hC4bC4bC4BP}] + [\mathrm{hC4bC4dCR1}] + [\mathrm{hC4bC4dC4BP}]}\cdots\right)$$

S207

$$\frac{d[\mathrm{hiC3bC4b}]}{dt} =$$

$$\left(\frac{k^{\mathrm{FI}}_{\mathrm{catC3bH}}[\mathrm{I}][\mathrm{hC3bC4bH}]}{K^{\mathrm{FI}}_{\mathrm{mC3bH}} + [\mathrm{C3(H_2O)H}] + [\mathrm{C3(H_2O)HL}] + [\mathrm{fC3bH}] + [\mathrm{fC3bHL}] + [\mathrm{fC3bCR1}] + [\mathrm{fiC3bCR1}] + [\mathrm{IgGC3bC3bH}] + [\mathrm{IgGC3biC3bH}] + [\mathrm{IgGC3bC3bHL}]}\cdots\right.$$

$$\frac{1}{+[\mathrm{IgGC3biC3bHL}] + [\mathrm{IgGC3bC3bCR1}] + [\mathrm{IgGC3biC3bCR1}] + [\mathrm{IgGiC3biC3bCR1}] + [\mathrm{IgGiC3bC3dgCR1}] + [\mathrm{fC3bC4bH}] + [\mathrm{fC3bC4bHL}]}\cdots$$

$$\frac{1}{+[\mathrm{fC3bC4bCR1}] + [\mathrm{fC3bC4bC4BP}] + [\mathrm{fC3bC4dH}] + [\mathrm{fC3bC4dHL}] + [\mathrm{fC3bC4dCR1}] + [\mathrm{fiC3bC4dCR1}] + [\mathrm{fiC3bC4bCR1}] + [\mathrm{fiC3bC4bC4BP}]}\cdots$$

$$\frac{1}{+[\mathrm{fC3dgC4bCR1}] + [\mathrm{fC3dgC4bC4BP}] + [\mathrm{fC3bC3bH}] + [\mathrm{fC3bC3bHL}] + [\mathrm{fC3bC3bCR1}] + [\mathrm{fC3biC3bH}] + [\mathrm{fC3biC3bHL}] + [\mathrm{fC3biC3bCR1}]}\cdots$$

$$\frac{1}{+[\mathrm{fiC3biC3bCR1}] + [\mathrm{fiC3bC3dgCR1}] + [\mathrm{hC3bH}] + [\mathrm{hC3bHL}] + [\mathrm{hC3bCR1}] + [\mathrm{hiC3bCR1}] + [\mathrm{hC3bC4bH}] + [\mathrm{hC3bC4bHL}] + [\mathrm{hC3bC4bCR1}]}\cdots$$

$$\frac{1}{+[\mathrm{hC3bC4bC4BP}] + [\mathrm{hC3bC4dH}] + [\mathrm{hC3bC4dHL}] + [\mathrm{hC3bC4dCR1}] + [\mathrm{hiC3bC4dCR1}] + [\mathrm{hiC3bC4bCR1}] + [\mathrm{hiC3bC4bC4BP}] + [\mathrm{hC3dgC4bCR1}]}\cdots$$

$$\frac{1}{+[\mathrm{hC3dgC4bC4BP}] + [\mathrm{hC3bC3bH}] + [\mathrm{hC3bC3bHL}] + [\mathrm{hC3bC3bCR1}] + [\mathrm{hC3biC3bH}] + [\mathrm{hC3biC3bHL}] + [\mathrm{hC3biC3bCR1}] + [\mathrm{hiC3biC3bCR1}]}\cdots$$

$$\frac{1}{+[\mathrm{hiC3bC3dgCR1}] + [\mathrm{fC4bCR1}] + [\mathrm{nfC4bC4BP}] + [\mathrm{fC4bC4BP}] + [\mathrm{fC4bC4bCR1}] + [\mathrm{fC4bC4bC4BP}] + [\mathrm{fC4bC4dCR1}] + [\mathrm{fC4bC4dC4BP}] + [\mathrm{hC4bCR1}]}\cdots$$

$$\left.\frac{1}{+[\mathrm{hC4bC4BP}] + [\mathrm{hC4bC4bCR1}] + [\mathrm{hC4bC4bC4BP}] + [\mathrm{hC4bC4dCR1}] + [\mathrm{hC4bC4dC4BP}]}\cdots\right)$$

$$+\left(\frac{k_{\mathrm{catC3bH}}^{\mathrm{FI}}[\mathrm{I}][\mathrm{hC3bC4bHL}]}{K_{\mathrm{mC3bH}}^{\mathrm{FI}}+[\mathrm{C3(H_2O)H}]+[\mathrm{C3(H_2O)HL}]+[\mathrm{fC3bH}]+[\mathrm{fC3bHL}]+[\mathrm{fC3bCR1}]+[\mathrm{fiC3bCR1}]+[\mathrm{IgGC3bC3bH}]+[\mathrm{IgGC3biC3bH}]+[\mathrm{IgGC3bC3bHL}]}\cdots\right.$$

$$\frac{1}{+[\mathrm{IgGC3biC3bHL}]+[\mathrm{IgGC3bC3bCR1}]+[\mathrm{IgGC3biC3bCR1}]+[\mathrm{IgGiC3biC3bCR1}]+[\mathrm{IgGiC3bC3dgCR1}]+[\mathrm{fC3bC4bH}]+[\mathrm{fC3bC4bHL}]}\cdots$$

$$\frac{1}{+[\mathrm{fC3bC4bCR1}]+[\mathrm{fC3bC4bC4BP}]+[\mathrm{fC3bC4dH}]+[\mathrm{fC3bC4dHL}]+[\mathrm{fC3bC4dCR1}]+[\mathrm{fiC3bC4dCR1}]+[\mathrm{fiC3bC4bCR1}]+[\mathrm{fiC3bC4bC4BP}]}\cdots$$

$$\frac{1}{+[\mathrm{fC3dgC4bCR1}]+[\mathrm{fC3dgC4bC4BP}]+[\mathrm{fC3bC3bH}]+[\mathrm{fC3bC3bHL}]+[\mathrm{fC3bC3bCR1}]+[\mathrm{fC3biC3bH}]+[\mathrm{fC3biC3bHL}]+[\mathrm{fC3biC3bCR1}]}\cdots$$

$$\frac{1}{+[\mathrm{fiC3biC3bCR1}]+[\mathrm{fiC3bC3dgCR1}]+[\mathrm{hC3bH}]+[\mathrm{hC3bHL}]+[\mathrm{hC3bCR1}]+[\mathrm{hiC3bCR1}]+[\mathrm{hC3bC4bH}]+[\mathrm{hC3bC4bHL}]+[\mathrm{hC3bC4bCR1}]}\cdots$$

$$\frac{1}{+[\mathrm{hC3bC4bC4BP}]+[\mathrm{hC3bC4dH}]+[\mathrm{hC3bC4dHL}]+[\mathrm{hC3bC4dCR1}]+[\mathrm{hiC3bC4dCR1}]+[\mathrm{hiC3bC4bCR1}]+[\mathrm{hiC3bC4bC4BP}]+[\mathrm{hC3dgC4bCR1}]}\cdots$$

$$\frac{1}{+[\mathrm{hC3dgC4bC4BP}]+[\mathrm{hC3bC3bH}]+[\mathrm{hC3bC3bHL}]+[\mathrm{hC3bC3bCR1}]+[\mathrm{hC3biC3bH}]+[\mathrm{hC3biC3bHL}]+[\mathrm{hC3biC3bCR1}]+[\mathrm{hiC3biC3bCR1}]}\cdots$$

$$\frac{1}{+[\mathrm{hiC3bC3dgCR1}]+[\mathrm{fC4bCR1}]+[\mathrm{nfC4bC4BP}]+[\mathrm{fC4bC4BP}]+[\mathrm{fC4bC4bCR1}]+[\mathrm{fC4bC4bC4BP}]+[\mathrm{fC4bC4dCR1}]+[\mathrm{fC4bC4dC4BP}]+[\mathrm{hC4bCR1}]}\cdots$$

$$\left.\frac{1}{+[\mathrm{hC4bC4BP}]+[\mathrm{hC4bC4bCR1}]+[\mathrm{hC4bC4bC4BP}]+[\mathrm{hC4bC4dCR1}]+[\mathrm{hC4bC4dC4BP}]}\cdots\right)$$

$$+\left(\frac{k_{\mathrm{catC3bH}}^{\mathrm{FI}}[\mathrm{I}][\mathrm{hC3bC4bCR1}]}{K_{\mathrm{mC3bH}}^{\mathrm{FI}}+[\mathrm{C3(H_2O)H}]+[\mathrm{C3(H_2O)HL}]+[\mathrm{fC3bH}]+[\mathrm{fC3bHL}]+[\mathrm{fC3bCR1}]+[\mathrm{fiC3bCR1}]+[\mathrm{IgGC3bC3bH}]+[\mathrm{IgGC3biC3bH}]+[\mathrm{IgGC3bC3bHL}]}\cdots\right.$$

$$\frac{1}{+[\mathrm{IgGC3biC3bHL}]+[\mathrm{IgGC3bC3bCR1}]+[\mathrm{IgGC3biC3bCR1}]+[\mathrm{IgGiC3biC3bCR1}]+[\mathrm{IgGiC3bC3dgCR1}]+[\mathrm{fC3bC4bH}]+[\mathrm{fC3bC4bHL}]}\cdots$$

$$\frac{1}{+[\mathrm{fC3bC4bCR1}]+[\mathrm{fC3bC4bC4BP}]+[\mathrm{fC3bC4dH}]+[\mathrm{fC3bC4dHL}]+[\mathrm{fC3bC4dCR1}]+[\mathrm{fiC3bC4dCR1}]+[\mathrm{fiC3bC4bCR1}]+[\mathrm{fiC3bC4bC4BP}]}\cdots$$

$$\frac{1}{+[\mathrm{fC3dgC4bCR1}]+[\mathrm{fC3dgC4bC4BP}]+[\mathrm{fC3bC3bH}]+[\mathrm{fC3bC3bHL}]+[\mathrm{fC3bC3bCR1}]+[\mathrm{fC3biC3bH}]+[\mathrm{fC3biC3bHL}]+[\mathrm{fC3biC3bCR1}]}\cdots$$

$$\frac{1}{+[\mathrm{fiC3biC3bCR1}]+[\mathrm{fiC3bC3dgCR1}]+[\mathrm{hC3bH}]+[\mathrm{hC3bHL}]+[\mathrm{hC3bCR1}]+[\mathrm{hiC3bCR1}]+[\mathrm{hC3bC4bH}]+[\mathrm{hC3bC4bHL}]+[\mathrm{hC3bC4bCR1}]}\cdots$$

$$\frac{1}{+[\mathrm{hC3bC4bC4BP}]+[\mathrm{hC3bC4dH}]+[\mathrm{hC3bC4dHL}]+[\mathrm{hC3bC4dCR1}]+[\mathrm{hiC3bC4dCR1}]+[\mathrm{hiC3bC4bCR1}]+[\mathrm{hiC3bC4bC4BP}]+[\mathrm{hC3dgC4bCR1}]}\cdots$$

$$\frac{1}{+[\mathrm{hC3dgC4bC4BP}]+[\mathrm{hC3bC3bH}]+[\mathrm{hC3bC3bHL}]+[\mathrm{hC3bC3bCR1}]+[\mathrm{hC3biC3bH}]+[\mathrm{hC3biC3bHL}]+[\mathrm{hC3biC3bCR1}]+[\mathrm{hiC3biC3bCR1}]}\cdots$$

$$\frac{1}{+[\mathrm{hiC3bC3dgCR1}]+[\mathrm{fC4bCR1}]+[\mathrm{nfC4bC4BP}]+[\mathrm{fC4bC4BP}]+[\mathrm{fC4bC4bCR1}]+[\mathrm{fC4bC4bC4BP}]+[\mathrm{fC4bC4dCR1}]+[\mathrm{fC4bC4dC4BP}]+[\mathrm{hC4bCR1}]}\cdots$$

$$\left.\frac{1}{+[\mathrm{hC4bC4BP}]+[\mathrm{hC4bC4bCR1}]+[\mathrm{hC4bC4bC4BP}]+[\mathrm{hC4bC4dCR1}]+[\mathrm{hC4bC4dC4BP}]}\cdots\right)$$

$$-k_{\mathrm{iC3bC4bCR1}}^{+}[\mathrm{hiC3bC4b}][\mathrm{CR1}]+k_{\mathrm{iC3bC4bCR1}}^{-}[\mathrm{hiC3bC4bCR1}]-k_{\mathrm{C4bC4BP}}^{+}[\mathrm{hiC3bC4b}][\mathrm{C4BP}]+k_{\mathrm{C4bC4BP}}^{-}[\mathrm{hiC3bC4bC4BP}]$$

S208

$$\frac{d[\text{hiC3bC4bCR1}]}{dt} = k^{+}_{\text{iC3bC4bCR1}}[\text{hiC3bC4b}][\text{CR1}] - k^{-}_{\text{iC3bC4bCR1}}[\text{hiC3bC4bCR1}]$$

$$-\left(\begin{array}{l}
\dfrac{k^{\text{FI}}_{\text{catC3bH}}[\text{I}][\text{hiC3bC4bCR1}]}{K^{\text{FI}}_{\text{mC3bH}}+[\text{C3(H}_2\text{O)H}]+[\text{C3(H}_2\text{O)HL}]+[\text{fC3bH}]+[\text{fC3bHL}]+[\text{fC3bCR1}]+[\text{fiC3bCR1}]+[\text{IgGC3bC3bH}]+[\text{IgGC3biC3bH}]+[\text{IgGC3bC3bHL}]}\cdots \\
\dfrac{1}{+[\text{IgGC3biC3bHL}]+[\text{IgGC3bC3bCR1}]+[\text{IgGC3biC3bCR1}]+[\text{IgGiC3biC3bCR1}]+[\text{IgGiC3bC3dgCR1}]+[\text{fC3bC4bH}]+[\text{fC3bC4bHL}]}\cdots \\
\dfrac{1}{+[\text{fC3bC4bCR1}]+[\text{fC3bC4bC4BP}]+[\text{fC3bC4dH}]+[\text{fC3bC4dHL}]+[\text{fC3bC4dCR1}]+[\text{fiC3bC4dCR1}]+[\text{fiC3bC4bCR1}]+[\text{fiC3bC4bC4BP}]}\cdots \\
\dfrac{1}{+[\text{fC3dgC4bCR1}]+[\text{fC3dgC4bC4BP}]+[\text{fC3bC3bH}]+[\text{fC3bC3bHL}]+[\text{fC3bC3bCR1}]+[\text{fC3biC3bH}]+[\text{fC3biC3bHL}]+[\text{fC3biC3bCR1}]}\cdots \\
\dfrac{1}{+[\text{fiC3biC3bCR1}]+[\text{fiC3bC3dgCR1}]+[\text{hC3bH}]+[\text{hC3bHL}]+[\text{hC3bCR1}]+[\text{hiC3bCR1}]+[\text{hC3bC4bH}]+[\text{hC3bC4bHL}]+[\text{hC3bC4bCR1}]}\cdots \\
\dfrac{1}{+[\text{hC3bC4bC4BP}]+[\text{hC3bC4dH}]+[\text{hC3bC4dHL}]+[\text{hC3bC4dCR1}]+[\text{hiC3bC4dCR1}]+[\text{hiC3bC4bCR1}]+[\text{hiC3bC4bC4BP}]+[\text{hC3dgC4bCR1}]}\cdots \\
\dfrac{1}{+[\text{hC3dgC4bC4BP}]+[\text{hC3bC3bH}]+[\text{hC3bC3bHL}]+[\text{hC3bC3bCR1}]+[\text{hC3biC3bH}]+[\text{hC3biC3bHL}]+[\text{hC3biC3bCR1}]+[\text{hiC3biC3bCR1}]}\cdots \\
\dfrac{1}{+[\text{hiC3bC3dgCR1}]+[\text{fC4bCR1}]+[\text{nfC4bC4BP}]+[\text{fC4bC4BP}]+[\text{fC4bC4bCR1}]+[\text{fC4bC4bC4BP}]+[\text{fC4bC4dCR1}]+[\text{fC4bC4dC4BP}]+[\text{hC4bCR1}]}\cdots \\
\dfrac{1}{+[\text{hC4bC4BP}]+[\text{hC4bC4bCR1}]+[\text{hC4bC4bC4BP}]+[\text{hC4bC4dCR1}]+[\text{hC4bC4dC4BP}]}\cdots
\end{array}\right)$$

S209

$$\frac{d[\text{hiC3bC4bC4BP}]}{dt} = k^{+}_{\text{C4bC4BP}}[\text{hiC3bC4b}][\text{C4BP}] - k^{-}_{\text{C4bC4BP}}[\text{hiC3bC4bC4BP}]$$

$$-\left(\begin{array}{l}
\dfrac{k^{\text{FI}}_{\text{catC3bH}}[\text{I}][\text{hiC3bC4bC4BP}]}{K^{\text{FI}}_{\text{mC3bH}}+[\text{C3(H}_2\text{O)H}]+[\text{C3(H}_2\text{O)HL}]+[\text{fC3bH}]+[\text{fC3bHL}]+[\text{fC3bCR1}]+[\text{fiC3bCR1}]+[\text{IgGC3bC3bH}]+[\text{IgGC3biC3bH}]+[\text{IgGC3bC3bHL}]}\cdots \\
\dfrac{1}{+[\text{IgGC3biC3bHL}]+[\text{IgGC3bC3bCR1}]+[\text{IgGC3biC3bCR1}]+[\text{IgGiC3biC3bCR1}]+[\text{IgGiC3bC3dgCR1}]+[\text{fC3bC4bH}]+[\text{fC3bC4bHL}]}\cdots \\
\dfrac{1}{+[\text{fC3bC4bCR1}]+[\text{fC3bC4bC4BP}]+[\text{fC3bC4dH}]+[\text{fC3bC4dHL}]+[\text{fC3bC4dCR1}]+[\text{fiC3bC4dCR1}]+[\text{fiC3bC4bCR1}]+[\text{fiC3bC4bC4BP}]}\cdots \\
\dfrac{1}{+[\text{fC3dgC4bCR1}]+[\text{fC3dgC4bC4BP}]+[\text{fC3bC3bH}]+[\text{fC3bC3bHL}]+[\text{fC3bC3bCR1}]+[\text{fC3biC3bH}]+[\text{fC3biC3bHL}]+[\text{fC3biC3bCR1}]}\cdots \\
\dfrac{1}{+[\text{fiC3biC3bCR1}]+[\text{fiC3bC3dgCR1}]+[\text{hC3bH}]+[\text{hC3bHL}]+[\text{hC3bCR1}]+[\text{hiC3bCR1}]+[\text{hC3bC4bH}]+[\text{hC3bC4bHL}]+[\text{hC3bC4bCR1}]}\cdots \\
\dfrac{1}{+[\text{hC3bC4bC4BP}]+[\text{hC3bC4dH}]+[\text{hC3bC4dHL}]+[\text{hC3bC4dCR1}]+[\text{hiC3bC4dCR1}]+[\text{hiC3bC4bCR1}]+[\text{hiC3bC4bC4BP}]+[\text{hC3dgC4bCR1}]}\cdots \\
\dfrac{1}{+[\text{hC3dgC4bC4BP}]+[\text{hC3bC3bH}]+[\text{hC3bC3bHL}]+[\text{hC3bC3bCR1}]+[\text{hC3biC3bH}]+[\text{hC3biC3bHL}]+[\text{hC3biC3bCR1}]+[\text{hiC3biC3bCR1}]}\cdots \\
\dfrac{1}{+[\text{hiC3bC3dgCR1}]+[\text{fC4bCR1}]+[\text{nfC4bC4BP}]+[\text{fC4bC4BP}]+[\text{fC4bC4bCR1}]+[\text{fC4bC4bC4BP}]+[\text{fC4bC4dCR1}]+[\text{fC4bC4dC4BP}]+[\text{hC4bCR1}]}\cdots \\
\dfrac{1}{+[\text{hC4bC4BP}]+[\text{hC4bC4bCR1}]+[\text{hC4bC4bC4BP}]+[\text{hC4bC4dCR1}]+[\text{hC4bC4dC4BP}]}\cdots
\end{array}\right)$$

S210

$$\frac{d[\text{hC3bC4d}]}{dt} =$$

$$\left(\frac{k_{\text{catC3bH}}^{\text{FI}}[\text{I}][\text{hC3bC4bC4BP}]}{K_{\text{mC3bH}}^{\text{FI}} + [\text{C3(H}_2\text{O)H}] + [\text{C3(H}_2\text{O)HL}] + [\text{fC3bH}] + [\text{fC3bHL}] + [\text{fC3bCR1}] + [\text{fiC3bCR1}] + [\text{IgGC3bC3bH}] + [\text{IgGC3biC3bH}] + [\text{IgGC3bC3bHL}]} \cdots \right.$$
$$\frac{1}{+ [\text{IgGC3biC3bHL}] + [\text{IgGC3bC3bCR1}] + [\text{IgGC3biC3bCR1}] + [\text{IgGiC3biC3bCR1}] + [\text{IgGiC3bC3dgCR1}] + [\text{fC3bC4bH}] + [\text{fC3bC4bHL}]} \cdots$$
$$\frac{1}{+ [\text{fC3bC4bCR1}] + [\text{fC3bC4bC4BP}] + [\text{fC3bC4dH}] + [\text{fC3bC4dHL}] + [\text{fC3bC4dCR1}] + [\text{fiC3bC4dCR1}] + [\text{fiC3bC4bCR1}] + [\text{fiC3bC4bC4BP}]} \cdots$$
$$\frac{1}{+ [\text{fC3dgC4bCR1}] + [\text{fC3dgC4bC4BP}] + [\text{fC3bC3bH}] + [\text{fC3bC3bHL}] + [\text{fC3bC3bCR1}] + [\text{fC3biC3bH}] + [\text{fC3biC3bHL}] + [\text{fC3biC3bCR1}]} \cdots$$
$$\frac{1}{+ [\text{fiC3biC3bCR1}] + [\text{fiC3bC3dgCR1}] + [\text{hC3bH}] + [\text{hC3bHL}] + [\text{hC3bCR1}] + [\text{hiC3bCR1}] + [\text{hC3bC4bH}] + [\text{hC3bC4bHL}] + [\text{hC3bC4bCR1}]} \cdots$$
$$\frac{1}{+ [\text{hC3bC4bC4BP}] + [\text{hC3bC4dH}] + [\text{hC3bC4dHL}] + [\text{hC3bC4dCR1}] + [\text{hiC3bC4dCR1}] + [\text{hiC3bC4bCR1}] + [\text{hiC3bC4bC4BP}] + [\text{hC3dgC4bCR1}]} \cdots$$
$$\frac{1}{+ [\text{hC3dgC4bC4BP}] + [\text{hC3bC3bH}] + [\text{hC3bC3bHL}] + [\text{hC3bC3bCR1}] + [\text{hC3biC3bH}] + [\text{hC3biC3bHL}] + [\text{hC3biC3bCR1}] + [\text{hiC3biC3bCR1}]} \cdots$$
$$\frac{1}{+ [\text{hiC3bC3dgCR1}] + [\text{fC4bCR1}] + [\text{nfC4bC4BP}] + [\text{fC4bC4BP}] + [\text{fC4bC4bCR1}] + [\text{fC4bC4bC4BP}] + [\text{fC4bC4dCR1}] + [\text{fC4bC4dC4BP}] + [\text{hC4bCR1}]} \cdots$$
$$\left. \frac{1}{+ [\text{hC4bC4BP}] + [\text{hC4bC4bCR1}] + [\text{hC4bC4bC4BP}] + [\text{hC4bC4dCR1}] + [\text{hC4bC4dC4BP}]} \cdots \right)$$

$$- k_{\text{C3bH}}^{+}[\text{hC3bC4d}][\text{H}] + k_{\text{C3bH}}^{-}[\text{hC3bC4dH}] - k_{\text{C3bHL}}^{+}[\text{hC3bC4d}][\text{HL}] + k_{\text{C3bHL}}^{-}[\text{hC3bC4dHL}] - k_{\text{C3bC4dCR1}}^{+}[\text{hC3bC4d}][\text{CR1}]$$
$$+ k_{\text{C3bC4dCR1}}^{-}[\text{hC3bC4dCR1}]$$

S211

$$\frac{d[\text{hC3bC4dH}]}{dt} = k_{\text{C3bH}}^{+}[\text{hC3bC4d}][\text{H}] - k_{\text{C3bH}}^{-}[\text{hC3bC4dH}]$$

$$- \left(\frac{k_{\text{catC3bH}}^{\text{FI}}[\text{I}][\text{hC3bC4dH}]}{K_{\text{mC3bH}}^{\text{FI}} + [\text{C3(H}_2\text{O)H}] + [\text{C3(H}_2\text{O)HL}] + [\text{fC3bH}] + [\text{fC3bHL}] + [\text{fC3bCR1}] + [\text{fiC3bCR1}] + [\text{IgGC3bC3bH}] + [\text{IgGC3biC3bH}] + [\text{IgGC3bC3bHL}]} \cdots \right.$$
$$\frac{1}{+ [\text{IgGC3biC3bHL}] + [\text{IgGC3bC3bCR1}] + [\text{IgGC3biC3bCR1}] + [\text{IgGiC3biC3bCR1}] + [\text{IgGiC3bC3dgCR1}] + [\text{fC3bC4bH}] + [\text{fC3bC4bHL}]} \cdots$$
$$\frac{1}{+ [\text{fC3bC4bCR1}] + [\text{fC3bC4bC4BP}] + [\text{fC3bC4dH}] + [\text{fC3bC4dHL}] + [\text{fC3bC4dCR1}] + [\text{fiC3bC4dCR1}] + [\text{fiC3bC4bCR1}] + [\text{fiC3bC4bC4BP}]} \cdots$$
$$\frac{1}{+ [\text{fC3dgC4bCR1}] + [\text{fC3dgC4bC4BP}] + [\text{fC3bC3bH}] + [\text{fC3bC3bHL}] + [\text{fC3bC3bCR1}] + [\text{fC3biC3bH}] + [\text{fC3biC3bHL}] + [\text{fC3biC3bCR1}]} \cdots$$
$$\frac{1}{+ [\text{fiC3biC3bCR1}] + [\text{fiC3bC3dgCR1}] + [\text{hC3bH}] + [\text{hC3bHL}] + [\text{hC3bCR1}] + [\text{hiC3bCR1}] + [\text{hC3bC4bH}] + [\text{hC3bC4bHL}] + [\text{hC3bC4bCR1}]} \cdots$$
$$\frac{1}{+ [\text{hC3bC4bC4BP}] + [\text{hC3bC4dH}] + [\text{hC3bC4dHL}] + [\text{hC3bC4dCR1}] + [\text{hiC3bC4dCR1}] + [\text{hiC3bC4bCR1}] + [\text{hiC3bC4bC4BP}] + [\text{hC3dgC4bCR1}]} \cdots$$
$$\frac{1}{+ [\text{hC3dgC4bC4BP}] + [\text{hC3bC3bH}] + [\text{hC3bC3bHL}] + [\text{hC3bC3bCR1}] + [\text{hC3biC3bH}] + [\text{hC3biC3bHL}] + [\text{hC3biC3bCR1}] + [\text{hiC3biC3bCR1}]} \cdots$$
$$\frac{1}{+ [\text{hiC3bC3dgCR1}] + [\text{fC4bCR1}] + [\text{nfC4bC4BP}] + [\text{fC4bC4BP}] + [\text{fC4bC4bCR1}] + [\text{fC4bC4bC4BP}] + [\text{fC4bC4dCR1}] + [\text{fC4bC4dC4BP}] + [\text{hC4bCR1}]} \cdots$$
$$\left. \frac{1}{+ [\text{hC4bC4BP}] + [\text{hC4bC4bCR1}] + [\text{hC4bC4bC4BP}] + [\text{hC4bC4dCR1}] + [\text{hC4bC4dC4BP}]} \cdots \right)$$

S212

$$\frac{d[\mathrm{hC3bC4dHL}]}{dt} = k^{+}_{\mathrm{C3bHL}}[\mathrm{hC3bC4d}][\mathrm{HL}] - k^{-}_{\mathrm{C3bHL}}[\mathrm{hC3bC4dHL}]$$

$$- \left(\frac{k^{\mathrm{FI}}_{\mathrm{catC3bH}}[\mathrm{I}][\mathrm{hC3bC4dHL}]}{K^{\mathrm{FI}}_{\mathrm{mC3bH}} + [\mathrm{C3(H_2O)H}] + [\mathrm{C3(H_2O)HL}] + [\mathrm{fC3bH}] + [\mathrm{fC3bHL}] + [\mathrm{fC3bCR1}] + [\mathrm{fiC3bCR1}] + [\mathrm{IgGC3bC3bH}] + [\mathrm{IgGC3biC3bH}] + [\mathrm{IgGC3bC3bHL}]} \cdots \right.$$
$$\frac{1}{+[\mathrm{IgGC3biC3bHL}] + [\mathrm{IgGC3bC3bCR1}] + [\mathrm{IgGC3biC3bCR1}] + [\mathrm{IgGiC3biC3bCR1}] + [\mathrm{IgGiC3bC3dgCR1}] + [\mathrm{fC3bC4bH}] + [\mathrm{fC3bC4bHL}]} \cdots$$
$$\frac{1}{+[\mathrm{fC3bC4bCR1}] + [\mathrm{fC3bC4bC4BP}] + [\mathrm{fC3bC4dH}] + [\mathrm{fC3bC4dHL}] + [\mathrm{fC3bC4dCR1}] + [\mathrm{fiC3bC4dCR1}] + [\mathrm{fiC3bC4bCR1}] + [\mathrm{fiC3bC4bC4BP}]} \cdots$$
$$\frac{1}{+[\mathrm{fC3dgC4bCR1}] + [\mathrm{fC3dgC4bC4BP}] + [\mathrm{fC3bC3bH}] + [\mathrm{fC3bC3bHL}] + [\mathrm{fC3bC3bCR1}] + [\mathrm{fC3biC3bH}] + [\mathrm{fC3biC3bHL}] + [\mathrm{fC3biC3bCR1}]} \cdots$$
$$\frac{1}{+[\mathrm{fiC3biC3bCR1}] + [\mathrm{fiC3bC3dgCR1}] + [\mathrm{hC3bH}] + [\mathrm{hC3bHL}] + [\mathrm{hC3bCR1}] + [\mathrm{hiC3bCR1}] + [\mathrm{hC3bC4bH}] + [\mathrm{hC3bC4bHL}] + [\mathrm{hC3bC4bCR1}]} \cdots$$
$$\frac{1}{+[\mathrm{hC3bC4bC4BP}] + [\mathrm{hC3bC4dH}] + [\mathrm{hC3bC4dHL}] + [\mathrm{hC3bC4dCR1}] + [\mathrm{hiC3bC4dCR1}] + [\mathrm{hiC3bC4bCR1}] + [\mathrm{hiC3bC4bC4BP}] + [\mathrm{hC3dgC4bCR1}]} \cdots$$
$$\frac{1}{+[\mathrm{hC3dgC4bC4BP}] + [\mathrm{hC3bC3bH}] + [\mathrm{hC3bC3bHL}] + [\mathrm{hC3bC3bCR1}] + [\mathrm{hC3biC3bH}] + [\mathrm{hC3biC3bHL}] + [\mathrm{hC3biC3bCR1}] + [\mathrm{hiC3biC3bCR1}]} \cdots$$
$$\frac{1}{+[\mathrm{hiC3bC3dgCR1}] + [\mathrm{fC4bCR1}] + [\mathrm{nfC4bC4BP}] + [\mathrm{fC4bC4BP}] + [\mathrm{fC4bC4bCR1}] + [\mathrm{fC4bC4bC4BP}] + [\mathrm{fC4bC4dCR1}] + [\mathrm{fC4bC4dC4BP}] + [\mathrm{hC4bCR1}]} \cdots$$
$$\left. \frac{1}{+[\mathrm{hC4bC4BP}] + [\mathrm{hC4bC4bCR1}] + [\mathrm{hC4bC4bC4BP}] + [\mathrm{hC4bC4dCR1}] + [\mathrm{hC4bC4dC4BP}]} \cdots \right)$$

S213

$$\frac{d[\mathrm{hC3bC4dCR1}]}{dt} = k^{+}_{\mathrm{C3bC4dCR1}}[\mathrm{hC3bC4d}][\mathrm{CR1}] - k^{-}_{\mathrm{C3bC4dCR1}}[\mathrm{hC3bC4dCR1}]$$

$$- \left(\frac{k^{\mathrm{FI}}_{\mathrm{catC3bH}}[\mathrm{I}][\mathrm{hC3bC4dCR1}]}{K^{\mathrm{FI}}_{\mathrm{mC3bH}} + [\mathrm{C3(H_2O)H}] + [\mathrm{C3(H_2O)HL}] + [\mathrm{fC3bH}] + [\mathrm{fC3bHL}] + [\mathrm{fC3bCR1}] + [\mathrm{fiC3bCR1}] + [\mathrm{IgGC3bC3bH}] + [\mathrm{IgGC3biC3bH}] + [\mathrm{IgGC3bC3bHL}]} \cdots \right.$$
$$\frac{1}{+[\mathrm{IgGC3biC3bHL}] + [\mathrm{IgGC3bC3bCR1}] + [\mathrm{IgGC3biC3bCR1}] + [\mathrm{IgGiC3biC3bCR1}] + [\mathrm{IgGiC3bC3dgCR1}] + [\mathrm{fC3bC4bH}] + [\mathrm{fC3bC4bHL}]} \cdots$$
$$\frac{1}{+[\mathrm{fC3bC4bCR1}] + [\mathrm{fC3bC4bC4BP}] + [\mathrm{fC3bC4dH}] + [\mathrm{fC3bC4dHL}] + [\mathrm{fC3bC4dCR1}] + [\mathrm{fiC3bC4dCR1}] + [\mathrm{fiC3bC4bCR1}] + [\mathrm{fiC3bC4bC4BP}]} \cdots$$
$$\frac{1}{+[\mathrm{fC3dgC4bCR1}] + [\mathrm{fC3dgC4bC4BP}] + [\mathrm{fC3bC3bH}] + [\mathrm{fC3bC3bHL}] + [\mathrm{fC3bC3bCR1}] + [\mathrm{fC3biC3bH}] + [\mathrm{fC3biC3bHL}] + [\mathrm{fC3biC3bCR1}]} \cdots$$
$$\frac{1}{+[\mathrm{fiC3biC3bCR1}] + [\mathrm{fiC3bC3dgCR1}] + [\mathrm{hC3bH}] + [\mathrm{hC3bHL}] + [\mathrm{hC3bCR1}] + [\mathrm{hiC3bCR1}] + [\mathrm{hC3bC4bH}] + [\mathrm{hC3bC4bHL}] + [\mathrm{hC3bC4bCR1}]} \cdots$$
$$\frac{1}{+[\mathrm{hC3bC4bC4BP}] + [\mathrm{hC3bC4dH}] + [\mathrm{hC3bC4dHL}] + [\mathrm{hC3bC4dCR1}] + [\mathrm{hiC3bC4dCR1}] + [\mathrm{hiC3bC4bCR1}] + [\mathrm{hiC3bC4bC4BP}] + [\mathrm{hC3dgC4bCR1}]} \cdots$$
$$\frac{1}{+[\mathrm{hC3dgC4bC4BP}] + [\mathrm{hC3bC3bH}] + [\mathrm{hC3bC3bHL}] + [\mathrm{hC3bC3bCR1}] + [\mathrm{hC3biC3bH}] + [\mathrm{hC3biC3bHL}] + [\mathrm{hC3biC3bCR1}] + [\mathrm{hiC3biC3bCR1}]} \cdots$$
$$\frac{1}{+[\mathrm{hiC3bC3dgCR1}] + [\mathrm{fC4bCR1}] + [\mathrm{nfC4bC4BP}] + [\mathrm{fC4bC4BP}] + [\mathrm{fC4bC4bCR1}] + [\mathrm{fC4bC4bC4BP}] + [\mathrm{fC4bC4dCR1}] + [\mathrm{fC4bC4dC4BP}] + [\mathrm{hC4bCR1}]} \cdots$$
$$\left. \frac{1}{+[\mathrm{hC4bC4BP}] + [\mathrm{hC4bC4bCR1}] + [\mathrm{hC4bC4bC4BP}] + [\mathrm{hC4bC4dCR1}] + [\mathrm{hC4bC4dC4BP}]} \cdots \right)$$

S214

$$\frac{d[\mathrm{hiC3bC4d}]}{dt} =$$

$$\left(\frac{k^{\mathrm{FI}}_{\mathrm{catC3bH}}[\mathrm{I}][\mathrm{hC3bC4dH}]}{K^{\mathrm{FI}}_{\mathrm{mC3bH}} + [\mathrm{C3(H_2O)H}] + [\mathrm{C3(H_2O)HL}] + [\mathrm{fC3bH}] + [\mathrm{fC3bHL}] + [\mathrm{fC3bCR1}] + [\mathrm{fiC3bCR1}] + [\mathrm{IgGC3bC3bH}] + [\mathrm{IgGC3biC3bH}] + [\mathrm{IgGC3bC3bHL}]} \cdots \right.$$

$$\frac{1}{+ [\mathrm{IgGC3biC3bHL}] + [\mathrm{IgGC3bC3bCR1}] + [\mathrm{IgGC3biC3bCR1}] + [\mathrm{IgGiC3biC3bCR1}] + [\mathrm{IgGiC3bC3dgCR1}] + [\mathrm{fC3bC4bH}] + [\mathrm{fC3bC4bHL}]} \cdots$$

$$\frac{1}{+ [\mathrm{fC3bC4bCR1}] + [\mathrm{fC3bC4bC4BP}] + [\mathrm{fC3bC4dH}] + [\mathrm{fC3bC4dHL}] + [\mathrm{fC3bC4dCR1}] + [\mathrm{fiC3bC4dCR1}] + [\mathrm{fiC3bC4bCR1}] + [\mathrm{fiC3bC4bC4BP}]} \cdots$$

$$\frac{1}{+ [\mathrm{fC3dgC4bCR1}] + [\mathrm{fC3dgC4bC4BP}] + [\mathrm{fC3bC3bH}] + [\mathrm{fC3bC3bHL}] + [\mathrm{fC3bC3bCR1}] + [\mathrm{fC3biC3bH}] + [\mathrm{fC3biC3bHL}] + [\mathrm{fC3biC3bCR1}]} \cdots$$

$$\frac{1}{+ [\mathrm{fiC3biC3bCR1}] + [\mathrm{fiC3bC3dgCR1}] + [\mathrm{hC3bH}] + [\mathrm{hC3bHL}] + [\mathrm{hC3bCR1}] + [\mathrm{hiC3bCR1}] + [\mathrm{hC3bC4bH}] + [\mathrm{hC3bC4bHL}] + [\mathrm{hC3bC4bCR1}]} \cdots$$

$$\frac{1}{+ [\mathrm{hC3bC4bC4BP}] + [\mathrm{hC3bC4dH}] + [\mathrm{hC3bC4dHL}] + [\mathrm{hC3bC4dCR1}] + [\mathrm{hiC3bC4dCR1}] + [\mathrm{hiC3bC4bCR1}] + [\mathrm{hiC3bC4bC4BP}] + [\mathrm{hC3dgC4bCR1}]} \cdots$$

$$\frac{1}{+ [\mathrm{hC3dgC4bC4BP}] + [\mathrm{hC3bC3bH}] + [\mathrm{hC3bC3bHL}] + [\mathrm{hC3bC3bCR1}] + [\mathrm{hC3biC3bH}] + [\mathrm{hC3biC3bHL}] + [\mathrm{hC3biC3bCR1}] + [\mathrm{hiC3biC3bCR1}]} \cdots$$

$$\frac{1}{+ [\mathrm{hiC3bC3dgCR1}] + [\mathrm{fC4bCR1}] + [\mathrm{nfC4bC4BP}] + [\mathrm{fC4bC4BP}] + [\mathrm{fC4bC4bCR1}] + [\mathrm{fC4bC4bC4BP}] + [\mathrm{fC4bC4dCR1}] + [\mathrm{fC4bC4dC4BP}] + [\mathrm{hC4bCR1}]} \cdots$$

$$\left. \frac{1}{+ [\mathrm{hC4bC4BP}] + [\mathrm{hC4bC4bCR1}] + [\mathrm{hC4bC4bC4BP}] + [\mathrm{hC4bC4dCR1}] + [\mathrm{hC4bC4dC4BP}]} \cdots \right)$$

$$+ \left(\frac{k^{\mathrm{FI}}_{\mathrm{catC3bH}}[\mathrm{I}][\mathrm{hC3bC4dHL}]}{K^{\mathrm{FI}}_{\mathrm{mC3bH}} + [\mathrm{C3(H_2O)H}] + [\mathrm{C3(H_2O)HL}] + [\mathrm{fC3bH}] + [\mathrm{fC3bHL}] + [\mathrm{fC3bCR1}] + [\mathrm{fiC3bCR1}] + [\mathrm{IgGC3bC3bH}] + [\mathrm{IgGC3biC3bH}] + [\mathrm{IgGC3bC3bHL}]} \cdots \right.$$

$$\frac{1}{+ [\mathrm{IgGC3biC3bHL}] + [\mathrm{IgGC3bC3bCR1}] + [\mathrm{IgGC3biC3bCR1}] + [\mathrm{IgGiC3biC3bCR1}] + [\mathrm{IgGiC3bC3dgCR1}] + [\mathrm{fC3bC4bH}] + [\mathrm{fC3bC4bHL}]} \cdots$$

$$\frac{1}{+ [\mathrm{fC3bC4bCR1}] + [\mathrm{fC3bC4bC4BP}] + [\mathrm{fC3bC4dH}] + [\mathrm{fC3bC4dHL}] + [\mathrm{fC3bC4dCR1}] + [\mathrm{fiC3bC4dCR1}] + [\mathrm{fiC3bC4bCR1}] + [\mathrm{fiC3bC4bC4BP}]} \cdots$$

$$\frac{1}{+ [\mathrm{fC3dgC4bCR1}] + [\mathrm{fC3dgC4bC4BP}] + [\mathrm{fC3bC3bH}] + [\mathrm{fC3bC3bHL}] + [\mathrm{fC3bC3bCR1}] + [\mathrm{fC3biC3bH}] + [\mathrm{fC3biC3bHL}] + [\mathrm{fC3biC3bCR1}]} \cdots$$

$$\frac{1}{+ [\mathrm{fiC3biC3bCR1}] + [\mathrm{fiC3bC3dgCR1}] + [\mathrm{hC3bH}] + [\mathrm{hC3bHL}] + [\mathrm{hC3bCR1}] + [\mathrm{hiC3bCR1}] + [\mathrm{hC3bC4bH}] + [\mathrm{hC3bC4bHL}] + [\mathrm{hC3bC4bCR1}]} \cdots$$

$$\frac{1}{+ [\mathrm{hC3bC4bC4BP}] + [\mathrm{hC3bC4dH}] + [\mathrm{hC3bC4dHL}] + [\mathrm{hC3bC4dCR1}] + [\mathrm{hiC3bC4dCR1}] + [\mathrm{hiC3bC4bCR1}] + [\mathrm{hiC3bC4bC4BP}] + [\mathrm{hC3dgC4bCR1}]} \cdots$$

$$\frac{1}{+ [\mathrm{hC3dgC4bC4BP}] + [\mathrm{hC3bC3bH}] + [\mathrm{hC3bC3bHL}] + [\mathrm{hC3bC3bCR1}] + [\mathrm{hC3biC3bH}] + [\mathrm{hC3biC3bHL}] + [\mathrm{hC3biC3bCR1}] + [\mathrm{hiC3biC3bCR1}]} \cdots$$

$$\frac{1}{+ [\mathrm{hiC3bC3dgCR1}] + [\mathrm{fC4bCR1}] + [\mathrm{nfC4bC4BP}] + [\mathrm{fC4bC4BP}] + [\mathrm{fC4bC4bCR1}] + [\mathrm{fC4bC4bC4BP}] + [\mathrm{fC4bC4dCR1}] + [\mathrm{fC4bC4dC4BP}] + [\mathrm{hC4bCR1}]} \cdots$$

$$\left. \frac{1}{+ [\mathrm{hC4bC4BP}] + [\mathrm{hC4bC4bCR1}] + [\mathrm{hC4bC4bC4BP}] + [\mathrm{hC4bC4dCR1}] + [\mathrm{hC4bC4dC4BP}]} \cdots \right)$$

$$+\left(\frac{k_{\mathrm{catC3bH}}^{\mathrm{FI}}[\mathrm{I}][\mathrm{hC3bC4dCR1}]}{K_{\mathrm{mC3bH}}^{\mathrm{FI}}+[\mathrm{C3(H_2O)H}]+[\mathrm{C3(H_2O)HL}]+[\mathrm{fC3bH}]+[\mathrm{fC3bHL}]+[\mathrm{fC3bCR1}]+[\mathrm{fiC3bCR1}]+[\mathrm{IgGC3bC3bH}]+[\mathrm{IgGC3biC3bH}]+[\mathrm{IgGC3bC3bHL}]}\cdots\right.$$

$$\frac{1}{+[\mathrm{IgGC3biC3bHL}]+[\mathrm{IgGC3bC3bCR1}]+[\mathrm{IgGC3biC3bCR1}]+[\mathrm{IgGiC3biC3bCR1}]+[\mathrm{IgGiC3bC3dgCR1}]+[\mathrm{fC3bC4bH}]+[\mathrm{fC3bC4bHL}]}\cdots$$

$$\frac{1}{+[\mathrm{fC3bC4bCR1}]+[\mathrm{fC3bC4bC4BP}]+[\mathrm{fC3bC4dH}]+[\mathrm{fC3bC4dHL}]+[\mathrm{fC3bC4dCR1}]+[\mathrm{fiC3bC4dCR1}]+[\mathrm{fiC3bC4bCR1}]+[\mathrm{fiC3bC4bC4BP}]}\cdots$$

$$\frac{1}{+[\mathrm{fC3dgC4bCR1}]+[\mathrm{fC3dgC4bC4BP}]+[\mathrm{fC3bC3bH}]+[\mathrm{fC3bC3bHL}]+[\mathrm{fC3bC3bCR1}]+[\mathrm{fC3biC3bH}]+[\mathrm{fC3biC3bHL}]+[\mathrm{fC3biC3bCR1}]}\cdots$$

$$\frac{1}{+[\mathrm{fiC3biC3bCR1}]+[\mathrm{fiC3bC3dgCR1}]+[\mathrm{hC3bH}]+[\mathrm{hC3bHL}]+[\mathrm{hC3bCR1}]+[\mathrm{hiC3bCR1}]+[\mathrm{hC3bC4bH}]+[\mathrm{hC3bC4bHL}]+[\mathrm{hC3bC4bCR1}]}\cdots$$

$$\frac{1}{+[\mathrm{hC3bC4bC4BP}]+[\mathrm{hC3bC4dH}]+[\mathrm{hC3bC4dHL}]+[\mathrm{hC3bC4dCR1}]+[\mathrm{hiC3bC4dCR1}]+[\mathrm{hiC3bC4bCR1}]+[\mathrm{hiC3bC4bC4BP}]+[\mathrm{hC3dgC4bCR1}]}\cdots$$

$$\frac{1}{+[\mathrm{hC3dgC4bC4BP}]+[\mathrm{hC3bC3bH}]+[\mathrm{hC3bC3bHL}]+[\mathrm{hC3bC3bCR1}]+[\mathrm{hC3biC3bH}]+[\mathrm{hC3biC3bHL}]+[\mathrm{hC3biC3bCR1}]+[\mathrm{hiC3biC3bCR1}]}\cdots$$

$$\frac{1}{+[\mathrm{hiC3bC3dgCR1}]+[\mathrm{fC4bCR1}]+[\mathrm{nfC4bC4BP}]+[\mathrm{fC4bC4BP}]+[\mathrm{fC4bC4bCR1}]+[\mathrm{fC4bC4bC4BP}]+[\mathrm{fC4bC4dCR1}]+[\mathrm{fC4bC4dC4BP}]+[\mathrm{hC4bCR1}]}\cdots$$

$$\left.\frac{1}{+[\mathrm{hC4bC4BP}]+[\mathrm{hC4bC4bCR1}]+[\mathrm{hC4bC4bC4BP}]+[\mathrm{hC4bC4dCR1}]+[\mathrm{hC4bC4dC4BP}]}\cdots\right)$$

$$+\left(\frac{k_{\mathrm{catC3bH}}^{\mathrm{FI}}[\mathrm{I}][\mathrm{hiC3bC4bC4BP}]}{K_{\mathrm{mC3bH}}^{\mathrm{FI}}+[\mathrm{C3(H_2O)H}]+[\mathrm{C3(H_2O)HL}]+[\mathrm{fC3bH}]+[\mathrm{fC3bHL}]+[\mathrm{fC3bCR1}]+[\mathrm{fiC3bCR1}]+[\mathrm{IgGC3bC3bH}]+[\mathrm{IgGC3biC3bH}]+[\mathrm{IgGC3bC3bHL}]}\cdots\right.$$

$$\frac{1}{+[\mathrm{IgGC3biC3bHL}]+[\mathrm{IgGC3bC3bCR1}]+[\mathrm{IgGC3biC3bCR1}]+[\mathrm{IgGiC3biC3bCR1}]+[\mathrm{IgGiC3bC3dgCR1}]+[\mathrm{fC3bC4bH}]+[\mathrm{fC3bC4bHL}]}\cdots$$

$$\frac{1}{+[\mathrm{fC3bC4bCR1}]+[\mathrm{fC3bC4bC4BP}]+[\mathrm{fC3bC4dH}]+[\mathrm{fC3bC4dHL}]+[\mathrm{fC3bC4dCR1}]+[\mathrm{fiC3bC4dCR1}]+[\mathrm{fiC3bC4bCR1}]+[\mathrm{fiC3bC4bC4BP}]}\cdots$$

$$\frac{1}{+[\mathrm{fC3dgC4bCR1}]+[\mathrm{fC3dgC4bC4BP}]+[\mathrm{fC3bC3bH}]+[\mathrm{fC3bC3bHL}]+[\mathrm{fC3bC3bCR1}]+[\mathrm{fC3biC3bH}]+[\mathrm{fC3biC3bHL}]+[\mathrm{fC3biC3bCR1}]}\cdots$$

$$\frac{1}{+[\mathrm{fiC3biC3bCR1}]+[\mathrm{fiC3bC3dgCR1}]+[\mathrm{hC3bH}]+[\mathrm{hC3bHL}]+[\mathrm{hC3bCR1}]+[\mathrm{hiC3bCR1}]+[\mathrm{hC3bC4bH}]+[\mathrm{hC3bC4bHL}]+[\mathrm{hC3bC4bCR1}]}\cdots$$

$$\frac{1}{+[\mathrm{hC3bC4bC4BP}]+[\mathrm{hC3bC4dH}]+[\mathrm{hC3bC4dHL}]+[\mathrm{hC3bC4dCR1}]+[\mathrm{hiC3bC4dCR1}]+[\mathrm{hiC3bC4bCR1}]+[\mathrm{hiC3bC4bC4BP}]+[\mathrm{hC3dgC4bCR1}]}\cdots$$

$$\frac{1}{+[\mathrm{hC3dgC4bC4BP}]+[\mathrm{hC3bC3bH}]+[\mathrm{hC3bC3bHL}]+[\mathrm{hC3bC3bCR1}]+[\mathrm{hC3biC3bH}]+[\mathrm{hC3biC3bHL}]+[\mathrm{hC3biC3bCR1}]+[\mathrm{hiC3biC3bCR1}]}\cdots$$

$$\frac{1}{+[\mathrm{hiC3bC3dgCR1}]+[\mathrm{fC4bCR1}]+[\mathrm{nfC4bC4BP}]+[\mathrm{fC4bC4BP}]+[\mathrm{fC4bC4bCR1}]+[\mathrm{fC4bC4bC4BP}]+[\mathrm{fC4bC4dCR1}]+[\mathrm{fC4bC4dC4BP}]+[\mathrm{hC4bCR1}]}\cdots$$

$$\left.\frac{1}{+[\mathrm{hC4bC4BP}]+[\mathrm{hC4bC4bCR1}]+[\mathrm{hC4bC4bC4BP}]+[\mathrm{hC4bC4dCR1}]+[\mathrm{hC4bC4dC4BP}]}\cdots\right)$$

$$-k_{\mathrm{iC3bC4dCR1}}^{+}[\mathrm{hiC3bC4d}][\mathrm{CR1}]+k_{\mathrm{iC3bC4dCR1}}^{-}[\mathrm{hiC3bC4dCR1}]$$

S215

$$\frac{d[\text{hiC3bC4dCR1}]}{dt} = k^{+}_{\text{iC3bC4dCR1}}[\text{hiC3bC4d}][\text{CR1}] - k^{-}_{\text{iC3bC4dCR1}}[\text{hiC3bC4dCR1}]$$

$$- \left(\frac{k^{\text{FI}}_{\text{catC3bH}}[\text{I}][\text{hiC3bC4dCR1}]}{K^{\text{FI}}_{\text{mC3bH}} + [\text{C3(H}_2\text{O)H}] + [\text{C3(H}_2\text{O)HL}] + [\text{fC3bH}] + [\text{fC3bHL}] + [\text{fC3bCR1}] + [\text{fiC3bCR1}] + [\text{IgGC3bC3bH}] + [\text{IgGC3biC3bH}] + [\text{IgGC3bC3bHL}]} \cdots \right.$$
$$\frac{1}{+[\text{IgGC3biC3bHL}] + [\text{IgGC3bC3bCR1}] + [\text{IgGC3biC3bCR1}] + [\text{IgGiC3biC3bCR1}] + [\text{IgGiC3bC3dgCR1}] + [\text{fC3bC4bH}] + [\text{fC3bC4bHL}]} \cdots$$
$$\frac{1}{+[\text{fC3bC4bCR1}] + [\text{fC3bC4bC4BP}] + [\text{fC3bC4dH}] + [\text{fC3bC4dHL}] + [\text{fC3bC4dCR1}] + [\text{fiC3bC4dCR1}] + [\text{fiC3bC4bCR1}] + [\text{fiC3bC4bC4BP}]} \cdots$$
$$\frac{1}{+[\text{fC3dgC4bCR1}] + [\text{fC3dgC4bC4BP}] + [\text{fC3bC3bH}] + [\text{fC3bC3bHL}] + [\text{fC3bC3bCR1}] + [\text{fC3biC3bH}] + [\text{fC3biC3bHL}] + [\text{fC3biC3bCR1}]} \cdots$$
$$\frac{1}{+[\text{fiC3biC3bCR1}] + [\text{fiC3bC3dgCR1}] + [\text{hC3bH}] + [\text{hC3bHL}] + [\text{hC3bCR1}] + [\text{hiC3bCR1}] + [\text{hC3bC4bH}] + [\text{hC3bC4bHL}] + [\text{hC3bC4bCR1}]} \cdots$$
$$\frac{1}{+[\text{hC3bC4bC4BP}] + [\text{hC3bC4dH}] + [\text{hC3bC4dHL}] + [\text{hC3bC4dCR1}] + [\text{hiC3bC4dCR1}] + [\text{hiC3bC4bCR1}] + [\text{hiC3bC4bC4BP}] + [\text{hC3dgC4bCR1}]} \cdots$$
$$\frac{1}{+[\text{hC3dgC4bC4BP}] + [\text{hC3bC3bH}] + [\text{hC3bC3bHL}] + [\text{hC3bC3bCR1}] + [\text{hC3biC3bH}] + [\text{hC3biC3bHL}] + [\text{hC3biC3bCR1}] + [\text{hiC3biC3bCR1}]} \cdots$$
$$\frac{1}{+[\text{hiC3bC3dgCR1}] + [\text{fC4bCR1}] + [\text{nfC4bC4BP}] + [\text{fC4bC4BP}] + [\text{fC4bC4bCR1}] + [\text{fC4bC4bC4BP}] + [\text{fC4bC4dCR1}] + [\text{fC4bC4dC4BP}] + [\text{hC4bCR1}]} \cdots$$
$$\left. \frac{1}{+[\text{hC4bC4BP}] + [\text{hC4bC4bCR1}] + [\text{hC4bC4bC4BP}] + [\text{hC4bC4dCR1}] + [\text{hC4bC4dC4BP}]} \cdots \right)$$

S216

$$\frac{d[\text{hC3dgC4b}]}{dt} =$$

$$\left(\frac{k^{\text{FI}}_{\text{catC3bH}}[\text{I}][\text{hiC3bC4bCR1}]}{K^{\text{FI}}_{\text{mC3bH}} + [\text{C3(H}_2\text{O)H}] + [\text{C3(H}_2\text{O)HL}] + [\text{fC3bH}] + [\text{fC3bHL}] + [\text{fC3bCR1}] + [\text{fiC3bCR1}] + [\text{IgGC3bC3bH}] + [\text{IgGC3biC3bH}] + [\text{IgGC3bC3bHL}]} \cdots \right.$$
$$\frac{1}{+[\text{IgGC3biC3bHL}] + [\text{IgGC3bC3bCR1}] + [\text{IgGC3biC3bCR1}] + [\text{IgGiC3biC3bCR1}] + [\text{IgGiC3bC3dgCR1}] + [\text{fC3bC4bH}] + [\text{fC3bC4bHL}]} \cdots$$
$$\frac{1}{+[\text{fC3bC4bCR1}] + [\text{fC3bC4bC4BP}] + [\text{fC3bC4dH}] + [\text{fC3bC4dHL}] + [\text{fC3bC4dCR1}] + [\text{fiC3bC4dCR1}] + [\text{fiC3bC4bCR1}] + [\text{fiC3bC4bC4BP}]} \cdots$$
$$\frac{1}{+[\text{fC3dgC4bCR1}] + [\text{fC3dgC4bC4BP}] + [\text{fC3bC3bH}] + [\text{fC3bC3bHL}] + [\text{fC3bC3bCR1}] + [\text{fC3biC3bH}] + [\text{fC3biC3bHL}] + [\text{fC3biC3bCR1}]} \cdots$$
$$\frac{1}{+[\text{fiC3biC3bCR1}] + [\text{fiC3bC3dgCR1}] + [\text{hC3bH}] + [\text{hC3bHL}] + [\text{hC3bCR1}] + [\text{hiC3bCR1}] + [\text{hC3bC4bH}] + [\text{hC3bC4bHL}] + [\text{hC3bC4bCR1}]} \cdots$$
$$\frac{1}{+[\text{hC3bC4bC4BP}] + [\text{hC3bC4dH}] + [\text{hC3bC4dHL}] + [\text{hC3bC4dCR1}] + [\text{hiC3bC4dCR1}] + [\text{hiC3bC4bCR1}] + [\text{hiC3bC4bC4BP}] + [\text{hC3dgC4bCR1}]} \cdots$$
$$\frac{1}{+[\text{hC3dgC4bC4BP}] + [\text{hC3bC3bH}] + [\text{hC3bC3bHL}] + [\text{hC3bC3bCR1}] + [\text{hC3biC3bH}] + [\text{hC3biC3bHL}] + [\text{hC3biC3bCR1}] + [\text{hiC3biC3bCR1}]} \cdots$$
$$\frac{1}{+[\text{hiC3bC3dgCR1}] + [\text{fC4bCR1}] + [\text{nfC4bC4BP}] + [\text{fC4bC4BP}] + [\text{fC4bC4bCR1}] + [\text{fC4bC4bC4BP}] + [\text{fC4bC4dCR1}] + [\text{fC4bC4dC4BP}] + [\text{hC4bCR1}]} \cdots$$
$$\left. \frac{1}{+[\text{hC4bC4BP}] + [\text{hC4bC4bCR1}] + [\text{hC4bC4bC4BP}] + [\text{hC4bC4dCR1}] + [\text{hC4bC4dC4BP}]} \cdots \right)$$

$$- k^{+}_{\text{C3dgC4bCR1}}[\text{hC3dgC4b}][\text{CR1}] + k^{-}_{\text{C3dgC4bCR1}}[\text{hC3dgC4bCR1}] - k^{+}_{\text{C4bC4BP}}[\text{hC3dgC4b}][\text{C4BP}] + k^{-}_{\text{C4bC4BP}}[\text{hC3dgC4bC4BP}]$$

S217

$$\frac{d[\mathrm{hC3dgC4bCR1}]}{dt}=k^{+}_{\mathrm{C3dgC4bCR1}}[\mathrm{hC3dgC4b}][\mathrm{CR1}]-k^{-}_{\mathrm{C3dgC4bCR1}}[\mathrm{hC3dgC4bCR1}]$$

$$-\left(\frac{k^{\mathrm{FI}}_{\mathrm{catC3bH}}[\mathrm{I}][\mathrm{hC3dgC4bCR1}]}{K^{\mathrm{FI}}_{\mathrm{mC3bH}}+[\mathrm{C3(H_2O)H}]+[\mathrm{C3(H_2O)HL}]+[\mathrm{fC3bH}]+[\mathrm{fC3bHL}]+[\mathrm{fC3bCR1}]+[\mathrm{fiC3bCR1}]+[\mathrm{IgGC3bC3bH}]+[\mathrm{IgGC3biC3bH}]+[\mathrm{IgGC3bC3bHL}]}\cdots\right.$$
$$\frac{1}{+[\mathrm{IgGC3biC3bHL}]+[\mathrm{IgGC3bC3bCR1}]+[\mathrm{IgGC3biC3bCR1}]+[\mathrm{IgGiC3biC3bCR1}]+[\mathrm{IgGiC3bC3dgCR1}]+[\mathrm{fC3bC4bH}]+[\mathrm{fC3bC4bHL}]}\cdots$$
$$\frac{1}{+[\mathrm{fC3bC4bCR1}]+[\mathrm{fC3bC4bC4BP}]+[\mathrm{fC3bC4dH}]+[\mathrm{fC3bC4dHL}]+[\mathrm{fC3bC4dCR1}]+[\mathrm{fiC3bC4dCR1}]+[\mathrm{fiC3bC4bCR1}]+[\mathrm{fiC3bC4bC4BP}]}\cdots$$
$$\frac{1}{+[\mathrm{fC3dgC4bCR1}]+[\mathrm{fC3dgC4bC4BP}]+[\mathrm{fC3bC3bH}]+[\mathrm{fC3bC3bHL}]+[\mathrm{fC3bC3bCR1}]+[\mathrm{fC3biC3bH}]+[\mathrm{fC3biC3bHL}]+[\mathrm{fC3biC3bCR1}]}\cdots$$
$$\frac{1}{+[\mathrm{fiC3biC3bCR1}]+[\mathrm{fiC3bC3dgCR1}]+[\mathrm{hC3bH}]+[\mathrm{hC3bHL}]+[\mathrm{hC3bCR1}]+[\mathrm{hiC3bCR1}]+[\mathrm{hC3bC4bH}]+[\mathrm{hC3bC4bHL}]+[\mathrm{hC3bC4bCR1}]}\cdots$$
$$\frac{1}{+[\mathrm{hC3bC4bC4BP}]+[\mathrm{hC3bC4dH}]+[\mathrm{hC3bC4dHL}]+[\mathrm{hC3bC4dCR1}]+[\mathrm{hiC3bC4dCR1}]+[\mathrm{hiC3bC4bCR1}]+[\mathrm{hiC3bC4bC4BP}]+[\mathrm{hC3dgC4bCR1}]}\cdots$$
$$\frac{1}{+[\mathrm{hC3dgC4bC4BP}]+[\mathrm{hC3bC3bH}]+[\mathrm{hC3bC3bHL}]+[\mathrm{hC3bC3bCR1}]+[\mathrm{hC3biC3bH}]+[\mathrm{hC3biC3bHL}]+[\mathrm{hC3biC3bCR1}]+[\mathrm{hiC3biC3bCR1}]}\cdots$$
$$\frac{1}{+[\mathrm{hiC3bC3dgCR1}]+[\mathrm{fC4bCR1}]+[\mathrm{nfC4bC4BP}]+[\mathrm{fC4bC4BP}]+[\mathrm{fC4bC4bCR1}]+[\mathrm{fC4bC4bC4BP}]+[\mathrm{fC4bC4dCR1}]+[\mathrm{fC4bC4dC4BP}]+[\mathrm{hC4bCR1}]}\cdots$$
$$\left.\frac{1}{+[\mathrm{hC4bC4BP}]+[\mathrm{hC4bC4bCR1}]+[\mathrm{hC4bC4bC4BP}]+[\mathrm{hC4bC4dCR1}]+[\mathrm{hC4bC4dC4BP}]}\cdots\right)$$

S218

$$\frac{d[\mathrm{hC3dgC4bC4BP}]}{dt}=k^{+}_{\mathrm{C4bC4BP}}[\mathrm{hC3dgC4b}][\mathrm{C4BP}]-k^{-}_{\mathrm{C4bC4BP}}[\mathrm{hC3dgC4bC4BP}]$$

$$-\left(\frac{k^{\mathrm{FI}}_{\mathrm{catC3bH}}[\mathrm{I}][\mathrm{hC3dgC4bC4BP}]}{K^{\mathrm{FI}}_{\mathrm{mC3bH}}+[\mathrm{C3(H_2O)H}]+[\mathrm{C3(H_2O)HL}]+[\mathrm{fC3bH}]+[\mathrm{fC3bHL}]+[\mathrm{fC3bCR1}]+[\mathrm{fiC3bCR1}]+[\mathrm{IgGC3bC3bH}]+[\mathrm{IgGC3biC3bH}]+[\mathrm{IgGC3bC3bHL}]}\cdots\right.$$
$$\frac{1}{+[\mathrm{IgGC3biC3bHL}]+[\mathrm{IgGC3bC3bCR1}]+[\mathrm{IgGC3biC3bCR1}]+[\mathrm{IgGiC3biC3bCR1}]+[\mathrm{IgGiC3bC3dgCR1}]+[\mathrm{fC3bC4bH}]+[\mathrm{fC3bC4bHL}]}\cdots$$
$$\frac{1}{+[\mathrm{fC3bC4bCR1}]+[\mathrm{fC3bC4bC4BP}]+[\mathrm{fC3bC4dH}]+[\mathrm{fC3bC4dHL}]+[\mathrm{fC3bC4dCR1}]+[\mathrm{fiC3bC4dCR1}]+[\mathrm{fiC3bC4bCR1}]+[\mathrm{fiC3bC4bC4BP}]}\cdots$$
$$\frac{1}{+[\mathrm{fC3dgC4bCR1}]+[\mathrm{fC3dgC4bC4BP}]+[\mathrm{fC3bC3bH}]+[\mathrm{fC3bC3bHL}]+[\mathrm{fC3bC3bCR1}]+[\mathrm{fC3biC3bH}]+[\mathrm{fC3biC3bHL}]+[\mathrm{fC3biC3bCR1}]}\cdots$$
$$\frac{1}{+[\mathrm{fiC3biC3bCR1}]+[\mathrm{fiC3bC3dgCR1}]+[\mathrm{hC3bH}]+[\mathrm{hC3bHL}]+[\mathrm{hC3bCR1}]+[\mathrm{hiC3bCR1}]+[\mathrm{hC3bC4bH}]+[\mathrm{hC3bC4bHL}]+[\mathrm{hC3bC4bCR1}]}\cdots$$
$$\frac{1}{+[\mathrm{hC3bC4bC4BP}]+[\mathrm{hC3bC4dH}]+[\mathrm{hC3bC4dHL}]+[\mathrm{hC3bC4dCR1}]+[\mathrm{hiC3bC4dCR1}]+[\mathrm{hiC3bC4bCR1}]+[\mathrm{hiC3bC4bC4BP}]+[\mathrm{hC3dgC4bCR1}]}\cdots$$
$$\frac{1}{+[\mathrm{hC3dgC4bC4BP}]+[\mathrm{hC3bC3bH}]+[\mathrm{hC3bC3bHL}]+[\mathrm{hC3bC3bCR1}]+[\mathrm{hC3biC3bH}]+[\mathrm{hC3biC3bHL}]+[\mathrm{hC3biC3bCR1}]+[\mathrm{hiC3biC3bCR1}]}\cdots$$
$$\frac{1}{+[\mathrm{hiC3bC3dgCR1}]+[\mathrm{fC4bCR1}]+[\mathrm{nfC4bC4BP}]+[\mathrm{fC4bC4BP}]+[\mathrm{fC4bC4bCR1}]+[\mathrm{fC4bC4bC4BP}]+[\mathrm{fC4bC4dCR1}]+[\mathrm{fC4bC4dC4BP}]+[\mathrm{hC4bCR1}]}\cdots$$
$$\left.\frac{1}{+[\mathrm{hC4bC4BP}]+[\mathrm{hC4bC4bCR1}]+[\mathrm{hC4bC4bC4BP}]+[\mathrm{hC4bC4dCR1}]+[\mathrm{hC4bC4dC4BP}]}\cdots\right)$$

S219

$$\frac{d[\text{hC3dgC4d}]}{dt} =$$

$$+\left(\frac{k_{\text{catC3bH}}^{\text{FI}}[\text{I}][\text{hiC3bC4dCR1}]}{K_{\text{mC3bH}}^{\text{FI}}+[\text{C3(H}_2\text{O)H}]+[\text{C3(H}_2\text{O)HL}]+[\text{fC3bH}]+[\text{fC3bHL}]+[\text{fC3bCR1}]+[\text{fiC3bCR1}]+[\text{IgGC3bC3bH}]+[\text{IgGC3biC3bH}]+[\text{IgGC3bC3bHL}]}\cdots\right.$$
$$\frac{1}{+[\text{IgGC3biC3bHL}]+[\text{IgGC3bC3bCR1}]+[\text{IgGC3biC3bCR1}]+[\text{IgGiC3biC3bCR1}]+[\text{IgGiC3bC3dgCR1}]+[\text{fC3bC4bH}]+[\text{fC3bC4bHL}]}\cdots$$
$$\frac{1}{+[\text{fC3bC4bCR1}]+[\text{fC3bC4bC4BP}]+[\text{fC3bC4dH}]+[\text{fC3bC4dHL}]+[\text{fC3bC4dCR1}]+[\text{fiC3bC4dCR1}]+[\text{fiC3bC4bCR1}]+[\text{fiC3bC4bC4BP}]}\cdots$$
$$\frac{1}{+[\text{fC3dgC4bCR1}]+[\text{fC3dgC4bC4BP}]+[\text{fC3bC3bH}]+[\text{fC3bC3bHL}]+[\text{fC3bC3bCR1}]+[\text{fC3biC3bH}]+[\text{fC3biC3bHL}]+[\text{fC3biC3bCR1}]}\cdots$$
$$\frac{1}{+[\text{fiC3biC3bCR1}]+[\text{fiC3bC3dgCR1}]+[\text{hC3bH}]+[\text{hC3bHL}]+[\text{hC3bCR1}]+[\text{hiC3bCR1}]+[\text{hC3bC4bH}]+[\text{hC3bC4bHL}]+[\text{hC3bC4bCR1}]}\cdots$$
$$\frac{1}{+[\text{hC3bC4bC4BP}]+[\text{hC3bC4dH}]+[\text{hC3bC4dHL}]+[\text{hC3bC4dCR1}]+[\text{hiC3bC4dCR1}]+[\text{hiC3bC4bCR1}]+[\text{hiC3bC4bC4BP}]+[\text{hC3dgC4bCR1}]}\cdots$$
$$\frac{1}{+[\text{hC3dgC4bC4BP}]+[\text{hC3bC3bH}]+[\text{hC3bC3bHL}]+[\text{hC3bC3bCR1}]+[\text{hC3biC3bH}]+[\text{hC3biC3bHL}]+[\text{hC3biC3bCR1}]+[\text{hiC3biC3bCR1}]}\cdots$$
$$\frac{1}{+[\text{hiC3bC3dgCR1}]+[\text{fC4bCR1}]+[\text{nfC4bC4BP}]+[\text{fC4bC4BP}]+[\text{fC4bC4bCR1}]+[\text{fC4bC4bC4BP}]+[\text{fC4bC4dCR1}]+[\text{fC4bC4dC4BP}]+[\text{hC4bCR1}]}\cdots$$
$$\left.\frac{1}{+[\text{hC4bC4BP}]+[\text{hC4bC4bCR1}]+[\text{hC4bC4bC4BP}]+[\text{hC4bC4dCR1}]+[\text{hC4bC4dC4BP}]}\cdots\right)$$

$$+\left(\frac{k_{\text{catC3bH}}^{\text{FI}}[\text{I}][\text{hC3dgC4bCR1}]}{K_{\text{mC3bH}}^{\text{FI}}+[\text{C3(H}_2\text{O)H}]+[\text{C3(H}_2\text{O)HL}]+[\text{fC3bH}]+[\text{fC3bHL}]+[\text{fC3bCR1}]+[\text{fiC3bCR1}]+[\text{IgGC3bC3bH}]+[\text{IgGC3biC3bH}]+[\text{IgGC3bC3bHL}]}\cdots\right.$$
$$\frac{1}{+[\text{IgGC3biC3bHL}]+[\text{IgGC3bC3bCR1}]+[\text{IgGC3biC3bCR1}]+[\text{IgGiC3biC3bCR1}]+[\text{IgGiC3bC3dgCR1}]+[\text{fC3bC4bH}]+[\text{fC3bC4bHL}]}\cdots$$
$$\frac{1}{+[\text{fC3bC4bCR1}]+[\text{fC3bC4bC4BP}]+[\text{fC3bC4dH}]+[\text{fC3bC4dHL}]+[\text{fC3bC4dCR1}]+[\text{fiC3bC4dCR1}]+[\text{fiC3bC4bCR1}]+[\text{fiC3bC4bC4BP}]}\cdots$$
$$\frac{1}{+[\text{fC3dgC4bCR1}]+[\text{fC3dgC4bC4BP}]+[\text{fC3bC3bH}]+[\text{fC3bC3bHL}]+[\text{fC3bC3bCR1}]+[\text{fC3biC3bH}]+[\text{fC3biC3bHL}]+[\text{fC3biC3bCR1}]}\cdots$$
$$\frac{1}{+[\text{fiC3biC3bCR1}]+[\text{fiC3bC3dgCR1}]+[\text{hC3bH}]+[\text{hC3bHL}]+[\text{hC3bCR1}]+[\text{hiC3bCR1}]+[\text{hC3bC4bH}]+[\text{hC3bC4bHL}]+[\text{hC3bC4bCR1}]}\cdots$$
$$\frac{1}{+[\text{hC3bC4bC4BP}]+[\text{hC3bC4dH}]+[\text{hC3bC4dHL}]+[\text{hC3bC4dCR1}]+[\text{hiC3bC4dCR1}]+[\text{hiC3bC4bCR1}]+[\text{hiC3bC4bC4BP}]+[\text{hC3dgC4bCR1}]}\cdots$$
$$\frac{1}{+[\text{hC3dgC4bC4BP}]+[\text{hC3bC3bH}]+[\text{hC3bC3bHL}]+[\text{hC3bC3bCR1}]+[\text{hC3biC3bH}]+[\text{hC3biC3bHL}]+[\text{hC3biC3bCR1}]+[\text{hiC3biC3bCR1}]}\cdots$$
$$\frac{1}{+[\text{hiC3bC3dgCR1}]+[\text{fC4bCR1}]+[\text{nfC4bC4BP}]+[\text{fC4bC4BP}]+[\text{fC4bC4bCR1}]+[\text{fC4bC4bC4BP}]+[\text{fC4bC4dCR1}]+[\text{fC4bC4dC4BP}]+[\text{hC4bCR1}]}\cdots$$
$$\left.\frac{1}{+[\text{hC4bC4BP}]+[\text{hC4bC4bCR1}]+[\text{hC4bC4bC4BP}]+[\text{hC4bC4dCR1}]+[\text{hC4bC4dC4BP}]}\cdots\right)$$

$$+\left(\begin{array}{l}\frac{k^{FI}_{catC3bH}[I][hC3dgC4bC4BP]}{K^{FI}_{mC3bH}+[C3(H_2O)H]+[C3(H_2O)HL]+[fC3bH]+[fC3bHL]+[fC3bCR1]+[fiC3bCR1]+[IgGC3bC3bH]+[IgGC3biC3bH]+[IgGC3bC3bHL]}\cdots\\ \frac{1}{+[IgGC3biC3bHL]+[IgGC3bC3bCR1]+[IgGC3biC3bCR1]+[IgGiC3biC3bCR1]+[IgGiC3bC3dgCR1]+[fC3bC4bH]+[fC3bC4bHL]}\cdots\\ \frac{1}{+[fC3bC4bCR1]+[fC3bC4bC4BP]+[fC3bC4dH]+[fC3bC4dHL]+[fC3bC4dCR1]+[fiC3bC4dCR1]+[fiC3bC4bCR1]+[fiC3bC4bC4BP]}\cdots\\ \frac{1}{+[fC3dgC4bCR1]+[fC3dgC4bC4BP]+[fC3bC3bH]+[fC3bC3bHL]+[fC3bC3bCR1]+[fC3biC3bH]+[fC3biC3bHL]+[fC3biC3bCR1]}\cdots\\ \frac{1}{+[fiC3biC3bCR1]+[fiC3bC3dgCR1]+[hC3bH]+[hC3bHL]+[hC3bCR1]+[hiC3bCR1]+[hC3bC4bH]+[hC3bC4bHL]+[hC3bC4bCR1]}\cdots\\ \frac{1}{+[hC3bC4bC4BP]+[hC3bC4dH]+[hC3bC4dHL]+[hC3bC4dCR1]+[hiC3bC4dCR1]+[hiC3bC4bCR1]+[hiC3bC4bC4BP]+[hC3dgC4bCR1]}\cdots\\ \frac{1}{+[hC3dgC4bC4BP]+[hC3bC3bH]+[hC3bC3bHL]+[hC3bC3bCR1]+[hC3biC3bH]+[hC3biC3bHL]+[hC3biC3bCR1]+[hiC3biC3bCR1]}\cdots\\ \frac{1}{+[hiC3bC3dgCR1]+[fC4bCR1]+[nfC4bC4BP]+[fC4bC4BP]+[fC4bC4bCR1]+[fC4bC4bC4BP]+[fC4bC4dCR1]+[fC4bC4dC4BP]+[hC4bCR1]}\cdots\\ \frac{1}{+[hC4bC4BP]+[hC4bC4bCR1]+[hC4bC4bC4BP]+[hC4bC4dCR1]+[hC4bC4dC4BP]}\cdots\end{array}\right)$$

S220

$$\frac{d[hC3bC4bBbH]}{dt}=k^{+}_{C3bH}[hC3bC4bBb][H]-k^{-}_{C3bH}[hC3bC4bBbH]-k^{-}_{C3bBbH_{decay}}[hC3bC4bBbH]$$

S221

$$\frac{d[hC3bC4bBbHL]}{dt}=k^{+}_{C3bHL}[hC3bC4bBb][HL]-k^{-}_{C3bHL}[hC3bC4bBbHL]-k^{-}_{C3bBbHL_{decay}}[hC3bC4bBbHL]$$

S222

$$\frac{d[hC3bC4bBbCR1]}{dt}=k^{+}_{C3bC4bCR1}[hC3bC4bBb][CR1]-k^{-}_{C3bC4bCR1}[hC3bC4bBbCR1]-k^{-}_{C3bBbCR1_{decay}}[hC3bC4bBbCR1]$$

S223

$$\frac{d[hC3bC4bBbDAF]}{dt}=k^{+}_{C3bBbDAF}[hC3bC4bBb][DAF]-k^{-}_{C3bBbDAF}[hC3bC4bBbDAF]-k^{-}_{C3bBbDAF_{decay}}[hC3bC4bBbDAF]$$

S224

$$\frac{d[\mathrm{hC3bC3bH}]}{dt} = k^{+}_{\mathrm{C3bH}}[\mathrm{hC3bC3b}][\mathrm{H}] - k^{-}_{\mathrm{C3bH}}[\mathrm{hC3bC3bH}]$$

$$- \left(\begin{array}{l}
\frac{k^{\mathrm{FI}}_{\mathrm{catC3bH}}[\mathrm{I}][\mathrm{hC3bC3bH}]}{K^{\mathrm{FI}}_{\mathrm{mC3bH}} + [\mathrm{C3(H_2O)H}] + [\mathrm{C3(H_2O)HL}] + [\mathrm{fC3bH}] + [\mathrm{fC3bHL}] + [\mathrm{fC3bCR1}] + [\mathrm{fiC3bCR1}] + [\mathrm{IgGC3bC3bH}] + [\mathrm{IgGC3biC3bH}] + [\mathrm{IgGC3bC3bHL}]} \cdots \\
\frac{1}{+ [\mathrm{IgGC3biC3bHL}] + [\mathrm{IgGC3bC3bCR1}] + [\mathrm{IgGC3biC3bCR1}] + [\mathrm{IgGiC3biC3bCR1}] + [\mathrm{IgGiC3bC3dgCR1}] + [\mathrm{fC3bC4bH}] + [\mathrm{fC3bC4bHL}]} \cdots \\
\frac{1}{+ [\mathrm{fC3bC4bCR1}] + [\mathrm{fC3bC4bC4BP}] + [\mathrm{fC3bC4dH}] + [\mathrm{fC3bC4dHL}] + [\mathrm{fC3bC4dCR1}] + [\mathrm{fiC3bC4dCR1}] + [\mathrm{fiC3bC4bCR1}] + [\mathrm{fiC3bC4bC4BP}]} \cdots \\
\frac{1}{+ [\mathrm{fC3dgC4bCR1}] + [\mathrm{fC3dgC4bC4BP}] + [\mathrm{fC3bC3bH}] + [\mathrm{fC3bC3bHL}] + [\mathrm{fC3bC3bCR1}] + [\mathrm{fC3biC3bH}] + [\mathrm{fC3biC3bHL}] + [\mathrm{fC3biC3bCR1}]} \cdots \\
\frac{1}{+ [\mathrm{fiC3biC3bCR1}] + [\mathrm{fiC3bC3dgCR1}] + [\mathrm{hC3bH}] + [\mathrm{hC3bHL}] + [\mathrm{hC3bCR1}] + [\mathrm{hiC3bCR1}] + [\mathrm{hC3bC4bH}] + [\mathrm{hC3bC4bHL}] + [\mathrm{hC3bC4bCR1}]} \cdots \\
\frac{1}{+ [\mathrm{hC3bC4bC4BP}] + [\mathrm{hC3bC4dH}] + [\mathrm{hC3bC4dHL}] + [\mathrm{hC3bC4dCR1}] + [\mathrm{hiC3bC4dCR1}] + [\mathrm{hiC3bC4bCR1}] + [\mathrm{hiC3bC4bC4BP}] + [\mathrm{hC3dgC4bCR1}]} \cdots \\
\frac{1}{+ [\mathrm{hC3dgC4bC4BP}] + [\mathrm{hC3bC3bH}] + [\mathrm{hC3bC3bHL}] + [\mathrm{hC3bC3bCR1}] + [\mathrm{hC3biC3bH}] + [\mathrm{hC3biC3bHL}] + [\mathrm{hC3biC3bCR1}] + [\mathrm{hiC3biC3bCR1}]} \cdots \\
\frac{1}{+ [\mathrm{hiC3bC3dgCR1}] + [\mathrm{fC4bCR1}] + [\mathrm{nfC4bC4BP}] + [\mathrm{fC4bC4BP}] + [\mathrm{fC4bC4bCR1}] + [\mathrm{fC4bC4bC4BP}] + [\mathrm{fC4bC4dCR1}] + [\mathrm{fC4bC4dC4BP}] + [\mathrm{hC4bCR1}]} \cdots \\
\frac{1}{+ [\mathrm{hC4bC4BP}] + [\mathrm{hC4bC4bCR1}] + [\mathrm{hC4bC4bC4BP}] + [\mathrm{hC4bC4dCR1}] + [\mathrm{hC4bC4dC4BP}]} \cdots
\end{array} \right)$$

S225

$$\frac{d[\mathrm{hC3bC3bHL}]}{dt} = k^{+}_{\mathrm{C3bHL}}[\mathrm{hC3bC3b}][\mathrm{HL}] - k^{-}_{\mathrm{C3bHL}}[\mathrm{hC3bC3bHL}]$$

$$- \left(\begin{array}{l}
\frac{k^{\mathrm{FI}}_{\mathrm{catC3bH}}[\mathrm{I}][\mathrm{hC3bC3bHL}]}{K^{\mathrm{FI}}_{\mathrm{mC3bH}} + [\mathrm{C3(H_2O)H}] + [\mathrm{C3(H_2O)HL}] + [\mathrm{fC3bH}] + [\mathrm{fC3bHL}] + [\mathrm{fC3bCR1}] + [\mathrm{fiC3bCR1}] + [\mathrm{IgGC3bC3bH}] + [\mathrm{IgGC3biC3bH}] + [\mathrm{IgGC3bC3bHL}]} \cdots \\
\frac{1}{+ [\mathrm{IgGC3biC3bHL}] + [\mathrm{IgGC3bC3bCR1}] + [\mathrm{IgGC3biC3bCR1}] + [\mathrm{IgGiC3biC3bCR1}] + [\mathrm{IgGiC3bC3dgCR1}] + [\mathrm{fC3bC4bH}] + [\mathrm{fC3bC4bHL}]} \cdots \\
\frac{1}{+ [\mathrm{fC3bC4bCR1}] + [\mathrm{fC3bC4bC4BP}] + [\mathrm{fC3bC4dH}] + [\mathrm{fC3bC4dHL}] + [\mathrm{fC3bC4dCR1}] + [\mathrm{fiC3bC4dCR1}] + [\mathrm{fiC3bC4bCR1}] + [\mathrm{fiC3bC4bC4BP}]} \cdots \\
\frac{1}{+ [\mathrm{fC3dgC4bCR1}] + [\mathrm{fC3dgC4bC4BP}] + [\mathrm{fC3bC3bH}] + [\mathrm{fC3bC3bHL}] + [\mathrm{fC3bC3bCR1}] + [\mathrm{fC3biC3bH}] + [\mathrm{fC3biC3bHL}] + [\mathrm{fC3biC3bCR1}]} \cdots \\
\frac{1}{+ [\mathrm{fiC3biC3bCR1}] + [\mathrm{fiC3bC3dgCR1}] + [\mathrm{hC3bH}] + [\mathrm{hC3bHL}] + [\mathrm{hC3bCR1}] + [\mathrm{hiC3bCR1}] + [\mathrm{hC3bC4bH}] + [\mathrm{hC3bC4bHL}] + [\mathrm{hC3bC4bCR1}]} \cdots \\
\frac{1}{+ [\mathrm{hC3bC4bC4BP}] + [\mathrm{hC3bC4dH}] + [\mathrm{hC3bC4dHL}] + [\mathrm{hC3bC4dCR1}] + [\mathrm{hiC3bC4dCR1}] + [\mathrm{hiC3bC4bCR1}] + [\mathrm{hiC3bC4bC4BP}] + [\mathrm{hC3dgC4bCR1}]} \cdots \\
\frac{1}{+ [\mathrm{hC3dgC4bC4BP}] + [\mathrm{hC3bC3bH}] + [\mathrm{hC3bC3bHL}] + [\mathrm{hC3bC3bCR1}] + [\mathrm{hC3biC3bH}] + [\mathrm{hC3biC3bHL}] + [\mathrm{hC3biC3bCR1}] + [\mathrm{hiC3biC3bCR1}]} \cdots \\
\frac{1}{+ [\mathrm{hiC3bC3dgCR1}] + [\mathrm{fC4bCR1}] + [\mathrm{nfC4bC4BP}] + [\mathrm{fC4bC4BP}] + [\mathrm{fC4bC4bCR1}] + [\mathrm{fC4bC4bC4BP}] + [\mathrm{fC4bC4dCR1}] + [\mathrm{fC4bC4dC4BP}] + [\mathrm{hC4bCR1}]} \cdots \\
\frac{1}{+ [\mathrm{hC4bC4BP}] + [\mathrm{hC4bC4bCR1}] + [\mathrm{hC4bC4bC4BP}] + [\mathrm{hC4bC4dCR1}] + [\mathrm{hC4bC4dC4BP}]} \cdots
\end{array} \right)$$

S226

$$\frac{d[\mathrm{hC3bC3bCR1}]}{dt}=k^{+}_{\mathrm{C3bC3bCR1}}[\mathrm{hC3bC3b}][\mathrm{CR1}]-k^{-}_{\mathrm{C3bC3bCR1}}[\mathrm{hC3bC3bCR1}]$$

$$-\left(\begin{array}{l}\frac{k^{\mathrm{FI}}_{\mathrm{catC3bH}}[\mathrm{I}][\mathrm{hC3bC3bCR1}]}{K^{\mathrm{FI}}_{\mathrm{mC3bH}}+[\mathrm{C3(H_2O)H}]+[\mathrm{C3(H_2O)HL}]+[\mathrm{fC3bH}]+[\mathrm{fC3bHL}]+[\mathrm{fC3bCR1}]+[\mathrm{fiC3bCR1}]+[\mathrm{IgGC3bC3bH}]+[\mathrm{IgGC3biC3bH}]+[\mathrm{IgGC3bC3bHL}]}\cdots \\ \frac{1}{+[\mathrm{IgGC3biC3bHL}]+[\mathrm{IgGC3bC3bCR1}]+[\mathrm{IgGC3biC3bCR1}]+[\mathrm{IgGiC3biC3bCR1}]+[\mathrm{IgGiC3bC3dgCR1}]+[\mathrm{fC3bC4bH}]+[\mathrm{fC3bC4bHL}]}\cdots \\ \frac{1}{+[\mathrm{fC3bC4bCR1}]+[\mathrm{fC3bC4bC4BP}]+[\mathrm{fC3bC4dH}]+[\mathrm{fC3bC4dHL}]+[\mathrm{fC3bC4dCR1}]+[\mathrm{fiC3bC4dCR1}]+[\mathrm{fiC3bC4bCR1}]+[\mathrm{fiC3bC4bC4BP}]}\cdots \\ \frac{1}{+[\mathrm{fC3dgC4bCR1}]+[\mathrm{fC3dgC4bC4BP}]+[\mathrm{fC3bC3bH}]+[\mathrm{fC3bC3bHL}]+[\mathrm{fC3bC3bCR1}]+[\mathrm{fC3biC3bH}]+[\mathrm{fC3biC3bHL}]+[\mathrm{fC3biC3bCR1}]}\cdots \\ \frac{1}{+[\mathrm{fiC3biC3bCR1}]+[\mathrm{fiC3bC3dgCR1}]+[\mathrm{hC3bH}]+[\mathrm{hC3bHL}]+[\mathrm{hC3bCR1}]+[\mathrm{hiC3bCR1}]+[\mathrm{hC3bC4bH}]+[\mathrm{hC3bC4bHL}]+[\mathrm{hC3bC4bCR1}]}\cdots \\ \frac{1}{+[\mathrm{hC3bC4bC4BP}]+[\mathrm{hC3bC4dH}]+[\mathrm{hC3bC4dHL}]+[\mathrm{hC3bC4dCR1}]+[\mathrm{hiC3bC4dCR1}]+[\mathrm{hiC3bC4bCR1}]+[\mathrm{hiC3bC4bC4BP}]+[\mathrm{hC3dgC4bCR1}]}\cdots \\ \frac{1}{+[\mathrm{hC3dgC4bC4BP}]+[\mathrm{hC3bC3bH}]+[\mathrm{hC3bC3bHL}]+[\mathrm{hC3bC3bCR1}]+[\mathrm{hC3biC3bH}]+[\mathrm{hC3biC3bHL}]+[\mathrm{hC3biC3bCR1}]+[\mathrm{hiC3biC3bCR1}]}\cdots \\ \frac{1}{+[\mathrm{hiC3bC3dgCR1}]+[\mathrm{fC4bCR1}]+[\mathrm{nfC4bC4BP}]+[\mathrm{fC4bC4BP}]+[\mathrm{fC4bC4bCR1}]+[\mathrm{fC4bC4bC4BP}]+[\mathrm{fC4bC4dCR1}]+[\mathrm{fC4bC4dC4BP}]+[\mathrm{hC4bCR1}]}\cdots \\ \frac{1}{+[\mathrm{hC4bC4BP}]+[\mathrm{hC4bC4bCR1}]+[\mathrm{hC4bC4bC4BP}]+[\mathrm{hC4bC4dCR1}]+[\mathrm{hC4bC4dC4BP}]}\cdots\end{array}\right)$$

S227

$$\frac{d[\mathrm{hC3biC3b}]}{dt}=$$

$$\left(\begin{array}{l}\frac{k^{\mathrm{FI}}_{\mathrm{catC3bH}}[\mathrm{I}][\mathrm{hC3bC3bH}]}{K^{\mathrm{FI}}_{\mathrm{mC3bH}}+[\mathrm{C3(H_2O)H}]+[\mathrm{C3(H_2O)HL}]+[\mathrm{fC3bH}]+[\mathrm{fC3bHL}]+[\mathrm{fC3bCR1}]+[\mathrm{fiC3bCR1}]+[\mathrm{IgGC3bC3bH}]+[\mathrm{IgGC3biC3bH}]+[\mathrm{IgGC3bC3bHL}]}\cdots \\ \frac{1}{+[\mathrm{IgGC3biC3bHL}]+[\mathrm{IgGC3bC3bCR1}]+[\mathrm{IgGC3biC3bCR1}]+[\mathrm{IgGiC3biC3bCR1}]+[\mathrm{IgGiC3bC3dgCR1}]+[\mathrm{fC3bC4bH}]+[\mathrm{fC3bC4bHL}]}\cdots \\ \frac{1}{+[\mathrm{fC3bC4bCR1}]+[\mathrm{fC3bC4bC4BP}]+[\mathrm{fC3bC4dH}]+[\mathrm{fC3bC4dHL}]+[\mathrm{fC3bC4dCR1}]+[\mathrm{fiC3bC4dCR1}]+[\mathrm{fiC3bC4bCR1}]+[\mathrm{fiC3bC4bC4BP}]}\cdots \\ \frac{1}{+[\mathrm{fC3dgC4bCR1}]+[\mathrm{fC3dgC4bC4BP}]+[\mathrm{fC3bC3bH}]+[\mathrm{fC3bC3bHL}]+[\mathrm{fC3bC3bCR1}]+[\mathrm{fC3biC3bH}]+[\mathrm{fC3biC3bHL}]+[\mathrm{fC3biC3bCR1}]}\cdots \\ \frac{1}{+[\mathrm{fiC3biC3bCR1}]+[\mathrm{fiC3bC3dgCR1}]+[\mathrm{hC3bH}]+[\mathrm{hC3bHL}]+[\mathrm{hC3bCR1}]+[\mathrm{hiC3bCR1}]+[\mathrm{hC3bC4bH}]+[\mathrm{hC3bC4bHL}]+[\mathrm{hC3bC4bCR1}]}\cdots \\ \frac{1}{+[\mathrm{hC3bC4bC4BP}]+[\mathrm{hC3bC4dH}]+[\mathrm{hC3bC4dHL}]+[\mathrm{hC3bC4dCR1}]+[\mathrm{hiC3bC4dCR1}]+[\mathrm{hiC3bC4bCR1}]+[\mathrm{hiC3bC4bC4BP}]+[\mathrm{hC3dgC4bCR1}]}\cdots \\ \frac{1}{+[\mathrm{hC3dgC4bC4BP}]+[\mathrm{hC3bC3bH}]+[\mathrm{hC3bC3bHL}]+[\mathrm{hC3bC3bCR1}]+[\mathrm{hC3biC3bH}]+[\mathrm{hC3biC3bHL}]+[\mathrm{hC3biC3bCR1}]+[\mathrm{hiC3biC3bCR1}]}\cdots \\ \frac{1}{+[\mathrm{hiC3bC3dgCR1}]+[\mathrm{fC4bCR1}]+[\mathrm{nfC4bC4BP}]+[\mathrm{fC4bC4BP}]+[\mathrm{fC4bC4bCR1}]+[\mathrm{fC4bC4bC4BP}]+[\mathrm{fC4bC4dCR1}]+[\mathrm{fC4bC4dC4BP}]+[\mathrm{hC4bCR1}]}\cdots \\ \frac{1}{+[\mathrm{hC4bC4BP}]+[\mathrm{hC4bC4bCR1}]+[\mathrm{hC4bC4bC4BP}]+[\mathrm{hC4bC4dCR1}]+[\mathrm{hC4bC4dC4BP}]}\cdots\end{array}\right)$$

$$+\left(\begin{array}{l}
\frac{k_{\mathrm{catC3bH}}^{\mathrm{FI}}[\mathrm{I}][\mathrm{hC3bC3bHL}]}{K_{\mathrm{mC3bH}}^{\mathrm{FI}}+[\mathrm{C3(H_2O)H}]+[\mathrm{C3(H_2O)HL}]+[\mathrm{fC3bH}]+[\mathrm{fC3bHL}]+[\mathrm{fC3bCR1}]+[\mathrm{fiC3bCR1}]+[\mathrm{IgGC3bC3bH}]+[\mathrm{IgGC3biC3bH}]+[\mathrm{IgGC3bC3bHL}]}\cdots \\
\frac{1}{+[\mathrm{IgGC3biC3bHL}]+[\mathrm{IgGC3bC3bCR1}]+[\mathrm{IgGC3biC3bCR1}]+[\mathrm{IgGiC3biC3bCR1}]+[\mathrm{IgGiC3bC3dgCR1}]+[\mathrm{fC3bC4bH}]+[\mathrm{fC3bC4bHL}]}\cdots \\
\frac{1}{+[\mathrm{fC3bC4bCR1}]+[\mathrm{fC3bC4bC4BP}]+[\mathrm{fC3bC4dH}]+[\mathrm{fC3bC4dHL}]+[\mathrm{fC3bC4dCR1}]+[\mathrm{fiC3bC4dCR1}]+[\mathrm{fiC3bC4bCR1}]+[\mathrm{fiC3bC4bC4BP}]}\cdots \\
\frac{1}{+[\mathrm{fC3dgC4bCR1}]+[\mathrm{fC3dgC4bC4BP}]+[\mathrm{fC3bC3bH}]+[\mathrm{fC3bC3bHL}]+[\mathrm{fC3bC3bCR1}]+[\mathrm{fC3biC3bH}]+[\mathrm{fC3biC3bHL}]+[\mathrm{fC3biC3bCR1}]}\cdots \\
\frac{1}{+[\mathrm{fiC3biC3bCR1}]+[\mathrm{fiC3bC3dgCR1}]+[\mathrm{hC3bH}]+[\mathrm{hC3bHL}]+[\mathrm{hC3bCR1}]+[\mathrm{hiC3bCR1}]+[\mathrm{hC3bC4bH}]+[\mathrm{hC3bC4bHL}]+[\mathrm{hC3bC4bCR1}]}\cdots \\
\frac{1}{+[\mathrm{hC3bC4bC4BP}]+[\mathrm{hC3bC4dH}]+[\mathrm{hC3bC4dHL}]+[\mathrm{hC3bC4dCR1}]+[\mathrm{hiC3bC4dCR1}]+[\mathrm{hiC3bC4bCR1}]+[\mathrm{hiC3bC4bC4BP}]+[\mathrm{hC3dgC4bCR1}]}\cdots \\
\frac{1}{+[\mathrm{hC3dgC4bC4BP}]+[\mathrm{hC3bC3bH}]+[\mathrm{hC3bC3bHL}]+[\mathrm{hC3bC3bCR1}]+[\mathrm{hC3biC3bH}]+[\mathrm{hC3biC3bHL}]+[\mathrm{hC3biC3bCR1}]+[\mathrm{hiC3biC3bCR1}]}\cdots \\
\frac{1}{+[\mathrm{hiC3bC3dgCR1}]+[\mathrm{fC4bCR1}]+[\mathrm{nfC4bC4BP}]+[\mathrm{fC4bC4BP}]+[\mathrm{fC4bC4bCR1}]+[\mathrm{fC4bC4bC4BP}]+[\mathrm{fC4bC4dCR1}]+[\mathrm{fC4bC4dC4BP}]+[\mathrm{hC4bCR1}]}\cdots \\
\frac{1}{+[\mathrm{hC4bC4BP}]+[\mathrm{hC4bC4bCR1}]+[\mathrm{hC4bC4bC4BP}]+[\mathrm{hC4bC4dCR1}]+[\mathrm{hC4bC4dC4BP}]}\cdots
\end{array}\right)$$

$$+\left(\begin{array}{l}
\frac{k_{\mathrm{catC3bH}}^{\mathrm{FI}}[\mathrm{I}][\mathrm{hC3bC3bCR1}]}{K_{\mathrm{mC3bH}}^{\mathrm{FI}}+[\mathrm{C3(H_2O)H}]+[\mathrm{C3(H_2O)HL}]+[\mathrm{fC3bH}]+[\mathrm{fC3bHL}]+[\mathrm{fC3bCR1}]+[\mathrm{fiC3bCR1}]+[\mathrm{IgGC3bC3bH}]+[\mathrm{IgGC3biC3bH}]+[\mathrm{IgGC3bC3bHL}]}\cdots \\
\frac{1}{+[\mathrm{IgGC3biC3bHL}]+[\mathrm{IgGC3bC3bCR1}]+[\mathrm{IgGC3biC3bCR1}]+[\mathrm{IgGiC3biC3bCR1}]+[\mathrm{IgGiC3bC3dgCR1}]+[\mathrm{fC3bC4bH}]+[\mathrm{fC3bC4bHL}]}\cdots \\
\frac{1}{+[\mathrm{fC3bC4bCR1}]+[\mathrm{fC3bC4bC4BP}]+[\mathrm{fC3bC4dH}]+[\mathrm{fC3bC4dHL}]+[\mathrm{fC3bC4dCR1}]+[\mathrm{fiC3bC4dCR1}]+[\mathrm{fiC3bC4bCR1}]+[\mathrm{fiC3bC4bC4BP}]}\cdots \\
\frac{1}{+[\mathrm{fC3dgC4bCR1}]+[\mathrm{fC3dgC4bC4BP}]+[\mathrm{fC3bC3bH}]+[\mathrm{fC3bC3bHL}]+[\mathrm{fC3bC3bCR1}]+[\mathrm{fC3biC3bH}]+[\mathrm{fC3biC3bHL}]+[\mathrm{fC3biC3bCR1}]}\cdots \\
\frac{1}{+[\mathrm{fiC3biC3bCR1}]+[\mathrm{fiC3bC3dgCR1}]+[\mathrm{hC3bH}]+[\mathrm{hC3bHL}]+[\mathrm{hC3bCR1}]+[\mathrm{hiC3bCR1}]+[\mathrm{hC3bC4bH}]+[\mathrm{hC3bC4bHL}]+[\mathrm{hC3bC4bCR1}]}\cdots \\
\frac{1}{+[\mathrm{hC3bC4bC4BP}]+[\mathrm{hC3bC4dH}]+[\mathrm{hC3bC4dHL}]+[\mathrm{hC3bC4dCR1}]+[\mathrm{hiC3bC4dCR1}]+[\mathrm{hiC3bC4bCR1}]+[\mathrm{hiC3bC4bC4BP}]+[\mathrm{hC3dgC4bCR1}]}\cdots \\
\frac{1}{+[\mathrm{hC3dgC4bC4BP}]+[\mathrm{hC3bC3bH}]+[\mathrm{hC3bC3bHL}]+[\mathrm{hC3bC3bCR1}]+[\mathrm{hC3biC3bH}]+[\mathrm{hC3biC3bHL}]+[\mathrm{hC3biC3bCR1}]+[\mathrm{hiC3biC3bCR1}]}\cdots \\
\frac{1}{+[\mathrm{hiC3bC3dgCR1}]+[\mathrm{fC4bCR1}]+[\mathrm{nfC4bC4BP}]+[\mathrm{fC4bC4BP}]+[\mathrm{fC4bC4bCR1}]+[\mathrm{fC4bC4bC4BP}]+[\mathrm{fC4bC4dCR1}]+[\mathrm{fC4bC4dC4BP}]+[\mathrm{hC4bCR1}]}\cdots \\
\frac{1}{+[\mathrm{hC4bC4BP}]+[\mathrm{hC4bC4bCR1}]+[\mathrm{hC4bC4bC4BP}]+[\mathrm{hC4bC4dCR1}]+[\mathrm{hC4bC4dC4BP}]}\cdots
\end{array}\right)$$

$$-k_{\mathrm{C3bH}}^{+}[\mathrm{hC3biC3b}][\mathrm{H}]+k_{\mathrm{C3bH}}^{-}[\mathrm{hC3biC3bH}]-k_{\mathrm{C3bHL}}^{+}[\mathrm{hC3biC3b}][\mathrm{HL}]+k_{\mathrm{C3bHL}}^{-}[\mathrm{hC3biC3bHL}]-k_{\mathrm{C3biC3bCR1}}^{+}[\mathrm{hC3biC3b}][\mathrm{CR1}]$$

$$+k_{\mathrm{C3biC3bCR1}}^{-}[\mathrm{hC3biC3bCR1}]$$

S228

$$\frac{d[\text{hC3biC3bH}]}{dt} = k^{+}_{\text{C3bH}}[\text{hC3biC3b}][\text{H}] - k^{-}_{\text{C3bH}}[\text{hC3biC3bH}]$$

$$-\left(\frac{k^{\text{FI}}_{\text{catC3bH}}[\text{I}][\text{hC3biC3bH}]}{K^{\text{FI}}_{\text{mC3bH}} + [\text{C3(H}_2\text{O)H}] + [\text{C3(H}_2\text{O)HL}] + [\text{fC3bH}] + [\text{fC3bHL}] + [\text{fC3bCR1}] + [\text{fiC3bCR1}] + [\text{IgGC3bC3bH}] + [\text{IgGC3biC3bH}] + [\text{IgGC3bC3bHL}]} \cdots \right.$$

$$\frac{1}{+[\text{IgGC3biC3bHL}] + [\text{IgGC3bC3bCR1}] + [\text{IgGC3biC3bCR1}] + [\text{IgGiC3biC3bCR1}] + [\text{IgGiC3bC3dgCR1}] + [\text{fC3bC4bH}] + [\text{fC3bC4bHL}]} \cdots$$

$$\frac{1}{+[\text{fC3bC4bCR1}] + [\text{fC3bC4bC4BP}] + [\text{fC3bC4dH}] + [\text{fC3bC4dHL}] + [\text{fC3bC4dCR1}] + [\text{fiC3bC4dCR1}] + [\text{fiC3bC4bCR1}] + [\text{fiC3bC4bC4BP}]} \cdots$$

$$\frac{1}{+[\text{fC3dgC4bCR1}] + [\text{fC3dgC4bC4BP}] + [\text{fC3bC3bH}] + [\text{fC3bC3bHL}] + [\text{fC3bC3bCR1}] + [\text{fC3biC3bH}] + [\text{fC3biC3bHL}] + [\text{fC3biC3bCR1}]} \cdots$$

$$\frac{1}{+[\text{fiC3biC3bCR1}] + [\text{fiC3bC3dgCR1}] + [\text{hC3bH}] + [\text{hC3bHL}] + [\text{hC3bCR1}] + [\text{hiC3bCR1}] + [\text{hC3bC4bH}] + [\text{hC3bC4bHL}] + [\text{hC3bC4bCR1}]} \cdots$$

$$\frac{1}{+[\text{hC3bC4bC4BP}] + [\text{hC3bC4dH}] + [\text{hC3bC4dHL}] + [\text{hC3bC4dCR1}] + [\text{hiC3bC4dCR1}] + [\text{hiC3bC4bCR1}] + [\text{hiC3bC4bC4BP}] + [\text{hC3dgC4bCR1}]} \cdots$$

$$\frac{1}{+[\text{hC3dgC4bC4BP}] + [\text{hC3bC3bH}] + [\text{hC3bC3bHL}] + [\text{hC3bC3bCR1}] + [\text{hC3biC3bH}] + [\text{hC3biC3bHL}] + [\text{hC3biC3bCR1}] + [\text{hiC3biC3bCR1}]} \cdots$$

$$\frac{1}{+[\text{hiC3bC3dgCR1}] + [\text{fC4bCR1}] + [\text{nfC4bC4BP}] + [\text{fC4bC4BP}] + [\text{fC4bC4bCR1}] + [\text{fC4bC4bC4BP}] + [\text{fC4bC4dCR1}] + [\text{fC4bC4dC4BP}] + [\text{hC4bCR1}]} \cdots$$

$$\left. \frac{1}{+[\text{hC4bC4BP}] + [\text{hC4bC4bCR1}] + [\text{hC4bC4bC4BP}] + [\text{hC4bC4dCR1}] + [\text{hC4bC4dC4BP}]} \cdots \right)$$

S229

$$\frac{d[\text{hC3biC3bHL}]}{dt} = k^{+}_{\text{C3bHL}}[\text{hC3biC3b}][\text{HL}] - k^{-}_{\text{C3bHL}}[\text{hC3biC3bHL}]$$

$$-\left(\frac{k^{\text{FI}}_{\text{catC3bH}}[\text{I}][\text{hC3biC3bHL}]}{K^{\text{FI}}_{\text{mC3bH}} + [\text{C3(H}_2\text{O)H}] + [\text{C3(H}_2\text{O)HL}] + [\text{fC3bH}] + [\text{fC3bHL}] + [\text{fC3bCR1}] + [\text{fiC3bCR1}] + [\text{IgGC3bC3bH}] + [\text{IgGC3biC3bH}] + [\text{IgGC3bC3bHL}]} \cdots \right.$$

$$\frac{1}{+[\text{IgGC3biC3bHL}] + [\text{IgGC3bC3bCR1}] + [\text{IgGC3biC3bCR1}] + [\text{IgGiC3biC3bCR1}] + [\text{IgGiC3bC3dgCR1}] + [\text{fC3bC4bH}] + [\text{fC3bC4bHL}]} \cdots$$

$$\frac{1}{+[\text{fC3bC4bCR1}] + [\text{fC3bC4bC4BP}] + [\text{fC3bC4dH}] + [\text{fC3bC4dHL}] + [\text{fC3bC4dCR1}] + [\text{fiC3bC4dCR1}] + [\text{fiC3bC4bCR1}] + [\text{fiC3bC4bC4BP}]} \cdots$$

$$\frac{1}{+[\text{fC3dgC4bCR1}] + [\text{fC3dgC4bC4BP}] + [\text{fC3bC3bH}] + [\text{fC3bC3bHL}] + [\text{fC3bC3bCR1}] + [\text{fC3biC3bH}] + [\text{fC3biC3bHL}] + [\text{fC3biC3bCR1}]} \cdots$$

$$\frac{1}{+[\text{fiC3biC3bCR1}] + [\text{fiC3bC3dgCR1}] + [\text{hC3bH}] + [\text{hC3bHL}] + [\text{hC3bCR1}] + [\text{hiC3bCR1}] + [\text{hC3bC4bH}] + [\text{hC3bC4bHL}] + [\text{hC3bC4bCR1}]} \cdots$$

$$\frac{1}{+[\text{hC3bC4bC4BP}] + [\text{hC3bC4dH}] + [\text{hC3bC4dHL}] + [\text{hC3bC4dCR1}] + [\text{hiC3bC4dCR1}] + [\text{hiC3bC4bCR1}] + [\text{hiC3bC4bC4BP}] + [\text{hC3dgC4bCR1}]} \cdots$$

$$\frac{1}{+[\text{hC3dgC4bC4BP}] + [\text{hC3bC3bH}] + [\text{hC3bC3bHL}] + [\text{hC3bC3bCR1}] + [\text{hC3biC3bH}] + [\text{hC3biC3bHL}] + [\text{hC3biC3bCR1}] + [\text{hiC3biC3bCR1}]} \cdots$$

$$\frac{1}{+[\text{hiC3bC3dgCR1}] + [\text{fC4bCR1}] + [\text{nfC4bC4BP}] + [\text{fC4bC4BP}] + [\text{fC4bC4bCR1}] + [\text{fC4bC4bC4BP}] + [\text{fC4bC4dCR1}] + [\text{fC4bC4dC4BP}] + [\text{hC4bCR1}]} \cdots$$

$$\left. \frac{1}{+[\text{hC4bC4BP}] + [\text{hC4bC4bCR1}] + [\text{hC4bC4bC4BP}] + [\text{hC4bC4dCR1}] + [\text{hC4bC4dC4BP}]} \cdots \right)$$

S230

$$\frac{d[\mathrm{hC3biC3bCR1}]}{dt} = k^{+}_{\mathrm{C3biC3bCR1}}[\mathrm{hC3biC3b}][\mathrm{CR1}] - k^{-}_{\mathrm{C3biC3bCR1}}[\mathrm{hC3biC3bCR1}]$$

$$-\left(\begin{array}{l}
\frac{k^{\mathrm{FI}}_{\mathrm{catC3bH}}[\mathrm{I}][\mathrm{hC3biC3bCR1}]}{K^{\mathrm{FI}}_{\mathrm{mC3bH}} + [\mathrm{C3(H_2O)H}] + [\mathrm{C3(H_2O)HL}] + [\mathrm{fC3bH}] + [\mathrm{fC3bHL}] + [\mathrm{fC3bCR1}] + [\mathrm{fiC3bCR1}] + [\mathrm{IgGC3bC3bH}] + [\mathrm{IgGC3biC3bH}] + [\mathrm{IgGC3bC3bHL}]}\cdots \\
\frac{1}{+ [\mathrm{IgGC3biC3bHL}] + [\mathrm{IgGC3bC3bCR1}] + [\mathrm{IgGC3biC3bCR1}] + [\mathrm{IgGiC3biC3bCR1}] + [\mathrm{IgGiC3bC3dgCR1}] + [\mathrm{fC3bC4bH}] + [\mathrm{fC3bC4bHL}]}\cdots \\
\frac{1}{+ [\mathrm{fC3bC4bCR1}] + [\mathrm{fC3bC4bC4BP}] + [\mathrm{fC3bC4dH}] + [\mathrm{fC3bC4dHL}] + [\mathrm{fC3bC4dCR1}] + [\mathrm{fiC3bC4dCR1}] + [\mathrm{fiC3bC4bCR1}] + [\mathrm{fiC3bC4bC4BP}]}\cdots \\
\frac{1}{+ [\mathrm{fC3dgC4bCR1}] + [\mathrm{fC3dgC4bC4BP}] + [\mathrm{fC3bC3bH}] + [\mathrm{fC3bC3bHL}] + [\mathrm{fC3bC3bCR1}] + [\mathrm{fC3biC3bH}] + [\mathrm{fC3biC3bHL}] + [\mathrm{fC3biC3bCR1}]}\cdots \\
\frac{1}{+ [\mathrm{fiC3biC3bCR1}] + [\mathrm{fiC3bC3dgCR1}] + [\mathrm{hC3bH}] + [\mathrm{hC3bHL}] + [\mathrm{hC3bCR1}] + [\mathrm{hiC3bCR1}] + [\mathrm{hC3bC4bH}] + [\mathrm{hC3bC4bHL}] + [\mathrm{hC3bC4bCR1}]}\cdots \\
\frac{1}{+ [\mathrm{hC3bC4bC4BP}] + [\mathrm{hC3bC4dH}] + [\mathrm{hC3bC4dHL}] + [\mathrm{hC3bC4dCR1}] + [\mathrm{hiC3bC4dCR1}] + [\mathrm{hiC3bC4bCR1}] + [\mathrm{hiC3bC4bC4BP}] + [\mathrm{hC3dgC4bCR1}]}\cdots \\
\frac{1}{+ [\mathrm{hC3dgC4bC4BP}] + [\mathrm{hC3bC3bH}] + [\mathrm{hC3bC3bHL}] + [\mathrm{hC3bC3bCR1}] + [\mathrm{hC3biC3bH}] + [\mathrm{hC3biC3bHL}] + [\mathrm{hC3biC3bCR1}] + [\mathrm{hiC3biC3bCR1}]}\cdots \\
\frac{1}{+ [\mathrm{hiC3bC3dgCR1}] + [\mathrm{fC4bCR1}] + [\mathrm{nfC4bC4BP}] + [\mathrm{fC4bC4BP}] + [\mathrm{fC4bC4bCR1}] + [\mathrm{fC4bC4bC4BP}] + [\mathrm{fC4bC4dCR1}] + [\mathrm{fC4bC4dC4BP}] + [\mathrm{hC4bCR1}]}\cdots \\
\frac{1}{+ [\mathrm{hC4bC4BP}] + [\mathrm{hC4bC4bCR1}] + [\mathrm{hC4bC4bC4BP}] + [\mathrm{hC4bC4dCR1}] + [\mathrm{hC4bC4dC4BP}]}\cdots
\end{array}\right)$$

S231

$$\frac{d[\mathrm{hiC3biC3b}]}{dt} =$$

$$\left(\begin{array}{l}
\frac{k^{\mathrm{FI}}_{\mathrm{catC3bH}}[\mathrm{I}][\mathrm{hC3biC3bH}]}{K^{\mathrm{FI}}_{\mathrm{mC3bH}} + [\mathrm{C3(H_2O)H}] + [\mathrm{C3(H_2O)HL}] + [\mathrm{fC3bH}] + [\mathrm{fC3bHL}] + [\mathrm{fC3bCR1}] + [\mathrm{fiC3bCR1}] + [\mathrm{IgGC3bC3bH}] + [\mathrm{IgGC3biC3bH}] + [\mathrm{IgGC3bC3bHL}]}\cdots \\
\frac{1}{+ [\mathrm{IgGC3biC3bHL}] + [\mathrm{IgGC3bC3bCR1}] + [\mathrm{IgGC3biC3bCR1}] + [\mathrm{IgGiC3biC3bCR1}] + [\mathrm{IgGiC3bC3dgCR1}] + [\mathrm{fC3bC4bH}] + [\mathrm{fC3bC4bHL}]}\cdots \\
\frac{1}{+ [\mathrm{fC3bC4bCR1}] + [\mathrm{fC3bC4bC4BP}] + [\mathrm{fC3bC4dH}] + [\mathrm{fC3bC4dHL}] + [\mathrm{fC3bC4dCR1}] + [\mathrm{fiC3bC4dCR1}] + [\mathrm{fiC3bC4bCR1}] + [\mathrm{fiC3bC4bC4BP}]}\cdots \\
\frac{1}{+ [\mathrm{fC3dgC4bCR1}] + [\mathrm{fC3dgC4bC4BP}] + [\mathrm{fC3bC3bH}] + [\mathrm{fC3bC3bHL}] + [\mathrm{fC3bC3bCR1}] + [\mathrm{fC3biC3bH}] + [\mathrm{fC3biC3bHL}] + [\mathrm{fC3biC3bCR1}]}\cdots \\
\frac{1}{+ [\mathrm{fiC3biC3bCR1}] + [\mathrm{fiC3bC3dgCR1}] + [\mathrm{hC3bH}] + [\mathrm{hC3bHL}] + [\mathrm{hC3bCR1}] + [\mathrm{hiC3bCR1}] + [\mathrm{hC3bC4bH}] + [\mathrm{hC3bC4bHL}] + [\mathrm{hC3bC4bCR1}]}\cdots \\
\frac{1}{+ [\mathrm{hC3bC4bC4BP}] + [\mathrm{hC3bC4dH}] + [\mathrm{hC3bC4dHL}] + [\mathrm{hC3bC4dCR1}] + [\mathrm{hiC3bC4dCR1}] + [\mathrm{hiC3bC4bCR1}] + [\mathrm{hiC3bC4bC4BP}] + [\mathrm{hC3dgC4bCR1}]}\cdots \\
\frac{1}{+ [\mathrm{hC3dgC4bC4BP}] + [\mathrm{hC3bC3bH}] + [\mathrm{hC3bC3bHL}] + [\mathrm{hC3bC3bCR1}] + [\mathrm{hC3biC3bH}] + [\mathrm{hC3biC3bHL}] + [\mathrm{hC3biC3bCR1}] + [\mathrm{hiC3biC3bCR1}]}\cdots \\
\frac{1}{+ [\mathrm{hiC3bC3dgCR1}] + [\mathrm{fC4bCR1}] + [\mathrm{nfC4bC4BP}] + [\mathrm{fC4bC4BP}] + [\mathrm{fC4bC4bCR1}] + [\mathrm{fC4bC4bC4BP}] + [\mathrm{fC4bC4dCR1}] + [\mathrm{fC4bC4dC4BP}] + [\mathrm{hC4bCR1}]}\cdots \\
\frac{1}{+ [\mathrm{hC4bC4BP}] + [\mathrm{hC4bC4bCR1}] + [\mathrm{hC4bC4bC4BP}] + [\mathrm{hC4bC4dCR1}] + [\mathrm{hC4bC4dC4BP}]}\cdots
\end{array}\right)$$

$$+\left(\begin{array}{l}\frac{k_{\text{catC3bH}}^{\text{FI}}[\text{I}][\text{hC3biC3bHL}]}{K_{\text{mC3bH}}^{\text{FI}}+[\text{C3(H}_2\text{O)H}]+[\text{C3(H}_2\text{O)HL}]+[\text{fC3bH}]+[\text{fC3bHL}]+[\text{fC3bCR1}]+[\text{fiC3bCR1}]+[\text{IgGC3bC3bH}]+[\text{IgGC3biC3bH}]+[\text{IgGC3bC3bHL}]}\cdots \\ \frac{1}{+[\text{IgGC3biC3bHL}]+[\text{IgGC3bC3bCR1}]+[\text{IgGC3biC3bCR1}]+[\text{IgGiC3biC3bCR1}]+[\text{IgGiC3bC3dgCR1}]+[\text{fC3bC4bH}]+[\text{fC3bC4bHL}]}\cdots \\ \frac{1}{+[\text{fC3bC4bCR1}]+[\text{fC3bC4bC4BP}]+[\text{fC3bC4dH}]+[\text{fC3bC4dHL}]+[\text{fC3bC4dCR1}]+[\text{fiC3bC4dCR1}]+[\text{fiC3bC4bCR1}]+[\text{fiC3bC4bC4BP}]}\cdots \\ \frac{1}{+[\text{fC3dgC4bCR1}]+[\text{fC3dgC4bC4BP}]+[\text{fC3bC3bH}]+[\text{fC3bC3bHL}]+[\text{fC3bC3bCR1}]+[\text{fC3biC3bH}]+[\text{fC3biC3bHL}]+[\text{fC3biC3bCR1}]}\cdots \\ \frac{1}{+[\text{fiC3biC3bCR1}]+[\text{fiC3bC3dgCR1}]+[\text{hC3bH}]+[\text{hC3bHL}]+[\text{hC3bCR1}]+[\text{hiC3bCR1}]+[\text{hC3bC4bH}]+[\text{hC3bC4bHL}]+[\text{hC3bC4bCR1}]}\cdots \\ \frac{1}{+[\text{hC3bC4bC4BP}]+[\text{hC3bC4dH}]+[\text{hC3bC4dHL}]+[\text{hC3bC4dCR1}]+[\text{hiC3bC4dCR1}]+[\text{hiC3bC4bCR1}]+[\text{hiC3bC4bC4BP}]+[\text{hC3dgC4bCR1}]}\cdots \\ \frac{1}{+[\text{hC3dgC4bC4BP}]+[\text{hC3bC3bH}]+[\text{hC3bC3bHL}]+[\text{hC3bC3bCR1}]+[\text{hC3biC3bH}]+[\text{hC3biC3bHL}]+[\text{hC3biC3bCR1}]+[\text{hiC3biC3bCR1}]}\cdots \\ \frac{1}{+[\text{hiC3bC3dgCR1}]+[\text{fC4bCR1}]+[\text{nfC4bC4BP}]+[\text{fC4bC4BP}]+[\text{fC4bC4bCR1}]+[\text{fC4bC4bC4BP}]+[\text{fC4bC4dCR1}]+[\text{fC4bC4dC4BP}]+[\text{hC4bCR1}]}\cdots \\ \frac{1}{+[\text{hC4bC4BP}]+[\text{hC4bC4bCR1}]+[\text{hC4bC4bC4BP}]+[\text{hC4bC4dCR1}]+[\text{hC4bC4dC4BP}]}\cdots\end{array}\right)$$

$$+\left(\begin{array}{l}\frac{k_{\text{catC3bH}}^{\text{FI}}[\text{I}][\text{hC3biC3bCR1}]}{K_{\text{mC3bH}}^{\text{FI}}+[\text{C3(H}_2\text{O)H}]+[\text{C3(H}_2\text{O)HL}]+[\text{fC3bH}]+[\text{fC3bHL}]+[\text{fC3bCR1}]+[\text{fiC3bCR1}]+[\text{IgGC3bC3bH}]+[\text{IgGC3biC3bH}]+[\text{IgGC3bC3bHL}]}\cdots \\ \frac{1}{+[\text{IgGC3biC3bHL}]+[\text{IgGC3bC3bCR1}]+[\text{IgGC3biC3bCR1}]+[\text{IgGiC3biC3bCR1}]+[\text{IgGiC3bC3dgCR1}]+[\text{fC3bC4bH}]+[\text{fC3bC4bHL}]}\cdots \\ \frac{1}{+[\text{fC3bC4bCR1}]+[\text{fC3bC4bC4BP}]+[\text{fC3bC4dH}]+[\text{fC3bC4dHL}]+[\text{fC3bC4dCR1}]+[\text{fiC3bC4dCR1}]+[\text{fiC3bC4bCR1}]+[\text{fiC3bC4bC4BP}]}\cdots \\ \frac{1}{+[\text{fC3dgC4bCR1}]+[\text{fC3dgC4bC4BP}]+[\text{fC3bC3bH}]+[\text{fC3bC3bHL}]+[\text{fC3bC3bCR1}]+[\text{fC3biC3bH}]+[\text{fC3biC3bHL}]+[\text{fC3biC3bCR1}]}\cdots \\ \frac{1}{+[\text{fiC3biC3bCR1}]+[\text{fiC3bC3dgCR1}]+[\text{hC3bH}]+[\text{hC3bHL}]+[\text{hC3bCR1}]+[\text{hiC3bCR1}]+[\text{hC3bC4bH}]+[\text{hC3bC4bHL}]+[\text{hC3bC4bCR1}]}\cdots \\ \frac{1}{+[\text{hC3bC4bC4BP}]+[\text{hC3bC4dH}]+[\text{hC3bC4dHL}]+[\text{hC3bC4dCR1}]+[\text{hiC3bC4dCR1}]+[\text{hiC3bC4bCR1}]+[\text{hiC3bC4bC4BP}]+[\text{hC3dgC4bCR1}]}\cdots \\ \frac{1}{+[\text{hC3dgC4bC4BP}]+[\text{hC3bC3bH}]+[\text{hC3bC3bHL}]+[\text{hC3bC3bCR1}]+[\text{hC3biC3bH}]+[\text{hC3biC3bHL}]+[\text{hC3biC3bCR1}]+[\text{hiC3biC3bCR1}]}\cdots \\ \frac{1}{+[\text{hiC3bC3dgCR1}]+[\text{fC4bCR1}]+[\text{nfC4bC4BP}]+[\text{fC4bC4BP}]+[\text{fC4bC4bCR1}]+[\text{fC4bC4bC4BP}]+[\text{fC4bC4dCR1}]+[\text{fC4bC4dC4BP}]+[\text{hC4bCR1}]}\cdots \\ \frac{1}{+[\text{hC4bC4BP}]+[\text{hC4bC4bCR1}]+[\text{hC4bC4bC4BP}]+[\text{hC4bC4dCR1}]+[\text{hC4bC4dC4BP}]}\cdots\end{array}\right)$$

$$-k_{\text{iC3biC3bCR1}}^{+}[\text{hiC3biC3b}][\text{CR1}]+k_{\text{iC3biC3bCR1}}^{-}[\text{hiC3biC3bCR1}]$$

S232

$$\frac{d[\text{hiC3biC3bCR1}]}{dt} = k^{+}_{\text{iC3biC3bCR1}}[\text{hiC3biC3b}][\text{CR1}] - k^{-}_{\text{iC3biC3bCR1}}[\text{hiC3biC3bCR1}]$$

$$-\left(\frac{k^{\text{FI}}_{\text{catC3bH}}[\text{I}][\text{hiC3biC3bCR1}]}{K^{\text{FI}}_{\text{mC3bH}} + [\text{C3(H}_2\text{O)H}] + [\text{C3(H}_2\text{O)HL}] + [\text{fC3bH}] + [\text{fC3bHL}] + [\text{fC3bCR1}] + [\text{fiC3bCR1}] + [\text{IgGC3bC3bH}] + [\text{IgGC3biC3bH}] + [\text{IgGC3bC3bHL}]} \cdots \right.$$
$$\frac{1}{+[\text{IgGC3biC3bHL}] + [\text{IgGC3bC3bCR1}] + [\text{IgGC3biC3bCR1}] + [\text{IgGiC3biC3bCR1}] + [\text{IgGiC3bC3dgCR1}] + [\text{fC3bC4bH}] + [\text{fC3bC4bHL}]} \cdots$$
$$\frac{1}{+[\text{fC3bC4bCR1}] + [\text{fC3bC4bC4BP}] + [\text{fC3bC4dH}] + [\text{fC3bC4dHL}] + [\text{fC3bC4dCR1}] + [\text{fiC3bC4dCR1}] + [\text{fiC3bC4bCR1}] + [\text{fiC3bC4bC4BP}]} \cdots$$
$$\frac{1}{+[\text{fC3dgC4bCR1}] + [\text{fC3dgC4bC4BP}] + [\text{fC3bC3bH}] + [\text{fC3bC3bHL}] + [\text{fC3bC3bCR1}] + [\text{fC3biC3bH}] + [\text{fC3biC3bHL}] + [\text{fC3biC3bCR1}]} \cdots$$
$$\frac{1}{+[\text{fiC3biC3bCR1}] + [\text{fiC3bC3dgCR1}] + [\text{hC3bH}] + [\text{hC3bHL}] + [\text{hC3bCR1}] + [\text{hiC3bCR1}] + [\text{hC3bC4bH}] + [\text{hC3bC4bHL}] + [\text{hC3bC4bCR1}]} \cdots$$
$$\frac{1}{+[\text{hC3bC4bC4BP}] + [\text{hC3bC4dH}] + [\text{hC3bC4dHL}] + [\text{hC3bC4dCR1}] + [\text{hiC3bC4dCR1}] + [\text{hiC3bC4bCR1}] + [\text{hiC3bC4bC4BP}] + [\text{hC3dgC4bCR1}]} \cdots$$
$$\frac{1}{+[\text{hC3dgC4bC4BP}] + [\text{hC3bC3bH}] + [\text{hC3bC3bHL}] + [\text{hC3bC3bCR1}] + [\text{hC3biC3bH}] + [\text{hC3biC3bHL}] + [\text{hC3biC3bCR1}] + [\text{hiC3biC3bCR1}]} \cdots$$
$$\frac{1}{+[\text{hiC3bC3dgCR1}] + [\text{fC4bCR1}] + [\text{nfC4bC4BP}] + [\text{fC4bC4BP}] + [\text{fC4bC4bCR1}] + [\text{fC4bC4bC4BP}] + [\text{fC4bC4dCR1}] + [\text{fC4bC4dC4BP}] + [\text{hC4bCR1}]} \cdots$$
$$\left. \frac{1}{+[\text{hC4bC4BP}] + [\text{hC4bC4bCR1}] + [\text{hC4bC4bC4BP}] + [\text{hC4bC4dCR1}] + [\text{hC4bC4dC4BP}]} \cdots \right)$$

S233

$$\frac{d[\text{hiC3bC3dg}]}{dt} =$$

$$+\left(\frac{k^{\text{FI}}_{\text{catC3bH}}[\text{I}][\text{hiC3biC3bCR1}]}{K^{\text{FI}}_{\text{mC3bH}} + [\text{C3(H}_2\text{O)H}] + [\text{C3(H}_2\text{O)HL}] + [\text{fC3bH}] + [\text{fC3bHL}] + [\text{fC3bCR1}] + [\text{fiC3bCR1}] + [\text{IgGC3bC3bH}] + [\text{IgGC3biC3bH}] + [\text{IgGC3bC3bHL}]} \cdots \right.$$
$$\frac{1}{+[\text{IgGC3biC3bHL}] + [\text{IgGC3bC3bCR1}] + [\text{IgGC3biC3bCR1}] + [\text{IgGiC3biC3bCR1}] + [\text{IgGiC3bC3dgCR1}] + [\text{fC3bC4bH}] + [\text{fC3bC4bHL}]} \cdots$$
$$\frac{1}{+[\text{fC3bC4bCR1}] + [\text{fC3bC4bC4BP}] + [\text{fC3bC4dH}] + [\text{fC3bC4dHL}] + [\text{fC3bC4dCR1}] + [\text{fiC3bC4dCR1}] + [\text{fiC3bC4bCR1}] + [\text{fiC3bC4bC4BP}]} \cdots$$
$$\frac{1}{+[\text{fC3dgC4bCR1}] + [\text{fC3dgC4bC4BP}] + [\text{fC3bC3bH}] + [\text{fC3bC3bHL}] + [\text{fC3bC3bCR1}] + [\text{fC3biC3bH}] + [\text{fC3biC3bHL}] + [\text{fC3biC3bCR1}]} \cdots$$
$$\frac{1}{+[\text{fiC3biC3bCR1}] + [\text{fiC3bC3dgCR1}] + [\text{hC3bH}] + [\text{hC3bHL}] + [\text{hC3bCR1}] + [\text{hiC3bCR1}] + [\text{hC3bC4bH}] + [\text{hC3bC4bHL}] + [\text{hC3bC4bCR1}]} \cdots$$
$$\frac{1}{+[\text{hC3bC4bC4BP}] + [\text{hC3bC4dH}] + [\text{hC3bC4dHL}] + [\text{hC3bC4dCR1}] + [\text{hiC3bC4dCR1}] + [\text{hiC3bC4bCR1}] + [\text{hiC3bC4bC4BP}] + [\text{hC3dgC4bCR1}]} \cdots$$
$$\frac{1}{+[\text{hC3dgC4bC4BP}] + [\text{hC3bC3bH}] + [\text{hC3bC3bHL}] + [\text{hC3bC3bCR1}] + [\text{hC3biC3bH}] + [\text{hC3biC3bHL}] + [\text{hC3biC3bCR1}] + [\text{hiC3biC3bCR1}]} \cdots$$
$$\frac{1}{+[\text{hiC3bC3dgCR1}] + [\text{fC4bCR1}] + [\text{nfC4bC4BP}] + [\text{fC4bC4BP}] + [\text{fC4bC4bCR1}] + [\text{fC4bC4bC4BP}] + [\text{fC4bC4dCR1}] + [\text{fC4bC4dC4BP}] + [\text{hC4bCR1}]} \cdots$$
$$\left. \frac{1}{+[\text{hC4bC4BP}] + [\text{hC4bC4bCR1}] + [\text{hC4bC4bC4BP}] + [\text{hC4bC4dCR1}] + [\text{hC4bC4dC4BP}]} \cdots \right)$$

$$- k^{+}_{\text{iC3bC3dgCR1}}[\text{hiC3bC3dg}][\text{CR1}] + k^{-}_{\text{iC3bC3dgCR1}}[\text{hiC3bC3dgCR1}]$$

S234

$$\frac{d[\text{hiC3bC3dgCR1}]}{dt} = k^{+}_{\text{iC3bC3dgCR1}}[\text{hiC3bC3dg}][\text{CR1}] - k^{-}_{\text{iC3bC3dgCR1}}[\text{hiC3bC3dgCR1}]$$

$$-\left(\frac{k^{\text{FI}}_{\text{catC3bH}}[\text{I}][\text{hiC3bC3dgCR1}]}{K^{\text{FI}}_{\text{mC3bH}} + [\text{C3(H}_2\text{O)H}] + [\text{C3(H}_2\text{O)HL}] + [\text{fC3bH}] + [\text{fC3bHL}] + [\text{fC3bCR1}] + [\text{fiC3bCR1}] + [\text{IgGC3bC3bH}] + [\text{IgGC3biC3bH}] + [\text{IgGC3bC3bHL}]} \cdots \frac{1}{+[\text{IgGC3biC3bHL}] + [\text{IgGC3bC3bCR1}] + [\text{IgGC3biC3bCR1}] + [\text{IgGiC3biC3bCR1}] + [\text{IgGiC3bC3dgCR1}] + [\text{fC3bC4bH}] + [\text{fC3bC4bHL}]} \cdots \frac{1}{+[\text{fC3bC4bCR1}] + [\text{fC3bC4bC4BP}] + [\text{fC3bC4dH}] + [\text{fC3bC4dHL}] + [\text{fC3bC4dCR1}] + [\text{fiC3bC4dCR1}] + [\text{fiC3bC4bCR1}] + [\text{fiC3bC4bC4BP}]} \cdots \frac{1}{+[\text{fC3dgC4bCR1}] + [\text{fC3dgC4bC4BP}] + [\text{fC3bC3bH}] + [\text{fC3bC3bHL}] + [\text{fC3bC3bCR1}] + [\text{fC3biC3bH}] + [\text{fC3biC3bHL}] + [\text{fC3biC3bCR1}]} \cdots \frac{1}{+[\text{fiC3biC3bCR1}] + [\text{fiC3bC3dgCR1}] + [\text{hC3bH}] + [\text{hC3bHL}] + [\text{hC3bCR1}] + [\text{hiC3bCR1}] + [\text{hC3bC4bH}] + [\text{hC3bC4bHL}] + [\text{hC3bC4bCR1}]} \cdots \frac{1}{+[\text{hC3bC4bC4BP}] + [\text{hC3bC4dH}] + [\text{hC3bC4dHL}] + [\text{hC3bC4dCR1}] + [\text{hiC3bC4dCR1}] + [\text{hiC3bC4bCR1}] + [\text{hiC3bC4bC4BP}] + [\text{hC3dgC4bCR1}]} \cdots \frac{1}{+[\text{hC3dgC4bC4BP}] + [\text{hC3bC3bH}] + [\text{hC3bC3bHL}] + [\text{hC3bC3bCR1}] + [\text{hC3biC3bH}] + [\text{hC3biC3bHL}] + [\text{hC3biC3bCR1}] + [\text{hiC3biC3bCR1}]} \cdots \frac{1}{+[\text{hiC3bC3dgCR1}] + [\text{fC4bCR1}] + [\text{nfC4bC4BP}] + [\text{fC4bC4BP}] + [\text{fC4bC4bCR1}] + [\text{fC4bC4bC4BP}] + [\text{fC4bC4dCR1}] + [\text{fC4bC4dC4BP}] + [\text{hC4bCR1}]} \cdots \frac{1}{+[\text{hC4bC4BP}] + [\text{hC4bC4bCR1}] + [\text{hC4bC4bC4BP}] + [\text{hC4bC4dCR1}] + [\text{hC4bC4dC4BP}]} \cdots\right)$$

S235

$$\frac{d[\text{hC3dgC3dg}]}{dt} =$$

$$\left(\frac{k^{\text{FI}}_{\text{catC3bH}}[\text{I}][\text{hiC3bC3dgCR1}]}{K^{\text{FI}}_{\text{mC3bH}} + [\text{C3(H}_2\text{O)H}] + [\text{C3(H}_2\text{O)HL}] + [\text{fC3bH}] + [\text{fC3bHL}] + [\text{fC3bCR1}] + [\text{fiC3bCR1}] + [\text{IgGC3bC3bH}] + [\text{IgGC3biC3bH}] + [\text{IgGC3bC3bHL}]} \cdots \frac{1}{+[\text{IgGC3biC3bHL}] + [\text{IgGC3bC3bCR1}] + [\text{IgGC3biC3bCR1}] + [\text{IgGiC3biC3bCR1}] + [\text{IgGiC3bC3dgCR1}] + [\text{fC3bC4bH}] + [\text{fC3bC4bHL}]} \cdots \frac{1}{+[\text{fC3bC4bCR1}] + [\text{fC3bC4bC4BP}] + [\text{fC3bC4dH}] + [\text{fC3bC4dHL}] + [\text{fC3bC4dCR1}] + [\text{fiC3bC4dCR1}] + [\text{fiC3bC4bCR1}] + [\text{fiC3bC4bC4BP}]} \cdots \frac{1}{+[\text{fC3dgC4bCR1}] + [\text{fC3dgC4bC4BP}] + [\text{fC3bC3bH}] + [\text{fC3bC3bHL}] + [\text{fC3bC3bCR1}] + [\text{fC3biC3bH}] + [\text{fC3biC3bHL}] + [\text{fC3biC3bCR1}]} \cdots \frac{1}{+[\text{fiC3biC3bCR1}] + [\text{fiC3bC3dgCR1}] + [\text{hC3bH}] + [\text{hC3bHL}] + [\text{hC3bCR1}] + [\text{hiC3bCR1}] + [\text{hC3bC4bH}] + [\text{hC3bC4bHL}] + [\text{hC3bC4bCR1}]} \cdots \frac{1}{+[\text{hC3bC4bC4BP}] + [\text{hC3bC4dH}] + [\text{hC3bC4dHL}] + [\text{hC3bC4dCR1}] + [\text{hiC3bC4dCR1}] + [\text{hiC3bC4bCR1}] + [\text{hiC3bC4bC4BP}] + [\text{hC3dgC4bCR1}]} \cdots \frac{1}{+[\text{hC3dgC4bC4BP}] + [\text{hC3bC3bH}] + [\text{hC3bC3bHL}] + [\text{hC3bC3bCR1}] + [\text{hC3biC3bH}] + [\text{hC3biC3bHL}] + [\text{hC3biC3bCR1}] + [\text{hiC3biC3bCR1}]} \cdots \frac{1}{+[\text{hiC3bC3dgCR1}] + [\text{fC4bCR1}] + [\text{nfC4bC4BP}] + [\text{fC4bC4BP}] + [\text{fC4bC4bCR1}] + [\text{fC4bC4bC4BP}] + [\text{fC4bC4dCR1}] + [\text{fC4bC4dC4BP}] + [\text{hC4bCR1}]} \cdots \frac{1}{+[\text{hC4bC4BP}] + [\text{hC4bC4bCR1}] + [\text{hC4bC4bC4BP}] + [\text{hC4bC4dCR1}] + [\text{hC4bC4dC4BP}]} \cdots\right)$$

S236

$$\frac{d[\text{hC3bC3bBbH}]}{dt} = k^{+}_{\text{C3bH}}[\text{hC3bC3bBb}][\text{H}] - k^{-}_{\text{C3bH}}[\text{hC3bC3bBbH}] - k^{-}_{\text{C3bBbH}_{\text{decay}}}[\text{hC3bC3bBbH}]$$

S237

$$\frac{d[\text{hC3bC3bBbHL}]}{dt} = k^{+}_{\text{C3bHL}}[\text{hC3bC3bBb}][\text{HL}] - k^{-}_{\text{C3bHL}}[\text{hC3bC3bBbHL}] - k^{-}_{\text{C3bBbHL}_{\text{decay}}}[\text{hC3bC3bBbHL}]$$

S238

$$\frac{d[\text{hC3bC3bBbCR1}]}{dt} = k^{+}_{\text{C3bC3bCR1}}[\text{hC3bC3bBb}][\text{CR1}] - k^{-}_{\text{C3bC3bCR1}}[\text{hC3bC3bBbCR1}] - k^{-}_{\text{C3bBbCR1}_{\text{decay}}}[\text{hC3bC3bBbCR1}]$$

S239

$$\frac{d[\text{hC3bC3bBbDAF}]}{dt} = k^{+}_{\text{C3bBbDAF}}[\text{hC3bC3bBb}][\text{DAF}] - k^{-}_{\text{C3bBbDAF}}[\text{hC3bC3bBbDAF}] - k^{-}_{\text{C3bBbDAF}_{\text{decay}}}[\text{hC3bC3bBbDAF}]$$

S240

$$\frac{d[\text{hC4bC4BP}]}{dt} = k^{+}_{\text{C4bC4BP}}[\text{hC4b}][\text{C4BP}] - k^{-}_{\text{C4bC4BP}}[\text{hC4bC4BP}]$$

$$-\left(\frac{k^{\text{FI}}_{\text{catC3bH}}[\text{I}][\text{hC4bC4BP}]}{K^{\text{FI}}_{\text{mC3bH}} + [\text{C3(H}_2\text{O)H}] + [\text{C3(H}_2\text{O)HL}] + [\text{fC3bH}] + [\text{fC3bHL}] + [\text{fC3bCR1}] + [\text{fiC3bCR1}] + [\text{IgGC3bC3bH}] + [\text{IgGC3biC3bH}] + [\text{IgGC3bC3bHL}]} \cdots\right.$$

$$\frac{1}{+[\text{IgGC3biC3bHL}] + [\text{IgGC3bC3bCR1}] + [\text{IgGC3biC3bCR1}] + [\text{IgGiC3biC3bCR1}] + [\text{IgGiC3bC3dgCR1}] + [\text{fC3bC4bH}] + [\text{fC3bC4bHL}]} \cdots$$

$$\frac{1}{+[\text{fC3bC4bCR1}] + [\text{fC3bC4bC4BP}] + [\text{fC3bC4dH}] + [\text{fC3bC4dHL}] + [\text{fC3bC4dCR1}] + [\text{fiC3bC4dCR1}] + [\text{fiC3bC4bCR1}] + [\text{fiC3bC4bC4BP}]} \cdots$$

$$\frac{1}{+[\text{fC3dgC4bCR1}] + [\text{fC3dgC4bC4BP}] + [\text{fC3bC3bH}] + [\text{fC3bC3bHL}] + [\text{fC3bC3bCR1}] + [\text{fC3biC3bH}] + [\text{fC3biC3bHL}] + [\text{fC3biC3bCR1}]} \cdots$$

$$\frac{1}{+[\text{fiC3biC3bCR1}] + [\text{fiC3bC3dgCR1}] + [\text{hC3bH}] + [\text{hC3bHL}] + [\text{hC3bCR1}] + [\text{hiC3bCR1}] + [\text{hC3bC4bH}] + [\text{hC3bC4bHL}] + [\text{hC3bC4bCR1}]} \cdots$$

$$\frac{1}{+[\text{hC3bC4bC4BP}] + [\text{hC3bC4dH}] + [\text{hC3bC4dHL}] + [\text{hC3bC4dCR1}] + [\text{hiC3bC4dCR1}] + [\text{hiC3bC4bCR1}] + [\text{hiC3bC4bC4BP}] + [\text{hC3dgC4bCR1}]} \cdots$$

$$\frac{1}{+[\text{hC3dgC4bC4BP}] + [\text{hC3bC3bH}] + [\text{hC3bC3bHL}] + [\text{hC3bC3bCR1}] + [\text{hC3biC3bH}] + [\text{hC3biC3bHL}] + [\text{hC3biC3bCR1}] + [\text{hiC3biC3bCR1}]} \cdots$$

$$\frac{1}{+[\text{hiC3bC3dgCR1}] + [\text{fC4bCR1}] + [\text{nfC4bC4BP}] + [\text{fC4bC4BP}] + [\text{fC4bC4bCR1}] + [\text{fC4bC4bC4BP}] + [\text{fC4bC4dCR1}] + [\text{fC4bC4dC4BP}] + [\text{hC4bCR1}]} \cdots$$

$$\left.\frac{1}{+[\text{hC4bC4BP}] + [\text{hC4bC4bCR1}] + [\text{hC4bC4bC4BP}] + [\text{hC4bC4dCR1}] + [\text{hC4bC4dC4BP}]} \cdots\right)$$

S241

$$\frac{d[\text{hC4bCR1}]}{dt} = k^{+}_{\text{C4bCR1}}[\text{hC4b}][\text{CR1}] - k^{-}_{\text{C4bCR1}}[\text{hC4bCR1}]$$

$$-\left(\frac{k^{\text{FI}}_{\text{catC3bH}}[\text{I}][\text{hC4bCR1}]}{K^{\text{FI}}_{\text{mC3bH}} + [\text{C3(H}_2\text{O)H}] + [\text{C3(H}_2\text{O)HL}] + [\text{fC3bH}] + [\text{fC3bHL}] + [\text{fC3bCR1}] + [\text{fiC3bCR1}] + [\text{IgGC3bC3bH}] + [\text{IgGC3biC3bH}] + [\text{IgGC3bC3bHL}]} \cdots \right.$$

$$\frac{1}{+[\text{IgGC3biC3bHL}] + [\text{IgGC3bC3bCR1}] + [\text{IgGC3biC3bCR1}] + [\text{IgGiC3biC3bCR1}] + [\text{IgGiC3bC3dgCR1}] + [\text{fC3bC4bH}] + [\text{fC3bC4bHL}]} \cdots$$

$$\frac{1}{+[\text{fC3bC4bCR1}] + [\text{fC3bC4bC4BP}] + [\text{fC3bC4dH}] + [\text{fC3bC4dHL}] + [\text{fC3bC4dCR1}] + [\text{fiC3bC4dCR1}] + [\text{fiC3bC4bCR1}] + [\text{fiC3bC4bC4BP}]} \cdots$$

$$\frac{1}{+[\text{fC3dgC4bCR1}] + [\text{fC3dgC4bC4BP}] + [\text{fC3bC3bH}] + [\text{fC3bC3bHL}] + [\text{fC3bC3bCR1}] + [\text{fC3biC3bH}] + [\text{fC3biC3bHL}] + [\text{fC3biC3bCR1}]} \cdots$$

$$\frac{1}{+[\text{fiC3biC3bCR1}] + [\text{fiC3bC3dgCR1}] + [\text{hC3bH}] + [\text{hC3bHL}] + [\text{hC3bCR1}] + [\text{hiC3bCR1}] + [\text{hC3bC4bH}] + [\text{hC3bC4bHL}] + [\text{hC3bC4bCR1}]} \cdots$$

$$\frac{1}{+[\text{hC3bC4bC4BP}] + [\text{hC3bC4dH}] + [\text{hC3bC4dHL}] + [\text{hC3bC4dCR1}] + [\text{hiC3bC4dCR1}] + [\text{hiC3bC4bCR1}] + [\text{hiC3bC4bC4BP}] + [\text{hC3dgC4bCR1}]} \cdots$$

$$\frac{1}{+[\text{hC3dgC4bC4BP}] + [\text{hC3bC3bH}] + [\text{hC3bC3bHL}] + [\text{hC3bC3bCR1}] + [\text{hC3biC3bH}] + [\text{hC3biC3bHL}] + [\text{hC3biC3bCR1}] + [\text{hiC3biC3bCR1}]} \cdots$$

$$\frac{1}{+[\text{hiC3bC3dgCR1}] + [\text{fC4bCR1}] + [\text{nfC4bC4BP}] + [\text{fC4bC4BP}] + [\text{fC4bC4bCR1}] + [\text{fC4bC4bC4BP}] + [\text{fC4bC4dCR1}] + [\text{fC4bC4dC4BP}] + [\text{hC4bCR1}]} \cdots$$

$$\left. \frac{1}{+[\text{hC4bC4BP}] + [\text{hC4bC4bCR1}] + [\text{hC4bC4bC4BP}] + [\text{hC4bC4dCR1}] + [\text{hC4bC4dC4BP}]} \cdots \right)$$

S242

$$\frac{d[\text{hC4d}]}{dt} =$$

$$\left(\frac{k^{\text{FI}}_{\text{catC3bH}}[\text{I}][\text{hC4bC4BP}]}{K^{\text{FI}}_{\text{mC3bH}} + [\text{C3(H}_2\text{O)H}] + [\text{C3(H}_2\text{O)HL}] + [\text{fC3bH}] + [\text{fC3bHL}] + [\text{fC3bCR1}] + [\text{fiC3bCR1}] + [\text{IgGC3bC3bH}] + [\text{IgGC3biC3bH}] + [\text{IgGC3bC3bHL}]} \cdots \right.$$

$$\frac{1}{+[\text{IgGC3biC3bHL}] + [\text{IgGC3bC3bCR1}] + [\text{IgGC3biC3bCR1}] + [\text{IgGiC3biC3bCR1}] + [\text{IgGiC3bC3dgCR1}] + [\text{fC3bC4bH}] + [\text{fC3bC4bHL}]} \cdots$$

$$\frac{1}{+[\text{fC3bC4bCR1}] + [\text{fC3bC4bC4BP}] + [\text{fC3bC4dH}] + [\text{fC3bC4dHL}] + [\text{fC3bC4dCR1}] + [\text{fiC3bC4dCR1}] + [\text{fiC3bC4bCR1}] + [\text{fiC3bC4bC4BP}]} \cdots$$

$$\frac{1}{+[\text{fC3dgC4bCR1}] + [\text{fC3dgC4bC4BP}] + [\text{fC3bC3bH}] + [\text{fC3bC3bHL}] + [\text{fC3bC3bCR1}] + [\text{fC3biC3bH}] + [\text{fC3biC3bHL}] + [\text{fC3biC3bCR1}]} \cdots$$

$$\frac{1}{+[\text{fiC3biC3bCR1}] + [\text{fiC3bC3dgCR1}] + [\text{hC3bH}] + [\text{hC3bHL}] + [\text{hC3bCR1}] + [\text{hiC3bCR1}] + [\text{hC3bC4bH}] + [\text{hC3bC4bHL}] + [\text{hC3bC4bCR1}]} \cdots$$

$$\frac{1}{+[\text{hC3bC4bC4BP}] + [\text{hC3bC4dH}] + [\text{hC3bC4dHL}] + [\text{hC3bC4dCR1}] + [\text{hiC3bC4dCR1}] + [\text{hiC3bC4bCR1}] + [\text{hiC3bC4bC4BP}] + [\text{hC3dgC4bCR1}]} \cdots$$

$$\frac{1}{+[\text{hC3dgC4bC4BP}] + [\text{hC3bC3bH}] + [\text{hC3bC3bHL}] + [\text{hC3bC3bCR1}] + [\text{hC3biC3bH}] + [\text{hC3biC3bHL}] + [\text{hC3biC3bCR1}] + [\text{hiC3biC3bCR1}]} \cdots$$

$$\frac{1}{+[\text{hiC3bC3dgCR1}] + [\text{fC4bCR1}] + [\text{nfC4bC4BP}] + [\text{fC4bC4BP}] + [\text{fC4bC4bCR1}] + [\text{fC4bC4bC4BP}] + [\text{fC4bC4dCR1}] + [\text{fC4bC4dC4BP}] + [\text{hC4bCR1}]} \cdots$$

$$\left. \frac{1}{+[\text{hC4bC4BP}] + [\text{hC4bC4bCR1}] + [\text{hC4bC4bC4BP}] + [\text{hC4bC4dCR1}] + [\text{hC4bC4dC4BP}]} \cdots \right)$$

$$+\left(\frac{k_{\mathrm{catC3bH}}^{\mathrm{FI}}[\mathrm{I}][\mathrm{hC4bCR1}]}{K_{\mathrm{mC3bH}}^{\mathrm{FI}}+[\mathrm{C3(H_2O)H}]+[\mathrm{C3(H_2O)HL}]+[\mathrm{fC3bH}]+[\mathrm{fC3bHL}]+[\mathrm{fC3bCR1}]+[\mathrm{fiC3bCR1}]+[\mathrm{IgGC3bC3bH}]+[\mathrm{IgGC3biC3bH}]+[\mathrm{IgGC3bC3bHL}]}\cdots \frac{1}{+[\mathrm{IgGC3biC3bHL}]+[\mathrm{IgGC3bC3bCR1}]+[\mathrm{IgGC3biC3bCR1}]+[\mathrm{IgGiC3biC3bCR1}]+[\mathrm{IgGiC3bC3dgCR1}]+[\mathrm{fC3bC4bH}]+[\mathrm{fC3bC4bHL}]}\cdots \frac{1}{+[\mathrm{fC3bC4bCR1}]+[\mathrm{fC3bC4bC4BP}]+[\mathrm{fC3bC4dH}]+[\mathrm{fC3bC4dHL}]+[\mathrm{fC3bC4dCR1}]+[\mathrm{fiC3bC4dCR1}]+[\mathrm{fiC3bC4bCR1}]+[\mathrm{fiC3bC4bC4BP}]}\cdots \frac{1}{+[\mathrm{fC3dgC4bCR1}]+[\mathrm{fC3dgC4bC4BP}]+[\mathrm{fC3bC3bH}]+[\mathrm{fC3bC3bHL}]+[\mathrm{fC3bC3bCR1}]+[\mathrm{fC3biC3bH}]+[\mathrm{fC3biC3bHL}]+[\mathrm{fC3biC3bCR1}]}\cdots \frac{1}{+[\mathrm{fiC3biC3bCR1}]+[\mathrm{fiC3bC3dgCR1}]+[\mathrm{hC3bH}]+[\mathrm{hC3bHL}]+[\mathrm{hC3bCR1}]+[\mathrm{hiC3bCR1}]+[\mathrm{hC3bC4bH}]+[\mathrm{hC3bC4bHL}]+[\mathrm{hC3bC4bCR1}]}\cdots \frac{1}{+[\mathrm{hC3bC4bC4BP}]+[\mathrm{hC3bC4dH}]+[\mathrm{hC3bC4dHL}]+[\mathrm{hC3bC4dCR1}]+[\mathrm{hiC3bC4dCR1}]+[\mathrm{hiC3bC4bCR1}]+[\mathrm{hiC3bC4bC4BP}]+[\mathrm{hC3dgC4bCR1}]}\cdots \frac{1}{+[\mathrm{hC3dgC4bC4BP}]+[\mathrm{hC3bC3bH}]+[\mathrm{hC3bC3bHL}]+[\mathrm{hC3bC3bCR1}]+[\mathrm{hC3biC3bH}]+[\mathrm{hC3biC3bHL}]+[\mathrm{hC3biC3bCR1}]+[\mathrm{hiC3biC3bCR1}]}\cdots \frac{1}{+[\mathrm{hiC3bC3dgCR1}]+[\mathrm{fC4bCR1}]+[\mathrm{nfC4bC4BP}]+[\mathrm{fC4bC4BP}]+[\mathrm{fC4bC4bCR1}]+[\mathrm{fC4bC4bC4BP}]+[\mathrm{fC4bC4dCR1}]+[\mathrm{fC4bC4dC4BP}]+[\mathrm{hC4bCR1}]}\cdots \frac{1}{+[\mathrm{hC4bC4BP}]+[\mathrm{hC4bC4bCR1}]+[\mathrm{hC4bC4bC4BP}]+[\mathrm{hC4bC4dCR1}]+[\mathrm{hC4bC4dC4BP}]}\cdots\right)$$

S243

$$\frac{d[\mathrm{hC4bC4bC4BP}]}{dt}=k_{\mathrm{C4bC4BP}}^{+}[\mathrm{hC4bC4b}][\mathrm{C4BP}]-k_{\mathrm{C4bC4BP}}^{-}[\mathrm{hC4bC4bC4BP}]$$

$$-\left(\frac{k_{\mathrm{catC3bH}}^{\mathrm{FI}}[\mathrm{I}][\mathrm{hC4bC4bC4BP}]}{K_{\mathrm{mC3bH}}^{\mathrm{FI}}+[\mathrm{C3(H_2O)H}]+[\mathrm{C3(H_2O)HL}]+[\mathrm{fC3bH}]+[\mathrm{fC3bHL}]+[\mathrm{fC3bCR1}]+[\mathrm{fiC3bCR1}]+[\mathrm{IgGC3bC3bH}]+[\mathrm{IgGC3biC3bH}]+[\mathrm{IgGC3bC3bHL}]}\cdots \frac{1}{+[\mathrm{IgGC3biC3bHL}]+[\mathrm{IgGC3bC3bCR1}]+[\mathrm{IgGC3biC3bCR1}]+[\mathrm{IgGiC3biC3bCR1}]+[\mathrm{IgGiC3bC3dgCR1}]+[\mathrm{fC3bC4bH}]+[\mathrm{fC3bC4bHL}]}\cdots \frac{1}{+[\mathrm{fC3bC4bCR1}]+[\mathrm{fC3bC4bC4BP}]+[\mathrm{fC3bC4dH}]+[\mathrm{fC3bC4dHL}]+[\mathrm{fC3bC4dCR1}]+[\mathrm{fiC3bC4dCR1}]+[\mathrm{fiC3bC4bCR1}]+[\mathrm{fiC3bC4bC4BP}]}\cdots \frac{1}{+[\mathrm{fC3dgC4bCR1}]+[\mathrm{fC3dgC4bC4BP}]+[\mathrm{fC3bC3bH}]+[\mathrm{fC3bC3bHL}]+[\mathrm{fC3bC3bCR1}]+[\mathrm{fC3biC3bH}]+[\mathrm{fC3biC3bHL}]+[\mathrm{fC3biC3bCR1}]}\cdots \frac{1}{+[\mathrm{fiC3biC3bCR1}]+[\mathrm{fiC3bC3dgCR1}]+[\mathrm{hC3bH}]+[\mathrm{hC3bHL}]+[\mathrm{hC3bCR1}]+[\mathrm{hiC3bCR1}]+[\mathrm{hC3bC4bH}]+[\mathrm{hC3bC4bHL}]+[\mathrm{hC3bC4bCR1}]}\cdots \frac{1}{+[\mathrm{hC3bC4bC4BP}]+[\mathrm{hC3bC4dH}]+[\mathrm{hC3bC4dHL}]+[\mathrm{hC3bC4dCR1}]+[\mathrm{hiC3bC4dCR1}]+[\mathrm{hiC3bC4bCR1}]+[\mathrm{hiC3bC4bC4BP}]+[\mathrm{hC3dgC4bCR1}]}\cdots \frac{1}{+[\mathrm{hC3dgC4bC4BP}]+[\mathrm{hC3bC3bH}]+[\mathrm{hC3bC3bHL}]+[\mathrm{hC3bC3bCR1}]+[\mathrm{hC3biC3bH}]+[\mathrm{hC3biC3bHL}]+[\mathrm{hC3biC3bCR1}]+[\mathrm{hiC3biC3bCR1}]}\cdots \frac{1}{+[\mathrm{hiC3bC3dgCR1}]+[\mathrm{fC4bCR1}]+[\mathrm{nfC4bC4BP}]+[\mathrm{fC4bC4BP}]+[\mathrm{fC4bC4bCR1}]+[\mathrm{fC4bC4bC4BP}]+[\mathrm{fC4bC4dCR1}]+[\mathrm{fC4bC4dC4BP}]+[\mathrm{hC4bCR1}]}\cdots \frac{1}{+[\mathrm{hC4bC4BP}]+[\mathrm{hC4bC4bCR1}]+[\mathrm{hC4bC4bC4BP}]+[\mathrm{hC4bC4dCR1}]+[\mathrm{hC4bC4dC4BP}]}\cdots\right)$$

S244

$$\frac{d[\text{hC4bC4bCR1}]}{dt} = k^{+}_{\text{C4bC4bCR1}}[\text{hC4bC4b}][\text{CR1}] - k^{-}_{\text{C4bC4bCR1}}[\text{hC4bC4bCR1}]$$

$$-\left(\frac{k^{\text{FI}}_{\text{catC3bH}}[\text{I}][\text{hC4bC4bCR1}]}{K^{\text{FI}}_{\text{mC3bH}} + [\text{C3(H}_2\text{O)H}] + [\text{C3(H}_2\text{O)HL}] + [\text{fC3bH}] + [\text{fC3bHL}] + [\text{fC3bCR1}] + [\text{fiC3bCR1}] + [\text{IgGC3bC3bH}] + [\text{IgGC3biC3bH}] + [\text{IgGC3bC3bHL}]} \cdots \right.$$

$$\frac{1}{+[\text{IgGC3biC3bHL}] + [\text{IgGC3bC3bCR1}] + [\text{IgGC3biC3bCR1}] + [\text{IgGiC3biC3bCR1}] + [\text{IgGiC3bC3dgCR1}] + [\text{fC3bC4bH}] + [\text{fC3bC4bHL}]} \cdots$$

$$\frac{1}{+[\text{fC3bC4bCR1}] + [\text{fC3bC4bC4BP}] + [\text{fC3bC4dH}] + [\text{fC3bC4dHL}] + [\text{fC3bC4dCR1}] + [\text{fiC3bC4dCR1}] + [\text{fiC3bC4bCR1}] + [\text{fiC3bC4bC4BP}]} \cdots$$

$$\frac{1}{+[\text{fC3dgC4bCR1}] + [\text{fC3dgC4bC4BP}] + [\text{fC3bC3bH}] + [\text{fC3bC3bHL}] + [\text{fC3bC3bCR1}] + [\text{fC3biC3bH}] + [\text{fC3biC3bHL}] + [\text{fC3biC3bCR1}]} \cdots$$

$$\frac{1}{+[\text{fiC3biC3bCR1}] + [\text{fiC3bC3dgCR1}] + [\text{hC3bH}] + [\text{hC3bHL}] + [\text{hC3bCR1}] + [\text{hiC3bCR1}] + [\text{hC3bC4bH}] + [\text{hC3bC4bHL}] + [\text{hC3bC4bCR1}]} \cdots$$

$$\frac{1}{+[\text{hC3bC4bC4BP}] + [\text{hC3bC4dH}] + [\text{hC3bC4dHL}] + [\text{hC3bC4dCR1}] + [\text{hiC3bC4dCR1}] + [\text{hiC3bC4bCR1}] + [\text{hiC3bC4bC4BP}] + [\text{hC3dgC4bCR1}]} \cdots$$

$$\frac{1}{+[\text{hC3dgC4bC4BP}] + [\text{hC3bC3bH}] + [\text{hC3bC3bHL}] + [\text{hC3bC3bCR1}] + [\text{hC3biC3bH}] + [\text{hC3biC3bHL}] + [\text{hC3biC3bCR1}] + [\text{hiC3biC3bCR1}]} \cdots$$

$$\frac{1}{+[\text{hiC3bC3dgCR1}] + [\text{fC4bCR1}] + [\text{nfC4bC4BP}] + [\text{fC4bC4BP}] + [\text{fC4bC4bCR1}] + [\text{fC4bC4bC4BP}] + [\text{fC4bC4dCR1}] + [\text{fC4bC4dC4BP}] + [\text{hC4bCR1}]} \cdots$$

$$\left. \frac{1}{+[\text{hC4bC4BP}] + [\text{hC4bC4bCR1}] + [\text{hC4bC4bC4BP}] + [\text{hC4bC4dCR1}] + [\text{hC4bC4dC4BP}]} \cdots \right)$$

S245

$$\frac{d[\text{hC4bC4d}]}{dt} =$$

$$\left(\frac{k^{\text{FI}}_{\text{catC3bH}}[\text{I}][\text{hC4bC4bC4BP}]}{K^{\text{FI}}_{\text{mC3bH}} + [\text{C3(H}_2\text{O)H}] + [\text{C3(H}_2\text{O)HL}] + [\text{fC3bH}] + [\text{fC3bHL}] + [\text{fC3bCR1}] + [\text{fiC3bCR1}] + [\text{IgGC3bC3bH}] + [\text{IgGC3biC3bH}] + [\text{IgGC3bC3bHL}]} \cdots \right.$$

$$\frac{1}{+[\text{IgGC3biC3bHL}] + [\text{IgGC3bC3bCR1}] + [\text{IgGC3biC3bCR1}] + [\text{IgGiC3biC3bCR1}] + [\text{IgGiC3bC3dgCR1}] + [\text{fC3bC4bH}] + [\text{fC3bC4bHL}]} \cdots$$

$$\frac{1}{+[\text{fC3bC4bCR1}] + [\text{fC3bC4bC4BP}] + [\text{fC3bC4dH}] + [\text{fC3bC4dHL}] + [\text{fC3bC4dCR1}] + [\text{fiC3bC4dCR1}] + [\text{fiC3bC4bCR1}] + [\text{fiC3bC4bC4BP}]} \cdots$$

$$\frac{1}{+[\text{fC3dgC4bCR1}] + [\text{fC3dgC4bC4BP}] + [\text{fC3bC3bH}] + [\text{fC3bC3bHL}] + [\text{fC3bC3bCR1}] + [\text{fC3biC3bH}] + [\text{fC3biC3bHL}] + [\text{fC3biC3bCR1}]} \cdots$$

$$\frac{1}{+[\text{fiC3biC3bCR1}] + [\text{fiC3bC3dgCR1}] + [\text{hC3bH}] + [\text{hC3bHL}] + [\text{hC3bCR1}] + [\text{hiC3bCR1}] + [\text{hC3bC4bH}] + [\text{hC3bC4bHL}] + [\text{hC3bC4bCR1}]} \cdots$$

$$\frac{1}{+[\text{hC3bC4bC4BP}] + [\text{hC3bC4dH}] + [\text{hC3bC4dHL}] + [\text{hC3bC4dCR1}] + [\text{hiC3bC4dCR1}] + [\text{hiC3bC4bCR1}] + [\text{hiC3bC4bC4BP}] + [\text{hC3dgC4bCR1}]} \cdots$$

$$\frac{1}{+[\text{hC3dgC4bC4BP}] + [\text{hC3bC3bH}] + [\text{hC3bC3bHL}] + [\text{hC3bC3bCR1}] + [\text{hC3biC3bH}] + [\text{hC3biC3bHL}] + [\text{hC3biC3bCR1}] + [\text{hiC3biC3bCR1}]} \cdots$$

$$\frac{1}{+[\text{hiC3bC3dgCR1}] + [\text{fC4bCR1}] + [\text{nfC4bC4BP}] + [\text{fC4bC4BP}] + [\text{fC4bC4bCR1}] + [\text{fC4bC4bC4BP}] + [\text{fC4bC4dCR1}] + [\text{fC4bC4dC4BP}] + [\text{hC4bCR1}]} \cdots$$

$$\left. \frac{1}{+[\text{hC4bC4BP}] + [\text{hC4bC4bCR1}] + [\text{hC4bC4bC4BP}] + [\text{hC4bC4dCR1}] + [\text{hC4bC4dC4BP}]} \cdots \right)$$

$$
+\left(\begin{array}{l}
\frac{k_{\mathrm{catC3bH}}^{\mathrm{FI}}[\mathrm{I}][\mathrm{hC4bC4bCR1}]}{K_{\mathrm{mC3bH}}^{\mathrm{FI}}+[\mathrm{C3(H_2O)H}]+[\mathrm{C3(H_2O)HL}]+[\mathrm{fC3bH}]+[\mathrm{fC3bHL}]+[\mathrm{fC3bCR1}]+[\mathrm{fiC3bCR1}]+[\mathrm{IgGC3bC3bH}]+[\mathrm{IgGC3biC3bH}]+[\mathrm{IgGC3bC3bHL}]}\cdots \\
\frac{1}{+[\mathrm{IgGC3biC3bHL}]+[\mathrm{IgGC3bC3bCR1}]+[\mathrm{IgGC3biC3bCR1}]+[\mathrm{IgGiC3biC3bCR1}]+[\mathrm{IgGiC3bC3dgCR1}]+[\mathrm{fC3bC4bH}]+[\mathrm{fC3bC4bHL}]}\cdots \\
\frac{1}{+[\mathrm{fC3bC4bCR1}]+[\mathrm{fC3bC4bC4BP}]+[\mathrm{fC3bC4dH}]+[\mathrm{fC3bC4dHL}]+[\mathrm{fC3bC4dCR1}]+[\mathrm{fiC3bC4dCR1}]+[\mathrm{fiC3bC4bCR1}]+[\mathrm{fiC3bC4bC4BP}]}\cdots \\
\frac{1}{+[\mathrm{fC3dgC4bCR1}]+[\mathrm{fC3dgC4bC4BP}]+[\mathrm{fC3bC3bH}]+[\mathrm{fC3bC3bHL}]+[\mathrm{fC3bC3bCR1}]+[\mathrm{fC3biC3bH}]+[\mathrm{fC3biC3bHL}]+[\mathrm{fC3biC3bCR1}]}\cdots \\
\frac{1}{+[\mathrm{fiC3biC3bCR1}]+[\mathrm{fiC3bC3dgCR1}]+[\mathrm{hC3bH}]+[\mathrm{hC3bHL}]+[\mathrm{hC3bCR1}]+[\mathrm{hiC3bCR1}]+[\mathrm{hC3bC4bH}]+[\mathrm{hC3bC4bHL}]+[\mathrm{hC3bC4bCR1}]}\cdots \\
\frac{1}{+[\mathrm{hC3bC4bC4BP}]+[\mathrm{hC3bC4dH}]+[\mathrm{hC3bC4dHL}]+[\mathrm{hC3bC4dCR1}]+[\mathrm{hiC3bC4dCR1}]+[\mathrm{hiC3bC4bCR1}]+[\mathrm{hiC3bC4bC4BP}]+[\mathrm{hC3dgC4bCR1}]}\cdots \\
\frac{1}{+[\mathrm{hC3dgC4bC4BP}]+[\mathrm{hC3bC3bH}]+[\mathrm{hC3bC3bHL}]+[\mathrm{hC3bC3bCR1}]+[\mathrm{hC3biC3bH}]+[\mathrm{hC3biC3bHL}]+[\mathrm{hC3biC3bCR1}]+[\mathrm{hiC3biC3bCR1}]}\cdots \\
\frac{1}{+[\mathrm{hiC3bC3dgCR1}]+[\mathrm{fC4bCR1}]+[\mathrm{nfC4bC4BP}]+[\mathrm{fC4bC4BP}]+[\mathrm{fC4bC4bCR1}]+[\mathrm{fC4bC4bC4BP}]+[\mathrm{fC4bC4dCR1}]+[\mathrm{fC4bC4dC4BP}]+[\mathrm{hC4bCR1}]}\cdots \\
\frac{1}{+[\mathrm{hC4bC4BP}]+[\mathrm{hC4bC4bCR1}]+[\mathrm{hC4bC4bC4BP}]+[\mathrm{hC4bC4dCR1}]+[\mathrm{hC4bC4dC4BP}]}\cdots
\end{array}\right)
$$

$$
-k_{\mathrm{C4bC4BP}}^{+}[\mathrm{hC4bC4d}][\mathrm{C4BP}]+k_{\mathrm{C4bC4BP}}^{-}[\mathrm{hC4bC4dC4BP}]-k_{\mathrm{C4bC4dCR1}}^{+}[\mathrm{hC4bC4d}][\mathrm{CR1}]+k_{\mathrm{C4bC4dCR1}}^{-}[\mathrm{hC4bC4dCR1}]
$$

S246

$$
\frac{d[\mathrm{hC4bC4dC4BP}]}{dt}=k_{\mathrm{C4bC4BP}}^{+}[\mathrm{hC4bC4d}][\mathrm{C4BP}]-k_{\mathrm{C4bC4BP}}^{-}[\mathrm{hC4bC4dC4BP}]
$$

$$
-\left(\begin{array}{l}
\frac{k_{\mathrm{catC3bH}}^{\mathrm{FI}}[\mathrm{I}][\mathrm{hC4bC4dC4BP}]}{K_{\mathrm{mC3bH}}^{\mathrm{FI}}+[\mathrm{C3(H_2O)H}]+[\mathrm{C3(H_2O)HL}]+[\mathrm{fC3bH}]+[\mathrm{fC3bHL}]+[\mathrm{fC3bCR1}]+[\mathrm{fiC3bCR1}]+[\mathrm{IgGC3bC3bH}]+[\mathrm{IgGC3biC3bH}]+[\mathrm{IgGC3bC3bHL}]}\cdots \\
\frac{1}{+[\mathrm{IgGC3biC3bHL}]+[\mathrm{IgGC3bC3bCR1}]+[\mathrm{IgGC3biC3bCR1}]+[\mathrm{IgGiC3biC3bCR1}]+[\mathrm{IgGiC3bC3dgCR1}]+[\mathrm{fC3bC4bH}]+[\mathrm{fC3bC4bHL}]}\cdots \\
\frac{1}{+[\mathrm{fC3bC4bCR1}]+[\mathrm{fC3bC4bC4BP}]+[\mathrm{fC3bC4dH}]+[\mathrm{fC3bC4dHL}]+[\mathrm{fC3bC4dCR1}]+[\mathrm{fiC3bC4dCR1}]+[\mathrm{fiC3bC4bCR1}]+[\mathrm{fiC3bC4bC4BP}]}\cdots \\
\frac{1}{+[\mathrm{fC3dgC4bCR1}]+[\mathrm{fC3dgC4bC4BP}]+[\mathrm{fC3bC3bH}]+[\mathrm{fC3bC3bHL}]+[\mathrm{fC3bC3bCR1}]+[\mathrm{fC3biC3bH}]+[\mathrm{fC3biC3bHL}]+[\mathrm{fC3biC3bCR1}]}\cdots \\
\frac{1}{+[\mathrm{fiC3biC3bCR1}]+[\mathrm{fiC3bC3dgCR1}]+[\mathrm{hC3bH}]+[\mathrm{hC3bHL}]+[\mathrm{hC3bCR1}]+[\mathrm{hiC3bCR1}]+[\mathrm{hC3bC4bH}]+[\mathrm{hC3bC4bHL}]+[\mathrm{hC3bC4bCR1}]}\cdots \\
\frac{1}{+[\mathrm{hC3bC4bC4BP}]+[\mathrm{hC3bC4dH}]+[\mathrm{hC3bC4dHL}]+[\mathrm{hC3bC4dCR1}]+[\mathrm{hiC3bC4dCR1}]+[\mathrm{hiC3bC4bCR1}]+[\mathrm{hiC3bC4bC4BP}]+[\mathrm{hC3dgC4bCR1}]}\cdots \\
\frac{1}{+[\mathrm{hC3dgC4bC4BP}]+[\mathrm{hC3bC3bH}]+[\mathrm{hC3bC3bHL}]+[\mathrm{hC3bC3bCR1}]+[\mathrm{hC3biC3bH}]+[\mathrm{hC3biC3bHL}]+[\mathrm{hC3biC3bCR1}]+[\mathrm{hiC3biC3bCR1}]}\cdots \\
\frac{1}{+[\mathrm{hiC3bC3dgCR1}]+[\mathrm{fC4bCR1}]+[\mathrm{nfC4bC4BP}]+[\mathrm{fC4bC4BP}]+[\mathrm{fC4bC4bCR1}]+[\mathrm{fC4bC4bC4BP}]+[\mathrm{fC4bC4dCR1}]+[\mathrm{fC4bC4dC4BP}]+[\mathrm{hC4bCR1}]}\cdots \\
\frac{1}{+[\mathrm{hC4bC4BP}]+[\mathrm{hC4bC4bCR1}]+[\mathrm{hC4bC4bC4BP}]+[\mathrm{hC4bC4dCR1}]+[\mathrm{hC4bC4dC4BP}]}\cdots
\end{array}\right)
$$

S247

$$\frac{d[\mathrm{hC4bC4dCR1}]}{dt} = k^{+}_{\mathrm{C4bC4dCR1}}[\mathrm{hC4bC4d}][\mathrm{CR1}] - k^{-}_{\mathrm{C4bC4dCR1}}[\mathrm{hC4bC4dCR1}]$$

$$-\left(\frac{k^{\mathrm{FI}}_{\mathrm{catC3bH}}[\mathrm{I}][\mathrm{hC4bC4dCR1}]}{K^{\mathrm{FI}}_{\mathrm{mC3bH}} + [\mathrm{C3(H_2O)H}] + [\mathrm{C3(H_2O)HL}] + [\mathrm{fC3bH}] + [\mathrm{fC3bHL}] + [\mathrm{fC3bCR1}] + [\mathrm{fiC3bCR1}] + [\mathrm{IgGC3bC3bH}] + [\mathrm{IgGC3biC3bH}] + [\mathrm{IgGC3bC3bHL}]} \cdots\right.$$

$$\frac{1}{+ [\mathrm{IgGC3biC3bHL}] + [\mathrm{IgGC3bC3bCR1}] + [\mathrm{IgGC3biC3bCR1}] + [\mathrm{IgGiC3biC3bCR1}] + [\mathrm{IgGiC3bC3dgCR1}] + [\mathrm{fC3bC4bH}] + [\mathrm{fC3bC4bHL}]} \cdots$$

$$\frac{1}{+ [\mathrm{fC3bC4bCR1}] + [\mathrm{fC3bC4bC4BP}] + [\mathrm{fC3bC4dH}] + [\mathrm{fC3bC4dHL}] + [\mathrm{fC3bC4dCR1}] + [\mathrm{fiC3bC4dCR1}] + [\mathrm{fiC3bC4bCR1}] + [\mathrm{fiC3bC4bC4BP}]} \cdots$$

$$\frac{1}{+ [\mathrm{fC3dgC4bCR1}] + [\mathrm{fC3dgC4bC4BP}] + [\mathrm{fC3bC3bH}] + [\mathrm{fC3bC3bHL}] + [\mathrm{fC3bC3bCR1}] + [\mathrm{fC3biC3bH}] + [\mathrm{fC3biC3bHL}] + [\mathrm{fC3biC3bCR1}]} \cdots$$

$$\frac{1}{+ [\mathrm{fiC3biC3bCR1}] + [\mathrm{fiC3bC3dgCR1}] + [\mathrm{hC3bH}] + [\mathrm{hC3bHL}] + [\mathrm{hC3bCR1}] + [\mathrm{hiC3bCR1}] + [\mathrm{hC3bC4bH}] + [\mathrm{hC3bC4bHL}] + [\mathrm{hC3bC4bCR1}]} \cdots$$

$$\frac{1}{+ [\mathrm{hC3bC4bC4BP}] + [\mathrm{hC3bC4dH}] + [\mathrm{hC3bC4dHL}] + [\mathrm{hC3bC4dCR1}] + [\mathrm{hiC3bC4dCR1}] + [\mathrm{hiC3bC4bCR1}] + [\mathrm{hiC3bC4bC4BP}] + [\mathrm{hC3dgC4bCR1}]} \cdots$$

$$\frac{1}{+ [\mathrm{hC3dgC4bC4BP}] + [\mathrm{hC3bC3bH}] + [\mathrm{hC3bC3bHL}] + [\mathrm{hC3bC3bCR1}] + [\mathrm{hC3biC3bH}] + [\mathrm{hC3biC3bHL}] + [\mathrm{hC3biC3bCR1}] + [\mathrm{hiC3biC3bCR1}]} \cdots$$

$$\frac{1}{+ [\mathrm{hiC3bC3dgCR1}] + [\mathrm{fC4bCR1}] + [\mathrm{nfC4bC4BP}] + [\mathrm{fC4bC4BP}] + [\mathrm{fC4bC4bCR1}] + [\mathrm{fC4bC4bC4BP}] + [\mathrm{fC4bC4dCR1}] + [\mathrm{fC4bC4dC4BP}] + [\mathrm{hC4bCR1}]} \cdots$$

$$\left.\frac{1}{+ [\mathrm{hC4bC4BP}] + [\mathrm{hC4bC4bCR1}] + [\mathrm{hC4bC4bC4BP}] + [\mathrm{hC4bC4dCR1}] + [\mathrm{hC4bC4dC4BP}]} \cdots\right)$$

S248

$$\frac{d[\mathrm{hC4dC4d}]}{dt} =$$

$$\left(\frac{k^{\mathrm{FI}}_{\mathrm{catC3bH}}[\mathrm{I}][\mathrm{hC4bC4dC4BP}]}{K^{\mathrm{FI}}_{\mathrm{mC3bH}} + [\mathrm{C3(H_2O)H}] + [\mathrm{C3(H_2O)HL}] + [\mathrm{fC3bH}] + [\mathrm{fC3bHL}] + [\mathrm{fC3bCR1}] + [\mathrm{fiC3bCR1}] + [\mathrm{IgGC3bC3bH}] + [\mathrm{IgGC3biC3bH}] + [\mathrm{IgGC3bC3bHL}]} \cdots\right.$$

$$\frac{1}{+ [\mathrm{IgGC3biC3bHL}] + [\mathrm{IgGC3bC3bCR1}] + [\mathrm{IgGC3biC3bCR1}] + [\mathrm{IgGiC3biC3bCR1}] + [\mathrm{IgGiC3bC3dgCR1}] + [\mathrm{fC3bC4bH}] + [\mathrm{fC3bC4bHL}]} \cdots$$

$$\frac{1}{+ [\mathrm{fC3bC4bCR1}] + [\mathrm{fC3bC4bC4BP}] + [\mathrm{fC3bC4dH}] + [\mathrm{fC3bC4dHL}] + [\mathrm{fC3bC4dCR1}] + [\mathrm{fiC3bC4dCR1}] + [\mathrm{fiC3bC4bCR1}] + [\mathrm{fiC3bC4bC4BP}]} \cdots$$

$$\frac{1}{+ [\mathrm{fC3dgC4bCR1}] + [\mathrm{fC3dgC4bC4BP}] + [\mathrm{fC3bC3bH}] + [\mathrm{fC3bC3bHL}] + [\mathrm{fC3bC3bCR1}] + [\mathrm{fC3biC3bH}] + [\mathrm{fC3biC3bHL}] + [\mathrm{fC3biC3bCR1}]} \cdots$$

$$\frac{1}{+ [\mathrm{fiC3biC3bCR1}] + [\mathrm{fiC3bC3dgCR1}] + [\mathrm{hC3bH}] + [\mathrm{hC3bHL}] + [\mathrm{hC3bCR1}] + [\mathrm{hiC3bCR1}] + [\mathrm{hC3bC4bH}] + [\mathrm{hC3bC4bHL}] + [\mathrm{hC3bC4bCR1}]} \cdots$$

$$\frac{1}{+ [\mathrm{hC3bC4bC4BP}] + [\mathrm{hC3bC4dH}] + [\mathrm{hC3bC4dHL}] + [\mathrm{hC3bC4dCR1}] + [\mathrm{hiC3bC4dCR1}] + [\mathrm{hiC3bC4bCR1}] + [\mathrm{hiC3bC4bC4BP}] + [\mathrm{hC3dgC4bCR1}]} \cdots$$

$$\frac{1}{+ [\mathrm{hC3dgC4bC4BP}] + [\mathrm{hC3bC3bH}] + [\mathrm{hC3bC3bHL}] + [\mathrm{hC3bC3bCR1}] + [\mathrm{hC3biC3bH}] + [\mathrm{hC3biC3bHL}] + [\mathrm{hC3biC3bCR1}] + [\mathrm{hiC3biC3bCR1}]} \cdots$$

$$\frac{1}{+ [\mathrm{hiC3bC3dgCR1}] + [\mathrm{fC4bCR1}] + [\mathrm{nfC4bC4BP}] + [\mathrm{fC4bC4BP}] + [\mathrm{fC4bC4bCR1}] + [\mathrm{fC4bC4bC4BP}] + [\mathrm{fC4bC4dCR1}] + [\mathrm{fC4bC4dC4BP}] + [\mathrm{hC4bCR1}]} \cdots$$

$$\left.\frac{1}{+ [\mathrm{hC4bC4BP}] + [\mathrm{hC4bC4bCR1}] + [\mathrm{hC4bC4bC4BP}] + [\mathrm{hC4bC4dCR1}] + [\mathrm{hC4bC4dC4BP}]} \cdots\right)$$

$$+\left(\begin{array}{l}\frac{k^{\mathrm{FI}}_{\mathrm{catC3bH}}[\mathrm{I}][\mathrm{hC4bC4dCR1}]}{K^{\mathrm{FI}}_{\mathrm{mC3bH}}+[\mathrm{C3(H_2O)H}]+[\mathrm{C3(H_2O)HL}]+[\mathrm{fC3bH}]+[\mathrm{fC3bHL}]+[\mathrm{fC3bCR1}]+[\mathrm{fiC3bCR1}]+[\mathrm{IgGC3bC3bH}]+[\mathrm{IgGC3biC3bH}]+[\mathrm{IgGC3bC3bHL}]}\cdots \\ \frac{1}{+[\mathrm{IgGC3biC3bHL}]+[\mathrm{IgGC3bC3bCR1}]+[\mathrm{IgGC3biC3bCR1}]+[\mathrm{IgGiC3biC3bCR1}]+[\mathrm{IgGiC3bC3dgCR1}]+[\mathrm{fC3bC4bH}]+[\mathrm{fC3bC4bHL}]}\cdots \\ \frac{1}{+[\mathrm{fC3bC4bCR1}]+[\mathrm{fC3bC4bC4BP}]+[\mathrm{fC3bC4dH}]+[\mathrm{fC3bC4dHL}]+[\mathrm{fC3bC4dCR1}]+[\mathrm{fiC3bC4dCR1}]+[\mathrm{fiC3bC4bCR1}]+[\mathrm{fiC3bC4bC4BP}]}\cdots \\ \frac{1}{+[\mathrm{fC3dgC4bCR1}]+[\mathrm{fC3dgC4bC4BP}]+[\mathrm{fC3bC3bH}]+[\mathrm{fC3bC3bHL}]+[\mathrm{fC3bC3bCR1}]+[\mathrm{fC3biC3bH}]+[\mathrm{fC3biC3bHL}]+[\mathrm{fC3biC3bCR1}]}\cdots \\ \frac{1}{+[\mathrm{fiC3biC3bCR1}]+[\mathrm{fiC3bC3dgCR1}]+[\mathrm{hC3bH}]+[\mathrm{hC3bHL}]+[\mathrm{hC3bCR1}]+[\mathrm{hiC3bCR1}]+[\mathrm{hC3bC4bH}]+[\mathrm{hC3bC4bHL}]+[\mathrm{hC3bC4bCR1}]}\cdots \\ \frac{1}{+[\mathrm{hC3bC4bC4BP}]+[\mathrm{hC3bC4dH}]+[\mathrm{hC3bC4dHL}]+[\mathrm{hC3bC4dCR1}]+[\mathrm{hiC3bC4dCR1}]+[\mathrm{hiC3bC4bCR1}]+[\mathrm{hiC3bC4bC4BP}]+[\mathrm{hC3dgC4bCR1}]}\cdots \\ \frac{1}{+[\mathrm{hC3dgC4bC4BP}]+[\mathrm{hC3bC3bH}]+[\mathrm{hC3bC3bHL}]+[\mathrm{hC3bC3bCR1}]+[\mathrm{hC3biC3bH}]+[\mathrm{hC3biC3bHL}]+[\mathrm{hC3biC3bCR1}]+[\mathrm{hiC3biC3bCR1}]}\cdots \\ \frac{1}{+[\mathrm{hiC3bC3dgCR1}]+[\mathrm{fC4bCR1}]+[\mathrm{nfC4bC4BP}]+[\mathrm{fC4bC4BP}]+[\mathrm{fC4bC4bCR1}]+[\mathrm{fC4bC4bC4BP}]+[\mathrm{fC4bC4dCR1}]+[\mathrm{fC4bC4dC4BP}]+[\mathrm{hC4bCR1}]}\cdots \\ \frac{1}{+[\mathrm{hC4bC4BP}]+[\mathrm{hC4bC4bCR1}]+[\mathrm{hC4bC4bC4BP}]+[\mathrm{hC4bC4dCR1}]+[\mathrm{hC4bC4dC4BP}]}\cdots\end{array}\right)$$

S249

$$\frac{d[\mathrm{hC5b8CD59}]}{dt}=k^{+}_{\mathrm{C5b8CD59}}[\mathrm{hC5b8}][\mathrm{CD59}]-k^{-}_{\mathrm{C5b8CD59}}[\mathrm{hC5b8CD59}]$$

S250

$$\frac{d[\mathrm{hC5b9_1CD59}]}{dt}=k^{+}_{\mathrm{C5b9_1CD59}}[\mathrm{hC5b9_1}][\mathrm{CD59}]-k^{-}_{\mathrm{C5b9_1CD59}}[\mathrm{hC5b9_1CD59}]$$

S251

$$\frac{d[\mathrm{VnfC5b7}]}{dt}=k^{+}_{\mathrm{VnC5b7}}[\mathrm{fC5b7}][\mathrm{Vn}]-k^{-}_{\mathrm{VnC5b7}}[\mathrm{VnfC5b7}]-k^{+}_{\mathrm{C5b8}}[\mathrm{VnfC5b7}][\mathrm{C8}]+k^{-}_{\mathrm{C5b8}}[\mathrm{VnfC5b8}]$$

S252

$$\frac{d[\mathrm{VnfC5b8}]}{dt}=k^{+}_{\mathrm{C5b8}}[\mathrm{VnfC5b7}][\mathrm{C8}]-k^{-}_{\mathrm{C5b8}}[\mathrm{VnfC5b8}]+k^{+}_{\mathrm{VnC5b8}}[\mathrm{fC5b8}][\mathrm{Vn}]-k^{-}_{\mathrm{VnC5b8}}[\mathrm{VnfC5b8}]-k^{+}_{\mathrm{C5b9}}[\mathrm{VnfC5b8}][\mathrm{C9_1}]$$
$$+k^{-}_{\mathrm{C5b9}}[\mathrm{VnfC5b9_1}]$$

S253

$$\frac{d[\mathrm{VnfC5b9_1}]}{dt}=k^{+}_{\mathrm{C5b9}}[\mathrm{VnfC5b8}][\mathrm{C9_1}]-k^{-}_{\mathrm{C5b9}}[\mathrm{VnfC5b9_1}]+k^{+}_{\mathrm{VnC5b9}}[\mathrm{fC5b9_1}][\mathrm{Vn}]-k^{-}_{\mathrm{VnC5b9}}[\mathrm{VnfC5b9_1}]$$

S254

$$\frac{d[\mathrm{CnfC5b7}]}{dt}=k^{+}_{\mathrm{CnC5b7}}[\mathrm{fC5b7}][\mathrm{Cn}]-k^{-}_{\mathrm{CnC5b7}}[\mathrm{CnfC5b7}]-k^{+}_{\mathrm{C5b8}}[\mathrm{CnfC5b7}][\mathrm{C8}]+k^{-}_{\mathrm{C5b8}}[\mathrm{CnfC5b8}]$$

S255

$$\frac{d[\mathrm{CnfC5b8}]}{dt}=k^{+}_{\mathrm{C5b8}}[\mathrm{CnfC5b7}][\mathrm{C8}]-k^{-}_{\mathrm{C5b8}}[\mathrm{CnfC5b8}]+k^{+}_{\mathrm{CnC5b8}}[\mathrm{fC5b8}][\mathrm{Cn}]-k^{-}_{\mathrm{CnC5b8}}[\mathrm{CnfC5b8}]-k^{+}_{\mathrm{C5b9}}[\mathrm{CnfC5b8}][\mathrm{C9}_1]+k^{-}_{\mathrm{C5b9}}[\mathrm{CnfC5b9}_1]$$

S256

$$\frac{d[\mathrm{CnfC5b9}_1]}{dt}=k^{+}_{\mathrm{C5b9}}[\mathrm{CnfC5b8}][\mathrm{C9}_1]-k^{-}_{\mathrm{C5b9}}[\mathrm{CnfC5b9}_1]+k^{+}_{\mathrm{CnC5b9}}[\mathrm{fC5b9}_1][\mathrm{Cn}]-k^{-}_{\mathrm{CnC5b9}}[\mathrm{CnfC5b9}_1]$$

(v) Complement Proteins (host cell and fluid state)

S257

$$\frac{d[\mathrm{C3}]}{dt}=-k^{+}_{\mathrm{C3(H_2O)}}[\mathrm{C3}]-\frac{k^{\mathrm{C3}}_{\mathrm{catC3(H_2O)Bb}}[\mathrm{C3}][\mathrm{C3(H_2O)Bb}]}{K^{\mathrm{C3}}_{\mathrm{mC3(H_2O)Bb}}+[\mathrm{C3}]}-\frac{k^{\mathrm{C3}}_{\mathrm{catC3bBb}}[\mathrm{C3}][\mathrm{fC3bBb}]}{K^{\mathrm{C3}}_{\mathrm{mC3bBb}}\left(1+\frac{[\mathrm{C3}]}{K^{\mathrm{C3}}_{\mathrm{mC3bBb}}}+\frac{[\mathrm{C5}]}{K^{\mathrm{C5}}_{\mathrm{mC3bBb}}}\right)}$$

$$-\frac{k^{\mathrm{C3}}_{\mathrm{catC3bBb}}[\mathrm{C3}][\mathrm{hC3bBb}]}{K^{\mathrm{C3}}_{\mathrm{mC3bBb}}\left(1+\frac{[\mathrm{C3}]}{K^{\mathrm{C3}}_{\mathrm{mC3bBb}}}+\frac{[\mathrm{C5}]}{K^{\mathrm{C5}}_{\mathrm{mC3bBb}}}\right)}-\frac{k^{\mathrm{C3}}_{\mathrm{catC3bBb}}[\mathrm{C3}][\mathrm{hC3bBbP}]}{K^{\mathrm{C3}}_{\mathrm{mC3bBb}}\left(1+\frac{[\mathrm{C3}]}{K^{\mathrm{C3}}_{\mathrm{mC3bBb}}}+\frac{[\mathrm{C5}]}{K^{\mathrm{C5}}_{\mathrm{mC3bBb}}}\right)}-\frac{k^{\mathrm{C3}}_{\mathrm{catC3bBb}}[\mathrm{C3}][\mathrm{IgGC3bC3bBb}]}{K^{\mathrm{C3}}_{\mathrm{mC3bBb}}+[\mathrm{C3}]}$$

$$-\frac{k^{\mathrm{C3}}_{\mathrm{catC3bBb}}[\mathrm{C3}][\mathrm{IgGC3bC3bBbP}]}{K^{\mathrm{C3}}_{\mathrm{mC3bBb}}+[\mathrm{C3}]}-\frac{k^{\mathrm{C3}}_{\mathrm{catC3bBb}}[\mathrm{C3}][\mathrm{fC4bC2a}]}{K^{\mathrm{C3}}_{\mathrm{mC3bBb}}\left(1+\frac{[\mathrm{C3}]}{K^{\mathrm{C3}}_{\mathrm{mC4bC2a}}}+\frac{[\mathrm{C5}]}{K^{\mathrm{C5}}_{\mathrm{mC4bC2a}}}\right)}$$

$$-\frac{k^{\mathrm{C3}}_{\mathrm{catC3bBb}}[\mathrm{C3}][\mathrm{fC3bC3bBb}]}{K^{\mathrm{C3}}_{\mathrm{mC3bBb}}\left(1+\frac{[\mathrm{C3}]}{K^{\mathrm{C3}}_{\mathrm{mC3bBb}}}+\frac{[\mathrm{C5}]}{K^{\mathrm{C5}}_{\mathrm{mC3bC3bBb}}}\right)}-\frac{k^{\mathrm{C3}}_{\mathrm{catC3bBb}}[\mathrm{C3}][\mathrm{fC3bC3bBbP}]}{K^{\mathrm{C3}}_{\mathrm{mC3bBb}}\left(1+\frac{[\mathrm{C3}]}{K^{\mathrm{C3}}_{\mathrm{mC3bBb}}}+\frac{[\mathrm{C5}]}{K^{\mathrm{C5}}_{\mathrm{mC3bC3bBb}}}\right)}$$

$$-\frac{k^{\mathrm{C3}}_{\mathrm{catC3bBb}}[\mathrm{C3}][\mathrm{fC3bC4bBb}]}{K^{\mathrm{C3}}_{\mathrm{mC3bBb}}\left(1+\frac{[\mathrm{C3}]}{K^{\mathrm{C3}}_{\mathrm{mC3bBb}}}+\frac{[\mathrm{C5}]}{K^{\mathrm{C5}}_{\mathrm{mC3bC4bBb}}}\right)}-\frac{k^{\mathrm{C3}}_{\mathrm{catC3bBb}}[\mathrm{C3}][\mathrm{fC3bC4bBbP}]}{K^{\mathrm{C3}}_{\mathrm{mC3bBb}}\left(1+\frac{[\mathrm{C3}]}{K^{\mathrm{C3}}_{\mathrm{mC3bBb}}}+\frac{[\mathrm{C5}]}{K^{\mathrm{C5}}_{\mathrm{mC3bC4bBb}}}\right)}$$

$$-\frac{k^{\mathrm{C3}}_{\mathrm{catC4bC2a}}[\mathrm{C3}][\mathrm{fC3bC4bC2a}]}{K^{\mathrm{C3}}_{\mathrm{mC4bC2a}}\left(1+\frac{[\mathrm{C3}]}{K^{\mathrm{C3}}_{\mathrm{mC4bC2a}}}+\frac{[\mathrm{C5}]}{K^{\mathrm{C5}}_{\mathrm{mC3bC4bC2a}}}\right)}-\frac{k^{\mathrm{C3}}_{\mathrm{catC4bC2a}}[\mathrm{C3}][\mathrm{fC4bC4bC2a}]}{K^{\mathrm{C3}}_{\mathrm{mC4bC2a}}\left(1+\frac{[\mathrm{C3}]}{K^{\mathrm{C3}}_{\mathrm{mC4bC2a}}}+\frac{[\mathrm{C5}]}{K^{\mathrm{C5}}_{\mathrm{mC4bC4bC2a}}}\right)}$$

$$-\frac{k^{\mathrm{C3}}_{\mathrm{catC3bBb}}[\mathrm{C3}][\mathrm{hC3bC3bBb}]}{K^{\mathrm{C3}}_{\mathrm{mC3bBb}}\left(1+\frac{[\mathrm{C3}]}{K^{\mathrm{C3}}_{\mathrm{mC3bBb}}}+\frac{[\mathrm{C5}]}{K^{\mathrm{C5}}_{\mathrm{mC3bC3bBb}}}\right)}-\frac{k^{\mathrm{C3}}_{\mathrm{catC3bBb}}[\mathrm{C3}][\mathrm{hC3bC3bBbP}]}{K^{\mathrm{C3}}_{\mathrm{mC3bBb}}\left(1+\frac{[\mathrm{C3}]}{K^{\mathrm{C3}}_{\mathrm{mC3bBb}}}+\frac{[\mathrm{C5}]}{K^{\mathrm{C5}}_{\mathrm{mC3bC3bBb}}}\right)}$$

$$-\frac{k^{\mathrm{C3}}_{\mathrm{catC3bBb}}[\mathrm{C3}][\mathrm{hC3bC4bBb}]}{K^{\mathrm{C3}}_{\mathrm{mC3bBb}}\left(1+\frac{[\mathrm{C3}]}{K^{\mathrm{C3}}_{\mathrm{mC3bBb}}}+\frac{[\mathrm{C5}]}{K^{\mathrm{C5}}_{\mathrm{mC3bC4bBb}}}\right)}-\frac{k^{\mathrm{C3}}_{\mathrm{catC3bBb}}[\mathrm{C3}][\mathrm{hC3bC4bBbP}]}{K^{\mathrm{C3}}_{\mathrm{mC3bBb}}\left(1+\frac{[\mathrm{C3}]}{K^{\mathrm{C3}}_{\mathrm{mC3bBb}}}+\frac{[\mathrm{C5}]}{K^{\mathrm{C5}}_{\mathrm{mC3bC4bBb}}}\right)}$$

S258

$$\frac{d[\mathrm{C3a}]}{dt}=\frac{k_{\mathrm{catC3(H_2O)Bb}}^{\mathrm{C3}}[\mathrm{C3}][\mathrm{C3(H_2O)Bb}]}{K_{\mathrm{mC3(H_2O)Bb}}^{\mathrm{C3}}+[\mathrm{C3}]}+\frac{k_{\mathrm{catC3bBb}}^{\mathrm{C3}}[\mathrm{C3}][\mathrm{fC3bBb}]}{K_{\mathrm{mC3bBb}}^{\mathrm{C3}}\left(1+\frac{[\mathrm{C3}]}{K_{\mathrm{mC3bBb}}^{\mathrm{C3}}}+\frac{[\mathrm{C5}]}{K_{\mathrm{mC3bBb}}^{\mathrm{C5}}}\right)}+\frac{k_{\mathrm{catC3bBb}}^{\mathrm{C3}}[\mathrm{C3}][\mathrm{hC3bBb}]}{K_{\mathrm{mC3bBb}}^{\mathrm{C3}}\left(1+\frac{[\mathrm{C3}]}{K_{\mathrm{mC3bBb}}^{\mathrm{C3}}}+\frac{[\mathrm{C5}]}{K_{\mathrm{mC3bBb}}^{\mathrm{C5}}}\right)}$$

$$+\frac{k_{\mathrm{catC3bBb}}^{\mathrm{C3}}[\mathrm{C3}][\mathrm{hC3bBbP}]}{K_{\mathrm{mC3bBb}}^{\mathrm{C3}}\left(1+\frac{[\mathrm{C3}]}{K_{\mathrm{mC3bBb}}^{\mathrm{C3}}}+\frac{[\mathrm{C5}]}{K_{\mathrm{mC3bBb}}^{\mathrm{C5}}}\right)}+\frac{k_{\mathrm{catC3bBb}}^{\mathrm{C3}}[\mathrm{C3}][\mathrm{IgGC3bC3bBb}]}{K_{\mathrm{mC3bBb}}^{\mathrm{C3}}+[\mathrm{C3}]}+\frac{k_{\mathrm{catC3bBb}}^{\mathrm{C3}}[\mathrm{C3}][\mathrm{IgGC3bC3bBbP}]}{K_{\mathrm{mC3bBb}}^{\mathrm{C3}}+[\mathrm{C3}]}$$

$$+\frac{k_{\mathrm{catC3bBb}}^{\mathrm{C3}}[\mathrm{C3}][\mathrm{fC4bC2a}]}{K_{\mathrm{mC3bBb}}^{\mathrm{C3}}\left(1+\frac{[\mathrm{C3}]}{K_{\mathrm{mC4bC2a}}^{\mathrm{C3}}}+\frac{[\mathrm{C5}]}{K_{\mathrm{mC4bC2a}}^{\mathrm{C5}}}\right)}+\frac{k_{\mathrm{catC3bBb}}^{\mathrm{C3}}[\mathrm{C3}][\mathrm{fC3bC3bBb}]}{K_{\mathrm{mC3bBb}}^{\mathrm{C3}}\left(1+\frac{[\mathrm{C3}]}{K_{\mathrm{mC3bBb}}^{\mathrm{C3}}}+\frac{[\mathrm{C5}]}{K_{\mathrm{mC3bC3bBb}}^{\mathrm{C5}}}\right)}$$

$$+\frac{k_{\mathrm{catC3bBb}}^{\mathrm{C3}}[\mathrm{C3}][\mathrm{fC3bC3bBbP}]}{K_{\mathrm{mC3bBb}}^{\mathrm{C3}}\left(1+\frac{[\mathrm{C3}]}{K_{\mathrm{mC3bBb}}^{\mathrm{C3}}}+\frac{[\mathrm{C5}]}{K_{\mathrm{mC3bC3bBb}}^{\mathrm{C5}}}\right)}+\frac{k_{\mathrm{catC3bBb}}^{\mathrm{C3}}[\mathrm{C3}][\mathrm{fC3bC4bBb}]}{K_{\mathrm{mC3bBb}}^{\mathrm{C3}}\left(1+\frac{[\mathrm{C3}]}{K_{\mathrm{mC3bBb}}^{\mathrm{C3}}}+\frac{[\mathrm{C5}]}{K_{\mathrm{mC3bC4bBb}}^{\mathrm{C5}}}\right)}$$

$$+\frac{k_{\mathrm{catC3bBb}}^{\mathrm{C3}}[\mathrm{C3}][\mathrm{fC3bC4bBbP}]}{K_{\mathrm{mC3bBb}}^{\mathrm{C3}}\left(1+\frac{[\mathrm{C3}]}{K_{\mathrm{mC3bBb}}^{\mathrm{C3}}}+\frac{[\mathrm{C5}]}{K_{\mathrm{mC3bC4bBb}}^{\mathrm{C5}}}\right)}+\frac{k_{\mathrm{catC4bC2a}}^{\mathrm{C3}}[\mathrm{C3}][\mathrm{fC3bC4bC2a}]}{K_{\mathrm{mC4bC2a}}^{\mathrm{C3}}\left(1+\frac{[\mathrm{C3}]}{K_{\mathrm{mC4bC2a}}^{\mathrm{C3}}}+\frac{[\mathrm{C5}]}{K_{\mathrm{mC3bC4bC2a}}^{\mathrm{C5}}}\right)}$$

$$+\frac{k_{\mathrm{catC4bC2a}}^{\mathrm{C3}}[\mathrm{C3}][\mathrm{fC4bC4bC2a}]}{K_{\mathrm{mC4bC2a}}^{\mathrm{C3}}\left(1+\frac{[\mathrm{C3}]}{K_{\mathrm{mC4bC2a}}^{\mathrm{C3}}}+\frac{[\mathrm{C5}]}{K_{\mathrm{mC4bC4bC2a}}^{\mathrm{C5}}}\right)}+\frac{k_{\mathrm{catC3bBb}}^{\mathrm{C3}}[\mathrm{C3}][\mathrm{hC3bC3bBb}]}{K_{\mathrm{mC3bBb}}^{\mathrm{C3}}\left(1+\frac{[\mathrm{C3}]}{K_{\mathrm{mC3bBb}}^{\mathrm{C3}}}+\frac{[\mathrm{C5}]}{K_{\mathrm{mC3bC3bBb}}^{\mathrm{C5}}}\right)}$$

$$+\frac{k_{\mathrm{catC3bBb}}^{\mathrm{C3}}[\mathrm{C3}][\mathrm{hC3bC3bBbP}]}{K_{\mathrm{mC3bBb}}^{\mathrm{C3}}\left(1+\frac{[\mathrm{C3}]}{K_{\mathrm{mC3bBb}}^{\mathrm{C3}}}+\frac{[\mathrm{C5}]}{K_{\mathrm{mC3bC3bBb}}^{\mathrm{C5}}}\right)}+\frac{k_{\mathrm{catC3bBb}}^{\mathrm{C3}}[\mathrm{C3}][\mathrm{hC3bC4bBb}]}{K_{\mathrm{mC3bBb}}^{\mathrm{C3}}\left(1+\frac{[\mathrm{C3}]}{K_{\mathrm{mC3bBb}}^{\mathrm{C3}}}+\frac{[\mathrm{C5}]}{K_{\mathrm{mC3bC4bBb}}^{\mathrm{C5}}}\right)}$$

$$+\frac{k_{\mathrm{catC3bBb}}^{\mathrm{C3}}[\mathrm{C3}][\mathrm{hC3bC4bBbP}]}{K_{\mathrm{mC3bBb}}^{\mathrm{C3}}\left(1+\frac{[\mathrm{C3}]}{K_{\mathrm{mC3bBb}}^{\mathrm{C3}}}+\frac{[\mathrm{C5}]}{K_{\mathrm{mC3bC4bBb}}^{\mathrm{C5}}}\right)}-\frac{k_{\mathrm{catCPN}}^{\mathrm{C3a}}[\mathrm{C3a}][\mathrm{CPN}]}{K_{\mathrm{mCPN}}^{\mathrm{C3a}}\left(1+\frac{[\mathrm{C3a}]}{K_{\mathrm{mCPN}}^{\mathrm{C3a}}}+\frac{[\mathrm{C5a}]}{K_{\mathrm{mCPN}}^{\mathrm{C5a}}}\right)}$$

S259

$$\frac{d[\mathrm{C3adesArg}]}{dt}=\frac{k_{\mathrm{catCPN}}^{\mathrm{C3a}}[\mathrm{C3a}][\mathrm{CPN}]}{K_{\mathrm{mCPN}}^{\mathrm{C3a}}\left(1+\frac{[\mathrm{C3a}]}{K_{\mathrm{mCPN}}^{\mathrm{C3a}}}+\frac{[\mathrm{C5a}]}{K_{\mathrm{mCPN}}^{\mathrm{C5a}}}\right)}$$

S260

$$\frac{d[\mathrm{C4}]}{dt}=-\frac{k_{\mathrm{catC1*}}^{\mathrm{C4}}[\mathrm{C4}][\mathrm{C1*}]}{K_{\mathrm{mC1*}}^{\mathrm{C4}}\left(1+\frac{[\mathrm{fC4bC2}]}{K_{\mathrm{mC1*}}^{\mathrm{C2}}}+\frac{[\mathrm{fC4bC4bC2}]}{K_{\mathrm{mC1*}}^{\mathrm{C2}}}+\frac{[\mathrm{fC3bC4bC2}]}{K_{\mathrm{mC1*}}^{\mathrm{C2}}}+\frac{[\mathrm{C4}]}{K_{\mathrm{mC1*}}^{\mathrm{C4}}}\right)}$$

S261

$$\frac{d[\mathrm{C4a}]}{dt}=\frac{k_{\mathrm{catC1*}}^{\mathrm{C4}}[\mathrm{C4}][\mathrm{C1*}]}{K_{\mathrm{mC1*}}^{\mathrm{C4}}\left(1+\frac{[\mathrm{fC4bC2}]}{K_{\mathrm{mC1*}}^{\mathrm{C2}}}+\frac{[\mathrm{fC4bC4bC2}]}{K_{\mathrm{mC1*}}^{\mathrm{C2}}}+\frac{[\mathrm{fC3bC4bC2}]}{K_{\mathrm{mC1*}}^{\mathrm{C2}}}+\frac{[\mathrm{C4}]}{K_{\mathrm{mC1*}}^{\mathrm{C4}}}\right)}$$

S262

$$\frac{d[\mathrm{C5}]}{dt}=-\frac{k_{\mathrm{catC3bBb}}^{\mathrm{C5}}[\mathrm{C5}][\mathrm{fC3bBb}]}{K_{\mathrm{mC3bBb}}^{\mathrm{C5}}\left(1+\frac{[\mathrm{C3}]}{K_{\mathrm{mC3bBb}}^{\mathrm{C3}}}+\frac{[\mathrm{C5}]}{K_{\mathrm{mC3bBb}}^{\mathrm{C5}}}\right)}-\frac{k_{\mathrm{catC4bC2a}}^{\mathrm{C5}}[\mathrm{C5}][\mathrm{fC4bC2a}]}{K_{\mathrm{mC4bC2a}}^{\mathrm{C5}}\left(1+\frac{[\mathrm{C3}]}{K_{\mathrm{mC4bC2a}}^{\mathrm{C3}}}+\frac{[\mathrm{C5}]}{K_{\mathrm{mC4bC2a}}^{\mathrm{C5}}}\right)}$$

$$-\frac{k_{\mathrm{catC3bC3bBb}}^{\mathrm{C5}}[\mathrm{C5}][\mathrm{fC3bC3bBb}]}{K_{\mathrm{mC3bC3bBb}}^{\mathrm{C5}}\left(1+\frac{[\mathrm{C3}]}{K_{\mathrm{mC3bBb}}^{\mathrm{C3}}}+\frac{[\mathrm{C5}]}{K_{\mathrm{mC3bC3bBb}}^{\mathrm{C5}}}\right)}-\frac{k_{\mathrm{catC3bC3bBb}}^{\mathrm{C5}}[\mathrm{C5}][\mathrm{fC3bC3bBbP}]}{K_{\mathrm{mC3bC3bBb}}^{\mathrm{C5}}\left(1+\frac{[\mathrm{C3}]}{K_{\mathrm{mC3bBb}}^{\mathrm{C3}}}+\frac{[\mathrm{C5}]}{K_{\mathrm{mC3bC3bBb}}^{\mathrm{C5}}}\right)}$$

$$-\frac{k_{\mathrm{catC3bC4bBb}}^{\mathrm{C5}}[\mathrm{C5}][\mathrm{fC3bC4bBb}]}{K_{\mathrm{mC3bC4bBb}}^{\mathrm{C5}}\left(1+\frac{[\mathrm{C3}]}{K_{\mathrm{mC3bBb}}^{\mathrm{C3}}}+\frac{[\mathrm{C5}]}{K_{\mathrm{mC3bC4bBb}}^{\mathrm{C5}}}\right)}-\frac{k_{\mathrm{catC3bC4bBb}}^{\mathrm{C5}}[\mathrm{C5}][\mathrm{fC3bC4bBbP}]}{K_{\mathrm{mC3bC4bBb}}^{\mathrm{C5}}\left(1+\frac{[\mathrm{C3}]}{K_{\mathrm{mC3bBb}}^{\mathrm{C3}}}+\frac{[\mathrm{C5}]}{K_{\mathrm{mC3bC4bBb}}^{\mathrm{C5}}}\right)}$$

$$-\frac{k_{\mathrm{catC3bC4bC2a}}^{\mathrm{C5}}[\mathrm{C5}][\mathrm{fC3bC4bC2a}]}{K_{\mathrm{mC3bC4bC2a}}^{\mathrm{C5}}\left(1+\frac{[\mathrm{C3}]}{K_{\mathrm{mC4bC2a}}^{\mathrm{C3}}}+\frac{[\mathrm{C5}]}{K_{\mathrm{mC3bC4bC2a}}^{\mathrm{C5}}}\right)}-\frac{k_{\mathrm{catC4bC4bC2a}}^{\mathrm{C5}}[\mathrm{C5}][\mathrm{fC4bC4bC2a}]}{K_{\mathrm{mC4bC4bC2a}}^{\mathrm{C5}}\left(1+\frac{[\mathrm{C3}]}{K_{\mathrm{mC4bC2a}}^{\mathrm{C3}}}+\frac{[\mathrm{C5}]}{K_{\mathrm{mC4bC4bC2a}}^{\mathrm{C5}}}\right)}$$

$$-\frac{k_{\mathrm{catC3bBb}}^{\mathrm{C5}}[\mathrm{C5}][\mathrm{hC3bBb}]}{K_{\mathrm{mC3bBb}}^{\mathrm{C5}}\left(1+\frac{[\mathrm{C3}]}{K_{\mathrm{mC3bBb}}^{\mathrm{C3}}}+\frac{[\mathrm{C5}]}{K_{\mathrm{mC3bBb}}^{\mathrm{C5}}}\right)}-\frac{k_{\mathrm{catC3bBb}}^{\mathrm{C5}}[\mathrm{C5}][\mathrm{hC3bBbP}]}{K_{\mathrm{mC3bBb}}^{\mathrm{C5}}\left(1+\frac{[\mathrm{C3}]}{K_{\mathrm{mC3bBb}}^{\mathrm{C3}}}+\frac{[\mathrm{C5}]}{K_{\mathrm{mC3bBb}}^{\mathrm{C5}}}\right)}$$

$$-\frac{k_{\mathrm{catC3bC3bBb}}^{\mathrm{C5}}[\mathrm{C5}][\mathrm{hC3bC3bBb}]}{K_{\mathrm{mC3bC3bBb}}^{\mathrm{C5}}\left(1+\frac{[\mathrm{C3}]}{K_{\mathrm{mC3bBb}}^{\mathrm{C3}}}+\frac{[\mathrm{C5}]}{K_{\mathrm{mC3bC3bBb}}^{\mathrm{C5}}}\right)}-\frac{k_{\mathrm{catC3bC3bBb}}^{\mathrm{C5}}[\mathrm{C5}][\mathrm{hC3bC3bBbP}]}{K_{\mathrm{mC3bC3bBb}}^{\mathrm{C5}}\left(1+\frac{[\mathrm{C3}]}{K_{\mathrm{mC3bBb}}^{\mathrm{C3}}}+\frac{[\mathrm{C5}]}{K_{\mathrm{mC3bC3bBb}}^{\mathrm{C5}}}\right)}$$

$$-\frac{k_{\mathrm{catC3bC4bBb}}^{\mathrm{C5}}[\mathrm{C5}][\mathrm{hC3bC4bBb}]}{K_{\mathrm{mC3bC4bBb}}^{\mathrm{C5}}\left(1+\frac{[\mathrm{C3}]}{K_{\mathrm{mC3bBb}}^{\mathrm{C3}}}+\frac{[\mathrm{C5}]}{K_{\mathrm{mC3bC4bBb}}^{\mathrm{C5}}}\right)}-\frac{k_{\mathrm{catC3bC4bBb}}^{\mathrm{C5}}[\mathrm{C5}][\mathrm{hC3bC4bBbP}]}{K_{\mathrm{mC3bC4bBb}}^{\mathrm{C5}}\left(1+\frac{[\mathrm{C3}]}{K_{\mathrm{mC3bBb}}^{\mathrm{C3}}}+\frac{[\mathrm{C5}]}{K_{\mathrm{mC3bC4bBb}}^{\mathrm{C5}}}\right)}$$

S263

$$\frac{d[\text{C5a}]}{dt}=\frac{k_{\text{catC3bBb}}^{\text{C5}}[\text{C5}][\text{fC3bBb}]}{K_{\text{mC3bBb}}^{\text{C5}}\left(1+\frac{[\text{C3}]}{K_{\text{mC3bBb}}^{\text{C3}}}+\frac{[\text{C5}]}{K_{\text{mC3bBb}}^{\text{C5}}}\right)}+\frac{k_{\text{catC4bC2a}}^{\text{C5}}[\text{C5}][\text{fC4bC2a}]}{K_{\text{mC4bC2a}}^{\text{C5}}\left(1+\frac{[\text{C3}]}{K_{\text{mC4bC2a}}^{\text{C3}}}+\frac{[\text{C5}]}{K_{\text{mC4bC2a}}^{\text{C5}}}\right)}$$

$$+\frac{k_{\text{catC3bC3bBb}}^{\text{C5}}[\text{C5}][\text{fC3bC3bBb}]}{K_{\text{mC3bC3bBb}}^{\text{C5}}\left(1+\frac{[\text{C3}]}{K_{\text{mC3bBb}}^{\text{C3}}}+\frac{[\text{C5}]}{K_{\text{mC3bC3bBb}}^{\text{C5}}}\right)}+\frac{k_{\text{catC3bC3bBb}}^{\text{C5}}[\text{C5}][\text{fC3bC3bBbP}]}{K_{\text{mC3bC3bBb}}^{\text{C5}}\left(1+\frac{[\text{C3}]}{K_{\text{mC3bBb}}^{\text{C3}}}+\frac{[\text{C5}]}{K_{\text{mC3bC3bBb}}^{\text{C5}}}\right)}$$

$$+\frac{k_{\text{catC3bC4bBb}}^{\text{C5}}[\text{C5}][\text{fC3bC4bBb}]}{K_{\text{mC3bC4bBb}}^{\text{C5}}\left(1+\frac{[\text{C3}]}{K_{\text{mC3bBb}}^{\text{C3}}}+\frac{[\text{C5}]}{K_{\text{mC3bC4bBb}}^{\text{C5}}}\right)}+\frac{k_{\text{catC3bC4bBb}}^{\text{C5}}[\text{C5}][\text{fC3bC4bBbP}]}{K_{\text{mC3bC4bBb}}^{\text{C5}}\left(1+\frac{[\text{C3}]}{K_{\text{mC3bBb}}^{\text{C3}}}+\frac{[\text{C5}]}{K_{\text{mC3bC4bBb}}^{\text{C5}}}\right)}$$

$$+\frac{k_{\text{catC3bC4bC2a}}^{\text{C5}}[\text{C5}][\text{fC3bC4bC2a}]}{K_{\text{mC3bC4bC2a}}^{\text{C5}}\left(1+\frac{[\text{C3}]}{K_{\text{mC4bC2a}}^{\text{C3}}}+\frac{[\text{C5}]}{K_{\text{mC3bC4bC2a}}^{\text{C5}}}\right)}+\frac{k_{\text{catC4bC4bC2a}}^{\text{C5}}[\text{C5}][\text{fC4bC4bC2a}]}{K_{\text{mC4bC4bC2a}}^{\text{C5}}\left(1+\frac{[\text{C3}]}{K_{\text{mC4bC2a}}^{\text{C3}}}+\frac{[\text{C5}]}{K_{\text{mC4bC4bC2a}}^{\text{C5}}}\right)}$$

$$+\frac{k_{\text{catC3bBb}}^{\text{C5}}[\text{C5}][\text{hC3bBb}]}{K_{\text{mC3bBb}}^{\text{C5}}\left(1+\frac{[\text{C3}]}{K_{\text{mC3bBb}}^{\text{C3}}}+\frac{[\text{C5}]}{K_{\text{mC3bBb}}^{\text{C5}}}\right)}+\frac{k_{\text{catC3bBb}}^{\text{C5}}[\text{C5}][\text{hC3bBbP}]}{K_{\text{mC3bBb}}^{\text{C5}}\left(1+\frac{[\text{C3}]}{K_{\text{mC3bBb}}^{\text{C3}}}+\frac{[\text{C5}]}{K_{\text{mC3bBb}}^{\text{C5}}}\right)}$$

$$+\frac{k_{\text{catC3bC3bBb}}^{\text{C5}}[\text{C5}][\text{hC3bC3bBb}]}{K_{\text{mC3bC3bBb}}^{\text{C5}}\left(1+\frac{[\text{C3}]}{K_{\text{mC3bBb}}^{\text{C3}}}+\frac{[\text{C5}]}{K_{\text{mC3bC3bBb}}^{\text{C5}}}\right)}+\frac{k_{\text{catC3bC3bBb}}^{\text{C5}}[\text{C5}][\text{hC3bC3bBbP}]}{K_{\text{mC3bC3bBb}}^{\text{C5}}\left(1+\frac{[\text{C3}]}{K_{\text{mC3bBb}}^{\text{C3}}}+\frac{[\text{C5}]}{K_{\text{mC3bC3bBb}}^{\text{C5}}}\right)}$$

$$+\frac{k_{\text{catC3bC4bBb}}^{\text{C5}}[\text{C5}][\text{hC3bC4bBb}]}{K_{\text{mC3bC4bBb}}^{\text{C5}}\left(1+\frac{[\text{C3}]}{K_{\text{mC3bBb}}^{\text{C3}}}+\frac{[\text{C5}]}{K_{\text{mC3bC4bBb}}^{\text{C5}}}\right)}+\frac{k_{\text{catC3bC4bBb}}^{\text{C5}}[\text{C5}][\text{hC3bC4bBbP}]}{K_{\text{mC3bC4bBb}}^{\text{C5}}\left(1+\frac{[\text{C3}]}{K_{\text{mC3bBb}}^{\text{C3}}}+\frac{[\text{C5}]}{K_{\text{mC3bC4bBb}}^{\text{C5}}}\right)}-\frac{k_{\text{catCPN}}^{\text{C5a}}[\text{C5a}][\text{CPN}]}{K_{\text{mCPN}}^{\text{C5a}}\left(1+\frac{[\text{C3a}]}{K_{\text{mCPN}}^{\text{C3a}}}+\frac{[\text{C5a}]}{K_{\text{mCPN}}^{\text{C5a}}}\right)}$$

S264

$$\frac{d[\text{C5adesArg}]}{dt}=\frac{k_{\text{catCPN}}^{\text{C5a}}[\text{C5a}][\text{CPN}]}{K_{\text{mCPN}}^{\text{C5a}}\left(1+\frac{[\text{C3a}]}{K_{\text{mCPN}}^{\text{C3a}}}+\frac{[\text{C5a}]}{K_{\text{mCPN}}^{\text{C5a}}}\right)}$$

S265

$$\frac{d[\text{B}]}{dt}=-k_{\text{C3(H}_2\text{O)B}}^{+}[\text{C3(H}_2\text{O)}][\text{B}]+k_{\text{C3(H}_2\text{O)B}}^{-}[\text{C3(H}_2\text{O)B}]-k_{\text{C3bB}}^{+}[\text{fC3b}][\text{B}]+k_{\text{C3bB}}^{-}[\text{fC3bB}]-k_{\text{C3bB}}^{+}[\text{fC3bC3b}][\text{B}]+k_{\text{C3bB}}^{-}[\text{fC3bC3bB}]$$
$$-k_{\text{C3bB}}^{+}[\text{fC3bC3bP}][\text{B}]+k_{\text{C3bB}}^{-}[\text{fC3bC3bBP}]-k_{\text{C3bB}}^{+}[\text{fC3bC4b}][\text{B}]+k_{\text{C3bB}}^{-}[\text{fC3bC4bB}]-k_{\text{C3bB}}^{+}[\text{fC3bC4bP}][\text{B}]+k_{\text{C3bB}}^{-}[\text{fC3bC4bPB}]$$
$$-k_{\text{C3bB}}^{+}[\text{IgGC3bC3b}][\text{B}]+k_{\text{C3bB}}^{-}[\text{IgGC3bC3bB}]-k_{\text{C3bB}}^{+}[\text{IgGC3bC3bP}][\text{B}]+k_{\text{C3bB}}^{-}[\text{IgGC3bC3bBP}]-k_{\text{C3bB}}^{+}[\text{hC3b}][\text{B}]+k_{\text{C3bB}}^{-}[\text{hC3bB}]$$
$$-k_{\text{C3bB}}^{+}[\text{hC3bC3b}][\text{B}]+k_{\text{C3bB}}^{-}[\text{hC3bC3bB}]-k_{\text{C3bB}}^{+}[\text{hC3bC3bP}][\text{B}]+k_{\text{C3bB}}^{-}[\text{hC3bC3bBP}]-k_{\text{C3bB}}^{+}[\text{hC3bC4b}][\text{B}]+k_{\text{C3bB}}^{-}[\text{hC3bC4bB}]$$
$$-k_{\text{C3bB}}^{+}[\text{hC3bC4bP}][\text{B}]+k_{\text{C3bB}}^{-}[\text{hC3bC4bPB}]$$

S266

$$\frac{d[\mathrm{Ba}]}{dt}=$$

$$+\left(\frac{k^{\mathrm{D}}_{\mathrm{catC3(H_2O)B}}[\mathrm{D}][\mathrm{C3(H_2O)B}]}{K^{\mathrm{D}}_{\mathrm{mC3(H_2O)B}}+[\mathrm{C3(H_2O)B}]+[\mathrm{fC3bB}]+[\mathrm{fC3bC3bB}]+[\mathrm{fC3bC3bBP}]+[\mathrm{IgGC3bC3bB}]+[\mathrm{IgGC3bC3bBP}]+[\mathrm{fC3bC4bB}]+[\mathrm{fC3bC4bBP}]}\cdots \frac{1}{+[\mathrm{hC3bB}]+[\mathrm{hC3bC3bB}]+[\mathrm{hC3bC3bBP}]+[\mathrm{hC3bC4bB}]+[\mathrm{hC3bC4bBP}]}\right)$$

$$+\left(\frac{k^{\mathrm{D}}_{\mathrm{catC3(H_2O)B}}[\mathrm{D}][\mathrm{fC3bB}]}{K^{\mathrm{D}}_{\mathrm{mC3(H_2O)B}}+[\mathrm{C3(H_2O)B}]+[\mathrm{fC3bB}]+[\mathrm{fC3bC3bB}]+[\mathrm{fC3bC3bBP}]+[\mathrm{IgGC3bC3bB}]+[\mathrm{IgGC3bC3bBP}]+[\mathrm{fC3bC4bB}]+[\mathrm{fC3bC4bBP}]}\cdots \frac{1}{+[\mathrm{hC3bB}]+[\mathrm{hC3bC3bB}]+[\mathrm{hC3bC3bBP}]+[\mathrm{hC3bC4bB}]+[\mathrm{hC3bC4bBP}]}\right)$$

$$+\left(\frac{k^{\mathrm{D}}_{\mathrm{catC3(H_2O)B}}[\mathrm{D}][\mathrm{fC3bC3bB}]}{K^{\mathrm{D}}_{\mathrm{mC3(H_2O)B}}+[\mathrm{C3(H_2O)B}]+[\mathrm{fC3bB}]+[\mathrm{fC3bC3bB}]+[\mathrm{fC3bC3bBP}]+[\mathrm{IgGC3bC3bB}]+[\mathrm{IgGC3bC3bBP}]+[\mathrm{fC3bC4bB}]+[\mathrm{fC3bC4bBP}]}\cdots \frac{1}{+[\mathrm{hC3bB}]+[\mathrm{hC3bC3bB}]+[\mathrm{hC3bC3bBP}]+[\mathrm{hC3bC4bB}]+[\mathrm{hC3bC4bBP}]}\right)$$

$$+\left(\frac{k^{\mathrm{D}}_{\mathrm{catC3(H_2O)B}}[\mathrm{D}][\mathrm{fC3bC3bBP}]}{K^{\mathrm{D}}_{\mathrm{mC3(H_2O)B}}+[\mathrm{C3(H_2O)B}]+[\mathrm{fC3bB}]+[\mathrm{fC3bC3bB}]+[\mathrm{fC3bC3bBP}]+[\mathrm{IgGC3bC3bB}]+[\mathrm{IgGC3bC3bBP}]+[\mathrm{fC3bC4bB}]+[\mathrm{fC3bC4bBP}]}\cdots \frac{1}{+[\mathrm{hC3bB}]+[\mathrm{hC3bC3bB}]+[\mathrm{hC3bC3bBP}]+[\mathrm{hC3bC4bB}]+[\mathrm{hC3bC4bBP}]}\right)$$

$$+\left(\frac{k^{\mathrm{D}}_{\mathrm{catC3(H_2O)B}}[\mathrm{D}][\mathrm{IgGC3bC3bB}]}{K^{\mathrm{D}}_{\mathrm{mC3(H_2O)B}}+[\mathrm{C3(H_2O)B}]+[\mathrm{fC3bB}]+[\mathrm{fC3bC3bB}]+[\mathrm{fC3bC3bBP}]+[\mathrm{IgGC3bC3bB}]+[\mathrm{IgGC3bC3bBP}]+[\mathrm{fC3bC4bB}]+[\mathrm{fC3bC4bBP}]}\cdots \frac{1}{+[\mathrm{hC3bB}]+[\mathrm{hC3bC3bB}]+[\mathrm{hC3bC3bBP}]+[\mathrm{hC3bC4bB}]+[\mathrm{hC3bC4bBP}]}\right)$$

$$+\left(\frac{k^{\mathrm{D}}_{\mathrm{catC3(H_2O)B}}[\mathrm{D}][\mathrm{IgGC3bC3bBP}]}{K^{\mathrm{D}}_{\mathrm{mC3(H_2O)B}}+[\mathrm{C3(H_2O)B}]+[\mathrm{fC3bB}]+[\mathrm{fC3bC3bB}]+[\mathrm{fC3bC3bBP}]+[\mathrm{IgGC3bC3bB}]+[\mathrm{IgGC3bC3bBP}]+[\mathrm{fC3bC4bB}]+[\mathrm{fC3bC4bBP}]}\cdots \frac{1}{+[\mathrm{hC3bB}]+[\mathrm{hC3bC3bB}]+[\mathrm{hC3bC3bBP}]+[\mathrm{hC3bC4bB}]+[\mathrm{hC3bC4bBP}]}\right)$$

$$+\left(\frac{k^{\mathrm{D}}_{\mathrm{catC3(H_2O)B}}[\mathrm{D}][\mathrm{fC3bC4bB}]}{K^{\mathrm{D}}_{\mathrm{mC3(H_2O)B}}+[\mathrm{C3(H_2O)B}]+[\mathrm{fC3bB}]+[\mathrm{fC3bC3bB}]+[\mathrm{fC3bC3bBP}]+[\mathrm{IgGC3bC3bB}]+[\mathrm{IgGC3bC3bBP}]+[\mathrm{fC3bC4bB}]+[\mathrm{fC3bC4bBP}]}\cdots \frac{1}{+[\mathrm{hC3bB}]+[\mathrm{hC3bC3bB}]+[\mathrm{hC3bC3bBP}]+[\mathrm{hC3bC4bB}]+[\mathrm{hC3bC4bBP}]}\right)$$

$$+\left(\begin{array}{l}\dfrac{k^{\mathrm{D}}_{\mathrm{catC3(H_2O)B}}[\mathrm{D}][\mathrm{fC3bC4bBP}]}{K^{\mathrm{D}}_{\mathrm{mC3(H_2O)B}}+[\mathrm{C3(H_2O)B}]+[\mathrm{fC3bB}]+[\mathrm{fC3bC3bB}]+[\mathrm{fC3bC3bBP}]+[\mathrm{IgGC3bC3bB}]+[\mathrm{IgGC3bC3bBP}]+[\mathrm{fC3bC4bB}]+[\mathrm{fC3bC4bBP}]}\cdots\\ \dfrac{1}{+[\mathrm{hC3bB}]+[\mathrm{hC3bC3bB}]+[\mathrm{hC3bC3bBP}]+[\mathrm{hC3bC4bB}]+[\mathrm{hC3bC4bBP}]}\end{array}\right)$$

$$+\left(\begin{array}{l}\dfrac{k^{\mathrm{D}}_{\mathrm{catC3(H_2O)B}}[\mathrm{D}][\mathrm{hC3b}]}{K^{\mathrm{D}}_{\mathrm{mC3(H_2O)B}}+[\mathrm{C3(H_2O)B}]+[\mathrm{fC3bB}]+[\mathrm{fC3bC3bB}]+[\mathrm{fC3bC3bBP}]+[\mathrm{IgGC3bC3bB}]+[\mathrm{IgGC3bC3bBP}]+[\mathrm{fC3bC4bB}]+[\mathrm{fC3bC4bBP}]}\cdots\\ \dfrac{1}{+[\mathrm{hC3bB}]+[\mathrm{hC3bC3bB}]+[\mathrm{hC3bC3bBP}]+[\mathrm{hC3bC4bB}]+[\mathrm{hC3bC4bBP}]}\end{array}\right)$$

$$+\left(\begin{array}{l}\dfrac{k^{\mathrm{D}}_{\mathrm{catC3(H_2O)B}}[\mathrm{D}][\mathrm{hC3bC3bB}]}{K^{\mathrm{D}}_{\mathrm{mC3(H_2O)B}}+[\mathrm{C3(H_2O)B}]+[\mathrm{fC3bB}]+[\mathrm{fC3bC3bB}]+[\mathrm{fC3bC3bBP}]+[\mathrm{IgGC3bC3bB}]+[\mathrm{IgGC3bC3bBP}]+[\mathrm{fC3bC4bB}]+[\mathrm{fC3bC4bBP}]}\cdots\\ \dfrac{1}{+[\mathrm{hC3bB}]+[\mathrm{hC3bC3bB}]+[\mathrm{hC3bC3bBP}]+[\mathrm{hC3bC4bB}]+[\mathrm{hC3bC4bBP}]}\end{array}\right)$$

$$+\left(\begin{array}{l}\dfrac{k^{\mathrm{D}}_{\mathrm{catC3(H_2O)B}}[\mathrm{D}][\mathrm{hC3bC3bBP}]}{K^{\mathrm{D}}_{\mathrm{mC3(H_2O)B}}+[\mathrm{C3(H_2O)B}]+[\mathrm{fC3bB}]+[\mathrm{fC3bC3bB}]+[\mathrm{fC3bC3bBP}]+[\mathrm{IgGC3bC3bB}]+[\mathrm{IgGC3bC3bBP}]+[\mathrm{fC3bC4bB}]+[\mathrm{fC3bC4bBP}]}\cdots\\ \dfrac{1}{+[\mathrm{hC3bB}]+[\mathrm{hC3bC3bB}]+[\mathrm{hC3bC3bBP}]+[\mathrm{hC3bC4bB}]+[\mathrm{hC3bC4bBP}]}\end{array}\right)$$

$$+\left(\begin{array}{l}\dfrac{k^{\mathrm{D}}_{\mathrm{catC3(H_2O)B}}[\mathrm{D}][\mathrm{hC3bC4bB}]}{K^{\mathrm{D}}_{\mathrm{mC3(H_2O)B}}+[\mathrm{C3(H_2O)B}]+[\mathrm{fC3bB}]+[\mathrm{fC3bC3bB}]+[\mathrm{fC3bC3bBP}]+[\mathrm{IgGC3bC3bB}]+[\mathrm{IgGC3bC3bBP}]+[\mathrm{fC3bC4bB}]+[\mathrm{fC3bC4bBP}]}\cdots\\ \dfrac{1}{+[\mathrm{hC3bB}]+[\mathrm{hC3bC3bB}]+[\mathrm{hC3bC3bBP}]+[\mathrm{hC3bC4bB}]+[\mathrm{hC3bC4bBP}]}\end{array}\right)$$

$$+\left(\begin{array}{l}\dfrac{k^{\mathrm{D}}_{\mathrm{catC3(H_2O)B}}[\mathrm{D}][\mathrm{hC3bC4bBP}]}{K^{\mathrm{D}}_{\mathrm{mC3(H_2O)B}}+[\mathrm{C3(H_2O)B}]+[\mathrm{fC3bB}]+[\mathrm{fC3bC3bB}]+[\mathrm{fC3bC3bBP}]+[\mathrm{IgGC3bC3bB}]+[\mathrm{IgGC3bC3bBP}]+[\mathrm{fC3bC4bB}]+[\mathrm{fC3bC4bBP}]}\cdots\\ \dfrac{1}{+[\mathrm{hC3bB}]+[\mathrm{hC3bC3bB}]+[\mathrm{hC3bC3bBP}]+[\mathrm{hC3bC4bB}]+[\mathrm{hC3bC4bBP}]}\end{array}\right)$$

S267

$$\frac{d[\mathrm{Bb}]}{dt} = k^{-}_{\mathrm{C3(H_2O)Bb}}[\mathrm{C3(H_2O)Bb}] + k^{-}_{\mathrm{C3bBb}}[\mathrm{fC3bBb}] + k^{-}_{\mathrm{C3bC3bBb}}[\mathrm{fC3bC3bBb}] + k^{-}_{\mathrm{C3bC3bBbP}}[\mathrm{fC3bC3bBbP}] + k^{-}_{\mathrm{C3bC3bBb}}[\mathrm{IgGC3bC3bBb}]$$
$$+ k^{-}_{\mathrm{C3bC3bBbP}}[\mathrm{IgGC3bC3bBbP}] + k^{-}_{\mathrm{C3bC4bBb}}[\mathrm{fC3bC4bBb}] + k^{-}_{\mathrm{C3bC4bBbP}}[\mathrm{fC3bC4bBbP}] + k^{-}_{\mathrm{C3bBbH}_{\mathrm{decay}}}[\mathrm{C3(H_2O)BbH}] + k^{-}_{\mathrm{C3bBbHL}_{\mathrm{decay}}}[\mathrm{C3(H_2O)BbHL}]$$
$$+ k^{-}_{\mathrm{C3bBbH}_{\mathrm{decay}}}[\mathrm{fC3bBbH}] + k^{-}_{\mathrm{C3bBbHL}_{\mathrm{decay}}}[\mathrm{fC3bBbHL}] + k^{-}_{\mathrm{C3bBbCR1}_{\mathrm{decay}}}[\mathrm{fC3bBbCR1}] + k^{-}_{\mathrm{C3bBbH}_{\mathrm{decay}}}[\mathrm{fC3bC4bBbH}] + k^{-}_{\mathrm{C3bBbHL}_{\mathrm{decay}}}[\mathrm{fC3bC4bBbHL}]$$
$$+ k^{-}_{\mathrm{C3bBbCR1}_{\mathrm{decay}}}[\mathrm{fC3bC4bBbCR1}] + k^{-}_{\mathrm{C3bBbH}_{\mathrm{decay}}}[\mathrm{fC3bC3bBbH}] + k^{-}_{\mathrm{C3bBbHL}_{\mathrm{decay}}}[\mathrm{fC3bC3bBbHL}] + k^{-}_{\mathrm{C3bBbCR1}_{\mathrm{decay}}}[\mathrm{fC3bC3bBbCR1}]$$
$$+ k^{-}_{\mathrm{C3bBbH}_{\mathrm{decay}}}[\mathrm{IgGC3bC3bBbH}] + k^{-}_{\mathrm{C3bBbHL}_{\mathrm{decay}}}[\mathrm{IgGC3bC3bBbHL}] + k^{-}_{\mathrm{C3bBbCR1}_{\mathrm{decay}}}[\mathrm{IgGC3bC3bBbCR1}] + k^{-}_{\mathrm{C3bBb}}[\mathrm{hC3bBb}] + k^{-}_{\mathrm{C3bBbP}}[\mathrm{hC3bBbP}]$$
$$+ k^{-}_{\mathrm{C3bC4bBb}}[\mathrm{hC3bC4bBb}] + k^{-}_{\mathrm{C3bC4bBbP}}[\mathrm{hC3bC4bBbP}] + k^{-}_{\mathrm{C3bC3bBb}}[\mathrm{hC3bC3bBb}] + k^{-}_{\mathrm{C3bC3bBbP}}[\mathrm{hC3bC3bBbP}] + k^{-}_{\mathrm{C3bBbH}_{\mathrm{decay}}}[\mathrm{hC3bBbH}]$$
$$+ k^{-}_{\mathrm{C3bBbHL}_{\mathrm{decay}}}[\mathrm{hC3bBbHL}] + k^{-}_{\mathrm{C3bBbCR1}_{\mathrm{decay}}}[\mathrm{hC3bBbCR1}] + k^{-}_{\mathrm{C3bBbDAF}_{\mathrm{decay}}}[\mathrm{hC3bBbDAF}] + k^{-}_{\mathrm{C3bBbH}_{\mathrm{decay}}}[\mathrm{hC3bC4bBbH}]$$
$$+ k^{-}_{\mathrm{C3bBbHL}_{\mathrm{decay}}}[\mathrm{hC3bC4bBbHL}] + k^{-}_{\mathrm{C3bBbCR1}_{\mathrm{decay}}}[\mathrm{hC3bC4bBbCR1}] + k^{-}_{\mathrm{C3bBbDAF}_{\mathrm{decay}}}[\mathrm{hC3bC4bBbDAF}] + k^{-}_{\mathrm{C3bBbH}_{\mathrm{decay}}}[\mathrm{hC3bC3bBbH}]$$
$$+ k^{-}_{\mathrm{C3bBbHL}_{\mathrm{decay}}}[\mathrm{hC3bC3bBbHL}] + k^{-}_{\mathrm{C3bBbCR1}_{\mathrm{decay}}}[\mathrm{hC3bC3bBbCR1}] + k^{-}_{\mathrm{C3bBbDAF}_{\mathrm{decay}}}[\mathrm{hC3bC3bBbDAF}]$$

S268

$$\frac{d[\mathrm{P}]}{dt} = -k^{+}_{\mathrm{C3bP}}[\mathrm{fC3bC3b}][\mathrm{P}] + k^{-}_{\mathrm{C3bP}}[\mathrm{fC3bC3bP}] - k^{+}_{\mathrm{C3bP}}[\mathrm{fC3bC3bBb}][\mathrm{P}] + k^{-}_{\mathrm{C3bP}}[\mathrm{fC3bC3bBbP}] + k^{-}_{\mathrm{C3bC3bBbP}}[\mathrm{fC3bC3bBbP}]$$
$$- k^{+}_{\mathrm{C3bP}}[\mathrm{fC3bC4b}][\mathrm{P}] + k^{-}_{\mathrm{C3bP}}[\mathrm{fC3bC4bP}] - k^{+}_{\mathrm{C3bP}}[\mathrm{fC3bC4bBb}][\mathrm{P}] + k^{-}_{\mathrm{C3bP}}[\mathrm{fC3bC4bBbP}] + k^{-}_{\mathrm{C3bC4bBbP}}[\mathrm{fC3bC4bBbP}]$$
$$- k^{+}_{\mathrm{C3bP}}[\mathrm{IgGC3bC3b}][\mathrm{P}] + k^{-}_{\mathrm{C3bP}}[\mathrm{IgGC3bC3bP}] - k^{+}_{\mathrm{C3bP}}[\mathrm{IgGC3bC3bBb}][\mathrm{P}] + k^{-}_{\mathrm{C3bP}}[\mathrm{IgGC3bC3bBbP}] + k^{-}_{\mathrm{C3bC3bBbP}}[\mathrm{IgGC3bC3bBbP}]$$
$$- k^{+}_{\mathrm{C3bP}}[\mathrm{hC3bBb}][\mathrm{P}] + k^{-}_{\mathrm{C3bP}}[\mathrm{hC3bBbP}] + k^{-}_{\mathrm{C3bBbP}}[\mathrm{hC3bBbP}] - k^{+}_{\mathrm{C3bP}}[\mathrm{hC3bC3b}][\mathrm{P}] + k^{-}_{\mathrm{C3bP}}[\mathrm{hC3bC3bP}] - k^{+}_{\mathrm{C3bP}}[\mathrm{hC3bC3bBb}][\mathrm{P}]$$
$$+ k^{-}_{\mathrm{C3bP}}[\mathrm{hC3bC3bBbP}] + k^{-}_{\mathrm{C3bC3bBbP}}[\mathrm{hC3bC3bBbP}] - k^{+}_{\mathrm{C3bP}}[\mathrm{hC3bC4b}][\mathrm{P}] + k^{-}_{\mathrm{C3bP}}[\mathrm{hC3bC4bP}] - k^{+}_{\mathrm{C3bP}}[\mathrm{hC3bC4bBb}][\mathrm{P}]$$
$$+ k^{-}_{\mathrm{C3bP}}[\mathrm{hC3bC4bBbP}] + k^{-}_{\mathrm{C3bC4bBbP}}[\mathrm{hC3bC4bBbP}]$$

S269

$$\frac{d[\mathrm{C2}]}{dt} = -k^{+}_{\mathrm{C4bC2}}[\mathrm{fC4b}][\mathrm{C2}] + k^{-}_{\mathrm{C4bC2}}[\mathrm{fC4bC2}] - k^{+}_{\mathrm{C4bC2}}[\mathrm{fC4bC4b}][\mathrm{C2}] + k^{-}_{\mathrm{C4bC2}}[\mathrm{fC4bC4bC2}] - k^{+}_{\mathrm{C4bC2}}[\mathrm{fC3bC4b}][\mathrm{C2}] + k^{-}_{\mathrm{C4bC2}}[\mathrm{fC3bC4bC2}]$$
$$- k^{+}_{\mathrm{C4bC2}}[\mathrm{hC4b}][\mathrm{C2}] + k^{-}_{\mathrm{C4bC2}}[\mathrm{hC4bC2}] - k^{+}_{\mathrm{C4bC2}}[\mathrm{hC4bC4b}][\mathrm{C2}] + k^{-}_{\mathrm{C4bC2}}[\mathrm{hC4bC4bC2}] - k^{+}_{\mathrm{C4bC2}}[\mathrm{hC3bC4b}][\mathrm{C2}] + k^{-}_{\mathrm{C4bC2}}[\mathrm{hC3bC4bC2}]$$

S270

$$\frac{d[\mathrm{C2b}]}{dt} = \frac{k^{\mathrm{C2}}_{\mathrm{catC1*}}[\mathrm{fC4bC2}][\mathrm{C1*}]}{K^{\mathrm{C2}}_{\mathrm{mC1*}}\left(1+\frac{[\mathrm{fC4bC2}]}{K^{\mathrm{C2}}_{\mathrm{mC1*}}}+\frac{[\mathrm{fC4bC4bC2}]}{K^{\mathrm{C2}}_{\mathrm{mC1*}}}+\frac{[\mathrm{fC3bC4bC2}]}{K^{\mathrm{C2}}_{\mathrm{mC1*}}}+\frac{[\mathrm{C4}]}{K^{\mathrm{C4}}_{\mathrm{mC1*}}}\right)} + \frac{k^{\mathrm{C2}}_{\mathrm{catC1*}}[\mathrm{fC4bC4bC2}][\mathrm{C1*}]}{K^{\mathrm{C2}}_{\mathrm{mC1*}}\left(1+\frac{[\mathrm{fC4bC2}]}{K^{\mathrm{C2}}_{\mathrm{mC1*}}}+\frac{[\mathrm{fC4bC4bC2}]}{K^{\mathrm{C2}}_{\mathrm{mC1*}}}+\frac{[\mathrm{fC3bC4bC2}]}{K^{\mathrm{C2}}_{\mathrm{mC1*}}}+\frac{[\mathrm{C4}]}{K^{\mathrm{C4}}_{\mathrm{mC1*}}}\right)}$$
$$+ \frac{k^{\mathrm{C2}}_{\mathrm{catC1*}}[\mathrm{fC3bC4bC2}][\mathrm{C1*}]}{K^{\mathrm{C2}}_{\mathrm{mC1*}}\left(1+\frac{[\mathrm{fC4bC2}]}{K^{\mathrm{C2}}_{\mathrm{mC1*}}}+\frac{[\mathrm{fC4bC4bC2}]}{K^{\mathrm{C2}}_{\mathrm{mC1*}}}+\frac{[\mathrm{fC3bC4bC2}]}{K^{\mathrm{C2}}_{\mathrm{mC1*}}}+\frac{[\mathrm{C4}]}{K^{\mathrm{C4}}_{\mathrm{mC1*}}}\right)}$$

S271

$$\frac{d[\text{C2a}]}{dt} = k^{-}_{\text{C4bC2a}}[\text{fC4bC2a}] + k^{-}_{\text{C4bC2aC4BP}_{\text{decay}}}[\text{fC4bC2aC4BP}] + k^{-}_{\text{C4bC2aCR1}_{\text{decay}}}[\text{fC4bC2aCR1}] + k^{-}_{\text{C4bC4bC2a}}[\text{fC4bC4bC2a}]$$
$$+ k^{-}_{\text{C4bC2aC4BP}_{\text{decay}}}[\text{fC4bC4bC2aC4BP}] + k^{-}_{\text{C4bC2aCR1}_{\text{decay}}}[\text{fC4bC4bC2aCR1}] + k^{-}_{\text{C3bC4bC2a}}[\text{fC3bC4bC2a}]$$
$$+ k^{-}_{\text{C4bC2aC4BP}_{\text{decay}}}[\text{fC3bC4bC2aC4BP}] + k^{-}_{\text{C3bBbCR1}_{\text{decay}}}[\text{fC3bC4bC2aCR1}]$$

S272

$$\frac{d[\text{C1q}]}{dt} = -k^{+}_{\text{C1}}[\text{C1q}][(\text{C1rC1s})_2] + k^{-}_{\text{C1}}[\text{C1}] - k^{+}_{\text{C1}}[\text{C1q}][(\text{C1rC1s})^*_2] + k^{-}_{\text{C1}}[\text{C1*}] + k^{+}_{\text{C1*C1-INH}}[\text{C1*}][\text{C1-INH}]$$

S273

$$\frac{d[(\text{C1rC1s})_2]}{dt} = -k^{+}_{\text{C1}}[\text{C1q}][(\text{C1rC1s})_2] + k^{-}_{\text{C1}}[\text{C1}]$$

S274

$$\frac{d[(\text{C1rC1s})^*_2]}{dt} = -k^{+}_{\text{C1}}[\text{C1q}][(\text{C1rC1s})^*_2] + k^{-}_{\text{C1}}[\text{C1*}]$$

S275

$$\frac{d[\text{C1}]}{dt} = k^{+}_{\text{C1}}[\text{C1q}][(\text{C1rC1s})_2] - k^{-}_{\text{C1}}[\text{C1}] - k^{+}_{\text{activation}}[\text{C1}]$$

S276

$$\frac{d[\text{C1*}]}{dt} = k^{+}_{\text{activation}}[\text{C1}] + k^{+}_{\text{C1}}[\text{C1q}][(\text{C1rC1s})^*_2] - k^{-}_{\text{C1}}[\text{C1*}] - k^{+}_{\text{C1*C1-INH}}[\text{C1*}][\text{C1-INH}]$$

S277

$$\frac{d[\text{C1-INH}]}{dt} = -k^{+}_{\text{C1*C1-INH}}[\text{C1*}][\text{C1-INH}]$$

S278

$$\frac{d[\text{IgG}]}{dt} = -k^{+}_{\text{IgGC3b}}[\text{IgG}][\text{nfC3b}]$$

S279

$$\frac{d[\text{C6}]}{dt} = -k^{+}_{\text{C5b6}}[\text{fC5b}][\text{C6}] + k^{-}_{\text{C5b6}}[\text{fC5b6}] - k^{+}_{\text{C5b6}}[\text{fC3bC3bBbC5b}][\text{C6}] + k^{-}_{\text{C5b6}}[\text{fC3bC3bBbC5b6}] - k^{+}_{\text{C5b6}}[\text{fC3bC3bBbPC5b}][\text{C6}]$$
$$+ k^{-}_{\text{C5b6}}[\text{fC3bC3bBbPC5b6}] - k^{+}_{\text{C5b6}}[\text{fC3bC4bBbC5b}][\text{C6}] + k^{-}_{\text{C5b6}}[\text{fC3bC4bBbC5b6}] - k^{+}_{\text{C5b6}}[\text{fC3bC4bBbPC5b}][\text{C6}] + k^{-}_{\text{C5b6}}[\text{fC3bC4bBbPC5b6}]$$
$$- k^{+}_{\text{C5b6}}[\text{fC4bC4bC2aC5b}][\text{C6}] + k^{-}_{\text{C5b6}}[\text{fC4bC4bC2aC5b6}] - k^{+}_{\text{C5b6}}[\text{fC3bC4bC2aC5b}][\text{C6}] + k^{-}_{\text{C5b6}}[\text{fC3bC4bC2aC5b6}]$$
$$- k^{+}_{\text{C5b6}}[\text{hC3bC3bBbC5b}][\text{C6}] + k^{-}_{\text{C5b6}}[\text{hC3bC3bBbC5b6}] - k^{+}_{\text{C5b6}}[\text{hC3bC3bBbPC5b}][\text{C6}] + k^{-}_{\text{C5b6}}[\text{hC3bC3bBbPC5b6}] - k^{+}_{\text{C5b6}}[\text{hC3bC4bBbC5b}][\text{C6}]$$
$$+ k^{-}_{\text{C5b6}}[\text{hC3bC4bBbC5b6}] - k^{+}_{\text{C5b6}}[\text{hC3bC4bBbPC5b}][\text{C6}] + k^{-}_{\text{C5b6}}[\text{hC3bC4bBbPC5b6}]$$

S280

$$\frac{d[\mathrm{C7}]}{dt} = -k^{+}_{\mathrm{C5b7}}[\mathrm{fC5b6}][\mathrm{C7}] + k^{-}_{\mathrm{C5b7}}[\mathrm{fC5b7}] - k^{+}_{\mathrm{C5b7}}[\mathrm{fC3bC3bBbC5b6}][\mathrm{C7}] - k^{+}_{\mathrm{C5b7}}[\mathrm{fC3bC3bBbPC5b6}][\mathrm{C7}] - k^{+}_{\mathrm{C5b7}}[\mathrm{fC3bC4bBbC5b6}][\mathrm{C7}] - k^{+}_{\mathrm{C5b7}}[\mathrm{fC3bC4bBbPC5b6}][\mathrm{C7}] - k^{+}_{\mathrm{C5b7}}[\mathrm{fC3bC4bC2aC5b6}][\mathrm{C7}] - k^{+}_{\mathrm{C5b7}}[\mathrm{fC4bC4bC2aC5b6}][\mathrm{C7}] - k^{+}_{\mathrm{C5b7}}[\mathrm{hC3bC3bBbC5b6}][\mathrm{C7}] - k^{+}_{\mathrm{C5b7}}[\mathrm{hC3bC3bBbPC5b6}][\mathrm{C7}] - k^{+}_{\mathrm{C5b7}}[\mathrm{hC3bC4bBbC5b6}][\mathrm{C7}] - k^{+}_{\mathrm{C5b7}}[\mathrm{hC3bC4bBbPC5b6}][\mathrm{C7}]$$

S281

$$\frac{d[\mathrm{C8}]}{dt} = -k^{+}_{\mathrm{C5b8}}[\mathrm{fC5b7}][\mathrm{C8}] + k^{-}_{\mathrm{C5b8}}[\mathrm{fC5b8}] - k^{+}_{\mathrm{C5b8}}[\mathrm{VnC5b7}][\mathrm{C8}] + k^{-}_{\mathrm{C5b8}}[\mathrm{VnC5b8}] - k^{+}_{\mathrm{C5b8}}[\mathrm{CnC5b7}][\mathrm{C8}] + k^{-}_{\mathrm{C5b8}}[\mathrm{CnC5b8}] - k^{+}_{\mathrm{C5b8}}[\mathrm{hC5b7}][\mathrm{C8}]$$

S282

$$\frac{d[\mathrm{C9}]}{dt} = -k^{+}_{\mathrm{C5b9}}[\mathrm{fC5b8}][\mathrm{C9}_{1}] + k^{-}_{\mathrm{C5b9}}[\mathrm{fC5bC9}_{1}] - k^{+}_{\mathrm{C5b9}}[\mathrm{VnC5b8}][\mathrm{C9}_{1}] + k^{-}_{\mathrm{C5b9}}[\mathrm{VnC5bC9}_{1}] - k^{+}_{\mathrm{C5b9}}[\mathrm{CnC5b8}][\mathrm{C9}_{1}] + k^{-}_{\mathrm{C5b9}}[\mathrm{CnC5bC9}_{1}] - k^{+}_{\mathrm{C5b9}}[\mathrm{hC5b8}][\mathrm{C9}_{1}] - k^{+}_{\mathrm{C5b9}}[\mathrm{hC5b9}_{1}][\mathrm{C9}_{2}] - k^{+}_{\mathrm{C5b9}}[\mathrm{hC5b9}_{2}][\mathrm{C9}_{3}] - k^{+}_{\mathrm{C5b9}}[\mathrm{hC5b9}_{3}][\mathrm{C9}_{4}] - k^{+}_{\mathrm{C5b9}}[\mathrm{hC5b9}_{4}][\mathrm{C9}_{5}] - k^{+}_{\mathrm{C5b9}}[\mathrm{hC5b9}_{5}][\mathrm{C9}_{6}] - k^{+}_{\mathrm{C5b9}}[\mathrm{hC5b9}_{6}][\mathrm{C9}_{7}] - k^{+}_{\mathrm{C5b9}}[\mathrm{hC5b9}_{7}][\mathrm{C9}_{8}] - k^{+}_{\mathrm{C5b9}}[\mathrm{hC5b9}_{8}][\mathrm{C9}_{9}] - k^{+}_{\mathrm{C5b9}}[\mathrm{hC5b9}_{9}][\mathrm{C9}_{10}] - k^{+}_{\mathrm{C5b9}}[\mathrm{hC5b9}_{10}][\mathrm{C9}_{11}] - k^{+}_{\mathrm{C5b9}}[\mathrm{hC5b9}_{11}][\mathrm{C9}_{12}] - k^{+}_{\mathrm{C5b9}}[\mathrm{hC5b9}_{12}][\mathrm{C9}_{13}] - k^{+}_{\mathrm{C5b9}}[\mathrm{hC5b9}_{13}][\mathrm{C9}_{14}] - k^{+}_{\mathrm{C5b9}}[\mathrm{hC5b9}_{14}][\mathrm{C9}_{15}] - k^{+}_{\mathrm{C5b9}}[\mathrm{hC5b9}_{15}][\mathrm{C9}_{16}] - k^{+}_{\mathrm{C5b9}}[\mathrm{hC5b9}_{16}][\mathrm{C9}_{17}] - k^{+}_{\mathrm{C5b9}}[\mathrm{hC5b9}_{17}][\mathrm{C9}_{18}]$$

S283

$$\frac{d[\mathrm{Vn}]}{dt} = -k^{+}_{\mathrm{VnC5b7}}[\mathrm{fC5b7}][\mathrm{Vn}] + k^{-}_{\mathrm{VnC5b7}}[\mathrm{VnfC5b7}] - k^{+}_{\mathrm{VnC5b8}}[\mathrm{fC5b8}][\mathrm{Vn}] + k^{-}_{\mathrm{VnC5b8}}[\mathrm{VnfC5b8}] - k^{+}_{\mathrm{VnC5b9}}[\mathrm{fC5b9}_{1}][\mathrm{Vn}] + k^{-}_{\mathrm{VnC5b9}}[\mathrm{VnfC5b9}_{1}]$$

S284

$$\frac{d[\mathrm{Cn}]}{dt} = -k^{+}_{\mathrm{CnC5b7}}[\mathrm{fC5b7}][\mathrm{Cn}] + k^{-}_{\mathrm{CnC5b7}}[\mathrm{CnfC5b7}] - k^{+}_{\mathrm{CnC5b8}}[\mathrm{fC5b8}][\mathrm{Cn}] + k^{-}_{\mathrm{CnC5b8}}[\mathrm{CnfC5b8}] - k^{+}_{\mathrm{CnC5b9}}[\mathrm{fC5b9}_{1}][\mathrm{Cn}] + k^{-}_{\mathrm{CnC5b9}}[\mathrm{CnfC5b9}_{1}]$$

S285

$$\frac{d[\mathrm{DAF}]}{dt} = -k^{+}_{\mathrm{C3bBbDAF}}[\mathrm{hC3bBb}][\mathrm{DAF}] + k^{-}_{\mathrm{C3bBbDAF}}[\mathrm{hC3bBbDAF}] + k^{-}_{\mathrm{C3bBbDAF}_{\mathrm{decay}}}[\mathrm{hC3bBbDAF}] - k^{+}_{\mathrm{C3bBbDAF}}[\mathrm{hC3bC3bBb}][\mathrm{DAF}] + k^{-}_{\mathrm{C3bBbDAF}}[\mathrm{hC3bC3bBbDAF}] + k^{-}_{\mathrm{C3bBbDAF}_{\mathrm{decay}}}[\mathrm{hC3bC3bBbDAF}] - k^{+}_{\mathrm{C3bBbDAF}}[\mathrm{hC3bC4bBb}][\mathrm{DAF}] + k^{-}_{\mathrm{C3bBbDAF}}[\mathrm{hC3bC4bBbDAF}] + k^{-}_{\mathrm{C3bBbDAF}_{\mathrm{decay}}}[\mathrm{hC3bC4bBbDAF}]$$

S286

$$\frac{d[\mathrm{CD59}]}{dt} = -k^{+}_{\mathrm{C5b8CD59}}[\mathrm{hC5b8}][\mathrm{CD59}] + k^{-}_{\mathrm{C5b8CD59}}[\mathrm{hC5b8CD59}] - k^{+}_{\mathrm{C5b9_{1}CD59}}[\mathrm{hC5b9}_{1}][\mathrm{CD59}] + k^{-}_{\mathrm{C5b9_{1}CD59}}[\mathrm{hC5b9}_{1}\mathrm{CD59}]$$

S287

$$\frac{d[\mathrm{H}]}{dt} = -k^{+}_{\mathrm{C3bH}}[\mathrm{C3(H_2O)}][\mathrm{H}] + k^{-}_{\mathrm{C3bH}}[\mathrm{C3(H_2O)H}]$$

$$+\left(\begin{array}{l}
\dfrac{k^{\mathrm{FI}}_{\mathrm{catC3bH}}[\mathrm{I}][\mathrm{C3(H_2O)H}]}{K^{\mathrm{FI}}_{\mathrm{mC3bH}} + [\mathrm{C3(H_2O)H}] + [\mathrm{C3(H_2O)HL}] + [\mathrm{fC3bH}] + [\mathrm{fC3bHL}] + [\mathrm{fC3bCR1}] + [\mathrm{fiC3bCR1}] + [\mathrm{IgGC3bC3bH}] + [\mathrm{IgGC3biC3bH}] + [\mathrm{IgGC3bC3bHL}]} \cdots \\
\dfrac{1}{+ [\mathrm{IgGC3biC3bHL}] + [\mathrm{IgGC3bC3bCR1}] + [\mathrm{IgGC3biC3bCR1}] + [\mathrm{IgGiC3biC3bCR1}] + [\mathrm{IgGiC3bC3dgCR1}] + [\mathrm{fC3bC4bH}] + [\mathrm{fC3bC4bHL}]} \cdots \\
\dfrac{1}{+ [\mathrm{fC3bC4bCR1}] + [\mathrm{fC3bC4bC4BP}] + [\mathrm{fC3bC4dH}] + [\mathrm{fC3bC4dHL}] + [\mathrm{fC3bC4dCR1}] + [\mathrm{fiC3bC4dCR1}] + [\mathrm{fiC3bC4bCR1}] + [\mathrm{fiC3bC4bC4BP}]} \cdots \\
\dfrac{1}{+ [\mathrm{fC3dgC4bCR1}] + [\mathrm{fC3dgC4bC4BP}] + [\mathrm{fC3bC3bH}] + [\mathrm{fC3bC3bHL}] + [\mathrm{fC3bC3bCR1}] + [\mathrm{fC3biC3bH}] + [\mathrm{fC3biC3bHL}] + [\mathrm{fC3biC3bCR1}]} \cdots \\
\dfrac{1}{+ [\mathrm{fiC3biC3bCR1}] + [\mathrm{fiC3bC3dgCR1}] + [\mathrm{hC3bH}] + [\mathrm{hC3bHL}] + [\mathrm{hC3bCR1}] + [\mathrm{hiC3bCR1}] + [\mathrm{hC3bC4bH}] + [\mathrm{hC3bC4bHL}] + [\mathrm{hC3bC4bCR1}]} \cdots \\
\dfrac{1}{+ [\mathrm{hC3bC4bC4BP}] + [\mathrm{hC3bC4dH}] + [\mathrm{hC3bC4dHL}] + [\mathrm{hC3bC4dCR1}] + [\mathrm{hiC3bC4dCR1}] + [\mathrm{hiC3bC4bCR1}] + [\mathrm{hiC3bC4bC4BP}] + [\mathrm{hC3dgC4bCR1}]} \cdots \\
\dfrac{1}{+ [\mathrm{hC3dgC4bC4BP}] + [\mathrm{hC3bC3bH}] + [\mathrm{hC3bC3bHL}] + [\mathrm{hC3bC3bCR1}] + [\mathrm{hC3biC3bH}] + [\mathrm{hC3biC3bHL}] + [\mathrm{hC3biC3bCR1}] + [\mathrm{hiC3biC3bCR1}]} \cdots \\
\dfrac{1}{+ [\mathrm{hiC3bC3dgCR1}] + [\mathrm{fC4bCR1}] + [\mathrm{nfC4bC4BP}] + [\mathrm{fC4bC4BP}] + [\mathrm{fC4bC4bCR1}] + [\mathrm{fC4bC4bC4BP}] + [\mathrm{fC4bC4dCR1}] + [\mathrm{fC4bC4dC4BP}] + [\mathrm{hC4bCR1}]} \cdots \\
\dfrac{1}{+ [\mathrm{hC4bC4BP}] + [\mathrm{hC4bC4bCR1}] + [\mathrm{hC4bC4bC4BP}] + [\mathrm{hC4bC4dCR1}] + [\mathrm{hC4bC4dC4BP}]} \cdots
\end{array}\right)$$

$$- k^{+}_{\mathrm{C3bH}}[\mathrm{C3(H_2O)Bb}][\mathrm{H}] + k^{-}_{\mathrm{C3bH}}[\mathrm{C3(H_2O)BbH}] + k^{-}_{\mathrm{C3bBbH}_{\mathrm{decay}}}[\mathrm{C3(H_2O)BbH}] - k^{+}_{\mathrm{C3bH}}[\mathrm{fC3b}][\mathrm{H}] + k^{-}_{\mathrm{C3bH}}[\mathrm{fC3bH}]$$

$$+\left(\begin{array}{l}
\dfrac{k^{\mathrm{FI}}_{\mathrm{catC3bH}}[\mathrm{I}][\mathrm{fC3bH}]}{K^{\mathrm{FI}}_{\mathrm{mC3bH}} + [\mathrm{C3(H_2O)H}] + [\mathrm{C3(H_2O)HL}] + [\mathrm{fC3bH}] + [\mathrm{fC3bHL}] + [\mathrm{fC3bCR1}] + [\mathrm{fiC3bCR1}] + [\mathrm{IgGC3bC3bH}] + [\mathrm{IgGC3biC3bH}] + [\mathrm{IgGC3bC3bHL}]} \cdots \\
\dfrac{1}{+ [\mathrm{IgGC3biC3bHL}] + [\mathrm{IgGC3bC3bCR1}] + [\mathrm{IgGC3biC3bCR1}] + [\mathrm{IgGiC3biC3bCR1}] + [\mathrm{IgGiC3bC3dgCR1}] + [\mathrm{fC3bC4bH}] + [\mathrm{fC3bC4bHL}]} \cdots \\
\dfrac{1}{+ [\mathrm{fC3bC4bCR1}] + [\mathrm{fC3bC4bC4BP}] + [\mathrm{fC3bC4dH}] + [\mathrm{fC3bC4dHL}] + [\mathrm{fC3bC4dCR1}] + [\mathrm{fiC3bC4dCR1}] + [\mathrm{fiC3bC4bCR1}] + [\mathrm{fiC3bC4bC4BP}]} \cdots \\
\dfrac{1}{+ [\mathrm{fC3dgC4bCR1}] + [\mathrm{fC3dgC4bC4BP}] + [\mathrm{fC3bC3bH}] + [\mathrm{fC3bC3bHL}] + [\mathrm{fC3bC3bCR1}] + [\mathrm{fC3biC3bH}] + [\mathrm{fC3biC3bHL}] + [\mathrm{fC3biC3bCR1}]} \cdots \\
\dfrac{1}{+ [\mathrm{fiC3biC3bCR1}] + [\mathrm{fiC3bC3dgCR1}] + [\mathrm{hC3bH}] + [\mathrm{hC3bHL}] + [\mathrm{hC3bCR1}] + [\mathrm{hiC3bCR1}] + [\mathrm{hC3bC4bH}] + [\mathrm{hC3bC4bHL}] + [\mathrm{hC3bC4bCR1}]} \cdots \\
\dfrac{1}{+ [\mathrm{hC3bC4bC4BP}] + [\mathrm{hC3bC4dH}] + [\mathrm{hC3bC4dHL}] + [\mathrm{hC3bC4dCR1}] + [\mathrm{hiC3bC4dCR1}] + [\mathrm{hiC3bC4bCR1}] + [\mathrm{hiC3bC4bC4BP}] + [\mathrm{hC3dgC4bCR1}]} \cdots \\
\dfrac{1}{+ [\mathrm{hC3dgC4bC4BP}] + [\mathrm{hC3bC3bH}] + [\mathrm{hC3bC3bHL}] + [\mathrm{hC3bC3bCR1}] + [\mathrm{hC3biC3bH}] + [\mathrm{hC3biC3bHL}] + [\mathrm{hC3biC3bCR1}] + [\mathrm{hiC3biC3bCR1}]} \cdots \\
\dfrac{1}{+ [\mathrm{hiC3bC3dgCR1}] + [\mathrm{fC4bCR1}] + [\mathrm{nfC4bC4BP}] + [\mathrm{fC4bC4BP}] + [\mathrm{fC4bC4bCR1}] + [\mathrm{fC4bC4bC4BP}] + [\mathrm{fC4bC4dCR1}] + [\mathrm{fC4bC4dC4BP}] + [\mathrm{hC4bCR1}]} \cdots \\
\dfrac{1}{+ [\mathrm{hC4bC4BP}] + [\mathrm{hC4bC4bCR1}] + [\mathrm{hC4bC4bC4BP}] + [\mathrm{hC4bC4dCR1}] + [\mathrm{hC4bC4dC4BP}]} \cdots
\end{array}\right)$$

$$- k^{+}_{\text{C3bH}}[\text{fC3bBb}][\text{H}] + k^{-}_{\text{C3bH}}[\text{fC3bBbH}] + k^{-}_{\text{C3bBbH}_{\text{decay}}}[\text{fC3bBbH}] - k^{+}_{\text{C3bH}}[\text{IgGC3bC3b}][\text{H}] + k^{-}_{\text{C3bH}}[\text{IgGC3bC3bH}]$$

$$- k^{+}_{\text{C3bH}}[\text{IgGC3biC3b}][\text{H}] + k^{-}_{\text{C3bH}}[\text{IgGC3biC3bH}]$$

$$+ \left(\frac{k^{\text{FI}}_{\text{catC3bH}}[\text{I}][\text{IgGC3bC3bH}]}{K^{\text{FI}}_{\text{mC3bH}} + [\text{C3(H}_2\text{O)H}] + [\text{C3(H}_2\text{O)HL}] + [\text{fC3bH}] + [\text{fC3bHL}] + [\text{fC3bCR1}] + [\text{fiC3bCR1}] + [\text{IgGC3bC3bH}] + [\text{IgGC3biC3bH}] + [\text{IgGC3bC3bHL}]} \cdots \right.$$

$$\frac{1}{+[\text{IgGC3biC3bHL}] + [\text{IgGC3bC3bCR1}] + [\text{IgGC3biC3bCR1}] + [\text{IgGiC3biC3bCR1}] + [\text{IgGiC3bC3dgCR1}] + [\text{fC3bC4bH}] + [\text{fC3bC4bHL}]} \cdots$$

$$\frac{1}{+[\text{fC3bC4bCR1}] + [\text{fC3bC4bC4BP}] + [\text{fC3bC4dH}] + [\text{fC3bC4dHL}] + [\text{fC3bC4dCR1}] + [\text{fiC3bC4dCR1}] + [\text{fiC3bC4bCR1}] + [\text{fiC3bC4bC4BP}]} \cdots$$

$$\frac{1}{+[\text{fC3dgC4bCR1}] + [\text{fC3dgC4bC4BP}] + [\text{fC3bC3bH}] + [\text{fC3bC3bHL}] + [\text{fC3bC3bCR1}] + [\text{fC3biC3bH}] + [\text{fC3biC3bHL}] + [\text{fC3biC3bCR1}]} \cdots$$

$$\frac{1}{+[\text{fiC3biC3bCR1}] + [\text{fiC3bC3dgCR1}] + [\text{hC3bH}] + [\text{hC3bHL}] + [\text{hC3bCR1}] + [\text{hiC3bCR1}] + [\text{hC3bC4bH}] + [\text{hC3bC4bHL}] + [\text{hC3bC4bCR1}]} \cdots$$

$$\frac{1}{+[\text{hC3bC4bC4BP}] + [\text{hC3bC4dH}] + [\text{hC3bC4dHL}] + [\text{hC3bC4dCR1}] + [\text{hiC3bC4dCR1}] + [\text{hiC3bC4bCR1}] + [\text{hiC3bC4bC4BP}] + [\text{hC3dgC4bCR1}]} \cdots$$

$$\frac{1}{+[\text{hC3dgC4bC4BP}] + [\text{hC3bC3bH}] + [\text{hC3bC3bHL}] + [\text{hC3bC3bCR1}] + [\text{hC3biC3bH}] + [\text{hC3biC3bHL}] + [\text{hC3biC3bCR1}] + [\text{hiC3biC3bCR1}]} \cdots$$

$$\frac{1}{+[\text{hiC3bC3dgCR1}] + [\text{fC4bCR1}] + [\text{nfC4bC4BP}] + [\text{fC4bC4BP}] + [\text{fC4bC4bCR1}] + [\text{fC4bC4bC4BP}] + [\text{fC4bC4dCR1}] + [\text{fC4bC4dC4BP}] + [\text{hC4bCR1}]} \cdots$$

$$\left. \frac{1}{+[\text{hC4bC4BP}] + [\text{hC4bC4bCR1}] + [\text{hC4bC4bC4BP}] + [\text{hC4bC4dCR1}] + [\text{hC4bC4dC4BP}]} \cdots \right)$$

$$- k^{+}_{\text{C3bH}}[\text{IgGC3biC3b}][\text{H}] + k^{-}_{\text{C3bH}}[\text{IgGC3biC3bH}]$$

$$+ \left(\frac{k^{\text{FI}}_{\text{catC3bH}}[\text{I}][\text{IgGC3biC3bH}]}{K^{\text{FI}}_{\text{mC3bH}} + [\text{C3(H}_2\text{O)H}] + [\text{C3(H}_2\text{O)HL}] + [\text{fC3bH}] + [\text{fC3bHL}] + [\text{fC3bCR1}] + [\text{fiC3bCR1}] + [\text{IgGC3bC3bH}] + [\text{IgGC3biC3bH}] + [\text{IgGC3bC3bHL}]} \cdots \right.$$

$$\frac{1}{+[\text{IgGC3biC3bHL}] + [\text{IgGC3bC3bCR1}] + [\text{IgGC3biC3bCR1}] + [\text{IgGiC3biC3bCR1}] + [\text{IgGiC3bC3dgCR1}] + [\text{fC3bC4bH}] + [\text{fC3bC4bHL}]} \cdots$$

$$\frac{1}{+[\text{fC3bC4bCR1}] + [\text{fC3bC4bC4BP}] + [\text{fC3bC4dH}] + [\text{fC3bC4dHL}] + [\text{fC3bC4dCR1}] + [\text{fiC3bC4dCR1}] + [\text{fiC3bC4bCR1}] + [\text{fiC3bC4bC4BP}]} \cdots$$

$$\frac{1}{+[\text{fC3dgC4bCR1}] + [\text{fC3dgC4bC4BP}] + [\text{fC3bC3bH}] + [\text{fC3bC3bHL}] + [\text{fC3bC3bCR1}] + [\text{fC3biC3bH}] + [\text{fC3biC3bHL}] + [\text{fC3biC3bCR1}]} \cdots$$

$$\frac{1}{+[\text{fiC3biC3bCR1}] + [\text{fiC3bC3dgCR1}] + [\text{hC3bH}] + [\text{hC3bHL}] + [\text{hC3bCR1}] + [\text{hiC3bCR1}] + [\text{hC3bC4bH}] + [\text{hC3bC4bHL}] + [\text{hC3bC4bCR1}]} \cdots$$

$$\frac{1}{+[\text{hC3bC4bC4BP}] + [\text{hC3bC4dH}] + [\text{hC3bC4dHL}] + [\text{hC3bC4dCR1}] + [\text{hiC3bC4dCR1}] + [\text{hiC3bC4bCR1}] + [\text{hiC3bC4bC4BP}] + [\text{hC3dgC4bCR1}]} \cdots$$

$$\frac{1}{+[\text{hC3dgC4bC4BP}] + [\text{hC3bC3bH}] + [\text{hC3bC3bHL}] + [\text{hC3bC3bCR1}] + [\text{hC3biC3bH}] + [\text{hC3biC3bHL}] + [\text{hC3biC3bCR1}] + [\text{hiC3biC3bCR1}]} \cdots$$

$$\frac{1}{+[\text{hiC3bC3dgCR1}] + [\text{fC4bCR1}] + [\text{nfC4bC4BP}] + [\text{fC4bC4BP}] + [\text{fC4bC4bCR1}] + [\text{fC4bC4bC4BP}] + [\text{fC4bC4dCR1}] + [\text{fC4bC4dC4BP}] + [\text{hC4bCR1}]} \cdots$$

$$\left. \frac{1}{+[\text{hC4bC4BP}] + [\text{hC4bC4bCR1}] + [\text{hC4bC4bC4BP}] + [\text{hC4bC4dCR1}] + [\text{hC4bC4dC4BP}]} \cdots \right)$$

$$- k^{+}_{\text{C3bH}}[\text{IgGC3bC3bBb}][\text{H}] + k^{-}_{\text{C3bH}}[\text{IgGC3bC3bBbH}] + k^{-}_{\text{C3bBbH}_{\text{decay}}}[\text{IgGC3bC3bBbH}] - k^{+}_{\text{C3bH}}[\text{fC3bC4b}][\text{H}] + k^{-}_{\text{C3bH}}[\text{fC3bC4bH}]$$

$$+\left(\begin{array}{l}\dfrac{k_{\mathrm{catC3bH}}^{\mathrm{FI}}[\mathrm{I}][\mathrm{fC3bC4bH}]}{K_{\mathrm{mC3bH}}^{\mathrm{FI}}+[\mathrm{C3(H_2O)H}]+[\mathrm{C3(H_2O)HL}]+[\mathrm{fC3bH}]+[\mathrm{fC3bHL}]+[\mathrm{fC3bCR1}]+[\mathrm{fiC3bCR1}]+[\mathrm{IgGC3bC3bH}]+[\mathrm{IgGC3biC3bH}]+[\mathrm{IgGC3bC3bHL}]}\cdots\\ \dfrac{1}{+[\mathrm{IgGC3biC3bHL}]+[\mathrm{IgGC3bC3bCR1}]+[\mathrm{IgGC3biC3bCR1}]+[\mathrm{IgGiC3biC3bCR1}]+[\mathrm{IgGiC3bC3dgCR1}]+[\mathrm{fC3bC4bH}]+[\mathrm{fC3bC4bHL}]}\cdots\\ \dfrac{1}{+[\mathrm{fC3bC4bCR1}]+[\mathrm{fC3bC4bC4BP}]+[\mathrm{fC3bC4dH}]+[\mathrm{fC3bC4dHL}]+[\mathrm{fC3bC4dCR1}]+[\mathrm{fiC3bC4dCR1}]+[\mathrm{fiC3bC4bCR1}]+[\mathrm{fiC3bC4bC4BP}]}\cdots\\ \dfrac{1}{+[\mathrm{fC3dgC4bCR1}]+[\mathrm{fC3dgC4bC4BP}]+[\mathrm{fC3bC3bH}]+[\mathrm{fC3bC3bHL}]+[\mathrm{fC3bC3bCR1}]+[\mathrm{fC3biC3bH}]+[\mathrm{fC3biC3bHL}]+[\mathrm{fC3biC3bCR1}]}\cdots\\ \dfrac{1}{+[\mathrm{fiC3biC3bCR1}]+[\mathrm{fiC3bC3dgCR1}]+[\mathrm{hC3bH}]+[\mathrm{hC3bHL}]+[\mathrm{hC3bCR1}]+[\mathrm{hiC3bCR1}]+[\mathrm{hC3bC4bH}]+[\mathrm{hC3bC4bHL}]+[\mathrm{hC3bC4bCR1}]}\cdots\\ \dfrac{1}{+[\mathrm{hC3bC4bC4BP}]+[\mathrm{hC3bC4dH}]+[\mathrm{hC3bC4dHL}]+[\mathrm{hC3bC4dCR1}]+[\mathrm{hiC3bC4dCR1}]+[\mathrm{hiC3bC4bCR1}]+[\mathrm{hiC3bC4bC4BP}]+[\mathrm{hC3dgC4bCR1}]}\cdots\\ \dfrac{1}{+[\mathrm{hC3dgC4bC4BP}]+[\mathrm{hC3bC3bH}]+[\mathrm{hC3bC3bHL}]+[\mathrm{hC3bC3bCR1}]+[\mathrm{hC3biC3bH}]+[\mathrm{hC3biC3bHL}]+[\mathrm{hC3biC3bCR1}]+[\mathrm{hiC3biC3bCR1}]}\cdots\\ \dfrac{1}{+[\mathrm{hiC3bC3dgCR1}]+[\mathrm{fC4bCR1}]+[\mathrm{nfC4bC4BP}]+[\mathrm{fC4bC4BP}]+[\mathrm{fC4bC4bCR1}]+[\mathrm{fC4bC4bC4BP}]+[\mathrm{fC4bC4dCR1}]+[\mathrm{fC4bC4dC4BP}]+[\mathrm{hC4bCR1}]}\cdots\\ \dfrac{1}{+[\mathrm{hC4bC4BP}]+[\mathrm{hC4bC4bCR1}]+[\mathrm{hC4bC4bC4BP}]+[\mathrm{hC4bC4dCR1}]+[\mathrm{hC4bC4dC4BP}]}\cdots\end{array}\right)$$

$$-k_{\mathrm{C3bH}}^{+}[\mathrm{fC3bC4bBb}][\mathrm{H}]+k_{\mathrm{C3bH}}^{-}[\mathrm{fC3bC4bBbH}]+k_{\mathrm{C3bBbH}_{\mathrm{decay}}}^{-}[\mathrm{fC3bC4bBbH}]-k_{\mathrm{C3bH}}^{+}[\mathrm{fC3bC3b}][\mathrm{H}]+k_{\mathrm{C3bH}}^{-}[\mathrm{fC3bC3bH}]$$

$$+\left(\begin{array}{l}\dfrac{k_{\mathrm{catC3bH}}^{\mathrm{FI}}[\mathrm{I}][\mathrm{fC3bC3bH}]}{K_{\mathrm{mC3bH}}^{\mathrm{FI}}+[\mathrm{C3(H_2O)H}]+[\mathrm{C3(H_2O)HL}]+[\mathrm{fC3bH}]+[\mathrm{fC3bHL}]+[\mathrm{fC3bCR1}]+[\mathrm{fiC3bCR1}]+[\mathrm{IgGC3bC3bH}]+[\mathrm{IgGC3biC3bH}]+[\mathrm{IgGC3bC3bHL}]}\cdots\\ \dfrac{1}{+[\mathrm{IgGC3biC3bHL}]+[\mathrm{IgGC3bC3bCR1}]+[\mathrm{IgGC3biC3bCR1}]+[\mathrm{IgGiC3biC3bCR1}]+[\mathrm{IgGiC3bC3dgCR1}]+[\mathrm{fC3bC4bH}]+[\mathrm{fC3bC4bHL}]}\cdots\\ \dfrac{1}{+[\mathrm{fC3bC4bCR1}]+[\mathrm{fC3bC4bC4BP}]+[\mathrm{fC3bC4dH}]+[\mathrm{fC3bC4dHL}]+[\mathrm{fC3bC4dCR1}]+[\mathrm{fiC3bC4dCR1}]+[\mathrm{fiC3bC4bCR1}]+[\mathrm{fiC3bC4bC4BP}]}\cdots\\ \dfrac{1}{+[\mathrm{fC3dgC4bCR1}]+[\mathrm{fC3dgC4bC4BP}]+[\mathrm{fC3bC3bH}]+[\mathrm{fC3bC3bHL}]+[\mathrm{fC3bC3bCR1}]+[\mathrm{fC3biC3bH}]+[\mathrm{fC3biC3bHL}]+[\mathrm{fC3biC3bCR1}]}\cdots\\ \dfrac{1}{+[\mathrm{fiC3biC3bCR1}]+[\mathrm{fiC3bC3dgCR1}]+[\mathrm{hC3bH}]+[\mathrm{hC3bHL}]+[\mathrm{hC3bCR1}]+[\mathrm{hiC3bCR1}]+[\mathrm{hC3bC4bH}]+[\mathrm{hC3bC4bHL}]+[\mathrm{hC3bC4bCR1}]}\cdots\\ \dfrac{1}{+[\mathrm{hC3bC4bC4BP}]+[\mathrm{hC3bC4dH}]+[\mathrm{hC3bC4dHL}]+[\mathrm{hC3bC4dCR1}]+[\mathrm{hiC3bC4dCR1}]+[\mathrm{hiC3bC4bCR1}]+[\mathrm{hiC3bC4bC4BP}]+[\mathrm{hC3dgC4bCR1}]}\cdots\\ \dfrac{1}{+[\mathrm{hC3dgC4bC4BP}]+[\mathrm{hC3bC3bH}]+[\mathrm{hC3bC3bHL}]+[\mathrm{hC3bC3bCR1}]+[\mathrm{hC3biC3bH}]+[\mathrm{hC3biC3bHL}]+[\mathrm{hC3biC3bCR1}]+[\mathrm{hiC3biC3bCR1}]}\cdots\\ \dfrac{1}{+[\mathrm{hiC3bC3dgCR1}]+[\mathrm{fC4bCR1}]+[\mathrm{nfC4bC4BP}]+[\mathrm{fC4bC4BP}]+[\mathrm{fC4bC4bCR1}]+[\mathrm{fC4bC4bC4BP}]+[\mathrm{fC4bC4dCR1}]+[\mathrm{fC4bC4dC4BP}]+[\mathrm{hC4bCR1}]}\cdots\\ \dfrac{1}{+[\mathrm{hC4bC4BP}]+[\mathrm{hC4bC4bCR1}]+[\mathrm{hC4bC4bC4BP}]+[\mathrm{hC4bC4dCR1}]+[\mathrm{hC4bC4dC4BP}]}\cdots\end{array}\right)$$

$$-k^{+}_{\mathrm{C3bH}}[\mathrm{fC3biC3b}][\mathrm{H}]+k^{-}_{\mathrm{C3bH}}[\mathrm{fC3biC3bH}]$$

$$+\left(\frac{k^{\mathrm{FI}}_{\mathrm{catC3bH}}[\mathrm{I}][\mathrm{fC3biC3bH}]}{K^{\mathrm{FI}}_{\mathrm{mC3bH}}+[\mathrm{C3(H_2O)H}]+[\mathrm{C3(H_2O)HL}]+[\mathrm{fC3bH}]+[\mathrm{fC3bHL}]+[\mathrm{fC3bCR1}]+[\mathrm{fiC3bCR1}]+[\mathrm{IgGC3bC3bH}]+[\mathrm{IgGC3biC3bH}]+[\mathrm{IgGC3bC3bHL}]}\cdots\right.$$
$$\frac{1}{+[\mathrm{IgGC3biC3bHL}]+[\mathrm{IgGC3bC3bCR1}]+[\mathrm{IgGC3biC3bCR1}]+[\mathrm{IgGiC3biC3bCR1}]+[\mathrm{IgGiC3bC3dgCR1}]+[\mathrm{fC3bC4bH}]+[\mathrm{fC3bC4bHL}]}\cdots$$
$$\frac{1}{+[\mathrm{fC3bC4bCR1}]+[\mathrm{fC3bC4bC4BP}]+[\mathrm{fC3bC4dH}]+[\mathrm{fC3bC4dHL}]+[\mathrm{fC3bC4dCR1}]+[\mathrm{fiC3bC4dCR1}]+[\mathrm{fiC3bC4bCR1}]+[\mathrm{fiC3bC4bC4BP}]}\cdots$$
$$\frac{1}{+[\mathrm{fC3dgC4bCR1}]+[\mathrm{fC3dgC4bC4BP}]+[\mathrm{fC3bC3bH}]+[\mathrm{fC3bC3bHL}]+[\mathrm{fC3bC3bCR1}]+[\mathrm{fC3biC3bH}]+[\mathrm{fC3biC3bHL}]+[\mathrm{fC3biC3bCR1}]}\cdots$$
$$\frac{1}{+[\mathrm{fiC3biC3bCR1}]+[\mathrm{fiC3bC3dgCR1}]+[\mathrm{hC3bH}]+[\mathrm{hC3bHL}]+[\mathrm{hC3bCR1}]+[\mathrm{hiC3bCR1}]+[\mathrm{hC3bC4bH}]+[\mathrm{hC3bC4bHL}]+[\mathrm{hC3bC4bCR1}]}\cdots$$
$$\frac{1}{+[\mathrm{hC3bC4bC4BP}]+[\mathrm{hC3bC4dH}]+[\mathrm{hC3bC4dHL}]+[\mathrm{hC3bC4dCR1}]+[\mathrm{hiC3bC4dCR1}]+[\mathrm{hiC3bC4bCR1}]+[\mathrm{hiC3bC4bC4BP}]+[\mathrm{hC3dgC4bCR1}]}\cdots$$
$$\frac{1}{+[\mathrm{hC3dgC4bC4BP}]+[\mathrm{hC3bC3bH}]+[\mathrm{hC3bC3bHL}]+[\mathrm{hC3bC3bCR1}]+[\mathrm{hC3biC3bH}]+[\mathrm{hC3biC3bHL}]+[\mathrm{hC3biC3bCR1}]+[\mathrm{hiC3biC3bCR1}]}\cdots$$
$$\frac{1}{+[\mathrm{hiC3bC3dgCR1}]+[\mathrm{fC4bCR1}]+[\mathrm{nfC4bC4BP}]+[\mathrm{fC4bC4BP}]+[\mathrm{fC4bC4bCR1}]+[\mathrm{fC4bC4bC4BP}]+[\mathrm{fC4bC4dCR1}]+[\mathrm{fC4bC4dC4BP}]+[\mathrm{hC4bCR1}]}\cdots$$
$$\left.\frac{1}{+[\mathrm{hC4bC4BP}]+[\mathrm{hC4bC4bCR1}]+[\mathrm{hC4bC4bC4BP}]+[\mathrm{hC4bC4dCR1}]+[\mathrm{hC4bC4dC4BP}]}\cdots\right)$$

$$-k^{+}_{\mathrm{C3bH}}[\mathrm{fC3bC3bBb}][\mathrm{H}]+k^{-}_{\mathrm{C3bH}}[\mathrm{fC3bC3bBbH}]+k^{-}_{\mathrm{C3bBbH}_{\mathrm{decay}}}[\mathrm{fC3bC3bBbH}]\;-k^{+}_{\mathrm{C3bH}}[\mathrm{hC3bC4b}][\mathrm{H}]+k^{-}_{\mathrm{C3bH}}[\mathrm{hC3bC4bH}]$$

$$+\left(\frac{k^{\mathrm{FI}}_{\mathrm{catC3bH}}[\mathrm{I}][\mathrm{hC3bC4bH}]}{K^{\mathrm{FI}}_{\mathrm{mC3bH}}+[\mathrm{C3(H_2O)H}]+[\mathrm{C3(H_2O)HL}]+[\mathrm{fC3bH}]+[\mathrm{fC3bHL}]+[\mathrm{fC3bCR1}]+[\mathrm{fiC3bCR1}]+[\mathrm{IgGC3bC3bH}]+[\mathrm{IgGC3biC3bH}]+[\mathrm{IgGC3bC3bHL}]}\cdots\right.$$
$$\frac{1}{+[\mathrm{IgGC3biC3bHL}]+[\mathrm{IgGC3bC3bCR1}]+[\mathrm{IgGC3biC3bCR1}]+[\mathrm{IgGiC3biC3bCR1}]+[\mathrm{IgGiC3bC3dgCR1}]+[\mathrm{fC3bC4bH}]+[\mathrm{fC3bC4bHL}]}\cdots$$
$$\frac{1}{+[\mathrm{fC3bC4bCR1}]+[\mathrm{fC3bC4bC4BP}]+[\mathrm{fC3bC4dH}]+[\mathrm{fC3bC4dHL}]+[\mathrm{fC3bC4dCR1}]+[\mathrm{fiC3bC4dCR1}]+[\mathrm{fiC3bC4bCR1}]+[\mathrm{fiC3bC4bC4BP}]}\cdots$$
$$\frac{1}{+[\mathrm{fC3dgC4bCR1}]+[\mathrm{fC3dgC4bC4BP}]+[\mathrm{fC3bC3bH}]+[\mathrm{fC3bC3bHL}]+[\mathrm{fC3bC3bCR1}]+[\mathrm{fC3biC3bH}]+[\mathrm{fC3biC3bHL}]+[\mathrm{fC3biC3bCR1}]}\cdots$$
$$\frac{1}{+[\mathrm{fiC3biC3bCR1}]+[\mathrm{fiC3bC3dgCR1}]+[\mathrm{hC3bH}]+[\mathrm{hC3bHL}]+[\mathrm{hC3bCR1}]+[\mathrm{hiC3bCR1}]+[\mathrm{hC3bC4bH}]+[\mathrm{hC3bC4bHL}]+[\mathrm{hC3bC4bCR1}]}\cdots$$
$$\frac{1}{+[\mathrm{hC3bC4bC4BP}]+[\mathrm{hC3bC4dH}]+[\mathrm{hC3bC4dHL}]+[\mathrm{hC3bC4dCR1}]+[\mathrm{hiC3bC4dCR1}]+[\mathrm{hiC3bC4bCR1}]+[\mathrm{hiC3bC4bC4BP}]+[\mathrm{hC3dgC4bCR1}]}\cdots$$
$$\frac{1}{+[\mathrm{hC3dgC4bC4BP}]+[\mathrm{hC3bC3bH}]+[\mathrm{hC3bC3bHL}]+[\mathrm{hC3bC3bCR1}]+[\mathrm{hC3biC3bH}]+[\mathrm{hC3biC3bHL}]+[\mathrm{hC3biC3bCR1}]+[\mathrm{hiC3biC3bCR1}]}\cdots$$
$$\frac{1}{+[\mathrm{hiC3bC3dgCR1}]+[\mathrm{fC4bCR1}]+[\mathrm{nfC4bC4BP}]+[\mathrm{fC4bC4BP}]+[\mathrm{fC4bC4bCR1}]+[\mathrm{fC4bC4bC4BP}]+[\mathrm{fC4bC4dCR1}]+[\mathrm{fC4bC4dC4BP}]+[\mathrm{hC4bCR1}]}\cdots$$
$$\left.\frac{1}{+[\mathrm{hC4bC4BP}]+[\mathrm{hC4bC4bCR1}]+[\mathrm{hC4bC4bC4BP}]+[\mathrm{hC4bC4dCR1}]+[\mathrm{hC4bC4dC4BP}]}\cdots\right)$$

$$-k^{+}_{\mathrm{C3bH}}[\mathrm{hC3bC4d}][\mathrm{H}]+k^{-}_{\mathrm{C3bH}}[\mathrm{hC3bC4dH}]$$

$$+\left(\frac{k^{\mathrm{FI}}_{\mathrm{catC3bH}}[\mathrm{I}][\mathrm{hC3bC4dH}]}{K^{\mathrm{FI}}_{\mathrm{mC3bH}}+[\mathrm{C3(H_2O)H}]+[\mathrm{C3(H_2O)HL}]+[\mathrm{fC3bH}]+[\mathrm{fC3bHL}]+[\mathrm{fC3bCR1}]+[\mathrm{fiC3bCR1}]+[\mathrm{IgGC3bC3bH}]+[\mathrm{IgGC3biC3bH}]+[\mathrm{IgGC3bC3bHL}]}\cdots\right.$$

$$\frac{1}{+[\mathrm{IgGC3biC3bHL}]+[\mathrm{IgGC3bC3bCR1}]+[\mathrm{IgGC3biC3bCR1}]+[\mathrm{IgGiC3biC3bCR1}]+[\mathrm{IgGiC3bC3dgCR1}]+[\mathrm{fC3bC4bH}]+[\mathrm{fC3bC4bHL}]}\cdots$$

$$\frac{1}{+[\mathrm{fC3bC4bCR1}]+[\mathrm{fC3bC4bC4BP}]+[\mathrm{fC3bC4dH}]+[\mathrm{fC3bC4dHL}]+[\mathrm{fC3bC4dCR1}]+[\mathrm{fiC3bC4dCR1}]+[\mathrm{fiC3bC4bCR1}]+[\mathrm{fiC3bC4bC4BP}]}\cdots$$

$$\frac{1}{+[\mathrm{fC3dgC4bCR1}]+[\mathrm{fC3dgC4bC4BP}]+[\mathrm{fC3bC3bH}]+[\mathrm{fC3bC3bHL}]+[\mathrm{fC3bC3bCR1}]+[\mathrm{fC3biC3bH}]+[\mathrm{fC3biC3bHL}]+[\mathrm{fC3biC3bCR1}]}\cdots$$

$$\frac{1}{+[\mathrm{fiC3biC3bCR1}]+[\mathrm{fiC3bC3dgCR1}]+[\mathrm{hC3bH}]+[\mathrm{hC3bHL}]+[\mathrm{hC3bCR1}]+[\mathrm{hiC3bCR1}]+[\mathrm{hC3bC4bH}]+[\mathrm{hC3bC4bHL}]+[\mathrm{hC3bC4bCR1}]}\cdots$$

$$\frac{1}{+[\mathrm{hC3bC4bC4BP}]+[\mathrm{hC3bC4dH}]+[\mathrm{hC3bC4dHL}]+[\mathrm{hC3bC4dCR1}]+[\mathrm{hiC3bC4dCR1}]+[\mathrm{hiC3bC4bCR1}]+[\mathrm{hiC3bC4bC4BP}]+[\mathrm{hC3dgC4bCR1}]}\cdots$$

$$\frac{1}{+[\mathrm{hC3dgC4bC4BP}]+[\mathrm{hC3bC3bH}]+[\mathrm{hC3bC3bHL}]+[\mathrm{hC3bC3bCR1}]+[\mathrm{hC3biC3bH}]+[\mathrm{hC3biC3bHL}]+[\mathrm{hC3biC3bCR1}]+[\mathrm{hiC3biC3bCR1}]}\cdots$$

$$\frac{1}{+[\mathrm{hiC3bC3dgCR1}]+[\mathrm{fC4bCR1}]+[\mathrm{nfC4bC4BP}]+[\mathrm{fC4bC4BP}]+[\mathrm{fC4bC4bCR1}]+[\mathrm{fC4bC4bC4BP}]+[\mathrm{fC4bC4dCR1}]+[\mathrm{fC4bC4dC4BP}]+[\mathrm{hC4bCR1}]}\cdots$$

$$\left.\frac{1}{+[\mathrm{hC4bC4BP}]+[\mathrm{hC4bC4bCR1}]+[\mathrm{hC4bC4bC4BP}]+[\mathrm{hC4bC4dCR1}]+[\mathrm{hC4bC4dC4BP}]}\cdots\right)$$

$$-k^{+}_{\mathrm{C3bH}}[\mathrm{hC3bC4bBb}][\mathrm{H}]+k^{-}_{\mathrm{C3bH}}[\mathrm{hC3bC4bBbH}]+k^{-}_{\mathrm{C3bBbH}_{\mathrm{decay}}}[\mathrm{hC3bC4bBbH}]-k^{+}_{\mathrm{C3bH}}[\mathrm{hC3bC3b}][\mathrm{H}]+k^{-}_{\mathrm{C3bH}}[\mathrm{hC3bC3bH}]$$

$$+\left(\frac{k^{\mathrm{FI}}_{\mathrm{catC3bH}}[\mathrm{I}][\mathrm{hC3bC3bH}]}{K^{\mathrm{FI}}_{\mathrm{mC3bH}}+[\mathrm{C3(H_2O)H}]+[\mathrm{C3(H_2O)HL}]+[\mathrm{fC3bH}]+[\mathrm{fC3bHL}]+[\mathrm{fC3bCR1}]+[\mathrm{fiC3bCR1}]+[\mathrm{IgGC3bC3bH}]+[\mathrm{IgGC3biC3bH}]+[\mathrm{IgGC3bC3bHL}]}\cdots\right.$$

$$\frac{1}{+[\mathrm{IgGC3biC3bHL}]+[\mathrm{IgGC3bC3bCR1}]+[\mathrm{IgGC3biC3bCR1}]+[\mathrm{IgGiC3biC3bCR1}]+[\mathrm{IgGiC3bC3dgCR1}]+[\mathrm{fC3bC4bH}]+[\mathrm{fC3bC4bHL}]}\cdots$$

$$\frac{1}{+[\mathrm{fC3bC4bCR1}]+[\mathrm{fC3bC4bC4BP}]+[\mathrm{fC3bC4dH}]+[\mathrm{fC3bC4dHL}]+[\mathrm{fC3bC4dCR1}]+[\mathrm{fiC3bC4dCR1}]+[\mathrm{fiC3bC4bCR1}]+[\mathrm{fiC3bC4bC4BP}]}\cdots$$

$$\frac{1}{+[\mathrm{fC3dgC4bCR1}]+[\mathrm{fC3dgC4bC4BP}]+[\mathrm{fC3bC3bH}]+[\mathrm{fC3bC3bHL}]+[\mathrm{fC3bC3bCR1}]+[\mathrm{fC3biC3bH}]+[\mathrm{fC3biC3bHL}]+[\mathrm{fC3biC3bCR1}]}\cdots$$

$$\frac{1}{+[\mathrm{fiC3biC3bCR1}]+[\mathrm{fiC3bC3dgCR1}]+[\mathrm{hC3bH}]+[\mathrm{hC3bHL}]+[\mathrm{hC3bCR1}]+[\mathrm{hiC3bCR1}]+[\mathrm{hC3bC4bH}]+[\mathrm{hC3bC4bHL}]+[\mathrm{hC3bC4bCR1}]}\cdots$$

$$\frac{1}{+[\mathrm{hC3bC4bC4BP}]+[\mathrm{hC3bC4dH}]+[\mathrm{hC3bC4dHL}]+[\mathrm{hC3bC4dCR1}]+[\mathrm{hiC3bC4dCR1}]+[\mathrm{hiC3bC4bCR1}]+[\mathrm{hiC3bC4bC4BP}]+[\mathrm{hC3dgC4bCR1}]}\cdots$$

$$\frac{1}{+[\mathrm{hC3dgC4bC4BP}]+[\mathrm{hC3bC3bH}]+[\mathrm{hC3bC3bHL}]+[\mathrm{hC3bC3bCR1}]+[\mathrm{hC3biC3bH}]+[\mathrm{hC3biC3bHL}]+[\mathrm{hC3biC3bCR1}]+[\mathrm{hiC3biC3bCR1}]}\cdots$$

$$\frac{1}{+[\mathrm{hiC3bC3dgCR1}]+[\mathrm{fC4bCR1}]+[\mathrm{nfC4bC4BP}]+[\mathrm{fC4bC4BP}]+[\mathrm{fC4bC4bCR1}]+[\mathrm{fC4bC4bC4BP}]+[\mathrm{fC4bC4dCR1}]+[\mathrm{fC4bC4dC4BP}]+[\mathrm{hC4bCR1}]}\cdots$$

$$\left.\frac{1}{+[\mathrm{hC4bC4BP}]+[\mathrm{hC4bC4bCR1}]+[\mathrm{hC4bC4bC4BP}]+[\mathrm{hC4bC4dCR1}]+[\mathrm{hC4bC4dC4BP}]}\cdots\right)$$

$$-k^{+}_{\mathrm{C3bH}}[\mathrm{hC3biC3b}][\mathrm{H}]+k^{-}_{\mathrm{C3bH}}[\mathrm{hC3biC3bH}]$$

$$+\left(\frac{k_{\mathrm{catC3bH}}^{\mathrm{FI}}[\mathrm{I}][\mathrm{hC3biC3bH}]}{K_{\mathrm{mC3bH}}^{\mathrm{FI}}+[\mathrm{C3(H_2O)H}]+[\mathrm{C3(H_2O)HL}]+[\mathrm{fC3bH}]+[\mathrm{fC3bHL}]+[\mathrm{fC3bCR1}]+[\mathrm{fiC3bCR1}]+[\mathrm{IgGC3bC3bH}]+[\mathrm{IgGC3biC3bH}]+[\mathrm{IgGC3bC3bHL}]}\cdots\right.$$

$$\frac{1}{+[\mathrm{IgGC3biC3bHL}]+[\mathrm{IgGC3bC3bCR1}]+[\mathrm{IgGC3biC3bCR1}]+[\mathrm{IgGiC3biC3bCR1}]+[\mathrm{IgGiC3bC3dgCR1}]+[\mathrm{fC3bC4bH}]+[\mathrm{fC3bC4bHL}]}\cdots$$

$$\frac{1}{+[\mathrm{fC3bC4bCR1}]+[\mathrm{fC3bC4bC4BP}]+[\mathrm{fC3bC4dH}]+[\mathrm{fC3bC4dHL}]+[\mathrm{fC3bC4dCR1}]+[\mathrm{fiC3bC4dCR1}]+[\mathrm{fiC3bC4bCR1}]+[\mathrm{fiC3bC4bC4BP}]}\cdots$$

$$\frac{1}{+[\mathrm{fC3dgC4bCR1}]+[\mathrm{fC3dgC4bC4BP}]+[\mathrm{fC3bC3bH}]+[\mathrm{fC3bC3bHL}]+[\mathrm{fC3bC3bCR1}]+[\mathrm{fC3biC3bH}]+[\mathrm{fC3biC3bHL}]+[\mathrm{fC3biC3bCR1}]}\cdots$$

$$\frac{1}{+[\mathrm{fiC3biC3bCR1}]+[\mathrm{fiC3bC3dgCR1}]+[\mathrm{hC3bH}]+[\mathrm{hC3bHL}]+[\mathrm{hC3bCR1}]+[\mathrm{hiC3bCR1}]+[\mathrm{hC3bC4bH}]+[\mathrm{hC3bC4bHL}]+[\mathrm{hC3bC4bCR1}]}\cdots$$

$$\frac{1}{+[\mathrm{hC3bC4bC4BP}]+[\mathrm{hC3bC4dH}]+[\mathrm{hC3bC4dHL}]+[\mathrm{hC3bC4dCR1}]+[\mathrm{hiC3bC4dCR1}]+[\mathrm{hiC3bC4bCR1}]+[\mathrm{hiC3bC4bC4BP}]+[\mathrm{hC3dgC4bCR1}]}\cdots$$

$$\frac{1}{+[\mathrm{hC3dgC4bC4BP}]+[\mathrm{hC3bC3bH}]+[\mathrm{hC3bC3bHL}]+[\mathrm{hC3bC3bCR1}]+[\mathrm{hC3biC3bH}]+[\mathrm{hC3biC3bHL}]+[\mathrm{hC3biC3bCR1}]+[\mathrm{hiC3biC3bCR1}]}\cdots$$

$$\frac{1}{+[\mathrm{hiC3bC3dgCR1}]+[\mathrm{fC4bCR1}]+[\mathrm{nfC4bC4BP}]+[\mathrm{fC4bC4BP}]+[\mathrm{fC4bC4bCR1}]+[\mathrm{fC4bC4bC4BP}]+[\mathrm{fC4bC4dCR1}]+[\mathrm{fC4bC4dC4BP}]+[\mathrm{hC4bCR1}]}\cdots$$

$$\left.\frac{1}{+[\mathrm{hC4bC4BP}]+[\mathrm{hC4bC4bCR1}]+[\mathrm{hC4bC4bC4BP}]+[\mathrm{hC4bC4dCR1}]+[\mathrm{hC4bC4dC4BP}]}\cdots\right)$$

$$-k_{\mathrm{C3bH}}^{+}[\mathrm{hC3bC3bBb}][\mathrm{H}]+k_{\mathrm{C3bH}}^{-}[\mathrm{hC3bC3bBbH}]+k_{\mathrm{C3bBbH}_{\mathrm{decay}}}^{-}[\mathrm{hC3bC3bBbH}]-k_{\mathrm{C3bH}}^{+}[\mathrm{hC3b}][\mathrm{H}]+k_{\mathrm{C3bH}}^{-}[\mathrm{hC3bH}]$$

$$+\left(\frac{k_{\mathrm{catC3bH}}^{\mathrm{FI}}[\mathrm{I}][\mathrm{hC3bH}]}{K_{\mathrm{mC3bH}}^{\mathrm{FI}}+[\mathrm{C3(H_2O)H}]+[\mathrm{C3(H_2O)HL}]+[\mathrm{fC3bH}]+[\mathrm{fC3bHL}]+[\mathrm{fC3bCR1}]+[\mathrm{fiC3bCR1}]+[\mathrm{IgGC3bC3bH}]+[\mathrm{IgGC3biC3bH}]+[\mathrm{IgGC3bC3bHL}]}\cdots\right.$$

$$\frac{1}{+[\mathrm{IgGC3biC3bHL}]+[\mathrm{IgGC3bC3bCR1}]+[\mathrm{IgGC3biC3bCR1}]+[\mathrm{IgGiC3biC3bCR1}]+[\mathrm{IgGiC3bC3dgCR1}]+[\mathrm{fC3bC4bH}]+[\mathrm{fC3bC4bHL}]}\cdots$$

$$\frac{1}{+[\mathrm{fC3bC4bCR1}]+[\mathrm{fC3bC4bC4BP}]+[\mathrm{fC3bC4dH}]+[\mathrm{fC3bC4dHL}]+[\mathrm{fC3bC4dCR1}]+[\mathrm{fiC3bC4dCR1}]+[\mathrm{fiC3bC4bCR1}]+[\mathrm{fiC3bC4bC4BP}]}\cdots$$

$$\frac{1}{+[\mathrm{fC3dgC4bCR1}]+[\mathrm{fC3dgC4bC4BP}]+[\mathrm{fC3bC3bH}]+[\mathrm{fC3bC3bHL}]+[\mathrm{fC3bC3bCR1}]+[\mathrm{fC3biC3bH}]+[\mathrm{fC3biC3bHL}]+[\mathrm{fC3biC3bCR1}]}\cdots$$

$$\frac{1}{+[\mathrm{fiC3biC3bCR1}]+[\mathrm{fiC3bC3dgCR1}]+[\mathrm{hC3bH}]+[\mathrm{hC3bHL}]+[\mathrm{hC3bCR1}]+[\mathrm{hiC3bCR1}]+[\mathrm{hC3bC4bH}]+[\mathrm{hC3bC4bHL}]+[\mathrm{hC3bC4bCR1}]}\cdots$$

$$\frac{1}{+[\mathrm{hC3bC4bC4BP}]+[\mathrm{hC3bC4dH}]+[\mathrm{hC3bC4dHL}]+[\mathrm{hC3bC4dCR1}]+[\mathrm{hiC3bC4dCR1}]+[\mathrm{hiC3bC4bCR1}]+[\mathrm{hiC3bC4bC4BP}]+[\mathrm{hC3dgC4bCR1}]}\cdots$$

$$\frac{1}{+[\mathrm{hC3dgC4bC4BP}]+[\mathrm{hC3bC3bH}]+[\mathrm{hC3bC3bHL}]+[\mathrm{hC3bC3bCR1}]+[\mathrm{hC3biC3bH}]+[\mathrm{hC3biC3bHL}]+[\mathrm{hC3biC3bCR1}]+[\mathrm{hiC3biC3bCR1}]}\cdots$$

$$\frac{1}{+[\mathrm{hiC3bC3dgCR1}]+[\mathrm{fC4bCR1}]+[\mathrm{nfC4bC4BP}]+[\mathrm{fC4bC4BP}]+[\mathrm{fC4bC4bCR1}]+[\mathrm{fC4bC4bC4BP}]+[\mathrm{fC4bC4dCR1}]+[\mathrm{fC4bC4dC4BP}]+[\mathrm{hC4bCR1}]}\cdots$$

$$\left.\frac{1}{+[\mathrm{hC4bC4BP}]+[\mathrm{hC4bC4bCR1}]+[\mathrm{hC4bC4bC4BP}]+[\mathrm{hC4bC4dCR1}]+[\mathrm{hC4bC4dC4BP}]}\cdots\right)$$

$$-k_{\mathrm{C3bH}}^{+}[\mathrm{hC3bBb}][\mathrm{H}]+k_{\mathrm{C3bH}}^{-}[\mathrm{hC3bBbH}]+k_{\mathrm{C3bBbH}_{\mathrm{decay}}}^{-}[\mathrm{hC3bBbH}]$$

S288

$$\frac{d[\text{HL}]}{dt} = -k^{+}_{\text{C3bHL}}[\text{C3(H}_2\text{O)}][\text{HL}] + k^{-}_{\text{C3bHL}}[\text{C3(H}_2\text{O)HL}]$$

$$+\left(\frac{k^{\text{FI}}_{\text{catC3bH}}[\text{I}][\text{C3(H}_2\text{O)HL}]}{K^{\text{FI}}_{\text{mC3bH}} + [\text{C3(H}_2\text{O)H}] + [\text{C3(H}_2\text{O)HL}] + [\text{fC3bH}] + [\text{fC3bHL}] + [\text{fC3bCR1}] + [\text{fiC3bCR1}] + [\text{IgGC3bC3bH}] + [\text{IgGC3biC3bH}] + [\text{IgGC3bC3bHL}]}\cdots\right.$$
$$\frac{1}{+[\text{IgGC3biC3bHL}] + [\text{IgGC3bC3bCR1}] + [\text{IgGC3biC3bCR1}] + [\text{IgGiC3biC3bCR1}] + [\text{IgGiC3bC3dgCR1}] + [\text{fC3bC4bH}] + [\text{fC3bC4bHL}]}\cdots$$
$$\frac{1}{+[\text{fC3bC4bCR1}] + [\text{fC3bC4bC4BP}] + [\text{fC3bC4dH}] + [\text{fC3bC4dHL}] + [\text{fC3bC4dCR1}] + [\text{fiC3bC4dCR1}] + [\text{fiC3bC4bCR1}] + [\text{fiC3bC4bC4BP}]}\cdots$$
$$\frac{1}{+[\text{fC3dgC4bCR1}] + [\text{fC3dgC4bC4BP}] + [\text{fC3bC3bH}] + [\text{fC3bC3bHL}] + [\text{fC3bC3bCR1}] + [\text{fC3biC3bH}] + [\text{fC3biC3bHL}] + [\text{fC3biC3bCR1}]}\cdots$$
$$\frac{1}{+[\text{fiC3biC3bCR1}] + [\text{fiC3bC3dgCR1}] + [\text{hC3bH}] + [\text{hC3bHL}] + [\text{hC3bCR1}] + [\text{hiC3bCR1}] + [\text{hC3bC4bH}] + [\text{hC3bC4bHL}] + [\text{hC3bC4bCR1}]}\cdots$$
$$\frac{1}{+[\text{hC3bC4bC4BP}] + [\text{hC3bC4dH}] + [\text{hC3bC4dHL}] + [\text{hC3bC4dCR1}] + [\text{hiC3bC4dCR1}] + [\text{hiC3bC4bCR1}] + [\text{hiC3bC4bC4BP}] + [\text{hC3dgC4bCR1}]}\cdots$$
$$\frac{1}{+[\text{hC3dgC4bC4BP}] + [\text{hC3bC3bH}] + [\text{hC3bC3bHL}] + [\text{hC3bC3bCR1}] + [\text{hC3biC3bH}] + [\text{hC3biC3bHL}] + [\text{hC3biC3bCR1}] + [\text{hiC3biC3bCR1}]}\cdots$$
$$\frac{1}{+[\text{hiC3bC3dgCR1}] + [\text{fC4bCR1}] + [\text{nfC4bC4BP}] + [\text{fC4bC4BP}] + [\text{fC4bC4bCR1}] + [\text{fC4bC4bC4BP}] + [\text{fC4bC4dCR1}] + [\text{fC4bC4dC4BP}] + [\text{hC4bCR1}]}\cdots$$
$$\left.\frac{1}{+[\text{hC4bC4BP}] + [\text{hC4bC4bCR1}] + [\text{hC4bC4bC4BP}] + [\text{hC4bC4dCR1}] + [\text{hC4bC4dC4BP}]}\cdots\right)$$

$$-k^{+}_{\text{C3bHL}}[\text{C3(H}_2\text{O)Bb}][\text{HL}] + k^{-}_{\text{C3bHL}}[\text{C3(H}_2\text{O)BbHL}] + k^{-}_{\text{C3bBbHL}_{\text{decay}}}[\text{C3(H}_2\text{O)BbHL}] - k^{+}_{\text{C3bHL}}[\text{fC3b}][\text{HL}] + k^{-}_{\text{C3bHL}}[\text{fC3bHL}]$$

$$+\left(\frac{k^{\text{FI}}_{\text{catC3bH}}[\text{I}][\text{fC3bHL}]}{K^{\text{FI}}_{\text{mC3bH}} + [\text{C3(H}_2\text{O)H}] + [\text{C3(H}_2\text{O)HL}] + [\text{fC3bH}] + [\text{fC3bHL}] + [\text{fC3bCR1}] + [\text{fiC3bCR1}] + [\text{IgGC3bC3bH}] + [\text{IgGC3biC3bH}] + [\text{IgGC3bC3bHL}]}\cdots\right.$$
$$\frac{1}{+[\text{IgGC3biC3bHL}] + [\text{IgGC3bC3bCR1}] + [\text{IgGC3biC3bCR1}] + [\text{IgGiC3biC3bCR1}] + [\text{IgGiC3bC3dgCR1}] + [\text{fC3bC4bH}] + [\text{fC3bC4bHL}]}\cdots$$
$$\frac{1}{+[\text{fC3bC4bCR1}] + [\text{fC3bC4bC4BP}] + [\text{fC3bC4dH}] + [\text{fC3bC4dHL}] + [\text{fC3bC4dCR1}] + [\text{fiC3bC4dCR1}] + [\text{fiC3bC4bCR1}] + [\text{fiC3bC4bC4BP}]}\cdots$$
$$\frac{1}{+[\text{fC3dgC4bCR1}] + [\text{fC3dgC4bC4BP}] + [\text{fC3bC3bH}] + [\text{fC3bC3bHL}] + [\text{fC3bC3bCR1}] + [\text{fC3biC3bH}] + [\text{fC3biC3bHL}] + [\text{fC3biC3bCR1}]}\cdots$$
$$\frac{1}{+[\text{fiC3biC3bCR1}] + [\text{fiC3bC3dgCR1}] + [\text{hC3bH}] + [\text{hC3bHL}] + [\text{hC3bCR1}] + [\text{hiC3bCR1}] + [\text{hC3bC4bH}] + [\text{hC3bC4bHL}] + [\text{hC3bC4bCR1}]}\cdots$$
$$\frac{1}{+[\text{hC3bC4bC4BP}] + [\text{hC3bC4dH}] + [\text{hC3bC4dHL}] + [\text{hC3bC4dCR1}] + [\text{hiC3bC4dCR1}] + [\text{hiC3bC4bCR1}] + [\text{hiC3bC4bC4BP}] + [\text{hC3dgC4bCR1}]}\cdots$$
$$\frac{1}{+[\text{hC3dgC4bC4BP}] + [\text{hC3bC3bH}] + [\text{hC3bC3bHL}] + [\text{hC3bC3bCR1}] + [\text{hC3biC3bH}] + [\text{hC3biC3bHL}] + [\text{hC3biC3bCR1}] + [\text{hiC3biC3bCR1}]}\cdots$$
$$\frac{1}{+[\text{hiC3bC3dgCR1}] + [\text{fC4bCR1}] + [\text{nfC4bC4BP}] + [\text{fC4bC4BP}] + [\text{fC4bC4bCR1}] + [\text{fC4bC4bC4BP}] + [\text{fC4bC4dCR1}] + [\text{fC4bC4dC4BP}] + [\text{hC4bCR1}]}\cdots$$
$$\left.\frac{1}{+[\text{hC4bC4BP}] + [\text{hC4bC4bCR1}] + [\text{hC4bC4bC4BP}] + [\text{hC4bC4dCR1}] + [\text{hC4bC4dC4BP}]}\cdots\right)$$

$$-k^{+}_{\text{C3bHL}}[\text{fC3bBb}][\text{HL}] + k^{-}_{\text{C3bHL}}[\text{fC3bBbHL}] + k^{-}_{\text{C3bBbHL}_{\text{decay}}}[\text{fC3bBbHL}] - k^{+}_{\text{C3bHL}}[\text{IgGC3bC3b}][\text{HL}] + k^{-}_{\text{C3bHL}}[\text{IgGC3bC3bHL}]$$

$$+\left(\frac{k^{\text{FI}}_{\text{catC3bH}}[\text{I}][\text{IgGC3bC3bHL}]}{K^{\text{FI}}_{\text{mC3bH}}+[\text{C3(H}_2\text{O)H}]+[\text{C3(H}_2\text{O)HL}]+[\text{fC3bH}]+[\text{fC3bHL}]+[\text{fC3bCR1}]+[\text{fiC3bCR1}]+[\text{IgGC3bC3bH}]+[\text{IgGC3biC3bH}]+[\text{IgGC3bC3bHL}]}\ldots\right.$$

$$\frac{1}{+[\text{IgGC3biC3bHL}]+[\text{IgGC3bC3bCR1}]+[\text{IgGC3biC3bCR1}]+[\text{IgGiC3biC3bCR1}]+[\text{IgGiC3bC3dgCR1}]+[\text{fC3bC4bH}]+[\text{fC3bC4bHL}]}\ldots$$

$$\frac{1}{+[\text{fC3bC4bCR1}]+[\text{fC3bC4bC4BP}]+[\text{fC3bC4dH}]+[\text{fC3bC4dHL}]+[\text{fC3bC4dCR1}]+[\text{fiC3bC4dCR1}]+[\text{fiC3bC4bCR1}]+[\text{fiC3bC4bC4BP}]}\ldots$$

$$\frac{1}{+[\text{fC3dgC4bCR1}]+[\text{fC3dgC4bC4BP}]+[\text{fC3bC3bH}]+[\text{fC3bC3bHL}]+[\text{fC3bC3bCR1}]+[\text{fC3biC3bH}]+[\text{fC3biC3bHL}]+[\text{fC3biC3bCR1}]}\ldots$$

$$\frac{1}{+[\text{fiC3biC3bCR1}]+[\text{fiC3bC3dgCR1}]+[\text{hC3bH}]+[\text{hC3bHL}]+[\text{hC3bCR1}]+[\text{hiC3bCR1}]+[\text{hC3bC4bH}]+[\text{hC3bC4bHL}]+[\text{hC3bC4bCR1}]}\ldots$$

$$\frac{1}{+[\text{hC3bC4bC4BP}]+[\text{hC3bC4dH}]+[\text{hC3bC4dHL}]+[\text{hC3bC4dCR1}]+[\text{hiC3bC4dCR1}]+[\text{hiC3bC4bCR1}]+[\text{hiC3bC4bC4BP}]+[\text{hC3dgC4bCR1}]}\ldots$$

$$\frac{1}{+[\text{hC3dgC4bC4BP}]+[\text{hC3bC3bH}]+[\text{hC3bC3bHL}]+[\text{hC3bC3bCR1}]+[\text{hC3biC3bH}]+[\text{hC3biC3bHL}]+[\text{hC3biC3bCR1}]+[\text{hiC3biC3bCR1}]}\ldots$$

$$\frac{1}{+[\text{hiC3bC3dgCR1}]+[\text{fC4bCR1}]+[\text{nfC4bC4BP}]+[\text{fC4bC4BP}]+[\text{fC4bC4bCR1}]+[\text{fC4bC4bC4BP}]+[\text{fC4bC4dCR1}]+[\text{fC4bC4dC4BP}]+[\text{hC4bCR1}]}\ldots$$

$$\left.\frac{1}{+[\text{hC4bC4BP}]+[\text{hC4bC4bCR1}]+[\text{hC4bC4bC4BP}]+[\text{hC4bC4dCR1}]+[\text{hC4bC4dC4BP}]}\ldots\right)$$

$$-k^{+}_{\text{C3bHL}}[\text{IgGC3biC3b}][\text{HL}]+k^{-}_{\text{C3bHL}}[\text{IgGC3biC3bHL}]$$

$$+\left(\frac{k^{\text{FI}}_{\text{catC3bH}}[\text{I}][\text{IgGC3biC3bHL}]}{K^{\text{FI}}_{\text{mC3bH}}+[\text{C3(H}_2\text{O)H}]+[\text{C3(H}_2\text{O)HL}]+[\text{fC3bH}]+[\text{fC3bHL}]+[\text{fC3bCR1}]+[\text{fiC3bCR1}]+[\text{IgGC3bC3bH}]+[\text{IgGC3biC3bH}]+[\text{IgGC3bC3bHL}]}\ldots\right.$$

$$\frac{1}{+[\text{IgGC3biC3bHL}]+[\text{IgGC3bC3bCR1}]+[\text{IgGC3biC3bCR1}]+[\text{IgGiC3biC3bCR1}]+[\text{IgGiC3bC3dgCR1}]+[\text{fC3bC4bH}]+[\text{fC3bC4bHL}]}\ldots$$

$$\frac{1}{+[\text{fC3bC4bCR1}]+[\text{fC3bC4bC4BP}]+[\text{fC3bC4dH}]+[\text{fC3bC4dHL}]+[\text{fC3bC4dCR1}]+[\text{fiC3bC4dCR1}]+[\text{fiC3bC4bCR1}]+[\text{fiC3bC4bC4BP}]}\ldots$$

$$\frac{1}{+[\text{fC3dgC4bCR1}]+[\text{fC3dgC4bC4BP}]+[\text{fC3bC3bH}]+[\text{fC3bC3bHL}]+[\text{fC3bC3bCR1}]+[\text{fC3biC3bH}]+[\text{fC3biC3bHL}]+[\text{fC3biC3bCR1}]}\ldots$$

$$\frac{1}{+[\text{fiC3biC3bCR1}]+[\text{fiC3bC3dgCR1}]+[\text{hC3bH}]+[\text{hC3bHL}]+[\text{hC3bCR1}]+[\text{hiC3bCR1}]+[\text{hC3bC4bH}]+[\text{hC3bC4bHL}]+[\text{hC3bC4bCR1}]}\ldots$$

$$\frac{1}{+[\text{hC3bC4bC4BP}]+[\text{hC3bC4dH}]+[\text{hC3bC4dHL}]+[\text{hC3bC4dCR1}]+[\text{hiC3bC4dCR1}]+[\text{hiC3bC4bCR1}]+[\text{hiC3bC4bC4BP}]+[\text{hC3dgC4bCR1}]}\ldots$$

$$\frac{1}{+[\text{hC3dgC4bC4BP}]+[\text{hC3bC3bH}]+[\text{hC3bC3bHL}]+[\text{hC3bC3bCR1}]+[\text{hC3biC3bH}]+[\text{hC3biC3bHL}]+[\text{hC3biC3bCR1}]+[\text{hiC3biC3bCR1}]}\ldots$$

$$\frac{1}{+[\text{hiC3bC3dgCR1}]+[\text{fC4bCR1}]+[\text{nfC4bC4BP}]+[\text{fC4bC4BP}]+[\text{fC4bC4bCR1}]+[\text{fC4bC4bC4BP}]+[\text{fC4bC4dCR1}]+[\text{fC4bC4dC4BP}]+[\text{hC4bCR1}]}\ldots$$

$$\left.\frac{1}{+[\text{hC4bC4BP}]+[\text{hC4bC4bCR1}]+[\text{hC4bC4bC4BP}]+[\text{hC4bC4dCR1}]+[\text{hC4bC4dC4BP}]}\ldots\right)$$

$$-k^{+}_{\text{C3bHL}}[\text{IgGC3bC3bBb}][\text{HL}]+k^{-}_{\text{C3bHL}}[\text{IgGC3bC3bBbHL}]+k^{-}_{\text{C3bBbHL}_{\text{decay}}}[\text{IgGC3bC3bBbHL}]-k^{+}_{\text{C3bHL}}[\text{fC3bC4b}][\text{HL}]+k^{-}_{\text{C3bHL}}[\text{fC3bC4bHL}]$$

$$+\left(\frac{k^{\mathrm{FI}}_{\mathrm{catC3bH}}[\mathrm{I}][\mathrm{fC3bC4bHL}]}{K^{\mathrm{FI}}_{\mathrm{mC3bH}}+[\mathrm{C3(H_2O)H}]+[\mathrm{C3(H_2O)HL}]+[\mathrm{fC3bH}]+[\mathrm{fC3bHL}]+[\mathrm{fC3bCR1}]+[\mathrm{fiC3bCR1}]+[\mathrm{IgGC3bC3bH}]+[\mathrm{IgGC3biC3bH}]+[\mathrm{IgGC3bC3bHL}]}\cdots\right.$$

$$\frac{1}{+[\mathrm{IgGC3biC3bHL}]+[\mathrm{IgGC3bC3bCR1}]+[\mathrm{IgGC3biC3bCR1}]+[\mathrm{IgGiC3biC3bCR1}]+[\mathrm{IgGiC3bC3dgCR1}]+[\mathrm{fC3bC4bH}]+[\mathrm{fC3bC4bHL}]}\cdots$$

$$\frac{1}{+[\mathrm{fC3bC4bCR1}]+[\mathrm{fC3bC4bC4BP}]+[\mathrm{fC3bC4dH}]+[\mathrm{fC3bC4dHL}]+[\mathrm{fC3bC4dCR1}]+[\mathrm{fiC3bC4dCR1}]+[\mathrm{fiC3bC4bCR1}]+[\mathrm{fiC3bC4bC4BP}]}\cdots$$

$$\frac{1}{+[\mathrm{fC3dgC4bCR1}]+[\mathrm{fC3dgC4bC4BP}]+[\mathrm{fC3bC3bH}]+[\mathrm{fC3bC3bHL}]+[\mathrm{fC3bC3bCR1}]+[\mathrm{fC3biC3bH}]+[\mathrm{fC3biC3bHL}]+[\mathrm{fC3biC3bCR1}]}\cdots$$

$$\frac{1}{+[\mathrm{fiC3biC3bCR1}]+[\mathrm{fiC3bC3dgCR1}]+[\mathrm{hC3bH}]+[\mathrm{hC3bHL}]+[\mathrm{hC3bCR1}]+[\mathrm{hiC3bCR1}]+[\mathrm{hC3bC4bH}]+[\mathrm{hC3bC4bHL}]+[\mathrm{hC3bC4bCR1}]}\cdots$$

$$\frac{1}{+[\mathrm{hC3bC4bC4BP}]+[\mathrm{hC3bC4dH}]+[\mathrm{hC3bC4dHL}]+[\mathrm{hC3bC4dCR1}]+[\mathrm{hiC3bC4dCR1}]+[\mathrm{hiC3bC4bCR1}]+[\mathrm{hiC3bC4bC4BP}]+[\mathrm{hC3dgC4bCR1}]}\cdots$$

$$\frac{1}{+[\mathrm{hC3dgC4bC4BP}]+[\mathrm{hC3bC3bH}]+[\mathrm{hC3bC3bHL}]+[\mathrm{hC3bC3bCR1}]+[\mathrm{hC3biC3bH}]+[\mathrm{hC3biC3bHL}]+[\mathrm{hC3biC3bCR1}]+[\mathrm{hiC3biC3bCR1}]}\cdots$$

$$\frac{1}{+[\mathrm{hiC3bC3dgCR1}]+[\mathrm{fC4bCR1}]+[\mathrm{nfC4bC4BP}]+[\mathrm{fC4bC4BP}]+[\mathrm{fC4bC4bCR1}]+[\mathrm{fC4bC4bC4BP}]+[\mathrm{fC4bC4dCR1}]+[\mathrm{fC4bC4dC4BP}]+[\mathrm{hC4bCR1}]}\cdots$$

$$\left.\frac{1}{+[\mathrm{hC4bC4BP}]+[\mathrm{hC4bC4bCR1}]+[\mathrm{hC4bC4bC4BP}]+[\mathrm{hC4bC4dCR1}]+[\mathrm{hC4bC4dC4BP}]}\cdots\right)$$

$$-k^{+}_{\mathrm{C3bHL}}[\mathrm{fC3bC4d}][\mathrm{HL}]+k^{-}_{\mathrm{C3bHL}}[\mathrm{fC3bC4dHL}]$$

$$+\left(\frac{k^{\mathrm{FI}}_{\mathrm{catC3bH}}[\mathrm{I}][\mathrm{fC3bC4dHL}]}{K^{\mathrm{FI}}_{\mathrm{mC3bH}}+[\mathrm{C3(H_2O)H}]+[\mathrm{C3(H_2O)HL}]+[\mathrm{fC3bH}]+[\mathrm{fC3bHL}]+[\mathrm{fC3bCR1}]+[\mathrm{fiC3bCR1}]+[\mathrm{IgGC3bC3bH}]+[\mathrm{IgGC3biC3bH}]+[\mathrm{IgGC3bC3bHL}]}\cdots\right.$$

$$\frac{1}{+[\mathrm{IgGC3biC3bHL}]+[\mathrm{IgGC3bC3bCR1}]+[\mathrm{IgGC3biC3bCR1}]+[\mathrm{IgGiC3biC3bCR1}]+[\mathrm{IgGiC3bC3dgCR1}]+[\mathrm{fC3bC4bH}]+[\mathrm{fC3bC4bHL}]}\cdots$$

$$\frac{1}{+[\mathrm{fC3bC4bCR1}]+[\mathrm{fC3bC4bC4BP}]+[\mathrm{fC3bC4dH}]+[\mathrm{fC3bC4dHL}]+[\mathrm{fC3bC4dCR1}]+[\mathrm{fiC3bC4dCR1}]+[\mathrm{fiC3bC4bCR1}]+[\mathrm{fiC3bC4bC4BP}]}\cdots$$

$$\frac{1}{+[\mathrm{fC3dgC4bCR1}]+[\mathrm{fC3dgC4bC4BP}]+[\mathrm{fC3bC3bH}]+[\mathrm{fC3bC3bHL}]+[\mathrm{fC3bC3bCR1}]+[\mathrm{fC3biC3bH}]+[\mathrm{fC3biC3bHL}]+[\mathrm{fC3biC3bCR1}]}\cdots$$

$$\frac{1}{+[\mathrm{fiC3biC3bCR1}]+[\mathrm{fiC3bC3dgCR1}]+[\mathrm{hC3bH}]+[\mathrm{hC3bHL}]+[\mathrm{hC3bCR1}]+[\mathrm{hiC3bCR1}]+[\mathrm{hC3bC4bH}]+[\mathrm{hC3bC4bHL}]+[\mathrm{hC3bC4bCR1}]}\cdots$$

$$\frac{1}{+[\mathrm{hC3bC4bC4BP}]+[\mathrm{hC3bC4dH}]+[\mathrm{hC3bC4dHL}]+[\mathrm{hC3bC4dCR1}]+[\mathrm{hiC3bC4dCR1}]+[\mathrm{hiC3bC4bCR1}]+[\mathrm{hiC3bC4bC4BP}]+[\mathrm{hC3dgC4bCR1}]}\cdots$$

$$\frac{1}{+[\mathrm{hC3dgC4bC4BP}]+[\mathrm{hC3bC3bH}]+[\mathrm{hC3bC3bHL}]+[\mathrm{hC3bC3bCR1}]+[\mathrm{hC3biC3bH}]+[\mathrm{hC3biC3bHL}]+[\mathrm{hC3biC3bCR1}]+[\mathrm{hiC3biC3bCR1}]}\cdots$$

$$\frac{1}{+[\mathrm{hiC3bC3dgCR1}]+[\mathrm{fC4bCR1}]+[\mathrm{nfC4bC4BP}]+[\mathrm{fC4bC4BP}]+[\mathrm{fC4bC4bCR1}]+[\mathrm{fC4bC4bC4BP}]+[\mathrm{fC4bC4dCR1}]+[\mathrm{fC4bC4dC4BP}]+[\mathrm{hC4bCR1}]}\cdots$$

$$\left.\frac{1}{+[\mathrm{hC4bC4BP}]+[\mathrm{hC4bC4bCR1}]+[\mathrm{hC4bC4bC4BP}]+[\mathrm{hC4bC4dCR1}]+[\mathrm{hC4bC4dC4BP}]}\cdots\right)$$

$$-k^{+}_{\mathrm{C3bHL}}[\mathrm{fC3bC4bBb}][\mathrm{HL}]+k^{-}_{\mathrm{C3bHL}}[\mathrm{fC3bC4bBbHL}]+k^{-}_{\mathrm{C3bBbHL}_{\mathrm{decay}}}[\mathrm{fC3bC4bBbHL}]-k^{+}_{\mathrm{C3bHL}}[\mathrm{fC3bC3b}][\mathrm{HL}]+k^{-}_{\mathrm{C3bHL}}[\mathrm{fC3bC3bHL}]$$

$$
+\left(\begin{array}{l}
\dfrac{k_{\mathrm{catC3bH}}^{\mathrm{FI}}[\mathrm{I}][\mathrm{fC3bC3bHL}]}{K_{\mathrm{mC3bH}}^{\mathrm{FI}}+[\mathrm{C3(H_2O)H}]+[\mathrm{C3(H_2O)HL}]+[\mathrm{fC3bH}]+[\mathrm{fC3bHL}]+[\mathrm{fC3bCR1}]+[\mathrm{fiC3bCR1}]+[\mathrm{IgGC3bC3bH}]+[\mathrm{IgGC3biC3bH}]+[\mathrm{IgGC3bC3bHL}]}\cdots \\
\dfrac{1}{+[\mathrm{IgGC3biC3bHL}]+[\mathrm{IgGC3bC3bCR1}]+[\mathrm{IgGC3biC3bCR1}]+[\mathrm{IgGiC3biC3bCR1}]+[\mathrm{IgGiC3bC3dgCR1}]+[\mathrm{fC3bC4bH}]+[\mathrm{fC3bC4bHL}]}\cdots \\
\dfrac{1}{+[\mathrm{fC3bC4bCR1}]+[\mathrm{fC3bC4bC4BP}]+[\mathrm{fC3bC4dH}]+[\mathrm{fC3bC4dHL}]+[\mathrm{fC3bC4dCR1}]+[\mathrm{fiC3bC4dCR1}]+[\mathrm{fiC3bC4bCR1}]+[\mathrm{fiC3bC4bC4BP}]}\cdots \\
\dfrac{1}{+[\mathrm{fC3dgC4bCR1}]+[\mathrm{fC3dgC4bC4BP}]+[\mathrm{fC3bC3bH}]+[\mathrm{fC3bC3bHL}]+[\mathrm{fC3bC3bCR1}]+[\mathrm{fC3biC3bH}]+[\mathrm{fC3biC3bHL}]+[\mathrm{fC3biC3bCR1}]}\cdots \\
\dfrac{1}{+[\mathrm{fiC3biC3bCR1}]+[\mathrm{fiC3bC3dgCR1}]+[\mathrm{hC3bH}]+[\mathrm{hC3bHL}]+[\mathrm{hC3bCR1}]+[\mathrm{hiC3bCR1}]+[\mathrm{hC3bC4bH}]+[\mathrm{hC3bC4bHL}]+[\mathrm{hC3bC4bCR1}]}\cdots \\
\dfrac{1}{+[\mathrm{hC3bC4bC4BP}]+[\mathrm{hC3bC4dH}]+[\mathrm{hC3bC4dHL}]+[\mathrm{hC3bC4dCR1}]+[\mathrm{hiC3bC4dCR1}]+[\mathrm{hiC3bC4bCR1}]+[\mathrm{hiC3bC4bC4BP}]+[\mathrm{hC3dgC4bCR1}]}\cdots \\
\dfrac{1}{+[\mathrm{hC3dgC4bC4BP}]+[\mathrm{hC3bC3bH}]+[\mathrm{hC3bC3bHL}]+[\mathrm{hC3bC3bCR1}]+[\mathrm{hC3biC3bH}]+[\mathrm{hC3biC3bHL}]+[\mathrm{hC3biC3bCR1}]+[\mathrm{hiC3biC3bCR1}]}\cdots \\
\dfrac{1}{+[\mathrm{hiC3bC3dgCR1}]+[\mathrm{fC4bCR1}]+[\mathrm{nfC4bC4BP}]+[\mathrm{fC4bC4BP}]+[\mathrm{fC4bC4bCR1}]+[\mathrm{fC4bC4bC4BP}]+[\mathrm{fC4bC4dCR1}]+[\mathrm{fC4bC4dC4BP}]+[\mathrm{hC4bCR1}]}\cdots \\
\dfrac{1}{+[\mathrm{hC4bC4BP}]+[\mathrm{hC4bC4bCR1}]+[\mathrm{hC4bC4bC4BP}]+[\mathrm{hC4bC4dCR1}]+[\mathrm{hC4bC4dC4BP}]}\cdots
\end{array}\right)
$$

$$
-k_{\mathrm{C3bHL}}^{+}[\mathrm{fC3biC3b}][\mathrm{HL}]+k_{\mathrm{C3bHL}}^{-}[\mathrm{fC3biC3bHL}]
$$

$$
+\left(\begin{array}{l}
\dfrac{k_{\mathrm{catC3bH}}^{\mathrm{FI}}[\mathrm{I}][\mathrm{fC3biC3bHL}]}{K_{\mathrm{mC3bH}}^{\mathrm{FI}}+[\mathrm{C3(H_2O)H}]+[\mathrm{C3(H_2O)HL}]+[\mathrm{fC3bH}]+[\mathrm{fC3bHL}]+[\mathrm{fC3bCR1}]+[\mathrm{fiC3bCR1}]+[\mathrm{IgGC3bC3bH}]+[\mathrm{IgGC3biC3bH}]+[\mathrm{IgGC3bC3bHL}]}\cdots \\
\dfrac{1}{+[\mathrm{IgGC3biC3bHL}]+[\mathrm{IgGC3bC3bCR1}]+[\mathrm{IgGC3biC3bCR1}]+[\mathrm{IgGiC3biC3bCR1}]+[\mathrm{IgGiC3bC3dgCR1}]+[\mathrm{fC3bC4bH}]+[\mathrm{fC3bC4bHL}]}\cdots \\
\dfrac{1}{+[\mathrm{fC3bC4bCR1}]+[\mathrm{fC3bC4bC4BP}]+[\mathrm{fC3bC4dH}]+[\mathrm{fC3bC4dHL}]+[\mathrm{fC3bC4dCR1}]+[\mathrm{fiC3bC4dCR1}]+[\mathrm{fiC3bC4bCR1}]+[\mathrm{fiC3bC4bC4BP}]}\cdots \\
\dfrac{1}{+[\mathrm{fC3dgC4bCR1}]+[\mathrm{fC3dgC4bC4BP}]+[\mathrm{fC3bC3bH}]+[\mathrm{fC3bC3bHL}]+[\mathrm{fC3bC3bCR1}]+[\mathrm{fC3biC3bH}]+[\mathrm{fC3biC3bHL}]+[\mathrm{fC3biC3bCR1}]}\cdots \\
\dfrac{1}{+[\mathrm{fiC3biC3bCR1}]+[\mathrm{fiC3bC3dgCR1}]+[\mathrm{hC3bH}]+[\mathrm{hC3bHL}]+[\mathrm{hC3bCR1}]+[\mathrm{hiC3bCR1}]+[\mathrm{hC3bC4bH}]+[\mathrm{hC3bC4bHL}]+[\mathrm{hC3bC4bCR1}]}\cdots \\
\dfrac{1}{+[\mathrm{hC3bC4bC4BP}]+[\mathrm{hC3bC4dH}]+[\mathrm{hC3bC4dHL}]+[\mathrm{hC3bC4dCR1}]+[\mathrm{hiC3bC4dCR1}]+[\mathrm{hiC3bC4bCR1}]+[\mathrm{hiC3bC4bC4BP}]+[\mathrm{hC3dgC4bCR1}]}\cdots \\
\dfrac{1}{+[\mathrm{hC3dgC4bC4BP}]+[\mathrm{hC3bC3bH}]+[\mathrm{hC3bC3bHL}]+[\mathrm{hC3bC3bCR1}]+[\mathrm{hC3biC3bH}]+[\mathrm{hC3biC3bHL}]+[\mathrm{hC3biC3bCR1}]+[\mathrm{hiC3biC3bCR1}]}\cdots \\
\dfrac{1}{+[\mathrm{hiC3bC3dgCR1}]+[\mathrm{fC4bCR1}]+[\mathrm{nfC4bC4BP}]+[\mathrm{fC4bC4BP}]+[\mathrm{fC4bC4bCR1}]+[\mathrm{fC4bC4bC4BP}]+[\mathrm{fC4bC4dCR1}]+[\mathrm{fC4bC4dC4BP}]+[\mathrm{hC4bCR1}]}\cdots \\
\dfrac{1}{+[\mathrm{hC4bC4BP}]+[\mathrm{hC4bC4bCR1}]+[\mathrm{hC4bC4bC4BP}]+[\mathrm{hC4bC4dCR1}]+[\mathrm{hC4bC4dC4BP}]}\cdots
\end{array}\right)
$$

$$
-k_{\mathrm{C3bHL}}^{+}[\mathrm{fC3bC3bBb}][\mathrm{HL}]+k_{\mathrm{C3bHL}}^{-}[\mathrm{fC3bC3bBbHL}]+k_{\mathrm{C3bBbHL}_{\mathrm{decay}}}^{-}[\mathrm{fC3bC3bBbHL}]\;-k_{\mathrm{C3bHL}}^{+}[\mathrm{hC3bC4b}][\mathrm{HL}]+k_{\mathrm{C3bHL}}^{-}[\mathrm{hC3bC4bHL}]
$$

$$
+\left(\begin{array}{l}
\frac{k^{\mathrm{FI}}_{\mathrm{catC3bH}}[\mathrm{I}][\mathrm{hC3bC4bHL}]}{K^{\mathrm{FI}}_{\mathrm{mC3bH}}+[\mathrm{C3(H_2O)H}]+[\mathrm{C3(H_2O)HL}]+[\mathrm{fC3bH}]+[\mathrm{fC3bHL}]+[\mathrm{fC3bCR1}]+[\mathrm{fiC3bCR1}]+[\mathrm{IgGC3bC3bH}]+[\mathrm{IgGC3biC3bH}]+[\mathrm{IgGC3bC3bHL}]}\cdots \\
\frac{1}{+[\mathrm{IgGC3biC3bHL}]+[\mathrm{IgGC3bC3bCR1}]+[\mathrm{IgGC3biC3bCR1}]+[\mathrm{IgGiC3biC3bCR1}]+[\mathrm{IgGiC3bC3dgCR1}]+[\mathrm{fC3bC4bH}]+[\mathrm{fC3bC4bHL}]}\cdots \\
\frac{1}{+[\mathrm{fC3bC4bCR1}]+[\mathrm{fC3bC4bC4BP}]+[\mathrm{fC3bC4dH}]+[\mathrm{fC3bC4dHL}]+[\mathrm{fC3bC4dCR1}]+[\mathrm{fiC3bC4dCR1}]+[\mathrm{fiC3bC4bCR1}]+[\mathrm{fiC3bC4bC4BP}]}\cdots \\
\frac{1}{+[\mathrm{fC3dgC4bCR1}]+[\mathrm{fC3dgC4bC4BP}]+[\mathrm{fC3bC3bH}]+[\mathrm{fC3bC3bHL}]+[\mathrm{fC3bC3bCR1}]+[\mathrm{fC3biC3bH}]+[\mathrm{fC3biC3bHL}]+[\mathrm{fC3biC3bCR1}]}\cdots \\
\frac{1}{+[\mathrm{fiC3biC3bCR1}]+[\mathrm{fiC3bC3dgCR1}]+[\mathrm{hC3bH}]+[\mathrm{hC3bHL}]+[\mathrm{hC3bCR1}]+[\mathrm{hiC3bCR1}]+[\mathrm{hC3bC4bH}]+[\mathrm{hC3bC4bHL}]+[\mathrm{hC3bC4bCR1}]}\cdots \\
\frac{1}{+[\mathrm{hC3bC4bC4BP}]+[\mathrm{hC3bC4dH}]+[\mathrm{hC3bC4dHL}]+[\mathrm{hC3bC4dCR1}]+[\mathrm{hiC3bC4dCR1}]+[\mathrm{hiC3bC4bCR1}]+[\mathrm{hiC3bC4bC4BP}]+[\mathrm{hC3dgC4bCR1}]}\cdots \\
\frac{1}{+[\mathrm{hC3dgC4bC4BP}]+[\mathrm{hC3bC3bH}]+[\mathrm{hC3bC3bHL}]+[\mathrm{hC3bC3bCR1}]+[\mathrm{hC3biC3bH}]+[\mathrm{hC3biC3bHL}]+[\mathrm{hC3biC3bCR1}]+[\mathrm{hiC3biC3bCR1}]}\cdots \\
\frac{1}{+[\mathrm{hiC3bC3dgCR1}]+[\mathrm{fC4bCR1}]+[\mathrm{nfC4bC4BP}]+[\mathrm{fC4bC4BP}]+[\mathrm{fC4bC4bCR1}]+[\mathrm{fC4bC4bC4BP}]+[\mathrm{fC4bC4dCR1}]+[\mathrm{fC4bC4dC4BP}]+[\mathrm{hC4bCR1}]}\cdots \\
\frac{1}{+[\mathrm{hC4bC4BP}]+[\mathrm{hC4bC4bCR1}]+[\mathrm{hC4bC4bC4BP}]+[\mathrm{hC4bC4dCR1}]+[\mathrm{hC4bC4dC4BP}]}\cdots
\end{array}\right)
$$

$$
-k^{+}_{\mathrm{C3bHL}}[\mathrm{hC3bC4d}][\mathrm{HL}]+k^{-}_{\mathrm{C3bHL}}[\mathrm{hC3bC4dHL}]
$$

$$
+\left(\begin{array}{l}
\frac{k^{\mathrm{FI}}_{\mathrm{catC3bH}}[\mathrm{I}][\mathrm{hC3bC4dHL}]}{K^{\mathrm{FI}}_{\mathrm{mC3bH}}+[\mathrm{C3(H_2O)H}]+[\mathrm{C3(H_2O)HL}]+[\mathrm{fC3bH}]+[\mathrm{fC3bHL}]+[\mathrm{fC3bCR1}]+[\mathrm{fiC3bCR1}]+[\mathrm{IgGC3bC3bH}]+[\mathrm{IgGC3biC3bH}]+[\mathrm{IgGC3bC3bHL}]}\cdots \\
\frac{1}{+[\mathrm{IgGC3biC3bHL}]+[\mathrm{IgGC3bC3bCR1}]+[\mathrm{IgGC3biC3bCR1}]+[\mathrm{IgGiC3biC3bCR1}]+[\mathrm{IgGiC3bC3dgCR1}]+[\mathrm{fC3bC4bH}]+[\mathrm{fC3bC4bHL}]}\cdots \\
\frac{1}{+[\mathrm{fC3bC4bCR1}]+[\mathrm{fC3bC4bC4BP}]+[\mathrm{fC3bC4dH}]+[\mathrm{fC3bC4dHL}]+[\mathrm{fC3bC4dCR1}]+[\mathrm{fiC3bC4dCR1}]+[\mathrm{fiC3bC4bCR1}]+[\mathrm{fiC3bC4bC4BP}]}\cdots \\
\frac{1}{+[\mathrm{fC3dgC4bCR1}]+[\mathrm{fC3dgC4bC4BP}]+[\mathrm{fC3bC3bH}]+[\mathrm{fC3bC3bHL}]+[\mathrm{fC3bC3bCR1}]+[\mathrm{fC3biC3bH}]+[\mathrm{fC3biC3bHL}]+[\mathrm{fC3biC3bCR1}]}\cdots \\
\frac{1}{+[\mathrm{fiC3biC3bCR1}]+[\mathrm{fiC3bC3dgCR1}]+[\mathrm{hC3bH}]+[\mathrm{hC3bHL}]+[\mathrm{hC3bCR1}]+[\mathrm{hiC3bCR1}]+[\mathrm{hC3bC4bH}]+[\mathrm{hC3bC4bHL}]+[\mathrm{hC3bC4bCR1}]}\cdots \\
\frac{1}{+[\mathrm{hC3bC4bC4BP}]+[\mathrm{hC3bC4dH}]+[\mathrm{hC3bC4dHL}]+[\mathrm{hC3bC4dCR1}]+[\mathrm{hiC3bC4dCR1}]+[\mathrm{hiC3bC4bCR1}]+[\mathrm{hiC3bC4bC4BP}]+[\mathrm{hC3dgC4bCR1}]}\cdots \\
\frac{1}{+[\mathrm{hC3dgC4bC4BP}]+[\mathrm{hC3bC3bH}]+[\mathrm{hC3bC3bHL}]+[\mathrm{hC3bC3bCR1}]+[\mathrm{hC3biC3bH}]+[\mathrm{hC3biC3bHL}]+[\mathrm{hC3biC3bCR1}]+[\mathrm{hiC3biC3bCR1}]}\cdots \\
\frac{1}{+[\mathrm{hiC3bC3dgCR1}]+[\mathrm{fC4bCR1}]+[\mathrm{nfC4bC4BP}]+[\mathrm{fC4bC4BP}]+[\mathrm{fC4bC4bCR1}]+[\mathrm{fC4bC4bC4BP}]+[\mathrm{fC4bC4dCR1}]+[\mathrm{fC4bC4dC4BP}]+[\mathrm{hC4bCR1}]}\cdots \\
\frac{1}{+[\mathrm{hC4bC4BP}]+[\mathrm{hC4bC4bCR1}]+[\mathrm{hC4bC4bC4BP}]+[\mathrm{hC4bC4dCR1}]+[\mathrm{hC4bC4dC4BP}]}\cdots
\end{array}\right)
$$

$$
-k^{+}_{\mathrm{C3bHL}}[\mathrm{hC3bC4bBb}][\mathrm{HL}]+k^{-}_{\mathrm{C3bHL}}[\mathrm{hC3bC4bBbHL}]+k^{-}_{\mathrm{C3bBbHL}_{\mathrm{decay}}}[\mathrm{hC3bC4bBbHL}]-k^{+}_{\mathrm{C3bHL}}[\mathrm{hC3bC3b}][\mathrm{HL}]+k^{-}_{\mathrm{C3bHL}}[\mathrm{hC3bC3bHL}]
$$

$$+\left(\frac{k^{\mathrm{FI}}_{\mathrm{catC3bH}}[\mathrm{I}][\mathrm{hC3bC3bHL}]}{K^{\mathrm{FI}}_{\mathrm{mC3bH}}+[\mathrm{C3(H_2O)H}]+[\mathrm{C3(H_2O)HL}]+[\mathrm{fC3bH}]+[\mathrm{fC3bHL}]+[\mathrm{fC3bCR1}]+[\mathrm{fiC3bCR1}]+[\mathrm{IgGC3bC3bH}]+[\mathrm{IgGC3biC3bH}]+[\mathrm{IgGC3bC3bHL}]}\cdots\right.$$

$$\frac{1}{+[\mathrm{IgGC3biC3bHL}]+[\mathrm{IgGC3bC3bCR1}]+[\mathrm{IgGC3biC3bCR1}]+[\mathrm{IgGiC3biC3bCR1}]+[\mathrm{IgGiC3bC3dgCR1}]+[\mathrm{fC3bC4bH}]+[\mathrm{fC3bC4bHL}]}\cdots$$

$$\frac{1}{+[\mathrm{fC3bC4bCR1}]+[\mathrm{fC3bC4bC4BP}]+[\mathrm{fC3bC4dH}]+[\mathrm{fC3bC4dHL}]+[\mathrm{fC3bC4dCR1}]+[\mathrm{fiC3bC4dCR1}]+[\mathrm{fiC3bC4bCR1}]+[\mathrm{fiC3bC4bC4BP}]}\cdots$$

$$\frac{1}{+[\mathrm{fC3dgC4bCR1}]+[\mathrm{fC3dgC4bC4BP}]+[\mathrm{fC3bC3bH}]+[\mathrm{fC3bC3bHL}]+[\mathrm{fC3bC3bCR1}]+[\mathrm{fC3biC3bH}]+[\mathrm{fC3biC3bHL}]+[\mathrm{fC3biC3bCR1}]}\cdots$$

$$\frac{1}{+[\mathrm{fiC3biC3bCR1}]+[\mathrm{fiC3bC3dgCR1}]+[\mathrm{hC3bH}]+[\mathrm{hC3bHL}]+[\mathrm{hC3bCR1}]+[\mathrm{hiC3bCR1}]+[\mathrm{hC3bC4bH}]+[\mathrm{hC3bC4bHL}]+[\mathrm{hC3bC4bCR1}]}\cdots$$

$$\frac{1}{+[\mathrm{hC3bC4bC4BP}]+[\mathrm{hC3bC4dH}]+[\mathrm{hC3bC4dHL}]+[\mathrm{hC3bC4dCR1}]+[\mathrm{hiC3bC4dCR1}]+[\mathrm{hiC3bC4bCR1}]+[\mathrm{hiC3bC4bC4BP}]+[\mathrm{hC3dgC4bCR1}]}\cdots$$

$$\frac{1}{+[\mathrm{hC3dgC4bC4BP}]+[\mathrm{hC3bC3bH}]+[\mathrm{hC3bC3bHL}]+[\mathrm{hC3bC3bCR1}]+[\mathrm{hC3biC3bH}]+[\mathrm{hC3biC3bHL}]+[\mathrm{hC3biC3bCR1}]+[\mathrm{hiC3biC3bCR1}]}\cdots$$

$$\frac{1}{+[\mathrm{hiC3bC3dgCR1}]+[\mathrm{fC4bCR1}]+[\mathrm{nfC4bC4BP}]+[\mathrm{fC4bC4BP}]+[\mathrm{fC4bC4bCR1}]+[\mathrm{fC4bC4bC4BP}]+[\mathrm{fC4bC4dCR1}]+[\mathrm{fC4bC4dC4BP}]+[\mathrm{hC4bCR1}]}\cdots$$

$$\left.\frac{1}{+[\mathrm{hC4bC4BP}]+[\mathrm{hC4bC4bCR1}]+[\mathrm{hC4bC4bC4BP}]+[\mathrm{hC4bC4dCR1}]+[\mathrm{hC4bC4dC4BP}]}\cdots\right)$$

$$-k^{+}_{\mathrm{C3bHL}}[\mathrm{hC3biC3b}][\mathrm{HL}]+k^{-}_{\mathrm{C3bHL}}[\mathrm{hC3biC3bHL}]$$

$$+\left(\frac{k^{\mathrm{FI}}_{\mathrm{catC3bH}}[\mathrm{I}][\mathrm{hC3biC3bHL}]}{K^{\mathrm{FI}}_{\mathrm{mC3bH}}+[\mathrm{C3(H_2O)H}]+[\mathrm{C3(H_2O)HL}]+[\mathrm{fC3bH}]+[\mathrm{fC3bHL}]+[\mathrm{fC3bCR1}]+[\mathrm{fiC3bCR1}]+[\mathrm{IgGC3bC3bH}]+[\mathrm{IgGC3biC3bH}]+[\mathrm{IgGC3bC3bHL}]}\cdots\right.$$

$$\frac{1}{+[\mathrm{IgGC3biC3bHL}]+[\mathrm{IgGC3bC3bCR1}]+[\mathrm{IgGC3biC3bCR1}]+[\mathrm{IgGiC3biC3bCR1}]+[\mathrm{IgGiC3bC3dgCR1}]+[\mathrm{fC3bC4bH}]+[\mathrm{fC3bC4bHL}]}\cdots$$

$$\frac{1}{+[\mathrm{fC3bC4bCR1}]+[\mathrm{fC3bC4bC4BP}]+[\mathrm{fC3bC4dH}]+[\mathrm{fC3bC4dHL}]+[\mathrm{fC3bC4dCR1}]+[\mathrm{fiC3bC4dCR1}]+[\mathrm{fiC3bC4bCR1}]+[\mathrm{fiC3bC4bC4BP}]}\cdots$$

$$\frac{1}{+[\mathrm{fC3dgC4bCR1}]+[\mathrm{fC3dgC4bC4BP}]+[\mathrm{fC3bC3bH}]+[\mathrm{fC3bC3bHL}]+[\mathrm{fC3bC3bCR1}]+[\mathrm{fC3biC3bH}]+[\mathrm{fC3biC3bHL}]+[\mathrm{fC3biC3bCR1}]}\cdots$$

$$\frac{1}{+[\mathrm{fiC3biC3bCR1}]+[\mathrm{fiC3bC3dgCR1}]+[\mathrm{hC3bH}]+[\mathrm{hC3bHL}]+[\mathrm{hC3bCR1}]+[\mathrm{hiC3bCR1}]+[\mathrm{hC3bC4bH}]+[\mathrm{hC3bC4bHL}]+[\mathrm{hC3bC4bCR1}]}\cdots$$

$$\frac{1}{+[\mathrm{hC3bC4bC4BP}]+[\mathrm{hC3bC4dH}]+[\mathrm{hC3bC4dHL}]+[\mathrm{hC3bC4dCR1}]+[\mathrm{hiC3bC4dCR1}]+[\mathrm{hiC3bC4bCR1}]+[\mathrm{hiC3bC4bC4BP}]+[\mathrm{hC3dgC4bCR1}]}\cdots$$

$$\frac{1}{+[\mathrm{hC3dgC4bC4BP}]+[\mathrm{hC3bC3bH}]+[\mathrm{hC3bC3bHL}]+[\mathrm{hC3bC3bCR1}]+[\mathrm{hC3biC3bH}]+[\mathrm{hC3biC3bHL}]+[\mathrm{hC3biC3bCR1}]+[\mathrm{hiC3biC3bCR1}]}\cdots$$

$$\frac{1}{+[\mathrm{hiC3bC3dgCR1}]+[\mathrm{fC4bCR1}]+[\mathrm{nfC4bC4BP}]+[\mathrm{fC4bC4BP}]+[\mathrm{fC4bC4bCR1}]+[\mathrm{fC4bC4bC4BP}]+[\mathrm{fC4bC4dCR1}]+[\mathrm{fC4bC4dC4BP}]+[\mathrm{hC4bCR1}]}\cdots$$

$$\left.\frac{1}{+[\mathrm{hC4bC4BP}]+[\mathrm{hC4bC4bCR1}]+[\mathrm{hC4bC4bC4BP}]+[\mathrm{hC4bC4dCR1}]+[\mathrm{hC4bC4dC4BP}]}\cdots\right)$$

$$-k^{+}_{\mathrm{C3bHL}}[\mathrm{hC3bC3bBb}][\mathrm{HL}]+k^{-}_{\mathrm{C3bHL}}[\mathrm{hC3bC3bBbHL}]+k^{-}_{\mathrm{C3bBbHL}_{\mathrm{decay}}}[\mathrm{hC3bC3bBbHL}]-k^{+}_{\mathrm{C3bHL}}[\mathrm{hC3b}][\mathrm{HL}]+k^{-}_{\mathrm{C3bHL}}[\mathrm{hC3bHL}]$$

$$
+\left(\begin{array}{l}
\dfrac{k_{\mathrm{catC3bH}}^{\mathrm{FI}}[\mathrm{I}][\mathrm{hC3bHL}]}{K_{\mathrm{mC3bH}}^{\mathrm{FI}}+[\mathrm{C3(H_2O)H}]+[\mathrm{C3(H_2O)HL}]+[\mathrm{fC3bH}]+[\mathrm{fC3bHL}]+[\mathrm{fC3bCR1}]+[\mathrm{fiC3bCR1}]+[\mathrm{IgGC3bC3bH}]+[\mathrm{IgGC3biC3bH}]+[\mathrm{IgGC3bC3bHL}]}\cdots \\
\dfrac{1}{+[\mathrm{IgGC3biC3bHL}]+[\mathrm{IgGC3bC3bCR1}]+[\mathrm{IgGC3biC3bCR1}]+[\mathrm{IgGiC3biC3bCR1}]+[\mathrm{IgGiC3bC3dgCR1}]+[\mathrm{fC3bC4bH}]+[\mathrm{fC3bC4bHL}]}\cdots \\
\dfrac{1}{+[\mathrm{fC3bC4bCR1}]+[\mathrm{fC3bC4bC4BP}]+[\mathrm{fC3bC4dH}]+[\mathrm{fC3bC4dHL}]+[\mathrm{fC3bC4dCR1}]+[\mathrm{fiC3bC4dCR1}]+[\mathrm{fiC3bC4bCR1}]+[\mathrm{fiC3bC4bC4BP}]}\cdots \\
\dfrac{1}{+[\mathrm{fC3dgC4bCR1}]+[\mathrm{fC3dgC4bC4BP}]+[\mathrm{fC3bC3bH}]+[\mathrm{fC3bC3bHL}]+[\mathrm{fC3bC3bCR1}]+[\mathrm{fC3biC3bH}]+[\mathrm{fC3biC3bHL}]+[\mathrm{fC3biC3bCR1}]}\cdots \\
\dfrac{1}{+[\mathrm{fiC3biC3bCR1}]+[\mathrm{fiC3bC3dgCR1}]+[\mathrm{hC3bH}]+[\mathrm{hC3bHL}]+[\mathrm{hC3bCR1}]+[\mathrm{hiC3bCR1}]+[\mathrm{hC3bC4bH}]+[\mathrm{hC3bC4bHL}]+[\mathrm{hC3bC4bCR1}]}\cdots \\
\dfrac{1}{+[\mathrm{hC3bC4bC4BP}]+[\mathrm{hC3bC4dH}]+[\mathrm{hC3bC4dHL}]+[\mathrm{hC3bC4dCR1}]+[\mathrm{hiC3bC4dCR1}]+[\mathrm{hiC3bC4bCR1}]+[\mathrm{hiC3bC4bC4BP}]+[\mathrm{hC3dgC4bCR1}]}\cdots \\
\dfrac{1}{+[\mathrm{hC3dgC4bC4BP}]+[\mathrm{hC3bC3bH}]+[\mathrm{hC3bC3bHL}]+[\mathrm{hC3bC3bCR1}]+[\mathrm{hC3biC3bH}]+[\mathrm{hC3biC3bHL}]+[\mathrm{hC3biC3bCR1}]+[\mathrm{hiC3biC3bCR1}]}\cdots \\
\dfrac{1}{+[\mathrm{hiC3bC3dgCR1}]+[\mathrm{fC4bCR1}]+[\mathrm{nfC4bC4BP}]+[\mathrm{fC4bC4BP}]+[\mathrm{fC4bC4bCR1}]+[\mathrm{fC4bC4bC4BP}]+[\mathrm{fC4bC4dCR1}]+[\mathrm{fC4bC4dC4BP}]+[\mathrm{hC4bCR1}]}\cdots \\
\dfrac{1}{+[\mathrm{hC4bC4BP}]+[\mathrm{hC4bC4bCR1}]+[\mathrm{hC4bC4bC4BP}]+[\mathrm{hC4bC4dCR1}]+[\mathrm{hC4bC4dC4BP}]}\cdots
\end{array}\right)
$$

$$
-k_{\mathrm{C3bHL}}^{+}[\mathrm{hC3bBb}][\mathrm{HL}]+k_{\mathrm{C3bHL}}^{-}[\mathrm{hC3bBbHL}]+k_{\mathrm{C3bBbHL}_{\mathrm{decay}}}^{-}[\mathrm{hC3bBbHL}]
$$

S289

$$
\frac{d[\mathrm{CR1}]}{dt}=-k_{\mathrm{C3bCR1}}^{+}[\mathrm{fC3b}][\mathrm{CR1}]+k_{\mathrm{C3bCR1}}^{-}[\mathrm{fC3bCR1}]
$$

$$
+\left(\begin{array}{l}
\dfrac{k_{\mathrm{catC3bH}}^{\mathrm{FI}}[\mathrm{I}][\mathrm{fC3bCR1}]}{K_{\mathrm{mC3bH}}^{\mathrm{FI}}+[\mathrm{C3(H_2O)H}]+[\mathrm{C3(H_2O)HL}]+[\mathrm{fC3bH}]+[\mathrm{fC3bHL}]+[\mathrm{fC3bCR1}]+[\mathrm{fiC3bCR1}]+[\mathrm{IgGC3bC3bH}]+[\mathrm{IgGC3biC3bH}]+[\mathrm{IgGC3bC3bHL}]}\cdots \\
\dfrac{1}{+[\mathrm{IgGC3biC3bHL}]+[\mathrm{IgGC3bC3bCR1}]+[\mathrm{IgGC3biC3bCR1}]+[\mathrm{IgGiC3biC3bCR1}]+[\mathrm{IgGiC3bC3dgCR1}]+[\mathrm{fC3bC4bH}]+[\mathrm{fC3bC4bHL}]}\cdots \\
\dfrac{1}{+[\mathrm{fC3bC4bCR1}]+[\mathrm{fC3bC4bC4BP}]+[\mathrm{fC3bC4dH}]+[\mathrm{fC3bC4dHL}]+[\mathrm{fC3bC4dCR1}]+[\mathrm{fiC3bC4dCR1}]+[\mathrm{fiC3bC4bCR1}]+[\mathrm{fiC3bC4bC4BP}]}\cdots \\
\dfrac{1}{+[\mathrm{fC3dgC4bCR1}]+[\mathrm{fC3dgC4bC4BP}]+[\mathrm{fC3bC3bH}]+[\mathrm{fC3bC3bHL}]+[\mathrm{fC3bC3bCR1}]+[\mathrm{fC3biC3bH}]+[\mathrm{fC3biC3bHL}]+[\mathrm{fC3biC3bCR1}]}\cdots \\
\dfrac{1}{+[\mathrm{fiC3biC3bCR1}]+[\mathrm{fiC3bC3dgCR1}]+[\mathrm{hC3bH}]+[\mathrm{hC3bHL}]+[\mathrm{hC3bCR1}]+[\mathrm{hiC3bCR1}]+[\mathrm{hC3bC4bH}]+[\mathrm{hC3bC4bHL}]+[\mathrm{hC3bC4bCR1}]}\cdots \\
\dfrac{1}{+[\mathrm{hC3bC4bC4BP}]+[\mathrm{hC3bC4dH}]+[\mathrm{hC3bC4dHL}]+[\mathrm{hC3bC4dCR1}]+[\mathrm{hiC3bC4dCR1}]+[\mathrm{hiC3bC4bCR1}]+[\mathrm{hiC3bC4bC4BP}]+[\mathrm{hC3dgC4bCR1}]}\cdots \\
\dfrac{1}{+[\mathrm{hC3dgC4bC4BP}]+[\mathrm{hC3bC3bH}]+[\mathrm{hC3bC3bHL}]+[\mathrm{hC3bC3bCR1}]+[\mathrm{hC3biC3bH}]+[\mathrm{hC3biC3bHL}]+[\mathrm{hC3biC3bCR1}]+[\mathrm{hiC3biC3bCR1}]}\cdots \\
\dfrac{1}{+[\mathrm{hiC3bC3dgCR1}]+[\mathrm{fC4bCR1}]+[\mathrm{nfC4bC4BP}]+[\mathrm{fC4bC4BP}]+[\mathrm{fC4bC4bCR1}]+[\mathrm{fC4bC4bC4BP}]+[\mathrm{fC4bC4dCR1}]+[\mathrm{fC4bC4dC4BP}]+[\mathrm{hC4bCR1}]}\cdots \\
\dfrac{1}{+[\mathrm{hC4bC4BP}]+[\mathrm{hC4bC4bCR1}]+[\mathrm{hC4bC4bC4BP}]+[\mathrm{hC4bC4dCR1}]+[\mathrm{hC4bC4dC4BP}]}\cdots
\end{array}\right)
$$

$$
-k_{\mathrm{C3bCR1}}^{+}[\mathrm{fC3bBb}][\mathrm{CR1}]+k_{\mathrm{C3bCR1}}^{-}[\mathrm{fC3bBbCR1}]+k_{\mathrm{C3bBbCR1}_{\mathrm{decay}}}^{-}[\mathrm{fC3bBbCR1}]-k_{\mathrm{iC3bCR1}}^{+}[\mathrm{fiC3b}][\mathrm{CR1}]+k_{\mathrm{iC3bCR1}}^{-}[\mathrm{fiC3bCR1}]
$$

$$+\left(\frac{k^{\mathrm{FI}}_{\mathrm{catC3bH}}[\mathrm{I}][\mathrm{fiC3bCR1}]}{K^{\mathrm{FI}}_{\mathrm{mC3bH}}+[\mathrm{C3(H_2O)H}]+[\mathrm{C3(H_2O)HL}]+[\mathrm{fC3bH}]+[\mathrm{fC3bHL}]+[\mathrm{fC3bCR1}]+[\mathrm{fiC3bCR1}]+[\mathrm{IgGC3bC3bH}]+[\mathrm{IgGC3biC3bH}]+[\mathrm{IgGC3bC3bHL}]}\cdots\right.$$

$$\frac{1}{+[\mathrm{IgGC3biC3bHL}]+[\mathrm{IgGC3bC3bCR1}]+[\mathrm{IgGC3biC3bCR1}]+[\mathrm{IgGiC3biC3bCR1}]+[\mathrm{IgGiC3bC3dgCR1}]+[\mathrm{fC3bC4bH}]+[\mathrm{fC3bC4bHL}]}\cdots$$

$$\frac{1}{+[\mathrm{fC3bC4bCR1}]+[\mathrm{fC3bC4bC4BP}]+[\mathrm{fC3bC4dH}]+[\mathrm{fC3bC4dHL}]+[\mathrm{fC3bC4dCR1}]+[\mathrm{fiC3bC4dCR1}]+[\mathrm{fiC3bC4bCR1}]+[\mathrm{fiC3bC4bC4BP}]}\cdots$$

$$\frac{1}{+[\mathrm{fC3dgC4bCR1}]+[\mathrm{fC3dgC4bC4BP}]+[\mathrm{fC3bC3bH}]+[\mathrm{fC3bC3bHL}]+[\mathrm{fC3bC3bCR1}]+[\mathrm{fC3biC3bH}]+[\mathrm{fC3biC3bHL}]+[\mathrm{fC3biC3bCR1}]}\cdots$$

$$\frac{1}{+[\mathrm{fiC3biC3bCR1}]+[\mathrm{fiC3bC3dgCR1}]+[\mathrm{hC3bH}]+[\mathrm{hC3bHL}]+[\mathrm{hC3bCR1}]+[\mathrm{hiC3bCR1}]+[\mathrm{hC3bC4bH}]+[\mathrm{hC3bC4bHL}]+[\mathrm{hC3bC4bCR1}]}\cdots$$

$$\frac{1}{+[\mathrm{hC3bC4bC4BP}]+[\mathrm{hC3bC4dH}]+[\mathrm{hC3bC4dHL}]+[\mathrm{hC3bC4dCR1}]+[\mathrm{hiC3bC4dCR1}]+[\mathrm{hiC3bC4bCR1}]+[\mathrm{hiC3bC4bC4BP}]+[\mathrm{hC3dgC4bCR1}]}\cdots$$

$$\frac{1}{+[\mathrm{hC3dgC4bC4BP}]+[\mathrm{hC3bC3bH}]+[\mathrm{hC3bC3bHL}]+[\mathrm{hC3bC3bCR1}]+[\mathrm{hC3biC3bH}]+[\mathrm{hC3biC3bHL}]+[\mathrm{hC3biC3bCR1}]+[\mathrm{hiC3biC3bCR1}]}\cdots$$

$$\frac{1}{+[\mathrm{hiC3bC3dgCR1}]+[\mathrm{fC4bCR1}]+[\mathrm{nfC4bC4BP}]+[\mathrm{fC4bC4BP}]+[\mathrm{fC4bC4bCR1}]+[\mathrm{fC4bC4bC4BP}]+[\mathrm{fC4bC4dCR1}]+[\mathrm{fC4bC4dC4BP}]+[\mathrm{hC4bCR1}]}\cdots$$

$$\left.\frac{1}{+[\mathrm{hC4bC4BP}]+[\mathrm{hC4bC4bCR1}]+[\mathrm{hC4bC4bC4BP}]+[\mathrm{hC4bC4dCR1}]+[\mathrm{hC4bC4dC4BP}]}\cdots\right)$$

$$-k^{+}_{\mathrm{C3bC3bCR1}}[\mathrm{IgGC3bC3b}][\mathrm{CR1}]+k^{-}_{\mathrm{C3bC3bCR1}}[\mathrm{IgGC3bC3bCR1}]$$

$$+\left(\frac{k^{\mathrm{FI}}_{\mathrm{catC3bH}}[\mathrm{I}][\mathrm{IgGC3bC3bCR1}]}{K^{\mathrm{FI}}_{\mathrm{mC3bH}}+[\mathrm{C3(H_2O)H}]+[\mathrm{C3(H_2O)HL}]+[\mathrm{fC3bH}]+[\mathrm{fC3bHL}]+[\mathrm{fC3bCR1}]+[\mathrm{fiC3bCR1}]+[\mathrm{IgGC3bC3bH}]+[\mathrm{IgGC3biC3bH}]+[\mathrm{IgGC3bC3bHL}]}\cdots\right.$$

$$\frac{1}{+[\mathrm{IgGC3biC3bHL}]+[\mathrm{IgGC3bC3bCR1}]+[\mathrm{IgGC3biC3bCR1}]+[\mathrm{IgGiC3biC3bCR1}]+[\mathrm{IgGiC3bC3dgCR1}]+[\mathrm{fC3bC4bH}]+[\mathrm{fC3bC4bHL}]}\cdots$$

$$\frac{1}{+[\mathrm{fC3bC4bCR1}]+[\mathrm{fC3bC4bC4BP}]+[\mathrm{fC3bC4dH}]+[\mathrm{fC3bC4dHL}]+[\mathrm{fC3bC4dCR1}]+[\mathrm{fiC3bC4dCR1}]+[\mathrm{fiC3bC4bCR1}]+[\mathrm{fiC3bC4bC4BP}]}\cdots$$

$$\frac{1}{+[\mathrm{fC3dgC4bCR1}]+[\mathrm{fC3dgC4bC4BP}]+[\mathrm{fC3bC3bH}]+[\mathrm{fC3bC3bHL}]+[\mathrm{fC3bC3bCR1}]+[\mathrm{fC3biC3bH}]+[\mathrm{fC3biC3bHL}]+[\mathrm{fC3biC3bCR1}]}\cdots$$

$$\frac{1}{+[\mathrm{fiC3biC3bCR1}]+[\mathrm{fiC3bC3dgCR1}]+[\mathrm{hC3bH}]+[\mathrm{hC3bHL}]+[\mathrm{hC3bCR1}]+[\mathrm{hiC3bCR1}]+[\mathrm{hC3bC4bH}]+[\mathrm{hC3bC4bHL}]+[\mathrm{hC3bC4bCR1}]}\cdots$$

$$\frac{1}{+[\mathrm{hC3bC4bC4BP}]+[\mathrm{hC3bC4dH}]+[\mathrm{hC3bC4dHL}]+[\mathrm{hC3bC4dCR1}]+[\mathrm{hiC3bC4dCR1}]+[\mathrm{hiC3bC4bCR1}]+[\mathrm{hiC3bC4bC4BP}]+[\mathrm{hC3dgC4bCR1}]}\cdots$$

$$\frac{1}{+[\mathrm{hC3dgC4bC4BP}]+[\mathrm{hC3bC3bH}]+[\mathrm{hC3bC3bHL}]+[\mathrm{hC3bC3bCR1}]+[\mathrm{hC3biC3bH}]+[\mathrm{hC3biC3bHL}]+[\mathrm{hC3biC3bCR1}]+[\mathrm{hiC3biC3bCR1}]}\cdots$$

$$\frac{1}{+[\mathrm{hiC3bC3dgCR1}]+[\mathrm{fC4bCR1}]+[\mathrm{nfC4bC4BP}]+[\mathrm{fC4bC4BP}]+[\mathrm{fC4bC4bCR1}]+[\mathrm{fC4bC4bC4BP}]+[\mathrm{fC4bC4dCR1}]+[\mathrm{fC4bC4dC4BP}]+[\mathrm{hC4bCR1}]}\cdots$$

$$\left.\frac{1}{+[\mathrm{hC4bC4BP}]+[\mathrm{hC4bC4bCR1}]+[\mathrm{hC4bC4bC4BP}]+[\mathrm{hC4bC4dCR1}]+[\mathrm{hC4bC4dC4BP}]}\cdots\right)$$

$$-k^{+}_{\mathrm{C3biC3bCR1}}[\mathrm{IgGC3biC3b}][\mathrm{CR1}]+k^{-}_{\mathrm{C3biC3bCR1}}[\mathrm{IgGC3biC3bCR1}]$$

$$+\left(\frac{k_{\text{catC3bH}}^{\text{FI}}[\text{I}][\text{IgGC3biC3bCR1}]}{K_{\text{mC3bH}}^{\text{FI}}+[\text{C3(H}_2\text{O)H}]+[\text{C3(H}_2\text{O)HL}]+[\text{fC3bH}]+[\text{fC3bHL}]+[\text{fC3bCR1}]+[\text{fiC3bCR1}]+[\text{IgGC3bC3bH}]+[\text{IgGC3biC3bH}]+[\text{IgGC3bC3bHL}]}\cdots\right.$$

$$\frac{1}{+[\text{IgGC3biC3bHL}]+[\text{IgGC3bC3bCR1}]+[\text{IgGC3biC3bCR1}]+[\text{IgGiC3biC3bCR1}]+[\text{IgGiC3bC3dgCR1}]+[\text{fC3bC4bH}]+[\text{fC3bC4bHL}]}\cdots$$

$$\frac{1}{+[\text{fC3bC4bCR1}]+[\text{fC3bC4bC4BP}]+[\text{fC3bC4dH}]+[\text{fC3bC4dHL}]+[\text{fC3bC4dCR1}]+[\text{fiC3bC4dCR1}]+[\text{fiC3bC4bCR1}]+[\text{fiC3bC4bC4BP}]}\cdots$$

$$\frac{1}{+[\text{fC3dgC4bCR1}]+[\text{fC3dgC4bC4BP}]+[\text{fC3bC3bH}]+[\text{fC3bC3bHL}]+[\text{fC3bC3bCR1}]+[\text{fC3biC3bH}]+[\text{fC3biC3bHL}]+[\text{fC3biC3bCR1}]}\cdots$$

$$\frac{1}{+[\text{fiC3biC3bCR1}]+[\text{fiC3bC3dgCR1}]+[\text{hC3bH}]+[\text{hC3bHL}]+[\text{hC3bCR1}]+[\text{hiC3bCR1}]+[\text{hC3bC4bH}]+[\text{hC3bC4bHL}]+[\text{hC3bC4bCR1}]}\cdots$$

$$\frac{1}{+[\text{hC3bC4bC4BP}]+[\text{hC3bC4dH}]+[\text{hC3bC4dHL}]+[\text{hC3bC4dCR1}]+[\text{hiC3bC4dCR1}]+[\text{hiC3bC4bCR1}]+[\text{hiC3bC4bC4BP}]+[\text{hC3dgC4bCR1}]}\cdots$$

$$\frac{1}{+[\text{hC3dgC4bC4BP}]+[\text{hC3bC3bH}]+[\text{hC3bC3bHL}]+[\text{hC3bC3bCR1}]+[\text{hC3biC3bH}]+[\text{hC3biC3bHL}]+[\text{hC3biC3bCR1}]+[\text{hiC3biC3bCR1}]}\cdots$$

$$\frac{1}{+[\text{hiC3bC3dgCR1}]+[\text{fC4bCR1}]+[\text{nfC4bC4BP}]+[\text{fC4bC4BP}]+[\text{fC4bC4bCR1}]+[\text{fC4bC4bC4BP}]+[\text{fC4bC4dCR1}]+[\text{fC4bC4dC4BP}]+[\text{hC4bCR1}]}\cdots$$

$$\left.\frac{1}{+[\text{hC4bC4BP}]+[\text{hC4bC4bCR1}]+[\text{hC4bC4bC4BP}]+[\text{hC4bC4dCR1}]+[\text{hC4bC4dC4BP}]}\cdots\right)$$

$$-k_{\text{iC3biC3bCR1}}^{+}[\text{IgGiC3biC3b}][\text{CR1}]+k_{\text{iC3biC3bCR1}}^{-}[\text{IgGiC3biC3bCR1}]$$

$$+\left(\frac{k_{\text{catC3bH}}^{\text{FI}}[\text{I}][\text{IgGiC3biC3bCR1}]}{K_{\text{mC3bH}}^{\text{FI}}+[\text{C3(H}_2\text{O)H}]+[\text{C3(H}_2\text{O)HL}]+[\text{fC3bH}]+[\text{fC3bHL}]+[\text{fC3bCR1}]+[\text{fiC3bCR1}]+[\text{IgGC3bC3bH}]+[\text{IgGC3biC3bH}]+[\text{IgGC3bC3bHL}]}\cdots\right.$$

$$\frac{1}{+[\text{IgGC3biC3bHL}]+[\text{IgGC3bC3bCR1}]+[\text{IgGC3biC3bCR1}]+[\text{IgGiC3biC3bCR1}]+[\text{IgGiC3bC3dgCR1}]+[\text{fC3bC4bH}]+[\text{fC3bC4bHL}]}\cdots$$

$$\frac{1}{+[\text{fC3bC4bCR1}]+[\text{fC3bC4bC4BP}]+[\text{fC3bC4dH}]+[\text{fC3bC4dHL}]+[\text{fC3bC4dCR1}]+[\text{fiC3bC4dCR1}]+[\text{fiC3bC4bCR1}]+[\text{fiC3bC4bC4BP}]}\cdots$$

$$\frac{1}{+[\text{fC3dgC4bCR1}]+[\text{fC3dgC4bC4BP}]+[\text{fC3bC3bH}]+[\text{fC3bC3bHL}]+[\text{fC3bC3bCR1}]+[\text{fC3biC3bH}]+[\text{fC3biC3bHL}]+[\text{fC3biC3bCR1}]}\cdots$$

$$\frac{1}{+[\text{fiC3biC3bCR1}]+[\text{fiC3bC3dgCR1}]+[\text{hC3bH}]+[\text{hC3bHL}]+[\text{hC3bCR1}]+[\text{hiC3bCR1}]+[\text{hC3bC4bH}]+[\text{hC3bC4bHL}]+[\text{hC3bC4bCR1}]}\cdots$$

$$\frac{1}{+[\text{hC3bC4bC4BP}]+[\text{hC3bC4dH}]+[\text{hC3bC4dHL}]+[\text{hC3bC4dCR1}]+[\text{hiC3bC4dCR1}]+[\text{hiC3bC4bCR1}]+[\text{hiC3bC4bC4BP}]+[\text{hC3dgC4bCR1}]}\cdots$$

$$\frac{1}{+[\text{hC3dgC4bC4BP}]+[\text{hC3bC3bH}]+[\text{hC3bC3bHL}]+[\text{hC3bC3bCR1}]+[\text{hC3biC3bH}]+[\text{hC3biC3bHL}]+[\text{hC3biC3bCR1}]+[\text{hiC3biC3bCR1}]}\cdots$$

$$\frac{1}{+[\text{hiC3bC3dgCR1}]+[\text{fC4bCR1}]+[\text{nfC4bC4BP}]+[\text{fC4bC4BP}]+[\text{fC4bC4bCR1}]+[\text{fC4bC4bC4BP}]+[\text{fC4bC4dCR1}]+[\text{fC4bC4dC4BP}]+[\text{hC4bCR1}]}\cdots$$

$$\left.\frac{1}{+[\text{hC4bC4BP}]+[\text{hC4bC4bCR1}]+[\text{hC4bC4bC4BP}]+[\text{hC4bC4dCR1}]+[\text{hC4bC4dC4BP}]}\cdots\right)$$

$$-k_{\text{iC3bC3dgCR1}}^{+}[\text{IgGiC3bC3dg}][\text{CR1}]+k_{\text{iC3bC3dgCR1}}^{-}[\text{IgGiC3bC3dgCR1}]$$

$$
+\left(\begin{array}{l}
\frac{k^{\mathrm{FI}}_{\mathrm{catC3bH}}[\mathrm{I}][\mathrm{IgGiC3bC3dgCR1}]}{K^{\mathrm{FI}}_{\mathrm{mC3bH}}+[\mathrm{C3(H_2O)H}]+[\mathrm{C3(H_2O)HL}]+[\mathrm{fC3bH}]+[\mathrm{fC3bHL}]+[\mathrm{fC3bCR1}]+[\mathrm{fiC3bCR1}]+[\mathrm{IgGC3bC3bH}]+[\mathrm{IgGC3biC3bH}]+[\mathrm{IgGC3bC3bHL}]}\cdots \\
\frac{1}{+[\mathrm{IgGC3biC3bHL}]+[\mathrm{IgGC3bC3bCR1}]+[\mathrm{IgGC3biC3bCR1}]+[\mathrm{IgGiC3biC3bCR1}]+[\mathrm{IgGiC3bC3dgCR1}]+[\mathrm{fC3bC4bH}]+[\mathrm{fC3bC4bHL}]}\cdots \\
\frac{1}{+[\mathrm{fC3bC4bCR1}]+[\mathrm{fC3bC4bC4BP}]+[\mathrm{fC3bC4dH}]+[\mathrm{fC3bC4dHL}]+[\mathrm{fC3bC4dCR1}]+[\mathrm{fiC3bC4dCR1}]+[\mathrm{fiC3bC4bCR1}]+[\mathrm{fiC3bC4bC4BP}]}\cdots \\
\frac{1}{+[\mathrm{fC3dgC4bCR1}]+[\mathrm{fC3dgC4bC4BP}]+[\mathrm{fC3bC3bH}]+[\mathrm{fC3bC3bHL}]+[\mathrm{fC3bC3bCR1}]+[\mathrm{fC3biC3bH}]+[\mathrm{fC3biC3bHL}]+[\mathrm{fC3biC3bCR1}]}\cdots \\
\frac{1}{+[\mathrm{fiC3biC3bCR1}]+[\mathrm{fiC3bC3dgCR1}]+[\mathrm{hC3bH}]+[\mathrm{hC3bHL}]+[\mathrm{hC3bCR1}]+[\mathrm{hiC3bCR1}]+[\mathrm{hC3bC4bH}]+[\mathrm{hC3bC4bHL}]+[\mathrm{hC3bC4bCR1}]}\cdots \\
\frac{1}{+[\mathrm{hC3bC4bC4BP}]+[\mathrm{hC3bC4dH}]+[\mathrm{hC3bC4dHL}]+[\mathrm{hC3bC4dCR1}]+[\mathrm{hiC3bC4dCR1}]+[\mathrm{hiC3bC4bCR1}]+[\mathrm{hiC3bC4bC4BP}]+[\mathrm{hC3dgC4bCR1}]}\cdots \\
\frac{1}{+[\mathrm{hC3dgC4bC4BP}]+[\mathrm{hC3bC3bH}]+[\mathrm{hC3bC3bHL}]+[\mathrm{hC3bC3bCR1}]+[\mathrm{hC3biC3bH}]+[\mathrm{hC3biC3bHL}]+[\mathrm{hC3biC3bCR1}]+[\mathrm{hiC3biC3bCR1}]}\cdots \\
\frac{1}{+[\mathrm{hiC3bC3dgCR1}]+[\mathrm{fC4bCR1}]+[\mathrm{nfC4bC4BP}]+[\mathrm{fC4bC4BP}]+[\mathrm{fC4bC4bCR1}]+[\mathrm{fC4bC4bC4BP}]+[\mathrm{fC4bC4dCR1}]+[\mathrm{fC4bC4dC4BP}]+[\mathrm{hC4bCR1}]}\cdots \\
\frac{1}{+[\mathrm{hC4bC4BP}]+[\mathrm{hC4bC4bCR1}]+[\mathrm{hC4bC4bC4BP}]+[\mathrm{hC4bC4dCR1}]+[\mathrm{hC4bC4dC4BP}]}\cdots
\end{array}\right)
$$

$$
-k^{+}_{\mathrm{C3bC3bCR1}}[\mathrm{IgGC3bC3bBb}][\mathrm{CR1}]+k^{-}_{\mathrm{C3bC3bCR1}}[\mathrm{IgGC3bC3bBbCR1}]+k^{-}_{\mathrm{C3bBbCR1}_{\mathrm{decay}}}[\mathrm{IgGC3bC3bBbCR1}]-k^{+}_{\mathrm{C3bC4bCR1}}[\mathrm{fC3bC4b}][\mathrm{CR1}]
$$

$$
+k^{-}_{\mathrm{C3bC4bCR1}}[\mathrm{fC3bC4bCR1}]
$$

$$
+\left(\begin{array}{l}
\frac{k^{\mathrm{FI}}_{\mathrm{catC3bH}}[\mathrm{I}][\mathrm{fC3bC4bCR1}]}{K^{\mathrm{FI}}_{\mathrm{mC3bH}}+[\mathrm{C3(H_2O)H}]+[\mathrm{C3(H_2O)HL}]+[\mathrm{fC3bH}]+[\mathrm{fC3bHL}]+[\mathrm{fC3bCR1}]+[\mathrm{fiC3bCR1}]+[\mathrm{IgGC3bC3bH}]+[\mathrm{IgGC3biC3bH}]+[\mathrm{IgGC3bC3bHL}]}\cdots \\
\frac{1}{+[\mathrm{IgGC3biC3bHL}]+[\mathrm{IgGC3bC3bCR1}]+[\mathrm{IgGC3biC3bCR1}]+[\mathrm{IgGiC3biC3bCR1}]+[\mathrm{IgGiC3bC3dgCR1}]+[\mathrm{fC3bC4bH}]+[\mathrm{fC3bC4bHL}]}\cdots \\
\frac{1}{+[\mathrm{fC3bC4bCR1}]+[\mathrm{fC3bC4bC4BP}]+[\mathrm{fC3bC4dH}]+[\mathrm{fC3bC4dHL}]+[\mathrm{fC3bC4dCR1}]+[\mathrm{fiC3bC4dCR1}]+[\mathrm{fiC3bC4bCR1}]+[\mathrm{fiC3bC4bC4BP}]}\cdots \\
\frac{1}{+[\mathrm{fC3dgC4bCR1}]+[\mathrm{fC3dgC4bC4BP}]+[\mathrm{fC3bC3bH}]+[\mathrm{fC3bC3bHL}]+[\mathrm{fC3bC3bCR1}]+[\mathrm{fC3biC3bH}]+[\mathrm{fC3biC3bHL}]+[\mathrm{fC3biC3bCR1}]}\cdots \\
\frac{1}{+[\mathrm{fiC3biC3bCR1}]+[\mathrm{fiC3bC3dgCR1}]+[\mathrm{hC3bH}]+[\mathrm{hC3bHL}]+[\mathrm{hC3bCR1}]+[\mathrm{hiC3bCR1}]+[\mathrm{hC3bC4bH}]+[\mathrm{hC3bC4bHL}]+[\mathrm{hC3bC4bCR1}]}\cdots \\
\frac{1}{+[\mathrm{hC3bC4bC4BP}]+[\mathrm{hC3bC4dH}]+[\mathrm{hC3bC4dHL}]+[\mathrm{hC3bC4dCR1}]+[\mathrm{hiC3bC4dCR1}]+[\mathrm{hiC3bC4bCR1}]+[\mathrm{hiC3bC4bC4BP}]+[\mathrm{hC3dgC4bCR1}]}\cdots \\
\frac{1}{+[\mathrm{hC3dgC4bC4BP}]+[\mathrm{hC3bC3bH}]+[\mathrm{hC3bC3bHL}]+[\mathrm{hC3bC3bCR1}]+[\mathrm{hC3biC3bH}]+[\mathrm{hC3biC3bHL}]+[\mathrm{hC3biC3bCR1}]+[\mathrm{hiC3biC3bCR1}]}\cdots \\
\frac{1}{+[\mathrm{hiC3bC3dgCR1}]+[\mathrm{fC4bCR1}]+[\mathrm{nfC4bC4BP}]+[\mathrm{fC4bC4BP}]+[\mathrm{fC4bC4bCR1}]+[\mathrm{fC4bC4bC4BP}]+[\mathrm{fC4bC4dCR1}]+[\mathrm{fC4bC4dC4BP}]+[\mathrm{hC4bCR1}]}\cdots \\
\frac{1}{+[\mathrm{hC4bC4BP}]+[\mathrm{hC4bC4bCR1}]+[\mathrm{hC4bC4bC4BP}]+[\mathrm{hC4bC4dCR1}]+[\mathrm{hC4bC4dC4BP}]}\cdots
\end{array}\right)
$$

$$
-k^{+}_{\mathrm{C3bC4dCR1}}[\mathrm{fC3bC4d}][\mathrm{CR1}]+k^{-}_{\mathrm{C3bC4dCR1}}[\mathrm{fC3bC4dCR1}]
$$

$$+\left(\frac{k_{\mathrm{catC3bH}}^{\mathrm{FI}}[\mathrm{I}][\mathrm{fC3bC4dCR1}]}{K_{\mathrm{mC3bH}}^{\mathrm{FI}}+[\mathrm{C3(H_2O)H}]+[\mathrm{C3(H_2O)HL}]+[\mathrm{fC3bH}]+[\mathrm{fC3bHL}]+[\mathrm{fC3bCR1}]+[\mathrm{fiC3bCR1}]+[\mathrm{IgGC3bC3bH}]+[\mathrm{IgGC3biC3bH}]+[\mathrm{IgGC3bC3bHL}]}\cdots\right.$$

$$\frac{1}{+[\mathrm{IgGC3biC3bHL}]+[\mathrm{IgGC3bC3bCR1}]+[\mathrm{IgGC3biC3bCR1}]+[\mathrm{IgGiC3biC3bCR1}]+[\mathrm{IgGiC3bC3dgCR1}]+[\mathrm{fC3bC4bH}]+[\mathrm{fC3bC4bHL}]}\cdots$$

$$\frac{1}{+[\mathrm{fC3bC4bCR1}]+[\mathrm{fC3bC4bC4BP}]+[\mathrm{fC3bC4dH}]+[\mathrm{fC3bC4dHL}]+[\mathrm{fC3bC4dCR1}]+[\mathrm{fiC3bC4dCR1}]+[\mathrm{fiC3bC4bCR1}]+[\mathrm{fiC3bC4bC4BP}]}\cdots$$

$$\frac{1}{+[\mathrm{fC3dgC4bCR1}]+[\mathrm{fC3dgC4bC4BP}]+[\mathrm{fC3bC3bH}]+[\mathrm{fC3bC3bHL}]+[\mathrm{fC3bC3bCR1}]+[\mathrm{fC3biC3bH}]+[\mathrm{fC3biC3bHL}]+[\mathrm{fC3biC3bCR1}]}\cdots$$

$$\frac{1}{+[\mathrm{fiC3biC3bCR1}]+[\mathrm{fiC3bC3dgCR1}]+[\mathrm{hC3bH}]+[\mathrm{hC3bHL}]+[\mathrm{hC3bCR1}]+[\mathrm{hiC3bCR1}]+[\mathrm{hC3bC4bH}]+[\mathrm{hC3bC4bHL}]+[\mathrm{hC3bC4bCR1}]}\cdots$$

$$\frac{1}{+[\mathrm{hC3bC4bC4BP}]+[\mathrm{hC3bC4dH}]+[\mathrm{hC3bC4dHL}]+[\mathrm{hC3bC4dCR1}]+[\mathrm{hiC3bC4dCR1}]+[\mathrm{hiC3bC4bCR1}]+[\mathrm{hiC3bC4bC4BP}]+[\mathrm{hC3dgC4bCR1}]}\cdots$$

$$\frac{1}{+[\mathrm{hC3dgC4bC4BP}]+[\mathrm{hC3bC3bH}]+[\mathrm{hC3bC3bHL}]+[\mathrm{hC3bC3bCR1}]+[\mathrm{hC3biC3bH}]+[\mathrm{hC3biC3bHL}]+[\mathrm{hC3biC3bCR1}]+[\mathrm{hiC3biC3bCR1}]}\cdots$$

$$\frac{1}{+[\mathrm{hiC3bC3dgCR1}]+[\mathrm{fC4bCR1}]+[\mathrm{nfC4bC4BP}]+[\mathrm{fC4bC4BP}]+[\mathrm{fC4bC4bCR1}]+[\mathrm{fC4bC4bC4BP}]+[\mathrm{fC4bC4dCR1}]+[\mathrm{fC4bC4dC4BP}]+[\mathrm{hC4bCR1}]}\cdots$$

$$\left.\frac{1}{+[\mathrm{hC4bC4BP}]+[\mathrm{hC4bC4bCR1}]+[\mathrm{hC4bC4bC4BP}]+[\mathrm{hC4bC4dCR1}]+[\mathrm{hC4bC4dC4BP}]}\cdots\right)$$

$$-k_{\mathrm{iC3bC4bCR1}}^{+}[\mathrm{fiC3bC4b}][\mathrm{CR1}]+k_{\mathrm{iC3bC4bCR1}}^{-}[\mathrm{fiC3bC4bCR1}]$$

$$+\left(\frac{k_{\mathrm{catC3bH}}^{\mathrm{FI}}[\mathrm{I}][\mathrm{fiC3bC4bCR1}]}{K_{\mathrm{mC3bH}}^{\mathrm{FI}}+[\mathrm{C3(H_2O)H}]+[\mathrm{C3(H_2O)HL}]+[\mathrm{fC3bH}]+[\mathrm{fC3bHL}]+[\mathrm{fC3bCR1}]+[\mathrm{fiC3bCR1}]+[\mathrm{IgGC3bC3bH}]+[\mathrm{IgGC3biC3bH}]+[\mathrm{IgGC3bC3bHL}]}\cdots\right.$$

$$\frac{1}{+[\mathrm{IgGC3biC3bHL}]+[\mathrm{IgGC3bC3bCR1}]+[\mathrm{IgGC3biC3bCR1}]+[\mathrm{IgGiC3biC3bCR1}]+[\mathrm{IgGiC3bC3dgCR1}]+[\mathrm{fC3bC4bH}]+[\mathrm{fC3bC4bHL}]}\cdots$$

$$\frac{1}{+[\mathrm{fC3bC4bCR1}]+[\mathrm{fC3bC4bC4BP}]+[\mathrm{fC3bC4dH}]+[\mathrm{fC3bC4dHL}]+[\mathrm{fC3bC4dCR1}]+[\mathrm{fiC3bC4dCR1}]+[\mathrm{fiC3bC4bCR1}]+[\mathrm{fiC3bC4bC4BP}]}\cdots$$

$$\frac{1}{+[\mathrm{fC3dgC4bCR1}]+[\mathrm{fC3dgC4bC4BP}]+[\mathrm{fC3bC3bH}]+[\mathrm{fC3bC3bHL}]+[\mathrm{fC3bC3bCR1}]+[\mathrm{fC3biC3bH}]+[\mathrm{fC3biC3bHL}]+[\mathrm{fC3biC3bCR1}]}\cdots$$

$$\frac{1}{+[\mathrm{fiC3biC3bCR1}]+[\mathrm{fiC3bC3dgCR1}]+[\mathrm{hC3bH}]+[\mathrm{hC3bHL}]+[\mathrm{hC3bCR1}]+[\mathrm{hiC3bCR1}]+[\mathrm{hC3bC4bH}]+[\mathrm{hC3bC4bHL}]+[\mathrm{hC3bC4bCR1}]}\cdots$$

$$\frac{1}{+[\mathrm{hC3bC4bC4BP}]+[\mathrm{hC3bC4dH}]+[\mathrm{hC3bC4dHL}]+[\mathrm{hC3bC4dCR1}]+[\mathrm{hiC3bC4dCR1}]+[\mathrm{hiC3bC4bCR1}]+[\mathrm{hiC3bC4bC4BP}]+[\mathrm{hC3dgC4bCR1}]}\cdots$$

$$\frac{1}{+[\mathrm{hC3dgC4bC4BP}]+[\mathrm{hC3bC3bH}]+[\mathrm{hC3bC3bHL}]+[\mathrm{hC3bC3bCR1}]+[\mathrm{hC3biC3bH}]+[\mathrm{hC3biC3bHL}]+[\mathrm{hC3biC3bCR1}]+[\mathrm{hiC3biC3bCR1}]}\cdots$$

$$\frac{1}{+[\mathrm{hiC3bC3dgCR1}]+[\mathrm{fC4bCR1}]+[\mathrm{nfC4bC4BP}]+[\mathrm{fC4bC4BP}]+[\mathrm{fC4bC4bCR1}]+[\mathrm{fC4bC4bC4BP}]+[\mathrm{fC4bC4dCR1}]+[\mathrm{fC4bC4dC4BP}]+[\mathrm{hC4bCR1}]}\cdots$$

$$\left.\frac{1}{+[\mathrm{hC4bC4BP}]+[\mathrm{hC4bC4bCR1}]+[\mathrm{hC4bC4bC4BP}]+[\mathrm{hC4bC4dCR1}]+[\mathrm{hC4bC4dC4BP}]}\cdots\right)$$

$$-k_{\mathrm{C3dgC4bCR1}}^{+}[\mathrm{fC3dgC4b}][\mathrm{CR1}]+k_{\mathrm{C3dgC4bCR1}}^{-}[\mathrm{fC3dgC4bCR1}]$$

$$
+\left(\begin{array}{l}
\dfrac{k^{\mathrm{FI}}_{\mathrm{catC3bH}}[\mathrm{I}][\mathrm{fC3dgC4bCR1}]}{K^{\mathrm{FI}}_{\mathrm{mC3bH}}+[\mathrm{C3(H_2O)H}]+[\mathrm{C3(H_2O)HL}]+[\mathrm{fC3bH}]+[\mathrm{fC3bHL}]+[\mathrm{fC3bCR1}]+[\mathrm{fiC3bCR1}]+[\mathrm{IgGC3bC3bH}]+[\mathrm{IgGC3biC3bH}]+[\mathrm{IgGC3bC3bHL}]}\cdots \\
\dfrac{1}{+[\mathrm{IgGC3biC3bHL}]+[\mathrm{IgGC3bC3bCR1}]+[\mathrm{IgGC3biC3bCR1}]+[\mathrm{IgGiC3biC3bCR1}]+[\mathrm{IgGiC3bC3dgCR1}]+[\mathrm{fC3bC4bH}]+[\mathrm{fC3bC4bHL}]}\cdots \\
\dfrac{1}{+[\mathrm{fC3bC4bCR1}]+[\mathrm{fC3bC4bC4BP}]+[\mathrm{fC3bC4dH}]+[\mathrm{fC3bC4dHL}]+[\mathrm{fC3bC4dCR1}]+[\mathrm{fiC3bC4dCR1}]+[\mathrm{fiC3bC4bCR1}]+[\mathrm{fiC3bC4bC4BP}]}\cdots \\
\dfrac{1}{+[\mathrm{fC3dgC4bCR1}]+[\mathrm{fC3dgC4bC4BP}]+[\mathrm{fC3bC3bH}]+[\mathrm{fC3bC3bHL}]+[\mathrm{fC3bC3bCR1}]+[\mathrm{fC3biC3bH}]+[\mathrm{fC3biC3bHL}]+[\mathrm{fC3biC3bCR1}]}\cdots \\
\dfrac{1}{+[\mathrm{fiC3biC3bCR1}]+[\mathrm{fiC3bC3dgCR1}]+[\mathrm{hC3bH}]+[\mathrm{hC3bHL}]+[\mathrm{hC3bCR1}]+[\mathrm{hiC3bCR1}]+[\mathrm{hC3bC4bH}]+[\mathrm{hC3bC4bHL}]+[\mathrm{hC3bC4bCR1}]}\cdots \\
\dfrac{1}{+[\mathrm{hC3bC4bC4BP}]+[\mathrm{hC3bC4dH}]+[\mathrm{hC3bC4dHL}]+[\mathrm{hC3bC4dCR1}]+[\mathrm{hiC3bC4dCR1}]+[\mathrm{hiC3bC4bCR1}]+[\mathrm{hiC3bC4bC4BP}]+[\mathrm{hC3dgC4bCR1}]}\cdots \\
\dfrac{1}{+[\mathrm{hC3dgC4bC4BP}]+[\mathrm{hC3bC3bH}]+[\mathrm{hC3bC3bHL}]+[\mathrm{hC3bC3bCR1}]+[\mathrm{hC3biC3bH}]+[\mathrm{hC3biC3bHL}]+[\mathrm{hC3biC3bCR1}]+[\mathrm{hiC3biC3bCR1}]}\cdots \\
\dfrac{1}{+[\mathrm{hiC3bC3dgCR1}]+[\mathrm{fC4bCR1}]+[\mathrm{nfC4bC4BP}]+[\mathrm{fC4bC4BP}]+[\mathrm{fC4bC4bCR1}]+[\mathrm{fC4bC4bC4BP}]+[\mathrm{fC4bC4dCR1}]+[\mathrm{fC4bC4dC4BP}]+[\mathrm{hC4bCR1}]}\cdots \\
\dfrac{1}{+[\mathrm{hC4bC4BP}]+[\mathrm{hC4bC4bCR1}]+[\mathrm{hC4bC4bC4BP}]+[\mathrm{hC4bC4dCR1}]+[\mathrm{hC4bC4dC4BP}]}\cdots
\end{array}\right)
$$

$$
-k^{+}_{\mathrm{iC3bC4dCR1}}[\mathrm{fiC3bC4d}][\mathrm{CR1}]+k^{-}_{\mathrm{iC3bC4dCR1}}[\mathrm{fiC3bC4dCR1}]
$$

$$
+\left(\begin{array}{l}
\dfrac{k^{\mathrm{FI}}_{\mathrm{catC3bH}}[\mathrm{I}][\mathrm{fiC3bC4dCR1}]}{K^{\mathrm{FI}}_{\mathrm{mC3bH}}+[\mathrm{C3(H_2O)H}]+[\mathrm{C3(H_2O)HL}]+[\mathrm{fC3bH}]+[\mathrm{fC3bHL}]+[\mathrm{fC3bCR1}]+[\mathrm{fiC3bCR1}]+[\mathrm{IgGC3bC3bH}]+[\mathrm{IgGC3biC3bH}]+[\mathrm{IgGC3bC3bHL}]}\cdots \\
\dfrac{1}{+[\mathrm{IgGC3biC3bHL}]+[\mathrm{IgGC3bC3bCR1}]+[\mathrm{IgGC3biC3bCR1}]+[\mathrm{IgGiC3biC3bCR1}]+[\mathrm{IgGiC3bC3dgCR1}]+[\mathrm{fC3bC4bH}]+[\mathrm{fC3bC4bHL}]}\cdots \\
\dfrac{1}{+[\mathrm{fC3bC4bCR1}]+[\mathrm{fC3bC4bC4BP}]+[\mathrm{fC3bC4dH}]+[\mathrm{fC3bC4dHL}]+[\mathrm{fC3bC4dCR1}]+[\mathrm{fiC3bC4dCR1}]+[\mathrm{fiC3bC4bCR1}]+[\mathrm{fiC3bC4bC4BP}]}\cdots \\
\dfrac{1}{+[\mathrm{fC3dgC4bCR1}]+[\mathrm{fC3dgC4bC4BP}]+[\mathrm{fC3bC3bH}]+[\mathrm{fC3bC3bHL}]+[\mathrm{fC3bC3bCR1}]+[\mathrm{fC3biC3bH}]+[\mathrm{fC3biC3bHL}]+[\mathrm{fC3biC3bCR1}]}\cdots \\
\dfrac{1}{+[\mathrm{fiC3biC3bCR1}]+[\mathrm{fiC3bC3dgCR1}]+[\mathrm{hC3bH}]+[\mathrm{hC3bHL}]+[\mathrm{hC3bCR1}]+[\mathrm{hiC3bCR1}]+[\mathrm{hC3bC4bH}]+[\mathrm{hC3bC4bHL}]+[\mathrm{hC3bC4bCR1}]}\cdots \\
\dfrac{1}{+[\mathrm{hC3bC4bC4BP}]+[\mathrm{hC3bC4dH}]+[\mathrm{hC3bC4dHL}]+[\mathrm{hC3bC4dCR1}]+[\mathrm{hiC3bC4dCR1}]+[\mathrm{hiC3bC4bCR1}]+[\mathrm{hiC3bC4bC4BP}]+[\mathrm{hC3dgC4bCR1}]}\cdots \\
\dfrac{1}{+[\mathrm{hC3dgC4bC4BP}]+[\mathrm{hC3bC3bH}]+[\mathrm{hC3bC3bHL}]+[\mathrm{hC3bC3bCR1}]+[\mathrm{hC3biC3bH}]+[\mathrm{hC3biC3bHL}]+[\mathrm{hC3biC3bCR1}]+[\mathrm{hiC3biC3bCR1}]}\cdots \\
\dfrac{1}{+[\mathrm{hiC3bC3dgCR1}]+[\mathrm{fC4bCR1}]+[\mathrm{nfC4bC4BP}]+[\mathrm{fC4bC4BP}]+[\mathrm{fC4bC4bCR1}]+[\mathrm{fC4bC4bC4BP}]+[\mathrm{fC4bC4dCR1}]+[\mathrm{fC4bC4dC4BP}]+[\mathrm{hC4bCR1}]}\cdots \\
\dfrac{1}{+[\mathrm{hC4bC4BP}]+[\mathrm{hC4bC4bCR1}]+[\mathrm{hC4bC4bC4BP}]+[\mathrm{hC4bC4dCR1}]+[\mathrm{hC4bC4dC4BP}]}\cdots
\end{array}\right)
$$

$$
-k^{+}_{\mathrm{C3bC4bCR1}}[\mathrm{fC3bC4bBb}][\mathrm{CR1}]+k^{-}_{\mathrm{C3bC4bCR1}}[\mathrm{fC3bC4bBbCR1}]+k^{-}_{\mathrm{C3bBbCR1}_{\mathrm{decay}}}[\mathrm{fC3bC4bBbCR1}]-k^{+}_{\mathrm{C3bC3bCR1}}[\mathrm{fC3bC3b}][\mathrm{CR1}]
$$

$$
+k^{-}_{\mathrm{C3bC3bCR1}}[\mathrm{fC3bC3bCR1}]
$$

$$
+\left(\begin{array}{l}
\dfrac{k^{\mathrm{FI}}_{\mathrm{catC3bH}}[\mathrm{I}][\mathrm{fC3bC3bCR1}]}{K^{\mathrm{FI}}_{\mathrm{mC3bH}}+[\mathrm{C3(H_2O)H}]+[\mathrm{C3(H_2O)HL}]+[\mathrm{fC3bH}]+[\mathrm{fC3bHL}]+[\mathrm{fC3bCR1}]+[\mathrm{fiC3bCR1}]+[\mathrm{IgGC3bC3bH}]+[\mathrm{IgGC3biC3bH}]+[\mathrm{IgGC3bC3bHL}]}\cdots \\
\dfrac{1}{+[\mathrm{IgGC3biC3bHL}]+[\mathrm{IgGC3bC3bCR1}]+[\mathrm{IgGC3biC3bCR1}]+[\mathrm{IgGiC3biC3bCR1}]+[\mathrm{IgGiC3bC3dgCR1}]+[\mathrm{fC3bC4bH}]+[\mathrm{fC3bC4bHL}]}\cdots \\
\dfrac{1}{+[\mathrm{fC3bC4bCR1}]+[\mathrm{fC3bC4bC4BP}]+[\mathrm{fC3bC4dH}]+[\mathrm{fC3bC4dHL}]+[\mathrm{fC3bC4dCR1}]+[\mathrm{fiC3bC4dCR1}]+[\mathrm{fiC3bC4bCR1}]+[\mathrm{fiC3bC4bC4BP}]}\cdots \\
\dfrac{1}{+[\mathrm{fC3dgC4bCR1}]+[\mathrm{fC3dgC4bC4BP}]+[\mathrm{fC3bC3bH}]+[\mathrm{fC3bC3bHL}]+[\mathrm{fC3bC3bCR1}]+[\mathrm{fC3biC3bH}]+[\mathrm{fC3biC3bHL}]+[\mathrm{fC3biC3bCR1}]}\cdots \\
\dfrac{1}{+[\mathrm{fiC3biC3bCR1}]+[\mathrm{fiC3bC3dgCR1}]+[\mathrm{hC3bH}]+[\mathrm{hC3bHL}]+[\mathrm{hC3bCR1}]+[\mathrm{hiC3bCR1}]+[\mathrm{hC3bC4bH}]+[\mathrm{hC3bC4bHL}]+[\mathrm{hC3bC4bCR1}]}\cdots \\
\dfrac{1}{+[\mathrm{hC3bC4bC4BP}]+[\mathrm{hC3bC4dH}]+[\mathrm{hC3bC4dHL}]+[\mathrm{hC3bC4dCR1}]+[\mathrm{hiC3bC4dCR1}]+[\mathrm{hiC3bC4bCR1}]+[\mathrm{hiC3bC4bC4BP}]+[\mathrm{hC3dgC4bCR1}]}\cdots \\
\dfrac{1}{+[\mathrm{hC3dgC4bC4BP}]+[\mathrm{hC3bC3bH}]+[\mathrm{hC3bC3bHL}]+[\mathrm{hC3bC3bCR1}]+[\mathrm{hC3biC3bH}]+[\mathrm{hC3biC3bHL}]+[\mathrm{hC3biC3bCR1}]+[\mathrm{hiC3biC3bCR1}]}\cdots \\
\dfrac{1}{+[\mathrm{hiC3bC3dgCR1}]+[\mathrm{fC4bCR1}]+[\mathrm{nfC4bC4BP}]+[\mathrm{fC4bC4BP}]+[\mathrm{fC4bC4bCR1}]+[\mathrm{fC4bC4bC4BP}]+[\mathrm{fC4bC4dCR1}]+[\mathrm{fC4bC4dC4BP}]+[\mathrm{hC4bCR1}]}\cdots \\
\dfrac{1}{+[\mathrm{hC4bC4BP}]+[\mathrm{hC4bC4bCR1}]+[\mathrm{hC4bC4bC4BP}]+[\mathrm{hC4bC4dCR1}]+[\mathrm{hC4bC4dC4BP}]}\cdots
\end{array}\right)
$$

$$
-k^{+}_{\mathrm{C3biC3bCR1}}[\mathrm{fC3biC3b}][\mathrm{CR1}]+k^{-}_{\mathrm{C3biC3bCR1}}[\mathrm{fC3biC3bCR1}]
$$

$$
+\left(\begin{array}{l}
\dfrac{k^{\mathrm{FI}}_{\mathrm{catC3bH}}[\mathrm{I}][\mathrm{fC3biC3bCR1}]}{K^{\mathrm{FI}}_{\mathrm{mC3bH}}+[\mathrm{C3(H_2O)H}]+[\mathrm{C3(H_2O)HL}]+[\mathrm{fC3bH}]+[\mathrm{fC3bHL}]+[\mathrm{fC3bCR1}]+[\mathrm{fiC3bCR1}]+[\mathrm{IgGC3bC3bH}]+[\mathrm{IgGC3biC3bH}]+[\mathrm{IgGC3bC3bHL}]}\cdots \\
\dfrac{1}{+[\mathrm{IgGC3biC3bHL}]+[\mathrm{IgGC3bC3bCR1}]+[\mathrm{IgGC3biC3bCR1}]+[\mathrm{IgGiC3biC3bCR1}]+[\mathrm{IgGiC3bC3dgCR1}]+[\mathrm{fC3bC4bH}]+[\mathrm{fC3bC4bHL}]}\cdots \\
\dfrac{1}{+[\mathrm{fC3bC4bCR1}]+[\mathrm{fC3bC4bC4BP}]+[\mathrm{fC3bC4dH}]+[\mathrm{fC3bC4dHL}]+[\mathrm{fC3bC4dCR1}]+[\mathrm{fiC3bC4dCR1}]+[\mathrm{fiC3bC4bCR1}]+[\mathrm{fiC3bC4bC4BP}]}\cdots \\
\dfrac{1}{+[\mathrm{fC3dgC4bCR1}]+[\mathrm{fC3dgC4bC4BP}]+[\mathrm{fC3bC3bH}]+[\mathrm{fC3bC3bHL}]+[\mathrm{fC3bC3bCR1}]+[\mathrm{fC3biC3bH}]+[\mathrm{fC3biC3bHL}]+[\mathrm{fC3biC3bCR1}]}\cdots \\
\dfrac{1}{+[\mathrm{fiC3biC3bCR1}]+[\mathrm{fiC3bC3dgCR1}]+[\mathrm{hC3bH}]+[\mathrm{hC3bHL}]+[\mathrm{hC3bCR1}]+[\mathrm{hiC3bCR1}]+[\mathrm{hC3bC4bH}]+[\mathrm{hC3bC4bHL}]+[\mathrm{hC3bC4bCR1}]}\cdots \\
\dfrac{1}{+[\mathrm{hC3bC4bC4BP}]+[\mathrm{hC3bC4dH}]+[\mathrm{hC3bC4dHL}]+[\mathrm{hC3bC4dCR1}]+[\mathrm{hiC3bC4dCR1}]+[\mathrm{hiC3bC4bCR1}]+[\mathrm{hiC3bC4bC4BP}]+[\mathrm{hC3dgC4bCR1}]}\cdots \\
\dfrac{1}{+[\mathrm{hC3dgC4bC4BP}]+[\mathrm{hC3bC3bH}]+[\mathrm{hC3bC3bHL}]+[\mathrm{hC3bC3bCR1}]+[\mathrm{hC3biC3bH}]+[\mathrm{hC3biC3bHL}]+[\mathrm{hC3biC3bCR1}]+[\mathrm{hiC3biC3bCR1}]}\cdots \\
\dfrac{1}{+[\mathrm{hiC3bC3dgCR1}]+[\mathrm{fC4bCR1}]+[\mathrm{nfC4bC4BP}]+[\mathrm{fC4bC4BP}]+[\mathrm{fC4bC4bCR1}]+[\mathrm{fC4bC4bC4BP}]+[\mathrm{fC4bC4dCR1}]+[\mathrm{fC4bC4dC4BP}]+[\mathrm{hC4bCR1}]}\cdots \\
\dfrac{1}{+[\mathrm{hC4bC4BP}]+[\mathrm{hC4bC4bCR1}]+[\mathrm{hC4bC4bC4BP}]+[\mathrm{hC4bC4dCR1}]+[\mathrm{hC4bC4dC4BP}]}\cdots
\end{array}\right)
$$

$$
-k^{+}_{\mathrm{iC3biC3bCR1}}[\mathrm{fiC3biC3b}][\mathrm{CR1}]+k^{-}_{\mathrm{iC3biC3bCR1}}[\mathrm{fiC3biC3bCR1}]
$$

$$+\left(\frac{k^{\mathrm{FI}}_{\mathrm{catC3bH}}[\mathrm{I}][\mathrm{fiC3biC3bCR1}]}{K^{\mathrm{FI}}_{\mathrm{mC3bH}}+[\mathrm{C3(H_2O)H}]+[\mathrm{C3(H_2O)HL}]+[\mathrm{fC3bH}]+[\mathrm{fC3bHL}]+[\mathrm{fC3bCR1}]+[\mathrm{fiC3bCR1}]+[\mathrm{IgGC3bC3bH}]+[\mathrm{IgGC3biC3bH}]+[\mathrm{IgGC3bC3bHL}]}\cdots\right.$$

$$\frac{1}{+[\mathrm{IgGC3biC3bHL}]+[\mathrm{IgGC3bC3bCR1}]+[\mathrm{IgGC3biC3bCR1}]+[\mathrm{IgGiC3biC3bCR1}]+[\mathrm{IgGiC3bC3dgCR1}]+[\mathrm{fC3bC4bH}]+[\mathrm{fC3bC4bHL}]}\cdots$$

$$\frac{1}{+[\mathrm{fC3bC4bCR1}]+[\mathrm{fC3bC4bC4BP}]+[\mathrm{fC3bC4dH}]+[\mathrm{fC3bC4dHL}]+[\mathrm{fC3bC4dCR1}]+[\mathrm{fiC3bC4dCR1}]+[\mathrm{fiC3bC4bCR1}]+[\mathrm{fiC3bC4bC4BP}]}\cdots$$

$$\frac{1}{+[\mathrm{fC3dgC4bCR1}]+[\mathrm{fC3dgC4bC4BP}]+[\mathrm{fC3bC3bH}]+[\mathrm{fC3bC3bHL}]+[\mathrm{fC3bC3bCR1}]+[\mathrm{fC3biC3bH}]+[\mathrm{fC3biC3bHL}]+[\mathrm{fC3biC3bCR1}]}\cdots$$

$$\frac{1}{+[\mathrm{fiC3biC3bCR1}]+[\mathrm{fiC3bC3dgCR1}]+[\mathrm{hC3bH}]+[\mathrm{hC3bHL}]+[\mathrm{hC3bCR1}]+[\mathrm{hiC3bCR1}]+[\mathrm{hC3bC4bH}]+[\mathrm{hC3bC4bHL}]+[\mathrm{hC3bC4bCR1}]}\cdots$$

$$\frac{1}{+[\mathrm{hC3bC4bC4BP}]+[\mathrm{hC3bC4dH}]+[\mathrm{hC3bC4dHL}]+[\mathrm{hC3bC4dCR1}]+[\mathrm{hiC3bC4dCR1}]+[\mathrm{hiC3bC4bCR1}]+[\mathrm{hiC3bC4bC4BP}]+[\mathrm{hC3dgC4bCR1}]}\cdots$$

$$\frac{1}{+[\mathrm{hC3dgC4bC4BP}]+[\mathrm{hC3bC3bH}]+[\mathrm{hC3bC3bHL}]+[\mathrm{hC3bC3bCR1}]+[\mathrm{hC3biC3bH}]+[\mathrm{hC3biC3bHL}]+[\mathrm{hC3biC3bCR1}]+[\mathrm{hiC3biC3bCR1}]}\cdots$$

$$\frac{1}{+[\mathrm{hiC3bC3dgCR1}]+[\mathrm{fC4bCR1}]+[\mathrm{nfC4bC4BP}]+[\mathrm{fC4bC4BP}]+[\mathrm{fC4bC4bCR1}]+[\mathrm{fC4bC4bC4BP}]+[\mathrm{fC4bC4dCR1}]+[\mathrm{fC4bC4dC4BP}]+[\mathrm{hC4bCR1}]}\cdots$$

$$\left.\frac{1}{+[\mathrm{hC4bC4BP}]+[\mathrm{hC4bC4bCR1}]+[\mathrm{hC4bC4bC4BP}]+[\mathrm{hC4bC4dCR1}]+[\mathrm{hC4bC4dC4BP}]}\cdots\right)$$

$$-k^{+}_{\mathrm{iC3bC3dgCR1}}[\mathrm{fiC3bC3dg}][\mathrm{CR1}]+k^{-}_{\mathrm{iC3bC3dgCR1}}[\mathrm{fiC3bC3dgCR1}]$$

$$+\left(\frac{k^{\mathrm{FI}}_{\mathrm{catC3bH}}[\mathrm{I}][\mathrm{fiC3bC3dgCR1}]}{K^{\mathrm{FI}}_{\mathrm{mC3bH}}+[\mathrm{C3(H_2O)H}]+[\mathrm{C3(H_2O)HL}]+[\mathrm{fC3bH}]+[\mathrm{fC3bHL}]+[\mathrm{fC3bCR1}]+[\mathrm{fiC3bCR1}]+[\mathrm{IgGC3bC3bH}]+[\mathrm{IgGC3biC3bH}]+[\mathrm{IgGC3bC3bHL}]}\cdots\right.$$

$$\frac{1}{+[\mathrm{IgGC3biC3bHL}]+[\mathrm{IgGC3bC3bCR1}]+[\mathrm{IgGC3biC3bCR1}]+[\mathrm{IgGiC3biC3bCR1}]+[\mathrm{IgGiC3bC3dgCR1}]+[\mathrm{fC3bC4bH}]+[\mathrm{fC3bC4bHL}]}\cdots$$

$$\frac{1}{+[\mathrm{fC3bC4bCR1}]+[\mathrm{fC3bC4bC4BP}]+[\mathrm{fC3bC4dH}]+[\mathrm{fC3bC4dHL}]+[\mathrm{fC3bC4dCR1}]+[\mathrm{fiC3bC4dCR1}]+[\mathrm{fiC3bC4bCR1}]+[\mathrm{fiC3bC4bC4BP}]}\cdots$$

$$\frac{1}{+[\mathrm{fC3dgC4bCR1}]+[\mathrm{fC3dgC4bC4BP}]+[\mathrm{fC3bC3bH}]+[\mathrm{fC3bC3bHL}]+[\mathrm{fC3bC3bCR1}]+[\mathrm{fC3biC3bH}]+[\mathrm{fC3biC3bHL}]+[\mathrm{fC3biC3bCR1}]}\cdots$$

$$\frac{1}{+[\mathrm{fiC3biC3bCR1}]+[\mathrm{fiC3bC3dgCR1}]+[\mathrm{hC3bH}]+[\mathrm{hC3bHL}]+[\mathrm{hC3bCR1}]+[\mathrm{hiC3bCR1}]+[\mathrm{hC3bC4bH}]+[\mathrm{hC3bC4bHL}]+[\mathrm{hC3bC4bCR1}]}\cdots$$

$$\frac{1}{+[\mathrm{hC3bC4bC4BP}]+[\mathrm{hC3bC4dH}]+[\mathrm{hC3bC4dHL}]+[\mathrm{hC3bC4dCR1}]+[\mathrm{hiC3bC4dCR1}]+[\mathrm{hiC3bC4bCR1}]+[\mathrm{hiC3bC4bC4BP}]+[\mathrm{hC3dgC4bCR1}]}\cdots$$

$$\frac{1}{+[\mathrm{hC3dgC4bC4BP}]+[\mathrm{hC3bC3bH}]+[\mathrm{hC3bC3bHL}]+[\mathrm{hC3bC3bCR1}]+[\mathrm{hC3biC3bH}]+[\mathrm{hC3biC3bHL}]+[\mathrm{hC3biC3bCR1}]+[\mathrm{hiC3biC3bCR1}]}\cdots$$

$$\frac{1}{+[\mathrm{hiC3bC3dgCR1}]+[\mathrm{fC4bCR1}]+[\mathrm{nfC4bC4BP}]+[\mathrm{fC4bC4BP}]+[\mathrm{fC4bC4bCR1}]+[\mathrm{fC4bC4bC4BP}]+[\mathrm{fC4bC4dCR1}]+[\mathrm{fC4bC4dC4BP}]+[\mathrm{hC4bCR1}]}\cdots$$

$$\left.\frac{1}{+[\mathrm{hC4bC4BP}]+[\mathrm{hC4bC4bCR1}]+[\mathrm{hC4bC4bC4BP}]+[\mathrm{hC4bC4dCR1}]+[\mathrm{hC4bC4dC4BP}]}\cdots\right)$$

$$-k^{+}_{\mathrm{C3bC3bCR1}}[\mathrm{fC3bC3bBb}][\mathrm{CR1}]+k^{-}_{\mathrm{C3bC3bCR1}}[\mathrm{fC3bC3bBbCR1}]+k^{-}_{\mathrm{C3bBbCR1}_{\mathrm{decay}}}[\mathrm{fC3bC3bBbCR1}]-k^{+}_{\mathrm{C4bCR1}}[\mathrm{fC4b}][\mathrm{CR1}]+k^{-}_{\mathrm{C4bCR1}}[\mathrm{fC4bCR1}]$$

$$+\left(\frac{k^{\mathrm{FI}}_{\mathrm{catC3bH}}[\mathrm{I}][\mathrm{fC4bCR1}]}{K^{\mathrm{FI}}_{\mathrm{mC3bH}}+[\mathrm{C3(H_2O)H}]+[\mathrm{C3(H_2O)HL}]+[\mathrm{fC3bH}]+[\mathrm{fC3bHL}]+[\mathrm{fC3bCR1}]+[\mathrm{fiC3bCR1}]+[\mathrm{IgGC3bC3bH}]+[\mathrm{IgGC3biC3bH}]+[\mathrm{IgGC3bC3bHL}]}\cdots\right.$$

$$\frac{1}{+[\mathrm{IgGC3biC3bHL}]+[\mathrm{IgGC3bC3bCR1}]+[\mathrm{IgGC3biC3bCR1}]+[\mathrm{IgGiC3biC3bCR1}]+[\mathrm{IgGiC3bC3dgCR1}]+[\mathrm{fC3bC4bH}]+[\mathrm{fC3bC4bHL}]}\cdots$$

$$\frac{1}{+[\mathrm{fC3bC4bCR1}]+[\mathrm{fC3bC4bC4BP}]+[\mathrm{fC3bC4dH}]+[\mathrm{fC3bC4dHL}]+[\mathrm{fC3bC4dCR1}]+[\mathrm{fiC3bC4dCR1}]+[\mathrm{fiC3bC4bCR1}]+[\mathrm{fiC3bC4bC4BP}]}\cdots$$

$$\frac{1}{+[\mathrm{fC3dgC4bCR1}]+[\mathrm{fC3dgC4bC4BP}]+[\mathrm{fC3bC3bH}]+[\mathrm{fC3bC3bHL}]+[\mathrm{fC3bC3bCR1}]+[\mathrm{fC3biC3bH}]+[\mathrm{fC3biC3bHL}]+[\mathrm{fC3biC3bCR1}]}\cdots$$

$$\frac{1}{+[\mathrm{fiC3biC3bCR1}]+[\mathrm{fiC3bC3dgCR1}]+[\mathrm{hC3bH}]+[\mathrm{hC3bHL}]+[\mathrm{hC3bCR1}]+[\mathrm{hiC3bCR1}]+[\mathrm{hC3bC4bH}]+[\mathrm{hC3bC4bHL}]+[\mathrm{hC3bC4bCR1}]}\cdots$$

$$\frac{1}{+[\mathrm{hC3bC4bC4BP}]+[\mathrm{hC3bC4dH}]+[\mathrm{hC3bC4dHL}]+[\mathrm{hC3bC4dCR1}]+[\mathrm{hiC3bC4dCR1}]+[\mathrm{hiC3bC4bCR1}]+[\mathrm{hiC3bC4bC4BP}]+[\mathrm{hC3dgC4bCR1}]}\cdots$$

$$\frac{1}{+[\mathrm{hC3dgC4bC4BP}]+[\mathrm{hC3bC3bH}]+[\mathrm{hC3bC3bHL}]+[\mathrm{hC3bC3bCR1}]+[\mathrm{hC3biC3bH}]+[\mathrm{hC3biC3bHL}]+[\mathrm{hC3biC3bCR1}]+[\mathrm{hiC3biC3bCR1}]}\cdots$$

$$\frac{1}{+[\mathrm{hiC3bC3dgCR1}]+[\mathrm{fC4bCR1}]+[\mathrm{nfC4bC4BP}]+[\mathrm{fC4bC4BP}]+[\mathrm{fC4bC4bCR1}]+[\mathrm{fC4bC4bC4BP}]+[\mathrm{fC4bC4dCR1}]+[\mathrm{fC4bC4dC4BP}]+[\mathrm{hC4bCR1}]}\cdots$$

$$\left.\frac{1}{+[\mathrm{hC4bC4BP}]+[\mathrm{hC4bC4bCR1}]+[\mathrm{hC4bC4bC4BP}]+[\mathrm{hC4bC4dCR1}]+[\mathrm{hC4bC4dC4BP}]}\cdots\right)$$

$$-k^{+}_{\mathrm{C4bCR1}}[\mathrm{fC4bC2a}][\mathrm{CR1}]+k^{-}_{\mathrm{C4bCR1}}[\mathrm{fC4bC2aCR1}]+k^{-}_{\mathrm{C4bC2aCR1}_{\mathrm{decay}}}[\mathrm{fC4bC2aCR1}]-k^{+}_{\mathrm{C4bC4bCR1}}[\mathrm{fC4bC4bC2a}][\mathrm{CR1}]+k^{-}_{\mathrm{C4bC4bCR1}}[\mathrm{fC4bC4bC2aCR1}]$$

$$+k^{-}_{\mathrm{C4bC2aCR1}_{\mathrm{decay}}}[\mathrm{fC4bC4bC2aCR1}]-k^{+}_{\mathrm{C3bC4bCR1}}[\mathrm{fC3bC4bC2a}][\mathrm{CR1}]+k^{-}_{\mathrm{C3bC4bCR1}}[\mathrm{fC3bC4bC2aCR1}]+k^{-}_{\mathrm{C4bC2aCR1}_{\mathrm{decay}}}[\mathrm{fC3bC4bC2aCR1}]$$

$$-k^{+}_{\mathrm{C4bC4bCR1}}[\mathrm{fC4bC4b}][\mathrm{CR1}]+k^{-}_{\mathrm{C4bC4bCR1}}[\mathrm{fC4bC4bCR1}]$$

$$+\left(\frac{k^{\mathrm{FI}}_{\mathrm{catC3bH}}[\mathrm{I}][\mathrm{fC4bC4bCR1}]}{K^{\mathrm{FI}}_{\mathrm{mC3bH}}+[\mathrm{C3(H_2O)H}]+[\mathrm{C3(H_2O)HL}]+[\mathrm{fC3bH}]+[\mathrm{fC3bHL}]+[\mathrm{fC3bCR1}]+[\mathrm{fiC3bCR1}]+[\mathrm{IgGC3bC3bH}]+[\mathrm{IgGC3biC3bH}]+[\mathrm{IgGC3bC3bHL}]}\cdots\right.$$

$$\frac{1}{+[\mathrm{IgGC3biC3bHL}]+[\mathrm{IgGC3bC3bCR1}]+[\mathrm{IgGC3biC3bCR1}]+[\mathrm{IgGiC3biC3bCR1}]+[\mathrm{IgGiC3bC3dgCR1}]+[\mathrm{fC3bC4bH}]+[\mathrm{fC3bC4bHL}]}\cdots$$

$$\frac{1}{+[\mathrm{fC3bC4bCR1}]+[\mathrm{fC3bC4bC4BP}]+[\mathrm{fC3bC4dH}]+[\mathrm{fC3bC4dHL}]+[\mathrm{fC3bC4dCR1}]+[\mathrm{fiC3bC4dCR1}]+[\mathrm{fiC3bC4bCR1}]+[\mathrm{fiC3bC4bC4BP}]}\cdots$$

$$\frac{1}{+[\mathrm{fC3dgC4bCR1}]+[\mathrm{fC3dgC4bC4BP}]+[\mathrm{fC3bC3bH}]+[\mathrm{fC3bC3bHL}]+[\mathrm{fC3bC3bCR1}]+[\mathrm{fC3biC3bH}]+[\mathrm{fC3biC3bHL}]+[\mathrm{fC3biC3bCR1}]}\cdots$$

$$\frac{1}{+[\mathrm{fiC3biC3bCR1}]+[\mathrm{fiC3bC3dgCR1}]+[\mathrm{hC3bH}]+[\mathrm{hC3bHL}]+[\mathrm{hC3bCR1}]+[\mathrm{hiC3bCR1}]+[\mathrm{hC3bC4bH}]+[\mathrm{hC3bC4bHL}]+[\mathrm{hC3bC4bCR1}]}\cdots$$

$$\frac{1}{+[\mathrm{hC3bC4bC4BP}]+[\mathrm{hC3bC4dH}]+[\mathrm{hC3bC4dHL}]+[\mathrm{hC3bC4dCR1}]+[\mathrm{hiC3bC4dCR1}]+[\mathrm{hiC3bC4bCR1}]+[\mathrm{hiC3bC4bC4BP}]+[\mathrm{hC3dgC4bCR1}]}\cdots$$

$$\frac{1}{+[\mathrm{hC3dgC4bC4BP}]+[\mathrm{hC3bC3bH}]+[\mathrm{hC3bC3bHL}]+[\mathrm{hC3bC3bCR1}]+[\mathrm{hC3biC3bH}]+[\mathrm{hC3biC3bHL}]+[\mathrm{hC3biC3bCR1}]+[\mathrm{hiC3biC3bCR1}]}\cdots$$

$$\frac{1}{+[\mathrm{hiC3bC3dgCR1}]+[\mathrm{fC4bCR1}]+[\mathrm{nfC4bC4BP}]+[\mathrm{fC4bC4BP}]+[\mathrm{fC4bC4bCR1}]+[\mathrm{fC4bC4bC4BP}]+[\mathrm{fC4bC4dCR1}]+[\mathrm{fC4bC4dC4BP}]+[\mathrm{hC4bCR1}]}\cdots$$

$$\left.\frac{1}{+[\mathrm{hC4bC4BP}]+[\mathrm{hC4bC4bCR1}]+[\mathrm{hC4bC4bC4BP}]+[\mathrm{hC4bC4dCR1}]+[\mathrm{hC4bC4dC4BP}]}\cdots\right)$$

$$-k^{+}_{\mathrm{C4bC4dCR1}}[\mathrm{fC4bC4d}][\mathrm{CR1}]+k^{-}_{\mathrm{C4bC4dCR1}}[\mathrm{fC4bC4dCR1}]$$

$$+\left(\frac{k_{\mathrm{catC3bH}}^{\mathrm{FI}}[\mathrm{I}][\mathrm{fC4bC4dCR1}]}{K_{\mathrm{mC3bH}}^{\mathrm{FI}}+[\mathrm{C3(H_2O)H}]+[\mathrm{C3(H_2O)HL}]+[\mathrm{fC3bH}]+[\mathrm{fC3bHL}]+[\mathrm{fC3bCR1}]+[\mathrm{fiC3bCR1}]+[\mathrm{IgGC3bC3bH}]+[\mathrm{IgGC3biC3bH}]+[\mathrm{IgGC3bC3bHL}]}\cdots\right.$$

$$\frac{1}{+[\mathrm{IgGC3biC3bHL}]+[\mathrm{IgGC3bC3bCR1}]+[\mathrm{IgGC3biC3bCR1}]+[\mathrm{IgGiC3biC3bCR1}]+[\mathrm{IgGiC3bC3dgCR1}]+[\mathrm{fC3bC4bH}]+[\mathrm{fC3bC4bHL}]}\cdots$$

$$\frac{1}{+[\mathrm{fC3bC4bCR1}]+[\mathrm{fC3bC4bC4BP}]+[\mathrm{fC3bC4dH}]+[\mathrm{fC3bC4dHL}]+[\mathrm{fC3bC4dCR1}]+[\mathrm{fiC3bC4dCR1}]+[\mathrm{fiC3bC4bCR1}]+[\mathrm{fiC3bC4bC4BP}]}\cdots$$

$$\frac{1}{+[\mathrm{fC3dgC4bCR1}]+[\mathrm{fC3dgC4bC4BP}]+[\mathrm{fC3bC3bH}]+[\mathrm{fC3bC3bHL}]+[\mathrm{fC3bC3bCR1}]+[\mathrm{fC3biC3bH}]+[\mathrm{fC3biC3bHL}]+[\mathrm{fC3biC3bCR1}]}\cdots$$

$$\frac{1}{+[\mathrm{fiC3biC3bCR1}]+[\mathrm{fiC3bC3dgCR1}]+[\mathrm{hC3bH}]+[\mathrm{hC3bHL}]+[\mathrm{hC3bCR1}]+[\mathrm{hiC3bCR1}]+[\mathrm{hC3bC4bH}]+[\mathrm{hC3bC4bHL}]+[\mathrm{hC3bC4bCR1}]}\cdots$$

$$\frac{1}{+[\mathrm{hC3bC4bC4BP}]+[\mathrm{hC3bC4dH}]+[\mathrm{hC3bC4dHL}]+[\mathrm{hC3bC4dCR1}]+[\mathrm{hiC3bC4dCR1}]+[\mathrm{hiC3bC4bCR1}]+[\mathrm{hiC3bC4bC4BP}]+[\mathrm{hC3dgC4bCR1}]}\cdots$$

$$\frac{1}{+[\mathrm{hC3dgC4bC4BP}]+[\mathrm{hC3bC3bH}]+[\mathrm{hC3bC3bHL}]+[\mathrm{hC3bC3bCR1}]+[\mathrm{hC3biC3bH}]+[\mathrm{hC3biC3bHL}]+[\mathrm{hC3biC3bCR1}]+[\mathrm{hiC3biC3bCR1}]}\cdots$$

$$\frac{1}{+[\mathrm{hiC3bC3dgCR1}]+[\mathrm{fC4bCR1}]+[\mathrm{nfC4bC4BP}]+[\mathrm{fC4bC4BP}]+[\mathrm{fC4bC4bCR1}]+[\mathrm{fC4bC4bC4BP}]+[\mathrm{fC4bC4dCR1}]+[\mathrm{fC4bC4dC4BP}]+[\mathrm{hC4bCR1}]}\cdots$$

$$\left.\frac{1}{+[\mathrm{hC4bC4BP}]+[\mathrm{hC4bC4bCR1}]+[\mathrm{hC4bC4bC4BP}]+[\mathrm{hC4bC4dCR1}]+[\mathrm{hC4bC4dC4BP}]}\cdots\right)$$

$$-k_{\mathrm{C3bCR1}}^{+}[\mathrm{hC3b}][\mathrm{CR1}]+k_{\mathrm{C3bCR1}}^{-}[\mathrm{hC3bCR1}]$$

$$+\left(\frac{k_{\mathrm{catC3bH}}^{\mathrm{FI}}[\mathrm{I}][\mathrm{hC3bCR1}]}{K_{\mathrm{mC3bH}}^{\mathrm{FI}}+[\mathrm{C3(H_2O)H}]+[\mathrm{C3(H_2O)HL}]+[\mathrm{fC3bH}]+[\mathrm{fC3bHL}]+[\mathrm{fC3bCR1}]+[\mathrm{fiC3bCR1}]+[\mathrm{IgGC3bC3bH}]+[\mathrm{IgGC3biC3bH}]+[\mathrm{IgGC3bC3bHL}]}\cdots\right.$$

$$\frac{1}{+[\mathrm{IgGC3biC3bHL}]+[\mathrm{IgGC3bC3bCR1}]+[\mathrm{IgGC3biC3bCR1}]+[\mathrm{IgGiC3biC3bCR1}]+[\mathrm{IgGiC3bC3dgCR1}]+[\mathrm{fC3bC4bH}]+[\mathrm{fC3bC4bHL}]}\cdots$$

$$\frac{1}{+[\mathrm{fC3bC4bCR1}]+[\mathrm{fC3bC4bC4BP}]+[\mathrm{fC3bC4dH}]+[\mathrm{fC3bC4dHL}]+[\mathrm{fC3bC4dCR1}]+[\mathrm{fiC3bC4dCR1}]+[\mathrm{fiC3bC4bCR1}]+[\mathrm{fiC3bC4bC4BP}]}\cdots$$

$$\frac{1}{+[\mathrm{fC3dgC4bCR1}]+[\mathrm{fC3dgC4bC4BP}]+[\mathrm{fC3bC3bH}]+[\mathrm{fC3bC3bHL}]+[\mathrm{fC3bC3bCR1}]+[\mathrm{fC3biC3bH}]+[\mathrm{fC3biC3bHL}]+[\mathrm{fC3biC3bCR1}]}\cdots$$

$$\frac{1}{+[\mathrm{fiC3biC3bCR1}]+[\mathrm{fiC3bC3dgCR1}]+[\mathrm{hC3bH}]+[\mathrm{hC3bHL}]+[\mathrm{hC3bCR1}]+[\mathrm{hiC3bCR1}]+[\mathrm{hC3bC4bH}]+[\mathrm{hC3bC4bHL}]+[\mathrm{hC3bC4bCR1}]}\cdots$$

$$\frac{1}{+[\mathrm{hC3bC4bC4BP}]+[\mathrm{hC3bC4dH}]+[\mathrm{hC3bC4dHL}]+[\mathrm{hC3bC4dCR1}]+[\mathrm{hiC3bC4dCR1}]+[\mathrm{hiC3bC4bCR1}]+[\mathrm{hiC3bC4bC4BP}]+[\mathrm{hC3dgC4bCR1}]}\cdots$$

$$\frac{1}{+[\mathrm{hC3dgC4bC4BP}]+[\mathrm{hC3bC3bH}]+[\mathrm{hC3bC3bHL}]+[\mathrm{hC3bC3bCR1}]+[\mathrm{hC3biC3bH}]+[\mathrm{hC3biC3bHL}]+[\mathrm{hC3biC3bCR1}]+[\mathrm{hiC3biC3bCR1}]}\cdots$$

$$\frac{1}{+[\mathrm{hiC3bC3dgCR1}]+[\mathrm{fC4bCR1}]+[\mathrm{nfC4bC4BP}]+[\mathrm{fC4bC4BP}]+[\mathrm{fC4bC4bCR1}]+[\mathrm{fC4bC4bC4BP}]+[\mathrm{fC4bC4dCR1}]+[\mathrm{fC4bC4dC4BP}]+[\mathrm{hC4bCR1}]}\cdots$$

$$\left.\frac{1}{+[\mathrm{hC4bC4BP}]+[\mathrm{hC4bC4bCR1}]+[\mathrm{hC4bC4bC4BP}]+[\mathrm{hC4bC4dCR1}]+[\mathrm{hC4bC4dC4BP}]}\cdots\right)$$

$$-k_{\mathrm{C3bCR1}}^{+}[\mathrm{hC3bBb}][\mathrm{CR1}]+k_{\mathrm{C3bCR1}}^{-}[\mathrm{hC3bBbCR1}]+k_{\mathrm{C3bBbCR1}_{\mathrm{decay}}}^{-}[\mathrm{hC3bBbCR1}]-k_{\mathrm{iC3bCR1}}^{+}[\mathrm{hiC3b}][\mathrm{CR1}]+k_{\mathrm{iC3bCR1}}^{-}[\mathrm{hiC3bCR1}]$$

$$+\left(\frac{k_{\mathrm{catC3bH}}^{\mathrm{FI}}[\mathrm{I}][\mathrm{hiC3bCR1}]}{K_{\mathrm{mC3bH}}^{\mathrm{FI}}+[\mathrm{C3(H_2O)H}]+[\mathrm{C3(H_2O)HL}]+[\mathrm{fC3bH}]+[\mathrm{fC3bHL}]+[\mathrm{fC3bCR1}]+[\mathrm{fiC3bCR1}]+[\mathrm{IgGC3bC3bH}]+[\mathrm{IgGC3biC3bH}]+[\mathrm{IgGC3bC3bHL}]}\cdots\right.$$

$$\frac{1}{+[\mathrm{IgGC3biC3bHL}]+[\mathrm{IgGC3bC3bCR1}]+[\mathrm{IgGC3biC3bCR1}]+[\mathrm{IgGiC3biC3bCR1}]+[\mathrm{IgGiC3bC3dgCR1}]+[\mathrm{fC3bC4bH}]+[\mathrm{fC3bC4bHL}]}\cdots$$

$$\frac{1}{+[\mathrm{fC3bC4bCR1}]+[\mathrm{fC3bC4bC4BP}]+[\mathrm{fC3bC4dH}]+[\mathrm{fC3bC4dHL}]+[\mathrm{fC3bC4dCR1}]+[\mathrm{fiC3bC4dCR1}]+[\mathrm{fiC3bC4bCR1}]+[\mathrm{fiC3bC4bC4BP}]}\cdots$$

$$\frac{1}{+[\mathrm{fC3dgC4bCR1}]+[\mathrm{fC3dgC4bC4BP}]+[\mathrm{fC3bC3bH}]+[\mathrm{fC3bC3bHL}]+[\mathrm{fC3bC3bCR1}]+[\mathrm{fC3biC3bH}]+[\mathrm{fC3biC3bHL}]+[\mathrm{fC3biC3bCR1}]}\cdots$$

$$\frac{1}{+[\mathrm{fiC3biC3bCR1}]+[\mathrm{fiC3bC3dgCR1}]+[\mathrm{hC3bH}]+[\mathrm{hC3bHL}]+[\mathrm{hC3bCR1}]+[\mathrm{hiC3bCR1}]+[\mathrm{hC3bC4bH}]+[\mathrm{hC3bC4bHL}]+[\mathrm{hC3bC4bCR1}]}\cdots$$

$$\frac{1}{+[\mathrm{hC3bC4bC4BP}]+[\mathrm{hC3bC4dH}]+[\mathrm{hC3bC4dHL}]+[\mathrm{hC3bC4dCR1}]+[\mathrm{hiC3bC4dCR1}]+[\mathrm{hiC3bC4bCR1}]+[\mathrm{hiC3bC4bC4BP}]+[\mathrm{hC3dgC4bCR1}]}\cdots$$

$$\frac{1}{+[\mathrm{hC3dgC4bC4BP}]+[\mathrm{hC3bC3bH}]+[\mathrm{hC3bC3bHL}]+[\mathrm{hC3bC3bCR1}]+[\mathrm{hC3biC3bH}]+[\mathrm{hC3biC3bHL}]+[\mathrm{hC3biC3bCR1}]+[\mathrm{hiC3biC3bCR1}]}\cdots$$

$$\frac{1}{+[\mathrm{hiC3bC3dgCR1}]+[\mathrm{fC4bCR1}]+[\mathrm{nfC4bC4BP}]+[\mathrm{fC4bC4BP}]+[\mathrm{fC4bC4bCR1}]+[\mathrm{fC4bC4bC4BP}]+[\mathrm{fC4bC4dCR1}]+[\mathrm{fC4bC4dC4BP}]+[\mathrm{hC4bCR1}]}\cdots$$

$$\left.\frac{1}{+[\mathrm{hC4bC4BP}]+[\mathrm{hC4bC4bCR1}]+[\mathrm{hC4bC4bC4BP}]+[\mathrm{hC4bC4dCR1}]+[\mathrm{hC4bC4dC4BP}]}\cdots\right)$$

$$-k_{\mathrm{C3bC4bCR1}}^{+}[\mathrm{hC3bC4b}][\mathrm{CR1}]+k_{\mathrm{C3bC4bCR1}}^{-}[\mathrm{hC3bC4bCR1}]$$

$$+\left(\frac{k_{\mathrm{catC3bH}}^{\mathrm{FI}}[\mathrm{I}][\mathrm{hC3bC4bCR1}]}{K_{\mathrm{mC3bH}}^{\mathrm{FI}}+[\mathrm{C3(H_2O)H}]+[\mathrm{C3(H_2O)HL}]+[\mathrm{fC3bH}]+[\mathrm{fC3bHL}]+[\mathrm{fC3bCR1}]+[\mathrm{fiC3bCR1}]+[\mathrm{IgGC3bC3bH}]+[\mathrm{IgGC3biC3bH}]+[\mathrm{IgGC3bC3bHL}]}\cdots\right.$$

$$\frac{1}{+[\mathrm{IgGC3biC3bHL}]+[\mathrm{IgGC3bC3bCR1}]+[\mathrm{IgGC3biC3bCR1}]+[\mathrm{IgGiC3biC3bCR1}]+[\mathrm{IgGiC3bC3dgCR1}]+[\mathrm{fC3bC4bH}]+[\mathrm{fC3bC4bHL}]}\cdots$$

$$\frac{1}{+[\mathrm{fC3bC4bCR1}]+[\mathrm{fC3bC4bC4BP}]+[\mathrm{fC3bC4dH}]+[\mathrm{fC3bC4dHL}]+[\mathrm{fC3bC4dCR1}]+[\mathrm{fiC3bC4dCR1}]+[\mathrm{fiC3bC4bCR1}]+[\mathrm{fiC3bC4bC4BP}]}\cdots$$

$$\frac{1}{+[\mathrm{fC3dgC4bCR1}]+[\mathrm{fC3dgC4bC4BP}]+[\mathrm{fC3bC3bH}]+[\mathrm{fC3bC3bHL}]+[\mathrm{fC3bC3bCR1}]+[\mathrm{fC3biC3bH}]+[\mathrm{fC3biC3bHL}]+[\mathrm{fC3biC3bCR1}]}\cdots$$

$$\frac{1}{+[\mathrm{fiC3biC3bCR1}]+[\mathrm{fiC3bC3dgCR1}]+[\mathrm{hC3bH}]+[\mathrm{hC3bHL}]+[\mathrm{hC3bCR1}]+[\mathrm{hiC3bCR1}]+[\mathrm{hC3bC4bH}]+[\mathrm{hC3bC4bHL}]+[\mathrm{hC3bC4bCR1}]}\cdots$$

$$\frac{1}{+[\mathrm{hC3bC4bC4BP}]+[\mathrm{hC3bC4dH}]+[\mathrm{hC3bC4dHL}]+[\mathrm{hC3bC4dCR1}]+[\mathrm{hiC3bC4dCR1}]+[\mathrm{hiC3bC4bCR1}]+[\mathrm{hiC3bC4bC4BP}]+[\mathrm{hC3dgC4bCR1}]}\cdots$$

$$\frac{1}{+[\mathrm{hC3dgC4bC4BP}]+[\mathrm{hC3bC3bH}]+[\mathrm{hC3bC3bHL}]+[\mathrm{hC3bC3bCR1}]+[\mathrm{hC3biC3bH}]+[\mathrm{hC3biC3bHL}]+[\mathrm{hC3biC3bCR1}]+[\mathrm{hiC3biC3bCR1}]}\cdots$$

$$\frac{1}{+[\mathrm{hiC3bC3dgCR1}]+[\mathrm{fC4bCR1}]+[\mathrm{nfC4bC4BP}]+[\mathrm{fC4bC4BP}]+[\mathrm{fC4bC4bCR1}]+[\mathrm{fC4bC4bC4BP}]+[\mathrm{fC4bC4dCR1}]+[\mathrm{fC4bC4dC4BP}]+[\mathrm{hC4bCR1}]}\cdots$$

$$\left.\frac{1}{+[\mathrm{hC4bC4BP}]+[\mathrm{hC4bC4bCR1}]+[\mathrm{hC4bC4bC4BP}]+[\mathrm{hC4bC4dCR1}]+[\mathrm{hC4bC4dC4BP}]}\cdots\right)$$

$$-k_{\mathrm{C3bC4dCR1}}^{+}[\mathrm{hC3bC4d}][\mathrm{CR1}]+k_{\mathrm{C3bC4dCR1}}^{-}[\mathrm{hC3bC4dCR1}]$$

$$+\left(\begin{array}{l}
\dfrac{k^{\mathrm{FI}}_{\mathrm{catC3bH}}[\mathrm{I}][\mathrm{hC3bC4dCR1}]}{K^{\mathrm{FI}}_{\mathrm{mC3bH}}+[\mathrm{C3(H_2O)H}]+[\mathrm{C3(H_2O)HL}]+[\mathrm{fC3bH}]+[\mathrm{fC3bHL}]+[\mathrm{fC3bCR1}]+[\mathrm{fiC3bCR1}]+[\mathrm{IgGC3bC3bH}]+[\mathrm{IgGC3biC3bH}]+[\mathrm{IgGC3bC3bHL}]}\cdots \\
\dfrac{1}{+[\mathrm{IgGC3biC3bHL}]+[\mathrm{IgGC3bC3bCR1}]+[\mathrm{IgGC3biC3bCR1}]+[\mathrm{IgGiC3biC3bCR1}]+[\mathrm{IgGiC3bC3dgCR1}]+[\mathrm{fC3bC4bH}]+[\mathrm{fC3bC4bHL}]}\cdots \\
\dfrac{1}{+[\mathrm{fC3bC4bCR1}]+[\mathrm{fC3bC4bC4BP}]+[\mathrm{fC3bC4dH}]+[\mathrm{fC3bC4dHL}]+[\mathrm{fC3bC4dCR1}]+[\mathrm{fiC3bC4dCR1}]+[\mathrm{fiC3bC4bCR1}]+[\mathrm{fiC3bC4bC4BP}]}\cdots \\
\dfrac{1}{+[\mathrm{fC3dgC4bCR1}]+[\mathrm{fC3dgC4bC4BP}]+[\mathrm{fC3bC3bH}]+[\mathrm{fC3bC3bHL}]+[\mathrm{fC3bC3bCR1}]+[\mathrm{fC3biC3bH}]+[\mathrm{fC3biC3bHL}]+[\mathrm{fC3biC3bCR1}]}\cdots \\
\dfrac{1}{+[\mathrm{fiC3biC3bCR1}]+[\mathrm{fiC3bC3dgCR1}]+[\mathrm{hC3bH}]+[\mathrm{hC3bHL}]+[\mathrm{hC3bCR1}]+[\mathrm{hiC3bCR1}]+[\mathrm{hC3bC4bH}]+[\mathrm{hC3bC4bHL}]+[\mathrm{hC3bC4bCR1}]}\cdots \\
\dfrac{1}{+[\mathrm{hC3bC4bC4BP}]+[\mathrm{hC3bC4dH}]+[\mathrm{hC3bC4dHL}]+[\mathrm{hC3bC4dCR1}]+[\mathrm{hiC3bC4dCR1}]+[\mathrm{hiC3bC4bCR1}]+[\mathrm{hiC3bC4bC4BP}]+[\mathrm{hC3dgC4bCR1}]}\cdots \\
\dfrac{1}{+[\mathrm{hC3dgC4bC4BP}]+[\mathrm{hC3bC3bH}]+[\mathrm{hC3bC3bHL}]+[\mathrm{hC3bC3bCR1}]+[\mathrm{hC3biC3bH}]+[\mathrm{hC3biC3bHL}]+[\mathrm{hC3biC3bCR1}]+[\mathrm{hiC3biC3bCR1}]}\cdots \\
\dfrac{1}{+[\mathrm{hiC3bC3dgCR1}]+[\mathrm{fC4bCR1}]+[\mathrm{nfC4bC4BP}]+[\mathrm{fC4bC4BP}]+[\mathrm{fC4bC4bCR1}]+[\mathrm{fC4bC4bC4BP}]+[\mathrm{fC4bC4dCR1}]+[\mathrm{fC4bC4dC4BP}]+[\mathrm{hC4bCR1}]}\cdots \\
\dfrac{1}{+[\mathrm{hC4bC4BP}]+[\mathrm{hC4bC4bCR1}]+[\mathrm{hC4bC4bC4BP}]+[\mathrm{hC4bC4dCR1}]+[\mathrm{hC4bC4dC4BP}]}\cdots
\end{array}\right)$$

$$-k^{+}_{\mathrm{iC3bC4bCR1}}[\mathrm{hiC3bC4b}][\mathrm{CR1}]+k^{-}_{\mathrm{iC3bC4bCR1}}[\mathrm{hiC3bC4bCR1}]$$

$$+\left(\begin{array}{l}
\dfrac{k^{\mathrm{FI}}_{\mathrm{catC3bH}}[\mathrm{I}][\mathrm{hiC3bC4bCR1}]}{K^{\mathrm{FI}}_{\mathrm{mC3bH}}+[\mathrm{C3(H_2O)H}]+[\mathrm{C3(H_2O)HL}]+[\mathrm{fC3bH}]+[\mathrm{fC3bHL}]+[\mathrm{fC3bCR1}]+[\mathrm{fiC3bCR1}]+[\mathrm{IgGC3bC3bH}]+[\mathrm{IgGC3biC3bH}]+[\mathrm{IgGC3bC3bHL}]}\cdots \\
\dfrac{1}{+[\mathrm{IgGC3biC3bHL}]+[\mathrm{IgGC3bC3bCR1}]+[\mathrm{IgGC3biC3bCR1}]+[\mathrm{IgGiC3biC3bCR1}]+[\mathrm{IgGiC3bC3dgCR1}]+[\mathrm{fC3bC4bH}]+[\mathrm{fC3bC4bHL}]}\cdots \\
\dfrac{1}{+[\mathrm{fC3bC4bCR1}]+[\mathrm{fC3bC4bC4BP}]+[\mathrm{fC3bC4dH}]+[\mathrm{fC3bC4dHL}]+[\mathrm{fC3bC4dCR1}]+[\mathrm{fiC3bC4dCR1}]+[\mathrm{fiC3bC4bCR1}]+[\mathrm{fiC3bC4bC4BP}]}\cdots \\
\dfrac{1}{+[\mathrm{fC3dgC4bCR1}]+[\mathrm{fC3dgC4bC4BP}]+[\mathrm{fC3bC3bH}]+[\mathrm{fC3bC3bHL}]+[\mathrm{fC3bC3bCR1}]+[\mathrm{fC3biC3bH}]+[\mathrm{fC3biC3bHL}]+[\mathrm{fC3biC3bCR1}]}\cdots \\
\dfrac{1}{+[\mathrm{fiC3biC3bCR1}]+[\mathrm{fiC3bC3dgCR1}]+[\mathrm{hC3bH}]+[\mathrm{hC3bHL}]+[\mathrm{hC3bCR1}]+[\mathrm{hiC3bCR1}]+[\mathrm{hC3bC4bH}]+[\mathrm{hC3bC4bHL}]+[\mathrm{hC3bC4bCR1}]}\cdots \\
\dfrac{1}{+[\mathrm{hC3bC4bC4BP}]+[\mathrm{hC3bC4dH}]+[\mathrm{hC3bC4dHL}]+[\mathrm{hC3bC4dCR1}]+[\mathrm{hiC3bC4dCR1}]+[\mathrm{hiC3bC4bCR1}]+[\mathrm{hiC3bC4bC4BP}]+[\mathrm{hC3dgC4bCR1}]}\cdots \\
\dfrac{1}{+[\mathrm{hC3dgC4bC4BP}]+[\mathrm{hC3bC3bH}]+[\mathrm{hC3bC3bHL}]+[\mathrm{hC3bC3bCR1}]+[\mathrm{hC3biC3bH}]+[\mathrm{hC3biC3bHL}]+[\mathrm{hC3biC3bCR1}]+[\mathrm{hiC3biC3bCR1}]}\cdots \\
\dfrac{1}{+[\mathrm{hiC3bC3dgCR1}]+[\mathrm{fC4bCR1}]+[\mathrm{nfC4bC4BP}]+[\mathrm{fC4bC4BP}]+[\mathrm{fC4bC4bCR1}]+[\mathrm{fC4bC4bC4BP}]+[\mathrm{fC4bC4dCR1}]+[\mathrm{fC4bC4dC4BP}]+[\mathrm{hC4bCR1}]}\cdots \\
\dfrac{1}{+[\mathrm{hC4bC4BP}]+[\mathrm{hC4bC4bCR1}]+[\mathrm{hC4bC4bC4BP}]+[\mathrm{hC4bC4dCR1}]+[\mathrm{hC4bC4dC4BP}]}\cdots
\end{array}\right)$$

$$-k^{+}_{\mathrm{C3dgC4bCR1}}[\mathrm{hC3dgC4b}][\mathrm{CR1}]+k^{-}_{\mathrm{C3dgC4bCR1}}[\mathrm{hC3dgC4bCR1}]$$

$$+\left(\frac{k^{FI}_{catC3bH}[I][hC3dgC4bCR1]}{K^{FI}_{mC3bH}+[C3(H_2O)H]+[C3(H_2O)HL]+[fC3bH]+[fC3bHL]+[fC3bCR1]+[fiC3bCR1]+[IgGC3bC3bH]+[IgGC3biC3bH]+[IgGC3bC3bHL]}\cdots\right.$$

$$\frac{1}{+[IgGC3biC3bHL]+[IgGC3bC3bCR1]+[IgGC3biC3bCR1]+[IgGiC3biC3bCR1]+[IgGiC3bC3dgCR1]+[fC3bC4bH]+[fC3bC4bHL]}\cdots$$

$$\frac{1}{+[fC3bC4bCR1]+[fC3bC4bC4BP]+[fC3bC4dH]+[fC3bC4dHL]+[fC3bC4dCR1]+[fiC3bC4dCR1]+[fiC3bC4bCR1]+[fiC3bC4bC4BP]}\cdots$$

$$\frac{1}{+[fC3dgC4bCR1]+[fC3dgC4bC4BP]+[fC3bC3bH]+[fC3bC3bHL]+[fC3bC3bCR1]+[fC3biC3bH]+[fC3biC3bHL]+[fC3biC3bCR1]}\cdots$$

$$\frac{1}{+[fiC3biC3bCR1]+[fiC3bC3dgCR1]+[hC3bH]+[hC3bHL]+[hC3bCR1]+[hiC3bCR1]+[hC3bC4bH]+[hC3bC4bHL]+[hC3bC4bCR1]}\cdots$$

$$\frac{1}{+[hC3bC4bC4BP]+[hC3bC4dH]+[hC3bC4dHL]+[hC3bC4dCR1]+[hiC3bC4dCR1]+[hiC3bC4bCR1]+[hiC3bC4bC4BP]+[hC3dgC4bCR1]}\cdots$$

$$\frac{1}{+[hC3dgC4bC4BP]+[hC3bC3bH]+[hC3bC3bHL]+[hC3bC3bCR1]+[hC3biC3bH]+[hC3biC3bHL]+[hC3biC3bCR1]+[hiC3biC3bCR1]}\cdots$$

$$\frac{1}{+[hiC3bC3dgCR1]+[fC4bCR1]+[nfC4bC4BP]+[fC4bC4BP]+[fC4bC4bCR1]+[fC4bC4bC4BP]+[fC4bC4dCR1]+[fC4bC4dC4BP]+[hC4bCR1]}\cdots$$

$$\left.\frac{1}{+[hC4bC4BP]+[hC4bC4bCR1]+[hC4bC4bC4BP]+[hC4bC4dCR1]+[hC4bC4dC4BP]}\cdots\right)$$

$$-k^{+}_{iC3bC4dCR1}[hiC3bC4d][CR1]+k^{-}_{iC3bC4dCR1}[hiC3bC4dCR1]$$

$$+\left(\frac{k^{FI}_{catC3bH}[I][hiC3bC4dCR1]}{K^{FI}_{mC3bH}+[C3(H_2O)H]+[C3(H_2O)HL]+[fC3bH]+[fC3bHL]+[fC3bCR1]+[fiC3bCR1]+[IgGC3bC3bH]+[IgGC3biC3bH]+[IgGC3bC3bHL]}\cdots\right.$$

$$\frac{1}{+[IgGC3biC3bHL]+[IgGC3bC3bCR1]+[IgGC3biC3bCR1]+[IgGiC3biC3bCR1]+[IgGiC3bC3dgCR1]+[fC3bC4bH]+[fC3bC4bHL]}\cdots$$

$$\frac{1}{+[fC3bC4bCR1]+[fC3bC4bC4BP]+[fC3bC4dH]+[fC3bC4dHL]+[fC3bC4dCR1]+[fiC3bC4dCR1]+[fiC3bC4bCR1]+[fiC3bC4bC4BP]}\cdots$$

$$\frac{1}{+[fC3dgC4bCR1]+[fC3dgC4bC4BP]+[fC3bC3bH]+[fC3bC3bHL]+[fC3bC3bCR1]+[fC3biC3bH]+[fC3biC3bHL]+[fC3biC3bCR1]}\cdots$$

$$\frac{1}{+[fiC3biC3bCR1]+[fiC3bC3dgCR1]+[hC3bH]+[hC3bHL]+[hC3bCR1]+[hiC3bCR1]+[hC3bC4bH]+[hC3bC4bHL]+[hC3bC4bCR1]}\cdots$$

$$\frac{1}{+[hC3bC4bC4BP]+[hC3bC4dH]+[hC3bC4dHL]+[hC3bC4dCR1]+[hiC3bC4dCR1]+[hiC3bC4bCR1]+[hiC3bC4bC4BP]+[hC3dgC4bCR1]}\cdots$$

$$\frac{1}{+[hC3dgC4bC4BP]+[hC3bC3bH]+[hC3bC3bHL]+[hC3bC3bCR1]+[hC3biC3bH]+[hC3biC3bHL]+[hC3biC3bCR1]+[hiC3biC3bCR1]}\cdots$$

$$\frac{1}{+[hiC3bC3dgCR1]+[fC4bCR1]+[nfC4bC4BP]+[fC4bC4BP]+[fC4bC4bCR1]+[fC4bC4bC4BP]+[fC4bC4dCR1]+[fC4bC4dC4BP]+[hC4bCR1]}\cdots$$

$$\left.\frac{1}{+[hC4bC4BP]+[hC4bC4bCR1]+[hC4bC4bC4BP]+[hC4bC4dCR1]+[hC4bC4dC4BP]}\cdots\right)$$

$$-k^{+}_{C3bC4bCR1}[hC3bC4bBb][CR1]+k^{-}_{C3bC4bCR1}[hC3bC4bBbCR1]+k^{-}_{C3bBbCR1_{decay}}[hC3bC4bBbCR1]-k^{+}_{C3bC3bCR1}[hC3bC3b][CR1]$$

$$+k^{-}_{C3bC3bCR1}[hC3bC3bCR1]$$

$$
+\left(\begin{array}{l}
\dfrac{k_{\mathrm{catC3bH}}^{\mathrm{FI}}[\mathrm{I}][\mathrm{hC3bC3bCR1}]}{K_{\mathrm{mC3bH}}^{\mathrm{FI}}+[\mathrm{C3(H_2O)H}]+[\mathrm{C3(H_2O)HL}]+[\mathrm{fC3bH}]+[\mathrm{fC3bHL}]+[\mathrm{fC3bCR1}]+[\mathrm{fiC3bCR1}]+[\mathrm{IgGC3bC3bH}]+[\mathrm{IgGC3biC3bH}]+[\mathrm{IgGC3bC3bHL}]}\cdots \\
\dfrac{1}{+[\mathrm{IgGC3biC3bHL}]+[\mathrm{IgGC3bC3bCR1}]+[\mathrm{IgGC3biC3bCR1}]+[\mathrm{IgGiC3biC3bCR1}]+[\mathrm{IgGiC3bC3dgCR1}]+[\mathrm{fC3bC4bH}]+[\mathrm{fC3bC4bHL}]}\cdots \\
\dfrac{1}{+[\mathrm{fC3bC4bCR1}]+[\mathrm{fC3bC4bC4BP}]+[\mathrm{fC3bC4dH}]+[\mathrm{fC3bC4dHL}]+[\mathrm{fC3bC4dCR1}]+[\mathrm{fiC3bC4dCR1}]+[\mathrm{fiC3bC4bCR1}]+[\mathrm{fiC3bC4bC4BP}]}\cdots \\
\dfrac{1}{+[\mathrm{fC3dgC4bCR1}]+[\mathrm{fC3dgC4bC4BP}]+[\mathrm{fC3bC3bH}]+[\mathrm{fC3bC3bHL}]+[\mathrm{fC3bC3bCR1}]+[\mathrm{fC3biC3bH}]+[\mathrm{fC3biC3bHL}]+[\mathrm{fC3biC3bCR1}]}\cdots \\
\dfrac{1}{+[\mathrm{fiC3biC3bCR1}]+[\mathrm{fiC3bC3dgCR1}]+[\mathrm{hC3bH}]+[\mathrm{hC3bHL}]+[\mathrm{hC3bCR1}]+[\mathrm{hiC3bCR1}]+[\mathrm{hC3bC4bH}]+[\mathrm{hC3bC4bHL}]+[\mathrm{hC3bC4bCR1}]}\cdots \\
\dfrac{1}{+[\mathrm{hC3bC4bC4BP}]+[\mathrm{hC3bC4dH}]+[\mathrm{hC3bC4dHL}]+[\mathrm{hC3bC4dCR1}]+[\mathrm{hiC3bC4dCR1}]+[\mathrm{hiC3bC4bCR1}]+[\mathrm{hiC3bC4bC4BP}]+[\mathrm{hC3dgC4bCR1}]}\cdots \\
\dfrac{1}{+[\mathrm{hC3dgC4bC4BP}]+[\mathrm{hC3bC3bH}]+[\mathrm{hC3bC3bHL}]+[\mathrm{hC3bC3bCR1}]+[\mathrm{hC3biC3bH}]+[\mathrm{hC3biC3bHL}]+[\mathrm{hC3biC3bCR1}]+[\mathrm{hiC3biC3bCR1}]}\cdots \\
\dfrac{1}{+[\mathrm{hiC3bC3dgCR1}]+[\mathrm{fC4bCR1}]+[\mathrm{nfC4bC4BP}]+[\mathrm{fC4bC4BP}]+[\mathrm{fC4bC4bCR1}]+[\mathrm{fC4bC4bC4BP}]+[\mathrm{fC4bC4dCR1}]+[\mathrm{fC4bC4dC4BP}]+[\mathrm{hC4bCR1}]}\cdots \\
\dfrac{1}{+[\mathrm{hC4bC4BP}]+[\mathrm{hC4bC4bCR1}]+[\mathrm{hC4bC4bC4BP}]+[\mathrm{hC4bC4dCR1}]+[\mathrm{hC4bC4dC4BP}]}\cdots
\end{array}\right)
$$

$$
-k_{\mathrm{C3biC3bCR1}}^{+}[\mathrm{hC3biC3b}][\mathrm{CR1}]+k_{\mathrm{C3biC3bCR1}}^{-}[\mathrm{hC3biC3bCR1}]
$$

$$
+\left(\begin{array}{l}
\dfrac{k_{\mathrm{catC3bH}}^{\mathrm{FI}}[\mathrm{I}][\mathrm{hC3biC3bCR1}]}{K_{\mathrm{mC3bH}}^{\mathrm{FI}}+[\mathrm{C3(H_2O)H}]+[\mathrm{C3(H_2O)HL}]+[\mathrm{fC3bH}]+[\mathrm{fC3bHL}]+[\mathrm{fC3bCR1}]+[\mathrm{fiC3bCR1}]+[\mathrm{IgGC3bC3bH}]+[\mathrm{IgGC3biC3bH}]+[\mathrm{IgGC3bC3bHL}]}\cdots \\
\dfrac{1}{+[\mathrm{IgGC3biC3bHL}]+[\mathrm{IgGC3bC3bCR1}]+[\mathrm{IgGC3biC3bCR1}]+[\mathrm{IgGiC3biC3bCR1}]+[\mathrm{IgGiC3bC3dgCR1}]+[\mathrm{fC3bC4bH}]+[\mathrm{fC3bC4bHL}]}\cdots \\
\dfrac{1}{+[\mathrm{fC3bC4bCR1}]+[\mathrm{fC3bC4bC4BP}]+[\mathrm{fC3bC4dH}]+[\mathrm{fC3bC4dHL}]+[\mathrm{fC3bC4dCR1}]+[\mathrm{fiC3bC4dCR1}]+[\mathrm{fiC3bC4bCR1}]+[\mathrm{fiC3bC4bC4BP}]}\cdots \\
\dfrac{1}{+[\mathrm{fC3dgC4bCR1}]+[\mathrm{fC3dgC4bC4BP}]+[\mathrm{fC3bC3bH}]+[\mathrm{fC3bC3bHL}]+[\mathrm{fC3bC3bCR1}]+[\mathrm{fC3biC3bH}]+[\mathrm{fC3biC3bHL}]+[\mathrm{fC3biC3bCR1}]}\cdots \\
\dfrac{1}{+[\mathrm{fiC3biC3bCR1}]+[\mathrm{fiC3bC3dgCR1}]+[\mathrm{hC3bH}]+[\mathrm{hC3bHL}]+[\mathrm{hC3bCR1}]+[\mathrm{hiC3bCR1}]+[\mathrm{hC3bC4bH}]+[\mathrm{hC3bC4bHL}]+[\mathrm{hC3bC4bCR1}]}\cdots \\
\dfrac{1}{+[\mathrm{hC3bC4bC4BP}]+[\mathrm{hC3bC4dH}]+[\mathrm{hC3bC4dHL}]+[\mathrm{hC3bC4dCR1}]+[\mathrm{hiC3bC4dCR1}]+[\mathrm{hiC3bC4bCR1}]+[\mathrm{hiC3bC4bC4BP}]+[\mathrm{hC3dgC4bCR1}]}\cdots \\
\dfrac{1}{+[\mathrm{hC3dgC4bC4BP}]+[\mathrm{hC3bC3bH}]+[\mathrm{hC3bC3bHL}]+[\mathrm{hC3bC3bCR1}]+[\mathrm{hC3biC3bH}]+[\mathrm{hC3biC3bHL}]+[\mathrm{hC3biC3bCR1}]+[\mathrm{hiC3biC3bCR1}]}\cdots \\
\dfrac{1}{+[\mathrm{hiC3bC3dgCR1}]+[\mathrm{fC4bCR1}]+[\mathrm{nfC4bC4BP}]+[\mathrm{fC4bC4BP}]+[\mathrm{fC4bC4bCR1}]+[\mathrm{fC4bC4bC4BP}]+[\mathrm{fC4bC4dCR1}]+[\mathrm{fC4bC4dC4BP}]+[\mathrm{hC4bCR1}]}\cdots \\
\dfrac{1}{+[\mathrm{hC4bC4BP}]+[\mathrm{hC4bC4bCR1}]+[\mathrm{hC4bC4bC4BP}]+[\mathrm{hC4bC4dCR1}]+[\mathrm{hC4bC4dC4BP}]}\cdots
\end{array}\right)
$$

$$
-k_{\mathrm{iC3biC3bCR1}}^{+}[\mathrm{hiC3biC3b}][\mathrm{CR1}]+k_{\mathrm{iC3biC3bCR1}}^{-}[\mathrm{hiC3biC3bCR1}]
$$

$$+\left(\frac{k^{FI}_{catC3bH}[I][hiC3biC3bCR1]}{K^{FI}_{mC3bH}+[C3(H_2O)H]+[C3(H_2O)HL]+[fC3bH]+[fC3bHL]+[fC3bCR1]+[fiC3bCR1]+[IgGC3bC3bH]+[IgGC3biC3bH]+[IgGC3bC3bHL]}\cdots \right.$$

$$\frac{1}{+[IgGC3biC3bHL]+[IgGC3bC3bCR1]+[IgGC3biC3bCR1]+[IgGiC3biC3bCR1]+[IgGiC3bC3dgCR1]+[fC3bC4bH]+[fC3bC4bHL]}\cdots$$

$$\frac{1}{+[fC3bC4bCR1]+[fC3bC4bC4BP]+[fC3bC4dH]+[fC3bC4dHL]+[fC3bC4dCR1]+[fiC3bC4dCR1]+[fiC3bC4bCR1]+[fiC3bC4bC4BP]}\cdots$$

$$\frac{1}{+[fC3dgC4bCR1]+[fC3dgC4bC4BP]+[fC3bC3bH]+[fC3bC3bHL]+[fC3bC3bCR1]+[fC3biC3bH]+[fC3biC3bHL]+[fC3biC3bCR1]}\cdots$$

$$\frac{1}{+[fiC3biC3bCR1]+[fiC3bC3dgCR1]+[hC3bH]+[hC3bHL]+[hC3bCR1]+[hiC3bCR1]+[hC3bC4bH]+[hC3bC4bHL]+[hC3bC4bCR1]}\cdots$$

$$\frac{1}{+[hC3bC4bC4BP]+[hC3bC4dH]+[hC3bC4dHL]+[hC3bC4dCR1]+[hiC3bC4dCR1]+[hiC3bC4bCR1]+[hiC3bC4bC4BP]+[hC3dgC4bCR1]}\cdots$$

$$\frac{1}{+[hC3dgC4bC4BP]+[hC3bC3bH]+[hC3bC3bHL]+[hC3bC3bCR1]+[hC3biC3bH]+[hC3biC3bHL]+[hC3biC3bCR1]+[hiC3biC3bCR1]}\cdots$$

$$\frac{1}{+[hiC3bC3dgCR1]+[fC4bCR1]+[nfC4bC4BP]+[fC4bC4BP]+[fC4bC4bCR1]+[fC4bC4bC4BP]+[fC4bC4dCR1]+[fC4bC4dC4BP]+[hC4bCR1]}\cdots$$

$$\left.\frac{1}{+[hC4bC4BP]+[hC4bC4bCR1]+[hC4bC4bC4BP]+[hC4bC4dCR1]+[hC4bC4dC4BP]}\cdots\right)$$

$$-k^{+}_{iC3bC3dgCR1}[hiC3bC3dg][CR1]+k^{-}_{iC3bC3dgCR1}[hiC3bC3dgCR1]$$

$$+\left(\frac{k^{FI}_{catC3bH}[I][hiC3bC3dgCR1]}{K^{FI}_{mC3bH}+[C3(H_2O)H]+[C3(H_2O)HL]+[fC3bH]+[fC3bHL]+[fC3bCR1]+[fiC3bCR1]+[IgGC3bC3bH]+[IgGC3biC3bH]+[IgGC3bC3bHL]}\cdots \right.$$

$$\frac{1}{+[IgGC3biC3bHL]+[IgGC3bC3bCR1]+[IgGC3biC3bCR1]+[IgGiC3biC3bCR1]+[IgGiC3bC3dgCR1]+[fC3bC4bH]+[fC3bC4bHL]}\cdots$$

$$\frac{1}{+[fC3bC4bCR1]+[fC3bC4bC4BP]+[fC3bC4dH]+[fC3bC4dHL]+[fC3bC4dCR1]+[fiC3bC4dCR1]+[fiC3bC4bCR1]+[fiC3bC4bC4BP]}\cdots$$

$$\frac{1}{+[fC3dgC4bCR1]+[fC3dgC4bC4BP]+[fC3bC3bH]+[fC3bC3bHL]+[fC3bC3bCR1]+[fC3biC3bH]+[fC3biC3bHL]+[fC3biC3bCR1]}\cdots$$

$$\frac{1}{+[fiC3biC3bCR1]+[fiC3bC3dgCR1]+[hC3bH]+[hC3bHL]+[hC3bCR1]+[hiC3bCR1]+[hC3bC4bH]+[hC3bC4bHL]+[hC3bC4bCR1]}\cdots$$

$$\frac{1}{+[hC3bC4bC4BP]+[hC3bC4dH]+[hC3bC4dHL]+[hC3bC4dCR1]+[hiC3bC4dCR1]+[hiC3bC4bCR1]+[hiC3bC4bC4BP]+[hC3dgC4bCR1]}\cdots$$

$$\frac{1}{+[hC3dgC4bC4BP]+[hC3bC3bH]+[hC3bC3bHL]+[hC3bC3bCR1]+[hC3biC3bH]+[hC3biC3bHL]+[hC3biC3bCR1]+[hiC3biC3bCR1]}\cdots$$

$$\frac{1}{+[hiC3bC3dgCR1]+[fC4bCR1]+[nfC4bC4BP]+[fC4bC4BP]+[fC4bC4bCR1]+[fC4bC4bC4BP]+[fC4bC4dCR1]+[fC4bC4dC4BP]+[hC4bCR1]}\cdots$$

$$\left.\frac{1}{+[hC4bC4BP]+[hC4bC4bCR1]+[hC4bC4bC4BP]+[hC4bC4dCR1]+[hC4bC4dC4BP]}\cdots\right)$$

$$-k^{+}_{C3bC3bCR1}[hC3bC3bBb][CR1]+k^{-}_{C3bC3bCR1}[hC3bC3bBbCR1]+k^{-}_{C3bBbCR1_{decay}}[hC3bC3bBbCR1]-k^{+}_{C4bCR1}[hC4b][CR1]$$

$$+k^{-}_{C4bCR1}[hC4bCR1]$$

$$
+\left(\begin{array}{l}
\dfrac{k_{\mathrm{catC3bH}}^{\mathrm{FI}}[\mathrm{I}][\mathrm{hC4bCR1}]}{K_{\mathrm{mC3bH}}^{\mathrm{FI}}+[\mathrm{C3(H_2O)H}]+[\mathrm{C3(H_2O)HL}]+[\mathrm{fC3bH}]+[\mathrm{fC3bHL}]+[\mathrm{fC3bCR1}]+[\mathrm{fiC3bCR1}]+[\mathrm{IgGC3bC3bH}]+[\mathrm{IgGC3biC3bH}]+[\mathrm{IgGC3bC3bHL}]}\cdots \\
\dfrac{1}{+[\mathrm{IgGC3biC3bHL}]+[\mathrm{IgGC3bC3bCR1}]+[\mathrm{IgGC3biC3bCR1}]+[\mathrm{IgGiC3biC3bCR1}]+[\mathrm{IgGiC3bC3dgCR1}]+[\mathrm{fC3bC4bH}]+[\mathrm{fC3bC4bHL}]}\cdots \\
\dfrac{1}{+[\mathrm{fC3bC4bCR1}]+[\mathrm{fC3bC4bC4BP}]+[\mathrm{fC3bC4dH}]+[\mathrm{fC3bC4dHL}]+[\mathrm{fC3bC4dCR1}]+[\mathrm{fiC3bC4dCR1}]+[\mathrm{fiC3bC4bCR1}]+[\mathrm{fiC3bC4bC4BP}]}\cdots \\
\dfrac{1}{+[\mathrm{fC3dgC4bCR1}]+[\mathrm{fC3dgC4bC4BP}]+[\mathrm{fC3bC3bH}]+[\mathrm{fC3bC3bHL}]+[\mathrm{fC3bC3bCR1}]+[\mathrm{fC3biC3bH}]+[\mathrm{fC3biC3bHL}]+[\mathrm{fC3biC3bCR1}]}\cdots \\
\dfrac{1}{+[\mathrm{fiC3biC3bCR1}]+[\mathrm{fiC3bC3dgCR1}]+[\mathrm{hC3bH}]+[\mathrm{hC3bHL}]+[\mathrm{hC3bCR1}]+[\mathrm{hiC3bCR1}]+[\mathrm{hC3bC4bH}]+[\mathrm{hC3bC4bHL}]+[\mathrm{hC3bC4bCR1}]}\cdots \\
\dfrac{1}{+[\mathrm{hC3bC4bC4BP}]+[\mathrm{hC3bC4dH}]+[\mathrm{hC3bC4dHL}]+[\mathrm{hC3bC4dCR1}]+[\mathrm{hiC3bC4dCR1}]+[\mathrm{hiC3bC4bCR1}]+[\mathrm{hiC3bC4bC4BP}]+[\mathrm{hC3dgC4bCR1}]}\cdots \\
\dfrac{1}{+[\mathrm{hC3dgC4bC4BP}]+[\mathrm{hC3bC3bH}]+[\mathrm{hC3bC3bHL}]+[\mathrm{hC3bC3bCR1}]+[\mathrm{hC3biC3bH}]+[\mathrm{hC3biC3bHL}]+[\mathrm{hC3biC3bCR1}]+[\mathrm{hiC3biC3bCR1}]}\cdots \\
\dfrac{1}{+[\mathrm{hiC3bC3dgCR1}]+[\mathrm{fC4bCR1}]+[\mathrm{nfC4bC4BP}]+[\mathrm{fC4bC4BP}]+[\mathrm{fC4bC4bCR1}]+[\mathrm{fC4bC4bC4BP}]+[\mathrm{fC4bC4dCR1}]+[\mathrm{fC4bC4dC4BP}]+[\mathrm{hC4bCR1}]}\cdots \\
\dfrac{1}{+[\mathrm{hC4bC4BP}]+[\mathrm{hC4bC4bCR1}]+[\mathrm{hC4bC4bC4BP}]+[\mathrm{hC4bC4dCR1}]+[\mathrm{hC4bC4dC4BP}]}\cdots
\end{array}\right)
$$

$$
-k_{\mathrm{C4bC4bCR1}}^{+}[\mathrm{hC4bC4b}][\mathrm{CR1}]+k_{\mathrm{C4bC4bCR1}}^{-}[\mathrm{hC4bC4bCR1}]
$$

$$
+\left(\begin{array}{l}
\dfrac{k_{\mathrm{catC3bH}}^{\mathrm{FI}}[\mathrm{I}][\mathrm{hC4bC4bCR1}]}{K_{\mathrm{mC3bH}}^{\mathrm{FI}}+[\mathrm{C3(H_2O)H}]+[\mathrm{C3(H_2O)HL}]+[\mathrm{fC3bH}]+[\mathrm{fC3bHL}]+[\mathrm{fC3bCR1}]+[\mathrm{fiC3bCR1}]+[\mathrm{IgGC3bC3bH}]+[\mathrm{IgGC3biC3bH}]+[\mathrm{IgGC3bC3bHL}]}\cdots \\
\dfrac{1}{+[\mathrm{IgGC3biC3bHL}]+[\mathrm{IgGC3bC3bCR1}]+[\mathrm{IgGC3biC3bCR1}]+[\mathrm{IgGiC3biC3bCR1}]+[\mathrm{IgGiC3bC3dgCR1}]+[\mathrm{fC3bC4bH}]+[\mathrm{fC3bC4bHL}]}\cdots \\
\dfrac{1}{+[\mathrm{fC3bC4bCR1}]+[\mathrm{fC3bC4bC4BP}]+[\mathrm{fC3bC4dH}]+[\mathrm{fC3bC4dHL}]+[\mathrm{fC3bC4dCR1}]+[\mathrm{fiC3bC4dCR1}]+[\mathrm{fiC3bC4bCR1}]+[\mathrm{fiC3bC4bC4BP}]}\cdots \\
\dfrac{1}{+[\mathrm{fC3dgC4bCR1}]+[\mathrm{fC3dgC4bC4BP}]+[\mathrm{fC3bC3bH}]+[\mathrm{fC3bC3bHL}]+[\mathrm{fC3bC3bCR1}]+[\mathrm{fC3biC3bH}]+[\mathrm{fC3biC3bHL}]+[\mathrm{fC3biC3bCR1}]}\cdots \\
\dfrac{1}{+[\mathrm{fiC3biC3bCR1}]+[\mathrm{fiC3bC3dgCR1}]+[\mathrm{hC3bH}]+[\mathrm{hC3bHL}]+[\mathrm{hC3bCR1}]+[\mathrm{hiC3bCR1}]+[\mathrm{hC3bC4bH}]+[\mathrm{hC3bC4bHL}]+[\mathrm{hC3bC4bCR1}]}\cdots \\
\dfrac{1}{+[\mathrm{hC3bC4bC4BP}]+[\mathrm{hC3bC4dH}]+[\mathrm{hC3bC4dHL}]+[\mathrm{hC3bC4dCR1}]+[\mathrm{hiC3bC4dCR1}]+[\mathrm{hiC3bC4bCR1}]+[\mathrm{hiC3bC4bC4BP}]+[\mathrm{hC3dgC4bCR1}]}\cdots \\
\dfrac{1}{+[\mathrm{hC3dgC4bC4BP}]+[\mathrm{hC3bC3bH}]+[\mathrm{hC3bC3bHL}]+[\mathrm{hC3bC3bCR1}]+[\mathrm{hC3biC3bH}]+[\mathrm{hC3biC3bHL}]+[\mathrm{hC3biC3bCR1}]+[\mathrm{hiC3biC3bCR1}]}\cdots \\
\dfrac{1}{+[\mathrm{hiC3bC3dgCR1}]+[\mathrm{fC4bCR1}]+[\mathrm{nfC4bC4BP}]+[\mathrm{fC4bC4BP}]+[\mathrm{fC4bC4bCR1}]+[\mathrm{fC4bC4bC4BP}]+[\mathrm{fC4bC4dCR1}]+[\mathrm{fC4bC4dC4BP}]+[\mathrm{hC4bCR1}]}\cdots \\
\dfrac{1}{+[\mathrm{hC4bC4BP}]+[\mathrm{hC4bC4bCR1}]+[\mathrm{hC4bC4bC4BP}]+[\mathrm{hC4bC4dCR1}]+[\mathrm{hC4bC4dC4BP}]}\cdots
\end{array}\right)
$$

$$
-k_{\mathrm{C4bC4dCR1}}^{+}[\mathrm{hC4bC4d}][\mathrm{CR1}]+k_{\mathrm{C4bC4dCR1}}^{-}[\mathrm{hC4bC4dCR1}]
$$

$$+\left(\frac{k_{\text{catC3bH}}^{\text{FI}}[\text{I}][\text{hC4bC4dCR1}]}{K_{\text{mC3bH}}^{\text{FI}}+[\text{C3(H}_2\text{O)H}]+[\text{C3(H}_2\text{O)HL}]+[\text{fC3bH}]+[\text{fC3bHL}]+[\text{fC3bCR1}]+[\text{fiC3bCR1}]+[\text{IgGC3bC3bH}]+[\text{IgGC3biC3bH}]+[\text{IgGC3bC3bHL}]}\cdots \right.$$

$$\frac{1}{+[\text{IgGC3biC3bHL}]+[\text{IgGC3bC3bCR1}]+[\text{IgGC3biC3bCR1}]+[\text{IgGiC3biC3bCR1}]+[\text{IgGiC3bC3dgCR1}]+[\text{fC3bC4bH}]+[\text{fC3bC4bHL}]}\cdots$$

$$\frac{1}{+[\text{fC3bC4bCR1}]+[\text{fC3bC4bC4BP}]+[\text{fC3bC4dH}]+[\text{fC3bC4dHL}]+[\text{fC3bC4dCR1}]+[\text{fiC3bC4dCR1}]+[\text{fiC3bC4bCR1}]+[\text{fiC3bC4bC4BP}]}\cdots$$

$$\frac{1}{+[\text{fC3dgC4bCR1}]+[\text{fC3dgC4bC4BP}]+[\text{fC3bC3bH}]+[\text{fC3bC3bHL}]+[\text{fC3bC3bCR1}]+[\text{fC3biC3bH}]+[\text{fC3biC3bHL}]+[\text{fC3biC3bCR1}]}\cdots$$

$$\frac{1}{+[\text{fiC3biC3bCR1}]+[\text{fiC3bC3dgCR1}]+[\text{hC3bH}]+[\text{hC3bHL}]+[\text{hC3bCR1}]+[\text{hiC3bCR1}]+[\text{hC3bC4bH}]+[\text{hC3bC4bHL}]+[\text{hC3bC4bCR1}]}\cdots$$

$$\frac{1}{+[\text{hC3bC4bC4BP}]+[\text{hC3bC4dH}]+[\text{hC3bC4dHL}]+[\text{hC3bC4dCR1}]+[\text{hiC3bC4dCR1}]+[\text{hiC3bC4bCR1}]+[\text{hiC3bC4bC4BP}]+[\text{hC3dgC4bCR1}]}\cdots$$

$$\frac{1}{+[\text{hC3dgC4bC4BP}]+[\text{hC3bC3bH}]+[\text{hC3bC3bHL}]+[\text{hC3bC3bCR1}]+[\text{hC3biC3bH}]+[\text{hC3biC3bHL}]+[\text{hC3biC3bCR1}]+[\text{hiC3biC3bCR1}]}\cdots$$

$$\frac{1}{+[\text{hiC3bC3dgCR1}]+[\text{fC4bCR1}]+[\text{nfC4bC4BP}]+[\text{fC4bC4BP}]+[\text{fC4bC4bCR1}]+[\text{fC4bC4bC4BP}]+[\text{fC4bC4dCR1}]+[\text{fC4bC4dC4BP}]+[\text{hC4bCR1}]}\cdots$$

$$\left.\frac{1}{+[\text{hC4bC4BP}]+[\text{hC4bC4bCR1}]+[\text{hC4bC4bC4BP}]+[\text{hC4bC4dCR1}]+[\text{hC4bC4dC4BP}]}\cdots\right)$$

S290

$$\frac{d[\text{C4BP}]}{dt}=-k_{\text{C4bC4BP}}^{+}[\text{fC3bC4b}][\text{C4BP}]+k_{\text{C4bC4BP}}^{-}[\text{fC3bC4bC4BP}]$$

$$+\left(\frac{k_{\text{catC3bH}}^{\text{FI}}[\text{I}][\text{fC3bC4bC4BP}]}{K_{\text{mC3bH}}^{\text{FI}}+[\text{C3(H}_2\text{O)H}]+[\text{C3(H}_2\text{O)HL}]+[\text{fC3bH}]+[\text{fC3bHL}]+[\text{fC3bCR1}]+[\text{fiC3bCR1}]+[\text{IgGC3bC3bH}]+[\text{IgGC3biC3bH}]+[\text{IgGC3bC3bHL}]}\cdots \right.$$

$$\frac{1}{+[\text{IgGC3biC3bHL}]+[\text{IgGC3bC3bCR1}]+[\text{IgGC3biC3bCR1}]+[\text{IgGiC3biC3bCR1}]+[\text{IgGiC3bC3dgCR1}]+[\text{fC3bC4bH}]+[\text{fC3bC4bHL}]}\cdots$$

$$\frac{1}{+[\text{fC3bC4bCR1}]+[\text{fC3bC4bC4BP}]+[\text{fC3bC4dH}]+[\text{fC3bC4dHL}]+[\text{fC3bC4dCR1}]+[\text{fiC3bC4dCR1}]+[\text{fiC3bC4bCR1}]+[\text{fiC3bC4bC4BP}]}\cdots$$

$$\frac{1}{+[\text{fC3dgC4bCR1}]+[\text{fC3dgC4bC4BP}]+[\text{fC3bC3bH}]+[\text{fC3bC3bHL}]+[\text{fC3bC3bCR1}]+[\text{fC3biC3bH}]+[\text{fC3biC3bHL}]+[\text{fC3biC3bCR1}]}\cdots$$

$$\frac{1}{+[\text{fiC3biC3bCR1}]+[\text{fiC3bC3dgCR1}]+[\text{hC3bH}]+[\text{hC3bHL}]+[\text{hC3bCR1}]+[\text{hiC3bCR1}]+[\text{hC3bC4bH}]+[\text{hC3bC4bHL}]+[\text{hC3bC4bCR1}]}\cdots$$

$$\frac{1}{+[\text{hC3bC4bC4BP}]+[\text{hC3bC4dH}]+[\text{hC3bC4dHL}]+[\text{hC3bC4dCR1}]+[\text{hiC3bC4dCR1}]+[\text{hiC3bC4bCR1}]+[\text{hiC3bC4bC4BP}]+[\text{hC3dgC4bCR1}]}\cdots$$

$$\frac{1}{+[\text{hC3dgC4bC4BP}]+[\text{hC3bC3bH}]+[\text{hC3bC3bHL}]+[\text{hC3bC3bCR1}]+[\text{hC3biC3bH}]+[\text{hC3biC3bHL}]+[\text{hC3biC3bCR1}]+[\text{hiC3biC3bCR1}]}\cdots$$

$$\frac{1}{+[\text{hiC3bC3dgCR1}]+[\text{fC4bCR1}]+[\text{nfC4bC4BP}]+[\text{fC4bC4BP}]+[\text{fC4bC4bCR1}]+[\text{fC4bC4bC4BP}]+[\text{fC4bC4dCR1}]+[\text{fC4bC4dC4BP}]+[\text{hC4bCR1}]}\cdots$$

$$\left.\frac{1}{+[\text{hC4bC4BP}]+[\text{hC4bC4bCR1}]+[\text{hC4bC4bC4BP}]+[\text{hC4bC4dCR1}]+[\text{hC4bC4dC4BP}]}\cdots\right)$$

$$-k_{\text{C4bC4BP}}^{+}[\text{fiC3bC4b}][\text{C4BP}]+k_{\text{C4bC4BP}}^{-}[\text{fiC3bC4bC4BP}]$$

$$+\left(\frac{k^{\mathrm{FI}}_{\mathrm{catC3bH}}[\mathrm{I}][\mathrm{fiC3bC4bC4BP}]}{K^{\mathrm{FI}}_{\mathrm{mC3bH}}+[\mathrm{C3(H_2O)H}]+[\mathrm{C3(H_2O)HL}]+[\mathrm{fC3bH}]+[\mathrm{fC3bHL}]+[\mathrm{fC3bCR1}]+[\mathrm{fiC3bCR1}]+[\mathrm{IgGC3bC3bH}]+[\mathrm{IgGC3biC3bH}]+[\mathrm{IgGC3bC3bHL}]}\cdots\right.$$

$$\frac{1}{+[\mathrm{IgGC3biC3bHL}]+[\mathrm{IgGC3bC3bCR1}]+[\mathrm{IgGC3biC3bCR1}]+[\mathrm{IgGiC3biC3bCR1}]+[\mathrm{IgGiC3bC3dgCR1}]+[\mathrm{fC3bC4bH}]+[\mathrm{fC3bC4bHL}]}\cdots$$

$$\frac{1}{+[\mathrm{fC3bC4bCR1}]+[\mathrm{fC3bC4bC4BP}]+[\mathrm{fC3bC4dH}]+[\mathrm{fC3bC4dHL}]+[\mathrm{fC3bC4dCR1}]+[\mathrm{fiC3bC4dCR1}]+[\mathrm{fiC3bC4bCR1}]+[\mathrm{fiC3bC4bC4BP}]}\cdots$$

$$\frac{1}{+[\mathrm{fC3dgC4bCR1}]+[\mathrm{fC3dgC4bC4BP}]+[\mathrm{fC3bC3bH}]+[\mathrm{fC3bC3bHL}]+[\mathrm{fC3bC3bCR1}]+[\mathrm{fC3biC3bH}]+[\mathrm{fC3biC3bHL}]+[\mathrm{fC3biC3bCR1}]}\cdots$$

$$\frac{1}{+[\mathrm{fiC3biC3bCR1}]+[\mathrm{fiC3bC3dgCR1}]+[\mathrm{hC3bH}]+[\mathrm{hC3bHL}]+[\mathrm{hC3bCR1}]+[\mathrm{hiC3bCR1}]+[\mathrm{hC3bC4bH}]+[\mathrm{hC3bC4bHL}]+[\mathrm{hC3bC4bCR1}]}\cdots$$

$$\frac{1}{+[\mathrm{hC3bC4bC4BP}]+[\mathrm{hC3bC4dH}]+[\mathrm{hC3bC4dHL}]+[\mathrm{hC3bC4dCR1}]+[\mathrm{hiC3bC4dCR1}]+[\mathrm{hiC3bC4bCR1}]+[\mathrm{hiC3bC4bC4BP}]+[\mathrm{hC3dgC4bCR1}]}\cdots$$

$$\frac{1}{+[\mathrm{hC3dgC4bC4BP}]+[\mathrm{hC3bC3bH}]+[\mathrm{hC3bC3bHL}]+[\mathrm{hC3bC3bCR1}]+[\mathrm{hC3biC3bH}]+[\mathrm{hC3biC3bHL}]+[\mathrm{hC3biC3bCR1}]+[\mathrm{hiC3biC3bCR1}]}\cdots$$

$$\frac{1}{+[\mathrm{hiC3bC3dgCR1}]+[\mathrm{fC4bCR1}]+[\mathrm{nfC4bC4BP}]+[\mathrm{fC4bC4BP}]+[\mathrm{fC4bC4bCR1}]+[\mathrm{fC4bC4bC4BP}]+[\mathrm{fC4bC4dCR1}]+[\mathrm{fC4bC4dC4BP}]+[\mathrm{hC4bCR1}]}\cdots$$

$$\left.\frac{1}{+[\mathrm{hC4bC4BP}]+[\mathrm{hC4bC4bCR1}]+[\mathrm{hC4bC4bC4BP}]+[\mathrm{hC4bC4dCR1}]+[\mathrm{hC4bC4dC4BP}]}\cdots\right)$$

$$-k^{+}_{\mathrm{C4bC4BP}}[\mathrm{fC3dgC4b}][\mathrm{C4BP}]+k^{-}_{\mathrm{C4bC4BP}}[\mathrm{fC3dgC4bC4BP}]$$

$$+\left(\frac{k^{\mathrm{FI}}_{\mathrm{catC3bH}}[\mathrm{I}][\mathrm{fC3dgC4bC4BP}]}{K^{\mathrm{FI}}_{\mathrm{mC3bH}}+[\mathrm{C3(H_2O)H}]+[\mathrm{C3(H_2O)HL}]+[\mathrm{fC3bH}]+[\mathrm{fC3bHL}]+[\mathrm{fC3bCR1}]+[\mathrm{fiC3bCR1}]+[\mathrm{IgGC3bC3bH}]+[\mathrm{IgGC3biC3bH}]+[\mathrm{IgGC3bC3bHL}]}\cdots\right.$$

$$\frac{1}{+[\mathrm{IgGC3biC3bHL}]+[\mathrm{IgGC3bC3bCR1}]+[\mathrm{IgGC3biC3bCR1}]+[\mathrm{IgGiC3biC3bCR1}]+[\mathrm{IgGiC3bC3dgCR1}]+[\mathrm{fC3bC4bH}]+[\mathrm{fC3bC4bHL}]}\cdots$$

$$\frac{1}{+[\mathrm{fC3bC4bCR1}]+[\mathrm{fC3bC4bC4BP}]+[\mathrm{fC3bC4dH}]+[\mathrm{fC3bC4dHL}]+[\mathrm{fC3bC4dCR1}]+[\mathrm{fiC3bC4dCR1}]+[\mathrm{fiC3bC4bCR1}]+[\mathrm{fiC3bC4bC4BP}]}\cdots$$

$$\frac{1}{+[\mathrm{fC3dgC4bCR1}]+[\mathrm{fC3dgC4bC4BP}]+[\mathrm{fC3bC3bH}]+[\mathrm{fC3bC3bHL}]+[\mathrm{fC3bC3bCR1}]+[\mathrm{fC3biC3bH}]+[\mathrm{fC3biC3bHL}]+[\mathrm{fC3biC3bCR1}]}\cdots$$

$$\frac{1}{+[\mathrm{fiC3biC3bCR1}]+[\mathrm{fiC3bC3dgCR1}]+[\mathrm{hC3bH}]+[\mathrm{hC3bHL}]+[\mathrm{hC3bCR1}]+[\mathrm{hiC3bCR1}]+[\mathrm{hC3bC4bH}]+[\mathrm{hC3bC4bHL}]+[\mathrm{hC3bC4bCR1}]}\cdots$$

$$\frac{1}{+[\mathrm{hC3bC4bC4BP}]+[\mathrm{hC3bC4dH}]+[\mathrm{hC3bC4dHL}]+[\mathrm{hC3bC4dCR1}]+[\mathrm{hiC3bC4dCR1}]+[\mathrm{hiC3bC4bCR1}]+[\mathrm{hiC3bC4bC4BP}]+[\mathrm{hC3dgC4bCR1}]}\cdots$$

$$\frac{1}{+[\mathrm{hC3dgC4bC4BP}]+[\mathrm{hC3bC3bH}]+[\mathrm{hC3bC3bHL}]+[\mathrm{hC3bC3bCR1}]+[\mathrm{hC3biC3bH}]+[\mathrm{hC3biC3bHL}]+[\mathrm{hC3biC3bCR1}]+[\mathrm{hiC3biC3bCR1}]}\cdots$$

$$\frac{1}{+[\mathrm{hiC3bC3dgCR1}]+[\mathrm{fC4bCR1}]+[\mathrm{nfC4bC4BP}]+[\mathrm{fC4bC4BP}]+[\mathrm{fC4bC4bCR1}]+[\mathrm{fC4bC4bC4BP}]+[\mathrm{fC4bC4dCR1}]+[\mathrm{fC4bC4dC4BP}]+[\mathrm{hC4bCR1}]}\cdots$$

$$\left.\frac{1}{+[\mathrm{hC4bC4BP}]+[\mathrm{hC4bC4bCR1}]+[\mathrm{hC4bC4bC4BP}]+[\mathrm{hC4bC4dCR1}]+[\mathrm{hC4bC4dC4BP}]}\cdots\right)$$

$$-k^{+}_{\mathrm{C4bC4BP}}[\mathrm{nfC4b}][\mathrm{C4BP}]-k^{+}_{\mathrm{C4bC4BP}}[\mathrm{fC4b}][\mathrm{C4BP}]+k^{-}_{\mathrm{C4bC4BP}}[\mathrm{fC4bC4BP}]$$

$$
+\left(\begin{array}{l}
\frac{k^{FI}_{catC3bH}[\mathrm{I}][\mathrm{fC4bC4BP}]}{K^{FI}_{mC3bH}+[\mathrm{C3(H_2O)H}]+[\mathrm{C3(H_2O)HL}]+[\mathrm{fC3bH}]+[\mathrm{fC3bHL}]+[\mathrm{fC3bCR1}]+[\mathrm{fiC3bCR1}]+[\mathrm{IgGC3bC3bH}]+[\mathrm{IgGC3biC3bH}]+[\mathrm{IgGC3bC3bHL}]}\cdots \\
\frac{1}{+[\mathrm{IgGC3biC3bHL}]+[\mathrm{IgGC3bC3bCR1}]+[\mathrm{IgGC3biC3bCR1}]+[\mathrm{IgGiC3biC3bCR1}]+[\mathrm{IgGiC3bC3dgCR1}]+[\mathrm{fC3bC4bH}]+[\mathrm{fC3bC4bHL}]}\cdots \\
\frac{1}{+[\mathrm{fC3bC4bCR1}]+[\mathrm{fC3bC4bC4BP}]+[\mathrm{fC3bC4dH}]+[\mathrm{fC3bC4dHL}]+[\mathrm{fC3bC4dCR1}]+[\mathrm{fiC3bC4dCR1}]+[\mathrm{fiC3bC4bCR1}]+[\mathrm{fiC3bC4bC4BP}]}\cdots \\
\frac{1}{+[\mathrm{fC3dgC4bCR1}]+[\mathrm{fC3dgC4bC4BP}]+[\mathrm{fC3bC3bH}]+[\mathrm{fC3bC3bHL}]+[\mathrm{fC3bC3bCR1}]+[\mathrm{fC3biC3bH}]+[\mathrm{fC3biC3bHL}]+[\mathrm{fC3biC3bCR1}]}\cdots \\
\frac{1}{+[\mathrm{fiC3biC3bCR1}]+[\mathrm{fiC3bC3dgCR1}]+[\mathrm{hC3bH}]+[\mathrm{hC3bHL}]+[\mathrm{hC3bCR1}]+[\mathrm{hiC3bCR1}]+[\mathrm{hC3bC4bH}]+[\mathrm{hC3bC4bHL}]+[\mathrm{hC3bC4bCR1}]}\cdots \\
\frac{1}{+[\mathrm{hC3bC4bC4BP}]+[\mathrm{hC3bC4dH}]+[\mathrm{hC3bC4dHL}]+[\mathrm{hC3bC4dCR1}]+[\mathrm{hiC3bC4dCR1}]+[\mathrm{hiC3bC4bCR1}]+[\mathrm{hiC3bC4bC4BP}]+[\mathrm{hC3dgC4bCR1}]}\cdots \\
\frac{1}{+[\mathrm{hC3dgC4bC4BP}]+[\mathrm{hC3bC3bH}]+[\mathrm{hC3bC3bHL}]+[\mathrm{hC3bC3bCR1}]+[\mathrm{hC3biC3bH}]+[\mathrm{hC3biC3bHL}]+[\mathrm{hC3biC3bCR1}]+[\mathrm{hiC3biC3bCR1}]}\cdots \\
\frac{1}{+[\mathrm{hiC3bC3dgCR1}]+[\mathrm{fC4bCR1}]+[\mathrm{nfC4bC4BP}]+[\mathrm{fC4bC4BP}]+[\mathrm{fC4bC4bCR1}]+[\mathrm{fC4bC4bC4BP}]+[\mathrm{fC4bC4dCR1}]+[\mathrm{fC4bC4dC4BP}]+[\mathrm{hC4bCR1}]}\cdots \\
\frac{1}{+[\mathrm{hC4bC4BP}]+[\mathrm{hC4bC4bCR1}]+[\mathrm{hC4bC4bC4BP}]+[\mathrm{hC4bC4dCR1}]+[\mathrm{hC4bC4dC4BP}]}\cdots
\end{array}\right)
$$

$$
-k^{+}_{C4bC4BP}[\mathrm{fC4bC2a}][\mathrm{C4BP}]+k^{-}_{C4bC4BP}[\mathrm{fC4bC2aC4BP}]+k^{-}_{C4bC2aC4BP_{decay}}[\mathrm{fC4bC2aC4BP}]-k^{+}_{C4bC4BP}[\mathrm{fC4bC4bC2a}][\mathrm{C4BP}]
$$

$$
+k^{-}_{C4bC4BP}[\mathrm{fC4bC4bC2aC4BP}]+k^{-}_{C4bC2aC4BP_{decay}}[\mathrm{fC4bC4bC2aC4BP}]-k^{+}_{C4bC4BP}[\mathrm{fC3bC4bC2a}][\mathrm{C4BP}]+k^{-}_{C4bC4BP}[\mathrm{fC3bC4bC2aC4BP}]
$$

$$
+k^{-}_{C4bC2aC4BP_{decay}}[\mathrm{fC3bC4bC2aC4BP}]-k^{+}_{C4bC4BP}[\mathrm{fC4bC4b}][\mathrm{C4BP}]+k^{-}_{C4bC4BP}[\mathrm{fC4bC4bC4BP}]
$$

$$
+\left(\begin{array}{l}
\frac{k^{FI}_{catC3bH}[\mathrm{I}][\mathrm{fC4bC4bC4BP}]}{K^{FI}_{mC3bH}+[\mathrm{C3(H_2O)H}]+[\mathrm{C3(H_2O)HL}]+[\mathrm{fC3bH}]+[\mathrm{fC3bHL}]+[\mathrm{fC3bCR1}]+[\mathrm{fiC3bCR1}]+[\mathrm{IgGC3bC3bH}]+[\mathrm{IgGC3biC3bH}]+[\mathrm{IgGC3bC3bHL}]}\cdots \\
\frac{1}{+[\mathrm{IgGC3biC3bHL}]+[\mathrm{IgGC3bC3bCR1}]+[\mathrm{IgGC3biC3bCR1}]+[\mathrm{IgGiC3biC3bCR1}]+[\mathrm{IgGiC3bC3dgCR1}]+[\mathrm{fC3bC4bH}]+[\mathrm{fC3bC4bHL}]}\cdots \\
\frac{1}{+[\mathrm{fC3bC4bCR1}]+[\mathrm{fC3bC4bC4BP}]+[\mathrm{fC3bC4dH}]+[\mathrm{fC3bC4dHL}]+[\mathrm{fC3bC4dCR1}]+[\mathrm{fiC3bC4dCR1}]+[\mathrm{fiC3bC4bCR1}]+[\mathrm{fiC3bC4bC4BP}]}\cdots \\
\frac{1}{+[\mathrm{fC3dgC4bCR1}]+[\mathrm{fC3dgC4bC4BP}]+[\mathrm{fC3bC3bH}]+[\mathrm{fC3bC3bHL}]+[\mathrm{fC3bC3bCR1}]+[\mathrm{fC3biC3bH}]+[\mathrm{fC3biC3bHL}]+[\mathrm{fC3biC3bCR1}]}\cdots \\
\frac{1}{+[\mathrm{fiC3biC3bCR1}]+[\mathrm{fiC3bC3dgCR1}]+[\mathrm{hC3bH}]+[\mathrm{hC3bHL}]+[\mathrm{hC3bCR1}]+[\mathrm{hiC3bCR1}]+[\mathrm{hC3bC4bH}]+[\mathrm{hC3bC4bHL}]+[\mathrm{hC3bC4bCR1}]}\cdots \\
\frac{1}{+[\mathrm{hC3bC4bC4BP}]+[\mathrm{hC3bC4dH}]+[\mathrm{hC3bC4dHL}]+[\mathrm{hC3bC4dCR1}]+[\mathrm{hiC3bC4dCR1}]+[\mathrm{hiC3bC4bCR1}]+[\mathrm{hiC3bC4bC4BP}]+[\mathrm{hC3dgC4bCR1}]}\cdots \\
\frac{1}{+[\mathrm{hC3dgC4bC4BP}]+[\mathrm{hC3bC3bH}]+[\mathrm{hC3bC3bHL}]+[\mathrm{hC3bC3bCR1}]+[\mathrm{hC3biC3bH}]+[\mathrm{hC3biC3bHL}]+[\mathrm{hC3biC3bCR1}]+[\mathrm{hiC3biC3bCR1}]}\cdots \\
\frac{1}{+[\mathrm{hiC3bC3dgCR1}]+[\mathrm{fC4bCR1}]+[\mathrm{nfC4bC4BP}]+[\mathrm{fC4bC4BP}]+[\mathrm{fC4bC4bCR1}]+[\mathrm{fC4bC4bC4BP}]+[\mathrm{fC4bC4dCR1}]+[\mathrm{fC4bC4dC4BP}]+[\mathrm{hC4bCR1}]}\cdots \\
\frac{1}{+[\mathrm{hC4bC4BP}]+[\mathrm{hC4bC4bCR1}]+[\mathrm{hC4bC4bC4BP}]+[\mathrm{hC4bC4dCR1}]+[\mathrm{hC4bC4dC4BP}]}\cdots
\end{array}\right)
$$

$$- k^{+}_{C4bC4BP}[fC4bC4d][C4BP] + k^{-}_{C4bC4BP}[fC4bC4dC4BP]$$

$$+\left(\frac{k^{FI}_{catC3bH}[I][fC4bC4dC4BP]}{K^{FI}_{mC3bH} + [C3(H_2O)H] + [C3(H_2O)HL] + [fC3bH] + [fC3bHL] + [fC3bCR1] + [fiC3bCR1] + [IgGC3bC3bH] + [IgGC3biC3bH] + [IgGC3bC3bHL]} \cdots \right.$$

$$\frac{1}{+ [IgGC3biC3bHL] + [IgGC3bC3bCR1] + [IgGC3biC3bCR1] + [IgGiC3biC3bCR1] + [IgGiC3bC3dgCR1] + [fC3bC4bH] + [fC3bC4bHL]} \cdots$$

$$\frac{1}{+ [fC3bC4bCR1] + [fC3bC4bC4BP] + [fC3bC4dH] + [fC3bC4dHL] + [fC3bC4dCR1] + [fiC3bC4dCR1] + [fiC3bC4bCR1] + [fiC3bC4bC4BP]} \cdots$$

$$\frac{1}{+ [fC3dgC4bCR1] + [fC3dgC4bC4BP] + [fC3bC3bH] + [fC3bC3bHL] + [fC3bC3bCR1] + [fC3biC3bH] + [fC3biC3bHL] + [fC3biC3bCR1]} \cdots$$

$$\frac{1}{+ [fiC3biC3bCR1] + [fiC3bC3dgCR1] + [hC3bH] + [hC3bHL] + [hC3bCR1] + [hiC3bCR1] + [hC3bC4bH] + [hC3bC4bHL] + [hC3bC4bCR1]} \cdots$$

$$\frac{1}{+ [hC3bC4bC4BP] + [hC3bC4dH] + [hC3bC4dHL] + [hC3bC4dCR1] + [hiC3bC4dCR1] + [hiC3bC4bCR1] + [hiC3bC4bC4BP] + [hC3dgC4bCR1]} \cdots$$

$$\frac{1}{+ [hC3dgC4bC4BP] + [hC3bC3bH] + [hC3bC3bHL] + [hC3bC3bCR1] + [hC3biC3bH] + [hC3biC3bHL] + [hC3biC3bCR1] + [hiC3biC3bCR1]} \cdots$$

$$\frac{1}{+ [hiC3bC3dgCR1] + [fC4bCR1] + [nfC4bC4BP] + [fC4bC4BP] + [fC4bC4bCR1] + [fC4bC4bC4BP] + [fC4bC4dCR1] + [fC4bC4dC4BP] + [hC4bCR1]} \cdots$$

$$\left. \frac{1}{+ [hC4bC4BP] + [hC4bC4bCR1] + [hC4bC4bC4BP] + [hC4bC4dCR1] + [hC4bC4dC4BP]} \cdots \right)$$

$$- k^{+}_{C4bC4BP}[hC3bC4b][C4BP] + k^{-}_{C4bC4BP}[hC3bC4bC4BP]$$

$$+\left(\frac{k^{FI}_{catC3bH}[I][hC3bC4bC4BP]}{K^{FI}_{mC3bH} + [C3(H_2O)H] + [C3(H_2O)HL] + [fC3bH] + [fC3bHL] + [fC3bCR1] + [fiC3bCR1] + [IgGC3bC3bH] + [IgGC3biC3bH] + [IgGC3bC3bHL]} \cdots \right.$$

$$\frac{1}{+ [IgGC3biC3bHL] + [IgGC3bC3bCR1] + [IgGC3biC3bCR1] + [IgGiC3biC3bCR1] + [IgGiC3bC3dgCR1] + [fC3bC4bH] + [fC3bC4bHL]} \cdots$$

$$\frac{1}{+ [fC3bC4bCR1] + [fC3bC4bC4BP] + [fC3bC4dH] + [fC3bC4dHL] + [fC3bC4dCR1] + [fiC3bC4dCR1] + [fiC3bC4bCR1] + [fiC3bC4bC4BP]} \cdots$$

$$\frac{1}{+ [fC3dgC4bCR1] + [fC3dgC4bC4BP] + [fC3bC3bH] + [fC3bC3bHL] + [fC3bC3bCR1] + [fC3biC3bH] + [fC3biC3bHL] + [fC3biC3bCR1]} \cdots$$

$$\frac{1}{+ [fiC3biC3bCR1] + [fiC3bC3dgCR1] + [hC3bH] + [hC3bHL] + [hC3bCR1] + [hiC3bCR1] + [hC3bC4bH] + [hC3bC4bHL] + [hC3bC4bCR1]} \cdots$$

$$\frac{1}{+ [hC3bC4bC4BP] + [hC3bC4dH] + [hC3bC4dHL] + [hC3bC4dCR1] + [hiC3bC4dCR1] + [hiC3bC4bCR1] + [hiC3bC4bC4BP] + [hC3dgC4bCR1]} \cdots$$

$$\frac{1}{+ [hC3dgC4bC4BP] + [hC3bC3bH] + [hC3bC3bHL] + [hC3bC3bCR1] + [hC3biC3bH] + [hC3biC3bHL] + [hC3biC3bCR1] + [hiC3biC3bCR1]} \cdots$$

$$\frac{1}{+ [hiC3bC3dgCR1] + [fC4bCR1] + [nfC4bC4BP] + [fC4bC4BP] + [fC4bC4bCR1] + [fC4bC4bC4BP] + [fC4bC4dCR1] + [fC4bC4dC4BP] + [hC4bCR1]} \cdots$$

$$\left. \frac{1}{+ [hC4bC4BP] + [hC4bC4bCR1] + [hC4bC4bC4BP] + [hC4bC4dCR1] + [hC4bC4dC4BP]} \cdots \right)$$

$$- k^{+}_{C4bC4BP}[hiC3bC4b][C4BP] + k^{-}_{C4bC4BP}[hiC3bC4bC4BP]$$

$$
+\left(\begin{array}{l}
\dfrac{k^{\mathrm{FI}}_{\mathrm{catC3bH}}[\mathrm{I}][\mathrm{hiC3bC4bC4BP}]}{K^{\mathrm{FI}}_{\mathrm{mC3bH}}+[\mathrm{C3(H_2O)H}]+[\mathrm{C3(H_2O)HL}]+[\mathrm{fC3bH}]+[\mathrm{fC3bHL}]+[\mathrm{fC3bCR1}]+[\mathrm{fiC3bCR1}]+[\mathrm{IgGC3bC3bH}]+[\mathrm{IgGC3biC3bH}]+[\mathrm{IgGC3bC3bHL}]}\cdots \\
\dfrac{1}{+[\mathrm{IgGC3biC3bHL}]+[\mathrm{IgGC3bC3bCR1}]+[\mathrm{IgGC3biC3bCR1}]+[\mathrm{IgGiC3biC3bCR1}]+[\mathrm{IgGiC3bC3dgCR1}]+[\mathrm{fC3bC4bH}]+[\mathrm{fC3bC4bHL}]}\cdots \\
\dfrac{1}{+[\mathrm{fC3bC4bCR1}]+[\mathrm{fC3bC4bC4BP}]+[\mathrm{fC3bC4dH}]+[\mathrm{fC3bC4dHL}]+[\mathrm{fC3bC4dCR1}]+[\mathrm{fiC3bC4dCR1}]+[\mathrm{fiC3bC4bCR1}]+[\mathrm{fiC3bC4bC4BP}]}\cdots \\
\dfrac{1}{+[\mathrm{fC3dgC4bCR1}]+[\mathrm{fC3dgC4bC4BP}]+[\mathrm{fC3bC3bH}]+[\mathrm{fC3bC3bHL}]+[\mathrm{fC3bC3bCR1}]+[\mathrm{fC3biC3bH}]+[\mathrm{fC3biC3bHL}]+[\mathrm{fC3biC3bCR1}]}\cdots \\
\dfrac{1}{+[\mathrm{fiC3biC3bCR1}]+[\mathrm{fiC3bC3dgCR1}]+[\mathrm{hC3bH}]+[\mathrm{hC3bHL}]+[\mathrm{hC3bCR1}]+[\mathrm{hiC3bCR1}]+[\mathrm{hC3bC4bH}]+[\mathrm{hC3bC4bHL}]+[\mathrm{hC3bC4bCR1}]}\cdots \\
\dfrac{1}{+[\mathrm{hC3bC4bC4BP}]+[\mathrm{hC3bC4dH}]+[\mathrm{hC3bC4dHL}]+[\mathrm{hC3bC4dCR1}]+[\mathrm{hiC3bC4dCR1}]+[\mathrm{hiC3bC4bCR1}]+[\mathrm{hiC3bC4bC4BP}]+[\mathrm{hC3dgC4bCR1}]}\cdots \\
\dfrac{1}{+[\mathrm{hC3dgC4bC4BP}]+[\mathrm{hC3bC3bH}]+[\mathrm{hC3bC3bHL}]+[\mathrm{hC3bC3bCR1}]+[\mathrm{hC3biC3bH}]+[\mathrm{hC3biC3bHL}]+[\mathrm{hC3biC3bCR1}]+[\mathrm{hiC3biC3bCR1}]}\cdots \\
\dfrac{1}{+[\mathrm{hiC3bC3dgCR1}]+[\mathrm{fC4bCR1}]+[\mathrm{nfC4bC4BP}]+[\mathrm{fC4bC4BP}]+[\mathrm{fC4bC4bCR1}]+[\mathrm{fC4bC4bC4BP}]+[\mathrm{fC4bC4dCR1}]+[\mathrm{fC4bC4dC4BP}]+[\mathrm{hC4bCR1}]}\cdots \\
\dfrac{1}{+[\mathrm{hC4bC4BP}]+[\mathrm{hC4bC4bCR1}]+[\mathrm{hC4bC4bC4BP}]+[\mathrm{hC4bC4dCR1}]+[\mathrm{hC4bC4dC4BP}]}\cdots
\end{array}\right)
$$

$$
-k^{+}_{\mathrm{C4bC4BP}}[\mathrm{hC3dgC4b}][\mathrm{C4BP}]+k^{-}_{\mathrm{C4bC4BP}}[\mathrm{hC3dgC4bC4BP}]
$$

$$
+\left(\begin{array}{l}
\dfrac{k^{\mathrm{FI}}_{\mathrm{catC3bH}}[\mathrm{I}][\mathrm{hC3dgC4bC4BP}]}{K^{\mathrm{FI}}_{\mathrm{mC3bH}}+[\mathrm{C3(H_2O)H}]+[\mathrm{C3(H_2O)HL}]+[\mathrm{fC3bH}]+[\mathrm{fC3bHL}]+[\mathrm{fC3bCR1}]+[\mathrm{fiC3bCR1}]+[\mathrm{IgGC3bC3bH}]+[\mathrm{IgGC3biC3bH}]+[\mathrm{IgGC3bC3bHL}]}\cdots \\
\dfrac{1}{+[\mathrm{IgGC3biC3bHL}]+[\mathrm{IgGC3bC3bCR1}]+[\mathrm{IgGC3biC3bCR1}]+[\mathrm{IgGiC3biC3bCR1}]+[\mathrm{IgGiC3bC3dgCR1}]+[\mathrm{fC3bC4bH}]+[\mathrm{fC3bC4bHL}]}\cdots \\
\dfrac{1}{+[\mathrm{fC3bC4bCR1}]+[\mathrm{fC3bC4bC4BP}]+[\mathrm{fC3bC4dH}]+[\mathrm{fC3bC4dHL}]+[\mathrm{fC3bC4dCR1}]+[\mathrm{fiC3bC4dCR1}]+[\mathrm{fiC3bC4bCR1}]+[\mathrm{fiC3bC4bC4BP}]}\cdots \\
\dfrac{1}{+[\mathrm{fC3dgC4bCR1}]+[\mathrm{fC3dgC4bC4BP}]+[\mathrm{fC3bC3bH}]+[\mathrm{fC3bC3bHL}]+[\mathrm{fC3bC3bCR1}]+[\mathrm{fC3biC3bH}]+[\mathrm{fC3biC3bHL}]+[\mathrm{fC3biC3bCR1}]}\cdots \\
\dfrac{1}{+[\mathrm{fiC3biC3bCR1}]+[\mathrm{fiC3bC3dgCR1}]+[\mathrm{hC3bH}]+[\mathrm{hC3bHL}]+[\mathrm{hC3bCR1}]+[\mathrm{hiC3bCR1}]+[\mathrm{hC3bC4bH}]+[\mathrm{hC3bC4bHL}]+[\mathrm{hC3bC4bCR1}]}\cdots \\
\dfrac{1}{+[\mathrm{hC3bC4bC4BP}]+[\mathrm{hC3bC4dH}]+[\mathrm{hC3bC4dHL}]+[\mathrm{hC3bC4dCR1}]+[\mathrm{hiC3bC4dCR1}]+[\mathrm{hiC3bC4bCR1}]+[\mathrm{hiC3bC4bC4BP}]+[\mathrm{hC3dgC4bCR1}]}\cdots \\
\dfrac{1}{+[\mathrm{hC3dgC4bC4BP}]+[\mathrm{hC3bC3bH}]+[\mathrm{hC3bC3bHL}]+[\mathrm{hC3bC3bCR1}]+[\mathrm{hC3biC3bH}]+[\mathrm{hC3biC3bHL}]+[\mathrm{hC3biC3bCR1}]+[\mathrm{hiC3biC3bCR1}]}\cdots \\
\dfrac{1}{+[\mathrm{hiC3bC3dgCR1}]+[\mathrm{fC4bCR1}]+[\mathrm{nfC4bC4BP}]+[\mathrm{fC4bC4BP}]+[\mathrm{fC4bC4bCR1}]+[\mathrm{fC4bC4bC4BP}]+[\mathrm{fC4bC4dCR1}]+[\mathrm{fC4bC4dC4BP}]+[\mathrm{hC4bCR1}]}\cdots \\
\dfrac{1}{+[\mathrm{hC4bC4BP}]+[\mathrm{hC4bC4bCR1}]+[\mathrm{hC4bC4bC4BP}]+[\mathrm{hC4bC4dCR1}]+[\mathrm{hC4bC4dC4BP}]}\cdots
\end{array}\right)
$$

$$
-k^{+}_{\mathrm{C4bC4BP}}[\mathrm{hC4b}][\mathrm{C4BP}]+k^{-}_{\mathrm{C4bC4BP}}[\mathrm{hC4bC4BP}]
$$

$$+\left(\frac{k_{\mathrm{catC3bH}}^{\mathrm{FI}}[\mathrm{I}][\mathrm{hC4bC4BP}]}{K_{\mathrm{mC3bH}}^{\mathrm{FI}}+[\mathrm{C3(H_2O)H}]+[\mathrm{C3(H_2O)HL}]+[\mathrm{fC3bH}]+[\mathrm{fC3bHL}]+[\mathrm{fC3bCR1}]+[\mathrm{fiC3bCR1}]+[\mathrm{IgGC3bC3bH}]+[\mathrm{IgGC3biC3bH}]+[\mathrm{IgGC3bC3bHL}]}\cdots\right.$$
$$\frac{1}{+[\mathrm{IgGC3biC3bHL}]+[\mathrm{IgGC3bC3bCR1}]+[\mathrm{IgGC3biC3bCR1}]+[\mathrm{IgGiC3biC3bCR1}]+[\mathrm{IgGiC3bC3dgCR1}]+[\mathrm{fC3bC4bH}]+[\mathrm{fC3bC4bHL}]}\cdots$$
$$\frac{1}{+[\mathrm{fC3bC4bCR1}]+[\mathrm{fC3bC4bC4BP}]+[\mathrm{fC3bC4dH}]+[\mathrm{fC3bC4dHL}]+[\mathrm{fC3bC4dCR1}]+[\mathrm{fiC3bC4dCR1}]+[\mathrm{fiC3bC4bCR1}]+[\mathrm{fiC3bC4bC4BP}]}\cdots$$
$$\frac{1}{+[\mathrm{fC3dgC4bCR1}]+[\mathrm{fC3dgC4bC4BP}]+[\mathrm{fC3bC3bH}]+[\mathrm{fC3bC3bHL}]+[\mathrm{fC3bC3bCR1}]+[\mathrm{fC3biC3bH}]+[\mathrm{fC3biC3bHL}]+[\mathrm{fC3biC3bCR1}]}\cdots$$
$$\frac{1}{+[\mathrm{fiC3biC3bCR1}]+[\mathrm{fiC3bC3dgCR1}]+[\mathrm{hC3bH}]+[\mathrm{hC3bHL}]+[\mathrm{hC3bCR1}]+[\mathrm{hiC3bCR1}]+[\mathrm{hC3bC4bH}]+[\mathrm{hC3bC4bHL}]+[\mathrm{hC3bC4bCR1}]}\cdots$$
$$\frac{1}{+[\mathrm{hC3bC4bC4BP}]+[\mathrm{hC3bC4dH}]+[\mathrm{hC3bC4dHL}]+[\mathrm{hC3bC4dCR1}]+[\mathrm{hiC3bC4dCR1}]+[\mathrm{hiC3bC4bCR1}]+[\mathrm{hiC3bC4bC4BP}]+[\mathrm{hC3dgC4bCR1}]}\cdots$$
$$\frac{1}{+[\mathrm{hC3dgC4bC4BP}]+[\mathrm{hC3bC3bH}]+[\mathrm{hC3bC3bHL}]+[\mathrm{hC3bC3bCR1}]+[\mathrm{hC3biC3bH}]+[\mathrm{hC3biC3bHL}]+[\mathrm{hC3biC3bCR1}]+[\mathrm{hiC3biC3bCR1}]}\cdots$$
$$\frac{1}{+[\mathrm{hiC3bC3dgCR1}]+[\mathrm{fC4bCR1}]+[\mathrm{nfC4bC4BP}]+[\mathrm{fC4bC4BP}]+[\mathrm{fC4bC4bCR1}]+[\mathrm{fC4bC4bC4BP}]+[\mathrm{fC4bC4dCR1}]+[\mathrm{fC4bC4dC4BP}]+[\mathrm{hC4bCR1}]}\cdots$$
$$\left.\frac{1}{+[\mathrm{hC4bC4BP}]+[\mathrm{hC4bC4bCR1}]+[\mathrm{hC4bC4bC4BP}]+[\mathrm{hC4bC4dCR1}]+[\mathrm{hC4bC4dC4BP}]}\cdots\right)$$

$$-k_{\mathrm{C4bC4BP}}^{+}[\mathrm{hC4bC4b}][\mathrm{C4BP}]+k_{\mathrm{C4bC4BP}}^{-}[\mathrm{hC4bC4bC4BP}]$$

$$+\left(\frac{k_{\mathrm{catC3bH}}^{\mathrm{FI}}[\mathrm{I}][\mathrm{hC4bC4bC4BP}]}{K_{\mathrm{mC3bH}}^{\mathrm{FI}}+[\mathrm{C3(H_2O)H}]+[\mathrm{C3(H_2O)HL}]+[\mathrm{fC3bH}]+[\mathrm{fC3bHL}]+[\mathrm{fC3bCR1}]+[\mathrm{fiC3bCR1}]+[\mathrm{IgGC3bC3bH}]+[\mathrm{IgGC3biC3bH}]+[\mathrm{IgGC3bC3bHL}]}\cdots\right.$$
$$\frac{1}{+[\mathrm{IgGC3biC3bHL}]+[\mathrm{IgGC3bC3bCR1}]+[\mathrm{IgGC3biC3bCR1}]+[\mathrm{IgGiC3biC3bCR1}]+[\mathrm{IgGiC3bC3dgCR1}]+[\mathrm{fC3bC4bH}]+[\mathrm{fC3bC4bHL}]}\cdots$$
$$\frac{1}{+[\mathrm{fC3bC4bCR1}]+[\mathrm{fC3bC4bC4BP}]+[\mathrm{fC3bC4dH}]+[\mathrm{fC3bC4dHL}]+[\mathrm{fC3bC4dCR1}]+[\mathrm{fiC3bC4dCR1}]+[\mathrm{fiC3bC4bCR1}]+[\mathrm{fiC3bC4bC4BP}]}\cdots$$
$$\frac{1}{+[\mathrm{fC3dgC4bCR1}]+[\mathrm{fC3dgC4bC4BP}]+[\mathrm{fC3bC3bH}]+[\mathrm{fC3bC3bHL}]+[\mathrm{fC3bC3bCR1}]+[\mathrm{fC3biC3bH}]+[\mathrm{fC3biC3bHL}]+[\mathrm{fC3biC3bCR1}]}\cdots$$
$$\frac{1}{+[\mathrm{fiC3biC3bCR1}]+[\mathrm{fiC3bC3dgCR1}]+[\mathrm{hC3bH}]+[\mathrm{hC3bHL}]+[\mathrm{hC3bCR1}]+[\mathrm{hiC3bCR1}]+[\mathrm{hC3bC4bH}]+[\mathrm{hC3bC4bHL}]+[\mathrm{hC3bC4bCR1}]}\cdots$$
$$\frac{1}{+[\mathrm{hC3bC4bC4BP}]+[\mathrm{hC3bC4dH}]+[\mathrm{hC3bC4dHL}]+[\mathrm{hC3bC4dCR1}]+[\mathrm{hiC3bC4dCR1}]+[\mathrm{hiC3bC4bCR1}]+[\mathrm{hiC3bC4bC4BP}]+[\mathrm{hC3dgC4bCR1}]}\cdots$$
$$\frac{1}{+[\mathrm{hC3dgC4bC4BP}]+[\mathrm{hC3bC3bH}]+[\mathrm{hC3bC3bHL}]+[\mathrm{hC3bC3bCR1}]+[\mathrm{hC3biC3bH}]+[\mathrm{hC3biC3bHL}]+[\mathrm{hC3biC3bCR1}]+[\mathrm{hiC3biC3bCR1}]}\cdots$$
$$\frac{1}{+[\mathrm{hiC3bC3dgCR1}]+[\mathrm{fC4bCR1}]+[\mathrm{nfC4bC4BP}]+[\mathrm{fC4bC4BP}]+[\mathrm{fC4bC4bCR1}]+[\mathrm{fC4bC4bC4BP}]+[\mathrm{fC4bC4dCR1}]+[\mathrm{fC4bC4dC4BP}]+[\mathrm{hC4bCR1}]}\cdots$$
$$\left.\frac{1}{+[\mathrm{hC4bC4BP}]+[\mathrm{hC4bC4bCR1}]+[\mathrm{hC4bC4bC4BP}]+[\mathrm{hC4bC4dCR1}]+[\mathrm{hC4bC4dC4BP}]}\cdots\right)$$

$$-k_{\mathrm{C4bC4BP}}^{+}[\mathrm{hC4bC4d}][\mathrm{C4BP}]+k_{\mathrm{C4bC4BP}}^{-}[\mathrm{hC4bC4dC4BP}]$$

$$
+\left(
\begin{array}{l}
\dfrac{k^{\mathrm{FI}}_{\mathrm{catC3bH}}[\mathrm{I}][\mathrm{hC4bC4dC4BP}]}{K^{\mathrm{FI}}_{\mathrm{mC3bH}}+[\mathrm{C3(H_2O)H}]+[\mathrm{C3(H_2O)HL}]+[\mathrm{fC3bH}]+[\mathrm{fC3bHL}]+[\mathrm{fC3bCR1}]+[\mathrm{fiC3bCR1}]+[\mathrm{IgGC3bC3bH}]+[\mathrm{IgGC3biC3bH}]+[\mathrm{IgGC3bC3bHL}]}\cdots \\
\dfrac{1}{+[\mathrm{IgGC3biC3bHL}]+[\mathrm{IgGC3bC3bCR1}]+[\mathrm{IgGC3biC3bCR1}]+[\mathrm{IgGiC3biC3bCR1}]+[\mathrm{IgGiC3bC3dgCR1}]+[\mathrm{fC3bC4bH}]+[\mathrm{fC3bC4bHL}]}\cdots \\
\dfrac{1}{+[\mathrm{fC3bC4bCR1}]+[\mathrm{fC3bC4bC4BP}]+[\mathrm{fC3bC4dH}]+[\mathrm{fC3bC4dHL}]+[\mathrm{fC3bC4dCR1}]+[\mathrm{fiC3bC4dCR1}]+[\mathrm{fiC3bC4bCR1}]+[\mathrm{fiC3bC4bC4BP}]}\cdots \\
\dfrac{1}{+[\mathrm{fC3dgC4bCR1}]+[\mathrm{fC3dgC4bC4BP}]+[\mathrm{fC3bC3bH}]+[\mathrm{fC3bC3bHL}]+[\mathrm{fC3bC3bCR1}]+[\mathrm{fC3biC3bH}]+[\mathrm{fC3biC3bHL}]+[\mathrm{fC3biC3bCR1}]}\cdots \\
\dfrac{1}{+[\mathrm{fiC3biC3bCR1}]+[\mathrm{fiC3bC3dgCR1}]+[\mathrm{hC3bH}]+[\mathrm{hC3bHL}]+[\mathrm{hC3bCR1}]+[\mathrm{hiC3bCR1}]+[\mathrm{hC3bC4bH}]+[\mathrm{hC3bC4bHL}]+[\mathrm{hC3bC4bCR1}]}\cdots \\
\dfrac{1}{+[\mathrm{hC3bC4bC4BP}]+[\mathrm{hC3bC4dH}]+[\mathrm{hC3bC4dHL}]+[\mathrm{hC3bC4dCR1}]+[\mathrm{hiC3bC4dCR1}]+[\mathrm{hiC3bC4bCR1}]+[\mathrm{hiC3bC4bC4BP}]+[\mathrm{hC3dgC4bCR1}]}\cdots \\
\dfrac{1}{+[\mathrm{hC3dgC4bC4BP}]+[\mathrm{hC3bC3bH}]+[\mathrm{hC3bC3bHL}]+[\mathrm{hC3bC3bCR1}]+[\mathrm{hC3biC3bH}]+[\mathrm{hC3biC3bHL}]+[\mathrm{hC3biC3bCR1}]+[\mathrm{hiC3biC3bCR1}]}\cdots \\
\dfrac{1}{+[\mathrm{hiC3bC3dgCR1}]+[\mathrm{fC4bCR1}]+[\mathrm{nfC4bC4BP}]+[\mathrm{fC4bC4BP}]+[\mathrm{fC4bC4bCR1}]+[\mathrm{fC4bC4bC4BP}]+[\mathrm{fC4bC4dCR1}]+[\mathrm{fC4bC4dC4BP}]+[\mathrm{hC4bCR1}]}\cdots \\
\dfrac{1}{+[\mathrm{hC4bC4BP}]+[\mathrm{hC4bC4bCR1}]+[\mathrm{hC4bC4bC4BP}]+[\mathrm{hC4bC4dCR1}]+[\mathrm{hC4bC4dC4BP}]}\cdots
\end{array}
\right)
$$
