# Supplementary material for: A computational model for the evaluation of complement system regulation under homeostasis, disease, and drug intervention
Source: PLoS One. 2018 Jun 6;13(6):e0198644. doi: 10.1371/journal.pone.0198644 (PMC5991421; doi:10.1371/journal.pone.0198644)
Supplement: S2 Equations — Ordinary differential equations of complement system interactions with compstatin and eculizumab. (PDF) [file pone.0198644.s018.pdf]

S2 Equations. Mathematical models of inhibitor-complement interactions. Ordinary differential equations of complement system interactions with compstatin and eculizumab.

S291

$$\frac{d[\mathrm{C3}]}{dt} = -k^{+}_{\mathrm{C3(H_2O)}}[\mathrm{C3}] - \frac{k^{\mathrm{C3}}_{\mathrm{catC3(H_2O)Bb}}[\mathrm{C3}][\mathrm{C3(H_2O)Bb}]}{K^{\mathrm{C3}}_{\mathrm{mC3(H_2O)Bb}}+[\mathrm{C3}]} - \frac{k^{\mathrm{C3}}_{\mathrm{catC3bBb}}[\mathrm{C3}][\mathrm{fC3bBb}]}{K^{\mathrm{C3}}_{\mathrm{mC3bBb}}\left(1+\frac{[\mathrm{C3}]}{K^{\mathrm{C3}}_{\mathrm{mC3bBb}}}+\frac{[\mathrm{C5}]}{K^{\mathrm{C5}}_{\mathrm{mC3bBb}}}\right)}$$

$$- \frac{k^{\mathrm{C3}}_{\mathrm{catC3bBb}}[\mathrm{C3}][\mathrm{hC3bBb}]}{K^{\mathrm{C3}}_{\mathrm{mC3bBb}}\left(1+\frac{[\mathrm{C3}]}{K^{\mathrm{C3}}_{\mathrm{mC3bBb}}}+\frac{[\mathrm{C5}]}{K^{\mathrm{C5}}_{\mathrm{mC3bBb}}}\right)} - \frac{k^{\mathrm{C3}}_{\mathrm{catC3bBb}}[\mathrm{C3}][\mathrm{hC3bBbP}]}{K^{\mathrm{C3}}_{\mathrm{mC3bBb}}\left(1+\frac{[\mathrm{C3}]}{K^{\mathrm{C3}}_{\mathrm{mC3bBb}}}+\frac{[\mathrm{C5}]}{K^{\mathrm{C5}}_{\mathrm{mC3bBb}}}\right)} - \frac{k^{\mathrm{C3}}_{\mathrm{catC3bBb}}[\mathrm{C3}][\mathrm{IgGC3bC3bBb}]}{K^{\mathrm{C3}}_{\mathrm{mC3bBb}}+[\mathrm{C3}]}$$

$$- \frac{k^{\mathrm{C3}}_{\mathrm{catC3bBb}}[\mathrm{C3}][\mathrm{IgGC3bC3bBbP}]}{K^{\mathrm{C3}}_{\mathrm{mC3bBb}}+[\mathrm{C3}]} - \frac{k^{\mathrm{C3}}_{\mathrm{catC3bBb}}[\mathrm{C3}][\mathrm{fC4bC2a}]}{K^{\mathrm{C3}}_{\mathrm{mC3bBb}}\left(1+\frac{[\mathrm{C3}]}{K^{\mathrm{C3}}_{\mathrm{mC4bC2a}}}+\frac{[\mathrm{C5}]}{K^{\mathrm{C5}}_{\mathrm{mC4bC2a}}}\right)}$$

$$- \frac{k^{\mathrm{C3}}_{\mathrm{catC3bBb}}[\mathrm{C3}][\mathrm{fC3bC3bBb}]}{K^{\mathrm{C3}}_{\mathrm{mC3bBb}}\left(1+\frac{[\mathrm{C3}]}{K^{\mathrm{C3}}_{\mathrm{mC3bBb}}}+\frac{[\mathrm{C5}]}{K^{\mathrm{C5}}_{\mathrm{mC3bC3bBb}}}\right)} - \frac{k^{\mathrm{C3}}_{\mathrm{catC3bBb}}[\mathrm{C3}][\mathrm{fC3bC3bBbP}]}{K^{\mathrm{C3}}_{\mathrm{mC3bBb}}\left(1+\frac{[\mathrm{C3}]}{K^{\mathrm{C3}}_{\mathrm{mC3bBb}}}+\frac{[\mathrm{C5}]}{K^{\mathrm{C5}}_{\mathrm{mC3bC3bBb}}}\right)}$$

$$- \frac{k^{\mathrm{C3}}_{\mathrm{catC3bBb}}[\mathrm{C3}][\mathrm{fC3bC4bBb}]}{K^{\mathrm{C3}}_{\mathrm{mC3bBb}}\left(1+\frac{[\mathrm{C3}]}{K^{\mathrm{C3}}_{\mathrm{mC3bBb}}}+\frac{[\mathrm{C5}]}{K^{\mathrm{C5}}_{\mathrm{mC3bC4bBb}}}\right)} - \frac{k^{\mathrm{C3}}_{\mathrm{catC3bBb}}[\mathrm{C3}][\mathrm{fC3bC4bBbP}]}{K^{\mathrm{C3}}_{\mathrm{mC3bBb}}\left(1+\frac{[\mathrm{C3}]}{K^{\mathrm{C3}}_{\mathrm{mC3bBb}}}+\frac{[\mathrm{C5}]}{K^{\mathrm{C5}}_{\mathrm{mC3bC4bBb}}}\right)}$$

$$- \frac{k^{\mathrm{C3}}_{\mathrm{catC4bC2a}}[\mathrm{C3}][\mathrm{fC3bC4bC2a}]}{K^{\mathrm{C3}}_{\mathrm{mC4bC2a}}\left(1+\frac{[\mathrm{C3}]}{K^{\mathrm{C3}}_{\mathrm{mC4bC2a}}}+\frac{[\mathrm{C5}]}{K^{\mathrm{C5}}_{\mathrm{mC3bC4bC2a}}}\right)} - \frac{k^{\mathrm{C3}}_{\mathrm{catC4bC2a}}[\mathrm{C3}][\mathrm{fC4bC4bC2a}]}{K^{\mathrm{C3}}_{\mathrm{mC4bC2a}}\left(1+\frac{[\mathrm{C3}]}{K^{\mathrm{C3}}_{\mathrm{mC4bC2a}}}+\frac{[\mathrm{C5}]}{K^{\mathrm{C5}}_{\mathrm{mC4bC4bC2a}}}\right)}$$

$$- \frac{k^{\mathrm{C3}}_{\mathrm{catC3bBb}}[\mathrm{C3}][\mathrm{hC3bC3bBb}]}{K^{\mathrm{C3}}_{\mathrm{mC3bBb}}\left(1+\frac{[\mathrm{C3}]}{K^{\mathrm{C3}}_{\mathrm{mC3bBb}}}+\frac{[\mathrm{C5}]}{K^{\mathrm{C5}}_{\mathrm{mC3bC3bBb}}}\right)} - \frac{k^{\mathrm{C3}}_{\mathrm{catC3bBb}}[\mathrm{C3}][\mathrm{hC3bC3bBbP}]}{K^{\mathrm{C3}}_{\mathrm{mC3bBb}}\left(1+\frac{[\mathrm{C3}]}{K^{\mathrm{C3}}_{\mathrm{mC3bBb}}}+\frac{[\mathrm{C5}]}{K^{\mathrm{C5}}_{\mathrm{mC3bC3bBb}}}\right)}$$

$$- \frac{k^{\mathrm{C3}}_{\mathrm{catC3bBb}}[\mathrm{C3}][\mathrm{hC3bC4bBb}]}{K^{\mathrm{C3}}_{\mathrm{mC3bBb}}\left(1+\frac{[\mathrm{C3}]}{K^{\mathrm{C3}}_{\mathrm{mC3bBb}}}+\frac{[\mathrm{C5}]}{K^{\mathrm{C5}}_{\mathrm{mC3bC4bBb}}}\right)} - \frac{k^{\mathrm{C3}}_{\mathrm{catC3bBb}}[\mathrm{C3}][\mathrm{hC3bC4bBbP}]}{K^{\mathrm{C3}}_{\mathrm{mC3bBb}}\left(1+\frac{[\mathrm{C3}]}{K^{\mathrm{C3}}_{\mathrm{mC3bBb}}}+\frac{[\mathrm{C5}]}{K^{\mathrm{C5}}_{\mathrm{mC3bC4bBb}}}\right)}$$

$$- k^{+}_{\mathrm{CompC3}}[\mathrm{C3}][\mathrm{Compstatin}] + k^{-}_{\mathrm{CompC3}}[\mathrm{CompC3}]$$

S292

$$\frac{d[\mathrm{Compstatin}]}{dt}=-k^{+}_{\mathrm{CompC3}}[\mathrm{C3}][\mathrm{Compstatin}]+k^{-}_{\mathrm{CompC3}}[\mathrm{CompC3}]$$

S293

$$\frac{d[\mathrm{CompC3}]}{dt}=k^{+}_{\mathrm{CompC3}}[\mathrm{C3}][\mathrm{Compstatin}]-k^{-}_{\mathrm{CompC3}}[\mathrm{CompC3}]$$

S294

$$\begin{aligned}\frac{d[\mathrm{C5}]}{dt}=&-\frac{k^{\mathrm{C5}}_{\mathrm{catC3bBb}}[\mathrm{C5}][\mathrm{fC3bBb}]}{K^{\mathrm{C5}}_{\mathrm{mC3bBb}}\left(1+\frac{[\mathrm{C3}]}{K^{\mathrm{C3}}_{\mathrm{mC3bBb}}}+\frac{[\mathrm{C5}]}{K^{\mathrm{C5}}_{\mathrm{mC3bBb}}}\right)}-\frac{k^{\mathrm{C5}}_{\mathrm{catC4bC2a}}[\mathrm{C5}][\mathrm{fC4bC2a}]}{K^{\mathrm{C5}}_{\mathrm{mC4bC2a}}\left(1+\frac{[\mathrm{C3}]}{K^{\mathrm{C3}}_{\mathrm{mC4bC2a}}}+\frac{[\mathrm{C5}]}{K^{\mathrm{C5}}_{\mathrm{mC4bC2a}}}\right)}\\
&-\frac{k^{\mathrm{C5}}_{\mathrm{catC3bC3bBb}}[\mathrm{C5}][\mathrm{fC3bC3bBb}]}{K^{\mathrm{C5}}_{\mathrm{mC3bC3bBb}}\left(1+\frac{[\mathrm{C3}]}{K^{\mathrm{C3}}_{\mathrm{mC3bBb}}}+\frac{[\mathrm{C5}]}{K^{\mathrm{C5}}_{\mathrm{mC3bC3bBb}}}\right)}-\frac{k^{\mathrm{C5}}_{\mathrm{catC3bC3bBb}}[\mathrm{C5}][\mathrm{fC3bC3bBbP}]}{K^{\mathrm{C5}}_{\mathrm{mC3bC3bBb}}\left(1+\frac{[\mathrm{C3}]}{K^{\mathrm{C3}}_{\mathrm{mC3bBb}}}+\frac{[\mathrm{C5}]}{K^{\mathrm{C5}}_{\mathrm{mC3bC3bBb}}}\right)}\\
&-\frac{k^{\mathrm{C5}}_{\mathrm{catC3bC4bBb}}[\mathrm{C5}][\mathrm{fC3bC4bBb}]}{K^{\mathrm{C5}}_{\mathrm{mC3bC4bBb}}\left(1+\frac{[\mathrm{C3}]}{K^{\mathrm{C3}}_{\mathrm{mC3bBb}}}+\frac{[\mathrm{C5}]}{K^{\mathrm{C5}}_{\mathrm{mC3bC4bBb}}}\right)}-\frac{k^{\mathrm{C5}}_{\mathrm{catC3bC4bBb}}[\mathrm{C5}][\mathrm{fC3bC4bBbP}]}{K^{\mathrm{C5}}_{\mathrm{mC3bC4bBb}}\left(1+\frac{[\mathrm{C3}]}{K^{\mathrm{C3}}_{\mathrm{mC3bBb}}}+\frac{[\mathrm{C5}]}{K^{\mathrm{C5}}_{\mathrm{mC3bC4bBb}}}\right)}\\
&-\frac{k^{\mathrm{C5}}_{\mathrm{catC3bC4bC2a}}[\mathrm{C5}][\mathrm{fC3bC4bC2a}]}{K^{\mathrm{C5}}_{\mathrm{mC3bC4bC2a}}\left(1+\frac{[\mathrm{C3}]}{K^{\mathrm{C3}}_{\mathrm{mC4bC2a}}}+\frac{[\mathrm{C5}]}{K^{\mathrm{C5}}_{\mathrm{mC3bC4bC2a}}}\right)}-\frac{k^{\mathrm{C5}}_{\mathrm{catC4bC4bC2a}}[\mathrm{C5}][\mathrm{fC4bC4bC2a}]}{K^{\mathrm{C5}}_{\mathrm{mC4bC4bC2a}}\left(1+\frac{[\mathrm{C3}]}{K^{\mathrm{C3}}_{\mathrm{mC4bC2a}}}+\frac{[\mathrm{C5}]}{K^{\mathrm{C5}}_{\mathrm{mC4bC4bC2a}}}\right)}\\
&-\frac{k^{\mathrm{C5}}_{\mathrm{catC3bBb}}[\mathrm{C5}][\mathrm{hC3bBb}]}{K^{\mathrm{C5}}_{\mathrm{mC3bBb}}\left(1+\frac{[\mathrm{C3}]}{K^{\mathrm{C3}}_{\mathrm{mC3bBb}}}+\frac{[\mathrm{C5}]}{K^{\mathrm{C5}}_{\mathrm{mC3bBb}}}\right)}-\frac{k^{\mathrm{C5}}_{\mathrm{catC3bBb}}[\mathrm{C5}][\mathrm{hC3bBbP}]}{K^{\mathrm{C5}}_{\mathrm{mC3bBb}}\left(1+\frac{[\mathrm{C3}]}{K^{\mathrm{C3}}_{\mathrm{mC3bBb}}}+\frac{[\mathrm{C5}]}{K^{\mathrm{C5}}_{\mathrm{mC3bBb}}}\right)}\\
&-\frac{k^{\mathrm{C5}}_{\mathrm{catC3bC3bBb}}[\mathrm{C5}][\mathrm{hC3bC3bBb}]}{K^{\mathrm{C5}}_{\mathrm{mC3bC3bBb}}\left(1+\frac{[\mathrm{C3}]}{K^{\mathrm{C3}}_{\mathrm{mC3bBb}}}+\frac{[\mathrm{C5}]}{K^{\mathrm{C5}}_{\mathrm{mC3bC3bBb}}}\right)}-\frac{k^{\mathrm{C5}}_{\mathrm{catC3bC3bBb}}[\mathrm{C5}][\mathrm{hC3bC3bBbP}]}{K^{\mathrm{C5}}_{\mathrm{mC3bC3bBb}}\left(1+\frac{[\mathrm{C3}]}{K^{\mathrm{C3}}_{\mathrm{mC3bBb}}}+\frac{[\mathrm{C5}]}{K^{\mathrm{C5}}_{\mathrm{mC3bC3bBb}}}\right)}\\
&-\frac{k^{\mathrm{C5}}_{\mathrm{catC3bC4bBb}}[\mathrm{C5}][\mathrm{hC3bC4bBb}]}{K^{\mathrm{C5}}_{\mathrm{mC3bC4bBb}}\left(1+\frac{[\mathrm{C3}]}{K^{\mathrm{C3}}_{\mathrm{mC3bBb}}}+\frac{[\mathrm{C5}]}{K^{\mathrm{C5}}_{\mathrm{mC3bC4bBb}}}\right)}-\frac{k^{\mathrm{C5}}_{\mathrm{catC3bC4bBb}}[\mathrm{C5}][\mathrm{hC3bC4bBbP}]}{K^{\mathrm{C5}}_{\mathrm{mC3bC4bBb}}\left(1+\frac{[\mathrm{C3}]}{K^{\mathrm{C3}}_{\mathrm{mC3bBb}}}+\frac{[\mathrm{C5}]}{K^{\mathrm{C5}}_{\mathrm{mC3bC4bBb}}}\right)}\\
&-k^{+}_{\mathrm{EcuC5}}[\mathrm{C5}][\mathrm{Eculizumab}]+k^{-}_{\mathrm{EcuC5}}[\mathrm{EcuC5}]\end{aligned}$$

S295

$$\frac{d[\mathrm{Eculizumab}]}{dt}=-k^{+}_{\mathrm{EcuC5}}[\mathrm{C5}][\mathrm{Eculizumab}]+k^{-}_{\mathrm{EcuC5}}[\mathrm{EcuC5}]$$

S296

$$\frac{d[\mathrm{EcuC5}]}{dt} = k^{+}_{\mathrm{EcuC5}}[\mathrm{C5}][\mathrm{Eculizumab}] - k^{-}_{\mathrm{EcuC5}}[\mathrm{EcuC5}]$$
